# *In Silico* Analysis Highlights the Diversity and Novelty of Circular Bacteriocins in Sequenced Microbial Genomes

**DOI:** 10.1128/mSystems.00047-20

**Published:** 2020-06-02

**Authors:** Bingyue Xin, Hualin Liu, Jinshui Zheng, Chuanshuai Xie, Ying Gao, Dadong Dai, Donghai Peng, Lifang Ruan, Huanchun Chen, Ming Sun

**Affiliations:** a State Key Laboratory of Agricultural Microbiology, College of Life Science and Technology, Huazhong Agricultural University, Wuhan, China; b College of Life Sciences, Huaibei Normal University, Huaibei, China; c State Key Laboratory of Agricultural Microbiology, College of Veterinary Medicine, Huazhong Agricultural University, Wuhan, China; d Hubei Key Laboratory of Agricultural Bioinformatics, Huazhong Agricultural University, Wuhan, China; University of Pittsburgh Medical Center

**Keywords:** preservatives, circular bacteriocin, *in silico* analysis, *Bacillus cereus* group, cerecyclin

## Abstract

Consumer demand for “fresh food” with no chemical preservatives has prompted researchers to pay more attention to natural antimicrobial peptides such as bacteriocins. Nisin is currently the most widely used food biopreservative among the bacteriocins; however, its applications are restricted due to its low stability at neutral and alkaline pH values. Circular bacteriocins have potent antimicrobial activity against foodborne pathogens, show exceptional stability, and have great potential to be developed as biopreservatives. Here, we take advantage of the precursor peptides of 15 reported circular bacteriocins to devise an *in silico* approach to identify potential circular bacteriocins in sequenced microbial genomes. A total of nearly 7,000 putative precursor peptides were identified from 86 species of bacteria and further classified into 28 groups based on their amino acid similarity. Among the groups, 19 showed low similarity (less than 50%) to any known precursor peptide of circular bacteriocins. One novel circular bacteriocin in group 11, cerecyclin, showed the highest identity (34%) to the known circular bacteriocin enterocin NKR-5-3B and was selected for verification. Cerecyclin showed antimicrobial activity against several Gram-positive bacteria, inhibited the outgrowth of Bacillus cereus spores, and did not exhibit hemolysis activity. Moreover, it showed 4-fold- to 8-fold-higher antimicrobial activity against B. cereus and Listeria monocytogenes than nisin A. Cerecyclin also had increased stability compared to nisin A under neutral or alkaline conditions. This work not only identified a promising food biopreservative but also provided a rich source for novel circular bacteriocins.

**IMPORTANCE** Circular bacteriocins are promising biopreservatives, and it is important to identify more novel circular bacteriocins to enhance the current arsenal of antimicrobials. In this study, we used an *in silico* approach to identify a large number of novel circular bacteriocins and classified these bacteriocins into 28 groups rather than the 2 groups that were described in previous studies. Nineteen groups were novel and had low similarity (less than 50%) to any known precursor peptides of circular bacteriocins; this finding greatly expands the awareness of the novelty and diversity of circular bacteriocins. A novel circular bacteriocin which we named cerecyclin was identified in the B. cereus group; this circular bacteriocin had great antimicrobial activity against some foodborne pathogens and showed extreme stability. This study not only identified a promising food biopreservative but also provided a rich source for the identification of novel circular bacteriocins and the development of new biopreservatives.

## INTRODUCTION

Food preservatives are usually used to inhibit or prevent food spoilage caused by spoilage and pathogenic microorganisms and, consequently, improve food safety and prolong the shelf life of food (1). The growing consumer demand for foods that are safe, minimally processed, and free from chemical additives have posed great challenges to the food industry (2). The preservation of foods by natural and biological methods, such as biopreservation, is a relatively approved method to resolve current food-related issues (3). Biopreservatives include natural microflora and/or their antibacterial products that can extend storage life and enhance the safety of foods. The ribosomally synthesized antimicrobial peptides produced by microorganisms, namely, bacteriocins, especially those produced by lactic acid bacteria (LAB), have attracted great attention as food biopreservatives (4, 5). One LAB bacteriocin, nisin, which contains intramolecular covalent linkages (lanthionine rings) and belongs to a subgroup of bacteriocins called lantibiotics, was the first bacteriocin to gain widespread commercial application since 1969. Nisin is currently permitted as a food preservative in more than 50 countries (6, 7).

Although nisin has been used as a biopreservative for many years, its limited stability at neutral and alkaline pH values restricts its application (8–10). This circumstance stimulated the search for new bacteriocins which could either replace or be used in combination with nisin to extend the shelf life of food and improve food safety. In recent years, an increasing number of studies have illustrated that circular bacteriocins have great potential for use as biopreservatives (11). Circular bacteriocins are part of a growing family of bacteriocins and have N-to-C-terminal covalent linkages, allowing the formation of structurally conserved circular peptide backbones (12). All circular bacteriocins are produced as precursors with N-terminal extensions ranging between 2 and 48 amino acids, and the mature circular peptides range from 58 to 70 amino acid residues (11, 13). Most circular bacteriocins exhibit broad-spectrum antimicrobial activity against various Gram-positive bacteria, including some foodborne pathogens, such as *Bacillus* spp., *Clostridium* spp., and *Listeria* spp. Moreover, as a result of their unique circular structures, circular bacteriocins exhibit superior stability to temperature variation and pH stress and increased resistance to digestion by numerous proteases compared to many linear bacteriocins (13).

It is worth noting that only a few circular bacteriocins have been characterized so far, and systematic studies of mining novel circular bacteriocins are scarce. *In silico* approaches have been widely used to mine and identify bacteriocins because of their ability to reduce time and cost compared to culture-based screening strategies (14–17). In this study, we used an *in silico* approach to mine novel circular bacteriocins in sequenced microbial genomes. A total of 6,928 potential precursor peptides were identified and reclassified into 28 groups based on their amino acid similarity. A novel circular bacteriocin, cerecyclin (group 11), was selected to validate the antimicrobial activity of the *in silico*-predicted circular bacteriocins. The potent antimicrobial activity and stability of cerecyclin highlight its potential for use as a new biopreservative.

## RESULTS

### Identification and classification of putative circular bacteriocins and their biosynthetic gene clusters in bacteria.

Using all 15 reported precursor peptides of circular bacteriocins as driver sequences, 6,928 candidate precursor peptides were identified in 86 species of bacteria with bacterial genome sequences available in GenBank (see Text S1 in the supplemental material). Approximately 90% of the putative precursor peptides showed less than 50% sequence similarity to the reported precursor peptides of circular bacteriocins. Moreover, 71 out of 86 species had not previously been associated with circular bacteriocin production, implying that circular bacteriocins are much more widespread than previously thought. The precursor peptides were predicted from bacteria of the phylum *Firmicutes* (6,925), and a few were predicted from bacteria in the phyla *Actinobacteria* (2) and *Thermotogae* (1). The precursor peptides were mainly present in the genera *Staphylococcus* (3,034), *Streptococcus* (2,927), *Bacillus* (688), and *Enterococcus* (237). In addition, there was a great difference in the distribution of the precursor peptides. One conserved precursor peptide (more than 99% identity) was identified in 3,024 strains of Staphylococcus aureus, and another conserved precursor peptide (more than 98% identity) was found in 2,606 strains of Streptococcus pneumoniae (Text S1). In contrast, some precursor peptides existed in only one strain; for example, the precursor peptides of garvicin ML (GarA), carnocyclin A (CclA), and aureocyclicin 4185 (AclA) (18–20).

10.1128/mSystems.00047-20.1 TEXT S1 The (putative) precursor peptides of circular bacteriocins identified in this study. Download Text S1, DOC file, 2.8 MB. Copyright © 2020 Xin et al. 2020 Xin et al. This content is distributed under the terms of the Creative Commons Attribution 4.0 International license.

**FIG 1:**
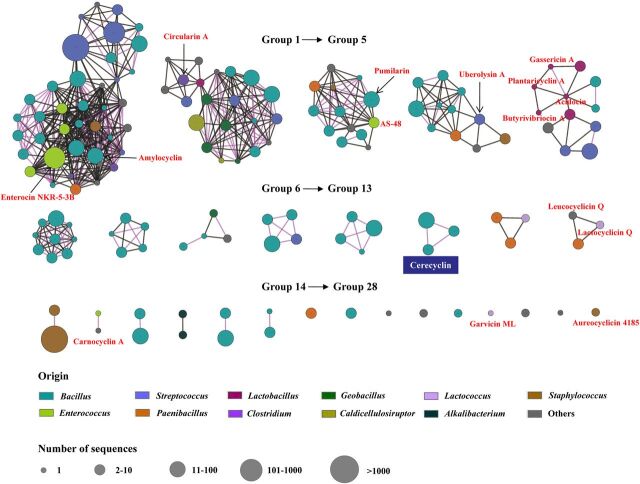
Amino acid sequence similarity networks of the identified precursor peptides of circular bacteriocins in publicly available microbial genomes. All the identified precursor peptides were classified into 28 groups based on amino acid sequence similarities. All 15 reported circular bacteriocins are shown in red font. The precursor peptides in one node originate from the same species, and the amino acid sequence similarity between each peptide is more than 85%. The size of the node represents the number of sequences. The similarity between any two nodes in the same group is more than 50%. The color of the lines between any two nodes indicates the similarities between the two nodes. A black line indicates between 50% and 85% similarity. A lavender line indicates more than 85% similarity.

To reveal the diversity of circular bacteriocins, we classified the identified circular bacteriocins based on the similarity (more than 50%) of their precursor peptides. All (putative) circular bacteriocins were classified into 28 groups (Fig. 1). Fifteen known circular bacteriocins existed in 9 of the 28 groups. Four circular bacteriocins, acidocin, gassericin A, butyrivibriocin A, and plantaricyclin A, which were classified into subgroup II circular bacteriocins in previous studies, were classified into group 5, and the other 11 circular bacteriocins, which were classified into subgroup I in previous studies, were classified into 8 different groups (Fig. 1). The putative precursor peptides that existed in the other 19 groups were novel and had low similarity (less than 50%) to any known precursor peptides of circular bacteriocins.

**FIG 2:**
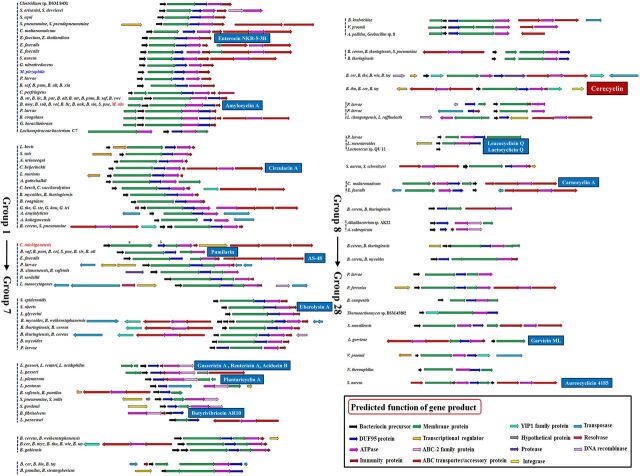
Biosynthetic gene clusters of (putative) circular bacteriocins. The previously reported circular bacteriocins are shown in white font on a blue background, and the circular bacteriocin identified in this study (cerecyclin) is shown in white font on a red background. Species of the phyla *Firmicutes*, *Actinobacteria*, and *Thermotogae* are shown in black, blue, and red fonts, respectively.

The biosynthetic gene clusters of the (putative) circular bacteriocins are shown in Fig. 2. They all contained at least three putative genes coding for precursor peptides, DUF95 family proteins, and ATP-binding proteins as well as the gene clusters of all reported circular bacteriocins. Most gene clusters contained one precursor peptide gene, and a few contained two or three precursor peptide genes. In addition, most gene clusters in the same group (according to the above classification of the [putative] precursor peptides) had the same or similar gene organization, implying that they are closely related in evolution (Fig. 1 and Fig. 2). It is a general phenomenon that different species of bacteria had highly similar circular bacteriocin gene clusters (the same gene organization and at least 85% identity among coding genes) (Fig. 2). For example, the amylocyclin A gene cluster, or the gene cluster with high sequence similarity to it, was widely distributed in the species B. amyloliquefaciens, B. subtilis, S. pneumoniae, Mycobacterium abscessus, and so on.

### Characterization of a putative circular bacteriocin gene cluster in the Bacillus cereus group.

**FIG 3:**
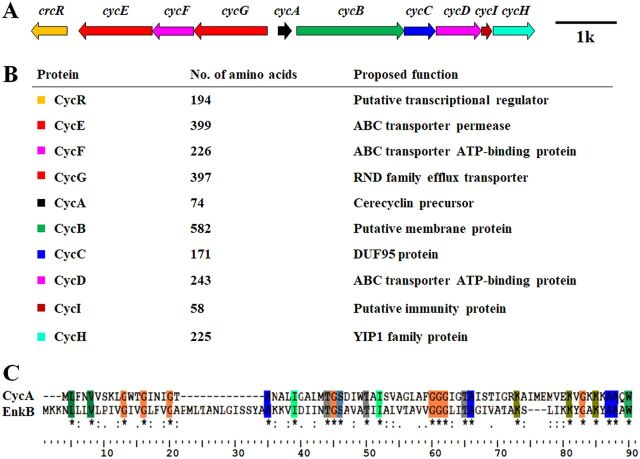
Cerecyclin gene cluster. (A) Gene cluster for the biosynthesis of cerecyclin (8,196 bp). (B) Characteristics of predicted proteins encoded by putative genes in the cerecyclin gene cluster. (C) Sequence alignment of the precursor peptide of cerecyclin (CycA) with the precursor peptide of enterocin NKR-5-3B (EnkB).

The above analysis showed that the sequenced bacterial genomes contain a large number of novel circular bacteriocins. To verify these *in silico* analyses, we focused on the putative circular bacteriocins in group 11 (Fig. 1 and Fig. 2). There were 22 putative precursor peptides in group 11, and they were highly similar (more than 85%). Their biosynthetic gene clusters were widespread in the B. cereus group (3 strains of B. cereus, 5 strains of B. toyonensis, and 14 strains of B. thuringiensis) (Fig. 2 and Text S1). We selected this group because the putative circular bacteriocins in this group exhibit weak similarity to other known circular bacteriocin precursor peptides. Moreover, B. cereus DDD103, which contains a putative circular bacteriocin gene cluster in group 11 (termed the *cyc* gene cluster), was isolated and preserved by our group. The *cyc* gene cluster consisted of 10 genes, which encoded a precursor peptide (*cycA*), a putative membrane protein (*cycB*), a putative membrane protein with a DUF95 conserved domain (*cycC*), a protein with an ATP-binding conserved domain (*cycD*), a 58-amino-acid protein with a hypothetical function as an immunity protein (*cycI*), a YIP1 family protein, an ABC transporter that could be involved in transport and/or immunity (*cycEFG*), and a regulatory protein (*cycR*) (Fig. 3A and B). The closest reported precursor peptide of circular bacteriocins to the precursor peptide CycA is EnkB (the precursor peptide of enterocin NKR-5-3B; coverage, 83%; identity, 34%) (Fig. 3C). The synthesized product of the *cyc* gene cluster, named cerecyclin, was then identified in B. cereus DDD103.

### Purification and characterization of the circular bacteriocin cerecyclin.

**FIG 4:**
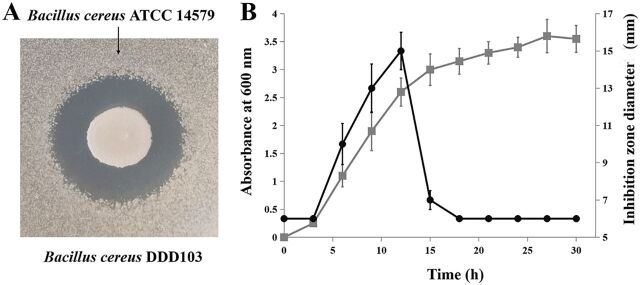
Antimicrobials produced by B. cereus DDD103. (A) Bioactivity assay with B. cereus DDD103 against B. cereus ATCC 14579 on solid agar surfaces. (B) Kinetics of antimicrobial substances produced during the growth of B. cereus DDD103 in liquid culture. The optical density of the DDD103 culture was measured at 600 nm (gray squares). The antimicrobial activity of the cell-free supernatant of B. cereus DDD103 against B. cereus ATCC 14579 was expressed as the inhibition zone diameter (black circles). The error bars indicate the standard deviations (SD) from three different experiments.

**FIG 5:**
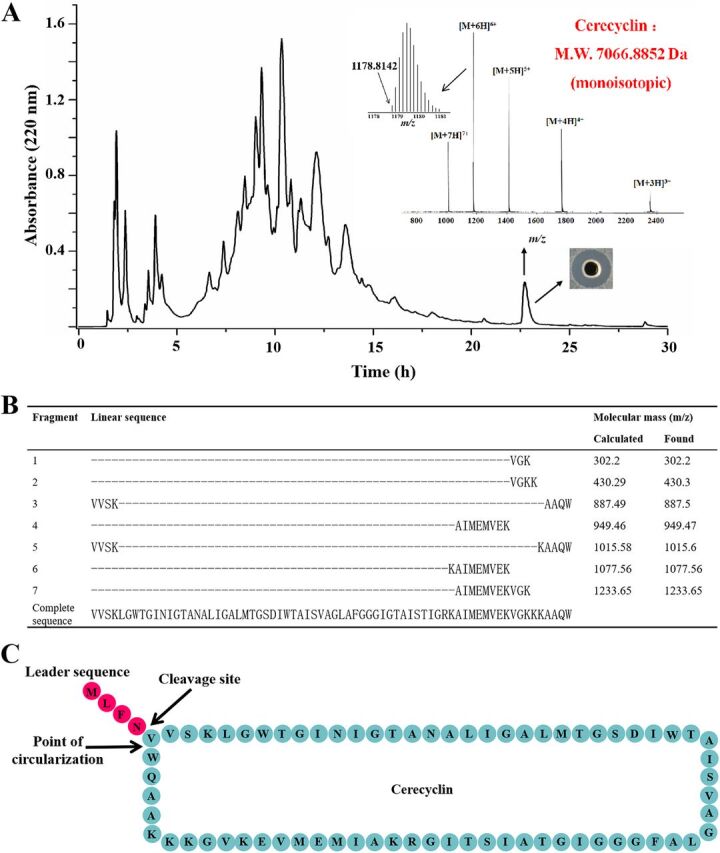
Identification of the circular bacteriocin cerecyclin. (A) Liquid chromatography-mass spectrometry (LC-MS) analysis of cerecyclin. M.W., molecular weight. (B) LC-MS analysis of fragments of cerecyclin after trypsin digestion. (C) Putative maturation process of cerecyclin. The peptide bond between Asn^4^ and Val^5^ is cleaved, and a new amide bond is formed between Trp^70^ and Val^5^.

The antimicrobials produced by B. cereus DDD103 in solid and liquid culture were measured. As shown in Fig. 4A, DDD103 demonstrably prevented the growth of B. cereus ATCC 14579 on solid medium. The kinetics of the antimicrobial production assay showed that B. cereus DDD103 produced antimicrobials during the exponential phase (Fig. 4B). The antimicrobials in the supernatant of an overnight culture (optical density at 600 nm [OD_600_] ≈ 2.5) were purified by a two-step procedure comprising adsorption on Amberlite XAD-7HP resin and reverse-phase high-performance liquid chromatography (HPLC). One fraction corresponding to the peak with a retention time of 22.8 min was active against B. cereus ATCC 14579 (Fig. 5A). Liquid chromatography-mass spectrometry (LC-MS) analysis revealed that the molecular mass of this fraction was 7066.8852 Da (monoisotopic signal). The calculated molecular mass of the predicted mature peptide of CycA (V5-W75) was 7066.84 Da (=7084.84 − 18 Da [a water molecule that arises from an N to C cyclization]), which coincided with the above fraction.

To confirm the above assumed circularity and the site of cyclization in cerecyclin, tryptic digestion and mass spectrometric analysis were performed. Seven fragments were obtained, and the N- and C-terminal amino acid residues involved in the cyclization of the peptide were observed in fragments 3 and 5 (Fig. 5B). The other five obtained fragments were also consistent with the fragments of cerecyclin. These results confirmed the circular nature of cerecyclin through amide bond formation between the two terminal residues (V5 and W75) and the cleavage site of the precursor CycA (N4-V5) (Fig. 5C).

### Cerecyclin is a nonhemolytic bacteriocin.

The antimicrobial spectrum of cerecyclin was tested against several Gram-positive and Gram-negative bacteria by the well diffusion method (see Table S1 in the supplemental material). Cerecyclin showed antimicrobial activity toward all 5 tested strains in the B. cereus group, as well as strains of B. amyloliquefaciens, B. firmus, B. subtilis, B. pumilus, Enterococcus faecalis, and Listeria monocytogenes. No antimicrobial activity was detected against S. aureus or any of the tested Gram-negative bacteria. We also compared the antimicrobial activities of cerecyclin and nisin A against five strains of the foodborne pathogens B. cereus and Listeria monocytogenes based on the MIC and 50% inhibitory concentration (IC_50_) values (Table 1). Cerecyclin exhibited antimicrobial activity with MICs ranging from 0.29 to 0.78 μM and IC_50_ ranging from 0.22 to 0.62 μM. Nisin A showed relatively weak antimicrobial activity with MIC values between 1.17 and 6.25 μM and IC_50_ ranging from 0.92 to 5.31 μM. In parallel experiments, cerecyclin demonstrated 4-fold- to 8-fold-higher antimicrobial activity against any one of five indicator strains than nisin A by comparing the MIC or IC_50_ value.

**TABLE 1:** Relative antimicrobial activities of cerecyclin and nisin A

Strain	MIC (μM)^*a*^		IC_50_ (μM)^*a*^	
	Cerecyclin	Nisin A	Cerecyclin	Nisin A
Bacillus cereus strains				
ATCC 14579	0.39 ± 0.00	1.56 ± 0.00	0.31 ± 0.02	1.28 ± 0.05
ATCC 49064	0.29 ± 0.14	1.17 ± 0.55	0.21 ± 0.01	0.92 ± 0.03
Listeria monocytogenes strains				
LM201	0.39 ± 0.00	3.13 ± 0.00	0.29 ± 0.05	2.35 ± 0.06
CMCC54002	0.39 ± 0.00	3.13 ± 0.00	0.31 ± 0.02	2.52 ± 0.05
ATCC 19115	0.78 ± 0.00	6.25 ± 0.00	0.62 ± 0.03	5.31 ± 0.08

a Average value ± standard deviation.

10.1128/mSystems.00047-20.2 TABLE S1 Antimicrobial activity of supernatant (12 h) of B. cereus DDD103 and MIC values of cerecyclin for indicator strains. Footnote a, NB, nutrient broth; LB, Luria-Bertani; TSB, tryptone soya broth. Footnote b, Antimicrobial activity was determined by measuring the diameter of the zone. Symbols: -, zones of size 6 mm; +, zones of size > 6 mm and ≤10 mm; ++, zones of size > 10 and ≤ 15 mm; +++, zones of size > 15 mm. Footnote c, -, no zone of inhibition observed when the concentration of cerecyclin was greater than 100 μM. Download Table S1, DOC file, 0.04 MB. Copyright © 2020 Xin et al. 2020 Xin et al. This content is distributed under the terms of the Creative Commons Attribution 4.0 International license.

**FIG 6:**
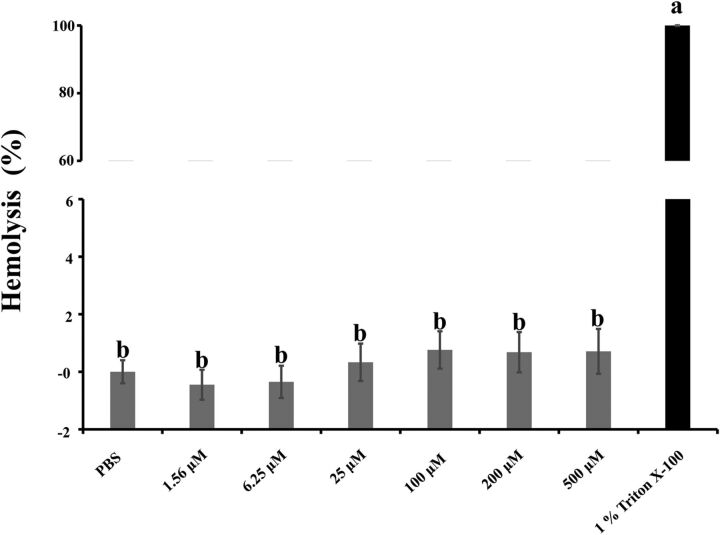
Hemolysis assay of cerecyclin. All experiments were performed in triplicate, and the error bars show the standard deviations (SD). Significantly different values are denoted by different lowercase letters (*P* < 0.05).

To be a good food preservative, cerecyclin should not be toxic to animal eukaryotic cells. To examine the cytotoxicity of cerecyclin, we performed a hemolysis assay using various concentrations (1.56 to 500 μM). As shown in Fig. 6, when the concentration of cerecyclin was less than or equal to 6.25 μM, no hemolysis of red blood cells (RBCs) was observed, and the results were not significantly different from the negative control, phosphate-buffered saline (PBS) buffer (*P* > 0.3). When the concentration of cerecyclin was between 25 μM and 500 μM, the hemolytic activity of cerecyclin increased slightly in comparison with the negative control but was significantly different from the positive control, 1% Triton X-100 (*P* < 0.05) (Fig. 6). Therefore, cerecyclin was determined to be a nonhemolytic bacteriocin, as it did not cause hemolysis even at concentrations much higher than the MIC values against various bacteria.

### Stability of cerecyclin.

**FIG 7:**
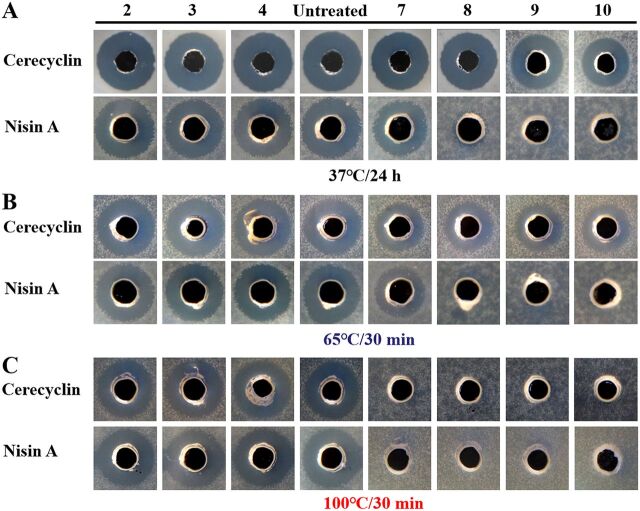
Agar well diffusion assay to determine the relative stabilities of cerecyclin and nisin A. Solutions of cerecyclin and nisin were adjusted to pH values between 2.0 and 10.0 (shown above the wells), and the preparations were kept at 37°C for 24 h or heated at 65°C and 100°C for 30 min. The residual bacteriocin activities were determined by employing B. cereus ATCC 14579 as an indicator.

The heat and acid-base stabilities of cerecyclin and nisin A were compared in this research. As shown in Fig. 7, cerecyclin and nisin A were extremely stable under acidic conditions (pH 2 to 4). However, cerecyclin was more stable than nisin A under neutral (pH 7) and alkaline (pH 8 to 10) conditions at 37°C and at 65°C. At 100°C, both cerecyclin and nisin A lost activity when incubated under either neutral (pH 7) or alkaline (pH 8 to 10) conditions.

### Inhibition of B. cereus spore outgrowth.

**FIG 8:**
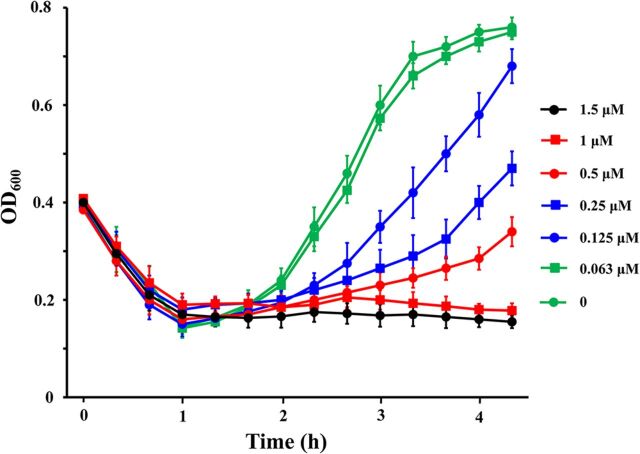
Inhibition of Bacillus cereus spore outgrowth by cerecyclin. The germination process of B. cereus ATCC 14579 spores with different concentrations of cerecyclin was monitored by measuring the OD_600_ every 20 min.

The ability of cerecyclin to inhibit B. cereus spore outgrowth was measured. Spores were germinated in Luria-Bertani (LB) medium with different concentrations of cerecyclin. As shown in Fig. 8, cerecyclin inhibited spore outgrowth at concentrations greater than 0.125 μM. The outgrowth of B. cereus spores was completely inhibited when the culture contained at least 1 μM cerecyclin.

## DISCUSSION

With the increase in concern regarding food quality and safety, some new types of preservatives, such as bacteriocins, are more accepted by consumers compared with traditional chemical preservatives (21, 22). The application of bacteriocins in food preservation provides several benefits for food industries and consumers, including the following: (i) extending the shelf life of food products, (ii) reducing the economic losses due to food spoilage, (iii) avoiding the transmission of foodborne pathogens through the food chain, and (iv) reducing the addition of chemical preservatives and the intensity of heat and other physical treatments (5). However, only a few bacteriocins are commercially available; Nisin is currently the most widely used food biopreservative among the bacteriocins; however, its applications are restricted due to its low stability at neutral and alkaline pH values (8–10). Previous studies have shown that circular bacteriocins have great potential to be developed as new preservatives based on their potent antimicrobial activity against some foodborne pathogens and their stable physical-chemical properties (13). In this research, we predicted a large number of novel circular bacteriocins and their gene clusters and identified a novel circular bacteriocin, cerecyclin, which had potent antimicrobial activity against some foodborne pathogens and extreme stability. This study not only improves understanding of the classification, distribution, and diversity of circular bacteriocin gene clusters in publicly available genomes but also provides a rich source of novel circular bacteriocins.

The reported circular bacteriocins were generally divided into two groups in previous studies based on physical-chemical characteristics and the level of sequence identity (13). Subgroup I circular bacteriocins include bacteriocins with a high level of positively charged amino acid residues, high isoelectric points, and low sequence similarity to each other (23, 24). Subgroup II circular bacteriocins include four members (gassericin A, butyrivibriocin AR10, acidocin B, and plantaricyclin A) with a small number of positively charged amino acid residues, low isoelectric points, and high sequence similarity (25, 26). In this research, all the (putative) circular bacteriocins were classified into 28 groups based on the similarity of the precursor peptides. Four circular bacteriocins in the previous subgroup II were reclassified into group 5, and the other 11 circular bacteriocins in the previous subgroup I were reclassified into 8 different groups. The remaining 19 groups were novel and had low similarity (less than 50%) to any known precursor peptides of circular bacteriocins. Thus, this analysis greatly expands the understanding of the diversity and novelty of circular bacteriocins.

It is noteworthy that the precursor peptides in 19 out of 28 groups were present in at least two different species, and most of their gene clusters had similar organization and high similarity (Fig. 1 and Fig. 2). For example, the gene clusters of AS-48, pumilarin, and other putative circular bacteriocins in group 3 all contain five conserved genes (e.g., *as-48A*, *as-48B*, *as-48C*, *as-48C1*, and *as-48D*). The As-48 gene cluster was found in strains of E. faecalis. The pumilarin gene cluster was found in strains of *B. pumilus*, B. safensis, B. altitudinis, B. stratosphericus, and S. pneumoniae. The sequence identity of the amino acid sequences of As-48A and PumA reached 70%. Moreover, some genes located adjacent to the above conserved genetic determinants, such as genes coding for transposase and DNA recombinase, are related to horizontal gene transfer (Fig. 2). This suggests that the origin of the circular bacteriocin gene clusters in the same group was the same and that horizontal transfer of the clusters occurred inside and outside the species during evolution.

All the precursor peptides of the reported circular bacteriocins have leader sequences ranging from 2 to 48 amino acid residues in length, and cleavage of the leader sequence has been assumed to be the first step in biosynthesis of the circular bacteriocins (13). However, no related peptidase/protease responsible for the cleavage of the leader peptide has been characterized in the reported circular bacteriocin gene clusters. Similarly, almost all circular bacteriocin gene clusters predicted in this study lack the genes encoding peptidases/proteases. The exception was the putative circular bacteriocin gene cluster in group 10, which had a gene encoding a putative protease (350 amino acids) with an S2P-M50 conserved domain. We presume that the leader peptides were almost all cleaved by a host-encoded peptidase that existed elsewhere, outside the gene cluster, although these enzymes have not been identified.

To date, the antimicrobial activity spectra of the reported circular bacteriocins are different. Lactocyclicin Q, leucocyclicin Q, enterocin AS-48, and pumilarin showed broad-spectrum antimicrobial activity. They not only inhibit a variety of Gram-positive bacteria but also display antimicrobial activity against Escherichia coli under certain conditions (27, 28). The circular bacteriocin identified in this study, cerecyclin, is also a broad-spectrum bacteriocin and exhibits activity against various species of *Bacillus* (B. cereus, B. firmus, B. thuringiensis, B. amyloliquefaciens, and B. pumilus), L. monocytogenes and E. faecalis, and no antimicrobial activity of cerecyclin against E. coli was detected even at high concentrations (see Table S1 in the supplemental material). The latest reported circular bacteriocin, plantaricyclin A, shows a narrow spectrum of activity against Alicyclobacillus acidoterrestris and Lactococcus lactis (29). *In silico* analysis in this study reveals that the publicly available genomes still contain a large number of novel circular bacteriocins with high amino acid sequence diversity. We can extensively mine new circular bacteriocins with different antimicrobial spectra and activities and utilize them as preservatives or alternative antimicrobial agents to combat multiple or specific pathogens.

Bacillus cereus is an important foodborne pathogen that causes an emetic or a diarrheal type of food-associated illness (30, 31). The emetic syndrome is caused by cereulide, a small ring-formed dodecadepsipeptide produced by cells growing in food, while the diarrheal syndrome is caused by complex enterotoxins (hemolysin BL, nonhemolytic enterotoxin, and cytotoxin K) produced during vegetative growth of B. cereus (ingested as viable cells or spores) in the small intestine (32). Due to the ubiquitous presence of B. cereus in nature, this pathogen can be easily spread to a broad array of foods, including raw and cooked rice, vegetables, meat, and dairy products (30, 33, 34). Although the common preservative method, heat treatment, can kill the vegetative cells of B. cereus, spores still survive the process, and B. cereus food contamination is difficult to control. Some bacteriocins, especially lantibiotics, inhibit the outgrowth of spores of B. cereus, but there are few reports about circular bacteriocins (35–37). In this research, we found that cerecyclin at low concentrations effectively inhibited not only the vegetative cells of B. cereus but also the outgrowth of the spores of B. cereus. Moreover, cerecyclin also showed potent antimicrobial activity against L. monocytogenes, a serious foodborne pathogen that can contaminate food during processing and distribution and cause listeriosis in humans (38, 39). This highlights the potential application of cerecyclin to eliminate or reduce the risk of B. cereus- and L. monocytogenes-associated food contamination in the food industry.

## MATERIALS AND METHODS

### Genome database mining to identify putative circular bacteriocins and their biosynthetic gene clusters.

Using the precursor peptides of all the reported circular bacteriocins, including acidocin B (AciA) (40), aureocyclicin 4185 (AclA) (18), amylocyclicin (AcnA) (41), enterocin AS-48 (As-48A) (42), butyrivibriocin AR10 (BviA) (25), carnocyclin A (CclA) (19), circularin A (CirA) (43), enterocin NKR-5-3B (EnkB) (44), gassericin A (GaaA) (45), garvicin ML (GarA) (20), leucocyclicin Q (LcyQ) (27), lactocyclicin Q (LycQ) (46), plantaricyclin A (PlcA) (29), pumilarin (PumA) (28), and uberolysin (UblA) (47) as driver sequences, all sequenced microbial gene sequences were mined for homologs using PSI-BLAST searches (48). The criteria for homolog detection were greater than 60% coverage and 30% identity. In cases where a putative precursor peptide gene encoding a circular bacteriocin was identified, the adjacent genes were subjected to BLAST analysis to identify their potential functions in circular bacteriocin synthesis. Because all the reported biosynthesis gene clusters of circular bacteriocins contain at least three genes, which encode a precursor peptide, a DUF95 family protein and an ATP-binding protein, the putative circular bacteriocin gene clusters identified in this study needed to contain these three genes as well.

### Group analysis of circular bacteriocins.

All the potential precursor peptides of circular bacteriocins were performed with all-against-all BLASTP analysis. We tested the best E-value pairwise comparison for each pair of sequences with similarity to obtain the best grouping results. When the similarity between any two sequences in the same group was more than 50%, most of their gene clusters had similar gene organization. Therefore, we divided the precursor peptides whose amino acid sequence similarity was more than 50% into a group. Protein sequence groups were visualized and edited using Cytoscape (49).

### Strains and growth conditions.

Bacillus cereus DDD103, which was isolated from soil in Hubei Province in China was sequenced using paired-end sequencing technology on the Illumina Hiseq 2500 platform and assembled by PGCGAP (50). B. cereus DDD103, which contained a putative circular bacteriocin gene cluster (group 11) termed the *cyc* gene cluster in its genome, was selected for further study. B. cereus DDD103 was stored at –80°C in Luria-Bertani (LB) medium with 15% glycerol and propagated overnight on LB agar plates at 28°C before use. The indicator strains used in this study and their culture conditions were shown in Table S1 in the supplemental material. Escherichia coli BL21 and E. coli DH5a were purchased from TaKaRa (Dalian, China). Salmonella enterica serotype Paratyphi CMCC50094, Staphylococcus aureus CMCC26003, and Listeria monocytogenes CMCC54002 were purchased from China Medical Culture Collection (CMCC). Pseudomonas aeruginosa ATCC 27853, B. cereus ATCC 14579, B. cereus ATCC 49064, S. aureus ATCC 29213, L. monocytogenes ATCC 19115, Enterococcus faecalis ATCC 29212, and E. faecalis ATCC 51299 were purchased from American Type Culture Collection (ATCC). *B. amyloliquefaciens* X1, B. firmus DS-1, *B. pumilus* SCG I, and P. putida *Pri3* were isolated and preserved by our group. For the sources of B. subtilis 168, B. thuringiensis BMB171, B. thuringiensis YBT-1518, and L. monocytogenes LM201, see references 51 to 54.

### Antimicrobial activity assay.

The antimicrobial activity of B. cereus DDD103 on solid culture medium was determined by the following methods. LB agar was inoculated 1:10,000 with an overnight culture of B. cereus ATCC 14579, an important foodborne pathogen. Five microliters of an overnight culture of B. cereus DDD103 was dropped on the surface of the resulting bacterial lawn, and the plates were incubated for 24 h at 30°C. The formation of the inhibition zone around the colony was observed. Then, the production of antimicrobials by strain DDD103 in liquid culture was detected. Overnight culture of DDD103 (1 ml) was inoculated into 100 ml of LB broth and incubated at 30°C for 30 h. The OD_600_ values of the cultures were measured every 3 h. The cell-free supernatants from 3 h to 30 h were also collected and tested against B. cereus ATCC 14579 using the agar well diffusion method as previously described (55). Briefly, 20 ml of melted agar (45°C) was seeded with 10^6^ CFU of the indicator strain in a petri dish, and holes (diameter, 6 mm) were bored in the agar. Next, 50 μl of the sample was pipetted into the wells, and the plates were incubated at 4°C for 2 h. Subsequently, the plates were incubated at 30°C for 16 h. The diameter of the inhibition zones was then measured and recorded. The antimicrobial activities of 12 h (the highest level of antimicrobial substance) supernatant of B. cereus DDD103 against all the tested strains (Table S1) were also detected using the above method.

The MICs of cerecyclin and nisin A for the indicator strains were measured by different final concentrations (0.2, 0.29, 0.39, 0.59, 0.78, 1.17, 1.56, 2.34, 3.13, 4.69, 6.25, 9.38, 12.5, 18.75, 25, 50, and 100 μM) of bacteriocin preparation using the well diffusion assays described above. The MIC was defined as the lowest concentration of samples that could form a clear zone of inhibition. The 50% inhibitory concentrations (IC_50s_) of cerecyclin and nisin A were determined by the following methods. Briefly, each well of the 96-well plate contained 10 μl of diluted bacteriocin at a defined concentration and 90 μl of overnight-cultured inoculum (final concentration of approximately 1 × 10^6^ CFU/ml). The negative-control well contained 10 μl of double-distilled water (ddH_2_O) and 90 μl of inoculum (1 × 10^6^ CFU/ml). The 96-well plate was agitated to mix the content of the wells and incubated under appropriate growth conditions (Table S1), and IC_50_ calculations were performed by monitoring the OD_600_. All assays were performed in triplicate.

### Purification of cerecyclin.

B. cereus DDD103 was grown overnight at 28°C in LB broth. The activated culture was inoculated (1%) into 1 liter of LB broth at 28°C with agitation at 220 rpm for 12 h (OD_600_ ≈ 2.5). The culture was centrifuged (12,000 rpm, 10 min, 4°C), and the supernatant was applied to a column containing 100 g of Amberlite XAD-7HP resin (Sigma, St. Louis, MO, USA). The resin was sequentially washed with 1 liter of distilled water and 1 liter of 20% (vol/vol) ethanol. The active substances were eluted with 500 ml of 80% (vol/vol) ethanol (pH 2.0). The eluate was concentrated by a rotary evaporator and then lyophilized into powder. The generated powder was dissolved in 5 ml of 50% (vol/vol) acetonitrile and subjected to HPLC analysis using an Agilent HC-C18(2) column (particle size, 5 μm; 4.6 mm by 250 mm) on a Waters 1525 Breeze system. A flow rate of 1 ml/min was employed with the use of HPLC-grade water containing 0.1% trifluoroacetic acid (TFA) and acetonitrile. Fifty microliters of the sample was injected per run and separated by a linear gradient of 10% to 90% acetonitrile from 0 to 30 min; the elute was monitored at a wavelength of 220 nm. Each fraction was tested for its antimicrobial activity against B. cereus ATCC 14579 (an important foodborne pathogen) with the agar well diffusion method (55). The active fraction was collected 50 times and then lyophilized into powder. The powder was then dissolved in ddH_2_O, and the concentration of bacteriocin preparation was quantified to 500 μM and used for further experiments.

### LC-MS analysis.

Liquid chromatography-mass spectrometry (LC-MS) was performed by the Agilent 6540 Ultra High Definition (UHD) Accurate-Mass quadrupole time of flight (Q-TOF) LC-MS system to reveal the molecular weight of cerecyclin. The analytical column was a ZORBAX Eclipse Plus C_18_ column (3.5 μm; 2.1 by 150 mm; Agilent). The MS operating conditions were as follows: flow rate of drying gas, 9 liters/min; capillary voltage, 3,500 V; nebulizer pressure, 35 lb/in^2^ gauge; and temperature, 350°C. The scanning range of Q-TOF MS was *m/z* 100 to 3,000.

### Enzymatic digestion.

An aliquot containing ∼5 μg of cerecyclin was dissolved in 20 μl of 100 mM ammonium bicarbonate buffer (pH 8.5) and digested with trypsin (Sigma) overnight at 37°C at an enzyme-to-peptide molar ratio of 1:40. The obtained fragments were then analyzed by liquid chromatography-mass spectrometry (LC-MS) as described above.

### Hemolysis assay.

The hemolytic activity of cerecyclin was determined by measuring the release of hemoglobin from a suspension of defibrillated sheep blood (Guangzhou Ruite Biotechnology Co., Ltd., China) at an absorbance of 540 nm. Briefly, defibrillated sheep blood was centrifuged, and the sedimented cells were washed three times with PBS buffer (pH 7.0). Then, 10 μl of resuspended red blood cells were added to a 96-well plate containing 90 μl of cerecyclin at different final concentrations (1.56, 6.25, 25, 100, 200, and 500 μM) and incubated for 1 h at 37°C. After incubation, the samples were centrifuged (3,000 rpm for 5 min), and the supernatants were transferred to a new 96-well plate to examine erythrocyte lysis at a wavelength of 540 nm. Triton X-100 (1%) was used as a positive control, and PBS buffer was used as a negative control in this experiment. All experiments were performed in triplicate, and the results are expressed as means ± standard deviations. All statistical analyses were conducted using SPSS statistical software 20.0. One-way analysis of variance with Tukey’s test was used to determine whether any significant differences existed between multiple treatments of data at *P* < 0.05.

### Inhibition of Bacillus cereus spore outgrowth.

Germination assays were performed as described previously (56). B. cereus ATCC 14579 spores were incubated in LB broth (OD_600_ ≈ 0.4) supplemented with cerecyclin (0.063, 0.125, 0.25, 0.5, 1, or 1.5 μM) or with ddH_2_O as a control. The germination process was monitored at a wavelength of 600 nm every 20 min.

### Characterization of the stability of cerecyclin.

To test the heat and pH stability of cerecyclin, the pH of the cerecyclin solution was adjusted to values between 2.0 and 10.0 using 1 M hydrochloric acid or 1 M sodium hydroxide and kept at 37°C for 24 h or heated at 65°C and 100°C for 30 min, respectively. The residual bacteriocin activities were determined as described above by employing B. cereus ATCC 14579 as an indicator strain. All tests were performed in triplicate. The bacteriocin activity of the pH 5.0 sample without heat treatment was defined as 100%.

### Data availability.

The whole-genome shotgun sequencing results for B. cereus DDD103 have been deposited in GenBank under accession no. PVRX00000000. The nucleotide sequence of the cerecyclin gene cluster has been deposited in GenBank under accession no. MH037333.
