# Supplementary material for: In Silico Analysis Highlights the Diversity and Novelty of Circular Bacteriocins in Sequenced Microbial Genomes
Source: mSystems. 2020 Jun 2;5(3):e00047-20. doi: 10.1128/mSystems.00047-20 (PMC8534725; doi:10.1128/mSystems.00047-20)
Supplement: TEXT S1 [file msystems.00047-20-s0001.doc]

**Group 1 (3302)**

>Bacillus xiamenensis EKF37307.1

MTKATDSKFYALLSLSLLAVTSLALVIGNGSLIAANLGVSTGTAFTIVNFLDTWSSVATVITIVGMFTGVGTISAGVAASILAILKKKGKAKAAAF

>Bacillus pumilus ALM27578.1

MTKATDSRFYVLLSLSLLAVTSVALVIGNGSLIAANLGVSTGTAFTIVNFLDTWSSVATVITIVGMFTGVGTISAGVAASILAILKKKGKAKAAAF

>Bacillus pumilus ALM44121.1

MTKATDSRFYVLLSLSLLAVTSVALVIGNGSLIAANLGVSTGTAFTIVNFLDTWSSVATVITIVGMFTGVGTISAGVAASILAILKKKGKAKAAAF

>Bacillus pumilus ANY95593.1

MTKATDSRFYVLLSLSLLAVTSVALVIGNGSLIAANLGVSTGTAFTIVNFLDTWSSVATVITIVGMFTGVGTISAGVAASILAILKKKGKAKAAAF

>Bacillus pumilus ANY95593.1

MTKATDSRFYVLLSLSLLAVTSVALVIGNGSLIAANLGVSTGTAFTIVNFLDTWSSVATVITIVGMFTGVGTISAGVAASILAILKKKGKAKAAAF

>Bacillus sp. M 2-6 EIL85778.1

MTKATDSRFYVLLSLSLLAVTSVALVIGNGSLIAANLGVSTGTAFTIVNFLDTWSSVATVITIVGMFTGVGTISAGVAASILAILKKKGKAKAAAF

>Bacillus sp. TH007 KRV44573.1

MTKATDSRFYVLLSLSLLAVTSVALVIGNGSLIAANLGVSTGTAFTIVNFLDTWSSVATVITIVGMFTGVGTISAGVAASILAILKKKGKAKAAAF

>Bacillus altitudinis KDE31176.1

MTKATDSKFYALLSLSLLAVTLIALVIGNGSLIAANLGVSTGTAFTIVNFLDAWSSVATVITIVGMFTGVGTISAGVAASILAIIKKKEKSKAAAF

>Bacillus pumilus AMM90677.1

MTKATDSKFYALLSLSLLAVTLIALVIGNGSLIAANLGVSTGTAFTIVNFLDAWSSVATVITIVGMFTGVGTISAGVAASILAIIKKKEKSKAAAF

>Bacillus pumilus ABV64019.2

MTKATDSKFYALLSLSLLAVTLVALVIGNGSLIAANLGVSTATAATVVNFLDTWSSVATVITIVGVFTGVGTISSGVAATILAILKKQGKAKAAAF

>Bacillus pumilus OUZ07374.1

MTKATDSKFYALLSLSLLAVTLVALVIGNGSLIAANLGVSTATAATVVNFLDTWSSVATVITIVGVFTGVGTISSGVAATILAILKKQGKAKAAAF

>Bacillus pumilus EDW21421.1

MTKATDSKFYALLSLSLLAVTLVALVIGNGSLIAANLGVSTATAATVVNFLDTWSSVATVITIVGVFTGVGTISSGVAAAILAILKKEGKAKAAAF

>Bacillus pumilus KIL19916.1

MTKATDSKFYALLSLSLLAVTLVALVIGNGSLIAANLGVSTATAATVVNFLDTWSSVATVITIVGVFTGVGTISSGVAAAILAILKKEGKAKAAAF

>Bacillus pumilus KRU17715.1

MTKATDSKFYALLSLSLLAVTLVALVIGNGSLIAANLGVSTATAATVVNFLDTWSSVATVITIVGVFTGVGTISSGVAAAILAILKKEGKAKAAAF

>Bacillus pumilus AMM99015.1

MTKATDSKFYALLSLSLLAVTLVALVIGNGSLIAANLGVSTATAATVVNFLDTWSSVATVITIVGVFTGVGTISSGVAAAILAILKKEGKAKAAAF

>Bacillus pumilus OBS85907.1

MTKATDSKFYALLSLSLLAVTLVALVIGNGSLIAANLGVSTATAATVVNFLDTWSSVATVITIVGVFTGVGTISSGVAAAILAILKKEGKAKAAAF

>Bacillus pumilus SNV15627.1

MTKATDSKFYALLSLSLLAVTLVALVIGNGSLIAANLGVSTATAATVVNFLDTWSSVATVITIVGVFTGVGTISSGVAAAILAILKKEGKAKAAAF

>Bacillus safensis APT51232.1

MTKATDSKFYALLSLLLLAVTLGALVIGNGSLIAANLGVSSGTAFAMVNFLDAWSSVATVITVVGMFTGVGTISAGVAATILAILKKKGKAKAAAF

>Bacillus safensis APT52496.1

MTKATDSKFYALLSLLLLAVTLGALVIGNGSLIAANLGVSSGTAFAMVNFLDAWSSVATVITVVGMFTGVGTISAGVAATILAILKKKGKAKAAAF

>Bacillus amyloliquefaciens OBR31826.1/Amylocyclin A

MFGAALAAATFVYALLLTGTELNVAAAHAFSANAELASTLGISAAAAKKAIDIIDAASTIASIISLIGIVTGAGAISYAIVATAKTMIKKYGKKYAAAW

>Bacillus amyloliquefaciens ARW40404.1

MFGAALAAATFVYALLLTGTELNVAAAHAFSANAELASTLGISAAAAKKAIDIIDAASTIASIISLIGIVTGAGAISYAIVATAKTMIKKYGKKYAAAW

>Bacillus amyloliquefaciens OXL21255.1

MVVKSNKKSFILFGAALAAATFVYALLLTGTELNVAAAHAFSANAELASTLGISAAAAKKAIDIIDAASTIASIISLIGIVTGAGAISYAIVATAKTMIKKYGKKYAAAW

>Bacillus amyloliquefaciens KYC99399.1

MMNLVKSNKKSFILFGAALAAATFVYALLLTGTELNVAAAHAFSANAELASTLGISAAAAKKAIDIIDAASTIASIISLIGIVTGAGAISYAIVATAKTMIKKYGKKYAAAW

>Bacillus amyloliquefaciens AEB25352.1

MNLVKSNKKSFILFGAALAAATLVYALLLTGTELNVAAAHAFSANAELASTLGISTAAAKKAIDIIDAASTIASIISLIGIVTGAGAISYAIVATAKTMIKKYGKKYAAAW

>Bacillus sp. 916 EJD68000.1

MNLVKSNKKSFILFGAALAAATLVYALLLTGTELNVAAAHAFSANAELASTLGISTAAAKKAIDIIDAASTIASIISLIGIVTGAGAISYAIVATAKTMIKKYGKKYAAAW

>Bacillus velezensis EKE49348.1

MNLVKSNKKSFILFGAALAAATLVYALLLTGTELNVAAAHAFSANAELASTLGISTAAAKKAIDIIDAASTIASIISLIGIVTGAGAISYAIVATAKTMIKKYGKKYAAAW

>Bacillus velezensis AFZ92002.1

MNLVKSNKKSFILFGAALAAATLVYALLLTGTELNVAAAHAFSANAELASTLGISTAAAKKAIDIIDAASTIASIISLIGIVTGAGAISYAIVATAKTMIKKYGKKYAAAW

>Bacillus amyloliquefaciens AGF26402.1

MNLVKSNKKSFILFGAALAAATLVYALLLTGTELNVAAAHAFSANAELASTLGISTAAAKKAIDIIDAASTIASIISLIGIVTGAGAISYAIVATAKTMIKKYGKKYAAAW

>Bacillus amyloliquefaciens ERH49637.1

MNLVKSNKKSFILFGAALAAATLVYALLLTGTELNVAAAHAFSANAELASTLGISTAAAKKAIDIIDAASTIASIISLIGIVTGAGAISYAIVATAKTMIKKYGKKYAAAW

>Bacillus amyloliquefaciens ERK84944.1

MNLVKSNKKSFILFGAALAAATLVYALLLTGTELNVAAAHAFSANAELASTLGISTAAAKKAIDIIDAASTIASIISLIGIVTGAGAISYAIVATAKTMIKKYGKKYAAAW

>Bacillus amyloliquefaciens AHC43492.1

MNLVKSNKKSFILFGAALAAATLVYALLLTGTELNVAAAHAFSANAELASTLGISTAAAKKAIDIIDAASTIASIISLIGIVTGAGAISYAIVATAKTMIKKYGKKYAAAW

>Bacillus velezensis AHK50417.1

MNLVKSNKKSFILFGAALAAATLVYALLLTGTELNVAAAHAFSANAELASTLGISTAAAKKAIDIIDAASTIASIISLIGIVTGAGAISYAIVATAKTMIKKYGKKYAAAW

>Bacillus amyloliquefaciens EYB35867.1

MNLVKSNKKSFILFGAALAAATLVYALLLTGTELNVAAAHAFSANAELASTLGISTAAAKKAIDIIDAASTIASIISLIGIVTGAGAISYAIVATAKTMIKKYGKKYAAAW

>Bacillus amyloliquefaciens KDN92969.1

MNLVKSNKKSFILFGAALAAATLVYALLLTGTELNVAAAHAFSANAELASTLGISTAAAKKAIDIIDAASTIASIISLIGIVTGAGAISYAIVATAKTMIKKYGKKYAAAW

>Bacillus subtilis KFI15261.1

MNLVKSNKKSFILFGAALAAATLVYALLLTGTELNVAAAHAFSANAELASTLGISTAAAKKAIDIIDAASTIASIISLIGIVTGAGAISYAIVATAKTMIKKYGKKYAAAW

>Bacillus amyloliquefaciens KFX37902.1

MNLVKSNKKSFILFGAALAAATLVYALLLTGTELNVAAAHAFSANAELASTLGISTAAAKKAIDIIDAASTIASIISLIGIVTGAGAISYAIVATAKTMIKKYGKKYAAAW

>Bacillus subtilis AIU75912.1

MNLVKSNKKSFILFGAALAAATLVYALLLTGTELNVAAAHAFSANAELASTLGISTAAAKKAIDIIDAASTIASIISLIGIVTGAGAISYAIVATAKTMIKKYGKKYAAAW

>Bacillus subtilis AIW31116.1

MNLVKSNKKSFILFGAALAAATLVYALLLTGTELNVAAAHAFSANAELASTLGISTAAAKKAIDIIDAASTIASIISLIGIVTGAGAISYAIVATAKTMIKKYGKKYAAAW

>Bacillus subtilis AIW35014.1

MNLVKSNKKSFILFGAALAAATLVYALLLTGTELNVAAAHAFSANAELASTLGISTAAAKKAIDIIDAASTIASIISLIGIVTGAGAISYAIVATAKTMIKKYGKKYAAAW

>Bacillus subtilis AIW38667.1

MNLVKSNKKSFILFGAALAAATLVYALLLTGTELNVAAAHAFSANAELASTLGISTAAAKKAIDIIDAASTIASIISLIGIVTGAGAISYAIVATAKTMIKKYGKKYAAAW

>Bacillus sp. Pc3 AJC26239.1

MNLVKSNKKSFILFGAALAAATLVYALLLTGTELNVAAAHAFSANAELASTLGISTAAAKKAIDIIDAASTIASIISLIGIVTGAGAISYAIVATAKTMIKKYGKKYAAAW

>Bacillus sp. BH072 AJE79886.1

MNLVKSNKKSFILFGAALAAATLVYALLLTGTELNVAAAHAFSANAELASTLGISTAAAKKAIDIIDAASTIASIISLIGIVTGAGAISYAIVATAKTMIKKYGKKYAAAW

>Bacillus amyloliquefaciens AJH25227.1

MNLVKSNKKSFILFGAALAAATLVYALLLTGTELNVAAAHAFSANAELASTLGISTAAAKKAIDIIDAASTIASIISLIGIVTGAGAISYAIVATAKTMIKKYGKKYAAAW

>Bacillus velezensis KIR07267.1

MNLVKSNKKSFILFGAALAAATLVYALLLTGTELNVAAAHAFSANAELASTLGISTAAAKKAIDIIDAASTIASIISLIGIVTGAGAISYAIVATAKTMIKKYGKKYAAAW

>Bacillus amyloliquefaciens KJD58522.1

MNLVKSNKKSFILFGAALAAATLVYALLLTGTELNVAAAHAFSANAELASTLGISTAAAKKAIDIIDAASTIASIISLIGIVTGAGAISYAIVATAKTMIKKYGKKYAAAW

>Bacillus amyloliquefaciens AKD23367.1

MNLVKSNKKSFILFGAALAAATLVYALLLTGTELNVAAAHAFSANAELASTLGISTAAAKKAIDIIDAASTIASIISLIGIVTGAGAISYAIVATAKTMIKKYGKKYAAAW

>Bacillus amyloliquefaciens KMO05949.1

MNLVKSNKKSFILFGAALAAATLVYALLLTGTELNVAAAHAFSANAELASTLGISTAAAKKAIDIIDAASTIASIISLIGIVTGAGAISYAIVATAKTMIKKYGKKYAAAW

>Bacillus velezensis KJR67929.1

MNLVKSNKKSFILFGAALAAATLVYALLLTGTELNVAAAHAFSANAELASTLGISTAAAKKAIDIIDAASTIASIISLIGIVTGAGAISYAIVATAKTMIKKYGKKYAAAW

>Bacillus velezensis AKF31937.1

MNLVKSNKKSFILFGAALAAATLVYALLLTGTELNVAAAHAFSANAELASTLGISTAAAKKAIDIIDAASTIASIISLIGIVTGAGAISYAIVATAKTMIKKYGKKYAAAW

>Bacillus velezensis AKF75467.1

MNLVKSNKKSFILFGAALAAATLVYALLLTGTELNVAAAHAFSANAELASTLGISTAAAKKAIDIIDAASTIASIISLIGIVTGAGAISYAIVATAKTMIKKYGKKYAAAW

>Bacillus sp. LK7 KMN57769.1

MNLVKSNKKSFILFGAALAAATLVYALLLTGTELNVAAAHAFSANAELASTLGISTAAAKKAIDIIDAASTIASIISLIGIVTGAGAISYAIVATAKTMIKKYGKKYAAAW

>Bacillus amyloliquefaciens KOS49520.1

MNLVKSNKKSFILFGAALAAATLVYALLLTGTELNVAAAHAFSANAELASTLGISTAAAKKAIDIIDAASTIASIISLIGIVTGAGAISYAIVATAKTMIKKYGKKYAAAW

>Bacillus amyloliquefaciens KPD34883.1

MNLVKSNKKSFILFGAALAAATLVYALLLTGTELNVAAAHAFSANAELASTLGISTAAAKKAIDIIDAASTIASIISLIGIVTGAGAISYAIVATAKTMIKKYGKKYAAAW

>Bacillus amyloliquefaciens KTF58703.1

MNLVKSNKKSFILFGAALAAATLVYALLLTGTELNVAAAHAFSANAELASTLGISTAAAKKAIDIIDAASTIASIISLIGIVTGAGAISYAIVATAKTMIKKYGKKYAAAW

>Bacillus amyloliquefaciens ALV02573.1

MNLVKSNKKSFILFGAALAAATLVYALLLTGTELNVAAAHAFSANAELASTLGISTAAAKKAIDIIDAASTIASIISLIGIVTGAGAISYAIVATAKTMIKKYGKKYAAAW

>Bacillus amyloliquefaciens AMP33166.1

MNLVKSNKKSFILFGAALAAATLVYALLLTGTELNVAAAHAFSANAELASTLGISTAAAKKAIDIIDAASTIASIISLIGIVTGAGAISYAIVATAKTMIKKYGKKYAAAW

>Bacillus amyloliquefaciens AMQ68952.1

MNLVKSNKKSFILFGAALAAATLVYALLLTGTELNVAAAHAFSANAELASTLGISTAAAKKAIDIIDAASTIASIISLIGIVTGAGAISYAIVATAKTMIKKYGKKYAAAW

>Bacillus amyloliquefaciens AMQ75379.1

MNLVKSNKKSFILFGAALAAATLVYALLLTGTELNVAAAHAFSANAELASTLGISTAAAKKAIDIIDAASTIASIISLIGIVTGAGAISYAIVATAKTMIKKYGKKYAAAW

>Bacillus amyloliquefaciens AMR51567.1

MNLVKSNKKSFILFGAALAAATLVYALLLTGTELNVAAAHAFSANAELASTLGISTAAAKKAIDIIDAASTIASIISLIGIVTGAGAISYAIVATAKTMIKKYGKKYAAAW

>Bacillus amyloliquefaciens APB83447.1

MNLVKSNKKSFILFGAALAAATLVYALLLTGTELNVAAAHAFSANAELASTLGISTAAAKKAIDIIDAASTIASIISLIGIVTGAGAISYAIVATAKTMIKKYGKKYAAAW

>Bacillus amyloliquefaciens APH48937.1

MNLVKSNKKSFILFGAALAAATLVYALLLTGTELNVAAAHAFSANAELASTLGISTAAAKKAIDIIDAASTIASIISLIGIVTGAGAISYAIVATAKTMIKKYGKKYAAAW

>Bacillus velezensis KOC22322.1

MNLVKSNKKSFILFGAALAAATLVYALLLTGTELNVAAAHAFSANAELASTLGISTAAAKKAIDIIDAASTIASIISLIGIVTGAGAISYAIVATAKTMIKKYGKKYAAAW

>Bacillus velezensis KOC23186.1

MNLVKSNKKSFILFGAALAAATLVYALLLTGTELNVAAAHAFSANAELASTLGISTAAAKKAIDIIDAASTIASIISLIGIVTGAGAISYAIVATAKTMIKKYGKKYAAAW

>Bacillus velezensis KOC81726.1

MNLVKSNKKSFILFGAALAAATLVYALLLTGTELNVAAAHAFSANAELASTLGISTAAAKKAIDIIDAASTIASIISLIGIVTGAGAISYAIVATAKTMIKKYGKKYAAAW

>Bacillus velezensis KRQ90812.1

MNLVKSNKKSFILFGAALAAATLVYALLLTGTELNVAAAHAFSANAELASTLGISTAAAKKAIDIIDAASTIASIISLIGIVTGAGAISYAIVATAKTMIKKYGKKYAAAW

>Bacillus velezensis KRT31641.1

MNLVKSNKKSFILFGAALAAATLVYALLLTGTELNVAAAHAFSANAELASTLGISTAAAKKAIDIIDAASTIASIISLIGIVTGAGAISYAIVATAKTMIKKYGKKYAAAW

>Bacillus velezensis KSV96151.1

MNLVKSNKKSFILFGAALAAATLVYALLLTGTELNVAAAHAFSANAELASTLGISTAAAKKAIDIIDAASTIASIISLIGIVTGAGAISYAIVATAKTMIKKYGKKYAAAW

>Bacillus velezensis KSW02156.1

MNLVKSNKKSFILFGAALAAATLVYALLLTGTELNVAAAHAFSANAELASTLGISTAAAKKAIDIIDAASTIASIISLIGIVTGAGAISYAIVATAKTMIKKYGKKYAAAW

>Bacillus velezensis KSW05131.1

MNLVKSNKKSFILFGAALAAATLVYALLLTGTELNVAAAHAFSANAELASTLGISTAAAKKAIDIIDAASTIASIISLIGIVTGAGAISYAIVATAKTMIKKYGKKYAAAW

>Bacillus velezensis KTF58469.1

MNLVKSNKKSFILFGAALAAATLVYALLLTGTELNVAAAHAFSANAELASTLGISTAAAKKAIDIIDAASTIASIISLIGIVTGAGAISYAIVATAKTMIKKYGKKYAAAW

>Bacillus velezensis KUP41219.1

MNLVKSNKKSFILFGAALAAATLVYALLLTGTELNVAAAHAFSANAELASTLGISTAAAKKAIDIIDAASTIASIISLIGIVTGAGAISYAIVATAKTMIKKYGKKYAAAW

>Bacillus velezensis ANB48348.1

MNLVKSNKKSFILFGAALAAATLVYALLLTGTELNVAAAHAFSANAELASTLGISTAAAKKAIDIIDAASTIASIISLIGIVTGAGAISYAIVATAKTMIKKYGKKYAAAW

>Bacillus velezensis ANB84905.1

MNLVKSNKKSFILFGAALAAATLVYALLLTGTELNVAAAHAFSANAELASTLGISTAAAKKAIDIIDAASTIASIISLIGIVTGAGAISYAIVATAKTMIKKYGKKYAAAW

>Bacillus velezensis OAL86105.1

MNLVKSNKKSFILFGAALAAATLVYALLLTGTELNVAAAHAFSANAELASTLGISTAAAKKAIDIIDAASTIASIISLIGIVTGAGAISYAIVATAKTMIKKYGKKYAAAW

>Bacillus velezensis OAL93459.1

MNLVKSNKKSFILFGAALAAATLVYALLLTGTELNVAAAHAFSANAELASTLGISTAAAKKAIDIIDAASTIASIISLIGIVTGAGAISYAIVATAKTMIKKYGKKYAAAW

>Bacillus velezensis ANS39540.1

MNLVKSNKKSFILFGAALAAATLVYALLLTGTELNVAAAHAFSANAELASTLGISTAAAKKAIDIIDAASTIASIISLIGIVTGAGAISYAIVATAKTMIKKYGKKYAAAW

>Bacillus velezensis ANU31297.1

MNLVKSNKKSFILFGAALAAATLVYALLLTGTELNVAAAHAFSANAELASTLGISTAAAKKAIDIIDAASTIASIISLIGIVTGAGAISYAIVATAKTMIKKYGKKYAAAW

>Bacillus velezensis OCJ73514.1

MNLVKSNKKSFILFGAALAAATLVYALLLTGTELNVAAAHAFSANAELASTLGISTAAAKKAIDIIDAASTIASIISLIGIVTGAGAISYAIVATAKTMIKKYGKKYAAAW

>Bacillus velezensis ODB64462.1

MNLVKSNKKSFILFGAALAAATLVYALLLTGTELNVAAAHAFSANAELASTLGISTAAAKKAIDIIDAASTIASIISLIGIVTGAGAISYAIVATAKTMIKKYGKKYAAAW

>Bacillus velezensis ODB72039.1

MNLVKSNKKSFILFGAALAAATLVYALLLTGTELNVAAAHAFSANAELASTLGISTAAAKKAIDIIDAASTIASIISLIGIVTGAGAISYAIVATAKTMIKKYGKKYAAAW

>Bacillus velezensis ODB75913.1

MNLVKSNKKSFILFGAALAAATLVYALLLTGTELNVAAAHAFSANAELASTLGISTAAAKKAIDIIDAASTIASIISLIGIVTGAGAISYAIVATAKTMIKKYGKKYAAAW

>Bacillus velezensis AOO62815.1

MNLVKSNKKSFILFGAALAAATLVYALLLTGTELNVAAAHAFSANAELASTLGISTAAAKKAIDIIDAASTIASIISLIGIVTGAGAISYAIVATAKTMIKKYGKKYAAAW

>Bacillus velezensis APA03995.1

MNLVKSNKKSFILFGAALAAATLVYALLLTGTELNVAAAHAFSANAELASTLGISTAAAKKAIDIIDAASTIASIISLIGIVTGAGAISYAIVATAKTMIKKYGKKYAAAW

>Bacillus velezensis OOI00150.1

MNLVKSNKKSFILFGAALAAATLVYALLLTGTELNVAAAHAFSANAELASTLGISTAAAKKAIDIIDAASTIASIISLIGIVTGAGAISYAIVATAKTMIKKYGKKYAAAW

>Bacillus velezensis AQZ72212.1

MNLVKSNKKSFILFGAALAAATLVYALLLTGTELNVAAAHAFSANAELASTLGISTAAAKKAIDIIDAASTIASIISLIGIVTGAGAISYAIVATAKTMIKKYGKKYAAAW

>Bacillus velezensis OQC80228.1

MNLVKSNKKSFILFGAALAAATLVYALLLTGTELNVAAAHAFSANAELASTLGISTAAAKKAIDIIDAASTIASIISLIGIVTGAGAISYAIVATAKTMIKKYGKKYAAAW

>Bacillus velezensis ARB34496.1

MNLVKSNKKSFILFGAALAAATLVYALLLTGTELNVAAAHAFSANAELASTLGISTAAAKKAIDIIDAASTIASIISLIGIVTGAGAISYAIVATAKTMIKKYGKKYAAAW

>Bacillus velezensis OQV40116.1

MNLVKSNKKSFILFGAALAAATLVYALLLTGTELNVAAAHAFSANAELASTLGISTAAAKKAIDIIDAASTIASIISLIGIVTGAGAISYAIVATAKTMIKKYGKKYAAAW

>Bacillus velezensis OQV50917.1

MNLVKSNKKSFILFGAALAAATLVYALLLTGTELNVAAAHAFSANAELASTLGISTAAAKKAIDIIDAASTIASIISLIGIVTGAGAISYAIVATAKTMIKKYGKKYAAAW

>Bacillus velezensis OQV53653.1

MNLVKSNKKSFILFGAALAAATLVYALLLTGTELNVAAAHAFSANAELASTLGISTAAAKKAIDIIDAASTIASIISLIGIVTGAGAISYAIVATAKTMIKKYGKKYAAAW

>Bacillus velezensis OQV55676.1

MNLVKSNKKSFILFGAALAAATLVYALLLTGTELNVAAAHAFSANAELASTLGISTAAAKKAIDIIDAASTIASIISLIGIVTGAGAISYAIVATAKTMIKKYGKKYAAAW

>Bacillus velezensis OQV61167.1

MNLVKSNKKSFILFGAALAAATLVYALLLTGTELNVAAAHAFSANAELASTLGISTAAAKKAIDIIDAASTIASIISLIGIVTGAGAISYAIVATAKTMIKKYGKKYAAAW

>Bacillus velezensis OQV62233.1

MNLVKSNKKSFILFGAALAAATLVYALLLTGTELNVAAAHAFSANAELASTLGISTAAAKKAIDIIDAASTIASIISLIGIVTGAGAISYAIVATAKTMIKKYGKKYAAAW

>Bacillus velezensis ARJ76287.1

MNLVKSNKKSFILFGAALAAATLVYALLLTGTELNVAAAHAFSANAELASTLGISTAAAKKAIDIIDAASTIASIISLIGIVTGAGAISYAIVATAKTMIKKYGKKYAAAW

>Bacillus velezensis ARM29043.1

MNLVKSNKKSFILFGAALAAATLVYALLLTGTELNVAAAHAFSANAELASTLGISTAAAKKAIDIIDAASTIASIISLIGIVTGAGAISYAIVATAKTMIKKYGKKYAAAW

>Bacillus velezensis ARN86275.1

MNLVKSNKKSFILFGAALAAATLVYALLLTGTELNVAAAHAFSANAELASTLGISTAAAKKAIDIIDAASTIASIISLIGIVTGAGAISYAIVATAKTMIKKYGKKYAAAW

>Bacillus velezensis OWP60096.1

MNLVKSNKKSFILFGAALAAATLVYALLLTGTELNVAAAHAFSANAELASTLGISTAAAKKAIDIIDAASTIASIISLIGIVTGAGAISYAIVATAKTMIKKYGKKYAAAW

>Bacillus velezensis ASF56335.1

MNLVKSNKKSFILFGAALAAATLVYALLLTGTELNVAAAHAFSANAELASTLGISTAAAKKAIDIIDAASTIASIISLIGIVTGAGAISYAIVATAKTMIKKYGKKYAAAW

>Bacillus velezensis ASK59655.1

MNLVKSNKKSFILFGAALAAATLVYALLLTGTELNVAAAHAFSANAELASTLGISTAAAKKAIDIIDAASTIASIISLIGIVTGAGAISYAIVATAKTMIKKYGKKYAAAW

>Bacillus velezensis ASP23908.1

MNLVKSNKKSFILFGAALAAATLVYALLLTGTELNVAAAHAFSANAELASTLGISTAAAKKAIDIIDAASTIASIISLIGIVTGAGAISYAIVATAKTMIKKYGKKYAAAW

>Bacillus velezensis OYD10509.1

MNLVKSNKKSFILFGAALAAATLVYALLLTGTELNVAAAHAFSANAELASTLGISTAAAKKAIDIIDAASTIASIISLIGIVTGAGAISYAIVATAKTMIKKYGKKYAAAW

>Bacillus velezensis PAB04836.1

MNLVKSNKKSFILFGAALAAATLVYALLLTGTELNVAAAHAFSANAELASTLGISTAAAKKAIDIIDAASTIASIISLIGIVTGAGAISYAIVATAKTMIKKYGKKYAAAW

>Bacillus velezensis PAC78692.1

MNLVKSNKKSFILFGAALAAATLVYALLLTGTELNVAAAHAFSANAELASTLGISTAAAKKAIDIIDAASTIASIISLIGIVTGAGAISYAIVATAKTMIKKYGKKYAAAW

>Bacillus velezensis PAD05495.1

MNLVKSNKKSFILFGAALAAATLVYALLLTGTELNVAAAHAFSANAELASTLGISTAAAKKAIDIIDAASTIASIISLIGIVTGAGAISYAIVATAKTMIKKYGKKYAAAW

>Bacillus velezensis PAD64202.1

MNLVKSNKKSFILFGAALAAATLVYALLLTGTELNVAAAHAFSANAELASTLGISTAAAKKAIDIIDAASTIASIISLIGIVTGAGAISYAIVATAKTMIKKYGKKYAAAW

>Bacillus velezensis PAE34172.1

MNLVKSNKKSFILFGAALAAATLVYALLLTGTELNVAAAHAFSANAELASTLGISTAAAKKAIDIIDAASTIASIISLIGIVTGAGAISYAIVATAKTMIKKYGKKYAAAW

>Bacillus velezensis PAE77274.1

MNLVKSNKKSFILFGAALAAATLVYALLLTGTELNVAAAHAFSANAELASTLGISTAAAKKAIDIIDAASTIASIISLIGIVTGAGAISYAIVATAKTMIKKYGKKYAAAW

>Bacillus velezensis PAE99657.1

MNLVKSNKKSFILFGAALAAATLVYALLLTGTELNVAAAHAFSANAELASTLGISTAAAKKAIDIIDAASTIASIISLIGIVTGAGAISYAIVATAKTMIKKYGKKYAAAW

>Bacillus velezensis PAK29737.1

MNLVKSNKKSFILFGAALAAATLVYALLLTGTELNVAAAHAFSANAELASTLGISTAAAKKAIDIIDAASTIASIISLIGIVTGAGAISYAIVATAKTMIKKYGKKYAAAW

>Bacillus velezensis ATL40769.1

MNLVKSNKKSFILFGAALAAATLVYALLLTGTELNVAAAHAFSANAELASTLGISTAAAKKAIDIIDAASTIASIISLIGIVTGAGAISYAIVATAKTMIKKYGKKYAAAW

>Bacillus velezensis ATO08608.1

MNLVKSNKKSFILFGAALAAATLVYALLLTGTELNVAAAHAFSANAELASTLGISTAAAKKAIDIIDAASTIASIISLIGIVTGAGAISYAIVATAKTMIKKYGKKYAAAW

>Bacillus velezensis PHQ05733.1

MNLVKSNKKSFILFGAALAAATLVYALLLTGTELNVAAAHAFSANAELASTLGISTAAAKKAIDIIDAASTIASIISLIGIVTGAGAISYAIVATAKTMIKKYGKKYAAAW

>Bacillus velezensis BBA77528.1

MNLVKSNKKSFILFGAALAAATLVYALLLTGTELNVAAAHAFSANAELASTLGISTAAAKKAIDIIDAASTIASIISLIGIVTGAGAISYAIVATAKTMIKKYGKKYAAAW

>Bacillus siamensis PIK29807.1

MNLVKSNKKSFILFGAALAAATLVYALLLTGTELNVAAAHAFSANAELASTLGISTAAAKKAIDIIDAAS

TIASIISLIGIVTGAGAISYAIVATAKTMIKKYGKKYAAAW

>Bacillus subtilis ATU27985.1

MNLVKSNKKSFILFGAALAAATLVYALLLTGTELNVAAAHAFSANAELASTLGISTAAAKKAIDIIDAASTIASIISLIGIVTGAGAISYAIVATAKTMIKKYGKKYAAAW

>Bacillus velezensis ATV02066.1

MNLVKSNKKSFILFGAALAAATLVYALLLTGTELNVAAAHAFSANAELASTLGISTAAAKKAIDIIDAASTIASIISLIGIVTGAGAISYAIVATAKTMIKKYGKKYAAAW

>Bacillus sp. Lzh-5 ATV24019.1

MNLVKSNKKSFILFGAALAAATLVYALLLTGTELNVAAAHAFSANAELASTLGISTAAAKKAIDIIDAASTIASIISLIGIVTGAGAISYAIVATAKTMIKKYGKKYAAAW

>Bacillus velezensis ATX84682.1

MNLVKSNKKSFILFGAALAAATLVYALLLTGTELNVAAAHAFSANAELASTLGISTAAAKKAIDIIDAASTIASIISLIGIVTGAGAISYAIVATAKTMIKKYGKKYAAAW

>Bacillus velezensis AUJ60072.1

MNLVKSNKKSFILFGAALAAATLVYALLLTGTELNVAAAHAFSANAELASTLGISTAAAKKAIDIIDAASTIASIISLIGIVTGAGAISYAIVATAKTMIKKYGKKYAAAW

>Bacillus velezensis ATY29557.1

MNLVKSNKKSFILFGAALAAATLVYALLLTGTELNVAAAHAFSANAELASTLGISTAAAKKAIDIIDAASTIASIISLIGIVTGAGAISYAIVATAKTMIKKYGKKYAAAW

>Bacillus velezensis PJN83896.1

MNLVKSNKKSFILFGAALAAATLVYALLLTGTELNVAAAHAFSANAELASTLGISTAAAKKAIDIIDAASTIASIISLIGIVTGAGAISYAIVATAKTMIKKYGKKYAAAW

>Bacillus velezensis PKF83794.1

MNLVKSNKKSFILFGAALAAATLVYALLLTGTELNVAAAHAFSANAELASTLGISTAAAKKAIDIIDAASTIASIISLIGIVTGAGAISYAIVATAKTMIKKYGKKYAAAW

>Bacillus velezensis AUG37295.1

MNLVKSNKKSFILFGAALAAATLVYALLLTGTELNVAAAHAFSANAELASTLGISTAAAKKAIDIIDAASTIASIISLIGIVTGAGAISYAIVATAKTMIKKYGKKYAAAW

>Bacillus siamensis AUJ76174.1

MNLVKSNKKSFILFGAALAAATLVYALLLTGTELNVAAAHAFSANAELASTLGISTAAAKKAIDIIDAASTIASIISLIGIVTGAGAISYAIVATAKTMIKKYGKKYAAAW

>Bacillus velezensis AUS17155.1

MNLVKSNKKSFILFGAALAAATLVYALLLTGTELNVAAAHAFSANAELASTLGISTAAAKKAIDIIDAASTIASIISLIGIVTGAGAISYAIVATAKTMIKKYGKKYAAAW

>Bacillus velezensis POI15437.1

MNLVKSNKKSFILFGAALAAATLVYALLLTGTELNVAAAHAFSANAELASTLGISTAAAKKAIDIIDAASTIASIISLIGIVTGAGAISYAIVATAKTMIKKYGKKYAAAW

>Bacillus velezensis POR14200.1

MNLVKSNKKSFILFGAALAAATLVYALLLTGTELNVAAAHAFSANAELASTLGISTAAAKKAIDIIDAASTIASIISLIGIVTGAGAISYAIVATAKTMIKKYGKKYAAAW

>Bacillus velezensis AVB08933.1

MNLVKSNKKSFILFGAALAAATLVYALLLTGTELNVAAAHAFSANAELASTLGISTAAAKKAIDIIDAASTIASIISLIGIVTGAGAISYAIVATAKTMIKKYGKKYAAAW

>Bacillus sp. SDLI1 AME07572.1

MNLVKSNKKSFILFGAALAAATLVYALLLTGTELNVAAAHAFSANAELASTLGISTAAAKKAIDIIDAASTIASIISLIGIVTGAGAISYAIVATAKTMIKKYGKKYAAAW

>Bacillus siamensis PAD64202.1

MNLVKSNKKSFILFGAALAAATLVYALLLTGTELNVAAAHAFSANAELASTLGISTAAAKKAIDIIDAASTIASIISLIGIVTGAGAISYAIVATAKTMIKKYGKKYAAAW

>Bacillus sp. LYLB4 OXS80963.1

MNLVKSNKKSFILFGAALAAATLVYALLLTGTELNVAAAHAFSANAELASTLGISTAAAKKAIDIIDAASTIASIISLIGIVTGAGAISYAIVATAKTMIKKYGKKYAAAW

>Bacillus velezensis CCF06485.1

MMNLVKSNKKSFILFGAALAAATLVYALLLTGTELNVAAAHAFSANAELASTLGISTAAAKKAIDIIDAASTIASIISLIGIVTGAGAISYAIVATAKTMIKKYGKKYAAAW

>Bacillus amyloliquefaciens AEK90387.1

MMNLVKSNKKSFILFGAALAAATLVYALLLTGTELNVAAAHAFSANAELASTLGISTAAAKKAIDIIDAASTIASIISLIGIVTGAGAISYAIVATAKTMIKKYGKKYAAAW

>Bacillus amyloliquefaciens AFJ63383.1

MMNLVKSNKKSFILFGAALAAATLVYALLLTGTELNVAAAHAFSANAELASTLGISTAAAKKAIDIIDAASTIASIISLIGIVTGAGAISYAIVATAKTMIKKYGKKYAAAW

>Bacillus amyloliquefaciens AGZ57729.1

MMNLVKSNKKSFILFGAALAAATLVYALLLTGTELNVAAAHAFSANAELASTLGISTAAAKKAIDIIDAASTIASIISLIGIVTGAGAISYAIVATAKTMIKKYGKKYAAAW

>Bacillus amyloliquefaciens AJK66622.1

MMNLVKSNKKSFILFGAALAAATLVYALLLTGTELNVAAAHAFSANAELASTLGISTAAAKKAIDIIDAASTIASIISLIGIVTGAGAISYAIVATAKTMIKKYGKKYAAAW

>Bacillus amyloliquefaciens CUB23002.1

MMNLVKSNKKSFILFGAALAAATLVYALLLTGTELNVAAAHAFSANAELASTLGISTAAAKKAIDIIDAASTIASIISLIGIVTGAGAISYAIVATAKTMIKKYGKKYAAAW

>Bacillus amyloliquefaciens CUB38513.1

MMNLVKSNKKSFILFGAALAAATLVYALLLTGTELNVAAAHAFSANAELASTLGISTAAAKKAIDIIDAASTIASIISLIGIVTGAGAISYAIVATAKTMIKKYGKKYAAAW

>Bacillus amyloliquefaciens KYC90196.1

MMNLVKSNKKSFILFGAALAAATLVYALLLTGTELNVAAAHAFSANAELASTLGISTAAAKKAIDIIDAASTIASIISLIGIVTGAGAISYAIVATAKTMIKKYGKKYAAAW

>Bacillus amyloliquefaciens APQ51496.1

MMNLVKSNKKSFILFGAALAAATLVYALLLTGTELNVAAAHAFSANAELASTLGISTAAAKKAIDIIDAASTIASIISLIGIVTGAGAISYAIVATAKTMIKKYGKKYAAAW

>Bacillus velezensis CCF06485.1

MMNLVKSNKKSFILFGAALAAATLVYALLLTGTELNVAAAHAFSANAELASTLGISTAAAKKAIDIIDAASTIASIISLIGIVTGAGAISYAIVATAKTMIKKYGKKYAAAW

>Bacillus velezensis CCG51133.1

MMNLVKSNKKSFILFGAALAAATLVYALLLTGTELNVAAAHAFSANAELASTLGISTAAAKKAIDIIDAASTIASIISLIGIVTGAGAISYAIVATAKTMIKKYGKKYAAAW

>Bacillus velezensis CCP22855.1

MMNLVKSNKKSFILFGAALAAATLVYALLLTGTELNVAAAHAFSANAELASTLGISTAAAKKAIDIIDAASTIASIISLIGIVTGAGAISYAIVATAKTMIKKYGKKYAAAW

>Bacillus velezensis CDG31019.1

MMNLVKSNKKSFILFGAALAAATLVYALLLTGTELNVAAAHAFSANAELASTLGISTAAAKKAIDIIDAASTIASIISLIGIVTGAGAISYAIVATAKTMIKKYGKKYAAAW

>Bacillus velezensis CDG27127.1

MMNLVKSNKKSFILFGAALAAATLVYALLLTGTELNVAAAHAFSANAELASTLGISTAAAKKAIDIIDAASTIASIISLIGIVTGAGAISYAIVATAKTMIKKYGKKYAAAW

>Bacillus velezensis CDH96883.1

MMNLVKSNKKSFILFGAALAAATLVYALLLTGTELNVAAAHAFSANAELASTLGISTAAAKKAIDIIDAASTIASIISLIGIVTGAGAISYAIVATAKTMIKKYGKKYAAAW

>Bacillus velezensis AHZ17210.1

MMNLVKSNKKSFILFGAALAAATLVYALLLTGTELNVAAAHAFSANAELASTLGISTAAAKKAIDIIDAASTIASIISLIGIVTGAGAISYAIVATAKTMIKKYGKKYAAAW

>Bacillus velezensis AIU83086.1

MMNLVKSNKKSFILFGAALAAATLVYALLLTGTELNVAAAHAFSANAELASTLGISTAAAKKAIDIIDAASTIASIISLIGIVTGAGAISYAIVATAKTMIKKYGKKYAAAW

>Bacillus velezensis AKD31190.1

MMNLVKSNKKSFILFGAALAAATLVYALLLTGTELNVAAAHAFSANAELASTLGISTAAAKKAIDIIDAASTIASIISLIGIVTGAGAISYAIVATAKTMIKKYGKKYAAAW

>Bacillus velezensis AKL77603.1

MMNLVKSNKKSFILFGAALAAATLVYALLLTGTELNVAAAHAFSANAELASTLGISTAAAKKAIDIIDAASTIASIISLIGIVTGAGAISYAIVATAKTMIKKYGKKYAAAW

>Bacillus velezensis CUX94729.1

MMNLVKSNKKSFILFGAALAAATLVYALLLTGTELNVAAAHAFSANAELASTLGISTAAAKKAIDIIDAASTIASIISLIGIVTGAGAISYAIVATAKTMIKKYGKKYAAAW

>Bacillus velezensis ANF37935.1

MMNLVKSNKKSFILFGAALAAATLVYALLLTGTELNVAAAHAFSANAELASTLGISTAAAKKAIDIIDAASTIASIISLIGIVTGAGAISYAIVATAKTMIKKYGKKYAAAW

>Bacillus velezensis OBR33854.1

MMNLVKSNKKSFILFGAALAAATLVYALLLTGTELNVAAAHAFSANAELASTLGISTAAAKKAIDIIDAASTIASIISLIGIVTGAGAISYAIVATAKTMIKKYGKKYAAAW

>Bacillus velezensis OCB92620.1

MMNLVKSNKKSFILFGAALAAATLVYALLLTGTELNVAAAHAFSANAELASTLGISTAAAKKAIDIIDAASTIASIISLIGIVTGAGAISYAIVATAKTMIKKYGKKYAAAW

>Bacillus velezensis ODS08713.1

MMNLVKSNKKSFILFGAALAAATLVYALLLTGTELNVAAAHAFSANAELASTLGISTAAAKKAIDIIDAASTIASIISLIGIVTGAGAISYAIVATAKTMIKKYGKKYAAAW

>Bacillus velezensis SDJ57659.1

MMNLVKSNKKSFILFGAALAAATLVYALLLTGTELNVAAAHAFSANAELASTLGISTAAAKKAIDIIDAASTIASIISLIGIVTGAGAISYAIVATAKTMIKKYGKKYAAAW

>Bacillus velezensis AQS45333.1

MMNLVKSNKKSFILFGAALAAATLVYALLLTGTELNVAAAHAFSANAELASTLGISTAAAKKAIDIIDAASTIASIISLIGIVTGAGAISYAIVATAKTMIKKYGKKYAAAW

>Bacillus velezensis ARZ59391.1

MMNLVKSNKKSFILFGAALAAATLVYALLLTGTELNVAAAHAFSANAELASTLGISTAAAKKAIDIIDAASTIASIISLIGIVTGAGAISYAIVATAKTMIKKYGKKYAAAW

>Bacillus velezensis ASB54456.1

MMNLVKSNKKSFILFGAALAAATLVYALLLTGTELNVAAAHAFSANAELASTLGISTAAAKKAIDIIDAASTIASIISLIGIVTGAGAISYAIVATAKTMIKKYGKKYAAAW

>Bacillus velezensis ASB66845.1

MMNLVKSNKKSFILFGAALAAATLVYALLLTGTELNVAAAHAFSANAELASTLGISTAAAKKAIDIIDAASTIASIISLIGIVTGAGAISYAIVATAKTMIKKYGKKYAAAW

>Bacillus velezensis ASS63500.1

MMNLVKSNKKSFILFGAALAAATLVYALLLTGTELNVAAAHAFSANAELASTLGISTAAAKKAIDIIDAASTIASIISLIGIVTGAGAISYAIVATAKTMIKKYGKKYAAAW

>Bacillus velezensis ATC50532.1

MMNLVKSNKKSFILFGAALAAATLVYALLLTGTELNVAAAHAFSANAELASTLGISTAAAKKAIDIIDAASTIASIISLIGIVTGAGAISYAIVATAKTMIKKYGKKYAAAW

>Bacillus velezensis ATD74285.1

MMNLVKSNKKSFILFGAALAAATLVYALLLTGTELNVAAAHAFSANAELASTLGISTAAAKKAIDIIDAASTIASIISLIGIVTGAGAISYAIVATAKTMIKKYGKKYAAAW

>Bacillus amyloliquefaciens PLT51586.1

MMNLVKSNKKSFILFGAALAAATLVYALLLTGTELNVAAAHAFSANAELASTLGISTAAAKKAIDIIDAASTIASIISLIGIVTGAGAISYAIVATAKTMIKKYGKKYAAAW

>Bacillus sp. RUPDJ SIR95129.1

MMNLVKSNKKSFILFGAALAAATLVYALLLTGTELNVAAAHAFSANAELASTLGISTAAAKKAIDIIDAASTIASIISLIGIVTGAGAISYAIVATAKTMIKKYGKKYAAAW

>Bacillus sp. GZB OMQ04971.1

MMNLVKSNKKSFILFGAALAAATLVYALLLTGTELNVAAAHAFSANAELASTLGISTAAAKKAIDIIDAASTIASIISLIGIVTGAGAISYAIVATAKTMIKKYGKKYAAAW

>Bacillus sp. 275 AQP97092.1

MMNLVKSNKKSFILFGAALAAATLVYALLLTGTELNVAAAHAFSANAELASTLGISTAAAKKAIDIIDAASTIASIISLIGIVTGAGAISYAIVATAKTMIKKYGKKYAAAW

>Bacillus velezensis AOU02193.1

MNLVKSNKKSFILFGAALAAATLVYALLLTGTELNVAAAHVFSANAELASTLGISTAAAKKAIDIIDAAS

TIASIISLIGIVTGAGAISYAIVATAKTMIKKYGKKYAAAW

>Bacillus subtilis AEP92227.1

MNLVKSNKKSFILFGAALAAATLVYALLLTGTELNVAAAHAFSANAELASTLGISTAAAKKAIDIIDAASTVASIISLIGIVTGAGAISYAIVATAKTMIKKYGKKYAAAW

>Bacillus sp. EGD-AK10 ERI43560.1

MNLVKSNKKSFILFGAALAAATLVYALLLTGTELNVAAAHAFSANAELASTLGISTAAAKKAIDIIDAASTVASIISLIGIVTGAGAISYAIVATAKTMIKKYGKKYAAAW

>Bacillus amyloliquefaciens KZE57960.1

MNLVKSNKKSFILFGAALAAATLVYALLLTGTELNVAAAHAFSANAELASTLGISTAAAKKAIDIIDAASTVASIISLIGIVTGAGAISYAIVATAKTMIKKYGKKYAAAW

>Bacillus amyloliquefaciens OPD44409.1

MNLVKSNKKSFILFGVALAAATLVYALLLTGTELNVAAAHAFSANAELASTLGISTAAAKKAIDIIDAASTIASIISLIGIVTGAGAISYAIVATAKTMIKKYGKKYAAAW

>Bacillus velezensis ASZ05280.1

MNLVKSNKKSFILFGVALAAATLVYALLLTGTELNVAAAHAFSANAELASTLGISTAAAKKAIDIIDAASTIASIISLIGIVTGAGAISYAIVATAKTMIKKYGKKYAAAW

>Bacillus velezensis PII43083.1

MNLVKSNKKSFILFGVALAAATLVYALLLTGTELNVAAAHAFSANAELASTLGISTAAAKKAIDIIDAASTIASIISLIGIVTGAGAISYAIVATAKTMIKKYGKKYAAAW

>Bacillus subtilis EFG92012.1

MNLVKSNKKSFVLFGAALAAATLVYALLLTGTELNVAAAHAFSANAELASTLGISTAAAKKAIDIIDAASTVASIISLIGIVTGAGAISYAIVATAKSMIKKYGKKYAAAW

>Bacillus subtilis ADM39168.1

MNLVKSNKKSFVLFGAALAAATLVYALLLTGTELNVAAAHAFSANAELASTLGISTAAAKKAIDIIDAASTVASIISLIGIVTGAGAISYAIVATAKSMIKKYGKKYAAAW

>Bacillus subtilis KFK77618.1

MNLVKSNKKSFVLFGAALAAATLVYALLLTGTELNVAAAHAFSANAELASTLGISTAAAKKAIDIIDAASTVASIISLIGIVTGAGAISYAIVATAKSMIKKYGKKYAAAW

>Bacillus subtilis AJD37641.1

MNLVKSNKKSFVLFGAALAAATLVYALLLTGTELNVAAAHAFSANAELASTLGISTAAAKKAIDIIDAASTVASIISLIGIVTGAGAISYAIVATAKSMIKKYGKKYAAAW

>Bacillus subtilis AJW84678.1

MNLVKSNKKSFVLFGAALAAATLVYALLLTGTELNVAAAHAFSANAELASTLGISTAAAKKAIDIIDAASTVASIISLIGIVTGAGAISYAIVATAKSMIKKYGKKYAAAW

>Bacillus licheniformis ARW41388.1

MMNLVKSNKKSFILFGAALAAATLVYALLLTGTELNVAAAHAFSANAELASTLGISTAAAKKAIDIIDAASTIASIISLIGIVTGAGAISYAIVATAKTMIKKYGKKYAAAW

>Bacillus nakamurai KXZ24165.1

MNLVKSNKKSFLLFGAALAAAALVYALLLTGTELNVAAAHTFSANAELASTLGISAAAAKKAIDIIDAASTIASIISLIGIVTGAGAISYAIVATAKTMIKKYGKKYAAAW

>Bacillus siamensis OAZ60702.1

MNLVKSNKKSFILFGAALAAATLVYALLLTGTELNVAAAHAFSANAELASTLGISTAAAKKAIDIIDAASTIASIISLIGIVTGAGAISYAIVATAKTMIKKYGKKYAAAW

>Enterococcus faecium BAT21392.1/Enterocin NKR-5-3B

MKKNLLLVLPIVGIVGLFVGAPMLTANLGISSYAAKKVIDIINTGSAVATIIALVTAVVGGGLITAGIVATAKSLIKKYGAKYAAAW

>Enterococcus faecium BAU40203.1

MKKNLLLVLPIVGIVGLFVGAPMLTANLGISSYAAKKVIDIINTGSAVATIIALVTAVVGGGLITAGIVATAKSLIKKYGAKYAAAW

>Enterococcus thailandicus OAQ55116.1

MKKNLLLVLPIVGIVGLFVGAPMLTANLGISSYAAKKVIDIINTGSAVATIIALVTAVVGGGLITAGIVATAKSLIKKYGAKYAAAW

>Enterococcus thailandicus OJG94406.1

MKKNLLLVLPIVGIVGLFVGAPMLTANLGISSYAAKKVIDIINTGSAVATIIALVTAVVGGGLITAGIVATAKSLIKKYGAKYAAAW

>Enterococcus sp. 5B7_DIV0075 OTP22298.1

MKKNLLLVLPIVGIVGLFVGAPMLTANLGISSYAAKKVIDIINTGSAVATIIALVTAVVGGGLITAGIVATAKSLIKKYGAKYAAAW

>Enterococcus thailandicus ASZ08576.1

MKKNLLLVLPIVGIVGLFVGAPMLTANLGISSYAAKKVIDIINTGSAVATIIALVTAVVGGGLITAGIVATAKSLIKKYGAKYAAAW

>Enterococcus-faecium-1,231,502_EEV46607.1

MKKNLLLVLPILGFAGFFVGVPMLSANIGISSYAAKKVIDIINTGSTVATIISIVAAVVGGGLITAGIVATAKSLIKKYGAKYAAAW

>Enterococcus-faecium-1,231,410_EEV54322.1

MKKNLLLVLPILGFAGFFVGVPMLSANIGISSYAAKKVIDIINTGSTVATIISIVAAVVGGGLITAGIVATAKSLIKKYGAKYAAAW

>Enterococcus-faecium-TC-6_EEW64128.1

MKKNLLLVLPILGFAGFFVGVPMLSANIGISSYAAKKVIDIINTGSTVATIISIVAAVVGGGLITAGIVATAKSLIKKYGAKYAAAW

>Enterococcus-faecium-E1679_EFF26988.1

MKKNLLLVLPILGFAGFFVGVPMLSANIGISSYAAKKVIDIINTGSTVATIISIVAAVVGGGLITAGIVATAKSLIKKYGAKYAAAW

>Enterococcus-faecium-U0317_EFF28479.1

MKKNLLLVLPILGFAGFFVGVPMLSANIGISSYAAKKVIDIINTGSTVATIISIVAAVVGGGLITAGIVATAKSLIKKYGAKYAAAW

>Enterococcus-faecium-TX0133a01_EFR67509.1

MKKNLLLVLPILGFAGFFVGVPMLSANIGISSYAAKKVIDIINTGSTVATIISIVAAVVGGGLITAGIVATAKSLIKKYGAKYAAAW

>Enterococcus-faecium-R501_EJX39301.1

MKKNLLLVLPILGFAGFFVGVPMLSANIGISSYAAKKVIDIINTGSTVATIISIVAAVVGGGLITAGIVATAKSLIKKYGAKYAAAW

>Enterococcus-faecium-S447_EJX40012.1

MKKNLLLVLPILGFAGFFVGVPMLSANIGISSYAAKKVIDIINTGSTVATIISIVAAVVGGGLITAGIVATAKSLIKKYGAKYAAAW

>Enterococcus-faecium-V689_EJX40364.1

MKKNLLLVLPILGFAGFFVGVPMLSANIGISSYAAKKVIDIINTGSTVATIISIVAAVVGGGLITAGIVATAKSLIKKYGAKYAAAW

>Enterococcus-faecium-R499_EJX46660.1

MKKNLLLVLPILGFAGFFVGVPMLSANIGISSYAAKKVIDIINTGSTVATIISIVAAVVGGGLITAGIVATAKSLIKKYGAKYAAAW

>Enterococcus-faecium-R496_EJX47823.1

MKKNLLLVLPILGFAGFFVGVPMLSANIGISSYAAKKVIDIINTGSTVATIISIVAAVVGGGLITAGIVATAKSLIKKYGAKYAAAW

>Enterococcus-faecium-R497_EJX49214.1

MKKNLLLVLPILGFAGFFVGVPMLSANIGISSYAAKKVIDIINTGSTVATIISIVAAVVGGGLITAGIVATAKSLIKKYGAKYAAAW

>Enterococcus-faecium-R494_EJX53820.1

MKKNLLLVLPILGFAGFFVGVPMLSANIGISSYAAKKVIDIINTGSTVATIISIVAAVVGGGLITAGIVATAKSLIKKYGAKYAAAW

>Enterococcus-faecium-R446_EJX59968.1

MKKNLLLVLPILGFAGFFVGVPMLSANIGISSYAAKKVIDIINTGSTVATIISIVAAVVGGGLITAGIVATAKSLIKKYGAKYAAAW

>Enterococcus-faecium-P1986_EJX61016.1

MKKNLLLVLPILGFAGFFVGVPMLSANIGISSYAAKKVIDIINTGSTVATIISIVAAVVGGGLITAGIVATAKSLIKKYGAKYAAAW

>Enterococcus-faecium-P1140_EJX66701.1

MKKNLLLVLPILGFAGFFVGVPMLSANIGISSYAAKKVIDIINTGSTVATIISIVAAVVGGGLITAGIVATAKSLIKKYGAKYAAAW

>Enterococcus-faecium-P1190_EJX66841.1

MKKNLLLVLPILGFAGFFVGVPMLSANIGISSYAAKKVIDIINTGSTVATIISIVAAVVGGGLITAGIVATAKSLIKKYGAKYAAAW

>Enterococcus-faecium-P1137_EJX69593.1

MKKNLLLVLPILGFAGFFVGVPMLSANIGISSYAAKKVIDIINTGSTVATIISIVAAVVGGGLITAGIVATAKSLIKKYGAKYAAAW

>Enterococcus-faecium-P1139_EJX69942.1

MKKNLLLVLPILGFAGFFVGVPMLSANIGISSYAAKKVIDIINTGSTVATIISIVAAVVGGGLITAGIVATAKSLIKKYGAKYAAAW

>Enterococcus-faecium-ERV99_EJX80995.1

MKKNLLLVLPILGFAGFFVGVPMLSANIGISSYAAKKVIDIINTGSTVATIISIVAAVVGGGLITAGIVATAKSLIKKYGAKYAAAW

>Enterococcus-faecium-P1123_EJX81010.1

MKKNLLLVLPILGFAGFFVGVPMLSANIGISSYAAKKVIDIINTGSTVATIISIVAAVVGGGLITAGIVATAKSLIKKYGAKYAAAW

>Enterococcus-faecium-ERV168_EJX90113.1

MKKNLLLVLPILGFAGFFVGVPMLSANIGISSYAAKKVIDIINTGSTVATIISIVAAVVGGGLITAGIVATAKSLIKKYGAKYAAAW

>Enterococcus-faecium-ERV26_EJX92184.1

MKKNLLLVLPILGFAGFFVGVPMLSANIGISSYAAKKVIDIINTGSTVATIISIVAAVVGGGLITAGIVATAKSLIKKYGAKYAAAW

>Enterococcus-faecium-ERV161_EJX97589.1

MKKNLLLVLPILGFAGFFVGVPMLSANIGISSYAAKKVIDIINTGSTVATIISIVAAVVGGGLITAGIVATAKSLIKKYGAKYAAAW

>Enterococcus-faecium-ERV102_EJX99890.1

MKKNLLLVLPILGFAGFFVGVPMLSANIGISSYAAKKVIDIINTGSTVATIISIVAAVVGGGLITAGIVATAKSLIKKYGAKYAAAW

>Enterococcus-faecium-ERV165_EJY00997.1

MKKNLLLVLPILGFAGFFVGVPMLSANIGISSYAAKKVIDIINTGSTVATIISIVAAVVGGGLITAGIVATAKSLIKKYGAKYAAAW

>Enterococcus-faecium-E422_EJY10040.1

MKKNLLLVLPILGFAGFFVGVPMLSANIGISSYAAKKVIDIINTGSTVATIISIVAAVVGGGLITAGIVATAKSLIKKYGAKYAAAW

>Enterococcus-faecium-C621_EJY12684.1

MKKNLLLVLPILGFAGFFVGVPMLSANIGISSYAAKKVIDIINTGSTVATIISIVAAVVGGGLITAGIVATAKSLIKKYGAKYAAAW

>Enterococcus-faecium-C1904_EJY16905.1

MKKNLLLVLPILGFAGFFVGVPMLSANIGISSYAAKKVIDIINTGSTVATIISIVAAVVGGGLITAGIVATAKSLIKKYGAKYAAAW

>Enterococcus-faecium-C497_EJY17635.1

MKKNLLLVLPILGFAGFFVGVPMLSANIGISSYAAKKVIDIINTGSTVATIISIVAAVVGGGLITAGIVATAKSLIKKYGAKYAAAW

>Enterococcus-faecium-515_EJY21857.1

MKKNLLLVLPILGFAGFFVGVPMLSANIGISSYAAKKVIDIINTGSTVATIISIVAAVVGGGLITAGIVATAKSLIKKYGAKYAAAW

>Enterococcus-faecium-511_EJY31109.1

MKKNLLLVLPILGFAGFFVGVPMLSANIGISSYAAKKVIDIINTGSTVATIISIVAAVVGGGLITAGIVATAKSLIKKYGAKYAAAW

>Enterococcus-faecium-513_EJY32524.1

MKKNLLLVLPILGFAGFFVGVPMLSANIGISSYAAKKVIDIINTGSTVATIISIVAAVVGGGLITAGIVATAKSLIKKYGAKYAAAW

>Enterococcus-faecium-509_EJY38862.1

MKKNLLLVLPILGFAGFFVGVPMLSANIGISSYAAKKVIDIINTGSTVATIISIVAAVVGGGLITAGIVATAKSLIKKYGAKYAAAW

>Enterococcus-faecium-EnGen0002_ELA74634.1

MKKNLLLVLPILGFAGFFVGVPMLSANIGISSYAAKKVIDIINTGSTVATIISIVAAVVGGGLITAGIVATAKSLIKKYGAKYAAAW

>Enterococcus-faecium-EnGen0016_ELA83179.1

MKKNLLLVLPILGFAGFFVGVPMLSANIGISSYAAKKVIDIINTGSTVATIISIVAAVVGGGLITAGIVATAKSLIKKYGAKYAAAW

>Enterococcus-faecium-EnGen0024_ELB33462.1

MKKNLLLVLPILGFAGFFVGVPMLSANIGISSYAAKKVIDIINTGSTVATIISIVAAVVGGGLITAGIVATAKSLIKKYGAKYAAAW

>Enterococcus-faecium-EnGen0034_ELB46684.1

MKKNLLLVLPILGFAGFFVGVPMLSANIGISSYAAKKVIDIINTGSTVATIISIVAAVVGGGLITAGIVATAKSLIKKYGAKYAAAW

>Enterococcus-faecium-EnGen0051_ELB68647.1

MKKNLLLVLPILGFAGFFVGVPMLSANIGISSYAAKKVIDIINTGSTVATIISIVAAVVGGGLITAGIVATAKSLIKKYGAKYAAAW

>Enterococcus-faecium-EnGen0050_ELB69078.1

MKKNLLLVLPILGFAGFFVGVPMLSANIGISSYAAKKVIDIINTGSTVATIISIVAAVVGGGLITAGIVATAKSLIKKYGAKYAAAW

>Enterococcus-faecium-EnGen0046_ELB74181.1

MKKNLLLVLPILGFAGFFVGVPMLSANIGISSYAAKKVIDIINTGSTVATIISIVAAVVGGGLITAGIVATAKSLIKKYGAKYAAAW

>Enterococcus-faecium-EnGen0057_ELB74540.1

MKKNLLLVLPILGFAGFFVGVPMLSANIGISSYAAKKVIDIINTGSTVATIISIVAAVVGGGLITAGIVATAKSLIKKYGAKYAAAW

>Enterococcus-faecium-EnGen0049_ELB80580.1

MKKNLLLVLPILGFAGFFVGVPMLSANIGISSYAAKKVIDIINTGSTVATIISIVAAVVGGGLITAGIVATAKSLIKKYGAKYAAAW

>Enterococcus-faecium-EnGen0006_EOD82183.1

MKKNLLLVLPILGFAGFFVGVPMLSANIGISSYAAKKVIDIINTGSTVATIISIVAAVVGGGLITAGIVATAKSLIKKYGAKYAAAW

>Enterococcus-faecium-EnGen0177_EOG14414.1

MKKNLLLVLPILGFAGFFVGVPMLSANIGISSYAAKKVIDIINTGSTVATIISIVAAVVGGGLITAGIVATAKSLIKKYGAKYAAAW

>Enterococcus-faecium-EnGen0178_EOG16750.1

MKKNLLLVLPILGFAGFFVGVPMLSANIGISSYAAKKVIDIINTGSTVATIISIVAAVVGGGLITAGIVATAKSLIKKYGAKYAAAW

>Enterococcus-faecium-EnGen0179_EOG16752.1

MKKNLLLVLPILGFAGFFVGVPMLSANIGISSYAAKKVIDIINTGSTVATIISIVAAVVGGGLITAGIVATAKSLIKKYGAKYAAAW

>Enterococcus-faecium-EnGen0181_EOG25237.1

MKKNLLLVLPILGFAGFFVGVPMLSANIGISSYAAKKVIDIINTGSTVATIISIVAAVVGGGLITAGIVATAKSLIKKYGAKYAAAW

>Enterococcus-faecium-EnGen0183_EOG36014.1

MKKNLLLVLPILGFAGFFVGVPMLSANIGISSYAAKKVIDIINTGSTVATIISIVAAVVGGGLITAGIVATAKSLIKKYGAKYAAAW

>Enterococcus-faecium-EnGen0265_EOH66000.1

MKKNLLLVLPILGFAGFFVGVPMLSANIGISSYAAKKVIDIINTGSTVATIISIVAAVVGGGLITAGIVATAKSLIKKYGAKYAAAW

>Enterococcus-faecium-EnGen0314_EOI57579.1

MKKNLLLVLPILGFAGFFVGVPMLSANIGISSYAAKKVIDIINTGSTVATIISIVAAVVGGGLITAGIVATAKSLIKKYGAKYAAAW

>Enterococcus-faecium-EnGen0318_EOI61123.1

MKKNLLLVLPILGFAGFFVGVPMLSANIGISSYAAKKVIDIINTGSTVATIISIVAAVVGGGLITAGIVATAKSLIKKYGAKYAAAW

>Enterococcus-faecium-EnGen0316_EOI65812.1

MKKNLLLVLPILGFAGFFVGVPMLSANIGISSYAAKKVIDIINTGSTVATIISIVAAVVGGGLITAGIVATAKSLIKKYGAKYAAAW

>Enterococcus-faecium-EnGen0319_EOI68306.1

MKKNLLLVLPILGFAGFFVGVPMLSANIGISSYAAKKVIDIINTGSTVATIISIVAAVVGGGLITAGIVATAKSLIKKYGAKYAAAW

>Enterococcus-faecium-EnGen0321_EOI69428.1

MKKNLLLVLPILGFAGFFVGVPMLSANIGISSYAAKKVIDIINTGSTVATIISIVAAVVGGGLITAGIVATAKSLIKKYGAKYAAAW

>Enterococcus-faecium-EnGen0323_EOI69851.1

MKKNLLLVLPILGFAGFFVGVPMLSANIGISSYAAKKVIDIINTGSTVATIISIVAAVVGGGLITAGIVATAKSLIKKYGAKYAAAW

>Enterococcus-faecium-EnGen0312_EOI77500.1

MKKNLLLVLPILGFAGFFVGVPMLSANIGISSYAAKKVIDIINTGSTVATIISIVAAVVGGGLITAGIVATAKSLIKKYGAKYAAAW

>Enterococcus-faecium-EnGen0260_EOM08969.1

MKKNLLLVLPILGFAGFFVGVPMLSANIGISSYAAKKVIDIINTGSTVATIISIVAAVVGGGLITAGIVATAKSLIKKYGAKYAAAW

>Enterococcus-faecium-EnGen0261_EOM12749.1

MKKNLLLVLPILGFAGFFVGVPMLSANIGISSYAAKKVIDIINTGSTVATIISIVAAVVGGGLITAGIVATAKSLIKKYGAKYAAAW

>Enterococcus-faecium-EnGen0256_EOM37854.1

MKKNLLLVLPILGFAGFFVGVPMLSANIGISSYAAKKVIDIINTGSTVATIISIVAAVVGGGLITAGIVATAKSLIKKYGAKYAAAW

>Enterococcus-faecium-EnGen0163_EOM67517.1

MKKNLLLVLPILGFAGFFVGVPMLSANIGISSYAAKKVIDIINTGSTVATIISIVAAVVGGGLITAGIVATAKSLIKKYGAKYAAAW

>Enterococcus-faecium-EnGen0376_EOT34491.1

MKKNLLLVLPILGFAGFFVGVPMLSANIGISSYAAKKVIDIINTGSTVATIISIVAAVVGGGLITAGIVATAKSLIKKYGAKYAAAW

>Enterococcus-faecium-EnGen0375_EOT53984.1

MKKNLLLVLPILGFAGFFVGVPMLSANIGISSYAAKKVIDIINTGSTVATIISIVAAVVGGGLITAGIVATAKSLIKKYGAKYAAAW

>Enterococcus-faecium-EnGen0375_EOT90930.1

MKKNLLLVLPILGFAGFFVGVPMLSANIGISSYAAKKVIDIINTGSTVATIISIVAAVVGGGLITAGIVATAKSLIKKYGAKYAAAW

>Enterococcus-faecium-EnGen0376_EOT92150.1

MKKNLLLVLPILGFAGFFVGVPMLSANIGISSYAAKKVIDIINTGSTVATIISIVAAVVGGGLITAGIVATAKSLIKKYGAKYAAAW

>Enterococcus-faecium-EnGen0376_EOT95291.1

MKKNLLLVLPILGFAGFFVGVPMLSANIGISSYAAKKVIDIINTGSTVATIISIVAAVVGGGLITAGIVATAKSLIKKYGAKYAAAW

>Enterococcus-faecium-EnGen0377_EOT98340.1

MKKNLLLVLPILGFAGFFVGVPMLSANIGISSYAAKKVIDIINTGSTVATIISIVAAVVGGGLITAGIVATAKSLIKKYGAKYAAAW

>Enterococcus-faecium-BM4538_ERT29762.1

MKKNLLLVLPILGFAGFFVGVPMLSANIGISSYAAKKVIDIINTGSTVATIISIVAAVVGGGLITAGIVATAKSLIKKYGAKYAAAW

>Enterococcus-faecium-10/96A_ERT47319.1

MKKNLLLVLPILGFAGFFVGVPMLSANIGISSYAAKKVIDIINTGSTVATIISIVAAVVGGGLITAGIVATAKSLIKKYGAKYAAAW

>Enterococcus-faecium-VRE1044_EZP88528.1

MKKNLLLVLPILGFAGFFVGVPMLSANIGISSYAAKKVIDIINTGSTVATIISIVAAVVGGGLITAGIVATAKSLIKKYGAKYAAAW

>Enterococcus-faecium-VSE1036_EZP88666.1

MKKNLLLVLPILGFAGFFVGVPMLSANIGISSYAAKKVIDIINTGSTVATIISIVAAVVGGGLITAGIVATAKSLIKKYGAKYAAAW

>Enterococcus-faecium-VRE1261_EZP91337.1

MKKNLLLVLPILGFAGFFVGVPMLSANIGISSYAAKKVIDIINTGSTVATIISIVAAVVGGGLITAGIVATAKSLIKKYGAKYAAAW

>Enterococcus-faecium-VRE0576_EZP94164.1

MKKNLLLVLPILGFAGFFVGVPMLSANIGISSYAAKKVIDIINTGSTVATIISIVAAVVGGGLITAGIVATAKSLIKKYGAKYAAAW

>Enterococcus-faecium_KGK72643.1

MKKNLLLVLPILGFAGFFVGVPMLSANIGISSYAAKKVIDIINTGSTVATIISIVAAVVGGGLITAGIVATAKSLIKKYGAKYAAAW

>Enterococcus-faecium_KTK56728.1

MKKNLLLVLPILGFAGFFVGVPMLSANIGISSYAAKKVIDIINTGSTVATIISIVAAVVGGGLITAGIVATAKSLIKKYGAKYAAAW

>Enterococcus-faecium_KWZ03283.1

MKKNLLLVLPILGFAGFFVGVPMLSANIGISSYAAKKVIDIINTGSTVATIISIVAAVVGGGLITAGIVATAKSLIKKYGAKYAAAW

>Enterococcus-faecium_KWZ05402.1

MKKNLLLVLPILGFAGFFVGVPMLSANIGISSYAAKKVIDIINTGSTVATIISIVAAVVGGGLITAGIVATAKSLIKKYGAKYAAAW

>Enterococcus-faecium_KWZ05434.1

MKKNLLLVLPILGFAGFFVGVPMLSANIGISSYAAKKVIDIINTGSTVATIISIVAAVVGGGLITAGIVATAKSLIKKYGAKYAAAW

>Enterococcus-faecium_KWZ07472.1

MKKNLLLVLPILGFAGFFVGVPMLSANIGISSYAAKKVIDIINTGSTVATIISIVAAVVGGGLITAGIVATAKSLIKKYGAKYAAAW

>Enterococcus-faecium_KWZ12684.1

MKKNLLLVLPILGFAGFFVGVPMLSANIGISSYAAKKVIDIINTGSTVATIISIVAAVVGGGLITAGIVATAKSLIKKYGAKYAAAW

>Enterococcus-faecium_KXH55220.1

MKKNLLLVLPILGFAGFFVGVPMLSANIGISSYAAKKVIDIINTGSTVATIISIVAAVVGGGLITAGIVATAKSLIKKYGAKYAAAW

>Enterococcus-faecium-C68_KXW79851.1

MKKNLLLVLPILGFAGFFVGVPMLSANIGISSYAAKKVIDIINTGSTVATIISIVAAVVGGGLITAGIVATAKSLIKKYGAKYAAAW

>Enterococcus-faecium_AMP62324.1

MKKNLLLVLPILGFAGFFVGVPMLSANIGISSYAAKKVIDIINTGSTVATIISIVAAVVGGGLITAGIVATAKSLIKKYGAKYAAAW

>Enterococcus-faecium_ANB95181.1

MKKNLLLVLPILGFAGFFVGVPMLSANIGISSYAAKKVIDIINTGSTVATIISIVAAVVGGGLITAGIVATAKSLIKKYGAKYAAAW

>Enterococcus-faecium_SAM49815.1

MKKNLLLVLPILGFAGFFVGVPMLSANIGISSYAAKKVIDIINTGSTVATIISIVAAVVGGGLITAGIVATAKSLIKKYGAKYAAAW

>Enterococcus-faecium_SAM50037.1

MKKNLLLVLPILGFAGFFVGVPMLSANIGISSYAAKKVIDIINTGSTVATIISIVAAVVGGGLITAGIVATAKSLIKKYGAKYAAAW

>Enterococcus-faecium_SAM53392.1

MKKNLLLVLPILGFAGFFVGVPMLSANIGISSYAAKKVIDIINTGSTVATIISIVAAVVGGGLITAGIVATAKSLIKKYGAKYAAAW

>Enterococcus-faecium_SAM81524.1

MKKNLLLVLPILGFAGFFVGVPMLSANIGISSYAAKKVIDIINTGSTVATIISIVAAVVGGGLITAGIVATAKSLIKKYGAKYAAAW

>Enterococcus-faecium_CVH54173.1

MKKNLLLVLPILGFAGFFVGVPMLSANIGISSYAAKKVIDIINTGSTVATIISIVAAVVGGGLITAGIVATAKSLIKKYGAKYAAAW

>Enterococcus-faecium_AOM20504.1

MKKNLLLVLPILGFAGFFVGVPMLSANIGISSYAAKKVIDIINTGSTVATIISIVAAVVGGGLITAGIVATAKSLIKKYGAKYAAAW

>Enterococcus-faecium_AOM23642.1

MKKNLLLVLPILGFAGFFVGVPMLSANIGISSYAAKKVIDIINTGSTVATIISIVAAVVGGGLITAGIVATAKSLIKKYGAKYAAAW

>Enterococcus-faecium_AOM26799.1

MKKNLLLVLPILGFAGFFVGVPMLSANIGISSYAAKKVIDIINTGSTVATIISIVAAVVGGGLITAGIVATAKSLIKKYGAKYAAAW

>Enterococcus-faecium_AOM29643.1

MKKNLLLVLPILGFAGFFVGVPMLSANIGISSYAAKKVIDIINTGSTVATIISIVAAVVGGGLITAGIVATAKSLIKKYGAKYAAAW

>Enterococcus-faecium_AOM32662.1

MKKNLLLVLPILGFAGFFVGVPMLSANIGISSYAAKKVIDIINTGSTVATIISIVAAVVGGGLITAGIVATAKSLIKKYGAKYAAAW

>Enterococcus-faecium_AOM35814.1

MKKNLLLVLPILGFAGFFVGVPMLSANIGISSYAAKKVIDIINTGSTVATIISIVAAVVGGGLITAGIVATAKSLIKKYGAKYAAAW

>Enterococcus-faecium_AOM38817.1

MKKNLLLVLPILGFAGFFVGVPMLSANIGISSYAAKKVIDIINTGSTVATIISIVAAVVGGGLITAGIVATAKSLIKKYGAKYAAAW

>Enterococcus-faecium_AON61970.1

MKKNLLLVLPILGFAGFFVGVPMLSANIGISSYAAKKVIDIINTGSTVATIISIVAAVVGGGLITAGIVATAKSLIKKYGAKYAAAW

>Enterococcus-faecium_AOT80035.1

MKKNLLLVLPILGFAGFFVGVPMLSANIGISSYAAKKVIDIINTGSTVATIISIVAAVVGGGLITAGIVATAKSLIKKYGAKYAAAW

>Enterococcus-sp.-HMSC077E07_OFK26708.1

MKKNLLLVLPILGFAGFFVGVPMLSANIGISSYAAKKVIDIINTGSTVATIISIVAAVVGGGLITAGIVATAKSLIKKYGAKYAAAW

>Enterococcus-sp.-HMSC073E08_OFK44762.1

MKKNLLLVLPILGFAGFFVGVPMLSANIGISSYAAKKVIDIINTGSTVATIISIVAAVVGGGLITAGIVATAKSLIKKYGAKYAAAW

>Enterococcus-sp.-HMSC055G03_OFK59128.1

MKKNLLLVLPILGFAGFFVGVPMLSANIGISSYAAKKVIDIINTGSTVATIISIVAAVVGGGLITAGIVATAKSLIKKYGAKYAAAW

>Enterococcus-sp.-HMSC073E07_OFL71591.1

MKKNLLLVLPILGFAGFFVGVPMLSANIGISSYAAKKVIDIINTGSTVATIISIVAAVVGGGLITAGIVATAKSLIKKYGAKYAAAW

>Enterococcus-sp.-HMSC077E04_OFN26822.1

MKKNLLLVLPILGFAGFFVGVPMLSANIGISSYAAKKVIDIINTGSTVATIISIVAAVVGGGLITAGIVATAKSLIKKYGAKYAAAW

>Enterococcus-sp.-HMSC056C08_OFN45078.1

MKKNLLLVLPILGFAGFFVGVPMLSANIGISSYAAKKVIDIINTGSTVATIISIVAAVVGGGLITAGIVATAKSLIKKYGAKYAAAW

>Enterococcus-sp.-HMSC072G01_OFN47082.1

MKKNLLLVLPILGFAGFFVGVPMLSANIGISSYAAKKVIDIINTGSTVATIISIVAAVVGGGLITAGIVATAKSLIKKYGAKYAAAW

>Enterococcus-sp.-HMSC076D08_OFO90480.1

MKKNLLLVLPILGFAGFFVGVPMLSANIGISSYAAKKVIDIINTGSTVATIISIVAAVVGGGLITAGIVATAKSLIKKYGAKYAAAW

>Enterococcus-sp.-HMSC065H03_OFP08594.1

MKKNLLLVLPILGFAGFFVGVPMLSANIGISSYAAKKVIDIINTGSTVATIISIVAAVVGGGLITAGIVATAKSLIKKYGAKYAAAW

>Enterococcus-sp.-HMSC060E05_OFP79693.1

MKKNLLLVLPILGFAGFFVGVPMLSANIGISSYAAKKVIDIINTGSTVATIISIVAAVVGGGLITAGIVATAKSLIKKYGAKYAAAW

>Enterococcus-sp.-HMSC063C12_OFP95991.1

MKKNLLLVLPILGFAGFFVGVPMLSANIGISSYAAKKVIDIINTGSTVATIISIVAAVVGGGLITAGIVATAKSLIKKYGAKYAAAW

>Enterococcus-sp.-HMSC065H12_OFQ80485.1

MKKNLLLVLPILGFAGFFVGVPMLSANIGISSYAAKKVIDIINTGSTVATIISIVAAVVGGGLITAGIVATAKSLIKKYGAKYAAAW

>Enterococcus-sp.-HMSC072F07_OFR81257.1

MKKNLLLVLPILGFAGFFVGVPMLSANIGISSYAAKKVIDIINTGSTVATIISIVAAVVGGGLITAGIVATAKSLIKKYGAKYAAAW

>Enterococcus-sp.-HMSC034B11_OHO31804.1

MKKNLLLVLPILGFAGFFVGVPMLSANIGISSYAAKKVIDIINTGSTVATIISIVAAVVGGGLITAGIVATAKSLIKKYGAKYAAAW

>Enterococcus-sp.-HMSC035B04_OHO47319.1

MKKNLLLVLPILGFAGFFVGVPMLSANIGISSYAAKKVIDIINTGSTVATIISIVAAVVGGGLITAGIVATAKSLIKKYGAKYAAAW

>Enterococcus-sp.-HMSC060D09_OHP22727.1

MKKNLLLVLPILGFAGFFVGVPMLSANIGISSYAAKKVIDIINTGSTVATIISIVAAVVGGGLITAGIVATAKSLIKKYGAKYAAAW

>Enterococcus-sp.-HMSC060D07_OHP30496.1

MKKNLLLVLPILGFAGFFVGVPMLSANIGISSYAAKKVIDIINTGSTVATIISIVAAVVGGGLITAGIVATAKSLIKKYGAKYAAAW

>Enterococcus-sp.-HMSC070F12_OHQ62732.1

MKKNLLLVLPILGFAGFFVGVPMLSANIGISSYAAKKVIDIINTGSTVATIISIVAAVVGGGLITAGIVATAKSLIKKYGAKYAAAW

>Enterococcus-sp.-HMSC072D11_OHQ81110.1

MKKNLLLVLPILGFAGFFVGVPMLSANIGISSYAAKKVIDIINTGSTVATIISIVAAVVGGGLITAGIVATAKSLIKKYGAKYAAAW

>Enterococcus-sp.-HMSC074F07_OHQ92886.1

MKKNLLLVLPILGFAGFFVGVPMLSANIGISSYAAKKVIDIINTGSTVATIISIVAAVVGGGLITAGIVATAKSLIKKYGAKYAAAW

>Enterococcus-sp.-HMSC34G12_OHR66221.1

MKKNLLLVLPILGFAGFFVGVPMLSANIGISSYAAKKVIDIINTGSTVATIISIVAAVVGGGLITAGIVATAKSLIKKYGAKYAAAW

>Enterococcus-faecium_APJ08257.1

MKKNLLLVLPILGFAGFFVGVPMLSANIGISSYAAKKVIDIINTGSTVATIISIVAAVVGGGLITAGIVATAKSLIKKYGAKYAAAW

>Enterococcus-faecium_APV52734.1

MKKNLLLVLPILGFAGFFVGVPMLSANIGISSYAAKKVIDIINTGSTVATIISIVAAVVGGGLITAGIVATAKSLIKKYGAKYAAAW

>Enterococcus-faecium_APV58631.1

MKKNLLLVLPILGFAGFFVGVPMLSANIGISSYAAKKVIDIINTGSTVATIISIVAAVVGGGLITAGIVATAKSLIKKYGAKYAAAW

>Enterococcus-faecium_OLZ33788.1

MKKNLLLVLPILGFAGFFVGVPMLSANIGISSYAAKKVIDIINTGSTVATIISIVAAVVGGGLITAGIVATAKSLIKKYGAKYAAAW

>Enterococcus-faecium_ONN33400.1

MKKNLLLVLPILGFAGFFVGVPMLSANIGISSYAAKKVIDIINTGSTVATIISIVAAVVGGGLITAGIVATAKSLIKKYGAKYAAAW

>Enterococcus-faecium_OOL50843.1

MKKNLLLVLPILGFAGFFVGVPMLSANIGISSYAAKKVIDIINTGSTVATIISIVAAVVGGGLITAGIVATAKSLIKKYGAKYAAAW

>Enterococcus-faecium_OOL57576.1

MKKNLLLVLPILGFAGFFVGVPMLSANIGISSYAAKKVIDIINTGSTVATIISIVAAVVGGGLITAGIVATAKSLIKKYGAKYAAAW

>Enterococcus-faecium_OOL73348.1

MKKNLLLVLPILGFAGFFVGVPMLSANIGISSYAAKKVIDIINTGSTVATIISIVAAVVGGGLITAGIVATAKSLIKKYGAKYAAAW

>Enterococcus-faecium_OOL79562.1

MKKNLLLVLPILGFAGFFVGVPMLSANIGISSYAAKKVIDIINTGSTVATIISIVAAVVGGGLITAGIVATAKSLIKKYGAKYAAAW

>Enterococcus-faecium_OOP65524.1

MKKNLLLVLPILGFAGFFVGVPMLSANIGISSYAAKKVIDIINTGSTVATIISIVAAVVGGGLITAGIVATAKSLIKKYGAKYAAAW

>Enterococcus-faecium_OPH30480.1

MKKNLLLVLPILGFAGFFVGVPMLSANIGISSYAAKKVIDIINTGSTVATIISIVAAVVGGGLITAGIVATAKSLIKKYGAKYAAAW

>Enterococcus-faecium_OPH32717.1

MKKNLLLVLPILGFAGFFVGVPMLSANIGISSYAAKKVIDIINTGSTVATIISIVAAVVGGGLITAGIVATAKSLIKKYGAKYAAAW

>Enterococcus-faecium_OPH32854.1

MKKNLLLVLPILGFAGFFVGVPMLSANIGISSYAAKKVIDIINTGSTVATIISIVAAVVGGGLITAGIVATAKSLIKKYGAKYAAAW

>Enterococcus-faecium_OSP57643.1

MKKNLLLVLPILGFAGFFVGVPMLSANIGISSYAAKKVIDIINTGSTVATIISIVAAVVGGGLITAGIVATAKSLIKKYGAKYAAAW

>Enterococcus-faecium_OSP80186.1

MKKNLLLVLPILGFAGFFVGVPMLSANIGISSYAAKKVIDIINTGSTVATIISIVAAVVGGGLITAGIVATAKSLIKKYGAKYAAAW

>Enterococcus-faecium_OUK05821.1

MKKNLLLVLPILGFAGFFVGVPMLSANIGISSYAAKKVIDIINTGSTVATIISIVAAVVGGGLITAGIVATAKSLIKKYGAKYAAAW

>Enterococcus-faecium_OUK12895.1

MKKNLLLVLPILGFAGFFVGVPMLSANIGISSYAAKKVIDIINTGSTVATIISIVAAVVGGGLITAGIVATAKSLIKKYGAKYAAAW

>Enterococcus-faecium_OUK18059.1

MKKNLLLVLPILGFAGFFVGVPMLSANIGISSYAAKKVIDIINTGSTVATIISIVAAVVGGGLITAGIVATAKSLIKKYGAKYAAAW

>Enterococcus-faecium_OUK19108.1

MKKNLLLVLPILGFAGFFVGVPMLSANIGISSYAAKKVIDIINTGSTVATIISIVAAVVGGGLITAGIVATAKSLIKKYGAKYAAAW

>Enterococcus-faecium_OUK19133.1

MKKNLLLVLPILGFAGFFVGVPMLSANIGISSYAAKKVIDIINTGSTVATIISIVAAVVGGGLITAGIVATAKSLIKKYGAKYAAAW

>Enterococcus-faecium_OUK25775.1

MKKNLLLVLPILGFAGFFVGVPMLSANIGISSYAAKKVIDIINTGSTVATIISIVAAVVGGGLITAGIVATAKSLIKKYGAKYAAAW

>Enterococcus-faecium_OUK26544.1

MKKNLLLVLPILGFAGFFVGVPMLSANIGISSYAAKKVIDIINTGSTVATIISIVAAVVGGGLITAGIVATAKSLIKKYGAKYAAAW

>Enterococcus-faecium_OUK32071.1

MKKNLLLVLPILGFAGFFVGVPMLSANIGISSYAAKKVIDIINTGSTVATIISIVAAVVGGGLITAGIVATAKSLIKKYGAKYAAAW

>Enterococcus-faecium_OUK32079.1

MKKNLLLVLPILGFAGFFVGVPMLSANIGISSYAAKKVIDIINTGSTVATIISIVAAVVGGGLITAGIVATAKSLIKKYGAKYAAAW

>Enterococcus-faecium_OUK39040.1

MKKNLLLVLPILGFAGFFVGVPMLSANIGISSYAAKKVIDIINTGSTVATIISIVAAVVGGGLITAGIVATAKSLIKKYGAKYAAAW

>Enterococcus-faecium_OUK39058.1

MKKNLLLVLPILGFAGFFVGVPMLSANIGISSYAAKKVIDIINTGSTVATIISIVAAVVGGGLITAGIVATAKSLIKKYGAKYAAAW

>Enterococcus-faecium_OUZ19615.1

MKKNLLLVLPILGFAGFFVGVPMLSANIGISSYAAKKVIDIINTGSTVATIISIVAAVVGGGLITAGIVATAKSLIKKYGAKYAAAW

>Enterococcus-faecium_OWS32545.1

MKKNLLLVLPILGFAGFFVGVPMLSANIGISSYAAKKVIDIINTGSTVATIISIVAAVVGGGLITAGIVATAKSLIKKYGAKYAAAW

>Enterococcus-faecium_OZH61829.1

MKKNLLLVLPILGFAGFFVGVPMLSANIGISSYAAKKVIDIINTGSTVATIISIVAAVVGGGLITAGIVATAKSLIKKYGAKYAAAW

>Enterococcus-faecium_OZH68648.1

MKKNLLLVLPILGFAGFFVGVPMLSANIGISSYAAKKVIDIINTGSTVATIISIVAAVVGGGLITAGIVATAKSLIKKYGAKYAAAW

>Enterococcus-faecium_OZH71166.1

MKKNLLLVLPILGFAGFFVGVPMLSANIGISSYAAKKVIDIINTGSTVATIISIVAAVVGGGLITAGIVATAKSLIKKYGAKYAAAW

>Enterococcus-faecium_ATD79713.1

MKKNLLLVLPILGFAGFFVGVPMLSANIGISSYAAKKVIDIINTGSTVATIISIVAAVVGGGLITAGIVATAKSLIKKYGAKYAAAW

>Enterococcus-faecium_PCO40089.1

MKKNLLLVLPILGFAGFFVGVPMLSANIGISSYAAKKVIDIINTGSTVATIISIVAAVVGGGLITAGIVATAKSLIKKYGAKYAAAW

>Enterococcus-faecium_PCT04264.1

MKKNLLLVLPILGFAGFFVGVPMLSANIGISSYAAKKVIDIINTGSTVATIISIVAAVVGGGLITAGIVATAKSLIKKYGAKYAAAW

>Enterococcus-faecium_PCT07534.1

MKKNLLLVLPILGFAGFFVGVPMLSANIGISSYAAKKVIDIINTGSTVATIISIVAAVVGGGLITAGIVATAKSLIKKYGAKYAAAW

>Enterococcus-faecium_PCT42371.1

MKKNLLLVLPILGFAGFFVGVPMLSANIGISSYAAKKVIDIINTGSTVATIISIVAAVVGGGLITAGIVATAKSLIKKYGAKYAAAW

>Enterococcus-faecium_PHL67091.1

MKKNLLLVLPILGFAGFFVGVPMLSANIGISSYAAKKVIDIINTGSTVATIISIVAAVVGGGLITAGIVATAKSLIKKYGAKYAAAW

>Enterococcus-faecium_PHL75178.1

MKKNLLLVLPILGFAGFFVGVPMLSANIGISSYAAKKVIDIINTGSTVATIISIVAAVVGGGLITAGIVATAKSLIKKYGAKYAAAW

>Enterococcus-faecium_PHL77927.1

MKKNLLLVLPILGFAGFFVGVPMLSANIGISSYAAKKVIDIINTGSTVATIISIVAAVVGGGLITAGIVATAKSLIKKYGAKYAAAW

>Enterococcus-faecium_PHL80834.1

MKKNLLLVLPILGFAGFFVGVPMLSANIGISSYAAKKVIDIINTGSTVATIISIVAAVVGGGLITAGIVATAKSLIKKYGAKYAAAW

>Enterococcus-faecium_PHL89871.1

MKKNLLLVLPILGFAGFFVGVPMLSANIGISSYAAKKVIDIINTGSTVATIISIVAAVVGGGLITAGIVATAKSLIKKYGAKYAAAW

>Enterococcus-faecium_ATU01610.1

MKKNLLLVLPILGFAGFFVGVPMLSANIGISSYAAKKVIDIINTGSTVATIISIVAAVVGGGLITAGIVATAKSLIKKYGAKYAAAW

>Enterococcus-faecium_ATU04560.1

MKKNLLLVLPILGFAGFFVGVPMLSANIGISSYAAKKVIDIINTGSTVATIISIVAAVVGGGLITAGIVATAKSLIKKYGAKYAAAW

>Enterococcus-faecium_ATW37583.1

MKKNLLLVLPILGFAGFFVGVPMLSANIGISSYAAKKVIDIINTGSTVATIISIVAAVVGGGLITAGIVATAKSLIKKYGAKYAAAW

>Enterococcus-faecium_KWY42088.1

MKKNLLLVLPILGFAGFFVGVPMLSANIGISSYAAKKVIDIINTGSTVATIISIVAAVVGGGLITAGIVATAKSLIKKYGAKYAAAW

>Enterococcus-faecium_KWY45279.1

MKKNLLLVLPILGFAGFFVGVPMLSANIGISSYAAKKVIDIINTGSTVATIISIVAAVVGGGLITAGIVATAKSLIKKYGAKYAAAW

>Enterococcus-faecium_KWY47068.1

MKKNLLLVLPILGFAGFFVGVPMLSANIGISSYAAKKVIDIINTGSTVATIISIVAAVVGGGLITAGIVATAKSLIKKYGAKYAAAW

>Enterococcus-faecium_KWY49763.1

MKKNLLLVLPILGFAGFFVGVPMLSANIGISSYAAKKVIDIINTGSTVATIISIVAAVVGGGLITAGIVATAKSLIKKYGAKYAAAW

>Enterococcus-faecium_KWY54194.1

MKKNLLLVLPILGFAGFFVGVPMLSANIGISSYAAKKVIDIINTGSTVATIISIVAAVVGGGLITAGIVATAKSLIKKYGAKYAAAW

>Enterococcus-faecium_KWY54689.1

MKKNLLLVLPILGFAGFFVGVPMLSANIGISSYAAKKVIDIINTGSTVATIISIVAAVVGGGLITAGIVATAKSLIKKYGAKYAAAW

>Enterococcus-faecium_KWY60643.1

MKKNLLLVLPILGFAGFFVGVPMLSANIGISSYAAKKVIDIINTGSTVATIISIVAAVVGGGLITAGIVATAKSLIKKYGAKYAAAW

>Enterococcus-faecium_KWY63207.1

MKKNLLLVLPILGFAGFFVGVPMLSANIGISSYAAKKVIDIINTGSTVATIISIVAAVVGGGLITAGIVATAKSLIKKYGAKYAAAW

>Enterococcus-faecium_KWY70716.1

MKKNLLLVLPILGFAGFFVGVPMLSANIGISSYAAKKVIDIINTGSTVATIISIVAAVVGGGLITAGIVATAKSLIKKYGAKYAAAW

>Enterococcus-faecium_KWY70724.1

MKKNLLLVLPILGFAGFFVGVPMLSANIGISSYAAKKVIDIINTGSTVATIISIVAAVVGGGLITAGIVATAKSLIKKYGAKYAAAW

>Enterococcus-faecium_KWY77911.1

MKKNLLLVLPILGFAGFFVGVPMLSANIGISSYAAKKVIDIINTGSTVATIISIVAAVVGGGLITAGIVATAKSLIKKYGAKYAAAW

>Enterococcus-faecium_KWY79446.1

MKKNLLLVLPILGFAGFFVGVPMLSANIGISSYAAKKVIDIINTGSTVATIISIVAAVVGGGLITAGIVATAKSLIKKYGAKYAAAW

>Enterococcus-faecium_KWY89303.1

MKKNLLLVLPILGFAGFFVGVPMLSANIGISSYAAKKVIDIINTGSTVATIISIVAAVVGGGLITAGIVATAKSLIKKYGAKYAAAW

>Enterococcus-faecium_KWY90731.1

MKKNLLLVLPILGFAGFFVGVPMLSANIGISSYAAKKVIDIINTGSTVATIISIVAAVVGGGLITAGIVATAKSLIKKYGAKYAAAW

>Enterococcus-faecium_KWY91345.1

MKKNLLLVLPILGFAGFFVGVPMLSANIGISSYAAKKVIDIINTGSTVATIISIVAAVVGGGLITAGIVATAKSLIKKYGAKYAAAW

>Enterococcus-faecium_KWY92126.1

MKKNLLLVLPILGFAGFFVGVPMLSANIGISSYAAKKVIDIINTGSTVATIISIVAAVVGGGLITAGIVATAKSLIKKYGAKYAAAW

>Enterococcus-faecium_PJN96895.1

MKKNLLLVLPILGFAGFFVGVPMLSANIGISSYAAKKVIDIINTGSTVATIISIVAAVVGGGLITAGIVATAKSLIKKYGAKYAAAW

>Enterococcus-faecium_AUI17123.1

MKKNLLLVLPILGFAGFFVGVPMLSANIGISSYAAKKVIDIINTGSTVATIISIVAAVVGGGLITAGIVATAKSLIKKYGAKYAAAW

>Enterococcus-faecium_AUI20050.1

MKKNLLLVLPILGFAGFFVGVPMLSANIGISSYAAKKVIDIINTGSTVATIISIVAAVVGGGLITAGIVATAKSLIKKYGAKYAAAW

>Enterococcus-faecium_AUI23019.1

MKKNLLLVLPILGFAGFFVGVPMLSANIGISSYAAKKVIDIINTGSTVATIISIVAAVVGGGLITAGIVATAKSLIKKYGAKYAAAW

>Enterococcus-faecium_AUI25993.1

MKKNLLLVLPILGFAGFFVGVPMLSANIGISSYAAKKVIDIINTGSTVATIISIVAAVVGGGLITAGIVATAKSLIKKYGAKYAAAW

>Enterococcus-faecium_AUI28967.1

MKKNLLLVLPILGFAGFFVGVPMLSANIGISSYAAKKVIDIINTGSTVATIISIVAAVVGGGLITAGIVATAKSLIKKYGAKYAAAW

>Enterococcus-faecium_AUI31944.1

MKKNLLLVLPILGFAGFFVGVPMLSANIGISSYAAKKVIDIINTGSTVATIISIVAAVVGGGLITAGIVATAKSLIKKYGAKYAAAW

>Enterococcus-faecium_AUI34873.1

MKKNLLLVLPILGFAGFFVGVPMLSANIGISSYAAKKVIDIINTGSTVATIISIVAAVVGGGLITAGIVATAKSLIKKYGAKYAAAW

>Enterococcus-faecium_AUI35069.1

MKKNLLLVLPILGFAGFFVGVPMLSANIGISSYAAKKVIDIINTGSTVATIISIVAAVVGGGLITAGIVATAKSLIKKYGAKYAAAW

>Enterococcus-faecium_AUO63196.1

MKKNLLLVLPILGFAGFFVGVPMLSANIGISSYAAKKVIDIINTGSTVATIISIVAAVVGGGLITAGIVATAKSLIKKYGAKYAAAW

>Enterococcus-faecium_PNE94076.1

MKKNLLLVLPILGFAGFFVGVPMLSANIGISSYAAKKVIDIINTGSTVATIISIVAAVVGGGLITAGIVATAKSLIKKYGAKYAAAW

>Enterococcus-faecium_PNE97427.1

MKKNLLLVLPILGFAGFFVGVPMLSANIGISSYAAKKVIDIINTGSTVATIISIVAAVVGGGLITAGIVATAKSLIKKYGAKYAAAW

>Enterococcus-faecium_PNL24919.1

MKKNLLLVLPILGFAGFFVGVPMLSANIGISSYAAKKVIDIINTGSTVATIISIVAAVVGGGLITAGIVATAKSLIKKYGAKYAAAW

>Enterococcus-faecium_PNN19998.1

MKKNLLLVLPILGFAGFFVGVPMLSANIGISSYAAKKVIDIINTGSTVATIISIVAAVVGGGLITAGIVATAKSLIKKYGAKYAAAW

>Enterococcus faecalis EEU67922.1

MKLDSSLFSKKMYMGVVFVMVVCVINLTQPHLTSTLGISTYAAKKVIDIISAAGSVWSVVGIVAAVVGGGGIGAAILVTAKSFVKRYGKAFATAW

>Enterococcus faecalis EFE17716.1

MKLDSSLFSKKMYMGVVFVMVVCVINLTQPHLTSTLGISTYAAKKVIDIISAAGSVWSVVGIVAAVVGGGGIGAAILVTAKSFVKRYGKAFATAW

>Enterococcus faecalis EFE20480.1

MKLDSSLFSKKMYMGVVFVMVVCVINLTQPHLTSTLGISTYAAKKVIDIISAAGSVWSVVGIVAAVVGGGGIGAAILVTAKSFVKRYGKAFATAW

>Enterococcus faecalis EFQ08505.1

MKLDSSLFSKKMYMGVVFVMVVCVINLTQPHLTSTLGISTYAAKKVIDIISAAGSVWSVVGIVAAVVGGGGIGAAILVTAKSFVKRYGKAFATAW

>Enterococcus faecalis EFQ66565.1

MKLDSSLFSKKMYMGVVFVMVVCVINLTQPHLTSTLGISTYAAKKVIDIISAAGSVWSVVGIVAAVVGGGGIGAAILVTAKSFVKRYGKAFATAW

>Enterococcus faecalis ETJ10753.1

MKLDSSLFSKKMYMGVVFVMVVCVINLTQPHLTSTLGISTYAAKKVIDIISAAGSVWSVVGIVAAVVGGGGIGAAILVTAKSFVKRYGKAFATAW

>Enterococcus faecalis OAD12169.1

MKLDSSLFSKKMYMGVVFVMVVCVINLTQPHLTSTLGISTYAAKKVIDIISAAGSVWSVVGIVAAVVGGGGIGAAILVTAKSFVKRYGKAFATAW

>Enterococcus faecalis EOD96444.1

MKLNNVLFNKKMYMGVAFVLVAFAISLTQPHLTSTLGISAYAAKKVIDIISAASSVAAVVGIIAAVVGGGGIGVAVLATAKALVKKYGKAYAAAW

>Enterococcus faecalis EOI48691.1

MKLNNVLFNKKMYMGVAFVLVAFAISLTQPHLTSTLGISAYAAKKVIDIISAASSVAAVVGIIAAVVGGGGIGVAVLATAKALVKKYGKAYAAAW

>Enterococcus faecalis EOJ98348.1

MKLNNVLFNKKMYMGVAFVLVAFAISLTQPHLTSTLGISAYAAKKVIDIISAASSVAAVVGIIAAVVGGGGIGVAVLATAKALVKKYGKAYAAAW

>Enterococcus faecalis EOK58536.1

MKLNNVLFNKKMYMGVAFVLVAFAISLTQPHLTSTLGISAYAAKKVIDIISAASSVAAVVGIIAAVVGGGGIGVAVLATAKALVKKYGKAYAAAW

>Enterococcus faecalis KAJ75337.1

MKLNNVLFNKKMYMGVAFVLVAFAISLTQPHLTSTLGISAYAAKKVIDIISAASSVAAVVGIIAAVVGGGGIGVAVLATAKALVKKYGKAYAAAW

>Staphylococcus aureus ELY17223.1

METLVKRRNTLIFSLLVTISIASLLFLTLTTPELTSTLGISSYAAKKAIDIIAAAGDVAAIVGLIGAVTGAGAIGAGILFTAKKLIKSYGKKYAAAW

>Staphylococcus aureus OAP75241.1

METLVKRRNTLIFSLLVTISIASLLFLTLTTPELTSTLGISSYAAKKAIDIIAAAGDVAAIVGLIGAVTGAGAIGAGILFTAKKLIKSYGKKYAAAW

>Staphylococcus aureus SGW13862.1

METLVKRRNTLIFSLLVTISIASLLFLTLTTPELTSTLGISSYAAKKAIDIIAAAGDVAAIVGLIGAVTGAGAIGAGILFTAKKLIKSYGKKYAAAW

>Staphylococcus aureus SGW00627.1

METLVKRRNTLIFSLLVTISIASLLFLTLTTPELTSTLGISSYAAKKAIDIIAAAGDVAAIVGLIGAVTGAGAIGAGILFTAKKLIKSYGKKYAAAW

>Staphylococcus aureus SHD23261.1

METLVKRRNTLIFSLLVTISIASLLFLTLTTPELTSTLGISSYAAKKAIDIIAAAGDVAAIVGLIGAVTGAGAIGAGILFTAKKLIKSYGKKYAAAW

>Streptococcus pneumoniae COC74999.1

MMNLVKSNKKSFILFGAALAAATLVYALLLTGTELNVAAAHAFSANAELASTLGISTAAAKKAIDIIDAASTIASIISLIGIVTGAGAISYAIVATAKTMIKKYGKKYAAAW

>Mycobacterium abscessus SLC22340.1

MMNLVKSNKKSFILFGAALAAATLVYALLLTGTELNVAAAHAFSANAELASTLGISTAAAKKAIDIIDAASTIASIISLIGIVTGAGAISYAIVATAKTMIKKYGKKYAAAW

>Paenibacillus larvae ETK29617.1

MNLVKSNKKVIVLLGLGLVSTSLLYMFLLTGTNLGVTPVQGFSGNAELASTLGISTAAAKKAIDIIDAASTIASIISLIGIVTGAGAVSYAIVATAKTMIKKYGKKYAAAW

>Paenibacillus larvae PCK70112.1

MNLVKSNKKVIVLLGLGLVSTSLLYMFLLTGTNLGVTPVQGFSGNAELASTLGISTAAAKKAIDIIDAASTIASIISLIGIVTGAGAVSYAIVATAKTMIKKYGKKYAAAW

>Paenibacillus larvae AVF23715.1

MNLVKSNKKVIVLLGLGLVSTSLLYMFLLTGTNLGVTPVQGFSGNAELASTLGISTAAAKKAIDIIDAASTIASIISLIGIVTGAGAVSYAIVATAKTMIKKYGKKYAAAW

>Paenibacillus larvae AVG13937.1

MNLVKSNKKVIVLLGLGLVSTSLLYMFLLTGTNLGVTPVQGFSGNAELASTLGISTAAAKKAIDIIDAASTIASIISLIGIVTGAGAVSYAIVATAKTMIKKYGKKYAAAW

>Paenibacillus larvae ARF68818.1

MNLVKSNKKVIVLLGLGLVSASLLYMFLLTGTNLGVTPVQGFSGNAELASTLGISTAAAKKAIDIIDAASTIASIISLIGIVTGAGAVSYAIVATAKTMIKKYGKKYAAAW

>Paenibacillus larvae AQZ47516.1

MNLVKSNKKVIVLLGLGLMSASLLYMFLLTGTNLGVTPVQGFSGNAELASTLGISTAAAKKAIDIIDAASTIASIISLIGIVTGAGAVSYAIVATAKTMIKKYGKKYAAAW

>Paenibacillus larvae AVF24873.1

MNLVKSNKKVIVLLGLGLMSASLLYMFLLTGTNLGVTPVQGFSGNAELASTLGISTAAAKKAIDIIDAASTIASIISLIGIVTGAGAVSYAIVATAKTMIKKYGKKYAAAW

>Paenibacillus larvae AVF29633.1

MNLVKSNKKVIVLLGLGLMSASLLYMFLLTGTNLGVTPVQGFSGNAELASTLGISTAAAKKAIDIIDAASTIASIISLIGIVTGAGAVSYAIVATAKTMIKKYGKKYAAAW

>Paenibacillus larvae AQR79158.1

MNLVKSNKKVIVLLGLGLVSTSLLYMFLLTGTNLGVTPVQGFSGNAELASTLGISTAAAKKAIDIIDAASTIASIISLIGIVTGAGAVSYAIVATAKTMIKKYGKKYAAAW

>Paenibacillus larvae AHD07374.1

MVKSNKKVIVLLGLGLVSTSLLYMFLLTGTNLGVTPVQGFSGNAELASTLGISTAAAKKAIDIIDAASTIASIISLIGIVTGAGAVSYAIVATAKTMIKKYGKKYAAAW

>Paenibacillus larvae AQT86857.1

MSASLLYMFLLTGTNLGVTPVQGFSGNAELASTLGISTAAAKKAIDIIDAASTIASIISLIGIVTGAGAVSYAIVATAKTMIKKYGKKYAAAW

>Bacillus coagulans AEP01267.1

MVNSLTNKKRVFLFVVIGLVLATLSSVAYISTLQITIHQTAVLPGNAYLASTLGISTAAAKKAIDIIDTASTIASIISLIGVVTGAGAISYAVVATAKAMIKKYGKKYAAAW

>Bacillus coagulans KGB29332.1

MVNSLSNKKRVFLFVVIGLVLATLSSVAYISTLQITIHQTAVLPGNAYLASTLGISTAAAKKAIDIIDTASTIASIISLIGVVTGAGAISYAVVATAKAMIKKYGKKYAAAW

>Bacillus coagulans KXT21788.1

MVNSLSNKKRVFLFVVIGLVLATLSSVAYISTLQITIHQTAVLPGNAYLASTLGISTAAAKKAIDIIDTASTIASIISLIGVVTGAGAISYAVVATAKAMIKKYGKKYAAAW

>Bacillus coagulans APB37089.1

MVNSLSNKKRVFLFVVIGLVLATLSSVAYISTLQITIHQTAVLPGNAYLASTLGISTAAAKKAIDIIDTASTIASIISLIGVVTGAGAISYAVVATAKAMIKKYGKKYAAAW

>Bacillus coagulans OZV96169.1

MVNSLSNKKRVFLFVVIGLVLATLSSVAYISTLQITIHQTAVLPGNAYLASTLGISTAAAKKAIDIIDTASTIASIISLIGVVTGAGAISYAVVATAKAMIKKYGKKYAAAW

>Bacillus coagulans ATW82219.1

MVNSLSNKKRVFLFVVIGLVLATLSSVAYISTLQITIHQTAVLPGNAYLASTLGISTAAAKKAIDIIDTASTIASIISLIGVVTGAGAISYAVVATAKAMIKKYGKKYAAAW

>Bacillus coagulans AVD57117.1

MVNSLSNKKRVFLFVVIGLVLATLSSVAYISTLQITIHQTAVLPGNAYLASTLGISTAAAKKAIDIIDTASTIASIISLIGVVTGAGAISYAVVATAKAMIKKYGKKYAAAW

>Bacillus coagulans KGT37609.1

MVNSLSNKKRVFLFVVIGLVLATLSSVAYISTLQITIHQTAVLPGNAYLASTLGISTAAAKKAIDIIDTASTIASIISLIGVVTGAGAISYAVVATAKAMIKKYGKKYAAAW

>Bacillus coagulans KWZ79712.1

MVNSLSNKKRVFLFVVIGLVLATLSSVAYISTLQITIHQTAVLPGNAYLASTLGISTAAAKKAIDIIDTASTIASIISLIGVVTGAGAISYAVVATAKAMIKKYGKKYAAAW

>Bacillus coagulans KYC65188.1

MVNSLSNKKRVFLFVVIGLVLATLSSVAYISTLQITIHQTAVLPGNAYLASTLGISTAAAKKAIDIIDTASTIASIISLIGVVTGAGAISYAVVATAKAMIKKYGKKYAAAW

>Gracilibacillus boraciitolerans GAE92418.1

MVNNLSNKYVFMIAIIGLAFATLSSIAFISTLQISIGDTALLAGNSHLASTLGISAYAAKKAVDIIDAASAVVSIISLIGIVTGGAGAISYAIVATAKYMIRNYGKKYAAAW

>Bacillus pumilus AHL73410.1

MKNSNLFKIASVTSIVSLFAIATFLMGSSEGIASLASIDFNSGNQFALDLATNLGISRKTAYAAIGVIMTTGDVLTILSLLAVVLGGTGLVTAAMVATAKKLATKHGKKYAAEW

>Bacillus pumilus OBS83806.1

MKNSNLFKIASVTSIVSLFAIATFLMGSSEGIASLASIDFNSGNQFALDLATNLGISRKTAYAAIGVIMTTGDVLTILSLLAVVLGGTGLVTAAMVATAKKLATKHGKKYAAEW

>Bacillus altitudinis OJT66332.1

MKNSNLFKIASVTSIVSLFAIATFLMGSSEGIASLASIDFNSGNQFALDLATNLGISRKTAYAAIGVIMTTGDVLTILSLLAVVLGGTGLVTAAMVATAKKLATKHGKKYAAEW

>Bacillus altitudinis OPW96241.1

MKNSNLFKIASVTSIVSLFAIATFLMGSSEGIASLASIDFNSGNQFALDLATNLGISRKTAYAAIGVIMTTGDVLTILSLLAVVLGGTGLVTAAMVATAKKLATKHGKKYAAEW

>Bacillus altitudinis PGD46397.1

MKNSNLFKIASVTSIVSLFAIATFLMGSSEGIASLASIDFNSGNQFALDLATNLGISRKTAYAAIGVIMTTGDVLTILSLLAVVLGGTGLVTAAMVATAKKLATKHGKKYAAEW

>Bacillus stratosphericus OQP18696.1

MKNSNLFKIASVTSIVSLFAIATFLMGSSEGIASLASIDFNSGNQFALDLATNLGISRKTAYAAIGVIMTTGDVLTILSLLAVVLGGTGLVTAAMVATAKKLATKHGKKYAAEW

>Bacillus safensis OUK96109.1

MKNSNLFKIASVTSIVSLFAIATFLMGSSEGIASLASIDFNSGNQFALDLATNLGISRKTAYAAIGVIMTTGDVLTILSLLAVVLGGTGLVTAAMVATAKKLATKHGKKYAAEW

>Bacillus safensis OLP67033.1

MKNSNLFKIASVTSVVSLFAIATFLMGSTDGIASLASIDFSSGSQFALDLSTNLGISKKTAYAAIAVIMTTGDVLTILSLLAVVLGGTGLITAAMVATAKKLAKKHGKKYAAEW

>Bacillus atrophaeus ARW06838.1

MKNSNLFKIVSVTSVVSLFAIATFLMGSSEGIAGLASIDFTSGNQFALDLSTNLGISRKTAYVAIGVIMTTGDILTILSLLAVVLGGTGLITAAMVATAKKLAKKYGKKYAAEW

>Bacillus atrophaeus ASS71217.1

MKNSNLFKIVSVTSVVSFFAIATFLMGSSEGIAGLASIDFSSGNQFALDLSTNLGISRKTAYVAIGVIMTTGDILTILSLLAVVLGGTGLITAAMVATAKKLAKKYGKKYAAEW

>Bacillus paralicheniformis AGN37105.1

MKNSNLFKIASITGVVSLFAIATFLMGSSEGIAGLASIDFSSGHQFALDLATNLGISKKTAYVAIGVIMTTGDVLTILSLLAVVLGGTGLITAAMVATAKKLAKKYGKKYAAEW

>Bacillus paralicheniformis KFM90639.1

MKNSNLFKIASITGVVSLFAIATFLMGSSEGIAGLASIDFSSGHQFALDLATNLGISKKTAYVAIGVIMTTGDVLTILSLLAVVLGGTGLITAAMVATAKKLAKKYGKKYAAEW

>Bacillus paralicheniformis AJO19047.1

MKNSNLFKIASITGVVSLFAIATFLMGSSEGIAGLASIDFSSGHQFALDLATNLGISKKTAYVAIGVIMTTGDVLTILSLLAVVLGGTGLITAAMVATAKKLAKKYGKKYAAEW

>Bacillus paralicheniformis OPF76179.1

MKNSNLFKIASITGVVSLFAIATFLMGSSEGIAGLASIDFSSGHQFALDLATNLGISKKTAYVAIGVIMTTGDVLTILSLLAVVLGGTGLITAAMVATAKKLAKKYGKKYAAEW

>Bacillus paralicheniformis PAC98098.1

MKNSNLFKIASITGVVSLFAIATFLMGSSEGIAGLASIDFSSGHQFALDLATNLGISKKTAYVAIGVIMTTGDVLTILSLLAVVLGGTGLITAAMVATAKKLAKKYGKKYAAEW

>Bacillus sp. MBGLi97 POO80987.1

MKNSNLFKIASITGVVSLFAIATFLMGSSEGIAGLASIDFSSGHQFALDLATNLGISKKTAYVAIGVIMTTGDVLTILSLLAVVLGGTGLITAAMVATAKKLAKKYGKKYAAEW

>Bacillus sp. FJAT-21955 KQL43090.1

MASLASIDFNSGNQFALDLATNLGISRKTAYAAIGVIMTTGDVLTILSLLAVVLGGTGLVTAAMVATAKKLATKHGKKYAAEW

>Bacillus swezeyi OMI31061.1

MDFSSGHQFALDLSTNLGISRKTAYVAIGVIMTTGDVLTILSLLAVVLGGTGLITAAMVATAKKLAKKYGKKYAAEW

>Bacillus paralicheniformis OCI07845.1

MDFSSGHQFALDLATNLGISKKTAYVAIGVIMTTGDVLTILSLLAVVLGGTGLITAAMVATAKKLAKKYGKKYAAEW

>Bacillus paralicheniformis OLQ52999.1

MDFSSGHQFALDLATNLGISKKTAYVAIGVIMTTGDVLTILSLLAVVLGGTGLITAAMVATAKKLAKKYGKKYAAEW

>Bacillus paralicheniformis PLC18202.1

MDFSSGHQFALDLATNLGISKKTAYVAIGVIMTTGDVLTILSLLAVVLGGTGLITAAMVATAKKLAKKYGKKYAAEW

>Bacillus swezeyi OMI31061.1

MDFSSGHQFALDLSTNLGISRKTAYVAIGVIMTTGDVLTILSLLAVVLGGTGLITAAMVATAKKLAKKYGKKYAAEW

>Bacillus licheniformis EWH20352.1

MKNSNLFKIASITGVVSLFAIATFLMGSSEGIAGLASIDFSSGHQFALDLATNLGISKKTAYVAIGVIMTTGDVLTILSLLAVVLGGTGLITAAMVATAKKLAKKYGKKYAAEW

>Bacillus licheniformis KUL13295.1

MKNSNLFKIASITGVVSLFAIATFLMGSSEGIAGLASIDFSSGHQFALDLATNLGISKKTAYVAIGVIMTTGDVLTILSLLAVVLGGTGLITAAMVATAKKLAKKYGKKYAAEW

>Bacillus licheniformis KUL17175.1

MKNSNLFKIASITGVVSLFAIATFLMGSSEGIAGLASIDFSSGHQFALDLATNLGISKKTAYVAIGVIMTTGDVLTILSLLAVVLGGTGLITAAMVATAKKLAKKYGKKYAAEW

>Bacillus sp. CPSM8 ETB72735.1

MKNSNLFKIASITGVVSLFAIATFLMGSSEGIAGLASIDFSSGHQFALDLATNLGISKKTAYVAIGVIMTTGDVLTILSLLAVVLGGTGLITAAMVATAKKLAKKYGKKYAAEW

>Streptococcus pseudopneumoniae AEL09589.1

MKSKRMEFHNKFLIVSAMLAVISWLSLGVVSFPMLAGTLGISTKAAATVVNLISAYSTVTAVISIVGAITGVGSIGSGIAATVLYILKKKSAAQAALW

>Streptococcus pseudopneumoniae EID70106.1

MKSKRMEFHNKFLIVSAMLAVISWLSLGVVSFPMLAGTLGISTKAAATVVNLISAYSTVTAVISIVGAITGVGSIGSGIAATVLYILKKKSAAQAALW

>Streptococcus pseudopneumoniae OOR84026.1

MKSKRMEFHNKFLIVSAMLAVISWLSLGVVSFPMLAGTLGISTKAAATVVNLISAYSTVTAVISIVGAITGVGSIGSGIAATVLYILKKKSAAQAALW

>Streptococcus pneumoniae SND42044.1

MKSKRMEFHNKFLIVSAMLAVISWLSLGVVSFPMLAGTLGISTKAAATVVNLISAYSTVTAVISIVGAITGVGSIGSGIAATVLYILKKKGAAKAALWLELDENTF

>Streptococcus pneumoniae-SP14-BS69_EDK64586.1

MKSKRMEFHNKFLIVSAMLAVISWLSLGVVSFPMLAGTLGISTKAAATVVNLISAYSTVTAVISIVGAITGVGSIGSGIAATVLYILKKKGAAKAALW

>Streptococcus-pneumoniae-SP6-BS73_EDK77301.1

MKSKRMEFHNKFLIVSAMLAVISWLSLGVVSFPMLAGTLGISTKAAATVVNLISAYSTVTAVISIVGAITGVGSIGSGIAATVLYILKKKGAAKAALW

>Streptococcus-pneumoniae-SP9-BS68_EDK77829.1

MKSKRMEFHNKFLIVSAMLAVISWLSLGVVSFPMLAGTLGISTKAAATVVNLISAYSTVTAVISIVGAITGVGSIGSGIAATVLYILKKKGAAKAALW

>Streptococcus-pneumoniae-SP23-BS72_EDK80341.1

MKSKRMEFHNKFLIVSAMLAVISWLSLGVVSFPMLAGTLGISTKAAATVVNLISAYSTVTAVISIVGAITGVGSIGSGIAATVLYILKKKGAAKAALW

>Streptococcus-pneumoniae-Hungary19A-6_ACA37165.1

MKSKRMEFHNKFLIVSAMLAVISWLSLGVVSFPMLAGTLGISTKAAATVVNLISAYSTVTAVISIVGAITGVGSIGSGIAATVLYILKKKGAAKAALW

>Streptococcus-pneumoniae-SP195_EDT92652.1

MKSKRMEFHNKFLIVSAMLAVISWLSLGVVSFPMLAGTLGISTKAAATVVNLISAYSTVTAVISIVGAITGVGSIGSGIAATVLYILKKKGAAKAALW

>Streptococcus-pneumoniae-CDC3059-06_EDT96606.1

MKSKRMEFHNKFLIVSAMLAVISWLSLGVVSFPMLAGTLGISTKAAATVVNLISAYSTVTAVISIVGAITGVGSIGSGIAATVLYILKKKGAAKAALW

>Streptococcus-pneumoniae-70585_ACO16313.1

MKSKRMEFHNKFLIVSAMLAVISWLSLGVVSFPMLAGTLGISTKAAATVVNLISAYSTVTAVISIVGAITGVGSIGSGIAATVLYILKKKGAAKAALW

>Streptococcus-pneumoniae-JJA_ACO19460.1

MKSKRMEFHNKFLIVSAMLAVISWLSLGVVSFPMLAGTLGISTKAAATVVNLISAYSTVTAVISIVGAITGVGSIGSGIAATVLYILKKKGAAKAALW

>Streptococcus-pneumoniae-GA17570_EGI86168.1

MKSKRMEFHNKFLIVSAMLAVISWLSLGVVSFPMLAGTLGISTKAAATVVNLISAYSTVTAVISIVGAITGVGSIGSGIAATVLYILKKKGAAKAALW

>Streptococcus-pneumoniae-GA47502_EHD31825.1

MKSKRMEFHNKFLIVSAMLAVISWLSLGVVSFPMLAGTLGISTKAAATVVNLISAYSTVTAVISIVGAITGVGSIGSGIAATVLYILKKKGAAKAALW

>Streptococcus-pneumoniae-GA11184_EHD33102.1

MKSKRMEFHNKFLIVSAMLAVISWLSLGVVSFPMLAGTLGISTKAAATVVNLISAYSTVTAVISIVGAITGVGSIGSGIAATVLYILKKKGAAKAALW

>Streptococcus-pneumoniae-6735-05_EHD34690.1

MKSKRMEFHNKFLIVSAMLAVISWLSLGVVSFPMLAGTLGISTKAAATVVNLISAYSTVTAVISIVGAITGVGSIGSGIAATVLYILKKKGAAKAALW

>Streptococcus-pneumoniae-GA47033_EHD40900.1

MKSKRMEFHNKFLIVSAMLAVISWLSLGVVSFPMLAGTLGISTKAAATVVNLISAYSTVTAVISIVGAITGVGSIGSGIAATVLYILKKKGAAKAALW

>Streptococcus-pneumoniae-GA43265_EHD44754.1

MKSKRMEFHNKFLIVSAMLAVISWLSLGVVSFPMLAGTLGISTKAAATVVNLISAYSTVTAVISIVGAITGVGSIGSGIAATVLYILKKKGAAKAALW

>Streptococcus-pneumoniae-6901-05_EHD52136.1

MKSKRMEFHNKFLIVSAMLAVISWLSLGVVSFPMLAGTLGISTKAAATVVNLISAYSTVTAVISIVGAITGVGSIGSGIAATVLYILKKKGAAKAALW

>Streptococcus-pneumoniae-NP070_EHD57939.1

MKSKRMEFHNKFLIVSAMLAVISWLSLGVVSFPMLAGTLGISTKAAATVVNLISAYSTVTAVISIVGAITGVGSIGSGIAATVLYILKKKGAAKAALW

>Streptococcus-pneumoniae-GA44500_EHD60559.1

MKSKRMEFHNKFLIVSAMLAVISWLSLGVVSFPMLAGTLGISTKAAATVVNLISAYSTVTAVISIVGAITGVGSIGSGIAATVLYILKKKGAAKAALW

>Streptococcus-pneumoniae-5787-06_EHD68714.1

MKSKRMEFHNKFLIVSAMLAVISWLSLGVVSFPMLAGTLGISTKAAATVVNLISAYSTVTAVISIVGAITGVGSIGSGIAATVLYILKKKGAAKAALW

>Streptococcus-pneumoniae-6963-05_EHD73192.1

MKSKRMEFHNKFLIVSAMLAVISWLSLGVVSFPMLAGTLGISTKAAATVVNLISAYSTVTAVISIVGAITGVGSIGSGIAATVLYILKKKGAAKAALW

>Streptococcus-pneumoniae-GA44511_EHD81823.1

MKSKRMEFHNKFLIVSAMLAVISWLSLGVVSFPMLAGTLGISTKAAATVVNLISAYSTVTAVISIVGAITGVGSIGSGIAATVLYILKKKGAAKAALW

>Streptococcus-pneumoniae-GA13494_EHD90839.1

MKSKRMEFHNKFLIVSAMLAVISWLSLGVVSFPMLAGTLGISTKAAATVVNLISAYSTVTAVISIVGAITGVGSIGSGIAATVLYILKKKGAAKAALW

>Streptococcus-pneumoniae-GA17971_EHE11877.1

MKSKRMEFHNKFLIVSAMLAVISWLSLGVVSFPMLAGTLGISTKAAATVVNLISAYSTVTAVISIVGAITGVGSIGSGIAATVLYILKKKGAAKAALW

>Streptococcus-pneumoniae-GA41277_EHE19224.1

MKSKRMEFHNKFLIVSAMLAVISWLSLGVVSFPMLAGTLGISTKAAATVVNLISAYSTVTAVISIVGAITGVGSIGSGIAATVLYILKKKGAAKAALW

>Streptococcus-pneumoniae-GA47388_EHE38073.1

MKSKRMEFHNKFLIVSAMLAVISWLSLGVVSFPMLAGTLGISTKAAATVVNLISAYSTVTAVISIVGAITGVGSIGSGIAATVLYILKKKGAAKAALW

>Streptococcus-pneumoniae-GA52306_EHE49875.1

MKSKRMEFHNKFLIVSAMLAVISWLSLGVVSFPMLAGTLGISTKAAATVVNLISAYSTVTAVISIVGAITGVGSIGSGIAATVLYILKKKGAAKAALW

>Streptococcus-pneumoniae-GA54644_EHE50397.1

MKSKRMEFHNKFLIVSAMLAVISWLSLGVVSFPMLAGTLGISTKAAATVVNLISAYSTVTAVISIVGAITGVGSIGSGIAATVLYILKKKGAAKAALW

>Streptococcus-pneumoniae-Netherlands15B-37_EHE54297.1

MKSKRMEFHNKFLIVSAMLAVISWLSLGVVSFPMLAGTLGISTKAAATVVNLISAYSTVTAVISIVGAITGVGSIGSGIAATVLYILKKKGAAKAALW

>Streptococcus-pneumoniae-GA08780_EHE69615.1

MKSKRMEFHNKFLIVSAMLAVISWLSLGVVSFPMLAGTLGISTKAAATVVNLISAYSTVTAVISIVGAITGVGSIGSGIAATVLYILKKKGAAKAALW

>Streptococcus-pneumoniae-GA11426_EHE79282.1

MKSKRMEFHNKFLIVSAMLAVISWLSLGVVSFPMLAGTLGISTKAAATVVNLISAYSTVTAVISIVGAITGVGSIGSGIAATVLYILKKKGAAKAALW

>Streptococcus-pneumoniae-GA02254_EHY98539.1

MKSKRMEFHNKFLIVSAMLAVISWLSLGVVSFPMLAGTLGISTKAAATVVNLISAYSTVTAVISIVGAITGVGSIGSGIAATVLYILKKKGAAKAALW

>Streptococcus-pneumoniae-GA04175_EHZ06199.1

MKSKRMEFHNKFLIVSAMLAVISWLSLGVVSFPMLAGTLGISTKAAATVVNLISAYSTVTAVISIVGAITGVGSIGSGIAATVLYILKKKGAAKAALW

>Streptococcus-pneumoniae-GA13224_EHZ20708.1

MKSKRMEFHNKFLIVSAMLAVISWLSLGVVSFPMLAGTLGISTKAAATVVNLISAYSTVTAVISIVGAITGVGSIGSGIAATVLYILKKKGAAKAALW

>Streptococcus-pneumoniae-GA14688_EHZ26949.1

MKSKRMEFHNKFLIVSAMLAVISWLSLGVVSFPMLAGTLGISTKAAATVVNLISAYSTVTAVISIVGAITGVGSIGSGIAATVLYILKKKGAAKAALW

>Streptococcus-pneumoniae-GA17457_EHZ33945.1

MKSKRMEFHNKFLIVSAMLAVISWLSLGVVSFPMLAGTLGISTKAAATVVNLISAYSTVTAVISIVGAITGVGSIGSGIAATVLYILKKKGAAKAALW

>Streptococcus-pneumoniae-GA40183_EHZ43727.1

MKSKRMEFHNKFLIVSAMLAVISWLSLGVVSFPMLAGTLGISTKAAATVVNLISAYSTVTAVISIVGAITGVGSIGSGIAATVLYILKKKGAAKAALW

>Streptococcus-pneumoniae-GA40410_EHZ47285.1

MKSKRMEFHNKFLIVSAMLAVISWLSLGVVSFPMLAGTLGISTKAAATVVNLISAYSTVTAVISIVGAITGVGSIGSGIAATVLYILKKKGAAKAALW

>Streptococcus-pneumoniae-GA43257_EHZ47702.1

MKSKRMEFHNKFLIVSAMLAVISWLSLGVVSFPMLAGTLGISTKAAATVVNLISAYSTVTAVISIVGAITGVGSIGSGIAATVLYILKKKGAAKAALW

>Streptococcus-pneumoniae-GA44128_EHZ53276.1

MKSKRMEFHNKFLIVSAMLAVISWLSLGVVSFPMLAGTLGISTKAAATVVNLISAYSTVTAVISIVGAITGVGSIGSGIAATVLYILKKKGAAKAALW

>Streptococcus-pneumoniae-GA47522_EHZ67390.1

MKSKRMEFHNKFLIVSAMLAVISWLSLGVVSFPMLAGTLGISTKAAATVVNLISAYSTVTAVISIVGAITGVGSIGSGIAATVLYILKKKGAAKAALW

>Streptococcus-pneumoniae-GA49542_EHZ77999.1

MKSKRMEFHNKFLIVSAMLAVISWLSLGVVSFPMLAGTLGISTKAAATVVNLISAYSTVTAVISIVGAITGVGSIGSGIAATVLYILKKKGAAKAALW

>Streptococcus-pneumoniae-EU-NP04_EHZ95490.1

MKSKRMEFHNKFLIVSAMLAVISWLSLGVVSFPMLAGTLGISTKAAATVVNLISAYSTVTAVISIVGAITGVGSIGSGIAATVLYILKKKGAAKAALW

>Streptococcus-pseudopneumoniae-ATCC-BAA-960-=-CCUG-49455_EID24185.1

MKSKRMEFHNKFLIVSAMLAVISWLSLGVVSFPMLAGTLGISTKAAATVVNLISAYSTVTAVISIVGAITGVGSIGSGIAATVLYILKKKGAAKAALW

>Streptococcus-pneumoniae-2070035_EJG37000.1

MKSKRMEFHNKFLIVSAMLAVISWLSLGVVSFPMLAGTLGISTKAAATVVNLISAYSTVTAVISIVGAITGVGSIGSGIAATVLYILKKKGAAKAALW

>Streptococcus-pneumoniae-2070005_EJG37998.1

MKSKRMEFHNKFLIVSAMLAVISWLSLGVVSFPMLAGTLGISTKAAATVVNLISAYSTVTAVISIVGAITGVGSIGSGIAATVLYILKKKGAAKAALW

>Streptococcus-pneumoniae-2070335_EJG46679.1

MKSKRMEFHNKFLIVSAMLAVISWLSLGVVSFPMLAGTLGISTKAAATVVNLISAYSTVTAVISIVGAITGVGSIGSGIAATVLYILKKKGAAKAALW

>Streptococcus-pneumoniae-2070531_EJG51399.1

MKSKRMEFHNKFLIVSAMLAVISWLSLGVVSFPMLAGTLGISTKAAATVVNLISAYSTVTAVISIVGAITGVGSIGSGIAATVLYILKKKGAAKAALW

>Streptococcus-pneumoniae-2070425_EJG52273.1

MKSKRMEFHNKFLIVSAMLAVISWLSLGVVSFPMLAGTLGISTKAAATVVNLISAYSTVTAVISIVGAITGVGSIGSGIAATVLYILKKKGAAKAALW

>Streptococcus-pneumoniae-2070768_EJG52413.1

MKSKRMEFHNKFLIVSAMLAVISWLSLGVVSFPMLAGTLGISTKAAATVVNLISAYSTVTAVISIVGAITGVGSIGSGIAATVLYILKKKGAAKAALW

>Streptococcus-pneumoniae-2080076_EJG58617.1

MKSKRMEFHNKFLIVSAMLAVISWLSLGVVSFPMLAGTLGISTKAAATVVNLISAYSTVTAVISIVGAITGVGSIGSGIAATVLYILKKKGAAKAALW

>Streptococcus-pneumoniae-2061617_EJG59224.1

MKSKRMEFHNKFLIVSAMLAVISWLSLGVVSFPMLAGTLGISTKAAATVVNLISAYSTVTAVISIVGAITGVGSIGSGIAATVLYILKKKGAAKAALW

>Streptococcus-pneumoniae-2080913_EJG71487.1

MKSKRMEFHNKFLIVSAMLAVISWLSLGVVSFPMLAGTLGISTKAAATVVNLISAYSTVTAVISIVGAITGVGSIGSGIAATVLYILKKKGAAKAALW

>Streptococcus-pneumoniae-2082239_EJG75980.1

MKSKRMEFHNKFLIVSAMLAVISWLSLGVVSFPMLAGTLGISTKAAATVVNLISAYSTVTAVISIVGAITGVGSIGSGIAATVLYILKKKGAAKAALW

>Streptococcus-pneumoniae-SPAR95_EJG83466.1

MKSKRMEFHNKFLIVSAMLAVISWLSLGVVSFPMLAGTLGISTKAAATVVNLISAYSTVTAVISIVGAITGVGSIGSGIAATVLYILKKKGAAKAALW

>Streptococcus-pneumoniae-GA17301_EJG94005.1

MKSKRMEFHNKFLIVSAMLAVISWLSLGVVSFPMLAGTLGISTKAAATVVNLISAYSTVTAVISIVGAITGVGSIGSGIAATVLYILKKKGAAKAALW

>Streptococcus-pneumoniae-GA04672_EJG94491.1

MKSKRMEFHNKFLIVSAMLAVISWLSLGVVSFPMLAGTLGISTKAAATVVNLISAYSTVTAVISIVGAITGVGSIGSGIAATVLYILKKKGAAKAALW

>Streptococcus-pneumoniae-GA60132_EJH20831.1

MKSKRMEFHNKFLIVSAMLAVISWLSLGVVSFPMLAGTLGISTKAAATVVNLISAYSTVTAVISIVGAITGVGSIGSGIAATVLYILKKKGAAKAALW

>Streptococcus-pneumoniae-2009_EOB17882.1

MKSKRMEFHNKFLIVSAMLAVISWLSLGVVSFPMLAGTLGISTKAAATVVNLISAYSTVTAVISIVGAITGVGSIGSGIAATVLYILKKKGAAKAALW

>Streptococcus-pneumoniae-801_EOB19656.1

MKSKRMEFHNKFLIVSAMLAVISWLSLGVVSFPMLAGTLGISTKAAATVVNLISAYSTVTAVISIVGAITGVGSIGSGIAATVLYILKKKGAAKAALW

>Streptococcus-pneumoniae-3051_EOB24553.1

MKSKRMEFHNKFLIVSAMLAVISWLSLGVVSFPMLAGTLGISTKAAATVVNLISAYSTVTAVISIVGAITGVGSIGSGIAATVLYILKKKGAAKAALW

>Streptococcus-pneumoniae-MNZ11b_EPD16058.1

MKSKRMEFHNKFLIVSAMLAVISWLSLGVVSFPMLAGTLGISTKAAATVVNLISAYSTVTAVISIVGAITGVGSIGSGIAATVLYILKKKGAAKAALW

>Streptococcus-pneumoniae-MNZ37_EPD16700.1

MKSKRMEFHNKFLIVSAMLAVISWLSLGVVSFPMLAGTLGISTKAAATVVNLISAYSTVTAVISIVGAITGVGSIGSGIAATVLYILKKKGAAKAALW

>Streptococcus-pneumoniae-MNZ41_EPD22567.1

MKSKRMEFHNKFLIVSAMLAVISWLSLGVVSFPMLAGTLGISTKAAATVVNLISAYSTVTAVISIVGAITGVGSIGSGIAATVLYILKKKGAAKAALW

>Streptococcus-pneumoniae-MNZ85_EPF48879.1

MKSKRMEFHNKFLIVSAMLAVISWLSLGVVSFPMLAGTLGISTKAAATVVNLISAYSTVTAVISIVGAITGVGSIGSGIAATVLYILKKKGAAKAALW

>Streptococcus-pseudopneumoniae-1321_ETD93798.1

MKSKRMEFHNKFLIVSAMLAVISWLSLGVVSFPMLAGTLGISTKAAATVVNLISAYSTVTAVISIVGAITGVGSIGSGIAATVLYILKKKGAAKAALW

>Streptococcus-pneumoniae-27_ETE01280.1

MKSKRMEFHNKFLIVSAMLAVISWLSLGVVSFPMLAGTLGISTKAAATVVNLISAYSTVTAVISIVGAITGVGSIGSGIAATVLYILKKKGAAKAALW

>Streptococcus-pseudopneumoniae-22725_ETE07336.1

MKSKRMEFHNKFLIVSAMLAVISWLSLGVVSFPMLAGTLGISTKAAATVVNLISAYSTVTAVISIVGAITGVGSIGSGIAATVLYILKKKGAAKAALW

>Streptococcus-pseudopneumoniae-G42_ETE08356.1

MKSKRMEFHNKFLIVSAMLAVISWLSLGVVSFPMLAGTLGISTKAAATVVNLISAYSTVTAVISIVGAITGVGSIGSGIAATVLYILKKKGAAKAALW

>Streptococcus-pneumoniae-13856_ETE16518.1

MKSKRMEFHNKFLIVSAMLAVISWLSLGVVSFPMLAGTLGISTKAAATVVNLISAYSTVTAVISIVGAITGVGSIGSGIAATVLYILKKKGAAKAALW

>Streptococcus-pneumoniae-1719_ETE26857.1

MKSKRMEFHNKFLIVSAMLAVISWLSLGVVSFPMLAGTLGISTKAAATVVNLISAYSTVTAVISIVGAITGVGSIGSGIAATVLYILKKKGAAKAALW

>Streptococcus-pneumoniae-DAR831_KAA02348.1

MKSKRMEFHNKFLIVSAMLAVISWLSLGVVSFPMLAGTLGISTKAAATVVNLISAYSTVTAVISIVGAITGVGSIGSGIAATVLYILKKKGAAKAALW

>Streptococcus-pneumoniae_KDA40108.1

MKSKRMEFHNKFLIVSAMLAVISWLSLGVVSFPMLAGTLGISTKAAATVVNLISAYSTVTAVISIVGAITGVGSIGSGIAATVLYILKKKGAAKAALW

>Streptococcus-pneumoniae_KDE93524.1

MKSKRMEFHNKFLIVSAMLAVISWLSLGVVSFPMLAGTLGISTKAAATVVNLISAYSTVTAVISIVGAITGVGSIGSGIAATVLYILKKKGAAKAALW

>Streptococcus-pneumoniae_KGI24285.1

MKSKRMEFHNKFLIVSAMLAVISWLSLGVVSFPMLAGTLGISTKAAATVVNLISAYSTVTAVISIVGAITGVGSIGSGIAATVLYILKKKGAAKAALW

>Streptococcus-pneumoniae_KGI28515.1

MKSKRMEFHNKFLIVSAMLAVISWLSLGVVSFPMLAGTLGISTKAAATVVNLISAYSTVTAVISIVGAITGVGSIGSGIAATVLYILKKKGAAKAALW

>Streptococcus-pneumoniae_KGI30845.1

MKSKRMEFHNKFLIVSAMLAVISWLSLGVVSFPMLAGTLGISTKAAATVVNLISAYSTVTAVISIVGAITGVGSIGSGIAATVLYILKKKGAAKAALW

>Streptococcus-pneumoniae_KGI33304.1

MKSKRMEFHNKFLIVSAMLAVISWLSLGVVSFPMLAGTLGISTKAAATVVNLISAYSTVTAVISIVGAITGVGSIGSGIAATVLYILKKKGAAKAALW

>Streptococcus-pneumoniae_AJD71006.1

MKSKRMEFHNKFLIVSAMLAVISWLSLGVVSFPMLAGTLGISTKAAATVVNLISAYSTVTAVISIVGAITGVGSIGSGIAATVLYILKKKGAAKAALW

>Streptococcus-pneumoniae_CEO74529.1

MKSKRMEFHNKFLIVSAMLAVISWLSLGVVSFPMLAGTLGISTKAAATVVNLISAYSTVTAVISIVGAITGVGSIGSGIAATVLYILKKKGAAKAALW

>Streptococcus-pneumoniae_CEO80945.1

MKSKRMEFHNKFLIVSAMLAVISWLSLGVVSFPMLAGTLGISTKAAATVVNLISAYSTVTAVISIVGAITGVGSIGSGIAATVLYILKKKGAAKAALW

>Streptococcus-pneumoniae_CEO71680.1

MKSKRMEFHNKFLIVSAMLAVISWLSLGVVSFPMLAGTLGISTKAAATVVNLISAYSTVTAVISIVGAITGVGSIGSGIAATVLYILKKKGAAKAALW

>Streptococcus-pneumoniae_CEO78384.1

MKSKRMEFHNKFLIVSAMLAVISWLSLGVVSFPMLAGTLGISTKAAATVVNLISAYSTVTAVISIVGAITGVGSIGSGIAATVLYILKKKGAAKAALW

>Streptococcus-pneumoniae_CEO76776.1

MKSKRMEFHNKFLIVSAMLAVISWLSLGVVSFPMLAGTLGISTKAAATVVNLISAYSTVTAVISIVGAITGVGSIGSGIAATVLYILKKKGAAKAALW

>Streptococcus-pneumoniae_CEO69970.1

MKSKRMEFHNKFLIVSAMLAVISWLSLGVVSFPMLAGTLGISTKAAATVVNLISAYSTVTAVISIVGAITGVGSIGSGIAATVLYILKKKGAAKAALW

>Streptococcus-pneumoniae_CEV50578.1

MKSKRMEFHNKFLIVSAMLAVISWLSLGVVSFPMLAGTLGISTKAAATVVNLISAYSTVTAVISIVGAITGVGSIGSGIAATVLYILKKKGAAKAALW

>Streptococcus-pneumoniae_CEV58075.1

MKSKRMEFHNKFLIVSAMLAVISWLSLGVVSFPMLAGTLGISTKAAATVVNLISAYSTVTAVISIVGAITGVGSIGSGIAATVLYILKKKGAAKAALW

>Streptococcus-pneumoniae_CEV69022.1

MKSKRMEFHNKFLIVSAMLAVISWLSLGVVSFPMLAGTLGISTKAAATVVNLISAYSTVTAVISIVGAITGVGSIGSGIAATVLYILKKKGAAKAALW

>Streptococcus-pneumoniae_CEV70676.1

MKSKRMEFHNKFLIVSAMLAVISWLSLGVVSFPMLAGTLGISTKAAATVVNLISAYSTVTAVISIVGAITGVGSIGSGIAATVLYILKKKGAAKAALW

>Streptococcus-pneumoniae_CEV76495.1

MKSKRMEFHNKFLIVSAMLAVISWLSLGVVSFPMLAGTLGISTKAAATVVNLISAYSTVTAVISIVGAITGVGSIGSGIAATVLYILKKKGAAKAALW

>Streptococcus-pneumoniae_CEV88097.1

MKSKRMEFHNKFLIVSAMLAVISWLSLGVVSFPMLAGTLGISTKAAATVVNLISAYSTVTAVISIVGAITGVGSIGSGIAATVLYILKKKGAAKAALW

>Streptococcus-pneumoniae_CEW00982.1

MKSKRMEFHNKFLIVSAMLAVISWLSLGVVSFPMLAGTLGISTKAAATVVNLISAYSTVTAVISIVGAITGVGSIGSGIAATVLYILKKKGAAKAALW

>Streptococcus-pneumoniae_CEW12205.1

MKSKRMEFHNKFLIVSAMLAVISWLSLGVVSFPMLAGTLGISTKAAATVVNLISAYSTVTAVISIVGAITGVGSIGSGIAATVLYILKKKGAAKAALW

>Streptococcus-pneumoniae_CEW17979.1

MKSKRMEFHNKFLIVSAMLAVISWLSLGVVSFPMLAGTLGISTKAAATVVNLISAYSTVTAVISIVGAITGVGSIGSGIAATVLYILKKKGAAKAALW

>Streptococcus-pneumoniae_CEW25936.1

MKSKRMEFHNKFLIVSAMLAVISWLSLGVVSFPMLAGTLGISTKAAATVVNLISAYSTVTAVISIVGAITGVGSIGSGIAATVLYILKKKGAAKAALW

>Streptococcus-pneumoniae_CEW29985.1

MKSKRMEFHNKFLIVSAMLAVISWLSLGVVSFPMLAGTLGISTKAAATVVNLISAYSTVTAVISIVGAITGVGSIGSGIAATVLYILKKKGAAKAALW

>Streptococcus-pneumoniae_CEW34551.1

MKSKRMEFHNKFLIVSAMLAVISWLSLGVVSFPMLAGTLGISTKAAATVVNLISAYSTVTAVISIVGAITGVGSIGSGIAATVLYILKKKGAAKAALW

>Streptococcus-pneumoniae_CEW44671.1

MKSKRMEFHNKFLIVSAMLAVISWLSLGVVSFPMLAGTLGISTKAAATVVNLISAYSTVTAVISIVGAITGVGSIGSGIAATVLYILKKKGAAKAALW

>Streptococcus-pneumoniae_CEW79329.1

MKSKRMEFHNKFLIVSAMLAVISWLSLGVVSFPMLAGTLGISTKAAATVVNLISAYSTVTAVISIVGAITGVGSIGSGIAATVLYILKKKGAAKAALW

>Streptococcus-pneumoniae_CEW91410.1

MKSKRMEFHNKFLIVSAMLAVISWLSLGVVSFPMLAGTLGISTKAAATVVNLISAYSTVTAVISIVGAITGVGSIGSGIAATVLYILKKKGAAKAALW

>Streptococcus-pneumoniae_CEW93465.1

MKSKRMEFHNKFLIVSAMLAVISWLSLGVVSFPMLAGTLGISTKAAATVVNLISAYSTVTAVISIVGAITGVGSIGSGIAATVLYILKKKGAAKAALW

>Streptococcus-pneumoniae_CEX16387.1

MKSKRMEFHNKFLIVSAMLAVISWLSLGVVSFPMLAGTLGISTKAAATVVNLISAYSTVTAVISIVGAITGVGSIGSGIAATVLYILKKKGAAKAALW

>Streptococcus-pneumoniae_CEX19954.1

MKSKRMEFHNKFLIVSAMLAVISWLSLGVVSFPMLAGTLGISTKAAATVVNLISAYSTVTAVISIVGAITGVGSIGSGIAATVLYILKKKGAAKAALW

>Streptococcus-pneumoniae_CEX26137.1

MKSKRMEFHNKFLIVSAMLAVISWLSLGVVSFPMLAGTLGISTKAAATVVNLISAYSTVTAVISIVGAITGVGSIGSGIAATVLYILKKKGAAKAALW

>Streptococcus-pneumoniae_CEX53688.1

MKSKRMEFHNKFLIVSAMLAVISWLSLGVVSFPMLAGTLGISTKAAATVVNLISAYSTVTAVISIVGAITGVGSIGSGIAATVLYILKKKGAAKAALW

>Streptococcus-pneumoniae_CEY00604.1

MKSKRMEFHNKFLIVSAMLAVISWLSLGVVSFPMLAGTLGISTKAAATVVNLISAYSTVTAVISIVGAITGVGSIGSGIAATVLYILKKKGAAKAALW

>Streptococcus-pneumoniae_CEX97714.1

MKSKRMEFHNKFLIVSAMLAVISWLSLGVVSFPMLAGTLGISTKAAATVVNLISAYSTVTAVISIVGAITGVGSIGSGIAATVLYILKKKGAAKAALW

>Streptococcus-pneumoniae_CEX97723.1

MKSKRMEFHNKFLIVSAMLAVISWLSLGVVSFPMLAGTLGISTKAAATVVNLISAYSTVTAVISIVGAITGVGSIGSGIAATVLYILKKKGAAKAALW

>Streptococcus-pneumoniae_CEX97728.1

MKSKRMEFHNKFLIVSAMLAVISWLSLGVVSFPMLAGTLGISTKAAATVVNLISAYSTVTAVISIVGAITGVGSIGSGIAATVLYILKKKGAAKAALW

>Streptococcus-pneumoniae_CEY02890.1

MKSKRMEFHNKFLIVSAMLAVISWLSLGVVSFPMLAGTLGISTKAAATVVNLISAYSTVTAVISIVGAITGVGSIGSGIAATVLYILKKKGAAKAALW

>Streptococcus-pneumoniae_CEY11782.1

MKSKRMEFHNKFLIVSAMLAVISWLSLGVVSFPMLAGTLGISTKAAATVVNLISAYSTVTAVISIVGAITGVGSIGSGIAATVLYILKKKGAAKAALW

>Streptococcus-pneumoniae_CEY23928.1

MKSKRMEFHNKFLIVSAMLAVISWLSLGVVSFPMLAGTLGISTKAAATVVNLISAYSTVTAVISIVGAITGVGSIGSGIAATVLYILKKKGAAKAALW

>Streptococcus-pneumoniae_CEY26320.1

MKSKRMEFHNKFLIVSAMLAVISWLSLGVVSFPMLAGTLGISTKAAATVVNLISAYSTVTAVISIVGAITGVGSIGSGIAATVLYILKKKGAAKAALW

>Streptococcus-pneumoniae_CEY61056.1

MKSKRMEFHNKFLIVSAMLAVISWLSLGVVSFPMLAGTLGISTKAAATVVNLISAYSTVTAVISIVGAITGVGSIGSGIAATVLYILKKKGAAKAALW

>Streptococcus-pneumoniae_CEY64412.1

MKSKRMEFHNKFLIVSAMLAVISWLSLGVVSFPMLAGTLGISTKAAATVVNLISAYSTVTAVISIVGAITGVGSIGSGIAATVLYILKKKGAAKAALW

>Streptococcus-pneumoniae_CEZ03511.1

MKSKRMEFHNKFLIVSAMLAVISWLSLGVVSFPMLAGTLGISTKAAATVVNLISAYSTVTAVISIVGAITGVGSIGSGIAATVLYILKKKGAAKAALW

>Streptococcus-pneumoniae_CEZ11146.1

MKSKRMEFHNKFLIVSAMLAVISWLSLGVVSFPMLAGTLGISTKAAATVVNLISAYSTVTAVISIVGAITGVGSIGSGIAATVLYILKKKGAAKAALW

>Streptococcus-pneumoniae_CEZ18442.1

MKSKRMEFHNKFLIVSAMLAVISWLSLGVVSFPMLAGTLGISTKAAATVVNLISAYSTVTAVISIVGAITGVGSIGSGIAATVLYILKKKGAAKAALW

>Streptococcus-pneumoniae_CFA01089.1

MKSKRMEFHNKFLIVSAMLAVISWLSLGVVSFPMLAGTLGISTKAAATVVNLISAYSTVTAVISIVGAITGVGSIGSGIAATVLYILKKKGAAKAALW

>Streptococcus-pneumoniae_CFK39405.1

MKSKRMEFHNKFLIVSAMLAVISWLSLGVVSFPMLAGTLGISTKAAATVVNLISAYSTVTAVISIVGAITGVGSIGSGIAATVLYILKKKGAAKAALW

>Streptococcus-pneumoniae_CEW21559.1

MKSKRMEFHNKFLIVSAMLAVISWLSLGVVSFPMLAGTLGISTKAAATVVNLISAYSTVTAVISIVGAITGVGSIGSGIAATVLYILKKKGAAKAALW

>Streptococcus-pneumoniae_CEX12970.1

MKSKRMEFHNKFLIVSAMLAVISWLSLGVVSFPMLAGTLGISTKAAATVVNLISAYSTVTAVISIVGAITGVGSIGSGIAATVLYILKKKGAAKAALW

>Streptococcus-pneumoniae_CEX37336.1

MKSKRMEFHNKFLIVSAMLAVISWLSLGVVSFPMLAGTLGISTKAAATVVNLISAYSTVTAVISIVGAITGVGSIGSGIAATVLYILKKKGAAKAALW

>Streptococcus-pneumoniae_CEX40027.1

MKSKRMEFHNKFLIVSAMLAVISWLSLGVVSFPMLAGTLGISTKAAATVVNLISAYSTVTAVISIVGAITGVGSIGSGIAATVLYILKKKGAAKAALW

>Streptococcus-pneumoniae_CEX75264.1

MKSKRMEFHNKFLIVSAMLAVISWLSLGVVSFPMLAGTLGISTKAAATVVNLISAYSTVTAVISIVGAITGVGSIGSGIAATVLYILKKKGAAKAALW

>Streptococcus-pneumoniae_CEY36005.1

MKSKRMEFHNKFLIVSAMLAVISWLSLGVVSFPMLAGTLGISTKAAATVVNLISAYSTVTAVISIVGAITGVGSIGSGIAATVLYILKKKGAAKAALW

>Streptococcus-pneumoniae_CEY62534.1

MKSKRMEFHNKFLIVSAMLAVISWLSLGVVSFPMLAGTLGISTKAAATVVNLISAYSTVTAVISIVGAITGVGSIGSGIAATVLYILKKKGAAKAALW

>Streptococcus-pneumoniae_CEZ50397.1

MKSKRMEFHNKFLIVSAMLAVISWLSLGVVSFPMLAGTLGISTKAAATVVNLISAYSTVTAVISIVGAITGVGSIGSGIAATVLYILKKKGAAKAALW

>Streptococcus-pneumoniae_CFB58706.1

MKSKRMEFHNKFLIVSAMLAVISWLSLGVVSFPMLAGTLGISTKAAATVVNLISAYSTVTAVISIVGAITGVGSIGSGIAATVLYILKKKGAAKAALW

>Streptococcus-pneumoniae_CFE10084.1

MKSKRMEFHNKFLIVSAMLAVISWLSLGVVSFPMLAGTLGISTKAAATVVNLISAYSTVTAVISIVGAITGVGSIGSGIAATVLYILKKKGAAKAALW

>Streptococcus-pneumoniae_CFP78579.1

MKSKRMEFHNKFLIVSAMLAVISWLSLGVVSFPMLAGTLGISTKAAATVVNLISAYSTVTAVISIVGAITGVGSIGSGIAATVLYILKKKGAAKAALW

>Streptococcus-pneumoniae_CRC32937.1

MKSKRMEFHNKFLIVSAMLAVISWLSLGVVSFPMLAGTLGISTKAAATVVNLISAYSTVTAVISIVGAITGVGSIGSGIAATVLYILKKKGAAKAALW

>Streptococcus-pneumoniae_CRC25227.1

MKSKRMEFHNKFLIVSAMLAVISWLSLGVVSFPMLAGTLGISTKAAATVVNLISAYSTVTAVISIVGAITGVGSIGSGIAATVLYILKKKGAAKAALW

>Streptococcus-pneumoniae_CRF13184.1

MKSKRMEFHNKFLIVSAMLAVISWLSLGVVSFPMLAGTLGISTKAAATVVNLISAYSTVTAVISIVGAITGVGSIGSGIAATVLYILKKKGAAKAALW

>Streptococcus-pneumoniae_CRF30372.1

MKSKRMEFHNKFLIVSAMLAVISWLSLGVVSFPMLAGTLGISTKAAATVVNLISAYSTVTAVISIVGAITGVGSIGSGIAATVLYILKKKGAAKAALW

>Streptococcus-pneumoniae_CRF94166.1

MKSKRMEFHNKFLIVSAMLAVISWLSLGVVSFPMLAGTLGISTKAAATVVNLISAYSTVTAVISIVGAITGVGSIGSGIAATVLYILKKKGAAKAALW

>Streptococcus-pneumoniae_CJD41373.1

MKSKRMEFHNKFLIVSAMLAVISWLSLGVVSFPMLAGTLGISTKAAATVVNLISAYSTVTAVISIVGAITGVGSIGSGIAATVLYILKKKGAAKAALW

>Streptococcus-pneumoniae_CJB46737.1

MKSKRMEFHNKFLIVSAMLAVISWLSLGVVSFPMLAGTLGISTKAAATVVNLISAYSTVTAVISIVGAITGVGSIGSGIAATVLYILKKKGAAKAALW

>Streptococcus-pneumoniae_CIS74627.1

MKSKRMEFHNKFLIVSAMLAVISWLSLGVVSFPMLAGTLGISTKAAATVVNLISAYSTVTAVISIVGAITGVGSIGSGIAATVLYILKKKGAAKAALW

>Streptococcus-pneumoniae_CJN82289.1

MKSKRMEFHNKFLIVSAMLAVISWLSLGVVSFPMLAGTLGISTKAAATVVNLISAYSTVTAVISIVGAITGVGSIGSGIAATVLYILKKKGAAKAALW

>Streptococcus-pneumoniae_CIP53671.1

MKSKRMEFHNKFLIVSAMLAVISWLSLGVVSFPMLAGTLGISTKAAATVVNLISAYSTVTAVISIVGAITGVGSIGSGIAATVLYILKKKGAAKAALW

>Streptococcus-pneumoniae_COG17956.1

MKSKRMEFHNKFLIVSAMLAVISWLSLGVVSFPMLAGTLGISTKAAATVVNLISAYSTVTAVISIVGAITGVGSIGSGIAATVLYILKKKGAAKAALW

>Streptococcus-pneumoniae_CJL80022.1

MKSKRMEFHNKFLIVSAMLAVISWLSLGVVSFPMLAGTLGISTKAAATVVNLISAYSTVTAVISIVGAITGVGSIGSGIAATVLYILKKKGAAKAALW

>Streptococcus-pneumoniae_CGE89024.1

MKSKRMEFHNKFLIVSAMLAVISWLSLGVVSFPMLAGTLGISTKAAATVVNLISAYSTVTAVISIVGAITGVGSIGSGIAATVLYILKKKGAAKAALW

>Streptococcus-pneumoniae_CGF82321.1

MKSKRMEFHNKFLIVSAMLAVISWLSLGVVSFPMLAGTLGISTKAAATVVNLISAYSTVTAVISIVGAITGVGSIGSGIAATVLYILKKKGAAKAALW

>Streptococcus-pneumoniae_CIZ43360.1

MKSKRMEFHNKFLIVSAMLAVISWLSLGVVSFPMLAGTLGISTKAAATVVNLISAYSTVTAVISIVGAITGVGSIGSGIAATVLYILKKKGAAKAALW

>Streptococcus-pneumoniae_COK82599.1

MKSKRMEFHNKFLIVSAMLAVISWLSLGVVSFPMLAGTLGISTKAAATVVNLISAYSTVTAVISIVGAITGVGSIGSGIAATVLYILKKKGAAKAALW

>Streptococcus-pneumoniae_CJZ27396.1

MKSKRMEFHNKFLIVSAMLAVISWLSLGVVSFPMLAGTLGISTKAAATVVNLISAYSTVTAVISIVGAITGVGSIGSGIAATVLYILKKKGAAKAALW

>Streptococcus-pneumoniae_CIT62391.1

MKSKRMEFHNKFLIVSAMLAVISWLSLGVVSFPMLAGTLGISTKAAATVVNLISAYSTVTAVISIVGAITGVGSIGSGIAATVLYILKKKGAAKAALW

>Streptococcus-pneumoniae_CNA72164.1

MKSKRMEFHNKFLIVSAMLAVISWLSLGVVSFPMLAGTLGISTKAAATVVNLISAYSTVTAVISIVGAITGVGSIGSGIAATVLYILKKKGAAKAALW

>Streptococcus-pneumoniae_CIO74536.1

MKSKRMEFHNKFLIVSAMLAVISWLSLGVVSFPMLAGTLGISTKAAATVVNLISAYSTVTAVISIVGAITGVGSIGSGIAATVLYILKKKGAAKAALW

>Streptococcus-pneumoniae_CMW82810.1

MKSKRMEFHNKFLIVSAMLAVISWLSLGVVSFPMLAGTLGISTKAAATVVNLISAYSTVTAVISIVGAITGVGSIGSGIAATVLYILKKKGAAKAALW

>Streptococcus-pneumoniae_CGG84745.1

MKSKRMEFHNKFLIVSAMLAVISWLSLGVVSFPMLAGTLGISTKAAATVVNLISAYSTVTAVISIVGAITGVGSIGSGIAATVLYILKKKGAAKAALW

>Streptococcus-pneumoniae_CGG12620.1

MKSKRMEFHNKFLIVSAMLAVISWLSLGVVSFPMLAGTLGISTKAAATVVNLISAYSTVTAVISIVGAITGVGSIGSGIAATVLYILKKKGAAKAALW

>Streptococcus-pneumoniae_COK74084.1

MKSKRMEFHNKFLIVSAMLAVISWLSLGVVSFPMLAGTLGISTKAAATVVNLISAYSTVTAVISIVGAITGVGSIGSGIAATVLYILKKKGAAKAALW

>Streptococcus-pneumoniae_CIP20100.1

MKSKRMEFHNKFLIVSAMLAVISWLSLGVVSFPMLAGTLGISTKAAATVVNLISAYSTVTAVISIVGAITGVGSIGSGIAATVLYILKKKGAAKAALW

>Streptococcus-pneumoniae_CJD34135.1

MKSKRMEFHNKFLIVSAMLAVISWLSLGVVSFPMLAGTLGISTKAAATVVNLISAYSTVTAVISIVGAITGVGSIGSGIAATVLYILKKKGAAKAALW

>Streptococcus-pneumoniae_COM90092.1

MKSKRMEFHNKFLIVSAMLAVISWLSLGVVSFPMLAGTLGISTKAAATVVNLISAYSTVTAVISIVGAITGVGSIGSGIAATVLYILKKKGAAKAALW

>Streptococcus-pneumoniae_CJN22312.1

MKSKRMEFHNKFLIVSAMLAVISWLSLGVVSFPMLAGTLGISTKAAATVVNLISAYSTVTAVISIVGAITGVGSIGSGIAATVLYILKKKGAAKAALW

>Streptococcus-pneumoniae_CGE76054.1

MKSKRMEFHNKFLIVSAMLAVISWLSLGVVSFPMLAGTLGISTKAAATVVNLISAYSTVTAVISIVGAITGVGSIGSGIAATVLYILKKKGAAKAALW

>Streptococcus-pneumoniae_CKD67552.1

MKSKRMEFHNKFLIVSAMLAVISWLSLGVVSFPMLAGTLGISTKAAATVVNLISAYSTVTAVISIVGAITGVGSIGSGIAATVLYILKKKGAAKAALW

>Streptococcus-pneumoniae_CNA92163.1

MKSKRMEFHNKFLIVSAMLAVISWLSLGVVSFPMLAGTLGISTKAAATVVNLISAYSTVTAVISIVGAITGVGSIGSGIAATVLYILKKKGAAKAALW

>Streptococcus-pneumoniae_CKB58308.1

MKSKRMEFHNKFLIVSAMLAVISWLSLGVVSFPMLAGTLGISTKAAATVVNLISAYSTVTAVISIVGAITGVGSIGSGIAATVLYILKKKGAAKAALW

>Streptococcus-pneumoniae_CGG41103.1

MKSKRMEFHNKFLIVSAMLAVISWLSLGVVSFPMLAGTLGISTKAAATVVNLISAYSTVTAVISIVGAITGVGSIGSGIAATVLYILKKKGAAKAALW

>Streptococcus-pneumoniae_CJJ71512.1

MKSKRMEFHNKFLIVSAMLAVISWLSLGVVSFPMLAGTLGISTKAAATVVNLISAYSTVTAVISIVGAITGVGSIGSGIAATVLYILKKKGAAKAALW

>Streptococcus-pneumoniae_COE36678.1

MKSKRMEFHNKFLIVSAMLAVISWLSLGVVSFPMLAGTLGISTKAAATVVNLISAYSTVTAVISIVGAITGVGSIGSGIAATVLYILKKKGAAKAALW

>Streptococcus-pneumoniae_COR16868.1

MKSKRMEFHNKFLIVSAMLAVISWLSLGVVSFPMLAGTLGISTKAAATVVNLISAYSTVTAVISIVGAITGVGSIGSGIAATVLYILKKKGAAKAALW

>Streptococcus-pneumoniae_CJI40204.1

MKSKRMEFHNKFLIVSAMLAVISWLSLGVVSFPMLAGTLGISTKAAATVVNLISAYSTVTAVISIVGAITGVGSIGSGIAATVLYILKKKGAAKAALW

>Streptococcus-pneumoniae_CMW96286.1

MKSKRMEFHNKFLIVSAMLAVISWLSLGVVSFPMLAGTLGISTKAAATVVNLISAYSTVTAVISIVGAITGVGSIGSGIAATVLYILKKKGAAKAALW

>Streptococcus-pneumoniae_CJL28436.1

MKSKRMEFHNKFLIVSAMLAVISWLSLGVVSFPMLAGTLGISTKAAATVVNLISAYSTVTAVISIVGAITGVGSIGSGIAATVLYILKKKGAAKAALW

>Streptococcus-pneumoniae_CON64591.1

MKSKRMEFHNKFLIVSAMLAVISWLSLGVVSFPMLAGTLGISTKAAATVVNLISAYSTVTAVISIVGAITGVGSIGSGIAATVLYILKKKGAAKAALW

>Streptococcus-pneumoniae_CGF95565.1

MKSKRMEFHNKFLIVSAMLAVISWLSLGVVSFPMLAGTLGISTKAAATVVNLISAYSTVTAVISIVGAITGVGSIGSGIAATVLYILKKKGAAKAALW

>Streptococcus-pneumoniae_CIT24567.1

MKSKRMEFHNKFLIVSAMLAVISWLSLGVVSFPMLAGTLGISTKAAATVVNLISAYSTVTAVISIVGAITGVGSIGSGIAATVLYILKKKGAAKAALW

>Streptococcus-pneumoniae_COI95172.1

MKSKRMEFHNKFLIVSAMLAVISWLSLGVVSFPMLAGTLGISTKAAATVVNLISAYSTVTAVISIVGAITGVGSIGSGIAATVLYILKKKGAAKAALW

>Streptococcus-pneumoniae_CGG97574.1

MKSKRMEFHNKFLIVSAMLAVISWLSLGVVSFPMLAGTLGISTKAAATVVNLISAYSTVTAVISIVGAITGVGSIGSGIAATVLYILKKKGAAKAALW

>Streptococcus-pneumoniae_CJV37071.1

MKSKRMEFHNKFLIVSAMLAVISWLSLGVVSFPMLAGTLGISTKAAATVVNLISAYSTVTAVISIVGAITGVGSIGSGIAATVLYILKKKGAAKAALW

>Streptococcus-pneumoniae_CGF54646.1

MKSKRMEFHNKFLIVSAMLAVISWLSLGVVSFPMLAGTLGISTKAAATVVNLISAYSTVTAVISIVGAITGVGSIGSGIAATVLYILKKKGAAKAALW

>Streptococcus-pneumoniae_COL85861.1

MKSKRMEFHNKFLIVSAMLAVISWLSLGVVSFPMLAGTLGISTKAAATVVNLISAYSTVTAVISIVGAITGVGSIGSGIAATVLYILKKKGAAKAALW

>Streptococcus-pneumoniae_COR83459.1

MKSKRMEFHNKFLIVSAMLAVISWLSLGVVSFPMLAGTLGISTKAAATVVNLISAYSTVTAVISIVGAITGVGSIGSGIAATVLYILKKKGAAKAALW

>Streptococcus-pneumoniae_CON11383.1

MKSKRMEFHNKFLIVSAMLAVISWLSLGVVSFPMLAGTLGISTKAAATVVNLISAYSTVTAVISIVGAITGVGSIGSGIAATVLYILKKKGAAKAALW

>Streptococcus-pneumoniae_COR06523.1

MKSKRMEFHNKFLIVSAMLAVISWLSLGVVSFPMLAGTLGISTKAAATVVNLISAYSTVTAVISIVGAITGVGSIGSGIAATVLYILKKKGAAKAALW

>Streptococcus-pneumoniae_CIZ53176.1

MKSKRMEFHNKFLIVSAMLAVISWLSLGVVSFPMLAGTLGISTKAAATVVNLISAYSTVTAVISIVGAITGVGSIGSGIAATVLYILKKKGAAKAALW

>Streptococcus-pneumoniae_CJU37046.1

MKSKRMEFHNKFLIVSAMLAVISWLSLGVVSFPMLAGTLGISTKAAATVVNLISAYSTVTAVISIVGAITGVGSIGSGIAATVLYILKKKGAAKAALW

>Streptococcus-pneumoniae_COK41130.1

MKSKRMEFHNKFLIVSAMLAVISWLSLGVVSFPMLAGTLGISTKAAATVVNLISAYSTVTAVISIVGAITGVGSIGSGIAATVLYILKKKGAAKAALW

>Streptococcus-pneumoniae_CGG31012.1

MKSKRMEFHNKFLIVSAMLAVISWLSLGVVSFPMLAGTLGISTKAAATVVNLISAYSTVTAVISIVGAITGVGSIGSGIAATVLYILKKKGAAKAALW

>Streptococcus-pneumoniae_CGF40771.1

MKSKRMEFHNKFLIVSAMLAVISWLSLGVVSFPMLAGTLGISTKAAATVVNLISAYSTVTAVISIVGAITGVGSIGSGIAATVLYILKKKGAAKAALW

>Streptococcus-pneumoniae_CJK04154.1

MKSKRMEFHNKFLIVSAMLAVISWLSLGVVSFPMLAGTLGISTKAAATVVNLISAYSTVTAVISIVGAITGVGSIGSGIAATVLYILKKKGAAKAALW

>Streptococcus-pneumoniae_CON33613.1

MKSKRMEFHNKFLIVSAMLAVISWLSLGVVSFPMLAGTLGISTKAAATVVNLISAYSTVTAVISIVGAITGVGSIGSGIAATVLYILKKKGAAKAALW

>Streptococcus-pneumoniae_CKB38099.1

MKSKRMEFHNKFLIVSAMLAVISWLSLGVVSFPMLAGTLGISTKAAATVVNLISAYSTVTAVISIVGAITGVGSIGSGIAATVLYILKKKGAAKAALW

>Streptococcus-pneumoniae_CIN99116.1

MKSKRMEFHNKFLIVSAMLAVISWLSLGVVSFPMLAGTLGISTKAAATVVNLISAYSTVTAVISIVGAITGVGSIGSGIAATVLYILKKKGAAKAALW

>Streptococcus-pneumoniae_CGG66686.1

MKSKRMEFHNKFLIVSAMLAVISWLSLGVVSFPMLAGTLGISTKAAATVVNLISAYSTVTAVISIVGAITGVGSIGSGIAATVLYILKKKGAAKAALW

>Streptococcus-pneumoniae_CJN54651.1

MKSKRMEFHNKFLIVSAMLAVISWLSLGVVSFPMLAGTLGISTKAAATVVNLISAYSTVTAVISIVGAITGVGSIGSGIAATVLYILKKKGAAKAALW

>Streptococcus-pneumoniae_COF94342.1

MKSKRMEFHNKFLIVSAMLAVISWLSLGVVSFPMLAGTLGISTKAAATVVNLISAYSTVTAVISIVGAITGVGSIGSGIAATVLYILKKKGAAKAALW

>Streptococcus-pneumoniae_COD03818.1

MKSKRMEFHNKFLIVSAMLAVISWLSLGVVSFPMLAGTLGISTKAAATVVNLISAYSTVTAVISIVGAITGVGSIGSGIAATVLYILKKKGAAKAALW

>Streptococcus-pneumoniae_CJP02267.1

MKSKRMEFHNKFLIVSAMLAVISWLSLGVVSFPMLAGTLGISTKAAATVVNLISAYSTVTAVISIVGAITGVGSIGSGIAATVLYILKKKGAAKAALW

>Streptococcus-pneumoniae_CIR60908.1

MKSKRMEFHNKFLIVSAMLAVISWLSLGVVSFPMLAGTLGISTKAAATVVNLISAYSTVTAVISIVGAITGVGSIGSGIAATVLYILKKKGAAKAALW

>Streptococcus-pneumoniae_CJU91169.1

MKSKRMEFHNKFLIVSAMLAVISWLSLGVVSFPMLAGTLGISTKAAATVVNLISAYSTVTAVISIVGAITGVGSIGSGIAATVLYILKKKGAAKAALW

>Streptococcus-pneumoniae_CIS37601.1

MKSKRMEFHNKFLIVSAMLAVISWLSLGVVSFPMLAGTLGISTKAAATVVNLISAYSTVTAVISIVGAITGVGSIGSGIAATVLYILKKKGAAKAALW

>Streptococcus-pneumoniae_CJA66493.1

MKSKRMEFHNKFLIVSAMLAVISWLSLGVVSFPMLAGTLGISTKAAATVVNLISAYSTVTAVISIVGAITGVGSIGSGIAATVLYILKKKGAAKAALW

>Streptococcus-pneumoniae_CJY33455.1

MKSKRMEFHNKFLIVSAMLAVISWLSLGVVSFPMLAGTLGISTKAAATVVNLISAYSTVTAVISIVGAITGVGSIGSGIAATVLYILKKKGAAKAALW

>Streptococcus-pneumoniae_COQ48266.1

MKSKRMEFHNKFLIVSAMLAVISWLSLGVVSFPMLAGTLGISTKAAATVVNLISAYSTVTAVISIVGAITGVGSIGSGIAATVLYILKKKGAAKAALW

>Streptococcus-pneumoniae_CIR10799.1

MKSKRMEFHNKFLIVSAMLAVISWLSLGVVSFPMLAGTLGISTKAAATVVNLISAYSTVTAVISIVGAITGVGSIGSGIAATVLYILKKKGAAKAALW

>Streptococcus-pneumoniae_COB52995.1

MKSKRMEFHNKFLIVSAMLAVISWLSLGVVSFPMLAGTLGISTKAAATVVNLISAYSTVTAVISIVGAITGVGSIGSGIAATVLYILKKKGAAKAALW

>Streptococcus-pneumoniae_CJY20826.1

MKSKRMEFHNKFLIVSAMLAVISWLSLGVVSFPMLAGTLGISTKAAATVVNLISAYSTVTAVISIVGAITGVGSIGSGIAATVLYILKKKGAAKAALW

>Streptococcus-pneumoniae_CMY55260.1

MKSKRMEFHNKFLIVSAMLAVISWLSLGVVSFPMLAGTLGISTKAAATVVNLISAYSTVTAVISIVGAITGVGSIGSGIAATVLYILKKKGAAKAALW

>Streptococcus-pneumoniae_COH62856.1

MKSKRMEFHNKFLIVSAMLAVISWLSLGVVSFPMLAGTLGISTKAAATVVNLISAYSTVTAVISIVGAITGVGSIGSGIAATVLYILKKKGAAKAALW

>Streptococcus-pneumoniae_CGF03499.1

MKSKRMEFHNKFLIVSAMLAVISWLSLGVVSFPMLAGTLGISTKAAATVVNLISAYSTVTAVISIVGAITGVGSIGSGIAATVLYILKKKGAAKAALW

>Streptococcus-pneumoniae_COE83020.1

MKSKRMEFHNKFLIVSAMLAVISWLSLGVVSFPMLAGTLGISTKAAATVVNLISAYSTVTAVISIVGAITGVGSIGSGIAATVLYILKKKGAAKAALW

>Streptococcus-pneumoniae_CNA86954.1

MKSKRMEFHNKFLIVSAMLAVISWLSLGVVSFPMLAGTLGISTKAAATVVNLISAYSTVTAVISIVGAITGVGSIGSGIAATVLYILKKKGAAKAALW

>Streptococcus-pneumoniae_COG69011.1

MKSKRMEFHNKFLIVSAMLAVISWLSLGVVSFPMLAGTLGISTKAAATVVNLISAYSTVTAVISIVGAITGVGSIGSGIAATVLYILKKKGAAKAALW

>Streptococcus-pneumoniae_COM96031.1

MKSKRMEFHNKFLIVSAMLAVISWLSLGVVSFPMLAGTLGISTKAAATVVNLISAYSTVTAVISIVGAITGVGSIGSGIAATVLYILKKKGAAKAALW

>Streptococcus-pneumoniae_COJ42594.1

MKSKRMEFHNKFLIVSAMLAVISWLSLGVVSFPMLAGTLGISTKAAATVVNLISAYSTVTAVISIVGAITGVGSIGSGIAATVLYILKKKGAAKAALW

>Streptococcus-pneumoniae_CGF11728.1

MKSKRMEFHNKFLIVSAMLAVISWLSLGVVSFPMLAGTLGISTKAAATVVNLISAYSTVTAVISIVGAITGVGSIGSGIAATVLYILKKKGAAKAALW

>Streptococcus-pneumoniae_CJW18772.1

MKSKRMEFHNKFLIVSAMLAVISWLSLGVVSFPMLAGTLGISTKAAATVVNLISAYSTVTAVISIVGAITGVGSIGSGIAATVLYILKKKGAAKAALW

>Streptococcus-pneumoniae_CJL74032.1

MKSKRMEFHNKFLIVSAMLAVISWLSLGVVSFPMLAGTLGISTKAAATVVNLISAYSTVTAVISIVGAITGVGSIGSGIAATVLYILKKKGAAKAALW

>Streptococcus-pneumoniae_CJD33243.1

MKSKRMEFHNKFLIVSAMLAVISWLSLGVVSFPMLAGTLGISTKAAATVVNLISAYSTVTAVISIVGAITGVGSIGSGIAATVLYILKKKGAAKAALW

>Streptococcus-pneumoniae_CGG67183.1

MKSKRMEFHNKFLIVSAMLAVISWLSLGVVSFPMLAGTLGISTKAAATVVNLISAYSTVTAVISIVGAITGVGSIGSGIAATVLYILKKKGAAKAALW

>Streptococcus-pneumoniae_CGE90005.1

MKSKRMEFHNKFLIVSAMLAVISWLSLGVVSFPMLAGTLGISTKAAATVVNLISAYSTVTAVISIVGAITGVGSIGSGIAATVLYILKKKGAAKAALW

>Streptococcus-pneumoniae_COG56259.1

MKSKRMEFHNKFLIVSAMLAVISWLSLGVVSFPMLAGTLGISTKAAATVVNLISAYSTVTAVISIVGAITGVGSIGSGIAATVLYILKKKGAAKAALW

>Streptococcus-pneumoniae_CJQ09915.1

MKSKRMEFHNKFLIVSAMLAVISWLSLGVVSFPMLAGTLGISTKAAATVVNLISAYSTVTAVISIVGAITGVGSIGSGIAATVLYILKKKGAAKAALW

>Streptococcus-pneumoniae_CIO68597.1

MKSKRMEFHNKFLIVSAMLAVISWLSLGVVSFPMLAGTLGISTKAAATVVNLISAYSTVTAVISIVGAITGVGSIGSGIAATVLYILKKKGAAKAALW

>Streptococcus-pneumoniae_CJO32782.1

MKSKRMEFHNKFLIVSAMLAVISWLSLGVVSFPMLAGTLGISTKAAATVVNLISAYSTVTAVISIVGAITGVGSIGSGIAATVLYILKKKGAAKAALW

>Streptococcus-pneumoniae_CJC96631.1

MKSKRMEFHNKFLIVSAMLAVISWLSLGVVSFPMLAGTLGISTKAAATVVNLISAYSTVTAVISIVGAITGVGSIGSGIAATVLYILKKKGAAKAALW

>Streptococcus-pneumoniae_COI29340.1

MKSKRMEFHNKFLIVSAMLAVISWLSLGVVSFPMLAGTLGISTKAAATVVNLISAYSTVTAVISIVGAITGVGSIGSGIAATVLYILKKKGAAKAALW

>Streptococcus-pneumoniae_CJN56591.1

MKSKRMEFHNKFLIVSAMLAVISWLSLGVVSFPMLAGTLGISTKAAATVVNLISAYSTVTAVISIVGAITGVGSIGSGIAATVLYILKKKGAAKAALW

>Streptococcus-pneumoniae_CKL63460.1

MKSKRMEFHNKFLIVSAMLAVISWLSLGVVSFPMLAGTLGISTKAAATVVNLISAYSTVTAVISIVGAITGVGSIGSGIAATVLYILKKKGAAKAALW

>Streptococcus-pneumoniae_COD29241.1

MKSKRMEFHNKFLIVSAMLAVISWLSLGVVSFPMLAGTLGISTKAAATVVNLISAYSTVTAVISIVGAITGVGSIGSGIAATVLYILKKKGAAKAALW

>Streptococcus-pneumoniae_COH15141.1

MKSKRMEFHNKFLIVSAMLAVISWLSLGVVSFPMLAGTLGISTKAAATVVNLISAYSTVTAVISIVGAITGVGSIGSGIAATVLYILKKKGAAKAALW

>Streptococcus-pneumoniae_COM92703.1

MKSKRMEFHNKFLIVSAMLAVISWLSLGVVSFPMLAGTLGISTKAAATVVNLISAYSTVTAVISIVGAITGVGSIGSGIAATVLYILKKKGAAKAALW

>Streptococcus-pneumoniae_CIT47543.1

MKSKRMEFHNKFLIVSAMLAVISWLSLGVVSFPMLAGTLGISTKAAATVVNLISAYSTVTAVISIVGAITGVGSIGSGIAATVLYILKKKGAAKAALW

>Streptococcus-pneumoniae_COQ18132.1

MKSKRMEFHNKFLIVSAMLAVISWLSLGVVSFPMLAGTLGISTKAAATVVNLISAYSTVTAVISIVGAITGVGSIGSGIAATVLYILKKKGAAKAALW

>Streptococcus-pneumoniae_CJV90258.1

MKSKRMEFHNKFLIVSAMLAVISWLSLGVVSFPMLAGTLGISTKAAATVVNLISAYSTVTAVISIVGAITGVGSIGSGIAATVLYILKKKGAAKAALW

>Streptococcus-pneumoniae_CJR91250.1

MKSKRMEFHNKFLIVSAMLAVISWLSLGVVSFPMLAGTLGISTKAAATVVNLISAYSTVTAVISIVGAITGVGSIGSGIAATVLYILKKKGAAKAALW

>Streptococcus-pneumoniae_COK05291.1

MKSKRMEFHNKFLIVSAMLAVISWLSLGVVSFPMLAGTLGISTKAAATVVNLISAYSTVTAVISIVGAITGVGSIGSGIAATVLYILKKKGAAKAALW

>Streptococcus-pneumoniae_COB84870.1

MKSKRMEFHNKFLIVSAMLAVISWLSLGVVSFPMLAGTLGISTKAAATVVNLISAYSTVTAVISIVGAITGVGSIGSGIAATVLYILKKKGAAKAALW

>Streptococcus-pneumoniae_CMZ34526.1

MKSKRMEFHNKFLIVSAMLAVISWLSLGVVSFPMLAGTLGISTKAAATVVNLISAYSTVTAVISIVGAITGVGSIGSGIAATVLYILKKKGAAKAALW

>Streptococcus-pneumoniae_CIU79493.1

MKSKRMEFHNKFLIVSAMLAVISWLSLGVVSFPMLAGTLGISTKAAATVVNLISAYSTVTAVISIVGAITGVGSIGSGIAATVLYILKKKGAAKAALW

>Streptococcus-pneumoniae_COG61487.1

MKSKRMEFHNKFLIVSAMLAVISWLSLGVVSFPMLAGTLGISTKAAATVVNLISAYSTVTAVISIVGAITGVGSIGSGIAATVLYILKKKGAAKAALW

>Streptococcus-pneumoniae_CKE18268.1

MKSKRMEFHNKFLIVSAMLAVISWLSLGVVSFPMLAGTLGISTKAAATVVNLISAYSTVTAVISIVGAITGVGSIGSGIAATVLYILKKKGAAKAALW

>Streptococcus-pneumoniae_CJX18841.1

MKSKRMEFHNKFLIVSAMLAVISWLSLGVVSFPMLAGTLGISTKAAATVVNLISAYSTVTAVISIVGAITGVGSIGSGIAATVLYILKKKGAAKAALW

>Streptococcus-pneumoniae_COG77131.1

MKSKRMEFHNKFLIVSAMLAVISWLSLGVVSFPMLAGTLGISTKAAATVVNLISAYSTVTAVISIVGAITGVGSIGSGIAATVLYILKKKGAAKAALW

>Streptococcus-pneumoniae_CGG97664.1

MKSKRMEFHNKFLIVSAMLAVISWLSLGVVSFPMLAGTLGISTKAAATVVNLISAYSTVTAVISIVGAITGVGSIGSGIAATVLYILKKKGAAKAALW

>Streptococcus-pneumoniae_COI85301.1

MKSKRMEFHNKFLIVSAMLAVISWLSLGVVSFPMLAGTLGISTKAAATVVNLISAYSTVTAVISIVGAITGVGSIGSGIAATVLYILKKKGAAKAALW

>Streptococcus-pneumoniae_COE67000.1

MKSKRMEFHNKFLIVSAMLAVISWLSLGVVSFPMLAGTLGISTKAAATVVNLISAYSTVTAVISIVGAITGVGSIGSGIAATVLYILKKKGAAKAALW

>Streptococcus-pneumoniae_CGF12015.1

MKSKRMEFHNKFLIVSAMLAVISWLSLGVVSFPMLAGTLGISTKAAATVVNLISAYSTVTAVISIVGAITGVGSIGSGIAATVLYILKKKGAAKAALW

>Streptococcus-pneumoniae_COS44391.1

MKSKRMEFHNKFLIVSAMLAVISWLSLGVVSFPMLAGTLGISTKAAATVVNLISAYSTVTAVISIVGAITGVGSIGSGIAATVLYILKKKGAAKAALW

>Streptococcus-pneumoniae_CGH03327.1

MKSKRMEFHNKFLIVSAMLAVISWLSLGVVSFPMLAGTLGISTKAAATVVNLISAYSTVTAVISIVGAITGVGSIGSGIAATVLYILKKKGAAKAALW

>Streptococcus-pneumoniae_COS09913.1

MKSKRMEFHNKFLIVSAMLAVISWLSLGVVSFPMLAGTLGISTKAAATVVNLISAYSTVTAVISIVGAITGVGSIGSGIAATVLYILKKKGAAKAALW

>Streptococcus-pneumoniae_CJL53330.1

MKSKRMEFHNKFLIVSAMLAVISWLSLGVVSFPMLAGTLGISTKAAATVVNLISAYSTVTAVISIVGAITGVGSIGSGIAATVLYILKKKGAAKAALW

>Streptococcus-pneumoniae_CKI61990.1

MKSKRMEFHNKFLIVSAMLAVISWLSLGVVSFPMLAGTLGISTKAAATVVNLISAYSTVTAVISIVGAITGVGSIGSGIAATVLYILKKKGAAKAALW

>Streptococcus-pneumoniae_CIR88463.1

MKSKRMEFHNKFLIVSAMLAVISWLSLGVVSFPMLAGTLGISTKAAATVVNLISAYSTVTAVISIVGAITGVGSIGSGIAATVLYILKKKGAAKAALW

>Streptococcus-pneumoniae_COP49976.1

MKSKRMEFHNKFLIVSAMLAVISWLSLGVVSFPMLAGTLGISTKAAATVVNLISAYSTVTAVISIVGAITGVGSIGSGIAATVLYILKKKGAAKAALW

>Streptococcus-pneumoniae_COC73256.1

MKSKRMEFHNKFLIVSAMLAVISWLSLGVVSFPMLAGTLGISTKAAATVVNLISAYSTVTAVISIVGAITGVGSIGSGIAATVLYILKKKGAAKAALW

>Streptococcus-pneumoniae_CJP43794.1

MKSKRMEFHNKFLIVSAMLAVISWLSLGVVSFPMLAGTLGISTKAAATVVNLISAYSTVTAVISIVGAITGVGSIGSGIAATVLYILKKKGAAKAALW

>Streptococcus-pneumoniae_COE85683.1

MKSKRMEFHNKFLIVSAMLAVISWLSLGVVSFPMLAGTLGISTKAAATVVNLISAYSTVTAVISIVGAITGVGSIGSGIAATVLYILKKKGAAKAALW

>Streptococcus-pneumoniae_COP20504.1

MKSKRMEFHNKFLIVSAMLAVISWLSLGVVSFPMLAGTLGISTKAAATVVNLISAYSTVTAVISIVGAITGVGSIGSGIAATVLYILKKKGAAKAALW

>Streptococcus-pneumoniae_CJT90786.1

MKSKRMEFHNKFLIVSAMLAVISWLSLGVVSFPMLAGTLGISTKAAATVVNLISAYSTVTAVISIVGAITGVGSIGSGIAATVLYILKKKGAAKAALW

>Streptococcus-pneumoniae_COO47081.1

MKSKRMEFHNKFLIVSAMLAVISWLSLGVVSFPMLAGTLGISTKAAATVVNLISAYSTVTAVISIVGAITGVGSIGSGIAATVLYILKKKGAAKAALW

>Streptococcus-pneumoniae_CON60580.1

MKSKRMEFHNKFLIVSAMLAVISWLSLGVVSFPMLAGTLGISTKAAATVVNLISAYSTVTAVISIVGAITGVGSIGSGIAATVLYILKKKGAAKAALW

>Streptococcus-pneumoniae_CJX71441.1

MKSKRMEFHNKFLIVSAMLAVISWLSLGVVSFPMLAGTLGISTKAAATVVNLISAYSTVTAVISIVGAITGVGSIGSGIAATVLYILKKKGAAKAALW

>Streptococcus-pneumoniae_COE51025.1

MKSKRMEFHNKFLIVSAMLAVISWLSLGVVSFPMLAGTLGISTKAAATVVNLISAYSTVTAVISIVGAITGVGSIGSGIAATVLYILKKKGAAKAALW

>Streptococcus-pneumoniae_COH65316.1

MKSKRMEFHNKFLIVSAMLAVISWLSLGVVSFPMLAGTLGISTKAAATVVNLISAYSTVTAVISIVGAITGVGSIGSGIAATVLYILKKKGAAKAALW

>Streptococcus-pneumoniae_CIS15772.1

MKSKRMEFHNKFLIVSAMLAVISWLSLGVVSFPMLAGTLGISTKAAATVVNLISAYSTVTAVISIVGAITGVGSIGSGIAATVLYILKKKGAAKAALW

>Streptococcus-pneumoniae_CIV42364.1

MKSKRMEFHNKFLIVSAMLAVISWLSLGVVSFPMLAGTLGISTKAAATVVNLISAYSTVTAVISIVGAITGVGSIGSGIAATVLYILKKKGAAKAALW

>Streptococcus-pneumoniae_CJF70304.1

MKSKRMEFHNKFLIVSAMLAVISWLSLGVVSFPMLAGTLGISTKAAATVVNLISAYSTVTAVISIVGAITGVGSIGSGIAATVLYILKKKGAAKAALW

>Streptococcus-pneumoniae_CKJ56436.1

MKSKRMEFHNKFLIVSAMLAVISWLSLGVVSFPMLAGTLGISTKAAATVVNLISAYSTVTAVISIVGAITGVGSIGSGIAATVLYILKKKGAAKAALW

>Streptococcus-pneumoniae_CGE96363.1

MKSKRMEFHNKFLIVSAMLAVISWLSLGVVSFPMLAGTLGISTKAAATVVNLISAYSTVTAVISIVGAITGVGSIGSGIAATVLYILKKKGAAKAALW

>Streptococcus-pneumoniae_CIZ44806.1

MKSKRMEFHNKFLIVSAMLAVISWLSLGVVSFPMLAGTLGISTKAAATVVNLISAYSTVTAVISIVGAITGVGSIGSGIAATVLYILKKKGAAKAALW

>Streptococcus-pneumoniae_COO89981.1

MKSKRMEFHNKFLIVSAMLAVISWLSLGVVSFPMLAGTLGISTKAAATVVNLISAYSTVTAVISIVGAITGVGSIGSGIAATVLYILKKKGAAKAALW

>Streptococcus-pneumoniae_CIU18971.1

MKSKRMEFHNKFLIVSAMLAVISWLSLGVVSFPMLAGTLGISTKAAATVVNLISAYSTVTAVISIVGAITGVGSIGSGIAATVLYILKKKGAAKAALW

>Streptococcus-pneumoniae_COB36331.1

MKSKRMEFHNKFLIVSAMLAVISWLSLGVVSFPMLAGTLGISTKAAATVVNLISAYSTVTAVISIVGAITGVGSIGSGIAATVLYILKKKGAAKAALW

>Streptococcus-pneumoniae_COO64450.1

MKSKRMEFHNKFLIVSAMLAVISWLSLGVVSFPMLAGTLGISTKAAATVVNLISAYSTVTAVISIVGAITGVGSIGSGIAATVLYILKKKGAAKAALW

>Streptococcus-pneumoniae_CON16515.1

MKSKRMEFHNKFLIVSAMLAVISWLSLGVVSFPMLAGTLGISTKAAATVVNLISAYSTVTAVISIVGAITGVGSIGSGIAATVLYILKKKGAAKAALW

>Streptococcus-pneumoniae_COO96037.1

MKSKRMEFHNKFLIVSAMLAVISWLSLGVVSFPMLAGTLGISTKAAATVVNLISAYSTVTAVISIVGAITGVGSIGSGIAATVLYILKKKGAAKAALW

>Streptococcus-pneumoniae_CIZ97959.1

MKSKRMEFHNKFLIVSAMLAVISWLSLGVVSFPMLAGTLGISTKAAATVVNLISAYSTVTAVISIVGAITGVGSIGSGIAATVLYILKKKGAAKAALW

>Streptococcus-pneumoniae_CRI60760.1

MKSKRMEFHNKFLIVSAMLAVISWLSLGVVSFPMLAGTLGISTKAAATVVNLISAYSTVTAVISIVGAITGVGSIGSGIAATVLYILKKKGAAKAALW

>Streptococcus-pneumoniae_CGG05167.1

MKSKRMEFHNKFLIVSAMLAVISWLSLGVVSFPMLAGTLGISTKAAATVVNLISAYSTVTAVISIVGAITGVGSIGSGIAATVLYILKKKGAAKAALW

>Streptococcus-pneumoniae_CGF13661.1

MKSKRMEFHNKFLIVSAMLAVISWLSLGVVSFPMLAGTLGISTKAAATVVNLISAYSTVTAVISIVGAITGVGSIGSGIAATVLYILKKKGAAKAALW

>Streptococcus-pneumoniae_CGG80301.1

MKSKRMEFHNKFLIVSAMLAVISWLSLGVVSFPMLAGTLGISTKAAATVVNLISAYSTVTAVISIVGAITGVGSIGSGIAATVLYILKKKGAAKAALW

>Streptococcus-pneumoniae_CGE84123.1

MKSKRMEFHNKFLIVSAMLAVISWLSLGVVSFPMLAGTLGISTKAAATVVNLISAYSTVTAVISIVGAITGVGSIGSGIAATVLYILKKKGAAKAALW

>Streptococcus-pneumoniae_CGF15766.1

MKSKRMEFHNKFLIVSAMLAVISWLSLGVVSFPMLAGTLGISTKAAATVVNLISAYSTVTAVISIVGAITGVGSIGSGIAATVLYILKKKGAAKAALW

>Streptococcus-pneumoniae_CGF51226.1

MKSKRMEFHNKFLIVSAMLAVISWLSLGVVSFPMLAGTLGISTKAAATVVNLISAYSTVTAVISIVGAITGVGSIGSGIAATVLYILKKKGAAKAALW

>Streptococcus-pneumoniae_CGE95654.1

MKSKRMEFHNKFLIVSAMLAVISWLSLGVVSFPMLAGTLGISTKAAATVVNLISAYSTVTAVISIVGAITGVGSIGSGIAATVLYILKKKGAAKAALW

>Streptococcus-pneumoniae_CGF35731.1

MKSKRMEFHNKFLIVSAMLAVISWLSLGVVSFPMLAGTLGISTKAAATVVNLISAYSTVTAVISIVGAITGVGSIGSGIAATVLYILKKKGAAKAALW

>Streptococcus-pneumoniae_CIW24930.1

MKSKRMEFHNKFLIVSAMLAVISWLSLGVVSFPMLAGTLGISTKAAATVVNLISAYSTVTAVISIVGAITGVGSIGSGIAATVLYILKKKGAAKAALW

>Streptococcus-pneumoniae_CIV98464.1

MKSKRMEFHNKFLIVSAMLAVISWLSLGVVSFPMLAGTLGISTKAAATVVNLISAYSTVTAVISIVGAITGVGSIGSGIAATVLYILKKKGAAKAALW

>Streptococcus-pneumoniae_CIW03142.1

MKSKRMEFHNKFLIVSAMLAVISWLSLGVVSFPMLAGTLGISTKAAATVVNLISAYSTVTAVISIVGAITGVGSIGSGIAATVLYILKKKGAAKAALW

>Streptococcus-pneumoniae_CIW92864.1

MKSKRMEFHNKFLIVSAMLAVISWLSLGVVSFPMLAGTLGISTKAAATVVNLISAYSTVTAVISIVGAITGVGSIGSGIAATVLYILKKKGAAKAALW

>Streptococcus-pneumoniae_CIW06882.1

MKSKRMEFHNKFLIVSAMLAVISWLSLGVVSFPMLAGTLGISTKAAATVVNLISAYSTVTAVISIVGAITGVGSIGSGIAATVLYILKKKGAAKAALW

>Streptococcus-pneumoniae_CIV38572.1

MKSKRMEFHNKFLIVSAMLAVISWLSLGVVSFPMLAGTLGISTKAAATVVNLISAYSTVTAVISIVGAITGVGSIGSGIAATVLYILKKKGAAKAALW

>Streptococcus-pneumoniae_CIO58844.1

MKSKRMEFHNKFLIVSAMLAVISWLSLGVVSFPMLAGTLGISTKAAATVVNLISAYSTVTAVISIVGAITGVGSIGSGIAATVLYILKKKGAAKAALW

>Streptococcus-pneumoniae_CIU11406.1

MKSKRMEFHNKFLIVSAMLAVISWLSLGVVSFPMLAGTLGISTKAAATVVNLISAYSTVTAVISIVGAITGVGSIGSGIAATVLYILKKKGAAKAALW

>Streptococcus-pneumoniae_CIV27646.1

MKSKRMEFHNKFLIVSAMLAVISWLSLGVVSFPMLAGTLGISTKAAATVVNLISAYSTVTAVISIVGAITGVGSIGSGIAATVLYILKKKGAAKAALW

>Streptococcus-pneumoniae_CIS78580.1

MKSKRMEFHNKFLIVSAMLAVISWLSLGVVSFPMLAGTLGISTKAAATVVNLISAYSTVTAVISIVGAITGVGSIGSGIAATVLYILKKKGAAKAALW

>Streptococcus-pneumoniae_CIU97404.1

MKSKRMEFHNKFLIVSAMLAVISWLSLGVVSFPMLAGTLGISTKAAATVVNLISAYSTVTAVISIVGAITGVGSIGSGIAATVLYILKKKGAAKAALW

>Streptococcus-pneumoniae_CIO64333.1

MKSKRMEFHNKFLIVSAMLAVISWLSLGVVSFPMLAGTLGISTKAAATVVNLISAYSTVTAVISIVGAITGVGSIGSGIAATVLYILKKKGAAKAALW

>Streptococcus-pneumoniae_CIU94382.1

MKSKRMEFHNKFLIVSAMLAVISWLSLGVVSFPMLAGTLGISTKAAATVVNLISAYSTVTAVISIVGAITGVGSIGSGIAATVLYILKKKGAAKAALW

>Streptococcus-pneumoniae_CIO90462.1

MKSKRMEFHNKFLIVSAMLAVISWLSLGVVSFPMLAGTLGISTKAAATVVNLISAYSTVTAVISIVGAITGVGSIGSGIAATVLYILKKKGAAKAALW

>Streptococcus-pneumoniae_CIR66944.1

MKSKRMEFHNKFLIVSAMLAVISWLSLGVVSFPMLAGTLGISTKAAATVVNLISAYSTVTAVISIVGAITGVGSIGSGIAATVLYILKKKGAAKAALW

>Streptococcus-pneumoniae_CIN97819.1

MKSKRMEFHNKFLIVSAMLAVISWLSLGVVSFPMLAGTLGISTKAAATVVNLISAYSTVTAVISIVGAITGVGSIGSGIAATVLYILKKKGAAKAALW

>Streptococcus-pneumoniae_CIW91742.1

MKSKRMEFHNKFLIVSAMLAVISWLSLGVVSFPMLAGTLGISTKAAATVVNLISAYSTVTAVISIVGAITGVGSIGSGIAATVLYILKKKGAAKAALW

>Streptococcus-pneumoniae_CIO14343.1

MKSKRMEFHNKFLIVSAMLAVISWLSLGVVSFPMLAGTLGISTKAAATVVNLISAYSTVTAVISIVGAITGVGSIGSGIAATVLYILKKKGAAKAALW

>Streptococcus-pneumoniae_CIR66848.1

MKSKRMEFHNKFLIVSAMLAVISWLSLGVVSFPMLAGTLGISTKAAATVVNLISAYSTVTAVISIVGAITGVGSIGSGIAATVLYILKKKGAAKAALW

>Streptococcus-pneumoniae_CIS62216.1

MKSKRMEFHNKFLIVSAMLAVISWLSLGVVSFPMLAGTLGISTKAAATVVNLISAYSTVTAVISIVGAITGVGSIGSGIAATVLYILKKKGAAKAALW

>Streptococcus-pneumoniae_CIX99884.1

MKSKRMEFHNKFLIVSAMLAVISWLSLGVVSFPMLAGTLGISTKAAATVVNLISAYSTVTAVISIVGAITGVGSIGSGIAATVLYILKKKGAAKAALW

>Streptococcus-pneumoniae_CIU17471.1

MKSKRMEFHNKFLIVSAMLAVISWLSLGVVSFPMLAGTLGISTKAAATVVNLISAYSTVTAVISIVGAITGVGSIGSGIAATVLYILKKKGAAKAALW

>Streptococcus-pneumoniae_CIV65996.1

MKSKRMEFHNKFLIVSAMLAVISWLSLGVVSFPMLAGTLGISTKAAATVVNLISAYSTVTAVISIVGAITGVGSIGSGIAATVLYILKKKGAAKAALW

>Streptococcus-pneumoniae_CIS64206.1

MKSKRMEFHNKFLIVSAMLAVISWLSLGVVSFPMLAGTLGISTKAAATVVNLISAYSTVTAVISIVGAITGVGSIGSGIAATVLYILKKKGAAKAALW

>Streptococcus-pneumoniae_CIQ45441.1

MKSKRMEFHNKFLIVSAMLAVISWLSLGVVSFPMLAGTLGISTKAAATVVNLISAYSTVTAVISIVGAITGVGSIGSGIAATVLYILKKKGAAKAALW

>Streptococcus-pneumoniae_CIP04776.1

MKSKRMEFHNKFLIVSAMLAVISWLSLGVVSFPMLAGTLGISTKAAATVVNLISAYSTVTAVISIVGAITGVGSIGSGIAATVLYILKKKGAAKAALW

>Streptococcus-pneumoniae_CIY83717.1

MKSKRMEFHNKFLIVSAMLAVISWLSLGVVSFPMLAGTLGISTKAAATVVNLISAYSTVTAVISIVGAITGVGSIGSGIAATVLYILKKKGAAKAALW

>Streptococcus-pneumoniae_CIP78316.1

MKSKRMEFHNKFLIVSAMLAVISWLSLGVVSFPMLAGTLGISTKAAATVVNLISAYSTVTAVISIVGAITGVGSIGSGIAATVLYILKKKGAAKAALW

>Streptococcus-pneumoniae_CIN57434.1

MKSKRMEFHNKFLIVSAMLAVISWLSLGVVSFPMLAGTLGISTKAAATVVNLISAYSTVTAVISIVGAITGVGSIGSGIAATVLYILKKKGAAKAALW

>Streptococcus-pneumoniae_CIX41607.1

MKSKRMEFHNKFLIVSAMLAVISWLSLGVVSFPMLAGTLGISTKAAATVVNLISAYSTVTAVISIVGAITGVGSIGSGIAATVLYILKKKGAAKAALW

>Streptococcus-pneumoniae_CIR45824.1

MKSKRMEFHNKFLIVSAMLAVISWLSLGVVSFPMLAGTLGISTKAAATVVNLISAYSTVTAVISIVGAITGVGSIGSGIAATVLYILKKKGAAKAALW

>Streptococcus-pneumoniae_CIS07722.1

MKSKRMEFHNKFLIVSAMLAVISWLSLGVVSFPMLAGTLGISTKAAATVVNLISAYSTVTAVISIVGAITGVGSIGSGIAATVLYILKKKGAAKAALW

>Streptococcus-pneumoniae_CIP64521.1

MKSKRMEFHNKFLIVSAMLAVISWLSLGVVSFPMLAGTLGISTKAAATVVNLISAYSTVTAVISIVGAITGVGSIGSGIAATVLYILKKKGAAKAALW

>Streptococcus-pneumoniae_CIT74737.1

MKSKRMEFHNKFLIVSAMLAVISWLSLGVVSFPMLAGTLGISTKAAATVVNLISAYSTVTAVISIVGAITGVGSIGSGIAATVLYILKKKGAAKAALW

>Streptococcus-pneumoniae_CIT71269.1

MKSKRMEFHNKFLIVSAMLAVISWLSLGVVSFPMLAGTLGISTKAAATVVNLISAYSTVTAVISIVGAITGVGSIGSGIAATVLYILKKKGAAKAALW

>Streptococcus-pneumoniae_CIZ60987.1

MKSKRMEFHNKFLIVSAMLAVISWLSLGVVSFPMLAGTLGISTKAAATVVNLISAYSTVTAVISIVGAITGVGSIGSGIAATVLYILKKKGAAKAALW

>Streptococcus-pneumoniae_CIO48456.1

MKSKRMEFHNKFLIVSAMLAVISWLSLGVVSFPMLAGTLGISTKAAATVVNLISAYSTVTAVISIVGAITGVGSIGSGIAATVLYILKKKGAAKAALW

>Streptococcus-pneumoniae_CIW53115.1

MKSKRMEFHNKFLIVSAMLAVISWLSLGVVSFPMLAGTLGISTKAAATVVNLISAYSTVTAVISIVGAITGVGSIGSGIAATVLYILKKKGAAKAALW

>Streptococcus-pneumoniae_CIY76277.1

MKSKRMEFHNKFLIVSAMLAVISWLSLGVVSFPMLAGTLGISTKAAATVVNLISAYSTVTAVISIVGAITGVGSIGSGIAATVLYILKKKGAAKAALW

>Streptococcus-pneumoniae_CIY94206.1

MKSKRMEFHNKFLIVSAMLAVISWLSLGVVSFPMLAGTLGISTKAAATVVNLISAYSTVTAVISIVGAITGVGSIGSGIAATVLYILKKKGAAKAALW

>Streptococcus-pneumoniae_CIU58890.1

MKSKRMEFHNKFLIVSAMLAVISWLSLGVVSFPMLAGTLGISTKAAATVVNLISAYSTVTAVISIVGAITGVGSIGSGIAATVLYILKKKGAAKAALW

>Streptococcus-pneumoniae_CIR36018.1

MKSKRMEFHNKFLIVSAMLAVISWLSLGVVSFPMLAGTLGISTKAAATVVNLISAYSTVTAVISIVGAITGVGSIGSGIAATVLYILKKKGAAKAALW

>Streptococcus-pneumoniae_CIR33158.1

MKSKRMEFHNKFLIVSAMLAVISWLSLGVVSFPMLAGTLGISTKAAATVVNLISAYSTVTAVISIVGAITGVGSIGSGIAATVLYILKKKGAAKAALW

>Streptococcus-pneumoniae_CIX30544.1

MKSKRMEFHNKFLIVSAMLAVISWLSLGVVSFPMLAGTLGISTKAAATVVNLISAYSTVTAVISIVGAITGVGSIGSGIAATVLYILKKKGAAKAALW

>Streptococcus-pneumoniae_CIW93627.1

MKSKRMEFHNKFLIVSAMLAVISWLSLGVVSFPMLAGTLGISTKAAATVVNLISAYSTVTAVISIVGAITGVGSIGSGIAATVLYILKKKGAAKAALW

>Streptococcus-pneumoniae_CIX23347.1

MKSKRMEFHNKFLIVSAMLAVISWLSLGVVSFPMLAGTLGISTKAAATVVNLISAYSTVTAVISIVGAITGVGSIGSGIAATVLYILKKKGAAKAALW

>Streptococcus-pneumoniae_CIW34313.1

MKSKRMEFHNKFLIVSAMLAVISWLSLGVVSFPMLAGTLGISTKAAATVVNLISAYSTVTAVISIVGAITGVGSIGSGIAATVLYILKKKGAAKAALW

>Streptococcus-pneumoniae_CIW03821.1

MKSKRMEFHNKFLIVSAMLAVISWLSLGVVSFPMLAGTLGISTKAAATVVNLISAYSTVTAVISIVGAITGVGSIGSGIAATVLYILKKKGAAKAALW

>Streptococcus-pneumoniae_CIW95869.1

MKSKRMEFHNKFLIVSAMLAVISWLSLGVVSFPMLAGTLGISTKAAATVVNLISAYSTVTAVISIVGAITGVGSIGSGIAATVLYILKKKGAAKAALW

>Streptococcus-pneumoniae_CIZ62265.1

MKSKRMEFHNKFLIVSAMLAVISWLSLGVVSFPMLAGTLGISTKAAATVVNLISAYSTVTAVISIVGAITGVGSIGSGIAATVLYILKKKGAAKAALW

>Streptococcus-pneumoniae_CIS38497.1

MKSKRMEFHNKFLIVSAMLAVISWLSLGVVSFPMLAGTLGISTKAAATVVNLISAYSTVTAVISIVGAITGVGSIGSGIAATVLYILKKKGAAKAALW

>Streptococcus-pneumoniae_CIO63504.1

MKSKRMEFHNKFLIVSAMLAVISWLSLGVVSFPMLAGTLGISTKAAATVVNLISAYSTVTAVISIVGAITGVGSIGSGIAATVLYILKKKGAAKAALW

>Streptococcus-pneumoniae_CIR99990.1

MKSKRMEFHNKFLIVSAMLAVISWLSLGVVSFPMLAGTLGISTKAAATVVNLISAYSTVTAVISIVGAITGVGSIGSGIAATVLYILKKKGAAKAALW

>Streptococcus-pneumoniae_CIU15866.1

MKSKRMEFHNKFLIVSAMLAVISWLSLGVVSFPMLAGTLGISTKAAATVVNLISAYSTVTAVISIVGAITGVGSIGSGIAATVLYILKKKGAAKAALW

>Streptococcus-pneumoniae_CIQ70181.1

MKSKRMEFHNKFLIVSAMLAVISWLSLGVVSFPMLAGTLGISTKAAATVVNLISAYSTVTAVISIVGAITGVGSIGSGIAATVLYILKKKGAAKAALW

>Streptococcus-pneumoniae_CIP59882.1

MKSKRMEFHNKFLIVSAMLAVISWLSLGVVSFPMLAGTLGISTKAAATVVNLISAYSTVTAVISIVGAITGVGSIGSGIAATVLYILKKKGAAKAALW

>Streptococcus-pneumoniae_CIP30468.1

MKSKRMEFHNKFLIVSAMLAVISWLSLGVVSFPMLAGTLGISTKAAATVVNLISAYSTVTAVISIVGAITGVGSIGSGIAATVLYILKKKGAAKAALW

>Streptococcus-pneumoniae_CIV43899.1

MKSKRMEFHNKFLIVSAMLAVISWLSLGVVSFPMLAGTLGISTKAAATVVNLISAYSTVTAVISIVGAITGVGSIGSGIAATVLYILKKKGAAKAALW

>Streptococcus-pneumoniae_CIP63614.1

MKSKRMEFHNKFLIVSAMLAVISWLSLGVVSFPMLAGTLGISTKAAATVVNLISAYSTVTAVISIVGAITGVGSIGSGIAATVLYILKKKGAAKAALW

>Streptococcus-pneumoniae_CIP43324.1

MKSKRMEFHNKFLIVSAMLAVISWLSLGVVSFPMLAGTLGISTKAAATVVNLISAYSTVTAVISIVGAITGVGSIGSGIAATVLYILKKKGAAKAALW

>Streptococcus-pneumoniae_CIR67594.1

MKSKRMEFHNKFLIVSAMLAVISWLSLGVVSFPMLAGTLGISTKAAATVVNLISAYSTVTAVISIVGAITGVGSIGSGIAATVLYILKKKGAAKAALW

>Streptococcus-pneumoniae_CIR03820.1

MKSKRMEFHNKFLIVSAMLAVISWLSLGVVSFPMLAGTLGISTKAAATVVNLISAYSTVTAVISIVGAITGVGSIGSGIAATVLYILKKKGAAKAALW

>Streptococcus-pneumoniae_CIZ89192.1

MKSKRMEFHNKFLIVSAMLAVISWLSLGVVSFPMLAGTLGISTKAAATVVNLISAYSTVTAVISIVGAITGVGSIGSGIAATVLYILKKKGAAKAALW

>Streptococcus-pneumoniae_CIX01147.1

MKSKRMEFHNKFLIVSAMLAVISWLSLGVVSFPMLAGTLGISTKAAATVVNLISAYSTVTAVISIVGAITGVGSIGSGIAATVLYILKKKGAAKAALW

>Streptococcus-pneumoniae_CIP96776.1

MKSKRMEFHNKFLIVSAMLAVISWLSLGVVSFPMLAGTLGISTKAAATVVNLISAYSTVTAVISIVGAITGVGSIGSGIAATVLYILKKKGAAKAALW

>Streptococcus-pneumoniae_CIS47653.1

MKSKRMEFHNKFLIVSAMLAVISWLSLGVVSFPMLAGTLGISTKAAATVVNLISAYSTVTAVISIVGAITGVGSIGSGIAATVLYILKKKGAAKAALW

>Streptococcus-pneumoniae_CIS96785.1

MKSKRMEFHNKFLIVSAMLAVISWLSLGVVSFPMLAGTLGISTKAAATVVNLISAYSTVTAVISIVGAITGVGSIGSGIAATVLYILKKKGAAKAALW

>Streptococcus-pneumoniae_CIZ49176.1

MKSKRMEFHNKFLIVSAMLAVISWLSLGVVSFPMLAGTLGISTKAAATVVNLISAYSTVTAVISIVGAITGVGSIGSGIAATVLYILKKKGAAKAALW

>Streptococcus-pneumoniae_CIS56632.1

MKSKRMEFHNKFLIVSAMLAVISWLSLGVVSFPMLAGTLGISTKAAATVVNLISAYSTVTAVISIVGAITGVGSIGSGIAATVLYILKKKGAAKAALW

>Streptococcus-pneumoniae_CIW67210.1

MKSKRMEFHNKFLIVSAMLAVISWLSLGVVSFPMLAGTLGISTKAAATVVNLISAYSTVTAVISIVGAITGVGSIGSGIAATVLYILKKKGAAKAALW

>Streptococcus-pneumoniae_CIV51979.1

MKSKRMEFHNKFLIVSAMLAVISWLSLGVVSFPMLAGTLGISTKAAATVVNLISAYSTVTAVISIVGAITGVGSIGSGIAATVLYILKKKGAAKAALW

>Streptococcus-pneumoniae_CIU90994.1

MKSKRMEFHNKFLIVSAMLAVISWLSLGVVSFPMLAGTLGISTKAAATVVNLISAYSTVTAVISIVGAITGVGSIGSGIAATVLYILKKKGAAKAALW

>Streptococcus-pneumoniae_CIO12039.1

MKSKRMEFHNKFLIVSAMLAVISWLSLGVVSFPMLAGTLGISTKAAATVVNLISAYSTVTAVISIVGAITGVGSIGSGIAATVLYILKKKGAAKAALW

>Streptococcus-pneumoniae_CIW07450.1

MKSKRMEFHNKFLIVSAMLAVISWLSLGVVSFPMLAGTLGISTKAAATVVNLISAYSTVTAVISIVGAITGVGSIGSGIAATVLYILKKKGAAKAALW

>Streptococcus-pneumoniae_CIQ32546.1

MKSKRMEFHNKFLIVSAMLAVISWLSLGVVSFPMLAGTLGISTKAAATVVNLISAYSTVTAVISIVGAITGVGSIGSGIAATVLYILKKKGAAKAALW

>Streptococcus-pneumoniae_CIZ87098.1

MKSKRMEFHNKFLIVSAMLAVISWLSLGVVSFPMLAGTLGISTKAAATVVNLISAYSTVTAVISIVGAITGVGSIGSGIAATVLYILKKKGAAKAALW

>Streptococcus-pneumoniae_CIY05403.1

MKSKRMEFHNKFLIVSAMLAVISWLSLGVVSFPMLAGTLGISTKAAATVVNLISAYSTVTAVISIVGAITGVGSIGSGIAATVLYILKKKGAAKAALW

>Streptococcus-pneumoniae_CIX61240.1

MKSKRMEFHNKFLIVSAMLAVISWLSLGVVSFPMLAGTLGISTKAAATVVNLISAYSTVTAVISIVGAITGVGSIGSGIAATVLYILKKKGAAKAALW

>Streptococcus-pneumoniae_CIP85427.1

MKSKRMEFHNKFLIVSAMLAVISWLSLGVVSFPMLAGTLGISTKAAATVVNLISAYSTVTAVISIVGAITGVGSIGSGIAATVLYILKKKGAAKAALW

>Streptococcus-pneumoniae_CIR40143.1

MKSKRMEFHNKFLIVSAMLAVISWLSLGVVSFPMLAGTLGISTKAAATVVNLISAYSTVTAVISIVGAITGVGSIGSGIAATVLYILKKKGAAKAALW

>Streptococcus-pneumoniae_CIO60737.1

MKSKRMEFHNKFLIVSAMLAVISWLSLGVVSFPMLAGTLGISTKAAATVVNLISAYSTVTAVISIVGAITGVGSIGSGIAATVLYILKKKGAAKAALW

>Streptococcus-pneumoniae_CIU79224.1

MKSKRMEFHNKFLIVSAMLAVISWLSLGVVSFPMLAGTLGISTKAAATVVNLISAYSTVTAVISIVGAITGVGSIGSGIAATVLYILKKKGAAKAALW

>Streptococcus-pneumoniae_CIW41044.1

MKSKRMEFHNKFLIVSAMLAVISWLSLGVVSFPMLAGTLGISTKAAATVVNLISAYSTVTAVISIVGAITGVGSIGSGIAATVLYILKKKGAAKAALW

>Streptococcus-pneumoniae_CIW10716.1

MKSKRMEFHNKFLIVSAMLAVISWLSLGVVSFPMLAGTLGISTKAAATVVNLISAYSTVTAVISIVGAITGVGSIGSGIAATVLYILKKKGAAKAALW

>Streptococcus-pneumoniae_CIT61402.1

MKSKRMEFHNKFLIVSAMLAVISWLSLGVVSFPMLAGTLGISTKAAATVVNLISAYSTVTAVISIVGAITGVGSIGSGIAATVLYILKKKGAAKAALW

>Streptococcus-pneumoniae_CIP81088.1

MKSKRMEFHNKFLIVSAMLAVISWLSLGVVSFPMLAGTLGISTKAAATVVNLISAYSTVTAVISIVGAITGVGSIGSGIAATVLYILKKKGAAKAALW

>Streptococcus-pneumoniae_CIP23691.1

MKSKRMEFHNKFLIVSAMLAVISWLSLGVVSFPMLAGTLGISTKAAATVVNLISAYSTVTAVISIVGAITGVGSIGSGIAATVLYILKKKGAAKAALW

>Streptococcus-pneumoniae_CIV73727.1

MKSKRMEFHNKFLIVSAMLAVISWLSLGVVSFPMLAGTLGISTKAAATVVNLISAYSTVTAVISIVGAITGVGSIGSGIAATVLYILKKKGAAKAALW

>Streptococcus-pneumoniae_CIQ75628.1

MKSKRMEFHNKFLIVSAMLAVISWLSLGVVSFPMLAGTLGISTKAAATVVNLISAYSTVTAVISIVGAITGVGSIGSGIAATVLYILKKKGAAKAALW

>Streptococcus-pneumoniae_CIR98351.1

MKSKRMEFHNKFLIVSAMLAVISWLSLGVVSFPMLAGTLGISTKAAATVVNLISAYSTVTAVISIVGAITGVGSIGSGIAATVLYILKKKGAAKAALW

>Streptococcus-pneumoniae_CIW42664.1

MKSKRMEFHNKFLIVSAMLAVISWLSLGVVSFPMLAGTLGISTKAAATVVNLISAYSTVTAVISIVGAITGVGSIGSGIAATVLYILKKKGAAKAALW

>Streptococcus-pneumoniae_CIU61957.1

MKSKRMEFHNKFLIVSAMLAVISWLSLGVVSFPMLAGTLGISTKAAATVVNLISAYSTVTAVISIVGAITGVGSIGSGIAATVLYILKKKGAAKAALW

>Streptococcus-pneumoniae_CIO55951.1

MKSKRMEFHNKFLIVSAMLAVISWLSLGVVSFPMLAGTLGISTKAAATVVNLISAYSTVTAVISIVGAITGVGSIGSGIAATVLYILKKKGAAKAALW

>Streptococcus-pneumoniae_CIY94558.1

MKSKRMEFHNKFLIVSAMLAVISWLSLGVVSFPMLAGTLGISTKAAATVVNLISAYSTVTAVISIVGAITGVGSIGSGIAATVLYILKKKGAAKAALW

>Streptococcus-pneumoniae_CIZ88879.1

MKSKRMEFHNKFLIVSAMLAVISWLSLGVVSFPMLAGTLGISTKAAATVVNLISAYSTVTAVISIVGAITGVGSIGSGIAATVLYILKKKGAAKAALW

>Streptococcus-pneumoniae_CIQ20255.1

MKSKRMEFHNKFLIVSAMLAVISWLSLGVVSFPMLAGTLGISTKAAATVVNLISAYSTVTAVISIVGAITGVGSIGSGIAATVLYILKKKGAAKAALW

>Streptococcus-pneumoniae_CIQ54247.1

MKSKRMEFHNKFLIVSAMLAVISWLSLGVVSFPMLAGTLGISTKAAATVVNLISAYSTVTAVISIVGAITGVGSIGSGIAATVLYILKKKGAAKAALW

>Streptococcus-pneumoniae_CIV62750.1

MKSKRMEFHNKFLIVSAMLAVISWLSLGVVSFPMLAGTLGISTKAAATVVNLISAYSTVTAVISIVGAITGVGSIGSGIAATVLYILKKKGAAKAALW

>Streptococcus-pneumoniae_CIV34095.1

MKSKRMEFHNKFLIVSAMLAVISWLSLGVVSFPMLAGTLGISTKAAATVVNLISAYSTVTAVISIVGAITGVGSIGSGIAATVLYILKKKGAAKAALW

>Streptococcus-pneumoniae_CIQ73682.1

MKSKRMEFHNKFLIVSAMLAVISWLSLGVVSFPMLAGTLGISTKAAATVVNLISAYSTVTAVISIVGAITGVGSIGSGIAATVLYILKKKGAAKAALW

>Streptococcus-pneumoniae_CIQ39261.1

MKSKRMEFHNKFLIVSAMLAVISWLSLGVVSFPMLAGTLGISTKAAATVVNLISAYSTVTAVISIVGAITGVGSIGSGIAATVLYILKKKGAAKAALW

>Streptococcus-pneumoniae_CIY28727.1

MKSKRMEFHNKFLIVSAMLAVISWLSLGVVSFPMLAGTLGISTKAAATVVNLISAYSTVTAVISIVGAITGVGSIGSGIAATVLYILKKKGAAKAALW

>Streptococcus-pneumoniae_CIZ27996.1

MKSKRMEFHNKFLIVSAMLAVISWLSLGVVSFPMLAGTLGISTKAAATVVNLISAYSTVTAVISIVGAITGVGSIGSGIAATVLYILKKKGAAKAALW

>Streptococcus-pneumoniae_CIX65134.1

MKSKRMEFHNKFLIVSAMLAVISWLSLGVVSFPMLAGTLGISTKAAATVVNLISAYSTVTAVISIVGAITGVGSIGSGIAATVLYILKKKGAAKAALW

>Streptococcus-pneumoniae_CIT75941.1

MKSKRMEFHNKFLIVSAMLAVISWLSLGVVSFPMLAGTLGISTKAAATVVNLISAYSTVTAVISIVGAITGVGSIGSGIAATVLYILKKKGAAKAALW

>Streptococcus-pneumoniae_CIU98662.1

MKSKRMEFHNKFLIVSAMLAVISWLSLGVVSFPMLAGTLGISTKAAATVVNLISAYSTVTAVISIVGAITGVGSIGSGIAATVLYILKKKGAAKAALW

>Streptococcus-pneumoniae_CIY19577.1

MKSKRMEFHNKFLIVSAMLAVISWLSLGVVSFPMLAGTLGISTKAAATVVNLISAYSTVTAVISIVGAITGVGSIGSGIAATVLYILKKKGAAKAALW

>Streptococcus-pneumoniae_CIP40627.1

MKSKRMEFHNKFLIVSAMLAVISWLSLGVVSFPMLAGTLGISTKAAATVVNLISAYSTVTAVISIVGAITGVGSIGSGIAATVLYILKKKGAAKAALW

>Streptococcus-pneumoniae_CIU71187.1

MKSKRMEFHNKFLIVSAMLAVISWLSLGVVSFPMLAGTLGISTKAAATVVNLISAYSTVTAVISIVGAITGVGSIGSGIAATVLYILKKKGAAKAALW

>Streptococcus-pneumoniae_CIX26102.1

MKSKRMEFHNKFLIVSAMLAVISWLSLGVVSFPMLAGTLGISTKAAATVVNLISAYSTVTAVISIVGAITGVGSIGSGIAATVLYILKKKGAAKAALW

>Streptococcus-pneumoniae_CIY35132.1

MKSKRMEFHNKFLIVSAMLAVISWLSLGVVSFPMLAGTLGISTKAAATVVNLISAYSTVTAVISIVGAITGVGSIGSGIAATVLYILKKKGAAKAALW

>Streptococcus-pneumoniae_CIU01329.1

MKSKRMEFHNKFLIVSAMLAVISWLSLGVVSFPMLAGTLGISTKAAATVVNLISAYSTVTAVISIVGAITGVGSIGSGIAATVLYILKKKGAAKAALW

>Streptococcus-pneumoniae_CIN97058.1

MKSKRMEFHNKFLIVSAMLAVISWLSLGVVSFPMLAGTLGISTKAAATVVNLISAYSTVTAVISIVGAITGVGSIGSGIAATVLYILKKKGAAKAALW

>Streptococcus-pneumoniae_CIP52079.1

MKSKRMEFHNKFLIVSAMLAVISWLSLGVVSFPMLAGTLGISTKAAATVVNLISAYSTVTAVISIVGAITGVGSIGSGIAATVLYILKKKGAAKAALW

>Streptococcus-pneumoniae_CJA03923.1

MKSKRMEFHNKFLIVSAMLAVISWLSLGVVSFPMLAGTLGISTKAAATVVNLISAYSTVTAVISIVGAITGVGSIGSGIAATVLYILKKKGAAKAALW

>Streptococcus-pneumoniae_CIR47327.1

MKSKRMEFHNKFLIVSAMLAVISWLSLGVVSFPMLAGTLGISTKAAATVVNLISAYSTVTAVISIVGAITGVGSIGSGIAATVLYILKKKGAAKAALW

>Streptococcus-pneumoniae_CIQ53188.1

MKSKRMEFHNKFLIVSAMLAVISWLSLGVVSFPMLAGTLGISTKAAATVVNLISAYSTVTAVISIVGAITGVGSIGSGIAATVLYILKKKGAAKAALW

>Streptococcus-pneumoniae_CIU23580.1

MKSKRMEFHNKFLIVSAMLAVISWLSLGVVSFPMLAGTLGISTKAAATVVNLISAYSTVTAVISIVGAITGVGSIGSGIAATVLYILKKKGAAKAALW

>Streptococcus-pneumoniae_CIR37583.1

MKSKRMEFHNKFLIVSAMLAVISWLSLGVVSFPMLAGTLGISTKAAATVVNLISAYSTVTAVISIVGAITGVGSIGSGIAATVLYILKKKGAAKAALW

>Streptococcus-pneumoniae_CIS78568.1

MKSKRMEFHNKFLIVSAMLAVISWLSLGVVSFPMLAGTLGISTKAAATVVNLISAYSTVTAVISIVGAITGVGSIGSGIAATVLYILKKKGAAKAALW

>Streptococcus-pneumoniae_CIU39378.1

MKSKRMEFHNKFLIVSAMLAVISWLSLGVVSFPMLAGTLGISTKAAATVVNLISAYSTVTAVISIVGAITGVGSIGSGIAATVLYILKKKGAAKAALW

>Streptococcus-pneumoniae_CIX03532.1

MKSKRMEFHNKFLIVSAMLAVISWLSLGVVSFPMLAGTLGISTKAAATVVNLISAYSTVTAVISIVGAITGVGSIGSGIAATVLYILKKKGAAKAALW

>Streptococcus-pneumoniae_CIT59978.1

MKSKRMEFHNKFLIVSAMLAVISWLSLGVVSFPMLAGTLGISTKAAATVVNLISAYSTVTAVISIVGAITGVGSIGSGIAATVLYILKKKGAAKAALW

>Streptococcus-pneumoniae_CIR51266.1

MKSKRMEFHNKFLIVSAMLAVISWLSLGVVSFPMLAGTLGISTKAAATVVNLISAYSTVTAVISIVGAITGVGSIGSGIAATVLYILKKKGAAKAALW

>Streptococcus-pneumoniae_CIU50553.1

MKSKRMEFHNKFLIVSAMLAVISWLSLGVVSFPMLAGTLGISTKAAATVVNLISAYSTVTAVISIVGAITGVGSIGSGIAATVLYILKKKGAAKAALW

>Streptococcus-pneumoniae_CIQ12045.1

MKSKRMEFHNKFLIVSAMLAVISWLSLGVVSFPMLAGTLGISTKAAATVVNLISAYSTVTAVISIVGAITGVGSIGSGIAATVLYILKKKGAAKAALW

>Streptococcus-pneumoniae_CIO91701.1

MKSKRMEFHNKFLIVSAMLAVISWLSLGVVSFPMLAGTLGISTKAAATVVNLISAYSTVTAVISIVGAITGVGSIGSGIAATVLYILKKKGAAKAALW

>Streptococcus-pneumoniae_CIP86321.1

MKSKRMEFHNKFLIVSAMLAVISWLSLGVVSFPMLAGTLGISTKAAATVVNLISAYSTVTAVISIVGAITGVGSIGSGIAATVLYILKKKGAAKAALW

>Streptococcus-pneumoniae_CIO41708.1

MKSKRMEFHNKFLIVSAMLAVISWLSLGVVSFPMLAGTLGISTKAAATVVNLISAYSTVTAVISIVGAITGVGSIGSGIAATVLYILKKKGAAKAALW

>Streptococcus-pneumoniae_CIO67067.1

MKSKRMEFHNKFLIVSAMLAVISWLSLGVVSFPMLAGTLGISTKAAATVVNLISAYSTVTAVISIVGAITGVGSIGSGIAATVLYILKKKGAAKAALW

>Streptococcus-pneumoniae_CIV54862.1

MKSKRMEFHNKFLIVSAMLAVISWLSLGVVSFPMLAGTLGISTKAAATVVNLISAYSTVTAVISIVGAITGVGSIGSGIAATVLYILKKKGAAKAALW

>Streptococcus-pneumoniae_CIQ87080.1

MKSKRMEFHNKFLIVSAMLAVISWLSLGVVSFPMLAGTLGISTKAAATVVNLISAYSTVTAVISIVGAITGVGSIGSGIAATVLYILKKKGAAKAALW

>Streptococcus-pneumoniae_CJM32993.1

MKSKRMEFHNKFLIVSAMLAVISWLSLGVVSFPMLAGTLGISTKAAATVVNLISAYSTVTAVISIVGAITGVGSIGSGIAATVLYILKKKGAAKAALW

>Streptococcus-pneumoniae_CJR87351.1

MKSKRMEFHNKFLIVSAMLAVISWLSLGVVSFPMLAGTLGISTKAAATVVNLISAYSTVTAVISIVGAITGVGSIGSGIAATVLYILKKKGAAKAALW

>Streptococcus-pneumoniae_CJA43362.1

MKSKRMEFHNKFLIVSAMLAVISWLSLGVVSFPMLAGTLGISTKAAATVVNLISAYSTVTAVISIVGAITGVGSIGSGIAATVLYILKKKGAAKAALW

>Streptococcus-pneumoniae_CJP07009.1

MKSKRMEFHNKFLIVSAMLAVISWLSLGVVSFPMLAGTLGISTKAAATVVNLISAYSTVTAVISIVGAITGVGSIGSGIAATVLYILKKKGAAKAALW

>Streptococcus-pneumoniae_CJC96632.1

MKSKRMEFHNKFLIVSAMLAVISWLSLGVVSFPMLAGTLGISTKAAATVVNLISAYSTVTAVISIVGAITGVGSIGSGIAATVLYILKKKGAAKAALW

>Streptococcus-pneumoniae_CJD59571.1

MKSKRMEFHNKFLIVSAMLAVISWLSLGVVSFPMLAGTLGISTKAAATVVNLISAYSTVTAVISIVGAITGVGSIGSGIAATVLYILKKKGAAKAALW

>Streptococcus-pneumoniae_CJD92621.1

MKSKRMEFHNKFLIVSAMLAVISWLSLGVVSFPMLAGTLGISTKAAATVVNLISAYSTVTAVISIVGAITGVGSIGSGIAATVLYILKKKGAAKAALW

>Streptococcus-pneumoniae_CJH73772.1

MKSKRMEFHNKFLIVSAMLAVISWLSLGVVSFPMLAGTLGISTKAAATVVNLISAYSTVTAVISIVGAITGVGSIGSGIAATVLYILKKKGAAKAALW

>Streptococcus-pneumoniae_CJP22023.1

MKSKRMEFHNKFLIVSAMLAVISWLSLGVVSFPMLAGTLGISTKAAATVVNLISAYSTVTAVISIVGAITGVGSIGSGIAATVLYILKKKGAAKAALW

>Streptococcus-pneumoniae_CJA70582.1

MKSKRMEFHNKFLIVSAMLAVISWLSLGVVSFPMLAGTLGISTKAAATVVNLISAYSTVTAVISIVGAITGVGSIGSGIAATVLYILKKKGAAKAALW

>Streptococcus-pneumoniae_CJQ83203.1

MKSKRMEFHNKFLIVSAMLAVISWLSLGVVSFPMLAGTLGISTKAAATVVNLISAYSTVTAVISIVGAITGVGSIGSGIAATVLYILKKKGAAKAALW

>Streptococcus-pneumoniae_CJP69145.1

MKSKRMEFHNKFLIVSAMLAVISWLSLGVVSFPMLAGTLGISTKAAATVVNLISAYSTVTAVISIVGAITGVGSIGSGIAATVLYILKKKGAAKAALW

>Streptococcus-pneumoniae_CJJ70274.1

MKSKRMEFHNKFLIVSAMLAVISWLSLGVVSFPMLAGTLGISTKAAATVVNLISAYSTVTAVISIVGAITGVGSIGSGIAATVLYILKKKGAAKAALW

>Streptococcus-pneumoniae_CJO62975.1

MKSKRMEFHNKFLIVSAMLAVISWLSLGVVSFPMLAGTLGISTKAAATVVNLISAYSTVTAVISIVGAITGVGSIGSGIAATVLYILKKKGAAKAALW

>Streptococcus-pneumoniae_CJD32582.1

MKSKRMEFHNKFLIVSAMLAVISWLSLGVVSFPMLAGTLGISTKAAATVVNLISAYSTVTAVISIVGAITGVGSIGSGIAATVLYILKKKGAAKAALW

>Streptococcus-pneumoniae_CJB34842.1

MKSKRMEFHNKFLIVSAMLAVISWLSLGVVSFPMLAGTLGISTKAAATVVNLISAYSTVTAVISIVGAITGVGSIGSGIAATVLYILKKKGAAKAALW

>Streptococcus-pneumoniae_CJT14214.1

MKSKRMEFHNKFLIVSAMLAVISWLSLGVVSFPMLAGTLGISTKAAATVVNLISAYSTVTAVISIVGAITGVGSIGSGIAATVLYILKKKGAAKAALW

>Streptococcus-pneumoniae_CJC13229.1

MKSKRMEFHNKFLIVSAMLAVISWLSLGVVSFPMLAGTLGISTKAAATVVNLISAYSTVTAVISIVGAITGVGSIGSGIAATVLYILKKKGAAKAALW

>Streptococcus-pneumoniae_CJM33200.1

MKSKRMEFHNKFLIVSAMLAVISWLSLGVVSFPMLAGTLGISTKAAATVVNLISAYSTVTAVISIVGAITGVGSIGSGIAATVLYILKKKGAAKAALW

>Streptococcus-pneumoniae_CJQ34143.1

MKSKRMEFHNKFLIVSAMLAVISWLSLGVVSFPMLAGTLGISTKAAATVVNLISAYSTVTAVISIVGAITGVGSIGSGIAATVLYILKKKGAAKAALW

>Streptococcus-pneumoniae_CJR32419.1

MKSKRMEFHNKFLIVSAMLAVISWLSLGVVSFPMLAGTLGISTKAAATVVNLISAYSTVTAVISIVGAITGVGSIGSGIAATVLYILKKKGAAKAALW

>Streptococcus-pneumoniae_CJQ26174.1

MKSKRMEFHNKFLIVSAMLAVISWLSLGVVSFPMLAGTLGISTKAAATVVNLISAYSTVTAVISIVGAITGVGSIGSGIAATVLYILKKKGAAKAALW

>Streptococcus-pneumoniae_CJQ31741.1

MKSKRMEFHNKFLIVSAMLAVISWLSLGVVSFPMLAGTLGISTKAAATVVNLISAYSTVTAVISIVGAITGVGSIGSGIAATVLYILKKKGAAKAALW

>Streptococcus-pneumoniae_CJN45546.1

MKSKRMEFHNKFLIVSAMLAVISWLSLGVVSFPMLAGTLGISTKAAATVVNLISAYSTVTAVISIVGAITGVGSIGSGIAATVLYILKKKGAAKAALW

>Streptococcus-pneumoniae_CJH69669.1

MKSKRMEFHNKFLIVSAMLAVISWLSLGVVSFPMLAGTLGISTKAAATVVNLISAYSTVTAVISIVGAITGVGSIGSGIAATVLYILKKKGAAKAALW

>Streptococcus-pneumoniae_CJJ53003.1

MKSKRMEFHNKFLIVSAMLAVISWLSLGVVSFPMLAGTLGISTKAAATVVNLISAYSTVTAVISIVGAITGVGSIGSGIAATVLYILKKKGAAKAALW

>Streptococcus-pneumoniae_CJQ43128.1

MKSKRMEFHNKFLIVSAMLAVISWLSLGVVSFPMLAGTLGISTKAAATVVNLISAYSTVTAVISIVGAITGVGSIGSGIAATVLYILKKKGAAKAALW

>Streptococcus-pneumoniae_CJR61865.1

MKSKRMEFHNKFLIVSAMLAVISWLSLGVVSFPMLAGTLGISTKAAATVVNLISAYSTVTAVISIVGAITGVGSIGSGIAATVLYILKKKGAAKAALW

>Streptococcus-pneumoniae_CJK88507.1

MKSKRMEFHNKFLIVSAMLAVISWLSLGVVSFPMLAGTLGISTKAAATVVNLISAYSTVTAVISIVGAITGVGSIGSGIAATVLYILKKKGAAKAALW

>Streptococcus-pneumoniae_CJK63180.1

MKSKRMEFHNKFLIVSAMLAVISWLSLGVVSFPMLAGTLGISTKAAATVVNLISAYSTVTAVISIVGAITGVGSIGSGIAATVLYILKKKGAAKAALW

>Streptococcus-pneumoniae_CJF19533.1

MKSKRMEFHNKFLIVSAMLAVISWLSLGVVSFPMLAGTLGISTKAAATVVNLISAYSTVTAVISIVGAITGVGSIGSGIAATVLYILKKKGAAKAALW

>Streptococcus-pneumoniae_CJH50290.1

MKSKRMEFHNKFLIVSAMLAVISWLSLGVVSFPMLAGTLGISTKAAATVVNLISAYSTVTAVISIVGAITGVGSIGSGIAATVLYILKKKGAAKAALW

>Streptococcus-pneumoniae_CJO06888.1

MKSKRMEFHNKFLIVSAMLAVISWLSLGVVSFPMLAGTLGISTKAAATVVNLISAYSTVTAVISIVGAITGVGSIGSGIAATVLYILKKKGAAKAALW

>Streptococcus-pneumoniae_CJK86779.1

MKSKRMEFHNKFLIVSAMLAVISWLSLGVVSFPMLAGTLGISTKAAATVVNLISAYSTVTAVISIVGAITGVGSIGSGIAATVLYILKKKGAAKAALW

>Streptococcus-pneumoniae_CJA64105.1

MKSKRMEFHNKFLIVSAMLAVISWLSLGVVSFPMLAGTLGISTKAAATVVNLISAYSTVTAVISIVGAITGVGSIGSGIAATVLYILKKKGAAKAALW

>Streptococcus-pneumoniae_CJI77581.1

MKSKRMEFHNKFLIVSAMLAVISWLSLGVVSFPMLAGTLGISTKAAATVVNLISAYSTVTAVISIVGAITGVGSIGSGIAATVLYILKKKGAAKAALW

>Streptococcus-pneumoniae_CJQ36407.1

MKSKRMEFHNKFLIVSAMLAVISWLSLGVVSFPMLAGTLGISTKAAATVVNLISAYSTVTAVISIVGAITGVGSIGSGIAATVLYILKKKGAAKAALW

>Streptococcus-pneumoniae_CJL11409.1

MKSKRMEFHNKFLIVSAMLAVISWLSLGVVSFPMLAGTLGISTKAAATVVNLISAYSTVTAVISIVGAITGVGSIGSGIAATVLYILKKKGAAKAALW

>Streptococcus-pneumoniae_CJL68987.1

MKSKRMEFHNKFLIVSAMLAVISWLSLGVVSFPMLAGTLGISTKAAATVVNLISAYSTVTAVISIVGAITGVGSIGSGIAATVLYILKKKGAAKAALW

>Streptococcus-pneumoniae_CJE66761.1

MKSKRMEFHNKFLIVSAMLAVISWLSLGVVSFPMLAGTLGISTKAAATVVNLISAYSTVTAVISIVGAITGVGSIGSGIAATVLYILKKKGAAKAALW

>Streptococcus-pneumoniae_CJD67786.1

MKSKRMEFHNKFLIVSAMLAVISWLSLGVVSFPMLAGTLGISTKAAATVVNLISAYSTVTAVISIVGAITGVGSIGSGIAATVLYILKKKGAAKAALW

>Streptococcus-pneumoniae_CJS56616.1

MKSKRMEFHNKFLIVSAMLAVISWLSLGVVSFPMLAGTLGISTKAAATVVNLISAYSTVTAVISIVGAITGVGSIGSGIAATVLYILKKKGAAKAALW

>Streptococcus-pneumoniae_CJN90638.1

MKSKRMEFHNKFLIVSAMLAVISWLSLGVVSFPMLAGTLGISTKAAATVVNLISAYSTVTAVISIVGAITGVGSIGSGIAATVLYILKKKGAAKAALW

>Streptococcus-pneumoniae_CJI72568.1

MKSKRMEFHNKFLIVSAMLAVISWLSLGVVSFPMLAGTLGISTKAAATVVNLISAYSTVTAVISIVGAITGVGSIGSGIAATVLYILKKKGAAKAALW

>Streptococcus-pneumoniae_CJC92768.1

MKSKRMEFHNKFLIVSAMLAVISWLSLGVVSFPMLAGTLGISTKAAATVVNLISAYSTVTAVISIVGAITGVGSIGSGIAATVLYILKKKGAAKAALW

>Streptococcus-pneumoniae_CJL58290.1

MKSKRMEFHNKFLIVSAMLAVISWLSLGVVSFPMLAGTLGISTKAAATVVNLISAYSTVTAVISIVGAITGVGSIGSGIAATVLYILKKKGAAKAALW

>Streptococcus-pneumoniae_CJC18069.1

MKSKRMEFHNKFLIVSAMLAVISWLSLGVVSFPMLAGTLGISTKAAATVVNLISAYSTVTAVISIVGAITGVGSIGSGIAATVLYILKKKGAAKAALW

>Streptococcus-pneumoniae_CJT19379.1

MKSKRMEFHNKFLIVSAMLAVISWLSLGVVSFPMLAGTLGISTKAAATVVNLISAYSTVTAVISIVGAITGVGSIGSGIAATVLYILKKKGAAKAALW

>Streptococcus-pneumoniae_CJK76110.1

MKSKRMEFHNKFLIVSAMLAVISWLSLGVVSFPMLAGTLGISTKAAATVVNLISAYSTVTAVISIVGAITGVGSIGSGIAATVLYILKKKGAAKAALW

>Streptococcus-pneumoniae_CJL83916.1

MKSKRMEFHNKFLIVSAMLAVISWLSLGVVSFPMLAGTLGISTKAAATVVNLISAYSTVTAVISIVGAITGVGSIGSGIAATVLYILKKKGAAKAALW

>Streptococcus-pneumoniae_CJS43538.1

MKSKRMEFHNKFLIVSAMLAVISWLSLGVVSFPMLAGTLGISTKAAATVVNLISAYSTVTAVISIVGAITGVGSIGSGIAATVLYILKKKGAAKAALW

>Streptococcus-pneumoniae_CJM57675.1

MKSKRMEFHNKFLIVSAMLAVISWLSLGVVSFPMLAGTLGISTKAAATVVNLISAYSTVTAVISIVGAITGVGSIGSGIAATVLYILKKKGAAKAALW

>Streptococcus-pneumoniae_CJC78182.1

MKSKRMEFHNKFLIVSAMLAVISWLSLGVVSFPMLAGTLGISTKAAATVVNLISAYSTVTAVISIVGAITGVGSIGSGIAATVLYILKKKGAAKAALW

>Streptococcus-pneumoniae_CJJ85583.1

MKSKRMEFHNKFLIVSAMLAVISWLSLGVVSFPMLAGTLGISTKAAATVVNLISAYSTVTAVISIVGAITGVGSIGSGIAATVLYILKKKGAAKAALW

>Streptococcus-pneumoniae_CJQ59542.1

MKSKRMEFHNKFLIVSAMLAVISWLSLGVVSFPMLAGTLGISTKAAATVVNLISAYSTVTAVISIVGAITGVGSIGSGIAATVLYILKKKGAAKAALW

>Streptococcus-pneumoniae_CJF80020.1

MKSKRMEFHNKFLIVSAMLAVISWLSLGVVSFPMLAGTLGISTKAAATVVNLISAYSTVTAVISIVGAITGVGSIGSGIAATVLYILKKKGAAKAALW

>Streptococcus-pneumoniae_CJM76195.1

MKSKRMEFHNKFLIVSAMLAVISWLSLGVVSFPMLAGTLGISTKAAATVVNLISAYSTVTAVISIVGAITGVGSIGSGIAATVLYILKKKGAAKAALW

>Streptococcus-pneumoniae_CJI70470.1

MKSKRMEFHNKFLIVSAMLAVISWLSLGVVSFPMLAGTLGISTKAAATVVNLISAYSTVTAVISIVGAITGVGSIGSGIAATVLYILKKKGAAKAALW

>Streptococcus-pneumoniae_CJE51729.1

MKSKRMEFHNKFLIVSAMLAVISWLSLGVVSFPMLAGTLGISTKAAATVVNLISAYSTVTAVISIVGAITGVGSIGSGIAATVLYILKKKGAAKAALW

>Streptococcus-pneumoniae_CJT60335.1

MKSKRMEFHNKFLIVSAMLAVISWLSLGVVSFPMLAGTLGISTKAAATVVNLISAYSTVTAVISIVGAITGVGSIGSGIAATVLYILKKKGAAKAALW

>Streptococcus-pneumoniae_CJH77531.1

MKSKRMEFHNKFLIVSAMLAVISWLSLGVVSFPMLAGTLGISTKAAATVVNLISAYSTVTAVISIVGAITGVGSIGSGIAATVLYILKKKGAAKAALW

>Streptococcus-pneumoniae_CJJ27624.1

MKSKRMEFHNKFLIVSAMLAVISWLSLGVVSFPMLAGTLGISTKAAATVVNLISAYSTVTAVISIVGAITGVGSIGSGIAATVLYILKKKGAAKAALW

>Streptococcus-pneumoniae_CJR21106.1

MKSKRMEFHNKFLIVSAMLAVISWLSLGVVSFPMLAGTLGISTKAAATVVNLISAYSTVTAVISIVGAITGVGSIGSGIAATVLYILKKKGAAKAALW

>Streptococcus-pneumoniae_CJQ66511.1

MKSKRMEFHNKFLIVSAMLAVISWLSLGVVSFPMLAGTLGISTKAAATVVNLISAYSTVTAVISIVGAITGVGSIGSGIAATVLYILKKKGAAKAALW

>Streptococcus-pneumoniae_CJB70414.1

MKSKRMEFHNKFLIVSAMLAVISWLSLGVVSFPMLAGTLGISTKAAATVVNLISAYSTVTAVISIVGAITGVGSIGSGIAATVLYILKKKGAAKAALW

>Streptococcus-pneumoniae_CJB76033.1

MKSKRMEFHNKFLIVSAMLAVISWLSLGVVSFPMLAGTLGISTKAAATVVNLISAYSTVTAVISIVGAITGVGSIGSGIAATVLYILKKKGAAKAALW

>Streptococcus-pneumoniae_CJS48734.1

MKSKRMEFHNKFLIVSAMLAVISWLSLGVVSFPMLAGTLGISTKAAATVVNLISAYSTVTAVISIVGAITGVGSIGSGIAATVLYILKKKGAAKAALW

>Streptococcus-pneumoniae_CJH71512.1

MKSKRMEFHNKFLIVSAMLAVISWLSLGVVSFPMLAGTLGISTKAAATVVNLISAYSTVTAVISIVGAITGVGSIGSGIAATVLYILKKKGAAKAALW

>Streptococcus-pneumoniae_CJJ72710.1

MKSKRMEFHNKFLIVSAMLAVISWLSLGVVSFPMLAGTLGISTKAAATVVNLISAYSTVTAVISIVGAITGVGSIGSGIAATVLYILKKKGAAKAALW

>Streptococcus-pneumoniae_CJT31149.1

MKSKRMEFHNKFLIVSAMLAVISWLSLGVVSFPMLAGTLGISTKAAATVVNLISAYSTVTAVISIVGAITGVGSIGSGIAATVLYILKKKGAAKAALW

>Streptococcus-pneumoniae_CJF32954.1

MKSKRMEFHNKFLIVSAMLAVISWLSLGVVSFPMLAGTLGISTKAAATVVNLISAYSTVTAVISIVGAITGVGSIGSGIAATVLYILKKKGAAKAALW

>Streptococcus-pneumoniae_CJT60645.1

MKSKRMEFHNKFLIVSAMLAVISWLSLGVVSFPMLAGTLGISTKAAATVVNLISAYSTVTAVISIVGAITGVGSIGSGIAATVLYILKKKGAAKAALW

>Streptococcus-pneumoniae_CJC64232.1

MKSKRMEFHNKFLIVSAMLAVISWLSLGVVSFPMLAGTLGISTKAAATVVNLISAYSTVTAVISIVGAITGVGSIGSGIAATVLYILKKKGAAKAALW

>Streptococcus-pneumoniae_CJS79423.1

MKSKRMEFHNKFLIVSAMLAVISWLSLGVVSFPMLAGTLGISTKAAATVVNLISAYSTVTAVISIVGAITGVGSIGSGIAATVLYILKKKGAAKAALW

>Streptococcus-pneumoniae_CJD82997.1

MKSKRMEFHNKFLIVSAMLAVISWLSLGVVSFPMLAGTLGISTKAAATVVNLISAYSTVTAVISIVGAITGVGSIGSGIAATVLYILKKKGAAKAALW

>Streptococcus-pneumoniae_CJL82592.1

MKSKRMEFHNKFLIVSAMLAVISWLSLGVVSFPMLAGTLGISTKAAATVVNLISAYSTVTAVISIVGAITGVGSIGSGIAATVLYILKKKGAAKAALW

>Streptococcus-pneumoniae_CJA40013.1

MKSKRMEFHNKFLIVSAMLAVISWLSLGVVSFPMLAGTLGISTKAAATVVNLISAYSTVTAVISIVGAITGVGSIGSGIAATVLYILKKKGAAKAALW

>Streptococcus-pneumoniae_CJN33309.1

MKSKRMEFHNKFLIVSAMLAVISWLSLGVVSFPMLAGTLGISTKAAATVVNLISAYSTVTAVISIVGAITGVGSIGSGIAATVLYILKKKGAAKAALW

>Streptococcus-pneumoniae_CJH82647.1

MKSKRMEFHNKFLIVSAMLAVISWLSLGVVSFPMLAGTLGISTKAAATVVNLISAYSTVTAVISIVGAITGVGSIGSGIAATVLYILKKKGAAKAALW

>Streptococcus-pneumoniae_CJJ42086.1

MKSKRMEFHNKFLIVSAMLAVISWLSLGVVSFPMLAGTLGISTKAAATVVNLISAYSTVTAVISIVGAITGVGSIGSGIAATVLYILKKKGAAKAALW

>Streptococcus-pneumoniae_CJD86744.1

MKSKRMEFHNKFLIVSAMLAVISWLSLGVVSFPMLAGTLGISTKAAATVVNLISAYSTVTAVISIVGAITGVGSIGSGIAATVLYILKKKGAAKAALW

>Streptococcus-pneumoniae_CJE79679.1

MKSKRMEFHNKFLIVSAMLAVISWLSLGVVSFPMLAGTLGISTKAAATVVNLISAYSTVTAVISIVGAITGVGSIGSGIAATVLYILKKKGAAKAALW

>Streptococcus-pneumoniae_CJS04830.1

MKSKRMEFHNKFLIVSAMLAVISWLSLGVVSFPMLAGTLGISTKAAATVVNLISAYSTVTAVISIVGAITGVGSIGSGIAATVLYILKKKGAAKAALW

>Streptococcus-pneumoniae_CJO35687.1

MKSKRMEFHNKFLIVSAMLAVISWLSLGVVSFPMLAGTLGISTKAAATVVNLISAYSTVTAVISIVGAITGVGSIGSGIAATVLYILKKKGAAKAALW

>Streptococcus-pneumoniae_CJO67869.1

MKSKRMEFHNKFLIVSAMLAVISWLSLGVVSFPMLAGTLGISTKAAATVVNLISAYSTVTAVISIVGAITGVGSIGSGIAATVLYILKKKGAAKAALW

>Streptococcus-pneumoniae_CJO78895.1

MKSKRMEFHNKFLIVSAMLAVISWLSLGVVSFPMLAGTLGISTKAAATVVNLISAYSTVTAVISIVGAITGVGSIGSGIAATVLYILKKKGAAKAALW

>Streptococcus-pneumoniae_CJS74733.1

MKSKRMEFHNKFLIVSAMLAVISWLSLGVVSFPMLAGTLGISTKAAATVVNLISAYSTVTAVISIVGAITGVGSIGSGIAATVLYILKKKGAAKAALW

>Streptococcus-pneumoniae_CJI54446.1

MKSKRMEFHNKFLIVSAMLAVISWLSLGVVSFPMLAGTLGISTKAAATVVNLISAYSTVTAVISIVGAITGVGSIGSGIAATVLYILKKKGAAKAALW

>Streptococcus-pneumoniae_CJN92276.1

MKSKRMEFHNKFLIVSAMLAVISWLSLGVVSFPMLAGTLGISTKAAATVVNLISAYSTVTAVISIVGAITGVGSIGSGIAATVLYILKKKGAAKAALW

>Streptococcus-pneumoniae_CJD45229.1

MKSKRMEFHNKFLIVSAMLAVISWLSLGVVSFPMLAGTLGISTKAAATVVNLISAYSTVTAVISIVGAITGVGSIGSGIAATVLYILKKKGAAKAALW

>Streptococcus-pneumoniae_CJK03597.1

MKSKRMEFHNKFLIVSAMLAVISWLSLGVVSFPMLAGTLGISTKAAATVVNLISAYSTVTAVISIVGAITGVGSIGSGIAATVLYILKKKGAAKAALW

>Streptococcus-pneumoniae_CJF52023.1

MKSKRMEFHNKFLIVSAMLAVISWLSLGVVSFPMLAGTLGISTKAAATVVNLISAYSTVTAVISIVGAITGVGSIGSGIAATVLYILKKKGAAKAALW

>Streptococcus-pneumoniae_CJL91513.1

MKSKRMEFHNKFLIVSAMLAVISWLSLGVVSFPMLAGTLGISTKAAATVVNLISAYSTVTAVISIVGAITGVGSIGSGIAATVLYILKKKGAAKAALW

>Streptococcus-pneumoniae_CJE28506.1

MKSKRMEFHNKFLIVSAMLAVISWLSLGVVSFPMLAGTLGISTKAAATVVNLISAYSTVTAVISIVGAITGVGSIGSGIAATVLYILKKKGAAKAALW

>Streptococcus-pneumoniae_CJF82807.1

MKSKRMEFHNKFLIVSAMLAVISWLSLGVVSFPMLAGTLGISTKAAATVVNLISAYSTVTAVISIVGAITGVGSIGSGIAATVLYILKKKGAAKAALW

>Streptococcus-pneumoniae_CJL76159.1

MKSKRMEFHNKFLIVSAMLAVISWLSLGVVSFPMLAGTLGISTKAAATVVNLISAYSTVTAVISIVGAITGVGSIGSGIAATVLYILKKKGAAKAALW

>Streptococcus-pneumoniae_CJP89685.1

MKSKRMEFHNKFLIVSAMLAVISWLSLGVVSFPMLAGTLGISTKAAATVVNLISAYSTVTAVISIVGAITGVGSIGSGIAATVLYILKKKGAAKAALW

>Streptococcus-pneumoniae_CJE14050.1

MKSKRMEFHNKFLIVSAMLAVISWLSLGVVSFPMLAGTLGISTKAAATVVNLISAYSTVTAVISIVGAITGVGSIGSGIAATVLYILKKKGAAKAALW

>Streptococcus-pneumoniae_CJO01335.1

MKSKRMEFHNKFLIVSAMLAVISWLSLGVVSFPMLAGTLGISTKAAATVVNLISAYSTVTAVISIVGAITGVGSIGSGIAATVLYILKKKGAAKAALW

>Streptococcus-pneumoniae_CJA73359.1

MKSKRMEFHNKFLIVSAMLAVISWLSLGVVSFPMLAGTLGISTKAAATVVNLISAYSTVTAVISIVGAITGVGSIGSGIAATVLYILKKKGAAKAALW

>Streptococcus-pneumoniae_CJB88388.1

MKSKRMEFHNKFLIVSAMLAVISWLSLGVVSFPMLAGTLGISTKAAATVVNLISAYSTVTAVISIVGAITGVGSIGSGIAATVLYILKKKGAAKAALW

>Streptococcus-pneumoniae_CJT32038.1

MKSKRMEFHNKFLIVSAMLAVISWLSLGVVSFPMLAGTLGISTKAAATVVNLISAYSTVTAVISIVGAITGVGSIGSGIAATVLYILKKKGAAKAALW

>Streptococcus-pneumoniae_CJG53532.1

MKSKRMEFHNKFLIVSAMLAVISWLSLGVVSFPMLAGTLGISTKAAATVVNLISAYSTVTAVISIVGAITGVGSIGSGIAATVLYILKKKGAAKAALW

>Streptococcus-pneumoniae_CJT41146.1

MKSKRMEFHNKFLIVSAMLAVISWLSLGVVSFPMLAGTLGISTKAAATVVNLISAYSTVTAVISIVGAITGVGSIGSGIAATVLYILKKKGAAKAALW

>Streptococcus-pneumoniae_CJL80129.1

MKSKRMEFHNKFLIVSAMLAVISWLSLGVVSFPMLAGTLGISTKAAATVVNLISAYSTVTAVISIVGAITGVGSIGSGIAATVLYILKKKGAAKAALW

>Streptococcus-pneumoniae_CJF91238.1

MKSKRMEFHNKFLIVSAMLAVISWLSLGVVSFPMLAGTLGISTKAAATVVNLISAYSTVTAVISIVGAITGVGSIGSGIAATVLYILKKKGAAKAALW

>Streptococcus-pneumoniae_CJR42350.1

MKSKRMEFHNKFLIVSAMLAVISWLSLGVVSFPMLAGTLGISTKAAATVVNLISAYSTVTAVISIVGAITGVGSIGSGIAATVLYILKKKGAAKAALW

>Streptococcus-pneumoniae_CJQ79346.1

MKSKRMEFHNKFLIVSAMLAVISWLSLGVVSFPMLAGTLGISTKAAATVVNLISAYSTVTAVISIVGAITGVGSIGSGIAATVLYILKKKGAAKAALW

>Streptococcus-pneumoniae_CJS38280.1

MKSKRMEFHNKFLIVSAMLAVISWLSLGVVSFPMLAGTLGISTKAAATVVNLISAYSTVTAVISIVGAITGVGSIGSGIAATVLYILKKKGAAKAALW

>Streptococcus-pneumoniae_CJL93036.1

MKSKRMEFHNKFLIVSAMLAVISWLSLGVVSFPMLAGTLGISTKAAATVVNLISAYSTVTAVISIVGAITGVGSIGSGIAATVLYILKKKGAAKAALW

>Streptococcus-pneumoniae_CJM54014.1

MKSKRMEFHNKFLIVSAMLAVISWLSLGVVSFPMLAGTLGISTKAAATVVNLISAYSTVTAVISIVGAITGVGSIGSGIAATVLYILKKKGAAKAALW

>Streptococcus-pneumoniae_CJG22828.1

MKSKRMEFHNKFLIVSAMLAVISWLSLGVVSFPMLAGTLGISTKAAATVVNLISAYSTVTAVISIVGAITGVGSIGSGIAATVLYILKKKGAAKAALW

>Streptococcus-pneumoniae_CJA03446.1

MKSKRMEFHNKFLIVSAMLAVISWLSLGVVSFPMLAGTLGISTKAAATVVNLISAYSTVTAVISIVGAITGVGSIGSGIAATVLYILKKKGAAKAALW

>Streptococcus-pneumoniae_CJM75937.1

MKSKRMEFHNKFLIVSAMLAVISWLSLGVVSFPMLAGTLGISTKAAATVVNLISAYSTVTAVISIVGAITGVGSIGSGIAATVLYILKKKGAAKAALW

>Streptococcus-pneumoniae_CJN53367.1

MKSKRMEFHNKFLIVSAMLAVISWLSLGVVSFPMLAGTLGISTKAAATVVNLISAYSTVTAVISIVGAITGVGSIGSGIAATVLYILKKKGAAKAALW

>Streptococcus-pneumoniae_CJJ77091.1

MKSKRMEFHNKFLIVSAMLAVISWLSLGVVSFPMLAGTLGISTKAAATVVNLISAYSTVTAVISIVGAITGVGSIGSGIAATVLYILKKKGAAKAALW

>Streptococcus-pneumoniae_CJO79524.1

MKSKRMEFHNKFLIVSAMLAVISWLSLGVVSFPMLAGTLGISTKAAATVVNLISAYSTVTAVISIVGAITGVGSIGSGIAATVLYILKKKGAAKAALW

>Streptococcus-pneumoniae_CJD75826.1

MKSKRMEFHNKFLIVSAMLAVISWLSLGVVSFPMLAGTLGISTKAAATVVNLISAYSTVTAVISIVGAITGVGSIGSGIAATVLYILKKKGAAKAALW

>Streptococcus-pneumoniae_CJM25807.1

MKSKRMEFHNKFLIVSAMLAVISWLSLGVVSFPMLAGTLGISTKAAATVVNLISAYSTVTAVISIVGAITGVGSIGSGIAATVLYILKKKGAAKAALW

>Streptococcus-pneumoniae_CIZ96333.1

MKSKRMEFHNKFLIVSAMLAVISWLSLGVVSFPMLAGTLGISTKAAATVVNLISAYSTVTAVISIVGAITGVGSIGSGIAATVLYILKKKGAAKAALW

>Streptococcus-pneumoniae_CJM53653.1

MKSKRMEFHNKFLIVSAMLAVISWLSLGVVSFPMLAGTLGISTKAAATVVNLISAYSTVTAVISIVGAITGVGSIGSGIAATVLYILKKKGAAKAALW

>Streptococcus-pneumoniae_CJS77452.1

MKSKRMEFHNKFLIVSAMLAVISWLSLGVVSFPMLAGTLGISTKAAATVVNLISAYSTVTAVISIVGAITGVGSIGSGIAATVLYILKKKGAAKAALW

>Streptococcus-pneumoniae_CJA84161.1

MKSKRMEFHNKFLIVSAMLAVISWLSLGVVSFPMLAGTLGISTKAAATVVNLISAYSTVTAVISIVGAITGVGSIGSGIAATVLYILKKKGAAKAALW

>Streptococcus-pneumoniae_CJP10979.1

MKSKRMEFHNKFLIVSAMLAVISWLSLGVVSFPMLAGTLGISTKAAATVVNLISAYSTVTAVISIVGAITGVGSIGSGIAATVLYILKKKGAAKAALW

>Streptococcus-pneumoniae_CJQ71550.1

MKSKRMEFHNKFLIVSAMLAVISWLSLGVVSFPMLAGTLGISTKAAATVVNLISAYSTVTAVISIVGAITGVGSIGSGIAATVLYILKKKGAAKAALW

>Streptococcus-pneumoniae_CJD33972.1

MKSKRMEFHNKFLIVSAMLAVISWLSLGVVSFPMLAGTLGISTKAAATVVNLISAYSTVTAVISIVGAITGVGSIGSGIAATVLYILKKKGAAKAALW

>Streptococcus-pneumoniae_CJM05375.1

MKSKRMEFHNKFLIVSAMLAVISWLSLGVVSFPMLAGTLGISTKAAATVVNLISAYSTVTAVISIVGAITGVGSIGSGIAATVLYILKKKGAAKAALW

>Streptococcus-pneumoniae_CJN34028.1

MKSKRMEFHNKFLIVSAMLAVISWLSLGVVSFPMLAGTLGISTKAAATVVNLISAYSTVTAVISIVGAITGVGSIGSGIAATVLYILKKKGAAKAALW

>Streptococcus-pneumoniae_CJT71088.1

MKSKRMEFHNKFLIVSAMLAVISWLSLGVVSFPMLAGTLGISTKAAATVVNLISAYSTVTAVISIVGAITGVGSIGSGIAATVLYILKKKGAAKAALW

>Streptococcus-pneumoniae_CJJ51315.1

MKSKRMEFHNKFLIVSAMLAVISWLSLGVVSFPMLAGTLGISTKAAATVVNLISAYSTVTAVISIVGAITGVGSIGSGIAATVLYILKKKGAAKAALW

>Streptococcus-pneumoniae_CJH02267.1

MKSKRMEFHNKFLIVSAMLAVISWLSLGVVSFPMLAGTLGISTKAAATVVNLISAYSTVTAVISIVGAITGVGSIGSGIAATVLYILKKKGAAKAALW

>Streptococcus-pneumoniae_CJK69522.1

MKSKRMEFHNKFLIVSAMLAVISWLSLGVVSFPMLAGTLGISTKAAATVVNLISAYSTVTAVISIVGAITGVGSIGSGIAATVLYILKKKGAAKAALW

>Streptococcus-pneumoniae_CJI87660.1

MKSKRMEFHNKFLIVSAMLAVISWLSLGVVSFPMLAGTLGISTKAAATVVNLISAYSTVTAVISIVGAITGVGSIGSGIAATVLYILKKKGAAKAALW

>Streptococcus-pneumoniae_CJG56793.1

MKSKRMEFHNKFLIVSAMLAVISWLSLGVVSFPMLAGTLGISTKAAATVVNLISAYSTVTAVISIVGAITGVGSIGSGIAATVLYILKKKGAAKAALW

>Streptococcus-pneumoniae_CJM44414.1

MKSKRMEFHNKFLIVSAMLAVISWLSLGVVSFPMLAGTLGISTKAAATVVNLISAYSTVTAVISIVGAITGVGSIGSGIAATVLYILKKKGAAKAALW

>Streptococcus-pneumoniae_CJB44727.1

MKSKRMEFHNKFLIVSAMLAVISWLSLGVVSFPMLAGTLGISTKAAATVVNLISAYSTVTAVISIVGAITGVGSIGSGIAATVLYILKKKGAAKAALW

>Streptococcus-pneumoniae_CJN18940.1

MKSKRMEFHNKFLIVSAMLAVISWLSLGVVSFPMLAGTLGISTKAAATVVNLISAYSTVTAVISIVGAITGVGSIGSGIAATVLYILKKKGAAKAALW

>Streptococcus-pneumoniae_CJN42351.1

MKSKRMEFHNKFLIVSAMLAVISWLSLGVVSFPMLAGTLGISTKAAATVVNLISAYSTVTAVISIVGAITGVGSIGSGIAATVLYILKKKGAAKAALW

>Streptococcus-pneumoniae_CJK86497.1

MKSKRMEFHNKFLIVSAMLAVISWLSLGVVSFPMLAGTLGISTKAAATVVNLISAYSTVTAVISIVGAITGVGSIGSGIAATVLYILKKKGAAKAALW

>Streptococcus-pneumoniae_CJC99311.1

MKSKRMEFHNKFLIVSAMLAVISWLSLGVVSFPMLAGTLGISTKAAATVVNLISAYSTVTAVISIVGAITGVGSIGSGIAATVLYILKKKGAAKAALW

>Streptococcus-pneumoniae_CJO91234.1

MKSKRMEFHNKFLIVSAMLAVISWLSLGVVSFPMLAGTLGISTKAAATVVNLISAYSTVTAVISIVGAITGVGSIGSGIAATVLYILKKKGAAKAALW

>Streptococcus-pneumoniae_CJO01934.1

MKSKRMEFHNKFLIVSAMLAVISWLSLGVVSFPMLAGTLGISTKAAATVVNLISAYSTVTAVISIVGAITGVGSIGSGIAATVLYILKKKGAAKAALW

>Streptococcus-pneumoniae_CJC66962.1

MKSKRMEFHNKFLIVSAMLAVISWLSLGVVSFPMLAGTLGISTKAAATVVNLISAYSTVTAVISIVGAITGVGSIGSGIAATVLYILKKKGAAKAALW

>Streptococcus-pneumoniae_CJH04338.1

MKSKRMEFHNKFLIVSAMLAVISWLSLGVVSFPMLAGTLGISTKAAATVVNLISAYSTVTAVISIVGAITGVGSIGSGIAATVLYILKKKGAAKAALW

>Streptococcus-pneumoniae_CJR22035.1

MKSKRMEFHNKFLIVSAMLAVISWLSLGVVSFPMLAGTLGISTKAAATVVNLISAYSTVTAVISIVGAITGVGSIGSGIAATVLYILKKKGAAKAALW

>Streptococcus-pneumoniae_CJT57674.1

MKSKRMEFHNKFLIVSAMLAVISWLSLGVVSFPMLAGTLGISTKAAATVVNLISAYSTVTAVISIVGAITGVGSIGSGIAATVLYILKKKGAAKAALW

>Streptococcus-pneumoniae_CJH25030.1

MKSKRMEFHNKFLIVSAMLAVISWLSLGVVSFPMLAGTLGISTKAAATVVNLISAYSTVTAVISIVGAITGVGSIGSGIAATVLYILKKKGAAKAALW

>Streptococcus-pneumoniae_CJR19435.1

MKSKRMEFHNKFLIVSAMLAVISWLSLGVVSFPMLAGTLGISTKAAATVVNLISAYSTVTAVISIVGAITGVGSIGSGIAATVLYILKKKGAAKAALW

>Streptococcus-pneumoniae_CJJ54423.1

MKSKRMEFHNKFLIVSAMLAVISWLSLGVVSFPMLAGTLGISTKAAATVVNLISAYSTVTAVISIVGAITGVGSIGSGIAATVLYILKKKGAAKAALW

>Streptococcus-pneumoniae_CJI47282.1

MKSKRMEFHNKFLIVSAMLAVISWLSLGVVSFPMLAGTLGISTKAAATVVNLISAYSTVTAVISIVGAITGVGSIGSGIAATVLYILKKKGAAKAALW

>Streptococcus-pneumoniae_CJG40526.1

MKSKRMEFHNKFLIVSAMLAVISWLSLGVVSFPMLAGTLGISTKAAATVVNLISAYSTVTAVISIVGAITGVGSIGSGIAATVLYILKKKGAAKAALW

>Streptococcus-pneumoniae_CJD50577.1

MKSKRMEFHNKFLIVSAMLAVISWLSLGVVSFPMLAGTLGISTKAAATVVNLISAYSTVTAVISIVGAITGVGSIGSGIAATVLYILKKKGAAKAALW

>Streptococcus-pneumoniae_CJI43720.1

MKSKRMEFHNKFLIVSAMLAVISWLSLGVVSFPMLAGTLGISTKAAATVVNLISAYSTVTAVISIVGAITGVGSIGSGIAATVLYILKKKGAAKAALW

>Streptococcus-pneumoniae_CJS18543.1

MKSKRMEFHNKFLIVSAMLAVISWLSLGVVSFPMLAGTLGISTKAAATVVNLISAYSTVTAVISIVGAITGVGSIGSGIAATVLYILKKKGAAKAALW

>Streptococcus-pneumoniae_CJQ22608.1

MKSKRMEFHNKFLIVSAMLAVISWLSLGVVSFPMLAGTLGISTKAAATVVNLISAYSTVTAVISIVGAITGVGSIGSGIAATVLYILKKKGAAKAALW

>Streptococcus-pneumoniae_CJR06847.1

MKSKRMEFHNKFLIVSAMLAVISWLSLGVVSFPMLAGTLGISTKAAATVVNLISAYSTVTAVISIVGAITGVGSIGSGIAATVLYILKKKGAAKAALW

>Streptococcus-pneumoniae_CJT79776.1

MKSKRMEFHNKFLIVSAMLAVISWLSLGVVSFPMLAGTLGISTKAAATVVNLISAYSTVTAVISIVGAITGVGSIGSGIAATVLYILKKKGAAKAALW

>Streptococcus-pneumoniae_CJC24216.1

MKSKRMEFHNKFLIVSAMLAVISWLSLGVVSFPMLAGTLGISTKAAATVVNLISAYSTVTAVISIVGAITGVGSIGSGIAATVLYILKKKGAAKAALW

>Streptococcus-pneumoniae_CJA33771.1

MKSKRMEFHNKFLIVSAMLAVISWLSLGVVSFPMLAGTLGISTKAAATVVNLISAYSTVTAVISIVGAITGVGSIGSGIAATVLYILKKKGAAKAALW

>Streptococcus-pneumoniae_CJK43158.1

MKSKRMEFHNKFLIVSAMLAVISWLSLGVVSFPMLAGTLGISTKAAATVVNLISAYSTVTAVISIVGAITGVGSIGSGIAATVLYILKKKGAAKAALW

>Streptococcus-pneumoniae_CJD37015.1

MKSKRMEFHNKFLIVSAMLAVISWLSLGVVSFPMLAGTLGISTKAAATVVNLISAYSTVTAVISIVGAITGVGSIGSGIAATVLYILKKKGAAKAALW

>Streptococcus-pneumoniae_CJH07757.1

MKSKRMEFHNKFLIVSAMLAVISWLSLGVVSFPMLAGTLGISTKAAATVVNLISAYSTVTAVISIVGAITGVGSIGSGIAATVLYILKKKGAAKAALW

>Streptococcus-pneumoniae_CJE36134.1

MKSKRMEFHNKFLIVSAMLAVISWLSLGVVSFPMLAGTLGISTKAAATVVNLISAYSTVTAVISIVGAITGVGSIGSGIAATVLYILKKKGAAKAALW

>Streptococcus-pneumoniae_CJN09896.1

MKSKRMEFHNKFLIVSAMLAVISWLSLGVVSFPMLAGTLGISTKAAATVVNLISAYSTVTAVISIVGAITGVGSIGSGIAATVLYILKKKGAAKAALW

>Streptococcus-pneumoniae_CJI63785.1

MKSKRMEFHNKFLIVSAMLAVISWLSLGVVSFPMLAGTLGISTKAAATVVNLISAYSTVTAVISIVGAITGVGSIGSGIAATVLYILKKKGAAKAALW

>Streptococcus-pneumoniae_CJR65315.1

MKSKRMEFHNKFLIVSAMLAVISWLSLGVVSFPMLAGTLGISTKAAATVVNLISAYSTVTAVISIVGAITGVGSIGSGIAATVLYILKKKGAAKAALW

>Streptococcus-pneumoniae_CJO54527.1

MKSKRMEFHNKFLIVSAMLAVISWLSLGVVSFPMLAGTLGISTKAAATVVNLISAYSTVTAVISIVGAITGVGSIGSGIAATVLYILKKKGAAKAALW

>Streptococcus-pneumoniae_CJP01233.1

MKSKRMEFHNKFLIVSAMLAVISWLSLGVVSFPMLAGTLGISTKAAATVVNLISAYSTVTAVISIVGAITGVGSIGSGIAATVLYILKKKGAAKAALW

>Streptococcus-pneumoniae_CJN11367.1

MKSKRMEFHNKFLIVSAMLAVISWLSLGVVSFPMLAGTLGISTKAAATVVNLISAYSTVTAVISIVGAITGVGSIGSGIAATVLYILKKKGAAKAALW

>Streptococcus-pneumoniae_CJA13560.1

MKSKRMEFHNKFLIVSAMLAVISWLSLGVVSFPMLAGTLGISTKAAATVVNLISAYSTVTAVISIVGAITGVGSIGSGIAATVLYILKKKGAAKAALW

>Streptococcus-pneumoniae_CJL38147.1

MKSKRMEFHNKFLIVSAMLAVISWLSLGVVSFPMLAGTLGISTKAAATVVNLISAYSTVTAVISIVGAITGVGSIGSGIAATVLYILKKKGAAKAALW

>Streptococcus-pneumoniae_CJP39833.1

MKSKRMEFHNKFLIVSAMLAVISWLSLGVVSFPMLAGTLGISTKAAATVVNLISAYSTVTAVISIVGAITGVGSIGSGIAATVLYILKKKGAAKAALW

>Streptococcus-pneumoniae_CJA58862.1

MKSKRMEFHNKFLIVSAMLAVISWLSLGVVSFPMLAGTLGISTKAAATVVNLISAYSTVTAVISIVGAITGVGSIGSGIAATVLYILKKKGAAKAALW

>Streptococcus-pneumoniae_CJE28777.1

MKSKRMEFHNKFLIVSAMLAVISWLSLGVVSFPMLAGTLGISTKAAATVVNLISAYSTVTAVISIVGAITGVGSIGSGIAATVLYILKKKGAAKAALW

>Streptococcus-pneumoniae_CJK63401.1

MKSKRMEFHNKFLIVSAMLAVISWLSLGVVSFPMLAGTLGISTKAAATVVNLISAYSTVTAVISIVGAITGVGSIGSGIAATVLYILKKKGAAKAALW

>Streptococcus-pneumoniae_CJN99071.1

MKSKRMEFHNKFLIVSAMLAVISWLSLGVVSFPMLAGTLGISTKAAATVVNLISAYSTVTAVISIVGAITGVGSIGSGIAATVLYILKKKGAAKAALW

>Streptococcus-pneumoniae_CJE83680.1

MKSKRMEFHNKFLIVSAMLAVISWLSLGVVSFPMLAGTLGISTKAAATVVNLISAYSTVTAVISIVGAITGVGSIGSGIAATVLYILKKKGAAKAALW

>Streptococcus-pneumoniae_CJM60782.1

MKSKRMEFHNKFLIVSAMLAVISWLSLGVVSFPMLAGTLGISTKAAATVVNLISAYSTVTAVISIVGAITGVGSIGSGIAATVLYILKKKGAAKAALW

>Streptococcus-pneumoniae_CJI14925.1

MKSKRMEFHNKFLIVSAMLAVISWLSLGVVSFPMLAGTLGISTKAAATVVNLISAYSTVTAVISIVGAITGVGSIGSGIAATVLYILKKKGAAKAALW

>Streptococcus-pneumoniae_CJB92623.1

MKSKRMEFHNKFLIVSAMLAVISWLSLGVVSFPMLAGTLGISTKAAATVVNLISAYSTVTAVISIVGAITGVGSIGSGIAATVLYILKKKGAAKAALW

>Streptococcus-pneumoniae_CJL66362.1

MKSKRMEFHNKFLIVSAMLAVISWLSLGVVSFPMLAGTLGISTKAAATVVNLISAYSTVTAVISIVGAITGVGSIGSGIAATVLYILKKKGAAKAALW

>Streptococcus-pneumoniae_CJP93465.1

MKSKRMEFHNKFLIVSAMLAVISWLSLGVVSFPMLAGTLGISTKAAATVVNLISAYSTVTAVISIVGAITGVGSIGSGIAATVLYILKKKGAAKAALW

>Streptococcus-pneumoniae_CJN94466.1

MKSKRMEFHNKFLIVSAMLAVISWLSLGVVSFPMLAGTLGISTKAAATVVNLISAYSTVTAVISIVGAITGVGSIGSGIAATVLYILKKKGAAKAALW

>Streptococcus-pneumoniae_CJC02701.1

MKSKRMEFHNKFLIVSAMLAVISWLSLGVVSFPMLAGTLGISTKAAATVVNLISAYSTVTAVISIVGAITGVGSIGSGIAATVLYILKKKGAAKAALW

>Streptococcus-pneumoniae_CJM26930.1

MKSKRMEFHNKFLIVSAMLAVISWLSLGVVSFPMLAGTLGISTKAAATVVNLISAYSTVTAVISIVGAITGVGSIGSGIAATVLYILKKKGAAKAALW

>Streptococcus-pneumoniae_CJR84197.1

MKSKRMEFHNKFLIVSAMLAVISWLSLGVVSFPMLAGTLGISTKAAATVVNLISAYSTVTAVISIVGAITGVGSIGSGIAATVLYILKKKGAAKAALW

>Streptococcus-pneumoniae_CJJ34971.1

MKSKRMEFHNKFLIVSAMLAVISWLSLGVVSFPMLAGTLGISTKAAATVVNLISAYSTVTAVISIVGAITGVGSIGSGIAATVLYILKKKGAAKAALW

>Streptococcus-pneumoniae_CJK47763.1

MKSKRMEFHNKFLIVSAMLAVISWLSLGVVSFPMLAGTLGISTKAAATVVNLISAYSTVTAVISIVGAITGVGSIGSGIAATVLYILKKKGAAKAALW

>Streptococcus-pneumoniae_CJP25869.1

MKSKRMEFHNKFLIVSAMLAVISWLSLGVVSFPMLAGTLGISTKAAATVVNLISAYSTVTAVISIVGAITGVGSIGSGIAATVLYILKKKGAAKAALW

>Streptococcus-pneumoniae_CJQ14116.1

MKSKRMEFHNKFLIVSAMLAVISWLSLGVVSFPMLAGTLGISTKAAATVVNLISAYSTVTAVISIVGAITGVGSIGSGIAATVLYILKKKGAAKAALW

>Streptococcus-pneumoniae_CJS14527.1

MKSKRMEFHNKFLIVSAMLAVISWLSLGVVSFPMLAGTLGISTKAAATVVNLISAYSTVTAVISIVGAITGVGSIGSGIAATVLYILKKKGAAKAALW

>Streptococcus-pneumoniae_CJE94798.1

MKSKRMEFHNKFLIVSAMLAVISWLSLGVVSFPMLAGTLGISTKAAATVVNLISAYSTVTAVISIVGAITGVGSIGSGIAATVLYILKKKGAAKAALW

>Streptococcus-pneumoniae_CJK27256.1

MKSKRMEFHNKFLIVSAMLAVISWLSLGVVSFPMLAGTLGISTKAAATVVNLISAYSTVTAVISIVGAITGVGSIGSGIAATVLYILKKKGAAKAALW

>Streptococcus-pneumoniae_CJQ07659.1

MKSKRMEFHNKFLIVSAMLAVISWLSLGVVSFPMLAGTLGISTKAAATVVNLISAYSTVTAVISIVGAITGVGSIGSGIAATVLYILKKKGAAKAALW

>Streptococcus-pneumoniae_CJE57011.1

MKSKRMEFHNKFLIVSAMLAVISWLSLGVVSFPMLAGTLGISTKAAATVVNLISAYSTVTAVISIVGAITGVGSIGSGIAATVLYILKKKGAAKAALW

>Streptococcus-pneumoniae_CJB38987.1

MKSKRMEFHNKFLIVSAMLAVISWLSLGVVSFPMLAGTLGISTKAAATVVNLISAYSTVTAVISIVGAITGVGSIGSGIAATVLYILKKKGAAKAALW

>Streptococcus-pneumoniae_CJL59534.1

MKSKRMEFHNKFLIVSAMLAVISWLSLGVVSFPMLAGTLGISTKAAATVVNLISAYSTVTAVISIVGAITGVGSIGSGIAATVLYILKKKGAAKAALW

>Streptococcus-pneumoniae_CJL89400.1

MKSKRMEFHNKFLIVSAMLAVISWLSLGVVSFPMLAGTLGISTKAAATVVNLISAYSTVTAVISIVGAITGVGSIGSGIAATVLYILKKKGAAKAALW

>Streptococcus-pneumoniae_CJK84098.1

MKSKRMEFHNKFLIVSAMLAVISWLSLGVVSFPMLAGTLGISTKAAATVVNLISAYSTVTAVISIVGAITGVGSIGSGIAATVLYILKKKGAAKAALW

>Streptococcus-pneumoniae_CJM69734.1

MKSKRMEFHNKFLIVSAMLAVISWLSLGVVSFPMLAGTLGISTKAAATVVNLISAYSTVTAVISIVGAITGVGSIGSGIAATVLYILKKKGAAKAALW

>Streptococcus-pneumoniae_CJM95478.1

MKSKRMEFHNKFLIVSAMLAVISWLSLGVVSFPMLAGTLGISTKAAATVVNLISAYSTVTAVISIVGAITGVGSIGSGIAATVLYILKKKGAAKAALW

>Streptococcus-pneumoniae_CJO97966.1

MKSKRMEFHNKFLIVSAMLAVISWLSLGVVSFPMLAGTLGISTKAAATVVNLISAYSTVTAVISIVGAITGVGSIGSGIAATVLYILKKKGAAKAALW

>Streptococcus-pneumoniae_CJR10666.1

MKSKRMEFHNKFLIVSAMLAVISWLSLGVVSFPMLAGTLGISTKAAATVVNLISAYSTVTAVISIVGAITGVGSIGSGIAATVLYILKKKGAAKAALW

>Streptococcus-pneumoniae_CJR43084.1

MKSKRMEFHNKFLIVSAMLAVISWLSLGVVSFPMLAGTLGISTKAAATVVNLISAYSTVTAVISIVGAITGVGSIGSGIAATVLYILKKKGAAKAALW

>Streptococcus-pneumoniae_CJS94667.1

MKSKRMEFHNKFLIVSAMLAVISWLSLGVVSFPMLAGTLGISTKAAATVVNLISAYSTVTAVISIVGAITGVGSIGSGIAATVLYILKKKGAAKAALW

>Streptococcus-pneumoniae_CJH79774.1

MKSKRMEFHNKFLIVSAMLAVISWLSLGVVSFPMLAGTLGISTKAAATVVNLISAYSTVTAVISIVGAITGVGSIGSGIAATVLYILKKKGAAKAALW

>Streptococcus-pneumoniae_CJP05455.1

MKSKRMEFHNKFLIVSAMLAVISWLSLGVVSFPMLAGTLGISTKAAATVVNLISAYSTVTAVISIVGAITGVGSIGSGIAATVLYILKKKGAAKAALW

>Streptococcus-pneumoniae_CJN00909.1

MKSKRMEFHNKFLIVSAMLAVISWLSLGVVSFPMLAGTLGISTKAAATVVNLISAYSTVTAVISIVGAITGVGSIGSGIAATVLYILKKKGAAKAALW

>Streptococcus-pneumoniae_CJG14433.1

MKSKRMEFHNKFLIVSAMLAVISWLSLGVVSFPMLAGTLGISTKAAATVVNLISAYSTVTAVISIVGAITGVGSIGSGIAATVLYILKKKGAAKAALW

>Streptococcus-pneumoniae_CJB08739.1

MKSKRMEFHNKFLIVSAMLAVISWLSLGVVSFPMLAGTLGISTKAAATVVNLISAYSTVTAVISIVGAITGVGSIGSGIAATVLYILKKKGAAKAALW

>Streptococcus-pneumoniae_CJK16663.1

MKSKRMEFHNKFLIVSAMLAVISWLSLGVVSFPMLAGTLGISTKAAATVVNLISAYSTVTAVISIVGAITGVGSIGSGIAATVLYILKKKGAAKAALW

>Streptococcus-pneumoniae_CJP36437.1

MKSKRMEFHNKFLIVSAMLAVISWLSLGVVSFPMLAGTLGISTKAAATVVNLISAYSTVTAVISIVGAITGVGSIGSGIAATVLYILKKKGAAKAALW

>Streptococcus-pneumoniae_CKC22640.1

MKSKRMEFHNKFLIVSAMLAVISWLSLGVVSFPMLAGTLGISTKAAATVVNLISAYSTVTAVISIVGAITGVGSIGSGIAATVLYILKKKGAAKAALW

>Streptococcus-pneumoniae_CJW35284.1

MKSKRMEFHNKFLIVSAMLAVISWLSLGVVSFPMLAGTLGISTKAAATVVNLISAYSTVTAVISIVGAITGVGSIGSGIAATVLYILKKKGAAKAALW

>Streptococcus-pneumoniae_CJY45069.1

MKSKRMEFHNKFLIVSAMLAVISWLSLGVVSFPMLAGTLGISTKAAATVVNLISAYSTVTAVISIVGAITGVGSIGSGIAATVLYILKKKGAAKAALW

>Streptococcus-pneumoniae_CKC30100.1

MKSKRMEFHNKFLIVSAMLAVISWLSLGVVSFPMLAGTLGISTKAAATVVNLISAYSTVTAVISIVGAITGVGSIGSGIAATVLYILKKKGAAKAALW

>Streptococcus-pneumoniae_CKE28833.1

MKSKRMEFHNKFLIVSAMLAVISWLSLGVVSFPMLAGTLGISTKAAATVVNLISAYSTVTAVISIVGAITGVGSIGSGIAATVLYILKKKGAAKAALW

>Streptococcus-pneumoniae_CKD69515.1

MKSKRMEFHNKFLIVSAMLAVISWLSLGVVSFPMLAGTLGISTKAAATVVNLISAYSTVTAVISIVGAITGVGSIGSGIAATVLYILKKKGAAKAALW

>Streptococcus-pneumoniae_CJV05413.1

MKSKRMEFHNKFLIVSAMLAVISWLSLGVVSFPMLAGTLGISTKAAATVVNLISAYSTVTAVISIVGAITGVGSIGSGIAATVLYILKKKGAAKAALW

>Streptococcus-pneumoniae_CKA82236.1

MKSKRMEFHNKFLIVSAMLAVISWLSLGVVSFPMLAGTLGISTKAAATVVNLISAYSTVTAVISIVGAITGVGSIGSGIAATVLYILKKKGAAKAALW

>Streptococcus-pneumoniae_CKA72830.1

MKSKRMEFHNKFLIVSAMLAVISWLSLGVVSFPMLAGTLGISTKAAATVVNLISAYSTVTAVISIVGAITGVGSIGSGIAATVLYILKKKGAAKAALW

>Streptococcus-pneumoniae_CKB67904.1

MKSKRMEFHNKFLIVSAMLAVISWLSLGVVSFPMLAGTLGISTKAAATVVNLISAYSTVTAVISIVGAITGVGSIGSGIAATVLYILKKKGAAKAALW

>Streptococcus-pneumoniae_CJZ85273.1

MKSKRMEFHNKFLIVSAMLAVISWLSLGVVSFPMLAGTLGISTKAAATVVNLISAYSTVTAVISIVGAITGVGSIGSGIAATVLYILKKKGAAKAALW

>Streptococcus-pneumoniae_CKB90923.1

MKSKRMEFHNKFLIVSAMLAVISWLSLGVVSFPMLAGTLGISTKAAATVVNLISAYSTVTAVISIVGAITGVGSIGSGIAATVLYILKKKGAAKAALW

>Streptococcus-pneumoniae_CKE31828.1

MKSKRMEFHNKFLIVSAMLAVISWLSLGVVSFPMLAGTLGISTKAAATVVNLISAYSTVTAVISIVGAITGVGSIGSGIAATVLYILKKKGAAKAALW

>Streptococcus-pneumoniae_CKA33302.1

MKSKRMEFHNKFLIVSAMLAVISWLSLGVVSFPMLAGTLGISTKAAATVVNLISAYSTVTAVISIVGAITGVGSIGSGIAATVLYILKKKGAAKAALW

>Streptococcus-pneumoniae_CKD25944.1

MKSKRMEFHNKFLIVSAMLAVISWLSLGVVSFPMLAGTLGISTKAAATVVNLISAYSTVTAVISIVGAITGVGSIGSGIAATVLYILKKKGAAKAALW

>Streptococcus-pneumoniae_CKF45823.1

MKSKRMEFHNKFLIVSAMLAVISWLSLGVVSFPMLAGTLGISTKAAATVVNLISAYSTVTAVISIVGAITGVGSIGSGIAATVLYILKKKGAAKAALW

>Streptococcus-pneumoniae_CJX33973.1

MKSKRMEFHNKFLIVSAMLAVISWLSLGVVSFPMLAGTLGISTKAAATVVNLISAYSTVTAVISIVGAITGVGSIGSGIAATVLYILKKKGAAKAALW

>Streptococcus-pneumoniae_CKG29042.1

MKSKRMEFHNKFLIVSAMLAVISWLSLGVVSFPMLAGTLGISTKAAATVVNLISAYSTVTAVISIVGAITGVGSIGSGIAATVLYILKKKGAAKAALW

>Streptococcus-pneumoniae_CKC08871.1

MKSKRMEFHNKFLIVSAMLAVISWLSLGVVSFPMLAGTLGISTKAAATVVNLISAYSTVTAVISIVGAITGVGSIGSGIAATVLYILKKKGAAKAALW

>Streptococcus-pneumoniae_CKD67443.1

MKSKRMEFHNKFLIVSAMLAVISWLSLGVVSFPMLAGTLGISTKAAATVVNLISAYSTVTAVISIVGAITGVGSIGSGIAATVLYILKKKGAAKAALW

>Streptococcus-pneumoniae_CKI49304.1

MKSKRMEFHNKFLIVSAMLAVISWLSLGVVSFPMLAGTLGISTKAAATVVNLISAYSTVTAVISIVGAITGVGSIGSGIAATVLYILKKKGAAKAALW

>Streptococcus-pneumoniae_CJY96967.1

MKSKRMEFHNKFLIVSAMLAVISWLSLGVVSFPMLAGTLGISTKAAATVVNLISAYSTVTAVISIVGAITGVGSIGSGIAATVLYILKKKGAAKAALW

>Streptococcus-pneumoniae_CKE55268.1

MKSKRMEFHNKFLIVSAMLAVISWLSLGVVSFPMLAGTLGISTKAAATVVNLISAYSTVTAVISIVGAITGVGSIGSGIAATVLYILKKKGAAKAALW

>Streptococcus-pneumoniae_CKD61844.1

MKSKRMEFHNKFLIVSAMLAVISWLSLGVVSFPMLAGTLGISTKAAATVVNLISAYSTVTAVISIVGAITGVGSIGSGIAATVLYILKKKGAAKAALW

>Streptococcus-pneumoniae_CKH68374.1

MKSKRMEFHNKFLIVSAMLAVISWLSLGVVSFPMLAGTLGISTKAAATVVNLISAYSTVTAVISIVGAITGVGSIGSGIAATVLYILKKKGAAKAALW

>Streptococcus-pneumoniae_CKA39393.1

MKSKRMEFHNKFLIVSAMLAVISWLSLGVVSFPMLAGTLGISTKAAATVVNLISAYSTVTAVISIVGAITGVGSIGSGIAATVLYILKKKGAAKAALW

>Streptococcus-pneumoniae_CJZ50746.1

MKSKRMEFHNKFLIVSAMLAVISWLSLGVVSFPMLAGTLGISTKAAATVVNLISAYSTVTAVISIVGAITGVGSIGSGIAATVLYILKKKGAAKAALW

>Streptococcus-pneumoniae_CKC36958.1

MKSKRMEFHNKFLIVSAMLAVISWLSLGVVSFPMLAGTLGISTKAAATVVNLISAYSTVTAVISIVGAITGVGSIGSGIAATVLYILKKKGAAKAALW

>Streptococcus-pneumoniae_CKC90752.1

MKSKRMEFHNKFLIVSAMLAVISWLSLGVVSFPMLAGTLGISTKAAATVVNLISAYSTVTAVISIVGAITGVGSIGSGIAATVLYILKKKGAAKAALW

>Streptococcus-pneumoniae_CKG65115.1

MKSKRMEFHNKFLIVSAMLAVISWLSLGVVSFPMLAGTLGISTKAAATVVNLISAYSTVTAVISIVGAITGVGSIGSGIAATVLYILKKKGAAKAALW

>Streptococcus-pneumoniae_CJU74231.1

MKSKRMEFHNKFLIVSAMLAVISWLSLGVVSFPMLAGTLGISTKAAATVVNLISAYSTVTAVISIVGAITGVGSIGSGIAATVLYILKKKGAAKAALW

>Streptococcus-pneumoniae_CKB07276.1

MKSKRMEFHNKFLIVSAMLAVISWLSLGVVSFPMLAGTLGISTKAAATVVNLISAYSTVTAVISIVGAITGVGSIGSGIAATVLYILKKKGAAKAALW

>Streptococcus-pneumoniae_CKC11370.1

MKSKRMEFHNKFLIVSAMLAVISWLSLGVVSFPMLAGTLGISTKAAATVVNLISAYSTVTAVISIVGAITGVGSIGSGIAATVLYILKKKGAAKAALW

>Streptococcus-pneumoniae_CJX10375.1

MKSKRMEFHNKFLIVSAMLAVISWLSLGVVSFPMLAGTLGISTKAAATVVNLISAYSTVTAVISIVGAITGVGSIGSGIAATVLYILKKKGAAKAALW

>Streptococcus-pneumoniae_CKC57891.1

MKSKRMEFHNKFLIVSAMLAVISWLSLGVVSFPMLAGTLGISTKAAATVVNLISAYSTVTAVISIVGAITGVGSIGSGIAATVLYILKKKGAAKAALW

>Streptococcus-pneumoniae_CKC29741.1

MKSKRMEFHNKFLIVSAMLAVISWLSLGVVSFPMLAGTLGISTKAAATVVNLISAYSTVTAVISIVGAITGVGSIGSGIAATVLYILKKKGAAKAALW

>Streptococcus-pneumoniae_CJV45265.1

MKSKRMEFHNKFLIVSAMLAVISWLSLGVVSFPMLAGTLGISTKAAATVVNLISAYSTVTAVISIVGAITGVGSIGSGIAATVLYILKKKGAAKAALW

>Streptococcus-pneumoniae_CKA38419.1

MKSKRMEFHNKFLIVSAMLAVISWLSLGVVSFPMLAGTLGISTKAAATVVNLISAYSTVTAVISIVGAITGVGSIGSGIAATVLYILKKKGAAKAALW

>Streptococcus-pneumoniae_CJW27488.1

MKSKRMEFHNKFLIVSAMLAVISWLSLGVVSFPMLAGTLGISTKAAATVVNLISAYSTVTAVISIVGAITGVGSIGSGIAATVLYILKKKGAAKAALW

>Streptococcus-pneumoniae_CKE21725.1

MKSKRMEFHNKFLIVSAMLAVISWLSLGVVSFPMLAGTLGISTKAAATVVNLISAYSTVTAVISIVGAITGVGSIGSGIAATVLYILKKKGAAKAALW

>Streptococcus-pneumoniae_CKC40214.1

MKSKRMEFHNKFLIVSAMLAVISWLSLGVVSFPMLAGTLGISTKAAATVVNLISAYSTVTAVISIVGAITGVGSIGSGIAATVLYILKKKGAAKAALW

>Streptococcus-pneumoniae_CKG33763.1

MKSKRMEFHNKFLIVSAMLAVISWLSLGVVSFPMLAGTLGISTKAAATVVNLISAYSTVTAVISIVGAITGVGSIGSGIAATVLYILKKKGAAKAALW

>Streptococcus-pneumoniae_CKD79728.1

MKSKRMEFHNKFLIVSAMLAVISWLSLGVVSFPMLAGTLGISTKAAATVVNLISAYSTVTAVISIVGAITGVGSIGSGIAATVLYILKKKGAAKAALW

>Streptococcus-pneumoniae_CKG54291.1

MKSKRMEFHNKFLIVSAMLAVISWLSLGVVSFPMLAGTLGISTKAAATVVNLISAYSTVTAVISIVGAITGVGSIGSGIAATVLYILKKKGAAKAALW

>Streptococcus-pneumoniae_CJY34106.1

MKSKRMEFHNKFLIVSAMLAVISWLSLGVVSFPMLAGTLGISTKAAATVVNLISAYSTVTAVISIVGAITGVGSIGSGIAATVLYILKKKGAAKAALW

>Streptococcus-pneumoniae_CJV03486.1

MKSKRMEFHNKFLIVSAMLAVISWLSLGVVSFPMLAGTLGISTKAAATVVNLISAYSTVTAVISIVGAITGVGSIGSGIAATVLYILKKKGAAKAALW

>Streptococcus-pneumoniae_CJV42874.1

MKSKRMEFHNKFLIVSAMLAVISWLSLGVVSFPMLAGTLGISTKAAATVVNLISAYSTVTAVISIVGAITGVGSIGSGIAATVLYILKKKGAAKAALW

>Streptococcus-pneumoniae_CKE68105.1

MKSKRMEFHNKFLIVSAMLAVISWLSLGVVSFPMLAGTLGISTKAAATVVNLISAYSTVTAVISIVGAITGVGSIGSGIAATVLYILKKKGAAKAALW

>Streptococcus-pneumoniae_CKA47380.1

MKSKRMEFHNKFLIVSAMLAVISWLSLGVVSFPMLAGTLGISTKAAATVVNLISAYSTVTAVISIVGAITGVGSIGSGIAATVLYILKKKGAAKAALW

>Streptococcus-pneumoniae_CJZ87398.1

MKSKRMEFHNKFLIVSAMLAVISWLSLGVVSFPMLAGTLGISTKAAATVVNLISAYSTVTAVISIVGAITGVGSIGSGIAATVLYILKKKGAAKAALW

>Streptococcus-pneumoniae_CJW51759.1

MKSKRMEFHNKFLIVSAMLAVISWLSLGVVSFPMLAGTLGISTKAAATVVNLISAYSTVTAVISIVGAITGVGSIGSGIAATVLYILKKKGAAKAALW

>Streptococcus-pneumoniae_CJU32732.1

MKSKRMEFHNKFLIVSAMLAVISWLSLGVVSFPMLAGTLGISTKAAATVVNLISAYSTVTAVISIVGAITGVGSIGSGIAATVLYILKKKGAAKAALW

>Streptococcus-pneumoniae_CJV25803.1

MKSKRMEFHNKFLIVSAMLAVISWLSLGVVSFPMLAGTLGISTKAAATVVNLISAYSTVTAVISIVGAITGVGSIGSGIAATVLYILKKKGAAKAALW

>Streptococcus-pneumoniae_CKD81584.1

MKSKRMEFHNKFLIVSAMLAVISWLSLGVVSFPMLAGTLGISTKAAATVVNLISAYSTVTAVISIVGAITGVGSIGSGIAATVLYILKKKGAAKAALW

>Streptococcus-pneumoniae_CKD89354.1

MKSKRMEFHNKFLIVSAMLAVISWLSLGVVSFPMLAGTLGISTKAAATVVNLISAYSTVTAVISIVGAITGVGSIGSGIAATVLYILKKKGAAKAALW

>Streptococcus-pneumoniae_CJX91050.1

MKSKRMEFHNKFLIVSAMLAVISWLSLGVVSFPMLAGTLGISTKAAATVVNLISAYSTVTAVISIVGAITGVGSIGSGIAATVLYILKKKGAAKAALW

>Streptococcus-pneumoniae_CJW84093.1

MKSKRMEFHNKFLIVSAMLAVISWLSLGVVSFPMLAGTLGISTKAAATVVNLISAYSTVTAVISIVGAITGVGSIGSGIAATVLYILKKKGAAKAALW

>Streptococcus-pneumoniae_CKB01128.1

MKSKRMEFHNKFLIVSAMLAVISWLSLGVVSFPMLAGTLGISTKAAATVVNLISAYSTVTAVISIVGAITGVGSIGSGIAATVLYILKKKGAAKAALW

>Streptococcus-pneumoniae_CKA11153.1

MKSKRMEFHNKFLIVSAMLAVISWLSLGVVSFPMLAGTLGISTKAAATVVNLISAYSTVTAVISIVGAITGVGSIGSGIAATVLYILKKKGAAKAALW

>Streptococcus-pneumoniae_CJW97861.1

MKSKRMEFHNKFLIVSAMLAVISWLSLGVVSFPMLAGTLGISTKAAATVVNLISAYSTVTAVISIVGAITGVGSIGSGIAATVLYILKKKGAAKAALW

>Streptococcus-pneumoniae_CJZ75153.1

MKSKRMEFHNKFLIVSAMLAVISWLSLGVVSFPMLAGTLGISTKAAATVVNLISAYSTVTAVISIVGAITGVGSIGSGIAATVLYILKKKGAAKAALW

>Streptococcus-pneumoniae_CKG71495.1

MKSKRMEFHNKFLIVSAMLAVISWLSLGVVSFPMLAGTLGISTKAAATVVNLISAYSTVTAVISIVGAITGVGSIGSGIAATVLYILKKKGAAKAALW

>Streptococcus-pneumoniae_CJZ14670.1

MKSKRMEFHNKFLIVSAMLAVISWLSLGVVSFPMLAGTLGISTKAAATVVNLISAYSTVTAVISIVGAITGVGSIGSGIAATVLYILKKKGAAKAALW

>Streptococcus-pneumoniae_CKA60083.1

MKSKRMEFHNKFLIVSAMLAVISWLSLGVVSFPMLAGTLGISTKAAATVVNLISAYSTVTAVISIVGAITGVGSIGSGIAATVLYILKKKGAAKAALW

>Streptococcus-pneumoniae_CKE64882.1

MKSKRMEFHNKFLIVSAMLAVISWLSLGVVSFPMLAGTLGISTKAAATVVNLISAYSTVTAVISIVGAITGVGSIGSGIAATVLYILKKKGAAKAALW

>Streptococcus-pneumoniae_CJW57933.1

MKSKRMEFHNKFLIVSAMLAVISWLSLGVVSFPMLAGTLGISTKAAATVVNLISAYSTVTAVISIVGAITGVGSIGSGIAATVLYILKKKGAAKAALW

>Streptococcus-pneumoniae_CKA57847.1

MKSKRMEFHNKFLIVSAMLAVISWLSLGVVSFPMLAGTLGISTKAAATVVNLISAYSTVTAVISIVGAITGVGSIGSGIAATVLYILKKKGAAKAALW

>Streptococcus-pneumoniae_CKA07289.1

MKSKRMEFHNKFLIVSAMLAVISWLSLGVVSFPMLAGTLGISTKAAATVVNLISAYSTVTAVISIVGAITGVGSIGSGIAATVLYILKKKGAAKAALW

>Streptococcus-pneumoniae_CKA76674.1

MKSKRMEFHNKFLIVSAMLAVISWLSLGVVSFPMLAGTLGISTKAAATVVNLISAYSTVTAVISIVGAITGVGSIGSGIAATVLYILKKKGAAKAALW

>Streptococcus-pneumoniae_CKA02677.1

MKSKRMEFHNKFLIVSAMLAVISWLSLGVVSFPMLAGTLGISTKAAATVVNLISAYSTVTAVISIVGAITGVGSIGSGIAATVLYILKKKGAAKAALW

>Streptococcus-pneumoniae_CJV03491.1

MKSKRMEFHNKFLIVSAMLAVISWLSLGVVSFPMLAGTLGISTKAAATVVNLISAYSTVTAVISIVGAITGVGSIGSGIAATVLYILKKKGAAKAALW

>Streptococcus-pneumoniae_CJT91676.1

MKSKRMEFHNKFLIVSAMLAVISWLSLGVVSFPMLAGTLGISTKAAATVVNLISAYSTVTAVISIVGAITGVGSIGSGIAATVLYILKKKGAAKAALW

>Streptococcus-pneumoniae_CJV70466.1

MKSKRMEFHNKFLIVSAMLAVISWLSLGVVSFPMLAGTLGISTKAAATVVNLISAYSTVTAVISIVGAITGVGSIGSGIAATVLYILKKKGAAKAALW

>Streptococcus-pneumoniae_CJV74461.1

MKSKRMEFHNKFLIVSAMLAVISWLSLGVVSFPMLAGTLGISTKAAATVVNLISAYSTVTAVISIVGAITGVGSIGSGIAATVLYILKKKGAAKAALW

>Streptococcus-pneumoniae_CKG24988.1

MKSKRMEFHNKFLIVSAMLAVISWLSLGVVSFPMLAGTLGISTKAAATVVNLISAYSTVTAVISIVGAITGVGSIGSGIAATVLYILKKKGAAKAALW

>Streptococcus-pneumoniae_CJW91052.1

MKSKRMEFHNKFLIVSAMLAVISWLSLGVVSFPMLAGTLGISTKAAATVVNLISAYSTVTAVISIVGAITGVGSIGSGIAATVLYILKKKGAAKAALW

>Streptococcus-pneumoniae_CJX72841.1

MKSKRMEFHNKFLIVSAMLAVISWLSLGVVSFPMLAGTLGISTKAAATVVNLISAYSTVTAVISIVGAITGVGSIGSGIAATVLYILKKKGAAKAALW

>Streptococcus-pneumoniae_CJX40889.1

MKSKRMEFHNKFLIVSAMLAVISWLSLGVVSFPMLAGTLGISTKAAATVVNLISAYSTVTAVISIVGAITGVGSIGSGIAATVLYILKKKGAAKAALW

>Streptococcus-pneumoniae_CJY76549.1

MKSKRMEFHNKFLIVSAMLAVISWLSLGVVSFPMLAGTLGISTKAAATVVNLISAYSTVTAVISIVGAITGVGSIGSGIAATVLYILKKKGAAKAALW

>Streptococcus-pneumoniae_CKD27263.1

MKSKRMEFHNKFLIVSAMLAVISWLSLGVVSFPMLAGTLGISTKAAATVVNLISAYSTVTAVISIVGAITGVGSIGSGIAATVLYILKKKGAAKAALW

>Streptococcus-pneumoniae_CJY89082.1

MKSKRMEFHNKFLIVSAMLAVISWLSLGVVSFPMLAGTLGISTKAAATVVNLISAYSTVTAVISIVGAITGVGSIGSGIAATVLYILKKKGAAKAALW

>Streptococcus-pneumoniae_CKA02399.1

MKSKRMEFHNKFLIVSAMLAVISWLSLGVVSFPMLAGTLGISTKAAATVVNLISAYSTVTAVISIVGAITGVGSIGSGIAATVLYILKKKGAAKAALW

>Streptococcus-pneumoniae_CKD56659.1

MKSKRMEFHNKFLIVSAMLAVISWLSLGVVSFPMLAGTLGISTKAAATVVNLISAYSTVTAVISIVGAITGVGSIGSGIAATVLYILKKKGAAKAALW

>Streptococcus-pneumoniae_CKI37676.1

MKSKRMEFHNKFLIVSAMLAVISWLSLGVVSFPMLAGTLGISTKAAATVVNLISAYSTVTAVISIVGAITGVGSIGSGIAATVLYILKKKGAAKAALW

>Streptococcus-pneumoniae_CJU01245.1

MKSKRMEFHNKFLIVSAMLAVISWLSLGVVSFPMLAGTLGISTKAAATVVNLISAYSTVTAVISIVGAITGVGSIGSGIAATVLYILKKKGAAKAALW

>Streptococcus-pneumoniae_CJZ28937.1

MKSKRMEFHNKFLIVSAMLAVISWLSLGVVSFPMLAGTLGISTKAAATVVNLISAYSTVTAVISIVGAITGVGSIGSGIAATVLYILKKKGAAKAALW

>Streptococcus-pneumoniae_CKG73576.1

MKSKRMEFHNKFLIVSAMLAVISWLSLGVVSFPMLAGTLGISTKAAATVVNLISAYSTVTAVISIVGAITGVGSIGSGIAATVLYILKKKGAAKAALW

>Streptococcus-pneumoniae_CKH53294.1

MKSKRMEFHNKFLIVSAMLAVISWLSLGVVSFPMLAGTLGISTKAAATVVNLISAYSTVTAVISIVGAITGVGSIGSGIAATVLYILKKKGAAKAALW

>Streptococcus-pneumoniae_CKG42960.1

MKSKRMEFHNKFLIVSAMLAVISWLSLGVVSFPMLAGTLGISTKAAATVVNLISAYSTVTAVISIVGAITGVGSIGSGIAATVLYILKKKGAAKAALW

>Streptococcus-pneumoniae_CKI73855.1

MKSKRMEFHNKFLIVSAMLAVISWLSLGVVSFPMLAGTLGISTKAAATVVNLISAYSTVTAVISIVGAITGVGSIGSGIAATVLYILKKKGAAKAALW

>Streptococcus-pneumoniae_CJU02921.1

MKSKRMEFHNKFLIVSAMLAVISWLSLGVVSFPMLAGTLGISTKAAATVVNLISAYSTVTAVISIVGAITGVGSIGSGIAATVLYILKKKGAAKAALW

>Streptococcus-pneumoniae_CKC02294.1

MKSKRMEFHNKFLIVSAMLAVISWLSLGVVSFPMLAGTLGISTKAAATVVNLISAYSTVTAVISIVGAITGVGSIGSGIAATVLYILKKKGAAKAALW

>Streptococcus-pneumoniae_CKH16308.1

MKSKRMEFHNKFLIVSAMLAVISWLSLGVVSFPMLAGTLGISTKAAATVVNLISAYSTVTAVISIVGAITGVGSIGSGIAATVLYILKKKGAAKAALW

>Streptococcus-pneumoniae_CJX73388.1

MKSKRMEFHNKFLIVSAMLAVISWLSLGVVSFPMLAGTLGISTKAAATVVNLISAYSTVTAVISIVGAITGVGSIGSGIAATVLYILKKKGAAKAALW

>Streptococcus-pneumoniae_CKG54506.1

MKSKRMEFHNKFLIVSAMLAVISWLSLGVVSFPMLAGTLGISTKAAATVVNLISAYSTVTAVISIVGAITGVGSIGSGIAATVLYILKKKGAAKAALW

>Streptococcus-pneumoniae_CKC88193.1

MKSKRMEFHNKFLIVSAMLAVISWLSLGVVSFPMLAGTLGISTKAAATVVNLISAYSTVTAVISIVGAITGVGSIGSGIAATVLYILKKKGAAKAALW

>Streptococcus-pneumoniae_CKH80006.1

MKSKRMEFHNKFLIVSAMLAVISWLSLGVVSFPMLAGTLGISTKAAATVVNLISAYSTVTAVISIVGAITGVGSIGSGIAATVLYILKKKGAAKAALW

>Streptococcus-pneumoniae_CKH78614.1

MKSKRMEFHNKFLIVSAMLAVISWLSLGVVSFPMLAGTLGISTKAAATVVNLISAYSTVTAVISIVGAITGVGSIGSGIAATVLYILKKKGAAKAALW

>Streptococcus-pneumoniae_CKA45989.1

MKSKRMEFHNKFLIVSAMLAVISWLSLGVVSFPMLAGTLGISTKAAATVVNLISAYSTVTAVISIVGAITGVGSIGSGIAATVLYILKKKGAAKAALW

>Streptococcus-pneumoniae_CKA05894.1

MKSKRMEFHNKFLIVSAMLAVISWLSLGVVSFPMLAGTLGISTKAAATVVNLISAYSTVTAVISIVGAITGVGSIGSGIAATVLYILKKKGAAKAALW

>Streptococcus-pneumoniae_CKF82905.1

MKSKRMEFHNKFLIVSAMLAVISWLSLGVVSFPMLAGTLGISTKAAATVVNLISAYSTVTAVISIVGAITGVGSIGSGIAATVLYILKKKGAAKAALW

>Streptococcus-pneumoniae_CJZ45346.1

MKSKRMEFHNKFLIVSAMLAVISWLSLGVVSFPMLAGTLGISTKAAATVVNLISAYSTVTAVISIVGAITGVGSIGSGIAATVLYILKKKGAAKAALW

>Streptococcus-pneumoniae_CKJ03297.1

MKSKRMEFHNKFLIVSAMLAVISWLSLGVVSFPMLAGTLGISTKAAATVVNLISAYSTVTAVISIVGAITGVGSIGSGIAATVLYILKKKGAAKAALW

>Streptococcus-pneumoniae_CKJ49634.1

MKSKRMEFHNKFLIVSAMLAVISWLSLGVVSFPMLAGTLGISTKAAATVVNLISAYSTVTAVISIVGAITGVGSIGSGIAATVLYILKKKGAAKAALW

>Streptococcus-pneumoniae_CKE76947.1

MKSKRMEFHNKFLIVSAMLAVISWLSLGVVSFPMLAGTLGISTKAAATVVNLISAYSTVTAVISIVGAITGVGSIGSGIAATVLYILKKKGAAKAALW

>Streptococcus-pneumoniae_CKL42937.1

MKSKRMEFHNKFLIVSAMLAVISWLSLGVVSFPMLAGTLGISTKAAATVVNLISAYSTVTAVISIVGAITGVGSIGSGIAATVLYILKKKGAAKAALW

>Streptococcus-pneumoniae_CKI96416.1

MKSKRMEFHNKFLIVSAMLAVISWLSLGVVSFPMLAGTLGISTKAAATVVNLISAYSTVTAVISIVGAITGVGSIGSGIAATVLYILKKKGAAKAALW

>Streptococcus-pneumoniae_CKH70834.1

MKSKRMEFHNKFLIVSAMLAVISWLSLGVVSFPMLAGTLGISTKAAATVVNLISAYSTVTAVISIVGAITGVGSIGSGIAATVLYILKKKGAAKAALW

>Streptococcus-pneumoniae_CJU07373.1

MKSKRMEFHNKFLIVSAMLAVISWLSLGVVSFPMLAGTLGISTKAAATVVNLISAYSTVTAVISIVGAITGVGSIGSGIAATVLYILKKKGAAKAALW

>Streptococcus-pneumoniae_CKJ01245.1

MKSKRMEFHNKFLIVSAMLAVISWLSLGVVSFPMLAGTLGISTKAAATVVNLISAYSTVTAVISIVGAITGVGSIGSGIAATVLYILKKKGAAKAALW

>Streptococcus-pneumoniae_CJZ24343.1

MKSKRMEFHNKFLIVSAMLAVISWLSLGVVSFPMLAGTLGISTKAAATVVNLISAYSTVTAVISIVGAITGVGSIGSGIAATVLYILKKKGAAKAALW

>Streptococcus-pneumoniae_CJY42173.1

MKSKRMEFHNKFLIVSAMLAVISWLSLGVVSFPMLAGTLGISTKAAATVVNLISAYSTVTAVISIVGAITGVGSIGSGIAATVLYILKKKGAAKAALW

>Streptococcus-pneumoniae_CKJ48287.1

MKSKRMEFHNKFLIVSAMLAVISWLSLGVVSFPMLAGTLGISTKAAATVVNLISAYSTVTAVISIVGAITGVGSIGSGIAATVLYILKKKGAAKAALW

>Streptococcus-pneumoniae_CJV18630.1

MKSKRMEFHNKFLIVSAMLAVISWLSLGVVSFPMLAGTLGISTKAAATVVNLISAYSTVTAVISIVGAITGVGSIGSGIAATVLYILKKKGAAKAALW

>Streptococcus-pneumoniae_CKH56891.1

MKSKRMEFHNKFLIVSAMLAVISWLSLGVVSFPMLAGTLGISTKAAATVVNLISAYSTVTAVISIVGAITGVGSIGSGIAATVLYILKKKGAAKAALW

>Streptococcus-pneumoniae_CKI75351.1

MKSKRMEFHNKFLIVSAMLAVISWLSLGVVSFPMLAGTLGISTKAAATVVNLISAYSTVTAVISIVGAITGVGSIGSGIAATVLYILKKKGAAKAALW

>Streptococcus-pneumoniae_CKI97750.1

MKSKRMEFHNKFLIVSAMLAVISWLSLGVVSFPMLAGTLGISTKAAATVVNLISAYSTVTAVISIVGAITGVGSIGSGIAATVLYILKKKGAAKAALW

>Streptococcus-pneumoniae_CKI63706.1

MKSKRMEFHNKFLIVSAMLAVISWLSLGVVSFPMLAGTLGISTKAAATVVNLISAYSTVTAVISIVGAITGVGSIGSGIAATVLYILKKKGAAKAALW

>Streptococcus-pneumoniae_CJU91744.1

MKSKRMEFHNKFLIVSAMLAVISWLSLGVVSFPMLAGTLGISTKAAATVVNLISAYSTVTAVISIVGAITGVGSIGSGIAATVLYILKKKGAAKAALW

>Streptococcus-pneumoniae_CKJ50508.1

MKSKRMEFHNKFLIVSAMLAVISWLSLGVVSFPMLAGTLGISTKAAATVVNLISAYSTVTAVISIVGAITGVGSIGSGIAATVLYILKKKGAAKAALW

>Streptococcus-pneumoniae_CKI68768.1

MKSKRMEFHNKFLIVSAMLAVISWLSLGVVSFPMLAGTLGISTKAAATVVNLISAYSTVTAVISIVGAITGVGSIGSGIAATVLYILKKKGAAKAALW

>Streptococcus-pneumoniae_CKF82157.1

MKSKRMEFHNKFLIVSAMLAVISWLSLGVVSFPMLAGTLGISTKAAATVVNLISAYSTVTAVISIVGAITGVGSIGSGIAATVLYILKKKGAAKAALW

>Streptococcus-pneumoniae_CKH29720.1

MKSKRMEFHNKFLIVSAMLAVISWLSLGVVSFPMLAGTLGISTKAAATVVNLISAYSTVTAVISIVGAITGVGSIGSGIAATVLYILKKKGAAKAALW

>Streptococcus-pneumoniae_CJZ66795.1

MKSKRMEFHNKFLIVSAMLAVISWLSLGVVSFPMLAGTLGISTKAAATVVNLISAYSTVTAVISIVGAITGVGSIGSGIAATVLYILKKKGAAKAALW

>Streptococcus-pneumoniae_CKI82847.1

MKSKRMEFHNKFLIVSAMLAVISWLSLGVVSFPMLAGTLGISTKAAATVVNLISAYSTVTAVISIVGAITGVGSIGSGIAATVLYILKKKGAAKAALW

>Streptococcus-pneumoniae_CKL50054.1

MKSKRMEFHNKFLIVSAMLAVISWLSLGVVSFPMLAGTLGISTKAAATVVNLISAYSTVTAVISIVGAITGVGSIGSGIAATVLYILKKKGAAKAALW

>Streptococcus-pneumoniae_CKI49978.1

MKSKRMEFHNKFLIVSAMLAVISWLSLGVVSFPMLAGTLGISTKAAATVVNLISAYSTVTAVISIVGAITGVGSIGSGIAATVLYILKKKGAAKAALW

>Streptococcus-pneumoniae_CKI59564.1

MKSKRMEFHNKFLIVSAMLAVISWLSLGVVSFPMLAGTLGISTKAAATVVNLISAYSTVTAVISIVGAITGVGSIGSGIAATVLYILKKKGAAKAALW

>Streptococcus-pneumoniae_CKL59343.1

MKSKRMEFHNKFLIVSAMLAVISWLSLGVVSFPMLAGTLGISTKAAATVVNLISAYSTVTAVISIVGAITGVGSIGSGIAATVLYILKKKGAAKAALW

>Streptococcus-pneumoniae_CKG32545.1

MKSKRMEFHNKFLIVSAMLAVISWLSLGVVSFPMLAGTLGISTKAAATVVNLISAYSTVTAVISIVGAITGVGSIGSGIAATVLYILKKKGAAKAALW

>Streptococcus-pneumoniae_CKI53562.1

MKSKRMEFHNKFLIVSAMLAVISWLSLGVVSFPMLAGTLGISTKAAATVVNLISAYSTVTAVISIVGAITGVGSIGSGIAATVLYILKKKGAAKAALW

>Streptococcus-pneumoniae_CKH50936.1

MKSKRMEFHNKFLIVSAMLAVISWLSLGVVSFPMLAGTLGISTKAAATVVNLISAYSTVTAVISIVGAITGVGSIGSGIAATVLYILKKKGAAKAALW

>Streptococcus-pneumoniae_CKI81463.1

MKSKRMEFHNKFLIVSAMLAVISWLSLGVVSFPMLAGTLGISTKAAATVVNLISAYSTVTAVISIVGAITGVGSIGSGIAATVLYILKKKGAAKAALW

>Streptococcus-pneumoniae_CKJ11449.1

MKSKRMEFHNKFLIVSAMLAVISWLSLGVVSFPMLAGTLGISTKAAATVVNLISAYSTVTAVISIVGAITGVGSIGSGIAATVLYILKKKGAAKAALW

>Streptococcus-pneumoniae_CKI63289.1

MKSKRMEFHNKFLIVSAMLAVISWLSLGVVSFPMLAGTLGISTKAAATVVNLISAYSTVTAVISIVGAITGVGSIGSGIAATVLYILKKKGAAKAALW

>Streptococcus-pneumoniae_CKJ62697.1

MKSKRMEFHNKFLIVSAMLAVISWLSLGVVSFPMLAGTLGISTKAAATVVNLISAYSTVTAVISIVGAITGVGSIGSGIAATVLYILKKKGAAKAALW

>Streptococcus-pneumoniae_CKI63837.1

MKSKRMEFHNKFLIVSAMLAVISWLSLGVVSFPMLAGTLGISTKAAATVVNLISAYSTVTAVISIVGAITGVGSIGSGIAATVLYILKKKGAAKAALW

>Streptococcus-pneumoniae_CKH37438.1

MKSKRMEFHNKFLIVSAMLAVISWLSLGVVSFPMLAGTLGISTKAAATVVNLISAYSTVTAVISIVGAITGVGSIGSGIAATVLYILKKKGAAKAALW

>Streptococcus-pneumoniae_CKH63526.1

MKSKRMEFHNKFLIVSAMLAVISWLSLGVVSFPMLAGTLGISTKAAATVVNLISAYSTVTAVISIVGAITGVGSIGSGIAATVLYILKKKGAAKAALW

>Streptococcus-pneumoniae_CKH35803.1

MKSKRMEFHNKFLIVSAMLAVISWLSLGVVSFPMLAGTLGISTKAAATVVNLISAYSTVTAVISIVGAITGVGSIGSGIAATVLYILKKKGAAKAALW

>Streptococcus-pneumoniae_CKH76572.1

MKSKRMEFHNKFLIVSAMLAVISWLSLGVVSFPMLAGTLGISTKAAATVVNLISAYSTVTAVISIVGAITGVGSIGSGIAATVLYILKKKGAAKAALW

>Streptococcus-pneumoniae_CKH80815.1

MKSKRMEFHNKFLIVSAMLAVISWLSLGVVSFPMLAGTLGISTKAAATVVNLISAYSTVTAVISIVGAITGVGSIGSGIAATVLYILKKKGAAKAALW

>Streptococcus-pneumoniae_CKJ35161.1

MKSKRMEFHNKFLIVSAMLAVISWLSLGVVSFPMLAGTLGISTKAAATVVNLISAYSTVTAVISIVGAITGVGSIGSGIAATVLYILKKKGAAKAALW

>Streptococcus-pneumoniae_CJU23622.1

MKSKRMEFHNKFLIVSAMLAVISWLSLGVVSFPMLAGTLGISTKAAATVVNLISAYSTVTAVISIVGAITGVGSIGSGIAATVLYILKKKGAAKAALW

>Streptococcus-pneumoniae_CKJ10190.1

MKSKRMEFHNKFLIVSAMLAVISWLSLGVVSFPMLAGTLGISTKAAATVVNLISAYSTVTAVISIVGAITGVGSIGSGIAATVLYILKKKGAAKAALW

>Streptococcus-pneumoniae_CKG79545.1

MKSKRMEFHNKFLIVSAMLAVISWLSLGVVSFPMLAGTLGISTKAAATVVNLISAYSTVTAVISIVGAITGVGSIGSGIAATVLYILKKKGAAKAALW

>Streptococcus-pneumoniae_CKJ18553.1

MKSKRMEFHNKFLIVSAMLAVISWLSLGVVSFPMLAGTLGISTKAAATVVNLISAYSTVTAVISIVGAITGVGSIGSGIAATVLYILKKKGAAKAALW

>Streptococcus-pneumoniae_CKI89594.1

MKSKRMEFHNKFLIVSAMLAVISWLSLGVVSFPMLAGTLGISTKAAATVVNLISAYSTVTAVISIVGAITGVGSIGSGIAATVLYILKKKGAAKAALW

>Streptococcus-pneumoniae_CJU30923.1

MKSKRMEFHNKFLIVSAMLAVISWLSLGVVSFPMLAGTLGISTKAAATVVNLISAYSTVTAVISIVGAITGVGSIGSGIAATVLYILKKKGAAKAALW

>Streptococcus-pneumoniae_CJY46521.1

MKSKRMEFHNKFLIVSAMLAVISWLSLGVVSFPMLAGTLGISTKAAATVVNLISAYSTVTAVISIVGAITGVGSIGSGIAATVLYILKKKGAAKAALW

>Streptococcus-pneumoniae_CKE42707.1

MKSKRMEFHNKFLIVSAMLAVISWLSLGVVSFPMLAGTLGISTKAAATVVNLISAYSTVTAVISIVGAITGVGSIGSGIAATVLYILKKKGAAKAALW

>Streptococcus-pneumoniae_CJY70842.1

MKSKRMEFHNKFLIVSAMLAVISWLSLGVVSFPMLAGTLGISTKAAATVVNLISAYSTVTAVISIVGAITGVGSIGSGIAATVLYILKKKGAAKAALW

>Streptococcus-pneumoniae_CKF92960.1

MKSKRMEFHNKFLIVSAMLAVISWLSLGVVSFPMLAGTLGISTKAAATVVNLISAYSTVTAVISIVGAITGVGSIGSGIAATVLYILKKKGAAKAALW

>Streptococcus-pneumoniae_CKH84502.1

MKSKRMEFHNKFLIVSAMLAVISWLSLGVVSFPMLAGTLGISTKAAATVVNLISAYSTVTAVISIVGAITGVGSIGSGIAATVLYILKKKGAAKAALW

>Streptococcus-pneumoniae_CJU89856.1

MKSKRMEFHNKFLIVSAMLAVISWLSLGVVSFPMLAGTLGISTKAAATVVNLISAYSTVTAVISIVGAITGVGSIGSGIAATVLYILKKKGAAKAALW

>Streptococcus-pneumoniae_CKG24364.1

MKSKRMEFHNKFLIVSAMLAVISWLSLGVVSFPMLAGTLGISTKAAATVVNLISAYSTVTAVISIVGAITGVGSIGSGIAATVLYILKKKGAAKAALW

>Streptococcus-pneumoniae_CJX44275.1

MKSKRMEFHNKFLIVSAMLAVISWLSLGVVSFPMLAGTLGISTKAAATVVNLISAYSTVTAVISIVGAITGVGSIGSGIAATVLYILKKKGAAKAALW

>Streptococcus-pneumoniae_CKH03636.1

MKSKRMEFHNKFLIVSAMLAVISWLSLGVVSFPMLAGTLGISTKAAATVVNLISAYSTVTAVISIVGAITGVGSIGSGIAATVLYILKKKGAAKAALW

>Streptococcus-pneumoniae_CJV35545.1

MKSKRMEFHNKFLIVSAMLAVISWLSLGVVSFPMLAGTLGISTKAAATVVNLISAYSTVTAVISIVGAITGVGSIGSGIAATVLYILKKKGAAKAALW

>Streptococcus-pneumoniae_CKH75409.1

MKSKRMEFHNKFLIVSAMLAVISWLSLGVVSFPMLAGTLGISTKAAATVVNLISAYSTVTAVISIVGAITGVGSIGSGIAATVLYILKKKGAAKAALW

>Streptococcus-pneumoniae_CJT80104.1

MKSKRMEFHNKFLIVSAMLAVISWLSLGVVSFPMLAGTLGISTKAAATVVNLISAYSTVTAVISIVGAITGVGSIGSGIAATVLYILKKKGAAKAALW

>Streptococcus-pneumoniae_CJV95935.1

MKSKRMEFHNKFLIVSAMLAVISWLSLGVVSFPMLAGTLGISTKAAATVVNLISAYSTVTAVISIVGAITGVGSIGSGIAATVLYILKKKGAAKAALW

>Streptococcus-pneumoniae_CJT72242.1

MKSKRMEFHNKFLIVSAMLAVISWLSLGVVSFPMLAGTLGISTKAAATVVNLISAYSTVTAVISIVGAITGVGSIGSGIAATVLYILKKKGAAKAALW

>Streptococcus-pneumoniae_CKB61725.1

MKSKRMEFHNKFLIVSAMLAVISWLSLGVVSFPMLAGTLGISTKAAATVVNLISAYSTVTAVISIVGAITGVGSIGSGIAATVLYILKKKGAAKAALW

>Streptococcus-pneumoniae_CKM20919.1

MKSKRMEFHNKFLIVSAMLAVISWLSLGVVSFPMLAGTLGISTKAAATVVNLISAYSTVTAVISIVGAITGVGSIGSGIAATVLYILKKKGAAKAALW

>Streptococcus-pneumoniae_CIW19200.1

MKSKRMEFHNKFLIVSAMLAVISWLSLGVVSFPMLAGTLGISTKAAATVVNLISAYSTVTAVISIVGAITGVGSIGSGIAATVLYILKKKGAAKAALW

>Streptococcus-pneumoniae_CIQ81748.1

MKSKRMEFHNKFLIVSAMLAVISWLSLGVVSFPMLAGTLGISTKAAATVVNLISAYSTVTAVISIVGAITGVGSIGSGIAATVLYILKKKGAAKAALW

>Streptococcus-pneumoniae_CKI28298.1

MKSKRMEFHNKFLIVSAMLAVISWLSLGVVSFPMLAGTLGISTKAAATVVNLISAYSTVTAVISIVGAITGVGSIGSGIAATVLYILKKKGAAKAALW

>Streptococcus-pneumoniae_CMU74667.1

MKSKRMEFHNKFLIVSAMLAVISWLSLGVVSFPMLAGTLGISTKAAATVVNLISAYSTVTAVISIVGAITGVGSIGSGIAATVLYILKKKGAAKAALW

>Streptococcus-pneumoniae_CMZ11496.1

MKSKRMEFHNKFLIVSAMLAVISWLSLGVVSFPMLAGTLGISTKAAATVVNLISAYSTVTAVISIVGAITGVGSIGSGIAATVLYILKKKGAAKAALW

>Streptococcus-pneumoniae_CNB00432.1

MKSKRMEFHNKFLIVSAMLAVISWLSLGVVSFPMLAGTLGISTKAAATVVNLISAYSTVTAVISIVGAITGVGSIGSGIAATVLYILKKKGAAKAALW

>Streptococcus-pneumoniae_CMW90795.1

MKSKRMEFHNKFLIVSAMLAVISWLSLGVVSFPMLAGTLGISTKAAATVVNLISAYSTVTAVISIVGAITGVGSIGSGIAATVLYILKKKGAAKAALW

>Streptococcus-pneumoniae_CMY13828.1

MKSKRMEFHNKFLIVSAMLAVISWLSLGVVSFPMLAGTLGISTKAAATVVNLISAYSTVTAVISIVGAITGVGSIGSGIAATVLYILKKKGAAKAALW

>Streptococcus-pneumoniae_CMX01577.1

MKSKRMEFHNKFLIVSAMLAVISWLSLGVVSFPMLAGTLGISTKAAATVVNLISAYSTVTAVISIVGAITGVGSIGSGIAATVLYILKKKGAAKAALW

>Streptococcus-pneumoniae_CMX17294.1

MKSKRMEFHNKFLIVSAMLAVISWLSLGVVSFPMLAGTLGISTKAAATVVNLISAYSTVTAVISIVGAITGVGSIGSGIAATVLYILKKKGAAKAALW

>Streptococcus-pneumoniae_CMV79674.1

MKSKRMEFHNKFLIVSAMLAVISWLSLGVVSFPMLAGTLGISTKAAATVVNLISAYSTVTAVISIVGAITGVGSIGSGIAATVLYILKKKGAAKAALW

>Streptococcus-pneumoniae_CMX48705.1

MKSKRMEFHNKFLIVSAMLAVISWLSLGVVSFPMLAGTLGISTKAAATVVNLISAYSTVTAVISIVGAITGVGSIGSGIAATVLYILKKKGAAKAALW

>Streptococcus-pneumoniae_CMZ76177.1

MKSKRMEFHNKFLIVSAMLAVISWLSLGVVSFPMLAGTLGISTKAAATVVNLISAYSTVTAVISIVGAITGVGSIGSGIAATVLYILKKKGAAKAALW

>Streptococcus-pneumoniae_CMW36475.1

MKSKRMEFHNKFLIVSAMLAVISWLSLGVVSFPMLAGTLGISTKAAATVVNLISAYSTVTAVISIVGAITGVGSIGSGIAATVLYILKKKGAAKAALW

>Streptococcus-pneumoniae_CMX14418.1

MKSKRMEFHNKFLIVSAMLAVISWLSLGVVSFPMLAGTLGISTKAAATVVNLISAYSTVTAVISIVGAITGVGSIGSGIAATVLYILKKKGAAKAALW

>Streptococcus-pneumoniae_COH35067.1

MKSKRMEFHNKFLIVSAMLAVISWLSLGVVSFPMLAGTLGISTKAAATVVNLISAYSTVTAVISIVGAITGVGSIGSGIAATVLYILKKKGAAKAALW

>Streptococcus-pneumoniae_COE49052.1

MKSKRMEFHNKFLIVSAMLAVISWLSLGVVSFPMLAGTLGISTKAAATVVNLISAYSTVTAVISIVGAITGVGSIGSGIAATVLYILKKKGAAKAALW

>Streptococcus-pneumoniae_COJ60376.1

MKSKRMEFHNKFLIVSAMLAVISWLSLGVVSFPMLAGTLGISTKAAATVVNLISAYSTVTAVISIVGAITGVGSIGSGIAATVLYILKKKGAAKAALW

>Streptococcus-pneumoniae_COR80650.1

MKSKRMEFHNKFLIVSAMLAVISWLSLGVVSFPMLAGTLGISTKAAATVVNLISAYSTVTAVISIVGAITGVGSIGSGIAATVLYILKKKGAAKAALW

>Streptococcus-pneumoniae_COA08233.1

MKSKRMEFHNKFLIVSAMLAVISWLSLGVVSFPMLAGTLGISTKAAATVVNLISAYSTVTAVISIVGAITGVGSIGSGIAATVLYILKKKGAAKAALW

>Streptococcus-pneumoniae_COR20996.1

MKSKRMEFHNKFLIVSAMLAVISWLSLGVVSFPMLAGTLGISTKAAATVVNLISAYSTVTAVISIVGAITGVGSIGSGIAATVLYILKKKGAAKAALW

>Streptococcus-pneumoniae_COK10421.1

MKSKRMEFHNKFLIVSAMLAVISWLSLGVVSFPMLAGTLGISTKAAATVVNLISAYSTVTAVISIVGAITGVGSIGSGIAATVLYILKKKGAAKAALW

>Streptococcus-pneumoniae_CON52863.1

MKSKRMEFHNKFLIVSAMLAVISWLSLGVVSFPMLAGTLGISTKAAATVVNLISAYSTVTAVISIVGAITGVGSIGSGIAATVLYILKKKGAAKAALW

>Streptococcus-pneumoniae_COJ56081.1

MKSKRMEFHNKFLIVSAMLAVISWLSLGVVSFPMLAGTLGISTKAAATVVNLISAYSTVTAVISIVGAITGVGSIGSGIAATVLYILKKKGAAKAALW

>Streptococcus-pneumoniae_COM84903.1

MKSKRMEFHNKFLIVSAMLAVISWLSLGVVSFPMLAGTLGISTKAAATVVNLISAYSTVTAVISIVGAITGVGSIGSGIAATVLYILKKKGAAKAALW

>Streptococcus-pneumoniae_COK15622.1

MKSKRMEFHNKFLIVSAMLAVISWLSLGVVSFPMLAGTLGISTKAAATVVNLISAYSTVTAVISIVGAITGVGSIGSGIAATVLYILKKKGAAKAALW

>Streptococcus-pneumoniae_COR31581.1

MKSKRMEFHNKFLIVSAMLAVISWLSLGVVSFPMLAGTLGISTKAAATVVNLISAYSTVTAVISIVGAITGVGSIGSGIAATVLYILKKKGAAKAALW

>Streptococcus-pneumoniae_COG81302.1

MKSKRMEFHNKFLIVSAMLAVISWLSLGVVSFPMLAGTLGISTKAAATVVNLISAYSTVTAVISIVGAITGVGSIGSGIAATVLYILKKKGAAKAALW

>Streptococcus-pneumoniae_COQ86444.1

MKSKRMEFHNKFLIVSAMLAVISWLSLGVVSFPMLAGTLGISTKAAATVVNLISAYSTVTAVISIVGAITGVGSIGSGIAATVLYILKKKGAAKAALW

>Streptococcus-pneumoniae_COF28266.1

MKSKRMEFHNKFLIVSAMLAVISWLSLGVVSFPMLAGTLGISTKAAATVVNLISAYSTVTAVISIVGAITGVGSIGSGIAATVLYILKKKGAAKAALW

>Streptococcus-pneumoniae_COI77091.1

MKSKRMEFHNKFLIVSAMLAVISWLSLGVVSFPMLAGTLGISTKAAATVVNLISAYSTVTAVISIVGAITGVGSIGSGIAATVLYILKKKGAAKAALW

>Streptococcus-pneumoniae_COC16909.1

MKSKRMEFHNKFLIVSAMLAVISWLSLGVVSFPMLAGTLGISTKAAATVVNLISAYSTVTAVISIVGAITGVGSIGSGIAATVLYILKKKGAAKAALW

>Streptococcus-pneumoniae_COJ77147.1

MKSKRMEFHNKFLIVSAMLAVISWLSLGVVSFPMLAGTLGISTKAAATVVNLISAYSTVTAVISIVGAITGVGSIGSGIAATVLYILKKKGAAKAALW

>Streptococcus-pneumoniae_COP30623.1

MKSKRMEFHNKFLIVSAMLAVISWLSLGVVSFPMLAGTLGISTKAAATVVNLISAYSTVTAVISIVGAITGVGSIGSGIAATVLYILKKKGAAKAALW

>Streptococcus-pneumoniae_COJ49798.1

MKSKRMEFHNKFLIVSAMLAVISWLSLGVVSFPMLAGTLGISTKAAATVVNLISAYSTVTAVISIVGAITGVGSIGSGIAATVLYILKKKGAAKAALW

>Streptococcus-pneumoniae_COA34107.1

MKSKRMEFHNKFLIVSAMLAVISWLSLGVVSFPMLAGTLGISTKAAATVVNLISAYSTVTAVISIVGAITGVGSIGSGIAATVLYILKKKGAAKAALW

>Streptococcus-pneumoniae_COP58670.1

MKSKRMEFHNKFLIVSAMLAVISWLSLGVVSFPMLAGTLGISTKAAATVVNLISAYSTVTAVISIVGAITGVGSIGSGIAATVLYILKKKGAAKAALW

>Streptococcus-pneumoniae_COI34252.1

MKSKRMEFHNKFLIVSAMLAVISWLSLGVVSFPMLAGTLGISTKAAATVVNLISAYSTVTAVISIVGAITGVGSIGSGIAATVLYILKKKGAAKAALW

>Streptococcus-pneumoniae_CNZ35165.1

MKSKRMEFHNKFLIVSAMLAVISWLSLGVVSFPMLAGTLGISTKAAATVVNLISAYSTVTAVISIVGAITGVGSIGSGIAATVLYILKKKGAAKAALW

>Streptococcus-pneumoniae_COO52748.1

MKSKRMEFHNKFLIVSAMLAVISWLSLGVVSFPMLAGTLGISTKAAATVVNLISAYSTVTAVISIVGAITGVGSIGSGIAATVLYILKKKGAAKAALW

>Streptococcus-pneumoniae_COR47393.1

MKSKRMEFHNKFLIVSAMLAVISWLSLGVVSFPMLAGTLGISTKAAATVVNLISAYSTVTAVISIVGAITGVGSIGSGIAATVLYILKKKGAAKAALW

>Streptococcus-pneumoniae_COI21854.1

MKSKRMEFHNKFLIVSAMLAVISWLSLGVVSFPMLAGTLGISTKAAATVVNLISAYSTVTAVISIVGAITGVGSIGSGIAATVLYILKKKGAAKAALW

>Streptococcus-pneumoniae_CNZ46898.1

MKSKRMEFHNKFLIVSAMLAVISWLSLGVVSFPMLAGTLGISTKAAATVVNLISAYSTVTAVISIVGAITGVGSIGSGIAATVLYILKKKGAAKAALW

>Streptococcus-pneumoniae_COG69983.1

MKSKRMEFHNKFLIVSAMLAVISWLSLGVVSFPMLAGTLGISTKAAATVVNLISAYSTVTAVISIVGAITGVGSIGSGIAATVLYILKKKGAAKAALW

>Streptococcus-pneumoniae_COO59633.1

MKSKRMEFHNKFLIVSAMLAVISWLSLGVVSFPMLAGTLGISTKAAATVVNLISAYSTVTAVISIVGAITGVGSIGSGIAATVLYILKKKGAAKAALW

>Streptococcus-pneumoniae_COH09874.1

MKSKRMEFHNKFLIVSAMLAVISWLSLGVVSFPMLAGTLGISTKAAATVVNLISAYSTVTAVISIVGAITGVGSIGSGIAATVLYILKKKGAAKAALW

>Streptococcus-pneumoniae_COD50607.1

MKSKRMEFHNKFLIVSAMLAVISWLSLGVVSFPMLAGTLGISTKAAATVVNLISAYSTVTAVISIVGAITGVGSIGSGIAATVLYILKKKGAAKAALW

>Streptococcus-pneumoniae_COK40337.1

MKSKRMEFHNKFLIVSAMLAVISWLSLGVVSFPMLAGTLGISTKAAATVVNLISAYSTVTAVISIVGAITGVGSIGSGIAATVLYILKKKGAAKAALW

>Streptococcus-pneumoniae_COP67515.1

MKSKRMEFHNKFLIVSAMLAVISWLSLGVVSFPMLAGTLGISTKAAATVVNLISAYSTVTAVISIVGAITGVGSIGSGIAATVLYILKKKGAAKAALW

>Streptococcus-pneumoniae_COQ54651.1

MKSKRMEFHNKFLIVSAMLAVISWLSLGVVSFPMLAGTLGISTKAAATVVNLISAYSTVTAVISIVGAITGVGSIGSGIAATVLYILKKKGAAKAALW

>Streptococcus-pneumoniae_COJ56272.1

MKSKRMEFHNKFLIVSAMLAVISWLSLGVVSFPMLAGTLGISTKAAATVVNLISAYSTVTAVISIVGAITGVGSIGSGIAATVLYILKKKGAAKAALW

>Streptococcus-pneumoniae_COM31491.1

MKSKRMEFHNKFLIVSAMLAVISWLSLGVVSFPMLAGTLGISTKAAATVVNLISAYSTVTAVISIVGAITGVGSIGSGIAATVLYILKKKGAAKAALW

>Streptococcus-pneumoniae_COR94268.1

MKSKRMEFHNKFLIVSAMLAVISWLSLGVVSFPMLAGTLGISTKAAATVVNLISAYSTVTAVISIVGAITGVGSIGSGIAATVLYILKKKGAAKAALW

>Streptococcus-pneumoniae_COJ80780.1

MKSKRMEFHNKFLIVSAMLAVISWLSLGVVSFPMLAGTLGISTKAAATVVNLISAYSTVTAVISIVGAITGVGSIGSGIAATVLYILKKKGAAKAALW

>Streptococcus-pneumoniae_COK32045.1

MKSKRMEFHNKFLIVSAMLAVISWLSLGVVSFPMLAGTLGISTKAAATVVNLISAYSTVTAVISIVGAITGVGSIGSGIAATVLYILKKKGAAKAALW

>Streptococcus-pneumoniae_CON51520.1

MKSKRMEFHNKFLIVSAMLAVISWLSLGVVSFPMLAGTLGISTKAAATVVNLISAYSTVTAVISIVGAITGVGSIGSGIAATVLYILKKKGAAKAALW

>Streptococcus-pneumoniae_COL30174.1

MKSKRMEFHNKFLIVSAMLAVISWLSLGVVSFPMLAGTLGISTKAAATVVNLISAYSTVTAVISIVGAITGVGSIGSGIAATVLYILKKKGAAKAALW

>Streptococcus-pneumoniae_COK83864.1

MKSKRMEFHNKFLIVSAMLAVISWLSLGVVSFPMLAGTLGISTKAAATVVNLISAYSTVTAVISIVGAITGVGSIGSGIAATVLYILKKKGAAKAALW

>Streptococcus-pneumoniae_CNZ96470.1

MKSKRMEFHNKFLIVSAMLAVISWLSLGVVSFPMLAGTLGISTKAAATVVNLISAYSTVTAVISIVGAITGVGSIGSGIAATVLYILKKKGAAKAALW

>Streptococcus-pneumoniae_COK70023.1

MKSKRMEFHNKFLIVSAMLAVISWLSLGVVSFPMLAGTLGISTKAAATVVNLISAYSTVTAVISIVGAITGVGSIGSGIAATVLYILKKKGAAKAALW

>Streptococcus-pneumoniae_CON77181.1

MKSKRMEFHNKFLIVSAMLAVISWLSLGVVSFPMLAGTLGISTKAAATVVNLISAYSTVTAVISIVGAITGVGSIGSGIAATVLYILKKKGAAKAALW

>Streptococcus-pneumoniae_COK28675.1

MKSKRMEFHNKFLIVSAMLAVISWLSLGVVSFPMLAGTLGISTKAAATVVNLISAYSTVTAVISIVGAITGVGSIGSGIAATVLYILKKKGAAKAALW

>Streptococcus-pneumoniae_COP91575.1

MKSKRMEFHNKFLIVSAMLAVISWLSLGVVSFPMLAGTLGISTKAAATVVNLISAYSTVTAVISIVGAITGVGSIGSGIAATVLYILKKKGAAKAALW

>Streptococcus-pneumoniae_COD28532.1

MKSKRMEFHNKFLIVSAMLAVISWLSLGVVSFPMLAGTLGISTKAAATVVNLISAYSTVTAVISIVGAITGVGSIGSGIAATVLYILKKKGAAKAALW

>Streptococcus-pneumoniae_COI92385.1

MKSKRMEFHNKFLIVSAMLAVISWLSLGVVSFPMLAGTLGISTKAAATVVNLISAYSTVTAVISIVGAITGVGSIGSGIAATVLYILKKKGAAKAALW

>Streptococcus-pneumoniae_COE56784.1

MKSKRMEFHNKFLIVSAMLAVISWLSLGVVSFPMLAGTLGISTKAAATVVNLISAYSTVTAVISIVGAITGVGSIGSGIAATVLYILKKKGAAKAALW

>Streptococcus-pneumoniae_COP77955.1

MKSKRMEFHNKFLIVSAMLAVISWLSLGVVSFPMLAGTLGISTKAAATVVNLISAYSTVTAVISIVGAITGVGSIGSGIAATVLYILKKKGAAKAALW

>Streptococcus-pneumoniae_CNZ13832.1

MKSKRMEFHNKFLIVSAMLAVISWLSLGVVSFPMLAGTLGISTKAAATVVNLISAYSTVTAVISIVGAITGVGSIGSGIAATVLYILKKKGAAKAALW

>Streptococcus-pneumoniae_COH11941.1

MKSKRMEFHNKFLIVSAMLAVISWLSLGVVSFPMLAGTLGISTKAAATVVNLISAYSTVTAVISIVGAITGVGSIGSGIAATVLYILKKKGAAKAALW

>Streptococcus-pneumoniae_COJ33250.1

MKSKRMEFHNKFLIVSAMLAVISWLSLGVVSFPMLAGTLGISTKAAATVVNLISAYSTVTAVISIVGAITGVGSIGSGIAATVLYILKKKGAAKAALW

>Streptococcus-pneumoniae_COO92692.1

MKSKRMEFHNKFLIVSAMLAVISWLSLGVVSFPMLAGTLGISTKAAATVVNLISAYSTVTAVISIVGAITGVGSIGSGIAATVLYILKKKGAAKAALW

>Streptococcus-pneumoniae_COG60669.1

MKSKRMEFHNKFLIVSAMLAVISWLSLGVVSFPMLAGTLGISTKAAATVVNLISAYSTVTAVISIVGAITGVGSIGSGIAATVLYILKKKGAAKAALW

>Streptococcus-pneumoniae_COJ14383.1

MKSKRMEFHNKFLIVSAMLAVISWLSLGVVSFPMLAGTLGISTKAAATVVNLISAYSTVTAVISIVGAITGVGSIGSGIAATVLYILKKKGAAKAALW

>Streptococcus-pneumoniae_COG66590.1

MKSKRMEFHNKFLIVSAMLAVISWLSLGVVSFPMLAGTLGISTKAAATVVNLISAYSTVTAVISIVGAITGVGSIGSGIAATVLYILKKKGAAKAALW

>Streptococcus-pneumoniae_COQ90958.1

MKSKRMEFHNKFLIVSAMLAVISWLSLGVVSFPMLAGTLGISTKAAATVVNLISAYSTVTAVISIVGAITGVGSIGSGIAATVLYILKKKGAAKAALW

>Streptococcus-pneumoniae_COJ47821.1

MKSKRMEFHNKFLIVSAMLAVISWLSLGVVSFPMLAGTLGISTKAAATVVNLISAYSTVTAVISIVGAITGVGSIGSGIAATVLYILKKKGAAKAALW

>Streptococcus-pneumoniae_COL70838.1

MKSKRMEFHNKFLIVSAMLAVISWLSLGVVSFPMLAGTLGISTKAAATVVNLISAYSTVTAVISIVGAITGVGSIGSGIAATVLYILKKKGAAKAALW

>Streptococcus-pneumoniae_COP96650.1

MKSKRMEFHNKFLIVSAMLAVISWLSLGVVSFPMLAGTLGISTKAAATVVNLISAYSTVTAVISIVGAITGVGSIGSGIAATVLYILKKKGAAKAALW

>Streptococcus-pneumoniae_COG38431.1

MKSKRMEFHNKFLIVSAMLAVISWLSLGVVSFPMLAGTLGISTKAAATVVNLISAYSTVTAVISIVGAITGVGSIGSGIAATVLYILKKKGAAKAALW

>Streptococcus-pneumoniae_COK46977.1

MKSKRMEFHNKFLIVSAMLAVISWLSLGVVSFPMLAGTLGISTKAAATVVNLISAYSTVTAVISIVGAITGVGSIGSGIAATVLYILKKKGAAKAALW

>Streptococcus-pneumoniae_CNZ83631.1

MKSKRMEFHNKFLIVSAMLAVISWLSLGVVSFPMLAGTLGISTKAAATVVNLISAYSTVTAVISIVGAITGVGSIGSGIAATVLYILKKKGAAKAALW

>Streptococcus-pneumoniae_COC42621.1

MKSKRMEFHNKFLIVSAMLAVISWLSLGVVSFPMLAGTLGISTKAAATVVNLISAYSTVTAVISIVGAITGVGSIGSGIAATVLYILKKKGAAKAALW

>Streptococcus-pneumoniae_COC47009.1

MKSKRMEFHNKFLIVSAMLAVISWLSLGVVSFPMLAGTLGISTKAAATVVNLISAYSTVTAVISIVGAITGVGSIGSGIAATVLYILKKKGAAKAALW

>Streptococcus-pneumoniae_COG43491.1

MKSKRMEFHNKFLIVSAMLAVISWLSLGVVSFPMLAGTLGISTKAAATVVNLISAYSTVTAVISIVGAITGVGSIGSGIAATVLYILKKKGAAKAALW

>Streptococcus-pneumoniae_COL27469.1

MKSKRMEFHNKFLIVSAMLAVISWLSLGVVSFPMLAGTLGISTKAAATVVNLISAYSTVTAVISIVGAITGVGSIGSGIAATVLYILKKKGAAKAALW

>Streptococcus-pneumoniae_COG50495.1

MKSKRMEFHNKFLIVSAMLAVISWLSLGVVSFPMLAGTLGISTKAAATVVNLISAYSTVTAVISIVGAITGVGSIGSGIAATVLYILKKKGAAKAALW

>Streptococcus-pneumoniae_CON12278.1

MKSKRMEFHNKFLIVSAMLAVISWLSLGVVSFPMLAGTLGISTKAAATVVNLISAYSTVTAVISIVGAITGVGSIGSGIAATVLYILKKKGAAKAALW

>Streptococcus-pneumoniae_COA66266.1

MKSKRMEFHNKFLIVSAMLAVISWLSLGVVSFPMLAGTLGISTKAAATVVNLISAYSTVTAVISIVGAITGVGSIGSGIAATVLYILKKKGAAKAALW

>Streptococcus-pneumoniae_COC05056.1

MKSKRMEFHNKFLIVSAMLAVISWLSLGVVSFPMLAGTLGISTKAAATVVNLISAYSTVTAVISIVGAITGVGSIGSGIAATVLYILKKKGAAKAALW

>Streptococcus-pneumoniae_COJ60304.1

MKSKRMEFHNKFLIVSAMLAVISWLSLGVVSFPMLAGTLGISTKAAATVVNLISAYSTVTAVISIVGAITGVGSIGSGIAATVLYILKKKGAAKAALW

>Streptococcus-pneumoniae_COB02800.1

MKSKRMEFHNKFLIVSAMLAVISWLSLGVVSFPMLAGTLGISTKAAATVVNLISAYSTVTAVISIVGAITGVGSIGSGIAATVLYILKKKGAAKAALW

>Streptococcus-pneumoniae_COG43103.1

MKSKRMEFHNKFLIVSAMLAVISWLSLGVVSFPMLAGTLGISTKAAATVVNLISAYSTVTAVISIVGAITGVGSIGSGIAATVLYILKKKGAAKAALW

>Streptococcus-pneumoniae_CON56362.1

MKSKRMEFHNKFLIVSAMLAVISWLSLGVVSFPMLAGTLGISTKAAATVVNLISAYSTVTAVISIVGAITGVGSIGSGIAATVLYILKKKGAAKAALW

>Streptococcus-pneumoniae_CON26282.1

MKSKRMEFHNKFLIVSAMLAVISWLSLGVVSFPMLAGTLGISTKAAATVVNLISAYSTVTAVISIVGAITGVGSIGSGIAATVLYILKKKGAAKAALW

>Streptococcus-pneumoniae_COM42190.1

MKSKRMEFHNKFLIVSAMLAVISWLSLGVVSFPMLAGTLGISTKAAATVVNLISAYSTVTAVISIVGAITGVGSIGSGIAATVLYILKKKGAAKAALW

>Streptococcus-pneumoniae_COK82516.1

MKSKRMEFHNKFLIVSAMLAVISWLSLGVVSFPMLAGTLGISTKAAATVVNLISAYSTVTAVISIVGAITGVGSIGSGIAATVLYILKKKGAAKAALW

>Streptococcus-pneumoniae_COL67184.1

MKSKRMEFHNKFLIVSAMLAVISWLSLGVVSFPMLAGTLGISTKAAATVVNLISAYSTVTAVISIVGAITGVGSIGSGIAATVLYILKKKGAAKAALW

>Streptococcus-pneumoniae_COB46936.1

MKSKRMEFHNKFLIVSAMLAVISWLSLGVVSFPMLAGTLGISTKAAATVVNLISAYSTVTAVISIVGAITGVGSIGSGIAATVLYILKKKGAAKAALW

>Streptococcus-pneumoniae_COD54158.1

MKSKRMEFHNKFLIVSAMLAVISWLSLGVVSFPMLAGTLGISTKAAATVVNLISAYSTVTAVISIVGAITGVGSIGSGIAATVLYILKKKGAAKAALW

>Streptococcus-pneumoniae_COE54669.1

MKSKRMEFHNKFLIVSAMLAVISWLSLGVVSFPMLAGTLGISTKAAATVVNLISAYSTVTAVISIVGAITGVGSIGSGIAATVLYILKKKGAAKAALW

>Streptococcus-pneumoniae_CON86586.1

MKSKRMEFHNKFLIVSAMLAVISWLSLGVVSFPMLAGTLGISTKAAATVVNLISAYSTVTAVISIVGAITGVGSIGSGIAATVLYILKKKGAAKAALW

>Streptococcus-pneumoniae_COI17106.1

MKSKRMEFHNKFLIVSAMLAVISWLSLGVVSFPMLAGTLGISTKAAATVVNLISAYSTVTAVISIVGAITGVGSIGSGIAATVLYILKKKGAAKAALW

>Streptococcus-pneumoniae_COP13016.1

MKSKRMEFHNKFLIVSAMLAVISWLSLGVVSFPMLAGTLGISTKAAATVVNLISAYSTVTAVISIVGAITGVGSIGSGIAATVLYILKKKGAAKAALW

>Streptococcus-pneumoniae_COL94627.1

MKSKRMEFHNKFLIVSAMLAVISWLSLGVVSFPMLAGTLGISTKAAATVVNLISAYSTVTAVISIVGAITGVGSIGSGIAATVLYILKKKGAAKAALW

>Streptococcus-pneumoniae_COH90971.1

MKSKRMEFHNKFLIVSAMLAVISWLSLGVVSFPMLAGTLGISTKAAATVVNLISAYSTVTAVISIVGAITGVGSIGSGIAATVLYILKKKGAAKAALW

>Streptococcus-pneumoniae_COJ92603.1

MKSKRMEFHNKFLIVSAMLAVISWLSLGVVSFPMLAGTLGISTKAAATVVNLISAYSTVTAVISIVGAITGVGSIGSGIAATVLYILKKKGAAKAALW

>Streptococcus-pneumoniae_CON47244.1

MKSKRMEFHNKFLIVSAMLAVISWLSLGVVSFPMLAGTLGISTKAAATVVNLISAYSTVTAVISIVGAITGVGSIGSGIAATVLYILKKKGAAKAALW

>Streptococcus-pneumoniae_COE58305.1

MKSKRMEFHNKFLIVSAMLAVISWLSLGVVSFPMLAGTLGISTKAAATVVNLISAYSTVTAVISIVGAITGVGSIGSGIAATVLYILKKKGAAKAALW

>Streptococcus-pneumoniae_COP75878.1

MKSKRMEFHNKFLIVSAMLAVISWLSLGVVSFPMLAGTLGISTKAAATVVNLISAYSTVTAVISIVGAITGVGSIGSGIAATVLYILKKKGAAKAALW

>Streptococcus-pneumoniae_COQ59474.1

MKSKRMEFHNKFLIVSAMLAVISWLSLGVVSFPMLAGTLGISTKAAATVVNLISAYSTVTAVISIVGAITGVGSIGSGIAATVLYILKKKGAAKAALW

>Streptococcus-pneumoniae_COK70594.1

MKSKRMEFHNKFLIVSAMLAVISWLSLGVVSFPMLAGTLGISTKAAATVVNLISAYSTVTAVISIVGAITGVGSIGSGIAATVLYILKKKGAAKAALW

>Streptococcus-pneumoniae_COO47930.1

MKSKRMEFHNKFLIVSAMLAVISWLSLGVVSFPMLAGTLGISTKAAATVVNLISAYSTVTAVISIVGAITGVGSIGSGIAATVLYILKKKGAAKAALW

>Streptococcus-pneumoniae_COR58834.1

MKSKRMEFHNKFLIVSAMLAVISWLSLGVVSFPMLAGTLGISTKAAATVVNLISAYSTVTAVISIVGAITGVGSIGSGIAATVLYILKKKGAAKAALW

>Streptococcus-pneumoniae_COO03711.1

MKSKRMEFHNKFLIVSAMLAVISWLSLGVVSFPMLAGTLGISTKAAATVVNLISAYSTVTAVISIVGAITGVGSIGSGIAATVLYILKKKGAAKAALW

>Streptococcus-pneumoniae_CNZ75886.1

MKSKRMEFHNKFLIVSAMLAVISWLSLGVVSFPMLAGTLGISTKAAATVVNLISAYSTVTAVISIVGAITGVGSIGSGIAATVLYILKKKGAAKAALW

>Streptococcus-pneumoniae_COG67373.1

MKSKRMEFHNKFLIVSAMLAVISWLSLGVVSFPMLAGTLGISTKAAATVVNLISAYSTVTAVISIVGAITGVGSIGSGIAATVLYILKKKGAAKAALW

>Streptococcus-pneumoniae_COH38836.1

MKSKRMEFHNKFLIVSAMLAVISWLSLGVVSFPMLAGTLGISTKAAATVVNLISAYSTVTAVISIVGAITGVGSIGSGIAATVLYILKKKGAAKAALW

>Streptococcus-pneumoniae_COA54145.1

MKSKRMEFHNKFLIVSAMLAVISWLSLGVVSFPMLAGTLGISTKAAATVVNLISAYSTVTAVISIVGAITGVGSIGSGIAATVLYILKKKGAAKAALW

>Streptococcus-pneumoniae_CON14608.1

MKSKRMEFHNKFLIVSAMLAVISWLSLGVVSFPMLAGTLGISTKAAATVVNLISAYSTVTAVISIVGAITGVGSIGSGIAATVLYILKKKGAAKAALW

>Streptococcus-pneumoniae_COC12522.1

MKSKRMEFHNKFLIVSAMLAVISWLSLGVVSFPMLAGTLGISTKAAATVVNLISAYSTVTAVISIVGAITGVGSIGSGIAATVLYILKKKGAAKAALW

>Streptococcus-pneumoniae_COD97033.1

MKSKRMEFHNKFLIVSAMLAVISWLSLGVVSFPMLAGTLGISTKAAATVVNLISAYSTVTAVISIVGAITGVGSIGSGIAATVLYILKKKGAAKAALW

>Streptococcus-pneumoniae_COF14567.1

MKSKRMEFHNKFLIVSAMLAVISWLSLGVVSFPMLAGTLGISTKAAATVVNLISAYSTVTAVISIVGAITGVGSIGSGIAATVLYILKKKGAAKAALW

>Streptococcus-pneumoniae_COD54771.1

MKSKRMEFHNKFLIVSAMLAVISWLSLGVVSFPMLAGTLGISTKAAATVVNLISAYSTVTAVISIVGAITGVGSIGSGIAATVLYILKKKGAAKAALW

>Streptococcus-pneumoniae_COB60750.1

MKSKRMEFHNKFLIVSAMLAVISWLSLGVVSFPMLAGTLGISTKAAATVVNLISAYSTVTAVISIVGAITGVGSIGSGIAATVLYILKKKGAAKAALW

>Streptococcus-pneumoniae_COF53795.1

MKSKRMEFHNKFLIVSAMLAVISWLSLGVVSFPMLAGTLGISTKAAATVVNLISAYSTVTAVISIVGAITGVGSIGSGIAATVLYILKKKGAAKAALW

>Streptococcus-pneumoniae_COM56451.1

MKSKRMEFHNKFLIVSAMLAVISWLSLGVVSFPMLAGTLGISTKAAATVVNLISAYSTVTAVISIVGAITGVGSIGSGIAATVLYILKKKGAAKAALW

>Streptococcus-pneumoniae_COQ09977.1

MKSKRMEFHNKFLIVSAMLAVISWLSLGVVSFPMLAGTLGISTKAAATVVNLISAYSTVTAVISIVGAITGVGSIGSGIAATVLYILKKKGAAKAALW

>Streptococcus-pneumoniae_CNZ63165.1

MKSKRMEFHNKFLIVSAMLAVISWLSLGVVSFPMLAGTLGISTKAAATVVNLISAYSTVTAVISIVGAITGVGSIGSGIAATVLYILKKKGAAKAALW

>Streptococcus-pneumoniae_COF01730.1

MKSKRMEFHNKFLIVSAMLAVISWLSLGVVSFPMLAGTLGISTKAAATVVNLISAYSTVTAVISIVGAITGVGSIGSGIAATVLYILKKKGAAKAALW

>Streptococcus-pneumoniae_COB08984.1

MKSKRMEFHNKFLIVSAMLAVISWLSLGVVSFPMLAGTLGISTKAAATVVNLISAYSTVTAVISIVGAITGVGSIGSGIAATVLYILKKKGAAKAALW

>Streptococcus-pneumoniae_COG75450.1

MKSKRMEFHNKFLIVSAMLAVISWLSLGVVSFPMLAGTLGISTKAAATVVNLISAYSTVTAVISIVGAITGVGSIGSGIAATVLYILKKKGAAKAALW

>Streptococcus-pneumoniae_COR65367.1

MKSKRMEFHNKFLIVSAMLAVISWLSLGVVSFPMLAGTLGISTKAAATVVNLISAYSTVTAVISIVGAITGVGSIGSGIAATVLYILKKKGAAKAALW

>Streptococcus-pneumoniae_COP21457.1

MKSKRMEFHNKFLIVSAMLAVISWLSLGVVSFPMLAGTLGISTKAAATVVNLISAYSTVTAVISIVGAITGVGSIGSGIAATVLYILKKKGAAKAALW

>Streptococcus-pneumoniae_COC37524.1

MKSKRMEFHNKFLIVSAMLAVISWLSLGVVSFPMLAGTLGISTKAAATVVNLISAYSTVTAVISIVGAITGVGSIGSGIAATVLYILKKKGAAKAALW

>Streptococcus-pneumoniae_COF39869.1

MKSKRMEFHNKFLIVSAMLAVISWLSLGVVSFPMLAGTLGISTKAAATVVNLISAYSTVTAVISIVGAITGVGSIGSGIAATVLYILKKKGAAKAALW

>Streptococcus-pneumoniae_COM96717.1

MKSKRMEFHNKFLIVSAMLAVISWLSLGVVSFPMLAGTLGISTKAAATVVNLISAYSTVTAVISIVGAITGVGSIGSGIAATVLYILKKKGAAKAALW

>Streptococcus-pneumoniae_COR15985.1

MKSKRMEFHNKFLIVSAMLAVISWLSLGVVSFPMLAGTLGISTKAAATVVNLISAYSTVTAVISIVGAITGVGSIGSGIAATVLYILKKKGAAKAALW

>Streptococcus-pneumoniae_COF22908.1

MKSKRMEFHNKFLIVSAMLAVISWLSLGVVSFPMLAGTLGISTKAAATVVNLISAYSTVTAVISIVGAITGVGSIGSGIAATVLYILKKKGAAKAALW

>Streptococcus-pneumoniae_COQ01369.1

MKSKRMEFHNKFLIVSAMLAVISWLSLGVVSFPMLAGTLGISTKAAATVVNLISAYSTVTAVISIVGAITGVGSIGSGIAATVLYILKKKGAAKAALW

>Streptococcus-pneumoniae_COO24771.1

MKSKRMEFHNKFLIVSAMLAVISWLSLGVVSFPMLAGTLGISTKAAATVVNLISAYSTVTAVISIVGAITGVGSIGSGIAATVLYILKKKGAAKAALW

>Streptococcus-pneumoniae_COO17030.1

MKSKRMEFHNKFLIVSAMLAVISWLSLGVVSFPMLAGTLGISTKAAATVVNLISAYSTVTAVISIVGAITGVGSIGSGIAATVLYILKKKGAAKAALW

>Streptococcus-pneumoniae_COA68484.1

MKSKRMEFHNKFLIVSAMLAVISWLSLGVVSFPMLAGTLGISTKAAATVVNLISAYSTVTAVISIVGAITGVGSIGSGIAATVLYILKKKGAAKAALW

>Streptococcus-pneumoniae_COC62203.1

MKSKRMEFHNKFLIVSAMLAVISWLSLGVVSFPMLAGTLGISTKAAATVVNLISAYSTVTAVISIVGAITGVGSIGSGIAATVLYILKKKGAAKAALW

>Streptococcus-pneumoniae_COI95982.1

MKSKRMEFHNKFLIVSAMLAVISWLSLGVVSFPMLAGTLGISTKAAATVVNLISAYSTVTAVISIVGAITGVGSIGSGIAATVLYILKKKGAAKAALW

>Streptococcus-pneumoniae_COR97608.1

MKSKRMEFHNKFLIVSAMLAVISWLSLGVVSFPMLAGTLGISTKAAATVVNLISAYSTVTAVISIVGAITGVGSIGSGIAATVLYILKKKGAAKAALW

>Streptococcus-pneumoniae_COP48477.1

MKSKRMEFHNKFLIVSAMLAVISWLSLGVVSFPMLAGTLGISTKAAATVVNLISAYSTVTAVISIVGAITGVGSIGSGIAATVLYILKKKGAAKAALW

>Streptococcus-pneumoniae_COO82481.1

MKSKRMEFHNKFLIVSAMLAVISWLSLGVVSFPMLAGTLGISTKAAATVVNLISAYSTVTAVISIVGAITGVGSIGSGIAATVLYILKKKGAAKAALW

>Streptococcus-pneumoniae_COF10773.1

MKSKRMEFHNKFLIVSAMLAVISWLSLGVVSFPMLAGTLGISTKAAATVVNLISAYSTVTAVISIVGAITGVGSIGSGIAATVLYILKKKGAAKAALW

>Streptococcus-pneumoniae_COF93477.1

MKSKRMEFHNKFLIVSAMLAVISWLSLGVVSFPMLAGTLGISTKAAATVVNLISAYSTVTAVISIVGAITGVGSIGSGIAATVLYILKKKGAAKAALW

>Streptococcus-pneumoniae_COI79592.1

MKSKRMEFHNKFLIVSAMLAVISWLSLGVVSFPMLAGTLGISTKAAATVVNLISAYSTVTAVISIVGAITGVGSIGSGIAATVLYILKKKGAAKAALW

>Streptococcus-pneumoniae_COA99478.1

MKSKRMEFHNKFLIVSAMLAVISWLSLGVVSFPMLAGTLGISTKAAATVVNLISAYSTVTAVISIVGAITGVGSIGSGIAATVLYILKKKGAAKAALW

>Streptococcus-pneumoniae_COO50896.1

MKSKRMEFHNKFLIVSAMLAVISWLSLGVVSFPMLAGTLGISTKAAATVVNLISAYSTVTAVISIVGAITGVGSIGSGIAATVLYILKKKGAAKAALW

>Streptococcus-pneumoniae_COQ63073.1

MKSKRMEFHNKFLIVSAMLAVISWLSLGVVSFPMLAGTLGISTKAAATVVNLISAYSTVTAVISIVGAITGVGSIGSGIAATVLYILKKKGAAKAALW

>Streptococcus-pneumoniae_COK72732.1

MKSKRMEFHNKFLIVSAMLAVISWLSLGVVSFPMLAGTLGISTKAAATVVNLISAYSTVTAVISIVGAITGVGSIGSGIAATVLYILKKKGAAKAALW

>Streptococcus-pneumoniae_COJ98556.1

MKSKRMEFHNKFLIVSAMLAVISWLSLGVVSFPMLAGTLGISTKAAATVVNLISAYSTVTAVISIVGAITGVGSIGSGIAATVLYILKKKGAAKAALW

>Streptococcus-pneumoniae_COL15500.1

MKSKRMEFHNKFLIVSAMLAVISWLSLGVVSFPMLAGTLGISTKAAATVVNLISAYSTVTAVISIVGAITGVGSIGSGIAATVLYILKKKGAAKAALW

>Streptococcus-pneumoniae_COL06277.1

MKSKRMEFHNKFLIVSAMLAVISWLSLGVVSFPMLAGTLGISTKAAATVVNLISAYSTVTAVISIVGAITGVGSIGSGIAATVLYILKKKGAAKAALW

>Streptococcus-pneumoniae_COC44438.1

MKSKRMEFHNKFLIVSAMLAVISWLSLGVVSFPMLAGTLGISTKAAATVVNLISAYSTVTAVISIVGAITGVGSIGSGIAATVLYILKKKGAAKAALW

>Streptococcus-pneumoniae_CNZ52329.1

MKSKRMEFHNKFLIVSAMLAVISWLSLGVVSFPMLAGTLGISTKAAATVVNLISAYSTVTAVISIVGAITGVGSIGSGIAATVLYILKKKGAAKAALW

>Streptococcus-pneumoniae_COG88529.1

MKSKRMEFHNKFLIVSAMLAVISWLSLGVVSFPMLAGTLGISTKAAATVVNLISAYSTVTAVISIVGAITGVGSIGSGIAATVLYILKKKGAAKAALW

>Streptococcus-pneumoniae_COM73451.1

MKSKRMEFHNKFLIVSAMLAVISWLSLGVVSFPMLAGTLGISTKAAATVVNLISAYSTVTAVISIVGAITGVGSIGSGIAATVLYILKKKGAAKAALW

>Streptococcus-pneumoniae_COS25702.1

MKSKRMEFHNKFLIVSAMLAVISWLSLGVVSFPMLAGTLGISTKAAATVVNLISAYSTVTAVISIVGAITGVGSIGSGIAATVLYILKKKGAAKAALW

>Streptococcus-pneumoniae_COJ12456.1

MKSKRMEFHNKFLIVSAMLAVISWLSLGVVSFPMLAGTLGISTKAAATVVNLISAYSTVTAVISIVGAITGVGSIGSGIAATVLYILKKKGAAKAALW

>Streptococcus-pneumoniae_COR86349.1

MKSKRMEFHNKFLIVSAMLAVISWLSLGVVSFPMLAGTLGISTKAAATVVNLISAYSTVTAVISIVGAITGVGSIGSGIAATVLYILKKKGAAKAALW

>Streptococcus-pneumoniae_COQ41081.1

MKSKRMEFHNKFLIVSAMLAVISWLSLGVVSFPMLAGTLGISTKAAATVVNLISAYSTVTAVISIVGAITGVGSIGSGIAATVLYILKKKGAAKAALW

>Streptococcus-pneumoniae_COL17148.1

MKSKRMEFHNKFLIVSAMLAVISWLSLGVVSFPMLAGTLGISTKAAATVVNLISAYSTVTAVISIVGAITGVGSIGSGIAATVLYILKKKGAAKAALW

>Streptococcus-pneumoniae_COI16348.1

MKSKRMEFHNKFLIVSAMLAVISWLSLGVVSFPMLAGTLGISTKAAATVVNLISAYSTVTAVISIVGAITGVGSIGSGIAATVLYILKKKGAAKAALW

>Streptococcus-pneumoniae_COK33393.1

MKSKRMEFHNKFLIVSAMLAVISWLSLGVVSFPMLAGTLGISTKAAATVVNLISAYSTVTAVISIVGAITGVGSIGSGIAATVLYILKKKGAAKAALW

>Streptococcus-pneumoniae_COI29873.1

MKSKRMEFHNKFLIVSAMLAVISWLSLGVVSFPMLAGTLGISTKAAATVVNLISAYSTVTAVISIVGAITGVGSIGSGIAATVLYILKKKGAAKAALW

>Streptococcus-pneumoniae_COF30903.1

MKSKRMEFHNKFLIVSAMLAVISWLSLGVVSFPMLAGTLGISTKAAATVVNLISAYSTVTAVISIVGAITGVGSIGSGIAATVLYILKKKGAAKAALW

>Streptococcus-pneumoniae_COS06163.1

MKSKRMEFHNKFLIVSAMLAVISWLSLGVVSFPMLAGTLGISTKAAATVVNLISAYSTVTAVISIVGAITGVGSIGSGIAATVLYILKKKGAAKAALW

>Streptococcus-pneumoniae_COR69196.1

MKSKRMEFHNKFLIVSAMLAVISWLSLGVVSFPMLAGTLGISTKAAATVVNLISAYSTVTAVISIVGAITGVGSIGSGIAATVLYILKKKGAAKAALW

>Streptococcus-pneumoniae_COP32285.1

MKSKRMEFHNKFLIVSAMLAVISWLSLGVVSFPMLAGTLGISTKAAATVVNLISAYSTVTAVISIVGAITGVGSIGSGIAATVLYILKKKGAAKAALW

>Streptococcus-pneumoniae_COL35339.1

MKSKRMEFHNKFLIVSAMLAVISWLSLGVVSFPMLAGTLGISTKAAATVVNLISAYSTVTAVISIVGAITGVGSIGSGIAATVLYILKKKGAAKAALW

>Streptococcus-pneumoniae_COP61920.1

MKSKRMEFHNKFLIVSAMLAVISWLSLGVVSFPMLAGTLGISTKAAATVVNLISAYSTVTAVISIVGAITGVGSIGSGIAATVLYILKKKGAAKAALW

>Streptococcus-pneumoniae_COQ87507.1

MKSKRMEFHNKFLIVSAMLAVISWLSLGVVSFPMLAGTLGISTKAAATVVNLISAYSTVTAVISIVGAITGVGSIGSGIAATVLYILKKKGAAKAALW

>Streptococcus-pneumoniae_COR91509.1

MKSKRMEFHNKFLIVSAMLAVISWLSLGVVSFPMLAGTLGISTKAAATVVNLISAYSTVTAVISIVGAITGVGSIGSGIAATVLYILKKKGAAKAALW

>Streptococcus-pneumoniae_COA39217.1

MKSKRMEFHNKFLIVSAMLAVISWLSLGVVSFPMLAGTLGISTKAAATVVNLISAYSTVTAVISIVGAITGVGSIGSGIAATVLYILKKKGAAKAALW

>Streptococcus-pneumoniae_COB66838.1

MKSKRMEFHNKFLIVSAMLAVISWLSLGVVSFPMLAGTLGISTKAAATVVNLISAYSTVTAVISIVGAITGVGSIGSGIAATVLYILKKKGAAKAALW

>Streptococcus-pneumoniae_CNZ08846.1

MKSKRMEFHNKFLIVSAMLAVISWLSLGVVSFPMLAGTLGISTKAAATVVNLISAYSTVTAVISIVGAITGVGSIGSGIAATVLYILKKKGAAKAALW

>Streptococcus-pneumoniae_COK84158.1

MKSKRMEFHNKFLIVSAMLAVISWLSLGVVSFPMLAGTLGISTKAAATVVNLISAYSTVTAVISIVGAITGVGSIGSGIAATVLYILKKKGAAKAALW

>Streptococcus-pneumoniae_COE42558.1

MKSKRMEFHNKFLIVSAMLAVISWLSLGVVSFPMLAGTLGISTKAAATVVNLISAYSTVTAVISIVGAITGVGSIGSGIAATVLYILKKKGAAKAALW

>Streptococcus-pneumoniae_COL60006.1

MKSKRMEFHNKFLIVSAMLAVISWLSLGVVSFPMLAGTLGISTKAAATVVNLISAYSTVTAVISIVGAITGVGSIGSGIAATVLYILKKKGAAKAALW

>Streptococcus-pneumoniae_COO57122.1

MKSKRMEFHNKFLIVSAMLAVISWLSLGVVSFPMLAGTLGISTKAAATVVNLISAYSTVTAVISIVGAITGVGSIGSGIAATVLYILKKKGAAKAALW

>Streptococcus-pneumoniae_COJ23020.1

MKSKRMEFHNKFLIVSAMLAVISWLSLGVVSFPMLAGTLGISTKAAATVVNLISAYSTVTAVISIVGAITGVGSIGSGIAATVLYILKKKGAAKAALW

>Streptococcus-pneumoniae_COR54490.1

MKSKRMEFHNKFLIVSAMLAVISWLSLGVVSFPMLAGTLGISTKAAATVVNLISAYSTVTAVISIVGAITGVGSIGSGIAATVLYILKKKGAAKAALW

>Streptococcus-pneumoniae_COI58727.1

MKSKRMEFHNKFLIVSAMLAVISWLSLGVVSFPMLAGTLGISTKAAATVVNLISAYSTVTAVISIVGAITGVGSIGSGIAATVLYILKKKGAAKAALW

>Streptococcus-pneumoniae_COL30990.1

MKSKRMEFHNKFLIVSAMLAVISWLSLGVVSFPMLAGTLGISTKAAATVVNLISAYSTVTAVISIVGAITGVGSIGSGIAATVLYILKKKGAAKAALW

>Streptococcus-pneumoniae_COC01986.1

MKSKRMEFHNKFLIVSAMLAVISWLSLGVVSFPMLAGTLGISTKAAATVVNLISAYSTVTAVISIVGAITGVGSIGSGIAATVLYILKKKGAAKAALW

>Streptococcus-pneumoniae_COR90677.1

MKSKRMEFHNKFLIVSAMLAVISWLSLGVVSFPMLAGTLGISTKAAATVVNLISAYSTVTAVISIVGAITGVGSIGSGIAATVLYILKKKGAAKAALW

>Streptococcus-pneumoniae_COC24614.1

MKSKRMEFHNKFLIVSAMLAVISWLSLGVVSFPMLAGTLGISTKAAATVVNLISAYSTVTAVISIVGAITGVGSIGSGIAATVLYILKKKGAAKAALW

>Streptococcus-pneumoniae_COD86358.1

MKSKRMEFHNKFLIVSAMLAVISWLSLGVVSFPMLAGTLGISTKAAATVVNLISAYSTVTAVISIVGAITGVGSIGSGIAATVLYILKKKGAAKAALW

>Streptococcus-pneumoniae_COE02196.1

MKSKRMEFHNKFLIVSAMLAVISWLSLGVVSFPMLAGTLGISTKAAATVVNLISAYSTVTAVISIVGAITGVGSIGSGIAATVLYILKKKGAAKAALW

>Streptococcus-pneumoniae_COB68681.1

MKSKRMEFHNKFLIVSAMLAVISWLSLGVVSFPMLAGTLGISTKAAATVVNLISAYSTVTAVISIVGAITGVGSIGSGIAATVLYILKKKGAAKAALW

>Streptococcus-pneumoniae_COE88785.1

MKSKRMEFHNKFLIVSAMLAVISWLSLGVVSFPMLAGTLGISTKAAATVVNLISAYSTVTAVISIVGAITGVGSIGSGIAATVLYILKKKGAAKAALW

>Streptococcus-pneumoniae_COQ74567.1

MKSKRMEFHNKFLIVSAMLAVISWLSLGVVSFPMLAGTLGISTKAAATVVNLISAYSTVTAVISIVGAITGVGSIGSGIAATVLYILKKKGAAKAALW

>Streptococcus-pneumoniae_COF77069.1

MKSKRMEFHNKFLIVSAMLAVISWLSLGVVSFPMLAGTLGISTKAAATVVNLISAYSTVTAVISIVGAITGVGSIGSGIAATVLYILKKKGAAKAALW

>Streptococcus-pneumoniae_CON11131.1

MKSKRMEFHNKFLIVSAMLAVISWLSLGVVSFPMLAGTLGISTKAAATVVNLISAYSTVTAVISIVGAITGVGSIGSGIAATVLYILKKKGAAKAALW

>Streptococcus-pneumoniae_COD11585.1

MKSKRMEFHNKFLIVSAMLAVISWLSLGVVSFPMLAGTLGISTKAAATVVNLISAYSTVTAVISIVGAITGVGSIGSGIAATVLYILKKKGAAKAALW

>Streptococcus-pneumoniae_CNZ95439.1

MKSKRMEFHNKFLIVSAMLAVISWLSLGVVSFPMLAGTLGISTKAAATVVNLISAYSTVTAVISIVGAITGVGSIGSGIAATVLYILKKKGAAKAALW

>Streptococcus-pneumoniae_COJ22945.1

MKSKRMEFHNKFLIVSAMLAVISWLSLGVVSFPMLAGTLGISTKAAATVVNLISAYSTVTAVISIVGAITGVGSIGSGIAATVLYILKKKGAAKAALW

>Streptococcus-pneumoniae_COS00399.1

MKSKRMEFHNKFLIVSAMLAVISWLSLGVVSFPMLAGTLGISTKAAATVVNLISAYSTVTAVISIVGAITGVGSIGSGIAATVLYILKKKGAAKAALW

>Streptococcus-pneumoniae_COB66956.1

MKSKRMEFHNKFLIVSAMLAVISWLSLGVVSFPMLAGTLGISTKAAATVVNLISAYSTVTAVISIVGAITGVGSIGSGIAATVLYILKKKGAAKAALW

>Streptococcus-pneumoniae_COD42047.1

MKSKRMEFHNKFLIVSAMLAVISWLSLGVVSFPMLAGTLGISTKAAATVVNLISAYSTVTAVISIVGAITGVGSIGSGIAATVLYILKKKGAAKAALW

>Streptococcus-pneumoniae_COL77007.1

MKSKRMEFHNKFLIVSAMLAVISWLSLGVVSFPMLAGTLGISTKAAATVVNLISAYSTVTAVISIVGAITGVGSIGSGIAATVLYILKKKGAAKAALW

>Streptococcus-pneumoniae_COF76570.1

MKSKRMEFHNKFLIVSAMLAVISWLSLGVVSFPMLAGTLGISTKAAATVVNLISAYSTVTAVISIVGAITGVGSIGSGIAATVLYILKKKGAAKAALW

>Streptococcus-pneumoniae_COE63710.1

MKSKRMEFHNKFLIVSAMLAVISWLSLGVVSFPMLAGTLGISTKAAATVVNLISAYSTVTAVISIVGAITGVGSIGSGIAATVLYILKKKGAAKAALW

>Streptococcus-pneumoniae_COB19955.1

MKSKRMEFHNKFLIVSAMLAVISWLSLGVVSFPMLAGTLGISTKAAATVVNLISAYSTVTAVISIVGAITGVGSIGSGIAATVLYILKKKGAAKAALW

>Streptococcus-pneumoniae_COD45686.1

MKSKRMEFHNKFLIVSAMLAVISWLSLGVVSFPMLAGTLGISTKAAATVVNLISAYSTVTAVISIVGAITGVGSIGSGIAATVLYILKKKGAAKAALW

>Streptococcus-pneumoniae_COF76665.1

MKSKRMEFHNKFLIVSAMLAVISWLSLGVVSFPMLAGTLGISTKAAATVVNLISAYSTVTAVISIVGAITGVGSIGSGIAATVLYILKKKGAAKAALW

>Streptococcus-pneumoniae_COG32204.1

MKSKRMEFHNKFLIVSAMLAVISWLSLGVVSFPMLAGTLGISTKAAATVVNLISAYSTVTAVISIVGAITGVGSIGSGIAATVLYILKKKGAAKAALW

>Streptococcus-pneumoniae_COC69098.1

MKSKRMEFHNKFLIVSAMLAVISWLSLGVVSFPMLAGTLGISTKAAATVVNLISAYSTVTAVISIVGAITGVGSIGSGIAATVLYILKKKGAAKAALW

>Streptococcus-pneumoniae_COC69037.1

MKSKRMEFHNKFLIVSAMLAVISWLSLGVVSFPMLAGTLGISTKAAATVVNLISAYSTVTAVISIVGAITGVGSIGSGIAATVLYILKKKGAAKAALW

>Streptococcus-pneumoniae_COH09421.1

MKSKRMEFHNKFLIVSAMLAVISWLSLGVVSFPMLAGTLGISTKAAATVVNLISAYSTVTAVISIVGAITGVGSIGSGIAATVLYILKKKGAAKAALW

>Streptococcus-pneumoniae_COS62455.1

MKSKRMEFHNKFLIVSAMLAVISWLSLGVVSFPMLAGTLGISTKAAATVVNLISAYSTVTAVISIVGAITGVGSIGSGIAATVLYILKKKGAAKAALW

>Streptococcus-pneumoniae_COS63116.1

MKSKRMEFHNKFLIVSAMLAVISWLSLGVVSFPMLAGTLGISTKAAATVVNLISAYSTVTAVISIVGAITGVGSIGSGIAATVLYILKKKGAAKAALW

>Streptococcus-pneumoniae_COS69674.1

MKSKRMEFHNKFLIVSAMLAVISWLSLGVVSFPMLAGTLGISTKAAATVVNLISAYSTVTAVISIVGAITGVGSIGSGIAATVLYILKKKGAAKAALW

>Streptococcus-pneumoniae_COS64910.1

MKSKRMEFHNKFLIVSAMLAVISWLSLGVVSFPMLAGTLGISTKAAATVVNLISAYSTVTAVISIVGAITGVGSIGSGIAATVLYILKKKGAAKAALW

>Streptococcus-pneumoniae_COS94449.1

MKSKRMEFHNKFLIVSAMLAVISWLSLGVVSFPMLAGTLGISTKAAATVVNLISAYSTVTAVISIVGAITGVGSIGSGIAATVLYILKKKGAAKAALW

>Streptococcus-pneumoniae_COT03938.1

MKSKRMEFHNKFLIVSAMLAVISWLSLGVVSFPMLAGTLGISTKAAATVVNLISAYSTVTAVISIVGAITGVGSIGSGIAATVLYILKKKGAAKAALW

>Streptococcus-pneumoniae_COS98679.1

MKSKRMEFHNKFLIVSAMLAVISWLSLGVVSFPMLAGTLGISTKAAATVVNLISAYSTVTAVISIVGAITGVGSIGSGIAATVLYILKKKGAAKAALW

>Streptococcus-pneumoniae_COT09131.1

MKSKRMEFHNKFLIVSAMLAVISWLSLGVVSFPMLAGTLGISTKAAATVVNLISAYSTVTAVISIVGAITGVGSIGSGIAATVLYILKKKGAAKAALW

>Streptococcus-pneumoniae_CTK26513.1

MKSKRMEFHNKFLIVSAMLAVISWLSLGVVSFPMLAGTLGISTKAAATVVNLISAYSTVTAVISIVGAITGVGSIGSGIAATVLYILKKKGAAKAALW

>Streptococcus-pneumoniae_CTP79016.1

MKSKRMEFHNKFLIVSAMLAVISWLSLGVVSFPMLAGTLGISTKAAATVVNLISAYSTVTAVISIVGAITGVGSIGSGIAATVLYILKKKGAAKAALW

>Streptococcus-pneumoniae_CTI86666.1

MKSKRMEFHNKFLIVSAMLAVISWLSLGVVSFPMLAGTLGISTKAAATVVNLISAYSTVTAVISIVGAITGVGSIGSGIAATVLYILKKKGAAKAALW

>Streptococcus-pneumoniae_CTI71209.1

MKSKRMEFHNKFLIVSAMLAVISWLSLGVVSFPMLAGTLGISTKAAATVVNLISAYSTVTAVISIVGAITGVGSIGSGIAATVLYILKKKGAAKAALW

>Streptococcus-pneumoniae_CTH91186.1

MKSKRMEFHNKFLIVSAMLAVISWLSLGVVSFPMLAGTLGISTKAAATVVNLISAYSTVTAVISIVGAITGVGSIGSGIAATVLYILKKKGAAKAALW

>Streptococcus-pneumoniae_CTI75149.1

MKSKRMEFHNKFLIVSAMLAVISWLSLGVVSFPMLAGTLGISTKAAATVVNLISAYSTVTAVISIVGAITGVGSIGSGIAATVLYILKKKGAAKAALW

>Streptococcus-pneumoniae_CTH75580.1

MKSKRMEFHNKFLIVSAMLAVISWLSLGVVSFPMLAGTLGISTKAAATVVNLISAYSTVTAVISIVGAITGVGSIGSGIAATVLYILKKKGAAKAALW

>Streptococcus-pneumoniae_CTI25255.1

MKSKRMEFHNKFLIVSAMLAVISWLSLGVVSFPMLAGTLGISTKAAATVVNLISAYSTVTAVISIVGAITGVGSIGSGIAATVLYILKKKGAAKAALW

>Streptococcus-pneumoniae_CTM25091.1

MKSKRMEFHNKFLIVSAMLAVISWLSLGVVSFPMLAGTLGISTKAAATVVNLISAYSTVTAVISIVGAITGVGSIGSGIAATVLYILKKKGAAKAALW

>Streptococcus-pneumoniae_CTJ78171.1

MKSKRMEFHNKFLIVSAMLAVISWLSLGVVSFPMLAGTLGISTKAAATVVNLISAYSTVTAVISIVGAITGVGSIGSGIAATVLYILKKKGAAKAALW

>Streptococcus-pneumoniae_CTK10257.1

MKSKRMEFHNKFLIVSAMLAVISWLSLGVVSFPMLAGTLGISTKAAATVVNLISAYSTVTAVISIVGAITGVGSIGSGIAATVLYILKKKGAAKAALW

>Streptococcus-pneumoniae_CTI58732.1

MKSKRMEFHNKFLIVSAMLAVISWLSLGVVSFPMLAGTLGISTKAAATVVNLISAYSTVTAVISIVGAITGVGSIGSGIAATVLYILKKKGAAKAALW

>Streptococcus-pneumoniae_CTC64450.1

MKSKRMEFHNKFLIVSAMLAVISWLSLGVVSFPMLAGTLGISTKAAATVVNLISAYSTVTAVISIVGAITGVGSIGSGIAATVLYILKKKGAAKAALW

>Streptococcus-pneumoniae_CTK36114.1

MKSKRMEFHNKFLIVSAMLAVISWLSLGVVSFPMLAGTLGISTKAAATVVNLISAYSTVTAVISIVGAITGVGSIGSGIAATVLYILKKKGAAKAALW

>Streptococcus-pneumoniae_CTE89280.1

MKSKRMEFHNKFLIVSAMLAVISWLSLGVVSFPMLAGTLGISTKAAATVVNLISAYSTVTAVISIVGAITGVGSIGSGIAATVLYILKKKGAAKAALW

>Streptococcus-pneumoniae_CTJ80552.1

MKSKRMEFHNKFLIVSAMLAVISWLSLGVVSFPMLAGTLGISTKAAATVVNLISAYSTVTAVISIVGAITGVGSIGSGIAATVLYILKKKGAAKAALW

>Streptococcus-pneumoniae_CTH44696.1

MKSKRMEFHNKFLIVSAMLAVISWLSLGVVSFPMLAGTLGISTKAAATVVNLISAYSTVTAVISIVGAITGVGSIGSGIAATVLYILKKKGAAKAALW

>Streptococcus-pneumoniae_CTI95728.1

MKSKRMEFHNKFLIVSAMLAVISWLSLGVVSFPMLAGTLGISTKAAATVVNLISAYSTVTAVISIVGAITGVGSIGSGIAATVLYILKKKGAAKAALW

>Streptococcus-pneumoniae_CTC96059.1

MKSKRMEFHNKFLIVSAMLAVISWLSLGVVSFPMLAGTLGISTKAAATVVNLISAYSTVTAVISIVGAITGVGSIGSGIAATVLYILKKKGAAKAALW

>Streptococcus-pneumoniae_CTI94273.1

MKSKRMEFHNKFLIVSAMLAVISWLSLGVVSFPMLAGTLGISTKAAATVVNLISAYSTVTAVISIVGAITGVGSIGSGIAATVLYILKKKGAAKAALW

>Streptococcus-pneumoniae_CTK36430.1

MKSKRMEFHNKFLIVSAMLAVISWLSLGVVSFPMLAGTLGISTKAAATVVNLISAYSTVTAVISIVGAITGVGSIGSGIAATVLYILKKKGAAKAALW

>Streptococcus-pneumoniae_CTG41041.1

MKSKRMEFHNKFLIVSAMLAVISWLSLGVVSFPMLAGTLGISTKAAATVVNLISAYSTVTAVISIVGAITGVGSIGSGIAATVLYILKKKGAAKAALW

>Streptococcus-pneumoniae_CTE44583.1

MKSKRMEFHNKFLIVSAMLAVISWLSLGVVSFPMLAGTLGISTKAAATVVNLISAYSTVTAVISIVGAITGVGSIGSGIAATVLYILKKKGAAKAALW

>Streptococcus-pneumoniae_CTO36871.1

MKSKRMEFHNKFLIVSAMLAVISWLSLGVVSFPMLAGTLGISTKAAATVVNLISAYSTVTAVISIVGAITGVGSIGSGIAATVLYILKKKGAAKAALW

>Streptococcus-pneumoniae_CTJ55325.1

MKSKRMEFHNKFLIVSAMLAVISWLSLGVVSFPMLAGTLGISTKAAATVVNLISAYSTVTAVISIVGAITGVGSIGSGIAATVLYILKKKGAAKAALW

>Streptococcus-pneumoniae_CTN91366.1

MKSKRMEFHNKFLIVSAMLAVISWLSLGVVSFPMLAGTLGISTKAAATVVNLISAYSTVTAVISIVGAITGVGSIGSGIAATVLYILKKKGAAKAALW

>Streptococcus-pneumoniae_CTJ32417.1

MKSKRMEFHNKFLIVSAMLAVISWLSLGVVSFPMLAGTLGISTKAAATVVNLISAYSTVTAVISIVGAITGVGSIGSGIAATVLYILKKKGAAKAALW

>Streptococcus-pneumoniae_CTH96045.1

MKSKRMEFHNKFLIVSAMLAVISWLSLGVVSFPMLAGTLGISTKAAATVVNLISAYSTVTAVISIVGAITGVGSIGSGIAATVLYILKKKGAAKAALW

>Streptococcus-pneumoniae_CTH55794.1

MKSKRMEFHNKFLIVSAMLAVISWLSLGVVSFPMLAGTLGISTKAAATVVNLISAYSTVTAVISIVGAITGVGSIGSGIAATVLYILKKKGAAKAALW

>Streptococcus-pneumoniae_CTK35845.1

MKSKRMEFHNKFLIVSAMLAVISWLSLGVVSFPMLAGTLGISTKAAATVVNLISAYSTVTAVISIVGAITGVGSIGSGIAATVLYILKKKGAAKAALW

>Streptococcus-pneumoniae_CTC64336.1

MKSKRMEFHNKFLIVSAMLAVISWLSLGVVSFPMLAGTLGISTKAAATVVNLISAYSTVTAVISIVGAITGVGSIGSGIAATVLYILKKKGAAKAALW

>Streptococcus-pneumoniae_CTD78966.1

MKSKRMEFHNKFLIVSAMLAVISWLSLGVVSFPMLAGTLGISTKAAATVVNLISAYSTVTAVISIVGAITGVGSIGSGIAATVLYILKKKGAAKAALW

>Streptococcus-pneumoniae_CTK42821.1

MKSKRMEFHNKFLIVSAMLAVISWLSLGVVSFPMLAGTLGISTKAAATVVNLISAYSTVTAVISIVGAITGVGSIGSGIAATVLYILKKKGAAKAALW

>Streptococcus-pneumoniae_CTI16622.1

MKSKRMEFHNKFLIVSAMLAVISWLSLGVVSFPMLAGTLGISTKAAATVVNLISAYSTVTAVISIVGAITGVGSIGSGIAATVLYILKKKGAAKAALW

>Streptococcus-pneumoniae_CTK51414.1

MKSKRMEFHNKFLIVSAMLAVISWLSLGVVSFPMLAGTLGISTKAAATVVNLISAYSTVTAVISIVGAITGVGSIGSGIAATVLYILKKKGAAKAALW

>Streptococcus-pneumoniae_CTD88668.1

MKSKRMEFHNKFLIVSAMLAVISWLSLGVVSFPMLAGTLGISTKAAATVVNLISAYSTVTAVISIVGAITGVGSIGSGIAATVLYILKKKGAAKAALW

>Streptococcus-pneumoniae_CTN88413.1

MKSKRMEFHNKFLIVSAMLAVISWLSLGVVSFPMLAGTLGISTKAAATVVNLISAYSTVTAVISIVGAITGVGSIGSGIAATVLYILKKKGAAKAALW

>Streptococcus-pneumoniae_CTJ00883.1

MKSKRMEFHNKFLIVSAMLAVISWLSLGVVSFPMLAGTLGISTKAAATVVNLISAYSTVTAVISIVGAITGVGSIGSGIAATVLYILKKKGAAKAALW

>Streptococcus-pneumoniae_CTG55486.1

MKSKRMEFHNKFLIVSAMLAVISWLSLGVVSFPMLAGTLGISTKAAATVVNLISAYSTVTAVISIVGAITGVGSIGSGIAATVLYILKKKGAAKAALW

>Streptococcus-pneumoniae_CTH36544.1

MKSKRMEFHNKFLIVSAMLAVISWLSLGVVSFPMLAGTLGISTKAAATVVNLISAYSTVTAVISIVGAITGVGSIGSGIAATVLYILKKKGAAKAALW

>Streptococcus-pneumoniae_CTK04493.1

MKSKRMEFHNKFLIVSAMLAVISWLSLGVVSFPMLAGTLGISTKAAATVVNLISAYSTVTAVISIVGAITGVGSIGSGIAATVLYILKKKGAAKAALW

>Streptococcus-pneumoniae_CTE01417.1

MKSKRMEFHNKFLIVSAMLAVISWLSLGVVSFPMLAGTLGISTKAAATVVNLISAYSTVTAVISIVGAITGVGSIGSGIAATVLYILKKKGAAKAALW

>Streptococcus-pneumoniae_CTO29417.1

MKSKRMEFHNKFLIVSAMLAVISWLSLGVVSFPMLAGTLGISTKAAATVVNLISAYSTVTAVISIVGAITGVGSIGSGIAATVLYILKKKGAAKAALW

>Streptococcus-pneumoniae_CTG31237.1

MKSKRMEFHNKFLIVSAMLAVISWLSLGVVSFPMLAGTLGISTKAAATVVNLISAYSTVTAVISIVGAITGVGSIGSGIAATVLYILKKKGAAKAALW

>Streptococcus-pneumoniae_CTJ42561.1

MKSKRMEFHNKFLIVSAMLAVISWLSLGVVSFPMLAGTLGISTKAAATVVNLISAYSTVTAVISIVGAITGVGSIGSGIAATVLYILKKKGAAKAALW

>Streptococcus-pneumoniae_CTE32374.1

MKSKRMEFHNKFLIVSAMLAVISWLSLGVVSFPMLAGTLGISTKAAATVVNLISAYSTVTAVISIVGAITGVGSIGSGIAATVLYILKKKGAAKAALW

>Streptococcus-pneumoniae_CTH01040.1

MKSKRMEFHNKFLIVSAMLAVISWLSLGVVSFPMLAGTLGISTKAAATVVNLISAYSTVTAVISIVGAITGVGSIGSGIAATVLYILKKKGAAKAALW

>Streptococcus-pneumoniae_CTK44698.1

MKSKRMEFHNKFLIVSAMLAVISWLSLGVVSFPMLAGTLGISTKAAATVVNLISAYSTVTAVISIVGAITGVGSIGSGIAATVLYILKKKGAAKAALW

>Streptococcus-pneumoniae_CTJ50147.1

MKSKRMEFHNKFLIVSAMLAVISWLSLGVVSFPMLAGTLGISTKAAATVVNLISAYSTVTAVISIVGAITGVGSIGSGIAATVLYILKKKGAAKAALW

>Streptococcus-pneumoniae_CTJ79290.1

MKSKRMEFHNKFLIVSAMLAVISWLSLGVVSFPMLAGTLGISTKAAATVVNLISAYSTVTAVISIVGAITGVGSIGSGIAATVLYILKKKGAAKAALW

>Streptococcus-pneumoniae_CTH87708.1

MKSKRMEFHNKFLIVSAMLAVISWLSLGVVSFPMLAGTLGISTKAAATVVNLISAYSTVTAVISIVGAITGVGSIGSGIAATVLYILKKKGAAKAALW

>Streptococcus-pneumoniae_CTI76579.1

MKSKRMEFHNKFLIVSAMLAVISWLSLGVVSFPMLAGTLGISTKAAATVVNLISAYSTVTAVISIVGAITGVGSIGSGIAATVLYILKKKGAAKAALW

>Streptococcus-pneumoniae_CTK67925.1

MKSKRMEFHNKFLIVSAMLAVISWLSLGVVSFPMLAGTLGISTKAAATVVNLISAYSTVTAVISIVGAITGVGSIGSGIAATVLYILKKKGAAKAALW

>Streptococcus-pneumoniae_CTH48937.1

MKSKRMEFHNKFLIVSAMLAVISWLSLGVVSFPMLAGTLGISTKAAATVVNLISAYSTVTAVISIVGAITGVGSIGSGIAATVLYILKKKGAAKAALW

>Streptococcus-pneumoniae_CTI53357.1

MKSKRMEFHNKFLIVSAMLAVISWLSLGVVSFPMLAGTLGISTKAAATVVNLISAYSTVTAVISIVGAITGVGSIGSGIAATVLYILKKKGAAKAALW

>Streptococcus-pneumoniae_CTD90041.1

MKSKRMEFHNKFLIVSAMLAVISWLSLGVVSFPMLAGTLGISTKAAATVVNLISAYSTVTAVISIVGAITGVGSIGSGIAATVLYILKKKGAAKAALW

>Streptococcus-pneumoniae_CTI50504.1

MKSKRMEFHNKFLIVSAMLAVISWLSLGVVSFPMLAGTLGISTKAAATVVNLISAYSTVTAVISIVGAITGVGSIGSGIAATVLYILKKKGAAKAALW

>Streptococcus-pneumoniae_CTJ39352.1

MKSKRMEFHNKFLIVSAMLAVISWLSLGVVSFPMLAGTLGISTKAAATVVNLISAYSTVTAVISIVGAITGVGSIGSGIAATVLYILKKKGAAKAALW

>Streptococcus-pneumoniae_CTO09455.1

MKSKRMEFHNKFLIVSAMLAVISWLSLGVVSFPMLAGTLGISTKAAATVVNLISAYSTVTAVISIVGAITGVGSIGSGIAATVLYILKKKGAAKAALW

>Streptococcus-pneumoniae_CTI84708.1

MKSKRMEFHNKFLIVSAMLAVISWLSLGVVSFPMLAGTLGISTKAAATVVNLISAYSTVTAVISIVGAITGVGSIGSGIAATVLYILKKKGAAKAALW

>Streptococcus-pneumoniae_CTJ50284.1

MKSKRMEFHNKFLIVSAMLAVISWLSLGVVSFPMLAGTLGISTKAAATVVNLISAYSTVTAVISIVGAITGVGSIGSGIAATVLYILKKKGAAKAALW

>Streptococcus-pneumoniae_CTN32177.1

MKSKRMEFHNKFLIVSAMLAVISWLSLGVVSFPMLAGTLGISTKAAATVVNLISAYSTVTAVISIVGAITGVGSIGSGIAATVLYILKKKGAAKAALW

>Streptococcus-pneumoniae_CTK65217.1

MKSKRMEFHNKFLIVSAMLAVISWLSLGVVSFPMLAGTLGISTKAAATVVNLISAYSTVTAVISIVGAITGVGSIGSGIAATVLYILKKKGAAKAALW

>Streptococcus-pneumoniae_CTG16210.1

MKSKRMEFHNKFLIVSAMLAVISWLSLGVVSFPMLAGTLGISTKAAATVVNLISAYSTVTAVISIVGAITGVGSIGSGIAATVLYILKKKGAAKAALW

>Streptococcus-pneumoniae_CTK12773.1

MKSKRMEFHNKFLIVSAMLAVISWLSLGVVSFPMLAGTLGISTKAAATVVNLISAYSTVTAVISIVGAITGVGSIGSGIAATVLYILKKKGAAKAALW

>Streptococcus-pneumoniae_CTE25729.1

MKSKRMEFHNKFLIVSAMLAVISWLSLGVVSFPMLAGTLGISTKAAATVVNLISAYSTVTAVISIVGAITGVGSIGSGIAATVLYILKKKGAAKAALW

>Streptococcus-pneumoniae_CTH76180.1

MKSKRMEFHNKFLIVSAMLAVISWLSLGVVSFPMLAGTLGISTKAAATVVNLISAYSTVTAVISIVGAITGVGSIGSGIAATVLYILKKKGAAKAALW

>Streptococcus-pneumoniae_CTI00366.1

MKSKRMEFHNKFLIVSAMLAVISWLSLGVVSFPMLAGTLGISTKAAATVVNLISAYSTVTAVISIVGAITGVGSIGSGIAATVLYILKKKGAAKAALW

>Streptococcus-pneumoniae_CTK76386.1

MKSKRMEFHNKFLIVSAMLAVISWLSLGVVSFPMLAGTLGISTKAAATVVNLISAYSTVTAVISIVGAITGVGSIGSGIAATVLYILKKKGAAKAALW

>Streptococcus-pneumoniae_CTI01870.1

MKSKRMEFHNKFLIVSAMLAVISWLSLGVVSFPMLAGTLGISTKAAATVVNLISAYSTVTAVISIVGAITGVGSIGSGIAATVLYILKKKGAAKAALW

>Streptococcus-pneumoniae_CTG37877.1

MKSKRMEFHNKFLIVSAMLAVISWLSLGVVSFPMLAGTLGISTKAAATVVNLISAYSTVTAVISIVGAITGVGSIGSGIAATVLYILKKKGAAKAALW

>Streptococcus-pneumoniae_CTJ35727.1

MKSKRMEFHNKFLIVSAMLAVISWLSLGVVSFPMLAGTLGISTKAAATVVNLISAYSTVTAVISIVGAITGVGSIGSGIAATVLYILKKKGAAKAALW

>Streptococcus-pneumoniae_CTN30516.1

MKSKRMEFHNKFLIVSAMLAVISWLSLGVVSFPMLAGTLGISTKAAATVVNLISAYSTVTAVISIVGAITGVGSIGSGIAATVLYILKKKGAAKAALW

>Streptococcus-pneumoniae_CTJ73721.1

MKSKRMEFHNKFLIVSAMLAVISWLSLGVVSFPMLAGTLGISTKAAATVVNLISAYSTVTAVISIVGAITGVGSIGSGIAATVLYILKKKGAAKAALW

>Streptococcus-pneumoniae_CTJ36092.1

MKSKRMEFHNKFLIVSAMLAVISWLSLGVVSFPMLAGTLGISTKAAATVVNLISAYSTVTAVISIVGAITGVGSIGSGIAATVLYILKKKGAAKAALW

>Streptococcus-pneumoniae_CTK86986.1

MKSKRMEFHNKFLIVSAMLAVISWLSLGVVSFPMLAGTLGISTKAAATVVNLISAYSTVTAVISIVGAITGVGSIGSGIAATVLYILKKKGAAKAALW

>Streptococcus-pneumoniae_CTJ58640.1

MKSKRMEFHNKFLIVSAMLAVISWLSLGVVSFPMLAGTLGISTKAAATVVNLISAYSTVTAVISIVGAITGVGSIGSGIAATVLYILKKKGAAKAALW

>Streptococcus-pneumoniae_CTJ57947.1

MKSKRMEFHNKFLIVSAMLAVISWLSLGVVSFPMLAGTLGISTKAAATVVNLISAYSTVTAVISIVGAITGVGSIGSGIAATVLYILKKKGAAKAALW

>Streptococcus-pneumoniae_CTO09024.1

MKSKRMEFHNKFLIVSAMLAVISWLSLGVVSFPMLAGTLGISTKAAATVVNLISAYSTVTAVISIVGAITGVGSIGSGIAATVLYILKKKGAAKAALW

>Streptococcus-pneumoniae_CTK81935.1

MKSKRMEFHNKFLIVSAMLAVISWLSLGVVSFPMLAGTLGISTKAAATVVNLISAYSTVTAVISIVGAITGVGSIGSGIAATVLYILKKKGAAKAALW

>Streptococcus-pneumoniae_CTO23979.1

MKSKRMEFHNKFLIVSAMLAVISWLSLGVVSFPMLAGTLGISTKAAATVVNLISAYSTVTAVISIVGAITGVGSIGSGIAATVLYILKKKGAAKAALW

>Streptococcus-pneumoniae_CTO05502.1

MKSKRMEFHNKFLIVSAMLAVISWLSLGVVSFPMLAGTLGISTKAAATVVNLISAYSTVTAVISIVGAITGVGSIGSGIAATVLYILKKKGAAKAALW

>Streptococcus-pneumoniae_CTI31414.1

MKSKRMEFHNKFLIVSAMLAVISWLSLGVVSFPMLAGTLGISTKAAATVVNLISAYSTVTAVISIVGAITGVGSIGSGIAATVLYILKKKGAAKAALW

>Streptococcus-pneumoniae_CTJ67815.1

MKSKRMEFHNKFLIVSAMLAVISWLSLGVVSFPMLAGTLGISTKAAATVVNLISAYSTVTAVISIVGAITGVGSIGSGIAATVLYILKKKGAAKAALW

>Streptococcus-pneumoniae_CTJ31599.1

MKSKRMEFHNKFLIVSAMLAVISWLSLGVVSFPMLAGTLGISTKAAATVVNLISAYSTVTAVISIVGAITGVGSIGSGIAATVLYILKKKGAAKAALW

>Streptococcus-pneumoniae_CTK82327.1

MKSKRMEFHNKFLIVSAMLAVISWLSLGVVSFPMLAGTLGISTKAAATVVNLISAYSTVTAVISIVGAITGVGSIGSGIAATVLYILKKKGAAKAALW

>Streptococcus-pneumoniae_CTJ02351.1

MKSKRMEFHNKFLIVSAMLAVISWLSLGVVSFPMLAGTLGISTKAAATVVNLISAYSTVTAVISIVGAITGVGSIGSGIAATVLYILKKKGAAKAALW

>Streptococcus-pneumoniae_CTK02823.1

MKSKRMEFHNKFLIVSAMLAVISWLSLGVVSFPMLAGTLGISTKAAATVVNLISAYSTVTAVISIVGAITGVGSIGSGIAATVLYILKKKGAAKAALW

>Streptococcus-pneumoniae_CTK31432.1

MKSKRMEFHNKFLIVSAMLAVISWLSLGVVSFPMLAGTLGISTKAAATVVNLISAYSTVTAVISIVGAITGVGSIGSGIAATVLYILKKKGAAKAALW

>Streptococcus-pneumoniae_CTD89154.1

MKSKRMEFHNKFLIVSAMLAVISWLSLGVVSFPMLAGTLGISTKAAATVVNLISAYSTVTAVISIVGAITGVGSIGSGIAATVLYILKKKGAAKAALW

>Streptococcus-pneumoniae_CTK14511.1

MKSKRMEFHNKFLIVSAMLAVISWLSLGVVSFPMLAGTLGISTKAAATVVNLISAYSTVTAVISIVGAITGVGSIGSGIAATVLYILKKKGAAKAALW

>Streptococcus-pneumoniae_CTK41601.1

MKSKRMEFHNKFLIVSAMLAVISWLSLGVVSFPMLAGTLGISTKAAATVVNLISAYSTVTAVISIVGAITGVGSIGSGIAATVLYILKKKGAAKAALW

>Streptococcus-pneumoniae_CTJ84310.1

MKSKRMEFHNKFLIVSAMLAVISWLSLGVVSFPMLAGTLGISTKAAATVVNLISAYSTVTAVISIVGAITGVGSIGSGIAATVLYILKKKGAAKAALW

>Streptococcus-pneumoniae_CTE35879.1

MKSKRMEFHNKFLIVSAMLAVISWLSLGVVSFPMLAGTLGISTKAAATVVNLISAYSTVTAVISIVGAITGVGSIGSGIAATVLYILKKKGAAKAALW

>Streptococcus-pneumoniae_CTK54455.1

MKSKRMEFHNKFLIVSAMLAVISWLSLGVVSFPMLAGTLGISTKAAATVVNLISAYSTVTAVISIVGAITGVGSIGSGIAATVLYILKKKGAAKAALW

>Streptococcus-pneumoniae_CTC80673.1

MKSKRMEFHNKFLIVSAMLAVISWLSLGVVSFPMLAGTLGISTKAAATVVNLISAYSTVTAVISIVGAITGVGSIGSGIAATVLYILKKKGAAKAALW

>Streptococcus-pneumoniae_CTD10620.1

MKSKRMEFHNKFLIVSAMLAVISWLSLGVVSFPMLAGTLGISTKAAATVVNLISAYSTVTAVISIVGAITGVGSIGSGIAATVLYILKKKGAAKAALW

>Streptococcus-pneumoniae_CTE34143.1

MKSKRMEFHNKFLIVSAMLAVISWLSLGVVSFPMLAGTLGISTKAAATVVNLISAYSTVTAVISIVGAITGVGSIGSGIAATVLYILKKKGAAKAALW

>Streptococcus-pneumoniae_CTK60624.1

MKSKRMEFHNKFLIVSAMLAVISWLSLGVVSFPMLAGTLGISTKAAATVVNLISAYSTVTAVISIVGAITGVGSIGSGIAATVLYILKKKGAAKAALW

>Streptococcus-pneumoniae_CTH86042.1

MKSKRMEFHNKFLIVSAMLAVISWLSLGVVSFPMLAGTLGISTKAAATVVNLISAYSTVTAVISIVGAITGVGSIGSGIAATVLYILKKKGAAKAALW

>Streptococcus-pneumoniae_CTH38350.1

MKSKRMEFHNKFLIVSAMLAVISWLSLGVVSFPMLAGTLGISTKAAATVVNLISAYSTVTAVISIVGAITGVGSIGSGIAATVLYILKKKGAAKAALW

>Streptococcus-pneumoniae_CTD72941.1

MKSKRMEFHNKFLIVSAMLAVISWLSLGVVSFPMLAGTLGISTKAAATVVNLISAYSTVTAVISIVGAITGVGSIGSGIAATVLYILKKKGAAKAALW

>Streptococcus-pneumoniae_CTH61029.1

MKSKRMEFHNKFLIVSAMLAVISWLSLGVVSFPMLAGTLGISTKAAATVVNLISAYSTVTAVISIVGAITGVGSIGSGIAATVLYILKKKGAAKAALW

>Streptococcus-pneumoniae_CTH45053.1

MKSKRMEFHNKFLIVSAMLAVISWLSLGVVSFPMLAGTLGISTKAAATVVNLISAYSTVTAVISIVGAITGVGSIGSGIAATVLYILKKKGAAKAALW

>Streptococcus-pneumoniae_CTK50504.1

MKSKRMEFHNKFLIVSAMLAVISWLSLGVVSFPMLAGTLGISTKAAATVVNLISAYSTVTAVISIVGAITGVGSIGSGIAATVLYILKKKGAAKAALW

>Streptococcus-pneumoniae_CTP52399.1

MKSKRMEFHNKFLIVSAMLAVISWLSLGVVSFPMLAGTLGISTKAAATVVNLISAYSTVTAVISIVGAITGVGSIGSGIAATVLYILKKKGAAKAALW

>Streptococcus-pneumoniae_CTN47942.1

MKSKRMEFHNKFLIVSAMLAVISWLSLGVVSFPMLAGTLGISTKAAATVVNLISAYSTVTAVISIVGAITGVGSIGSGIAATVLYILKKKGAAKAALW

>Streptococcus-pneumoniae_CTI14980.1

MKSKRMEFHNKFLIVSAMLAVISWLSLGVVSFPMLAGTLGISTKAAATVVNLISAYSTVTAVISIVGAITGVGSIGSGIAATVLYILKKKGAAKAALW

>Streptococcus-pneumoniae_CTH89223.1

MKSKRMEFHNKFLIVSAMLAVISWLSLGVVSFPMLAGTLGISTKAAATVVNLISAYSTVTAVISIVGAITGVGSIGSGIAATVLYILKKKGAAKAALW

>Streptococcus-pneumoniae_CTJ70506.1

MKSKRMEFHNKFLIVSAMLAVISWLSLGVVSFPMLAGTLGISTKAAATVVNLISAYSTVTAVISIVGAITGVGSIGSGIAATVLYILKKKGAAKAALW

>Streptococcus-pneumoniae_CTO01747.1

MKSKRMEFHNKFLIVSAMLAVISWLSLGVVSFPMLAGTLGISTKAAATVVNLISAYSTVTAVISIVGAITGVGSIGSGIAATVLYILKKKGAAKAALW

>Streptococcus-pneumoniae_CTJ72367.1

MKSKRMEFHNKFLIVSAMLAVISWLSLGVVSFPMLAGTLGISTKAAATVVNLISAYSTVTAVISIVGAITGVGSIGSGIAATVLYILKKKGAAKAALW

>Streptococcus-pneumoniae_CTD78117.1

MKSKRMEFHNKFLIVSAMLAVISWLSLGVVSFPMLAGTLGISTKAAATVVNLISAYSTVTAVISIVGAITGVGSIGSGIAATVLYILKKKGAAKAALW

>Streptococcus-pneumoniae_CTK46587.1

MKSKRMEFHNKFLIVSAMLAVISWLSLGVVSFPMLAGTLGISTKAAATVVNLISAYSTVTAVISIVGAITGVGSIGSGIAATVLYILKKKGAAKAALW

>Streptococcus-pneumoniae_CTD70544.1

MKSKRMEFHNKFLIVSAMLAVISWLSLGVVSFPMLAGTLGISTKAAATVVNLISAYSTVTAVISIVGAITGVGSIGSGIAATVLYILKKKGAAKAALW

>Streptococcus-pneumoniae_CTI24636.1

MKSKRMEFHNKFLIVSAMLAVISWLSLGVVSFPMLAGTLGISTKAAATVVNLISAYSTVTAVISIVGAITGVGSIGSGIAATVLYILKKKGAAKAALW

>Streptococcus-pneumoniae_CTJ16847.1

MKSKRMEFHNKFLIVSAMLAVISWLSLGVVSFPMLAGTLGISTKAAATVVNLISAYSTVTAVISIVGAITGVGSIGSGIAATVLYILKKKGAAKAALW

>Streptococcus-pneumoniae_CTD92419.1

MKSKRMEFHNKFLIVSAMLAVISWLSLGVVSFPMLAGTLGISTKAAATVVNLISAYSTVTAVISIVGAITGVGSIGSGIAATVLYILKKKGAAKAALW

>Streptococcus-pneumoniae_CTI18548.1

MKSKRMEFHNKFLIVSAMLAVISWLSLGVVSFPMLAGTLGISTKAAATVVNLISAYSTVTAVISIVGAITGVGSIGSGIAATVLYILKKKGAAKAALW

>Streptococcus-pneumoniae_CTK32941.1

MKSKRMEFHNKFLIVSAMLAVISWLSLGVVSFPMLAGTLGISTKAAATVVNLISAYSTVTAVISIVGAITGVGSIGSGIAATVLYILKKKGAAKAALW

>Streptococcus-pneumoniae_CTJ41606.1

MKSKRMEFHNKFLIVSAMLAVISWLSLGVVSFPMLAGTLGISTKAAATVVNLISAYSTVTAVISIVGAITGVGSIGSGIAATVLYILKKKGAAKAALW

>Streptococcus-pneumoniae_CTI23697.1

MKSKRMEFHNKFLIVSAMLAVISWLSLGVVSFPMLAGTLGISTKAAATVVNLISAYSTVTAVISIVGAITGVGSIGSGIAATVLYILKKKGAAKAALW

>Streptococcus-pneumoniae_CTE23960.1

MKSKRMEFHNKFLIVSAMLAVISWLSLGVVSFPMLAGTLGISTKAAATVVNLISAYSTVTAVISIVGAITGVGSIGSGIAATVLYILKKKGAAKAALW

>Streptococcus-pneumoniae_CTH64122.1

MKSKRMEFHNKFLIVSAMLAVISWLSLGVVSFPMLAGTLGISTKAAATVVNLISAYSTVTAVISIVGAITGVGSIGSGIAATVLYILKKKGAAKAALW

>Streptococcus-pneumoniae_CTD67977.1

MKSKRMEFHNKFLIVSAMLAVISWLSLGVVSFPMLAGTLGISTKAAATVVNLISAYSTVTAVISIVGAITGVGSIGSGIAATVLYILKKKGAAKAALW

>Streptococcus-pneumoniae_CTD98125.1

MKSKRMEFHNKFLIVSAMLAVISWLSLGVVSFPMLAGTLGISTKAAATVVNLISAYSTVTAVISIVGAITGVGSIGSGIAATVLYILKKKGAAKAALW

>Streptococcus-pneumoniae_CTD66145.1

MKSKRMEFHNKFLIVSAMLAVISWLSLGVVSFPMLAGTLGISTKAAATVVNLISAYSTVTAVISIVGAITGVGSIGSGIAATVLYILKKKGAAKAALW

>Streptococcus-pneumoniae_CTN24400.1

MKSKRMEFHNKFLIVSAMLAVISWLSLGVVSFPMLAGTLGISTKAAATVVNLISAYSTVTAVISIVGAITGVGSIGSGIAATVLYILKKKGAAKAALW

>Streptococcus-pneumoniae_CTO32799.1

MKSKRMEFHNKFLIVSAMLAVISWLSLGVVSFPMLAGTLGISTKAAATVVNLISAYSTVTAVISIVGAITGVGSIGSGIAATVLYILKKKGAAKAALW

>Streptococcus-pneumoniae_CTK45363.1

MKSKRMEFHNKFLIVSAMLAVISWLSLGVVSFPMLAGTLGISTKAAATVVNLISAYSTVTAVISIVGAITGVGSIGSGIAATVLYILKKKGAAKAALW

>Streptococcus-pneumoniae_CTG31233.1

MKSKRMEFHNKFLIVSAMLAVISWLSLGVVSFPMLAGTLGISTKAAATVVNLISAYSTVTAVISIVGAITGVGSIGSGIAATVLYILKKKGAAKAALW

>Streptococcus-pneumoniae_CTI09903.1

MKSKRMEFHNKFLIVSAMLAVISWLSLGVVSFPMLAGTLGISTKAAATVVNLISAYSTVTAVISIVGAITGVGSIGSGIAATVLYILKKKGAAKAALW

>Streptococcus-pneumoniae_CTE17742.1

MKSKRMEFHNKFLIVSAMLAVISWLSLGVVSFPMLAGTLGISTKAAATVVNLISAYSTVTAVISIVGAITGVGSIGSGIAATVLYILKKKGAAKAALW

>Streptococcus-pneumoniae_CRZ36387.1

MKSKRMEFHNKFLIVSAMLAVISWLSLGVVSFPMLAGTLGISTKAAATVVNLISAYSTVTAVISIVGAITGVGSIGSGIAATVLYILKKKGAAKAALW

>Streptococcus-pneumoniae_CTN32555.1

MKSKRMEFHNKFLIVSAMLAVISWLSLGVVSFPMLAGTLGISTKAAATVVNLISAYSTVTAVISIVGAITGVGSIGSGIAATVLYILKKKGAAKAALW

>Streptococcus-pneumoniae_CTE04069.1

MKSKRMEFHNKFLIVSAMLAVISWLSLGVVSFPMLAGTLGISTKAAATVVNLISAYSTVTAVISIVGAITGVGSIGSGIAATVLYILKKKGAAKAALW

>Streptococcus-pneumoniae_CTH35306.1

MKSKRMEFHNKFLIVSAMLAVISWLSLGVVSFPMLAGTLGISTKAAATVVNLISAYSTVTAVISIVGAITGVGSIGSGIAATVLYILKKKGAAKAALW

>Streptococcus-pneumoniae_CTH31056.1

MKSKRMEFHNKFLIVSAMLAVISWLSLGVVSFPMLAGTLGISTKAAATVVNLISAYSTVTAVISIVGAITGVGSIGSGIAATVLYILKKKGAAKAALW

>Streptococcus-pneumoniae_CTI41813.1

MKSKRMEFHNKFLIVSAMLAVISWLSLGVVSFPMLAGTLGISTKAAATVVNLISAYSTVTAVISIVGAITGVGSIGSGIAATVLYILKKKGAAKAALW

>Streptococcus-pneumoniae_CTK17938.1

MKSKRMEFHNKFLIVSAMLAVISWLSLGVVSFPMLAGTLGISTKAAATVVNLISAYSTVTAVISIVGAITGVGSIGSGIAATVLYILKKKGAAKAALW

>Streptococcus-pneumoniae_CTD93590.1

MKSKRMEFHNKFLIVSAMLAVISWLSLGVVSFPMLAGTLGISTKAAATVVNLISAYSTVTAVISIVGAITGVGSIGSGIAATVLYILKKKGAAKAALW

>Streptococcus-pneumoniae_CTD53574.1

MKSKRMEFHNKFLIVSAMLAVISWLSLGVVSFPMLAGTLGISTKAAATVVNLISAYSTVTAVISIVGAITGVGSIGSGIAATVLYILKKKGAAKAALW

>Streptococcus-pneumoniae_CTD92435.1

MKSKRMEFHNKFLIVSAMLAVISWLSLGVVSFPMLAGTLGISTKAAATVVNLISAYSTVTAVISIVGAITGVGSIGSGIAATVLYILKKKGAAKAALW

>Streptococcus-pneumoniae_CTH34175.1

MKSKRMEFHNKFLIVSAMLAVISWLSLGVVSFPMLAGTLGISTKAAATVVNLISAYSTVTAVISIVGAITGVGSIGSGIAATVLYILKKKGAAKAALW

>Streptococcus-pneumoniae_CTK05795.1

MKSKRMEFHNKFLIVSAMLAVISWLSLGVVSFPMLAGTLGISTKAAATVVNLISAYSTVTAVISIVGAITGVGSIGSGIAATVLYILKKKGAAKAALW

>Streptococcus-pneumoniae_CTI06250.1

MKSKRMEFHNKFLIVSAMLAVISWLSLGVVSFPMLAGTLGISTKAAATVVNLISAYSTVTAVISIVGAITGVGSIGSGIAATVLYILKKKGAAKAALW

>Streptococcus-pneumoniae_CTN28441.1

MKSKRMEFHNKFLIVSAMLAVISWLSLGVVSFPMLAGTLGISTKAAATVVNLISAYSTVTAVISIVGAITGVGSIGSGIAATVLYILKKKGAAKAALW

>Streptococcus-pneumoniae_CTE08513.1

MKSKRMEFHNKFLIVSAMLAVISWLSLGVVSFPMLAGTLGISTKAAATVVNLISAYSTVTAVISIVGAITGVGSIGSGIAATVLYILKKKGAAKAALW

>Streptococcus-pneumoniae_CTK04441.1

MKSKRMEFHNKFLIVSAMLAVISWLSLGVVSFPMLAGTLGISTKAAATVVNLISAYSTVTAVISIVGAITGVGSIGSGIAATVLYILKKKGAAKAALW

>Streptococcus-pneumoniae_CTI41719.1

MKSKRMEFHNKFLIVSAMLAVISWLSLGVVSFPMLAGTLGISTKAAATVVNLISAYSTVTAVISIVGAITGVGSIGSGIAATVLYILKKKGAAKAALW

>Streptococcus-pneumoniae_CTI37350.1

MKSKRMEFHNKFLIVSAMLAVISWLSLGVVSFPMLAGTLGISTKAAATVVNLISAYSTVTAVISIVGAITGVGSIGSGIAATVLYILKKKGAAKAALW

>Streptococcus-pneumoniae_CTI92240.1

MKSKRMEFHNKFLIVSAMLAVISWLSLGVVSFPMLAGTLGISTKAAATVVNLISAYSTVTAVISIVGAITGVGSIGSGIAATVLYILKKKGAAKAALW

>Streptococcus-pneumoniae_CTI19155.1

MKSKRMEFHNKFLIVSAMLAVISWLSLGVVSFPMLAGTLGISTKAAATVVNLISAYSTVTAVISIVGAITGVGSIGSGIAATVLYILKKKGAAKAALW

>Streptococcus-pneumoniae_CTI45336.1

MKSKRMEFHNKFLIVSAMLAVISWLSLGVVSFPMLAGTLGISTKAAATVVNLISAYSTVTAVISIVGAITGVGSIGSGIAATVLYILKKKGAAKAALW

>Streptococcus-pneumoniae_CTK40831.1

MKSKRMEFHNKFLIVSAMLAVISWLSLGVVSFPMLAGTLGISTKAAATVVNLISAYSTVTAVISIVGAITGVGSIGSGIAATVLYILKKKGAAKAALW

>Streptococcus-pneumoniae_CTK36975.1

MKSKRMEFHNKFLIVSAMLAVISWLSLGVVSFPMLAGTLGISTKAAATVVNLISAYSTVTAVISIVGAITGVGSIGSGIAATVLYILKKKGAAKAALW

>Streptococcus-pneumoniae_CTN86903.1

MKSKRMEFHNKFLIVSAMLAVISWLSLGVVSFPMLAGTLGISTKAAATVVNLISAYSTVTAVISIVGAITGVGSIGSGIAATVLYILKKKGAAKAALW

>Streptococcus-pneumoniae_CTC98619.1

MKSKRMEFHNKFLIVSAMLAVISWLSLGVVSFPMLAGTLGISTKAAATVVNLISAYSTVTAVISIVGAITGVGSIGSGIAATVLYILKKKGAAKAALW

>Streptococcus-pneumoniae_CTI47742.1

MKSKRMEFHNKFLIVSAMLAVISWLSLGVVSFPMLAGTLGISTKAAATVVNLISAYSTVTAVISIVGAITGVGSIGSGIAATVLYILKKKGAAKAALW

>Streptococcus-pneumoniae_CTD83021.1

MKSKRMEFHNKFLIVSAMLAVISWLSLGVVSFPMLAGTLGISTKAAATVVNLISAYSTVTAVISIVGAITGVGSIGSGIAATVLYILKKKGAAKAALW

>Streptococcus-pneumoniae_CTN28124.1

MKSKRMEFHNKFLIVSAMLAVISWLSLGVVSFPMLAGTLGISTKAAATVVNLISAYSTVTAVISIVGAITGVGSIGSGIAATVLYILKKKGAAKAALW

>Streptococcus-pneumoniae_CTJ57550.1

MKSKRMEFHNKFLIVSAMLAVISWLSLGVVSFPMLAGTLGISTKAAATVVNLISAYSTVTAVISIVGAITGVGSIGSGIAATVLYILKKKGAAKAALW

>Streptococcus-pneumoniae_CTG36314.1

MKSKRMEFHNKFLIVSAMLAVISWLSLGVVSFPMLAGTLGISTKAAATVVNLISAYSTVTAVISIVGAITGVGSIGSGIAATVLYILKKKGAAKAALW

>Streptococcus-pneumoniae_CTN19875.1

MKSKRMEFHNKFLIVSAMLAVISWLSLGVVSFPMLAGTLGISTKAAATVVNLISAYSTVTAVISIVGAITGVGSIGSGIAATVLYILKKKGAAKAALW

>Streptococcus-pneumoniae_CTL84954.1

MKSKRMEFHNKFLIVSAMLAVISWLSLGVVSFPMLAGTLGISTKAAATVVNLISAYSTVTAVISIVGAITGVGSIGSGIAATVLYILKKKGAAKAALW

>Streptococcus-pneumoniae_CTI50892.1

MKSKRMEFHNKFLIVSAMLAVISWLSLGVVSFPMLAGTLGISTKAAATVVNLISAYSTVTAVISIVGAITGVGSIGSGIAATVLYILKKKGAAKAALW

>Streptococcus-pneumoniae_CTD85968.1

MKSKRMEFHNKFLIVSAMLAVISWLSLGVVSFPMLAGTLGISTKAAATVVNLISAYSTVTAVISIVGAITGVGSIGSGIAATVLYILKKKGAAKAALW

>Streptococcus-pneumoniae_CTL81031.1

MKSKRMEFHNKFLIVSAMLAVISWLSLGVVSFPMLAGTLGISTKAAATVVNLISAYSTVTAVISIVGAITGVGSIGSGIAATVLYILKKKGAAKAALW

>Streptococcus-pneumoniae_CTI89760.1

MKSKRMEFHNKFLIVSAMLAVISWLSLGVVSFPMLAGTLGISTKAAATVVNLISAYSTVTAVISIVGAITGVGSIGSGIAATVLYILKKKGAAKAALW

>Streptococcus-pneumoniae_CTN40156.1

MKSKRMEFHNKFLIVSAMLAVISWLSLGVVSFPMLAGTLGISTKAAATVVNLISAYSTVTAVISIVGAITGVGSIGSGIAATVLYILKKKGAAKAALW

>Streptococcus-pneumoniae_CTH44700.1

MKSKRMEFHNKFLIVSAMLAVISWLSLGVVSFPMLAGTLGISTKAAATVVNLISAYSTVTAVISIVGAITGVGSIGSGIAATVLYILKKKGAAKAALW

>Streptococcus-pneumoniae_CTH56295.1

MKSKRMEFHNKFLIVSAMLAVISWLSLGVVSFPMLAGTLGISTKAAATVVNLISAYSTVTAVISIVGAITGVGSIGSGIAATVLYILKKKGAAKAALW

>Streptococcus-pneumoniae_CTL16124.1

MKSKRMEFHNKFLIVSAMLAVISWLSLGVVSFPMLAGTLGISTKAAATVVNLISAYSTVTAVISIVGAITGVGSIGSGIAATVLYILKKKGAAKAALW

>Streptococcus-pneumoniae_CTK90568.1

MKSKRMEFHNKFLIVSAMLAVISWLSLGVVSFPMLAGTLGISTKAAATVVNLISAYSTVTAVISIVGAITGVGSIGSGIAATVLYILKKKGAAKAALW

>Streptococcus-pneumoniae_CTG45121.1

MKSKRMEFHNKFLIVSAMLAVISWLSLGVVSFPMLAGTLGISTKAAATVVNLISAYSTVTAVISIVGAITGVGSIGSGIAATVLYILKKKGAAKAALW

>Streptococcus-pneumoniae_CTJ25047.1

MKSKRMEFHNKFLIVSAMLAVISWLSLGVVSFPMLAGTLGISTKAAATVVNLISAYSTVTAVISIVGAITGVGSIGSGIAATVLYILKKKGAAKAALW

>Streptococcus-pneumoniae_CTN56259.1

MKSKRMEFHNKFLIVSAMLAVISWLSLGVVSFPMLAGTLGISTKAAATVVNLISAYSTVTAVISIVGAITGVGSIGSGIAATVLYILKKKGAAKAALW

>Streptococcus-pneumoniae_CTD48319.1

MKSKRMEFHNKFLIVSAMLAVISWLSLGVVSFPMLAGTLGISTKAAATVVNLISAYSTVTAVISIVGAITGVGSIGSGIAATVLYILKKKGAAKAALW

>Streptococcus-pneumoniae_CTJ45385.1

MKSKRMEFHNKFLIVSAMLAVISWLSLGVVSFPMLAGTLGISTKAAATVVNLISAYSTVTAVISIVGAITGVGSIGSGIAATVLYILKKKGAAKAALW

>Streptococcus-pneumoniae_CTP44457.1

MKSKRMEFHNKFLIVSAMLAVISWLSLGVVSFPMLAGTLGISTKAAATVVNLISAYSTVTAVISIVGAITGVGSIGSGIAATVLYILKKKGAAKAALW

>Streptococcus-pneumoniae_CTL01748.1

MKSKRMEFHNKFLIVSAMLAVISWLSLGVVSFPMLAGTLGISTKAAATVVNLISAYSTVTAVISIVGAITGVGSIGSGIAATVLYILKKKGAAKAALW

>Streptococcus-pneumoniae_CTJ34366.1

MKSKRMEFHNKFLIVSAMLAVISWLSLGVVSFPMLAGTLGISTKAAATVVNLISAYSTVTAVISIVGAITGVGSIGSGIAATVLYILKKKGAAKAALW

>Streptococcus-pneumoniae_CTJ75874.1

MKSKRMEFHNKFLIVSAMLAVISWLSLGVVSFPMLAGTLGISTKAAATVVNLISAYSTVTAVISIVGAITGVGSIGSGIAATVLYILKKKGAAKAALW

>Streptococcus-pneumoniae_CTC96573.1

MKSKRMEFHNKFLIVSAMLAVISWLSLGVVSFPMLAGTLGISTKAAATVVNLISAYSTVTAVISIVGAITGVGSIGSGIAATVLYILKKKGAAKAALW

>Streptococcus-pneumoniae_CTI03146.1

MKSKRMEFHNKFLIVSAMLAVISWLSLGVVSFPMLAGTLGISTKAAATVVNLISAYSTVTAVISIVGAITGVGSIGSGIAATVLYILKKKGAAKAALW

>Streptococcus-pneumoniae_CTH80625.1

MKSKRMEFHNKFLIVSAMLAVISWLSLGVVSFPMLAGTLGISTKAAATVVNLISAYSTVTAVISIVGAITGVGSIGSGIAATVLYILKKKGAAKAALW

>Streptococcus-pneumoniae_CTH71264.1

MKSKRMEFHNKFLIVSAMLAVISWLSLGVVSFPMLAGTLGISTKAAATVVNLISAYSTVTAVISIVGAITGVGSIGSGIAATVLYILKKKGAAKAALW

>Streptococcus-pneumoniae_CTI39262.1

MKSKRMEFHNKFLIVSAMLAVISWLSLGVVSFPMLAGTLGISTKAAATVVNLISAYSTVTAVISIVGAITGVGSIGSGIAATVLYILKKKGAAKAALW

>Streptococcus-pneumoniae_CTK44211.1

MKSKRMEFHNKFLIVSAMLAVISWLSLGVVSFPMLAGTLGISTKAAATVVNLISAYSTVTAVISIVGAITGVGSIGSGIAATVLYILKKKGAAKAALW

>Streptococcus-pneumoniae_CTH87307.1

MKSKRMEFHNKFLIVSAMLAVISWLSLGVVSFPMLAGTLGISTKAAATVVNLISAYSTVTAVISIVGAITGVGSIGSGIAATVLYILKKKGAAKAALW

>Streptococcus-pneumoniae_CTI85883.1

MKSKRMEFHNKFLIVSAMLAVISWLSLGVVSFPMLAGTLGISTKAAATVVNLISAYSTVTAVISIVGAITGVGSIGSGIAATVLYILKKKGAAKAALW

>Streptococcus-pneumoniae_CTP21549.1

MKSKRMEFHNKFLIVSAMLAVISWLSLGVVSFPMLAGTLGISTKAAATVVNLISAYSTVTAVISIVGAITGVGSIGSGIAATVLYILKKKGAAKAALW

>Streptococcus-pneumoniae_CTK49245.1

MKSKRMEFHNKFLIVSAMLAVISWLSLGVVSFPMLAGTLGISTKAAATVVNLISAYSTVTAVISIVGAITGVGSIGSGIAATVLYILKKKGAAKAALW

>Streptococcus-pneumoniae_CTK72355.1

MKSKRMEFHNKFLIVSAMLAVISWLSLGVVSFPMLAGTLGISTKAAATVVNLISAYSTVTAVISIVGAITGVGSIGSGIAATVLYILKKKGAAKAALW

>Streptococcus-pneumoniae_CTD48226.1

MKSKRMEFHNKFLIVSAMLAVISWLSLGVVSFPMLAGTLGISTKAAATVVNLISAYSTVTAVISIVGAITGVGSIGSGIAATVLYILKKKGAAKAALW

>Streptococcus-pneumoniae_CTH92828.1

MKSKRMEFHNKFLIVSAMLAVISWLSLGVVSFPMLAGTLGISTKAAATVVNLISAYSTVTAVISIVGAITGVGSIGSGIAATVLYILKKKGAAKAALW

>Streptococcus-pneumoniae_CTO00491.1

MKSKRMEFHNKFLIVSAMLAVISWLSLGVVSFPMLAGTLGISTKAAATVVNLISAYSTVTAVISIVGAITGVGSIGSGIAATVLYILKKKGAAKAALW

>Streptococcus-pneumoniae_CTD33692.1

MKSKRMEFHNKFLIVSAMLAVISWLSLGVVSFPMLAGTLGISTKAAATVVNLISAYSTVTAVISIVGAITGVGSIGSGIAATVLYILKKKGAAKAALW

>Streptococcus-pneumoniae_CTK76368.1

MKSKRMEFHNKFLIVSAMLAVISWLSLGVVSFPMLAGTLGISTKAAATVVNLISAYSTVTAVISIVGAITGVGSIGSGIAATVLYILKKKGAAKAALW

>Streptococcus-pneumoniae_CTN77828.1

MKSKRMEFHNKFLIVSAMLAVISWLSLGVVSFPMLAGTLGISTKAAATVVNLISAYSTVTAVISIVGAITGVGSIGSGIAATVLYILKKKGAAKAALW

>Streptococcus-pneumoniae_CTK53476.1

MKSKRMEFHNKFLIVSAMLAVISWLSLGVVSFPMLAGTLGISTKAAATVVNLISAYSTVTAVISIVGAITGVGSIGSGIAATVLYILKKKGAAKAALW

>Streptococcus-pneumoniae_CTK47200.1

MKSKRMEFHNKFLIVSAMLAVISWLSLGVVSFPMLAGTLGISTKAAATVVNLISAYSTVTAVISIVGAITGVGSIGSGIAATVLYILKKKGAAKAALW

>Streptococcus-pneumoniae_CTI26073.1

MKSKRMEFHNKFLIVSAMLAVISWLSLGVVSFPMLAGTLGISTKAAATVVNLISAYSTVTAVISIVGAITGVGSIGSGIAATVLYILKKKGAAKAALW

>Streptococcus-pneumoniae_CTH87542.1

MKSKRMEFHNKFLIVSAMLAVISWLSLGVVSFPMLAGTLGISTKAAATVVNLISAYSTVTAVISIVGAITGVGSIGSGIAATVLYILKKKGAAKAALW

>Streptococcus-pneumoniae_CTH73239.1

MKSKRMEFHNKFLIVSAMLAVISWLSLGVVSFPMLAGTLGISTKAAATVVNLISAYSTVTAVISIVGAITGVGSIGSGIAATVLYILKKKGAAKAALW

>Streptococcus-pneumoniae_CTK69609.1

MKSKRMEFHNKFLIVSAMLAVISWLSLGVVSFPMLAGTLGISTKAAATVVNLISAYSTVTAVISIVGAITGVGSIGSGIAATVLYILKKKGAAKAALW

>Streptococcus-pneumoniae_CTJ08904.1

MKSKRMEFHNKFLIVSAMLAVISWLSLGVVSFPMLAGTLGISTKAAATVVNLISAYSTVTAVISIVGAITGVGSIGSGIAATVLYILKKKGAAKAALW

>Streptococcus-pneumoniae_CTJ32544.1

MKSKRMEFHNKFLIVSAMLAVISWLSLGVVSFPMLAGTLGISTKAAATVVNLISAYSTVTAVISIVGAITGVGSIGSGIAATVLYILKKKGAAKAALW

>Streptococcus-pneumoniae_CTN81330.1

MKSKRMEFHNKFLIVSAMLAVISWLSLGVVSFPMLAGTLGISTKAAATVVNLISAYSTVTAVISIVGAITGVGSIGSGIAATVLYILKKKGAAKAALW

>Streptococcus-pneumoniae_CTI92503.1

MKSKRMEFHNKFLIVSAMLAVISWLSLGVVSFPMLAGTLGISTKAAATVVNLISAYSTVTAVISIVGAITGVGSIGSGIAATVLYILKKKGAAKAALW

>Streptococcus-pneumoniae_CTJ01868.1

MKSKRMEFHNKFLIVSAMLAVISWLSLGVVSFPMLAGTLGISTKAAATVVNLISAYSTVTAVISIVGAITGVGSIGSGIAATVLYILKKKGAAKAALW

>Streptococcus-pneumoniae_CTJ08590.1

MKSKRMEFHNKFLIVSAMLAVISWLSLGVVSFPMLAGTLGISTKAAATVVNLISAYSTVTAVISIVGAITGVGSIGSGIAATVLYILKKKGAAKAALW

>Streptococcus-pneumoniae_CTD28338.1

MKSKRMEFHNKFLIVSAMLAVISWLSLGVVSFPMLAGTLGISTKAAATVVNLISAYSTVTAVISIVGAITGVGSIGSGIAATVLYILKKKGAAKAALW

>Streptococcus-pneumoniae_CTN91543.1

MKSKRMEFHNKFLIVSAMLAVISWLSLGVVSFPMLAGTLGISTKAAATVVNLISAYSTVTAVISIVGAITGVGSIGSGIAATVLYILKKKGAAKAALW

>Streptococcus-pneumoniae_CTK64109.1

MKSKRMEFHNKFLIVSAMLAVISWLSLGVVSFPMLAGTLGISTKAAATVVNLISAYSTVTAVISIVGAITGVGSIGSGIAATVLYILKKKGAAKAALW

>Streptococcus-pneumoniae_CTD53994.1

MKSKRMEFHNKFLIVSAMLAVISWLSLGVVSFPMLAGTLGISTKAAATVVNLISAYSTVTAVISIVGAITGVGSIGSGIAATVLYILKKKGAAKAALW

>Streptococcus-pneumoniae_CTO17602.1

MKSKRMEFHNKFLIVSAMLAVISWLSLGVVSFPMLAGTLGISTKAAATVVNLISAYSTVTAVISIVGAITGVGSIGSGIAATVLYILKKKGAAKAALW

>Streptococcus-pneumoniae_CTH52030.1

MKSKRMEFHNKFLIVSAMLAVISWLSLGVVSFPMLAGTLGISTKAAATVVNLISAYSTVTAVISIVGAITGVGSIGSGIAATVLYILKKKGAAKAALW

>Streptococcus-pneumoniae_CTG88202.1

MKSKRMEFHNKFLIVSAMLAVISWLSLGVVSFPMLAGTLGISTKAAATVVNLISAYSTVTAVISIVGAITGVGSIGSGIAATVLYILKKKGAAKAALW

>Streptococcus-pneumoniae_CTI77799.1

MKSKRMEFHNKFLIVSAMLAVISWLSLGVVSFPMLAGTLGISTKAAATVVNLISAYSTVTAVISIVGAITGVGSIGSGIAATVLYILKKKGAAKAALW

>Streptococcus-pneumoniae_CTK66111.1

MKSKRMEFHNKFLIVSAMLAVISWLSLGVVSFPMLAGTLGISTKAAATVVNLISAYSTVTAVISIVGAITGVGSIGSGIAATVLYILKKKGAAKAALW

>Streptococcus-pneumoniae_CTN47321.1

MKSKRMEFHNKFLIVSAMLAVISWLSLGVVSFPMLAGTLGISTKAAATVVNLISAYSTVTAVISIVGAITGVGSIGSGIAATVLYILKKKGAAKAALW

>Streptococcus-pneumoniae_CTN86338.1

MKSKRMEFHNKFLIVSAMLAVISWLSLGVVSFPMLAGTLGISTKAAATVVNLISAYSTVTAVISIVGAITGVGSIGSGIAATVLYILKKKGAAKAALW

>Streptococcus-pneumoniae_CTP28659.1

MKSKRMEFHNKFLIVSAMLAVISWLSLGVVSFPMLAGTLGISTKAAATVVNLISAYSTVTAVISIVGAITGVGSIGSGIAATVLYILKKKGAAKAALW

>Streptococcus-pneumoniae_CTH50992.1

MKSKRMEFHNKFLIVSAMLAVISWLSLGVVSFPMLAGTLGISTKAAATVVNLISAYSTVTAVISIVGAITGVGSIGSGIAATVLYILKKKGAAKAALW

>Streptococcus-pneumoniae_CTP69358.1

MKSKRMEFHNKFLIVSAMLAVISWLSLGVVSFPMLAGTLGISTKAAATVVNLISAYSTVTAVISIVGAITGVGSIGSGIAATVLYILKKKGAAKAALW

>Streptococcus-pneumoniae_CTC96541.1

MKSKRMEFHNKFLIVSAMLAVISWLSLGVVSFPMLAGTLGISTKAAATVVNLISAYSTVTAVISIVGAITGVGSIGSGIAATVLYILKKKGAAKAALW

>Streptococcus-pneumoniae_CTJ71723.1

MKSKRMEFHNKFLIVSAMLAVISWLSLGVVSFPMLAGTLGISTKAAATVVNLISAYSTVTAVISIVGAITGVGSIGSGIAATVLYILKKKGAAKAALW

>Streptococcus-pneumoniae_CTI55178.1

MKSKRMEFHNKFLIVSAMLAVISWLSLGVVSFPMLAGTLGISTKAAATVVNLISAYSTVTAVISIVGAITGVGSIGSGIAATVLYILKKKGAAKAALW

>Streptococcus-pneumoniae_CTJ12666.1

MKSKRMEFHNKFLIVSAMLAVISWLSLGVVSFPMLAGTLGISTKAAATVVNLISAYSTVTAVISIVGAITGVGSIGSGIAATVLYILKKKGAAKAALW

>Streptococcus-pneumoniae_CTO97205.1

MKSKRMEFHNKFLIVSAMLAVISWLSLGVVSFPMLAGTLGISTKAAATVVNLISAYSTVTAVISIVGAITGVGSIGSGIAATVLYILKKKGAAKAALW

>Streptococcus-pneumoniae_CTN46102.1

MKSKRMEFHNKFLIVSAMLAVISWLSLGVVSFPMLAGTLGISTKAAATVVNLISAYSTVTAVISIVGAITGVGSIGSGIAATVLYILKKKGAAKAALW

>Streptococcus-pneumoniae_CTI52078.1

MKSKRMEFHNKFLIVSAMLAVISWLSLGVVSFPMLAGTLGISTKAAATVVNLISAYSTVTAVISIVGAITGVGSIGSGIAATVLYILKKKGAAKAALW

>Streptococcus-pneumoniae_CTJ66601.1

MKSKRMEFHNKFLIVSAMLAVISWLSLGVVSFPMLAGTLGISTKAAATVVNLISAYSTVTAVISIVGAITGVGSIGSGIAATVLYILKKKGAAKAALW

>Streptococcus-pneumoniae_CTH68840.1

MKSKRMEFHNKFLIVSAMLAVISWLSLGVVSFPMLAGTLGISTKAAATVVNLISAYSTVTAVISIVGAITGVGSIGSGIAATVLYILKKKGAAKAALW

>Streptococcus-pneumoniae_CTH66929.1

MKSKRMEFHNKFLIVSAMLAVISWLSLGVVSFPMLAGTLGISTKAAATVVNLISAYSTVTAVISIVGAITGVGSIGSGIAATVLYILKKKGAAKAALW

>Streptococcus-pneumoniae_CTJ45861.1

MKSKRMEFHNKFLIVSAMLAVISWLSLGVVSFPMLAGTLGISTKAAATVVNLISAYSTVTAVISIVGAITGVGSIGSGIAATVLYILKKKGAAKAALW

>Streptococcus-pneumoniae_CTG95883.1

MKSKRMEFHNKFLIVSAMLAVISWLSLGVVSFPMLAGTLGISTKAAATVVNLISAYSTVTAVISIVGAITGVGSIGSGIAATVLYILKKKGAAKAALW

>Streptococcus-pneumoniae_CTJ16698.1

MKSKRMEFHNKFLIVSAMLAVISWLSLGVVSFPMLAGTLGISTKAAATVVNLISAYSTVTAVISIVGAITGVGSIGSGIAATVLYILKKKGAAKAALW

>Streptococcus-pneumoniae_CTH42200.1

MKSKRMEFHNKFLIVSAMLAVISWLSLGVVSFPMLAGTLGISTKAAATVVNLISAYSTVTAVISIVGAITGVGSIGSGIAATVLYILKKKGAAKAALW

>Streptococcus-pneumoniae_CTD15525.1

MKSKRMEFHNKFLIVSAMLAVISWLSLGVVSFPMLAGTLGISTKAAATVVNLISAYSTVTAVISIVGAITGVGSIGSGIAATVLYILKKKGAAKAALW

>Streptococcus-pneumoniae_CTD58919.1

MKSKRMEFHNKFLIVSAMLAVISWLSLGVVSFPMLAGTLGISTKAAATVVNLISAYSTVTAVISIVGAITGVGSIGSGIAATVLYILKKKGAAKAALW

>Streptococcus-pneumoniae_CTH96099.1

MKSKRMEFHNKFLIVSAMLAVISWLSLGVVSFPMLAGTLGISTKAAATVVNLISAYSTVTAVISIVGAITGVGSIGSGIAATVLYILKKKGAAKAALW

>Streptococcus-pneumoniae_CTK25620.1

MKSKRMEFHNKFLIVSAMLAVISWLSLGVVSFPMLAGTLGISTKAAATVVNLISAYSTVTAVISIVGAITGVGSIGSGIAATVLYILKKKGAAKAALW

>Streptococcus-pneumoniae_CTJ51872.1

MKSKRMEFHNKFLIVSAMLAVISWLSLGVVSFPMLAGTLGISTKAAATVVNLISAYSTVTAVISIVGAITGVGSIGSGIAATVLYILKKKGAAKAALW

>Streptococcus-pneumoniae_CTP32229.1

MKSKRMEFHNKFLIVSAMLAVISWLSLGVVSFPMLAGTLGISTKAAATVVNLISAYSTVTAVISIVGAITGVGSIGSGIAATVLYILKKKGAAKAALW

>Streptococcus-pneumoniae_CTG31560.1

MKSKRMEFHNKFLIVSAMLAVISWLSLGVVSFPMLAGTLGISTKAAATVVNLISAYSTVTAVISIVGAITGVGSIGSGIAATVLYILKKKGAAKAALW

>Streptococcus-pneumoniae_CTK51422.1

MKSKRMEFHNKFLIVSAMLAVISWLSLGVVSFPMLAGTLGISTKAAATVVNLISAYSTVTAVISIVGAITGVGSIGSGIAATVLYILKKKGAAKAALW

>Streptococcus-pneumoniae_CTI58449.1

MKSKRMEFHNKFLIVSAMLAVISWLSLGVVSFPMLAGTLGISTKAAATVVNLISAYSTVTAVISIVGAITGVGSIGSGIAATVLYILKKKGAAKAALW

>Streptococcus-pneumoniae_CTC95683.1

MKSKRMEFHNKFLIVSAMLAVISWLSLGVVSFPMLAGTLGISTKAAATVVNLISAYSTVTAVISIVGAITGVGSIGSGIAATVLYILKKKGAAKAALW

>Streptococcus-pneumoniae_CTI59873.1

MKSKRMEFHNKFLIVSAMLAVISWLSLGVVSFPMLAGTLGISTKAAATVVNLISAYSTVTAVISIVGAITGVGSIGSGIAATVLYILKKKGAAKAALW

>Streptococcus-pneumoniae_CTH48543.1

MKSKRMEFHNKFLIVSAMLAVISWLSLGVVSFPMLAGTLGISTKAAATVVNLISAYSTVTAVISIVGAITGVGSIGSGIAATVLYILKKKGAAKAALW

>Streptococcus-pneumoniae_CTD29163.1

MKSKRMEFHNKFLIVSAMLAVISWLSLGVVSFPMLAGTLGISTKAAATVVNLISAYSTVTAVISIVGAITGVGSIGSGIAATVLYILKKKGAAKAALW

>Streptococcus-pneumoniae_CTI83676.1

MKSKRMEFHNKFLIVSAMLAVISWLSLGVVSFPMLAGTLGISTKAAATVVNLISAYSTVTAVISIVGAITGVGSIGSGIAATVLYILKKKGAAKAALW

>Streptococcus-pneumoniae_CTJ07965.1

MKSKRMEFHNKFLIVSAMLAVISWLSLGVVSFPMLAGTLGISTKAAATVVNLISAYSTVTAVISIVGAITGVGSIGSGIAATVLYILKKKGAAKAALW

>Streptococcus-pneumoniae_CTI79564.1

MKSKRMEFHNKFLIVSAMLAVISWLSLGVVSFPMLAGTLGISTKAAATVVNLISAYSTVTAVISIVGAITGVGSIGSGIAATVLYILKKKGAAKAALW

>Streptococcus-pneumoniae_CTJ45561.1

MKSKRMEFHNKFLIVSAMLAVISWLSLGVVSFPMLAGTLGISTKAAATVVNLISAYSTVTAVISIVGAITGVGSIGSGIAATVLYILKKKGAAKAALW

>Streptococcus-pneumoniae_CTJ48085.1

MKSKRMEFHNKFLIVSAMLAVISWLSLGVVSFPMLAGTLGISTKAAATVVNLISAYSTVTAVISIVGAITGVGSIGSGIAATVLYILKKKGAAKAALW

>Streptococcus-pneumoniae_CTI03322.1

MKSKRMEFHNKFLIVSAMLAVISWLSLGVVSFPMLAGTLGISTKAAATVVNLISAYSTVTAVISIVGAITGVGSIGSGIAATVLYILKKKGAAKAALW

>Streptococcus-pneumoniae_CTI67563.1

MKSKRMEFHNKFLIVSAMLAVISWLSLGVVSFPMLAGTLGISTKAAATVVNLISAYSTVTAVISIVGAITGVGSIGSGIAATVLYILKKKGAAKAALW

>Streptococcus-pneumoniae_CTI62106.1

MKSKRMEFHNKFLIVSAMLAVISWLSLGVVSFPMLAGTLGISTKAAATVVNLISAYSTVTAVISIVGAITGVGSIGSGIAATVLYILKKKGAAKAALW

>Streptococcus-pneumoniae_CTK01813.1

MKSKRMEFHNKFLIVSAMLAVISWLSLGVVSFPMLAGTLGISTKAAATVVNLISAYSTVTAVISIVGAITGVGSIGSGIAATVLYILKKKGAAKAALW

>Streptococcus-pneumoniae_CTK00520.1

MKSKRMEFHNKFLIVSAMLAVISWLSLGVVSFPMLAGTLGISTKAAATVVNLISAYSTVTAVISIVGAITGVGSIGSGIAATVLYILKKKGAAKAALW

>Streptococcus-pneumoniae_CTC66069.1

MKSKRMEFHNKFLIVSAMLAVISWLSLGVVSFPMLAGTLGISTKAAATVVNLISAYSTVTAVISIVGAITGVGSIGSGIAATVLYILKKKGAAKAALW

>Streptococcus-pneumoniae_CTI61753.1

MKSKRMEFHNKFLIVSAMLAVISWLSLGVVSFPMLAGTLGISTKAAATVVNLISAYSTVTAVISIVGAITGVGSIGSGIAATVLYILKKKGAAKAALW

>Streptococcus-pneumoniae_CTK11595.1

MKSKRMEFHNKFLIVSAMLAVISWLSLGVVSFPMLAGTLGISTKAAATVVNLISAYSTVTAVISIVGAITGVGSIGSGIAATVLYILKKKGAAKAALW

>Streptococcus-pneumoniae_CTP52290.1

MKSKRMEFHNKFLIVSAMLAVISWLSLGVVSFPMLAGTLGISTKAAATVVNLISAYSTVTAVISIVGAITGVGSIGSGIAATVLYILKKKGAAKAALW

>Streptococcus-pneumoniae_CTC72594.1

MKSKRMEFHNKFLIVSAMLAVISWLSLGVVSFPMLAGTLGISTKAAATVVNLISAYSTVTAVISIVGAITGVGSIGSGIAATVLYILKKKGAAKAALW

>Streptococcus-pneumoniae_CTH62022.1

MKSKRMEFHNKFLIVSAMLAVISWLSLGVVSFPMLAGTLGISTKAAATVVNLISAYSTVTAVISIVGAITGVGSIGSGIAATVLYILKKKGAAKAALW

>Streptococcus-pneumoniae_CTK64225.1

MKSKRMEFHNKFLIVSAMLAVISWLSLGVVSFPMLAGTLGISTKAAATVVNLISAYSTVTAVISIVGAITGVGSIGSGIAATVLYILKKKGAAKAALW

>Streptococcus-pneumoniae_CTJ93764.1

MKSKRMEFHNKFLIVSAMLAVISWLSLGVVSFPMLAGTLGISTKAAATVVNLISAYSTVTAVISIVGAITGVGSIGSGIAATVLYILKKKGAAKAALW

>Streptococcus-pneumoniae_CTH75697.1

MKSKRMEFHNKFLIVSAMLAVISWLSLGVVSFPMLAGTLGISTKAAATVVNLISAYSTVTAVISIVGAITGVGSIGSGIAATVLYILKKKGAAKAALW

>Streptococcus-pneumoniae_CTN97253.1

MKSKRMEFHNKFLIVSAMLAVISWLSLGVVSFPMLAGTLGISTKAAATVVNLISAYSTVTAVISIVGAITGVGSIGSGIAATVLYILKKKGAAKAALW

>Streptococcus-pneumoniae_CTG43673.1

MKSKRMEFHNKFLIVSAMLAVISWLSLGVVSFPMLAGTLGISTKAAATVVNLISAYSTVTAVISIVGAITGVGSIGSGIAATVLYILKKKGAAKAALW

>Streptococcus-pneumoniae_CTI75048.1

MKSKRMEFHNKFLIVSAMLAVISWLSLGVVSFPMLAGTLGISTKAAATVVNLISAYSTVTAVISIVGAITGVGSIGSGIAATVLYILKKKGAAKAALW

>Streptococcus-pneumoniae_CTN73763.1

MKSKRMEFHNKFLIVSAMLAVISWLSLGVVSFPMLAGTLGISTKAAATVVNLISAYSTVTAVISIVGAITGVGSIGSGIAATVLYILKKKGAAKAALW

>Streptococcus-pneumoniae_CTH88005.1

MKSKRMEFHNKFLIVSAMLAVISWLSLGVVSFPMLAGTLGISTKAAATVVNLISAYSTVTAVISIVGAITGVGSIGSGIAATVLYILKKKGAAKAALW

>Streptococcus-pneumoniae_CTP21763.1

MKSKRMEFHNKFLIVSAMLAVISWLSLGVVSFPMLAGTLGISTKAAATVVNLISAYSTVTAVISIVGAITGVGSIGSGIAATVLYILKKKGAAKAALW

>Streptococcus-pneumoniae_CTK24151.1

MKSKRMEFHNKFLIVSAMLAVISWLSLGVVSFPMLAGTLGISTKAAATVVNLISAYSTVTAVISIVGAITGVGSIGSGIAATVLYILKKKGAAKAALW

>Streptococcus-pneumoniae_CTP51074.1

MKSKRMEFHNKFLIVSAMLAVISWLSLGVVSFPMLAGTLGISTKAAATVVNLISAYSTVTAVISIVGAITGVGSIGSGIAATVLYILKKKGAAKAALW

>Streptococcus-pneumoniae_CTG25440.1

MKSKRMEFHNKFLIVSAMLAVISWLSLGVVSFPMLAGTLGISTKAAATVVNLISAYSTVTAVISIVGAITGVGSIGSGIAATVLYILKKKGAAKAALW

>Streptococcus-pneumoniae_CTC95537.1

MKSKRMEFHNKFLIVSAMLAVISWLSLGVVSFPMLAGTLGISTKAAATVVNLISAYSTVTAVISIVGAITGVGSIGSGIAATVLYILKKKGAAKAALW

>Streptococcus-pneumoniae_CTJ03509.1

MKSKRMEFHNKFLIVSAMLAVISWLSLGVVSFPMLAGTLGISTKAAATVVNLISAYSTVTAVISIVGAITGVGSIGSGIAATVLYILKKKGAAKAALW

>Streptococcus-pneumoniae_CTK56237.1

MKSKRMEFHNKFLIVSAMLAVISWLSLGVVSFPMLAGTLGISTKAAATVVNLISAYSTVTAVISIVGAITGVGSIGSGIAATVLYILKKKGAAKAALW

>Streptococcus-pneumoniae_CTJ31031.1

MKSKRMEFHNKFLIVSAMLAVISWLSLGVVSFPMLAGTLGISTKAAATVVNLISAYSTVTAVISIVGAITGVGSIGSGIAATVLYILKKKGAAKAALW

>Streptococcus-pneumoniae_CTH10887.1

MKSKRMEFHNKFLIVSAMLAVISWLSLGVVSFPMLAGTLGISTKAAATVVNLISAYSTVTAVISIVGAITGVGSIGSGIAATVLYILKKKGAAKAALW

>Streptococcus-pneumoniae_CTH98430.1

MKSKRMEFHNKFLIVSAMLAVISWLSLGVVSFPMLAGTLGISTKAAATVVNLISAYSTVTAVISIVGAITGVGSIGSGIAATVLYILKKKGAAKAALW

>Streptococcus-pneumoniae_CKH84237.1

MKSKRMEFHNKFLIVSAMLAVISWLSLGVVSFPMLAGTLGISTKAAATVVNLISAYSTVTAVISIVGAITGVGSIGSGIAATVLYILKKKGAAKAALW

>Streptococcus-pneumoniae_CKJ10604.1

MKSKRMEFHNKFLIVSAMLAVISWLSLGVVSFPMLAGTLGISTKAAATVVNLISAYSTVTAVISIVGAITGVGSIGSGIAATVLYILKKKGAAKAALW

>Streptococcus-pneumoniae_CTD41302.1

MKSKRMEFHNKFLIVSAMLAVISWLSLGVVSFPMLAGTLGISTKAAATVVNLISAYSTVTAVISIVGAITGVGSIGSGIAATVLYILKKKGAAKAALW

>Streptococcus-pneumoniae_CTD58411.1

MKSKRMEFHNKFLIVSAMLAVISWLSLGVVSFPMLAGTLGISTKAAATVVNLISAYSTVTAVISIVGAITGVGSIGSGIAATVLYILKKKGAAKAALW

>Streptococcus-pseudopneumoniae_KPL38874.1

MKSKRMEFHNKFLIVSAMLAVISWLSLGVVSFPMLAGTLGISTKAAATVVNLISAYSTVTAVISIVGAITGVGSIGSGIAATVLYILKKKGAAKAALW

>Streptococcus-pseudopneumoniae_KPL41441.1

MKSKRMEFHNKFLIVSAMLAVISWLSLGVVSFPMLAGTLGISTKAAATVVNLISAYSTVTAVISIVGAITGVGSIGSGIAATVLYILKKKGAAKAALW

>Streptococcus-pseudopneumoniae_KPL43822.1

MKSKRMEFHNKFLIVSAMLAVISWLSLGVVSFPMLAGTLGISTKAAATVVNLISAYSTVTAVISIVGAITGVGSIGSGIAATVLYILKKKGAAKAALW

>Streptococcus-pneumoniae_CVU23528.1

MKSKRMEFHNKFLIVSAMLAVISWLSLGVVSFPMLAGTLGISTKAAATVVNLISAYSTVTAVISIVGAITGVGSIGSGIAATVLYILKKKGAAKAALW

>Streptococcus-pneumoniae_CVK46719.1

MKSKRMEFHNKFLIVSAMLAVISWLSLGVVSFPMLAGTLGISTKAAATVVNLISAYSTVTAVISIVGAITGVGSIGSGIAATVLYILKKKGAAKAALW

>Streptococcus-pneumoniae_CVT39294.1

MKSKRMEFHNKFLIVSAMLAVISWLSLGVVSFPMLAGTLGISTKAAATVVNLISAYSTVTAVISIVGAITGVGSIGSGIAATVLYILKKKGAAKAALW

>Streptococcus-pneumoniae_CVV95696.1

MKSKRMEFHNKFLIVSAMLAVISWLSLGVVSFPMLAGTLGISTKAAATVVNLISAYSTVTAVISIVGAITGVGSIGSGIAATVLYILKKKGAAKAALW

>Streptococcus-pneumoniae_CVV71336.1

MKSKRMEFHNKFLIVSAMLAVISWLSLGVVSFPMLAGTLGISTKAAATVVNLISAYSTVTAVISIVGAITGVGSIGSGIAATVLYILKKKGAAKAALW

>Streptococcus-pneumoniae_CVQ38429.1

MKSKRMEFHNKFLIVSAMLAVISWLSLGVVSFPMLAGTLGISTKAAATVVNLISAYSTVTAVISIVGAITGVGSIGSGIAATVLYILKKKGAAKAALW

>Streptococcus-pneumoniae_CVU21585.1

MKSKRMEFHNKFLIVSAMLAVISWLSLGVVSFPMLAGTLGISTKAAATVVNLISAYSTVTAVISIVGAITGVGSIGSGIAATVLYILKKKGAAKAALW

>Streptococcus-pneumoniae_CVT71365.1

MKSKRMEFHNKFLIVSAMLAVISWLSLGVVSFPMLAGTLGISTKAAATVVNLISAYSTVTAVISIVGAITGVGSIGSGIAATVLYILKKKGAAKAALW

>Streptococcus-pneumoniae_CVT53999.1

MKSKRMEFHNKFLIVSAMLAVISWLSLGVVSFPMLAGTLGISTKAAATVVNLISAYSTVTAVISIVGAITGVGSIGSGIAATVLYILKKKGAAKAALW

>Streptococcus-pneumoniae_CVL90205.1

MKSKRMEFHNKFLIVSAMLAVISWLSLGVVSFPMLAGTLGISTKAAATVVNLISAYSTVTAVISIVGAITGVGSIGSGIAATVLYILKKKGAAKAALW

>Streptococcus-pneumoniae_CVV94576.1

MKSKRMEFHNKFLIVSAMLAVISWLSLGVVSFPMLAGTLGISTKAAATVVNLISAYSTVTAVISIVGAITGVGSIGSGIAATVLYILKKKGAAKAALW

>Streptococcus-pneumoniae_CVT20608.1

MKSKRMEFHNKFLIVSAMLAVISWLSLGVVSFPMLAGTLGISTKAAATVVNLISAYSTVTAVISIVGAITGVGSIGSGIAATVLYILKKKGAAKAALW

>Streptococcus-pneumoniae_CVV32816.1

MKSKRMEFHNKFLIVSAMLAVISWLSLGVVSFPMLAGTLGISTKAAATVVNLISAYSTVTAVISIVGAITGVGSIGSGIAATVLYILKKKGAAKAALW

>Streptococcus-pneumoniae_CVP28154.1

MKSKRMEFHNKFLIVSAMLAVISWLSLGVVSFPMLAGTLGISTKAAATVVNLISAYSTVTAVISIVGAITGVGSIGSGIAATVLYILKKKGAAKAALW

>Streptococcus-pneumoniae_CVN02217.1

MKSKRMEFHNKFLIVSAMLAVISWLSLGVVSFPMLAGTLGISTKAAATVVNLISAYSTVTAVISIVGAITGVGSIGSGIAATVLYILKKKGAAKAALW

>Streptococcus-pneumoniae_CVN08625.1

MKSKRMEFHNKFLIVSAMLAVISWLSLGVVSFPMLAGTLGISTKAAATVVNLISAYSTVTAVISIVGAITGVGSIGSGIAATVLYILKKKGAAKAALW

>Streptococcus-pneumoniae_CVN90580.1

MKSKRMEFHNKFLIVSAMLAVISWLSLGVVSFPMLAGTLGISTKAAATVVNLISAYSTVTAVISIVGAITGVGSIGSGIAATVLYILKKKGAAKAALW

>Streptococcus-pneumoniae_CVT66571.1

MKSKRMEFHNKFLIVSAMLAVISWLSLGVVSFPMLAGTLGISTKAAATVVNLISAYSTVTAVISIVGAITGVGSIGSGIAATVLYILKKKGAAKAALW

>Streptococcus-pneumoniae_CVL33169.1

MKSKRMEFHNKFLIVSAMLAVISWLSLGVVSFPMLAGTLGISTKAAATVVNLISAYSTVTAVISIVGAITGVGSIGSGIAATVLYILKKKGAAKAALW

>Streptococcus-pneumoniae_CVL91043.1

MKSKRMEFHNKFLIVSAMLAVISWLSLGVVSFPMLAGTLGISTKAAATVVNLISAYSTVTAVISIVGAITGVGSIGSGIAATVLYILKKKGAAKAALW

>Streptococcus-pneumoniae_CVL77612.1

MKSKRMEFHNKFLIVSAMLAVISWLSLGVVSFPMLAGTLGISTKAAATVVNLISAYSTVTAVISIVGAITGVGSIGSGIAATVLYILKKKGAAKAALW

>Streptococcus-pneumoniae_CVR27988.1

MKSKRMEFHNKFLIVSAMLAVISWLSLGVVSFPMLAGTLGISTKAAATVVNLISAYSTVTAVISIVGAITGVGSIGSGIAATVLYILKKKGAAKAALW

>Streptococcus-pneumoniae_CVW74561.1

MKSKRMEFHNKFLIVSAMLAVISWLSLGVVSFPMLAGTLGISTKAAATVVNLISAYSTVTAVISIVGAITGVGSIGSGIAATVLYILKKKGAAKAALW

>Streptococcus-pneumoniae_CVY18199.1

MKSKRMEFHNKFLIVSAMLAVISWLSLGVVSFPMLAGTLGISTKAAATVVNLISAYSTVTAVISIVGAITGVGSIGSGIAATVLYILKKKGAAKAALW

>Streptococcus-pneumoniae_CVT78940.1

MKSKRMEFHNKFLIVSAMLAVISWLSLGVVSFPMLAGTLGISTKAAATVVNLISAYSTVTAVISIVGAITGVGSIGSGIAATVLYILKKKGAAKAALW

>Streptococcus-pneumoniae_CVQ31128.1

MKSKRMEFHNKFLIVSAMLAVISWLSLGVVSFPMLAGTLGISTKAAATVVNLISAYSTVTAVISIVGAITGVGSIGSGIAATVLYILKKKGAAKAALW

>Streptococcus-pneumoniae_CVX84700.1

MKSKRMEFHNKFLIVSAMLAVISWLSLGVVSFPMLAGTLGISTKAAATVVNLISAYSTVTAVISIVGAITGVGSIGSGIAATVLYILKKKGAAKAALW

>Streptococcus-pneumoniae_CVR12182.1

MKSKRMEFHNKFLIVSAMLAVISWLSLGVVSFPMLAGTLGISTKAAATVVNLISAYSTVTAVISIVGAITGVGSIGSGIAATVLYILKKKGAAKAALW

>Streptococcus-pneumoniae_CVM99897.1

MKSKRMEFHNKFLIVSAMLAVISWLSLGVVSFPMLAGTLGISTKAAATVVNLISAYSTVTAVISIVGAITGVGSIGSGIAATVLYILKKKGAAKAALW

>Streptococcus-pneumoniae_CVR38169.1

MKSKRMEFHNKFLIVSAMLAVISWLSLGVVSFPMLAGTLGISTKAAATVVNLISAYSTVTAVISIVGAITGVGSIGSGIAATVLYILKKKGAAKAALW

>Streptococcus-pneumoniae_CVT24703.1

MKSKRMEFHNKFLIVSAMLAVISWLSLGVVSFPMLAGTLGISTKAAATVVNLISAYSTVTAVISIVGAITGVGSIGSGIAATVLYILKKKGAAKAALW

>Streptococcus-pneumoniae_CVL96045.1

MKSKRMEFHNKFLIVSAMLAVISWLSLGVVSFPMLAGTLGISTKAAATVVNLISAYSTVTAVISIVGAITGVGSIGSGIAATVLYILKKKGAAKAALW

>Streptococcus-pneumoniae_CVO03535.1

MKSKRMEFHNKFLIVSAMLAVISWLSLGVVSFPMLAGTLGISTKAAATVVNLISAYSTVTAVISIVGAITGVGSIGSGIAATVLYILKKKGAAKAALW

>Streptococcus-pneumoniae_CVU63689.1

MKSKRMEFHNKFLIVSAMLAVISWLSLGVVSFPMLAGTLGISTKAAATVVNLISAYSTVTAVISIVGAITGVGSIGSGIAATVLYILKKKGAAKAALW

>Streptococcus-pneumoniae_CVW29170.1

MKSKRMEFHNKFLIVSAMLAVISWLSLGVVSFPMLAGTLGISTKAAATVVNLISAYSTVTAVISIVGAITGVGSIGSGIAATVLYILKKKGAAKAALW

>Streptococcus-pneumoniae_CVV30794.1

MKSKRMEFHNKFLIVSAMLAVISWLSLGVVSFPMLAGTLGISTKAAATVVNLISAYSTVTAVISIVGAITGVGSIGSGIAATVLYILKKKGAAKAALW

>Streptococcus-pneumoniae_CVQ12892.1

MKSKRMEFHNKFLIVSAMLAVISWLSLGVVSFPMLAGTLGISTKAAATVVNLISAYSTVTAVISIVGAITGVGSIGSGIAATVLYILKKKGAAKAALW

>Streptococcus-pneumoniae_CVS50661.1

MKSKRMEFHNKFLIVSAMLAVISWLSLGVVSFPMLAGTLGISTKAAATVVNLISAYSTVTAVISIVGAITGVGSIGSGIAATVLYILKKKGAAKAALW

>Streptococcus-pneumoniae_CVX98853.1

MKSKRMEFHNKFLIVSAMLAVISWLSLGVVSFPMLAGTLGISTKAAATVVNLISAYSTVTAVISIVGAITGVGSIGSGIAATVLYILKKKGAAKAALW

>Streptococcus-pneumoniae_CVQ52927.1

MKSKRMEFHNKFLIVSAMLAVISWLSLGVVSFPMLAGTLGISTKAAATVVNLISAYSTVTAVISIVGAITGVGSIGSGIAATVLYILKKKGAAKAALW

>Streptococcus-pneumoniae_CVQ73696.1

MKSKRMEFHNKFLIVSAMLAVISWLSLGVVSFPMLAGTLGISTKAAATVVNLISAYSTVTAVISIVGAITGVGSIGSGIAATVLYILKKKGAAKAALW

>Streptococcus-pneumoniae_CVP45360.1

MKSKRMEFHNKFLIVSAMLAVISWLSLGVVSFPMLAGTLGISTKAAATVVNLISAYSTVTAVISIVGAITGVGSIGSGIAATVLYILKKKGAAKAALW

>Streptococcus-pneumoniae_CVN37926.1

MKSKRMEFHNKFLIVSAMLAVISWLSLGVVSFPMLAGTLGISTKAAATVVNLISAYSTVTAVISIVGAITGVGSIGSGIAATVLYILKKKGAAKAALW

>Streptococcus-pneumoniae_CVR59704.1

MKSKRMEFHNKFLIVSAMLAVISWLSLGVVSFPMLAGTLGISTKAAATVVNLISAYSTVTAVISIVGAITGVGSIGSGIAATVLYILKKKGAAKAALW

>Streptococcus-pneumoniae_CVM87912.1

MKSKRMEFHNKFLIVSAMLAVISWLSLGVVSFPMLAGTLGISTKAAATVVNLISAYSTVTAVISIVGAITGVGSIGSGIAATVLYILKKKGAAKAALW

>Streptococcus-pneumoniae_CVV22873.1

MKSKRMEFHNKFLIVSAMLAVISWLSLGVVSFPMLAGTLGISTKAAATVVNLISAYSTVTAVISIVGAITGVGSIGSGIAATVLYILKKKGAAKAALW

>Streptococcus-pneumoniae_CVU37758.1

MKSKRMEFHNKFLIVSAMLAVISWLSLGVVSFPMLAGTLGISTKAAATVVNLISAYSTVTAVISIVGAITGVGSIGSGIAATVLYILKKKGAAKAALW

>Streptococcus-pneumoniae_CVU11180.1

MKSKRMEFHNKFLIVSAMLAVISWLSLGVVSFPMLAGTLGISTKAAATVVNLISAYSTVTAVISIVGAITGVGSIGSGIAATVLYILKKKGAAKAALW

>Streptococcus-pneumoniae_CVO87447.1

MKSKRMEFHNKFLIVSAMLAVISWLSLGVVSFPMLAGTLGISTKAAATVVNLISAYSTVTAVISIVGAITGVGSIGSGIAATVLYILKKKGAAKAALW

>Streptococcus-pneumoniae_CVV04383.1

MKSKRMEFHNKFLIVSAMLAVISWLSLGVVSFPMLAGTLGISTKAAATVVNLISAYSTVTAVISIVGAITGVGSIGSGIAATVLYILKKKGAAKAALW

>Streptococcus-pneumoniae_CVT72698.1

MKSKRMEFHNKFLIVSAMLAVISWLSLGVVSFPMLAGTLGISTKAAATVVNLISAYSTVTAVISIVGAITGVGSIGSGIAATVLYILKKKGAAKAALW

>Streptococcus-pneumoniae_CVO78065.1

MKSKRMEFHNKFLIVSAMLAVISWLSLGVVSFPMLAGTLGISTKAAATVVNLISAYSTVTAVISIVGAITGVGSIGSGIAATVLYILKKKGAAKAALW

>Streptococcus-pneumoniae_CVN65381.1

MKSKRMEFHNKFLIVSAMLAVISWLSLGVVSFPMLAGTLGISTKAAATVVNLISAYSTVTAVISIVGAITGVGSIGSGIAATVLYILKKKGAAKAALW

>Streptococcus-pneumoniae_CVV90293.1

MKSKRMEFHNKFLIVSAMLAVISWLSLGVVSFPMLAGTLGISTKAAATVVNLISAYSTVTAVISIVGAITGVGSIGSGIAATVLYILKKKGAAKAALW

>Streptococcus-pneumoniae_CVW14969.1

MKSKRMEFHNKFLIVSAMLAVISWLSLGVVSFPMLAGTLGISTKAAATVVNLISAYSTVTAVISIVGAITGVGSIGSGIAATVLYILKKKGAAKAALW

>Streptococcus-pneumoniae_CVV30494.1

MKSKRMEFHNKFLIVSAMLAVISWLSLGVVSFPMLAGTLGISTKAAATVVNLISAYSTVTAVISIVGAITGVGSIGSGIAATVLYILKKKGAAKAALW

>Streptococcus-pneumoniae_CVP71840.1

MKSKRMEFHNKFLIVSAMLAVISWLSLGVVSFPMLAGTLGISTKAAATVVNLISAYSTVTAVISIVGAITGVGSIGSGIAATVLYILKKKGAAKAALW

>Streptococcus-pneumoniae_CVR46802.1

MKSKRMEFHNKFLIVSAMLAVISWLSLGVVSFPMLAGTLGISTKAAATVVNLISAYSTVTAVISIVGAITGVGSIGSGIAATVLYILKKKGAAKAALW

>Streptococcus-pneumoniae_CVT95844.1

MKSKRMEFHNKFLIVSAMLAVISWLSLGVVSFPMLAGTLGISTKAAATVVNLISAYSTVTAVISIVGAITGVGSIGSGIAATVLYILKKKGAAKAALW

>Streptococcus-pneumoniae_CVR17365.1

MKSKRMEFHNKFLIVSAMLAVISWLSLGVVSFPMLAGTLGISTKAAATVVNLISAYSTVTAVISIVGAITGVGSIGSGIAATVLYILKKKGAAKAALW

>Streptococcus-pneumoniae_CVS37179.1

MKSKRMEFHNKFLIVSAMLAVISWLSLGVVSFPMLAGTLGISTKAAATVVNLISAYSTVTAVISIVGAITGVGSIGSGIAATVLYILKKKGAAKAALW

>Streptococcus-pneumoniae_CVQ05453.1

MKSKRMEFHNKFLIVSAMLAVISWLSLGVVSFPMLAGTLGISTKAAATVVNLISAYSTVTAVISIVGAITGVGSIGSGIAATVLYILKKKGAAKAALW

>Streptococcus-pneumoniae_CVT31414.1

MKSKRMEFHNKFLIVSAMLAVISWLSLGVVSFPMLAGTLGISTKAAATVVNLISAYSTVTAVISIVGAITGVGSIGSGIAATVLYILKKKGAAKAALW

>Streptococcus-pneumoniae_CVS70052.1

MKSKRMEFHNKFLIVSAMLAVISWLSLGVVSFPMLAGTLGISTKAAATVVNLISAYSTVTAVISIVGAITGVGSIGSGIAATVLYILKKKGAAKAALW

>Streptococcus-pneumoniae_CVR49339.1

MKSKRMEFHNKFLIVSAMLAVISWLSLGVVSFPMLAGTLGISTKAAATVVNLISAYSTVTAVISIVGAITGVGSIGSGIAATVLYILKKKGAAKAALW

>Streptococcus-pneumoniae_CVK52533.1

MKSKRMEFHNKFLIVSAMLAVISWLSLGVVSFPMLAGTLGISTKAAATVVNLISAYSTVTAVISIVGAITGVGSIGSGIAATVLYILKKKGAAKAALW

>Streptococcus-pneumoniae_CVW95670.1

MKSKRMEFHNKFLIVSAMLAVISWLSLGVVSFPMLAGTLGISTKAAATVVNLISAYSTVTAVISIVGAITGVGSIGSGIAATVLYILKKKGAAKAALW

>Streptococcus-pneumoniae_CVW76661.1

MKSKRMEFHNKFLIVSAMLAVISWLSLGVVSFPMLAGTLGISTKAAATVVNLISAYSTVTAVISIVGAITGVGSIGSGIAATVLYILKKKGAAKAALW

>Streptococcus-pneumoniae_CVS58402.1

MKSKRMEFHNKFLIVSAMLAVISWLSLGVVSFPMLAGTLGISTKAAATVVNLISAYSTVTAVISIVGAITGVGSIGSGIAATVLYILKKKGAAKAALW

>Streptococcus-pneumoniae_CVO91942.1

MKSKRMEFHNKFLIVSAMLAVISWLSLGVVSFPMLAGTLGISTKAAATVVNLISAYSTVTAVISIVGAITGVGSIGSGIAATVLYILKKKGAAKAALW

>Streptococcus-pneumoniae_CVL91762.1

MKSKRMEFHNKFLIVSAMLAVISWLSLGVVSFPMLAGTLGISTKAAATVVNLISAYSTVTAVISIVGAITGVGSIGSGIAATVLYILKKKGAAKAALW

>Streptococcus-pneumoniae_CVW80982.1

MKSKRMEFHNKFLIVSAMLAVISWLSLGVVSFPMLAGTLGISTKAAATVVNLISAYSTVTAVISIVGAITGVGSIGSGIAATVLYILKKKGAAKAALW

>Streptococcus-pneumoniae_CVR32715.1

MKSKRMEFHNKFLIVSAMLAVISWLSLGVVSFPMLAGTLGISTKAAATVVNLISAYSTVTAVISIVGAITGVGSIGSGIAATVLYILKKKGAAKAALW

>Streptococcus-pneumoniae_CVM78195.1

MKSKRMEFHNKFLIVSAMLAVISWLSLGVVSFPMLAGTLGISTKAAATVVNLISAYSTVTAVISIVGAITGVGSIGSGIAATVLYILKKKGAAKAALW

>Streptococcus-pneumoniae_CVM41349.1

MKSKRMEFHNKFLIVSAMLAVISWLSLGVVSFPMLAGTLGISTKAAATVVNLISAYSTVTAVISIVGAITGVGSIGSGIAATVLYILKKKGAAKAALW

>Streptococcus-pneumoniae_CVL57330.1

MKSKRMEFHNKFLIVSAMLAVISWLSLGVVSFPMLAGTLGISTKAAATVVNLISAYSTVTAVISIVGAITGVGSIGSGIAATVLYILKKKGAAKAALW

>Streptococcus-pneumoniae_CVW83678.1

MKSKRMEFHNKFLIVSAMLAVISWLSLGVVSFPMLAGTLGISTKAAATVVNLISAYSTVTAVISIVGAITGVGSIGSGIAATVLYILKKKGAAKAALW

>Streptococcus-pneumoniae_CVU03825.1

MKSKRMEFHNKFLIVSAMLAVISWLSLGVVSFPMLAGTLGISTKAAATVVNLISAYSTVTAVISIVGAITGVGSIGSGIAATVLYILKKKGAAKAALW

>Streptococcus-pneumoniae_CVP79901.1

MKSKRMEFHNKFLIVSAMLAVISWLSLGVVSFPMLAGTLGISTKAAATVVNLISAYSTVTAVISIVGAITGVGSIGSGIAATVLYILKKKGAAKAALW

>Streptococcus-pneumoniae_CVM92358.1

MKSKRMEFHNKFLIVSAMLAVISWLSLGVVSFPMLAGTLGISTKAAATVVNLISAYSTVTAVISIVGAITGVGSIGSGIAATVLYILKKKGAAKAALW

>Streptococcus-pneumoniae_CVN91204.1

MKSKRMEFHNKFLIVSAMLAVISWLSLGVVSFPMLAGTLGISTKAAATVVNLISAYSTVTAVISIVGAITGVGSIGSGIAATVLYILKKKGAAKAALW

>Streptococcus-pneumoniae_CVR74651.1

MKSKRMEFHNKFLIVSAMLAVISWLSLGVVSFPMLAGTLGISTKAAATVVNLISAYSTVTAVISIVGAITGVGSIGSGIAATVLYILKKKGAAKAALW

>Streptococcus-pneumoniae_CVP38238.1

MKSKRMEFHNKFLIVSAMLAVISWLSLGVVSFPMLAGTLGISTKAAATVVNLISAYSTVTAVISIVGAITGVGSIGSGIAATVLYILKKKGAAKAALW

>Streptococcus-pneumoniae_CVM68841.1

MKSKRMEFHNKFLIVSAMLAVISWLSLGVVSFPMLAGTLGISTKAAATVVNLISAYSTVTAVISIVGAITGVGSIGSGIAATVLYILKKKGAAKAALW

>Streptococcus-pneumoniae_CVM73390.1

MKSKRMEFHNKFLIVSAMLAVISWLSLGVVSFPMLAGTLGISTKAAATVVNLISAYSTVTAVISIVGAITGVGSIGSGIAATVLYILKKKGAAKAALW

>Streptococcus-pneumoniae_CVW41782.1

MKSKRMEFHNKFLIVSAMLAVISWLSLGVVSFPMLAGTLGISTKAAATVVNLISAYSTVTAVISIVGAITGVGSIGSGIAATVLYILKKKGAAKAALW

>Streptococcus-pneumoniae_CVV61639.1

MKSKRMEFHNKFLIVSAMLAVISWLSLGVVSFPMLAGTLGISTKAAATVVNLISAYSTVTAVISIVGAITGVGSIGSGIAATVLYILKKKGAAKAALW

>Streptococcus-pneumoniae_CVP53298.1

MKSKRMEFHNKFLIVSAMLAVISWLSLGVVSFPMLAGTLGISTKAAATVVNLISAYSTVTAVISIVGAITGVGSIGSGIAATVLYILKKKGAAKAALW

>Streptococcus-pneumoniae_CVN60446.1

MKSKRMEFHNKFLIVSAMLAVISWLSLGVVSFPMLAGTLGISTKAAATVVNLISAYSTVTAVISIVGAITGVGSIGSGIAATVLYILKKKGAAKAALW

>Streptococcus-pneumoniae_CVL81540.1

MKSKRMEFHNKFLIVSAMLAVISWLSLGVVSFPMLAGTLGISTKAAATVVNLISAYSTVTAVISIVGAITGVGSIGSGIAATVLYILKKKGAAKAALW

>Streptococcus-pneumoniae_CVU51210.1

MKSKRMEFHNKFLIVSAMLAVISWLSLGVVSFPMLAGTLGISTKAAATVVNLISAYSTVTAVISIVGAITGVGSIGSGIAATVLYILKKKGAAKAALW

>Streptococcus-pneumoniae_CVR87200.1

MKSKRMEFHNKFLIVSAMLAVISWLSLGVVSFPMLAGTLGISTKAAATVVNLISAYSTVTAVISIVGAITGVGSIGSGIAATVLYILKKKGAAKAALW

>Streptococcus-pneumoniae_CVN71227.1

MKSKRMEFHNKFLIVSAMLAVISWLSLGVVSFPMLAGTLGISTKAAATVVNLISAYSTVTAVISIVGAITGVGSIGSGIAATVLYILKKKGAAKAALW

>Streptococcus-pneumoniae_CVX49710.1

MKSKRMEFHNKFLIVSAMLAVISWLSLGVVSFPMLAGTLGISTKAAATVVNLISAYSTVTAVISIVGAITGVGSIGSGIAATVLYILKKKGAAKAALW

>Streptococcus-pneumoniae_CVR48359.1

MKSKRMEFHNKFLIVSAMLAVISWLSLGVVSFPMLAGTLGISTKAAATVVNLISAYSTVTAVISIVGAITGVGSIGSGIAATVLYILKKKGAAKAALW

>Streptococcus-pneumoniae_CVP96213.1

MKSKRMEFHNKFLIVSAMLAVISWLSLGVVSFPMLAGTLGISTKAAATVVNLISAYSTVTAVISIVGAITGVGSIGSGIAATVLYILKKKGAAKAALW

>Streptococcus-pneumoniae_CVK61248.1

MKSKRMEFHNKFLIVSAMLAVISWLSLGVVSFPMLAGTLGISTKAAATVVNLISAYSTVTAVISIVGAITGVGSIGSGIAATVLYILKKKGAAKAALW

>Streptococcus-pneumoniae_CVO69223.1

MKSKRMEFHNKFLIVSAMLAVISWLSLGVVSFPMLAGTLGISTKAAATVVNLISAYSTVTAVISIVGAITGVGSIGSGIAATVLYILKKKGAAKAALW

>Streptococcus-pneumoniae_CVM74494.1

MKSKRMEFHNKFLIVSAMLAVISWLSLGVVSFPMLAGTLGISTKAAATVVNLISAYSTVTAVISIVGAITGVGSIGSGIAATVLYILKKKGAAKAALW

>Streptococcus-pneumoniae_CVP81431.1

MKSKRMEFHNKFLIVSAMLAVISWLSLGVVSFPMLAGTLGISTKAAATVVNLISAYSTVTAVISIVGAITGVGSIGSGIAATVLYILKKKGAAKAALW

>Streptococcus-pneumoniae_CVR02704.1

MKSKRMEFHNKFLIVSAMLAVISWLSLGVVSFPMLAGTLGISTKAAATVVNLISAYSTVTAVISIVGAITGVGSIGSGIAATVLYILKKKGAAKAALW

>Streptococcus-pneumoniae_CVT73849.1

MKSKRMEFHNKFLIVSAMLAVISWLSLGVVSFPMLAGTLGISTKAAATVVNLISAYSTVTAVISIVGAITGVGSIGSGIAATVLYILKKKGAAKAALW

>Streptococcus-pneumoniae_CVK47107.1

MKSKRMEFHNKFLIVSAMLAVISWLSLGVVSFPMLAGTLGISTKAAATVVNLISAYSTVTAVISIVGAITGVGSIGSGIAATVLYILKKKGAAKAALW

>Streptococcus-pneumoniae_CVO52500.1

MKSKRMEFHNKFLIVSAMLAVISWLSLGVVSFPMLAGTLGISTKAAATVVNLISAYSTVTAVISIVGAITGVGSIGSGIAATVLYILKKKGAAKAALW

>Streptococcus-pneumoniae_CVT49064.1

MKSKRMEFHNKFLIVSAMLAVISWLSLGVVSFPMLAGTLGISTKAAATVVNLISAYSTVTAVISIVGAITGVGSIGSGIAATVLYILKKKGAAKAALW

>Streptococcus-pneumoniae_CVU22460.1

MKSKRMEFHNKFLIVSAMLAVISWLSLGVVSFPMLAGTLGISTKAAATVVNLISAYSTVTAVISIVGAITGVGSIGSGIAATVLYILKKKGAAKAALW

>Streptococcus-pneumoniae_CVX85549.1

MKSKRMEFHNKFLIVSAMLAVISWLSLGVVSFPMLAGTLGISTKAAATVVNLISAYSTVTAVISIVGAITGVGSIGSGIAATVLYILKKKGAAKAALW

>Streptococcus-pneumoniae_CVV76258.1

MKSKRMEFHNKFLIVSAMLAVISWLSLGVVSFPMLAGTLGISTKAAATVVNLISAYSTVTAVISIVGAITGVGSIGSGIAATVLYILKKKGAAKAALW

>Streptococcus-pneumoniae_CVO00001.1

MKSKRMEFHNKFLIVSAMLAVISWLSLGVVSFPMLAGTLGISTKAAATVVNLISAYSTVTAVISIVGAITGVGSIGSGIAATVLYILKKKGAAKAALW

>Streptococcus-pneumoniae_CVS99051.1

MKSKRMEFHNKFLIVSAMLAVISWLSLGVVSFPMLAGTLGISTKAAATVVNLISAYSTVTAVISIVGAITGVGSIGSGIAATVLYILKKKGAAKAALW

>Streptococcus-pneumoniae_CVV41698.1

MKSKRMEFHNKFLIVSAMLAVISWLSLGVVSFPMLAGTLGISTKAAATVVNLISAYSTVTAVISIVGAITGVGSIGSGIAATVLYILKKKGAAKAALW

>Streptococcus-pneumoniae_CVK49835.1

MKSKRMEFHNKFLIVSAMLAVISWLSLGVVSFPMLAGTLGISTKAAATVVNLISAYSTVTAVISIVGAITGVGSIGSGIAATVLYILKKKGAAKAALW

>Streptococcus-pneumoniae_CVR92139.1

MKSKRMEFHNKFLIVSAMLAVISWLSLGVVSFPMLAGTLGISTKAAATVVNLISAYSTVTAVISIVGAITGVGSIGSGIAATVLYILKKKGAAKAALW

>Streptococcus-pneumoniae_CVS66006.1

MKSKRMEFHNKFLIVSAMLAVISWLSLGVVSFPMLAGTLGISTKAAATVVNLISAYSTVTAVISIVGAITGVGSIGSGIAATVLYILKKKGAAKAALW

>Streptococcus-pneumoniae_CVU36799.1

MKSKRMEFHNKFLIVSAMLAVISWLSLGVVSFPMLAGTLGISTKAAATVVNLISAYSTVTAVISIVGAITGVGSIGSGIAATVLYILKKKGAAKAALW

>Streptococcus-pneumoniae_CVY43973.1

MKSKRMEFHNKFLIVSAMLAVISWLSLGVVSFPMLAGTLGISTKAAATVVNLISAYSTVTAVISIVGAITGVGSIGSGIAATVLYILKKKGAAKAALW

>Streptococcus-pneumoniae_CVO14520.1

MKSKRMEFHNKFLIVSAMLAVISWLSLGVVSFPMLAGTLGISTKAAATVVNLISAYSTVTAVISIVGAITGVGSIGSGIAATVLYILKKKGAAKAALW

>Streptococcus-pneumoniae_CVQ62039.1

MKSKRMEFHNKFLIVSAMLAVISWLSLGVVSFPMLAGTLGISTKAAATVVNLISAYSTVTAVISIVGAITGVGSIGSGIAATVLYILKKKGAAKAALW

>Streptococcus-pneumoniae_CVN68277.1

MKSKRMEFHNKFLIVSAMLAVISWLSLGVVSFPMLAGTLGISTKAAATVVNLISAYSTVTAVISIVGAITGVGSIGSGIAATVLYILKKKGAAKAALW

>Streptococcus-pneumoniae_CVT97935.1

MKSKRMEFHNKFLIVSAMLAVISWLSLGVVSFPMLAGTLGISTKAAATVVNLISAYSTVTAVISIVGAITGVGSIGSGIAATVLYILKKKGAAKAALW

>Streptococcus-pneumoniae_CVN07944.1

MKSKRMEFHNKFLIVSAMLAVISWLSLGVVSFPMLAGTLGISTKAAATVVNLISAYSTVTAVISIVGAITGVGSIGSGIAATVLYILKKKGAAKAALW

>Streptococcus-pneumoniae_CVT18312.1

MKSKRMEFHNKFLIVSAMLAVISWLSLGVVSFPMLAGTLGISTKAAATVVNLISAYSTVTAVISIVGAITGVGSIGSGIAATVLYILKKKGAAKAALW

>Streptococcus-pneumoniae_CVR16186.1

MKSKRMEFHNKFLIVSAMLAVISWLSLGVVSFPMLAGTLGISTKAAATVVNLISAYSTVTAVISIVGAITGVGSIGSGIAATVLYILKKKGAAKAALW

>Streptococcus-pneumoniae_CVX58297.1

MKSKRMEFHNKFLIVSAMLAVISWLSLGVVSFPMLAGTLGISTKAAATVVNLISAYSTVTAVISIVGAITGVGSIGSGIAATVLYILKKKGAAKAALW

>Streptococcus-pneumoniae_CVK53472.1

MKSKRMEFHNKFLIVSAMLAVISWLSLGVVSFPMLAGTLGISTKAAATVVNLISAYSTVTAVISIVGAITGVGSIGSGIAATVLYILKKKGAAKAALW

>Streptococcus-pneumoniae_CVU38067.1

MKSKRMEFHNKFLIVSAMLAVISWLSLGVVSFPMLAGTLGISTKAAATVVNLISAYSTVTAVISIVGAITGVGSIGSGIAATVLYILKKKGAAKAALW

>Streptococcus-pneumoniae_CVL39845.1

MKSKRMEFHNKFLIVSAMLAVISWLSLGVVSFPMLAGTLGISTKAAATVVNLISAYSTVTAVISIVGAITGVGSIGSGIAATVLYILKKKGAAKAALW

>Streptococcus-pneumoniae_CVQ96702.1

MKSKRMEFHNKFLIVSAMLAVISWLSLGVVSFPMLAGTLGISTKAAATVVNLISAYSTVTAVISIVGAITGVGSIGSGIAATVLYILKKKGAAKAALW

>Streptococcus-pneumoniae_CVO14925.1

MKSKRMEFHNKFLIVSAMLAVISWLSLGVVSFPMLAGTLGISTKAAATVVNLISAYSTVTAVISIVGAITGVGSIGSGIAATVLYILKKKGAAKAALW

>Streptococcus-pneumoniae_CVT85446.1

MKSKRMEFHNKFLIVSAMLAVISWLSLGVVSFPMLAGTLGISTKAAATVVNLISAYSTVTAVISIVGAITGVGSIGSGIAATVLYILKKKGAAKAALW

>Streptococcus-pneumoniae_CVN77655.1

MKSKRMEFHNKFLIVSAMLAVISWLSLGVVSFPMLAGTLGISTKAAATVVNLISAYSTVTAVISIVGAITGVGSIGSGIAATVLYILKKKGAAKAALW

>Streptococcus-pneumoniae_CVS43794.1

MKSKRMEFHNKFLIVSAMLAVISWLSLGVVSFPMLAGTLGISTKAAATVVNLISAYSTVTAVISIVGAITGVGSIGSGIAATVLYILKKKGAAKAALW

>Streptococcus-pneumoniae_CVU04876.1

MKSKRMEFHNKFLIVSAMLAVISWLSLGVVSFPMLAGTLGISTKAAATVVNLISAYSTVTAVISIVGAITGVGSIGSGIAATVLYILKKKGAAKAALW

>Streptococcus-pneumoniae_CVM45565.1

MKSKRMEFHNKFLIVSAMLAVISWLSLGVVSFPMLAGTLGISTKAAATVVNLISAYSTVTAVISIVGAITGVGSIGSGIAATVLYILKKKGAAKAALW

>Streptococcus-pneumoniae_CVR11020.1

MKSKRMEFHNKFLIVSAMLAVISWLSLGVVSFPMLAGTLGISTKAAATVVNLISAYSTVTAVISIVGAITGVGSIGSGIAATVLYILKKKGAAKAALW

>Streptococcus-pneumoniae_CVR28546.1

MKSKRMEFHNKFLIVSAMLAVISWLSLGVVSFPMLAGTLGISTKAAATVVNLISAYSTVTAVISIVGAITGVGSIGSGIAATVLYILKKKGAAKAALW

>Streptococcus-pneumoniae_CVS28095.1

MKSKRMEFHNKFLIVSAMLAVISWLSLGVVSFPMLAGTLGISTKAAATVVNLISAYSTVTAVISIVGAITGVGSIGSGIAATVLYILKKKGAAKAALW

>Streptococcus-pneumoniae_CVR80903.1

MKSKRMEFHNKFLIVSAMLAVISWLSLGVVSFPMLAGTLGISTKAAATVVNLISAYSTVTAVISIVGAITGVGSIGSGIAATVLYILKKKGAAKAALW

>Streptococcus-pneumoniae_CVS22646.1

MKSKRMEFHNKFLIVSAMLAVISWLSLGVVSFPMLAGTLGISTKAAATVVNLISAYSTVTAVISIVGAITGVGSIGSGIAATVLYILKKKGAAKAALW

>Streptococcus-pneumoniae_CVL91598.1

MKSKRMEFHNKFLIVSAMLAVISWLSLGVVSFPMLAGTLGISTKAAATVVNLISAYSTVTAVISIVGAITGVGSIGSGIAATVLYILKKKGAAKAALW

>Streptococcus-pneumoniae_CVP85589.1

MKSKRMEFHNKFLIVSAMLAVISWLSLGVVSFPMLAGTLGISTKAAATVVNLISAYSTVTAVISIVGAITGVGSIGSGIAATVLYILKKKGAAKAALW

>Streptococcus-pneumoniae_CVO57834.1

MKSKRMEFHNKFLIVSAMLAVISWLSLGVVSFPMLAGTLGISTKAAATVVNLISAYSTVTAVISIVGAITGVGSIGSGIAATVLYILKKKGAAKAALW

>Streptococcus-pneumoniae_CVR32018.1

MKSKRMEFHNKFLIVSAMLAVISWLSLGVVSFPMLAGTLGISTKAAATVVNLISAYSTVTAVISIVGAITGVGSIGSGIAATVLYILKKKGAAKAALW

>Streptococcus-pneumoniae_CVQ16846.1

MKSKRMEFHNKFLIVSAMLAVISWLSLGVVSFPMLAGTLGISTKAAATVVNLISAYSTVTAVISIVGAITGVGSIGSGIAATVLYILKKKGAAKAALW

>Streptococcus-pneumoniae_CVT42951.1

MKSKRMEFHNKFLIVSAMLAVISWLSLGVVSFPMLAGTLGISTKAAATVVNLISAYSTVTAVISIVGAITGVGSIGSGIAATVLYILKKKGAAKAALW

>Streptococcus-pneumoniae_CVL35418.1

MKSKRMEFHNKFLIVSAMLAVISWLSLGVVSFPMLAGTLGISTKAAATVVNLISAYSTVTAVISIVGAITGVGSIGSGIAATVLYILKKKGAAKAALW

>Streptococcus-pneumoniae_CVP96723.1

MKSKRMEFHNKFLIVSAMLAVISWLSLGVVSFPMLAGTLGISTKAAATVVNLISAYSTVTAVISIVGAITGVGSIGSGIAATVLYILKKKGAAKAALW

>Streptococcus-pneumoniae_CVS92083.1

MKSKRMEFHNKFLIVSAMLAVISWLSLGVVSFPMLAGTLGISTKAAATVVNLISAYSTVTAVISIVGAITGVGSIGSGIAATVLYILKKKGAAKAALW

>Streptococcus-pneumoniae_CVP85260.1

MKSKRMEFHNKFLIVSAMLAVISWLSLGVVSFPMLAGTLGISTKAAATVVNLISAYSTVTAVISIVGAITGVGSIGSGIAATVLYILKKKGAAKAALW

>Streptococcus-pneumoniae_CVL39659.1

MKSKRMEFHNKFLIVSAMLAVISWLSLGVVSFPMLAGTLGISTKAAATVVNLISAYSTVTAVISIVGAITGVGSIGSGIAATVLYILKKKGAAKAALW

>Streptococcus-pneumoniae_CVV89709.1

MKSKRMEFHNKFLIVSAMLAVISWLSLGVVSFPMLAGTLGISTKAAATVVNLISAYSTVTAVISIVGAITGVGSIGSGIAATVLYILKKKGAAKAALW

>Streptococcus-pneumoniae_CVK69346.1

MKSKRMEFHNKFLIVSAMLAVISWLSLGVVSFPMLAGTLGISTKAAATVVNLISAYSTVTAVISIVGAITGVGSIGSGIAATVLYILKKKGAAKAALW

>Streptococcus-pneumoniae_CVO28825.1

MKSKRMEFHNKFLIVSAMLAVISWLSLGVVSFPMLAGTLGISTKAAATVVNLISAYSTVTAVISIVGAITGVGSIGSGIAATVLYILKKKGAAKAALW

>Streptococcus-pneumoniae_CVX52038.1

MKSKRMEFHNKFLIVSAMLAVISWLSLGVVSFPMLAGTLGISTKAAATVVNLISAYSTVTAVISIVGAITGVGSIGSGIAATVLYILKKKGAAKAALW

>Streptococcus-pneumoniae_CVM85890.1

MKSKRMEFHNKFLIVSAMLAVISWLSLGVVSFPMLAGTLGISTKAAATVVNLISAYSTVTAVISIVGAITGVGSIGSGIAATVLYILKKKGAAKAALW

>Streptococcus-pneumoniae_CVX53006.1

MKSKRMEFHNKFLIVSAMLAVISWLSLGVVSFPMLAGTLGISTKAAATVVNLISAYSTVTAVISIVGAITGVGSIGSGIAATVLYILKKKGAAKAALW

>Streptococcus-pneumoniae_CVP63745.1

MKSKRMEFHNKFLIVSAMLAVISWLSLGVVSFPMLAGTLGISTKAAATVVNLISAYSTVTAVISIVGAITGVGSIGSGIAATVLYILKKKGAAKAALW

>Streptococcus-pneumoniae_CVN03369.1

MKSKRMEFHNKFLIVSAMLAVISWLSLGVVSFPMLAGTLGISTKAAATVVNLISAYSTVTAVISIVGAITGVGSIGSGIAATVLYILKKKGAAKAALW

>Streptococcus-pneumoniae_CVT30395.1

MKSKRMEFHNKFLIVSAMLAVISWLSLGVVSFPMLAGTLGISTKAAATVVNLISAYSTVTAVISIVGAITGVGSIGSGIAATVLYILKKKGAAKAALW

>Streptococcus-pneumoniae_CVM07755.1

MKSKRMEFHNKFLIVSAMLAVISWLSLGVVSFPMLAGTLGISTKAAATVVNLISAYSTVTAVISIVGAITGVGSIGSGIAATVLYILKKKGAAKAALW

>Streptococcus-pneumoniae_CVS96452.1

MKSKRMEFHNKFLIVSAMLAVISWLSLGVVSFPMLAGTLGISTKAAATVVNLISAYSTVTAVISIVGAITGVGSIGSGIAATVLYILKKKGAAKAALW

>Streptococcus-pneumoniae_CVW41904.1

MKSKRMEFHNKFLIVSAMLAVISWLSLGVVSFPMLAGTLGISTKAAATVVNLISAYSTVTAVISIVGAITGVGSIGSGIAATVLYILKKKGAAKAALW

>Streptococcus-pneumoniae_CVV66370.1

MKSKRMEFHNKFLIVSAMLAVISWLSLGVVSFPMLAGTLGISTKAAATVVNLISAYSTVTAVISIVGAITGVGSIGSGIAATVLYILKKKGAAKAALW

>Streptococcus-pneumoniae_CVQ47689.1

MKSKRMEFHNKFLIVSAMLAVISWLSLGVVSFPMLAGTLGISTKAAATVVNLISAYSTVTAVISIVGAITGVGSIGSGIAATVLYILKKKGAAKAALW

>Streptococcus-pneumoniae_CVY25244.1

MKSKRMEFHNKFLIVSAMLAVISWLSLGVVSFPMLAGTLGISTKAAATVVNLISAYSTVTAVISIVGAITGVGSIGSGIAATVLYILKKKGAAKAALW

>Streptococcus-pneumoniae_CVS71852.1

MKSKRMEFHNKFLIVSAMLAVISWLSLGVVSFPMLAGTLGISTKAAATVVNLISAYSTVTAVISIVGAITGVGSIGSGIAATVLYILKKKGAAKAALW

>Streptococcus-pneumoniae_CVN76537.1

MKSKRMEFHNKFLIVSAMLAVISWLSLGVVSFPMLAGTLGISTKAAATVVNLISAYSTVTAVISIVGAITGVGSIGSGIAATVLYILKKKGAAKAALW

>Streptococcus-pneumoniae_CVP15627.1

MKSKRMEFHNKFLIVSAMLAVISWLSLGVVSFPMLAGTLGISTKAAATVVNLISAYSTVTAVISIVGAITGVGSIGSGIAATVLYILKKKGAAKAALW

>Streptococcus-pneumoniae_CVV97016.1

MKSKRMEFHNKFLIVSAMLAVISWLSLGVVSFPMLAGTLGISTKAAATVVNLISAYSTVTAVISIVGAITGVGSIGSGIAATVLYILKKKGAAKAALW

>Streptococcus-pneumoniae_CVU63308.1

MKSKRMEFHNKFLIVSAMLAVISWLSLGVVSFPMLAGTLGISTKAAATVVNLISAYSTVTAVISIVGAITGVGSIGSGIAATVLYILKKKGAAKAALW

>Streptococcus-pneumoniae_CVO20904.1

MKSKRMEFHNKFLIVSAMLAVISWLSLGVVSFPMLAGTLGISTKAAATVVNLISAYSTVTAVISIVGAITGVGSIGSGIAATVLYILKKKGAAKAALW

>Streptococcus-pneumoniae_CVQ05183.1

MKSKRMEFHNKFLIVSAMLAVISWLSLGVVSFPMLAGTLGISTKAAATVVNLISAYSTVTAVISIVGAITGVGSIGSGIAATVLYILKKKGAAKAALW

>Streptococcus-pneumoniae_CVR73631.1

MKSKRMEFHNKFLIVSAMLAVISWLSLGVVSFPMLAGTLGISTKAAATVVNLISAYSTVTAVISIVGAITGVGSIGSGIAATVLYILKKKGAAKAALW

>Streptococcus-pneumoniae_CVL76638.1

MKSKRMEFHNKFLIVSAMLAVISWLSLGVVSFPMLAGTLGISTKAAATVVNLISAYSTVTAVISIVGAITGVGSIGSGIAATVLYILKKKGAAKAALW

>Streptococcus-pneumoniae_CVV13137.1

MKSKRMEFHNKFLIVSAMLAVISWLSLGVVSFPMLAGTLGISTKAAATVVNLISAYSTVTAVISIVGAITGVGSIGSGIAATVLYILKKKGAAKAALW

>Streptococcus-pneumoniae_CVP14130.1

MKSKRMEFHNKFLIVSAMLAVISWLSLGVVSFPMLAGTLGISTKAAATVVNLISAYSTVTAVISIVGAITGVGSIGSGIAATVLYILKKKGAAKAALW

>Streptococcus-pneumoniae_CVL68141.1

MKSKRMEFHNKFLIVSAMLAVISWLSLGVVSFPMLAGTLGISTKAAATVVNLISAYSTVTAVISIVGAITGVGSIGSGIAATVLYILKKKGAAKAALW

>Streptococcus-pneumoniae_CVT23323.1

MKSKRMEFHNKFLIVSAMLAVISWLSLGVVSFPMLAGTLGISTKAAATVVNLISAYSTVTAVISIVGAITGVGSIGSGIAATVLYILKKKGAAKAALW

>Streptococcus-pneumoniae_CVS32273.1

MKSKRMEFHNKFLIVSAMLAVISWLSLGVVSFPMLAGTLGISTKAAATVVNLISAYSTVTAVISIVGAITGVGSIGSGIAATVLYILKKKGAAKAALW

>Streptococcus-pneumoniae_CVQ50501.1

MKSKRMEFHNKFLIVSAMLAVISWLSLGVVSFPMLAGTLGISTKAAATVVNLISAYSTVTAVISIVGAITGVGSIGSGIAATVLYILKKKGAAKAALW

>Streptococcus-pneumoniae_CVR60048.1

MKSKRMEFHNKFLIVSAMLAVISWLSLGVVSFPMLAGTLGISTKAAATVVNLISAYSTVTAVISIVGAITGVGSIGSGIAATVLYILKKKGAAKAALW

>Streptococcus-pneumoniae_CVO12015.1

MKSKRMEFHNKFLIVSAMLAVISWLSLGVVSFPMLAGTLGISTKAAATVVNLISAYSTVTAVISIVGAITGVGSIGSGIAATVLYILKKKGAAKAALW

>Streptococcus-pneumoniae_CVW58886.1

MKSKRMEFHNKFLIVSAMLAVISWLSLGVVSFPMLAGTLGISTKAAATVVNLISAYSTVTAVISIVGAITGVGSIGSGIAATVLYILKKKGAAKAALW

>Streptococcus-pneumoniae_CVM49728.1

MKSKRMEFHNKFLIVSAMLAVISWLSLGVVSFPMLAGTLGISTKAAATVVNLISAYSTVTAVISIVGAITGVGSIGSGIAATVLYILKKKGAAKAALW

>Streptococcus-pneumoniae_CVQ79898.1

MKSKRMEFHNKFLIVSAMLAVISWLSLGVVSFPMLAGTLGISTKAAATVVNLISAYSTVTAVISIVGAITGVGSIGSGIAATVLYILKKKGAAKAALW

>Streptococcus-pneumoniae_CVU04362.1

MKSKRMEFHNKFLIVSAMLAVISWLSLGVVSFPMLAGTLGISTKAAATVVNLISAYSTVTAVISIVGAITGVGSIGSGIAATVLYILKKKGAAKAALW

>Streptococcus-pneumoniae_CVU89916.1

MKSKRMEFHNKFLIVSAMLAVISWLSLGVVSFPMLAGTLGISTKAAATVVNLISAYSTVTAVISIVGAITGVGSIGSGIAATVLYILKKKGAAKAALW

>Streptococcus-pneumoniae_CVW20215.1

MKSKRMEFHNKFLIVSAMLAVISWLSLGVVSFPMLAGTLGISTKAAATVVNLISAYSTVTAVISIVGAITGVGSIGSGIAATVLYILKKKGAAKAALW

>Streptococcus-pneumoniae_CVS03476.1

MKSKRMEFHNKFLIVSAMLAVISWLSLGVVSFPMLAGTLGISTKAAATVVNLISAYSTVTAVISIVGAITGVGSIGSGIAATVLYILKKKGAAKAALW

>Streptococcus-pneumoniae_CVV51334.1

MKSKRMEFHNKFLIVSAMLAVISWLSLGVVSFPMLAGTLGISTKAAATVVNLISAYSTVTAVISIVGAITGVGSIGSGIAATVLYILKKKGAAKAALW

>Streptococcus-pneumoniae_CVX42705.1

MKSKRMEFHNKFLIVSAMLAVISWLSLGVVSFPMLAGTLGISTKAAATVVNLISAYSTVTAVISIVGAITGVGSIGSGIAATVLYILKKKGAAKAALW

>Streptococcus-pneumoniae_CVS91736.1

MKSKRMEFHNKFLIVSAMLAVISWLSLGVVSFPMLAGTLGISTKAAATVVNLISAYSTVTAVISIVGAITGVGSIGSGIAATVLYILKKKGAAKAALW

>Streptococcus-pneumoniae_CVN73633.1

MKSKRMEFHNKFLIVSAMLAVISWLSLGVVSFPMLAGTLGISTKAAATVVNLISAYSTVTAVISIVGAITGVGSIGSGIAATVLYILKKKGAAKAALW

>Streptococcus-pneumoniae_CVT93259.1

MKSKRMEFHNKFLIVSAMLAVISWLSLGVVSFPMLAGTLGISTKAAATVVNLISAYSTVTAVISIVGAITGVGSIGSGIAATVLYILKKKGAAKAALW

>Streptococcus-pneumoniae_CVT23930.1

MKSKRMEFHNKFLIVSAMLAVISWLSLGVVSFPMLAGTLGISTKAAATVVNLISAYSTVTAVISIVGAITGVGSIGSGIAATVLYILKKKGAAKAALW

>Streptococcus-pneumoniae_CVT89197.1

MKSKRMEFHNKFLIVSAMLAVISWLSLGVVSFPMLAGTLGISTKAAATVVNLISAYSTVTAVISIVGAITGVGSIGSGIAATVLYILKKKGAAKAALW

>Streptococcus-pneumoniae_CVR09055.1

MKSKRMEFHNKFLIVSAMLAVISWLSLGVVSFPMLAGTLGISTKAAATVVNLISAYSTVTAVISIVGAITGVGSIGSGIAATVLYILKKKGAAKAALW

>Streptococcus-pneumoniae_CVT78111.1

MKSKRMEFHNKFLIVSAMLAVISWLSLGVVSFPMLAGTLGISTKAAATVVNLISAYSTVTAVISIVGAITGVGSIGSGIAATVLYILKKKGAAKAALW

>Streptococcus-pneumoniae_CVV89980.1

MKSKRMEFHNKFLIVSAMLAVISWLSLGVVSFPMLAGTLGISTKAAATVVNLISAYSTVTAVISIVGAITGVGSIGSGIAATVLYILKKKGAAKAALW

>Streptococcus-pneumoniae_CVS02208.1

MKSKRMEFHNKFLIVSAMLAVISWLSLGVVSFPMLAGTLGISTKAAATVVNLISAYSTVTAVISIVGAITGVGSIGSGIAATVLYILKKKGAAKAALW

>Streptococcus-pneumoniae_CVL88481.1

MKSKRMEFHNKFLIVSAMLAVISWLSLGVVSFPMLAGTLGISTKAAATVVNLISAYSTVTAVISIVGAITGVGSIGSGIAATVLYILKKKGAAKAALW

>Streptococcus-pneumoniae_CVP33272.1

MKSKRMEFHNKFLIVSAMLAVISWLSLGVVSFPMLAGTLGISTKAAATVVNLISAYSTVTAVISIVGAITGVGSIGSGIAATVLYILKKKGAAKAALW

>Streptococcus-pneumoniae_CVP34900.1

MKSKRMEFHNKFLIVSAMLAVISWLSLGVVSFPMLAGTLGISTKAAATVVNLISAYSTVTAVISIVGAITGVGSIGSGIAATVLYILKKKGAAKAALW

>Streptococcus-pneumoniae_CVQ64095.1

MKSKRMEFHNKFLIVSAMLAVISWLSLGVVSFPMLAGTLGISTKAAATVVNLISAYSTVTAVISIVGAITGVGSIGSGIAATVLYILKKKGAAKAALW

>Streptococcus-pneumoniae_CVU03002.1

MKSKRMEFHNKFLIVSAMLAVISWLSLGVVSFPMLAGTLGISTKAAATVVNLISAYSTVTAVISIVGAITGVGSIGSGIAATVLYILKKKGAAKAALW

>Streptococcus-pneumoniae_CVM17077.1

MKSKRMEFHNKFLIVSAMLAVISWLSLGVVSFPMLAGTLGISTKAAATVVNLISAYSTVTAVISIVGAITGVGSIGSGIAATVLYILKKKGAAKAALW

>Streptococcus-pneumoniae_CVR64745.1

MKSKRMEFHNKFLIVSAMLAVISWLSLGVVSFPMLAGTLGISTKAAATVVNLISAYSTVTAVISIVGAITGVGSIGSGIAATVLYILKKKGAAKAALW

>Streptococcus-pneumoniae_CVW00269.1

MKSKRMEFHNKFLIVSAMLAVISWLSLGVVSFPMLAGTLGISTKAAATVVNLISAYSTVTAVISIVGAITGVGSIGSGIAATVLYILKKKGAAKAALW

>Streptococcus-pneumoniae_CVS21233.1

MKSKRMEFHNKFLIVSAMLAVISWLSLGVVSFPMLAGTLGISTKAAATVVNLISAYSTVTAVISIVGAITGVGSIGSGIAATVLYILKKKGAAKAALW

>Streptococcus-pneumoniae_CVS17524.1

MKSKRMEFHNKFLIVSAMLAVISWLSLGVVSFPMLAGTLGISTKAAATVVNLISAYSTVTAVISIVGAITGVGSIGSGIAATVLYILKKKGAAKAALW

>Streptococcus-pneumoniae_CVV31629.1

MKSKRMEFHNKFLIVSAMLAVISWLSLGVVSFPMLAGTLGISTKAAATVVNLISAYSTVTAVISIVGAITGVGSIGSGIAATVLYILKKKGAAKAALW

>Streptococcus-pneumoniae_CVM12759.1

MKSKRMEFHNKFLIVSAMLAVISWLSLGVVSFPMLAGTLGISTKAAATVVNLISAYSTVTAVISIVGAITGVGSIGSGIAATVLYILKKKGAAKAALW

>Streptococcus-pneumoniae_CVO47928.1

MKSKRMEFHNKFLIVSAMLAVISWLSLGVVSFPMLAGTLGISTKAAATVVNLISAYSTVTAVISIVGAITGVGSIGSGIAATVLYILKKKGAAKAALW

>Streptococcus-pneumoniae_CVW87061.1

MKSKRMEFHNKFLIVSAMLAVISWLSLGVVSFPMLAGTLGISTKAAATVVNLISAYSTVTAVISIVGAITGVGSIGSGIAATVLYILKKKGAAKAALW

>Streptococcus-pneumoniae_CVW59960.1

MKSKRMEFHNKFLIVSAMLAVISWLSLGVVSFPMLAGTLGISTKAAATVVNLISAYSTVTAVISIVGAITGVGSIGSGIAATVLYILKKKGAAKAALW

>Streptococcus-pneumoniae_CVV99149.1

MKSKRMEFHNKFLIVSAMLAVISWLSLGVVSFPMLAGTLGISTKAAATVVNLISAYSTVTAVISIVGAITGVGSIGSGIAATVLYILKKKGAAKAALW

>Streptococcus-pneumoniae_CVQ41497.1

MKSKRMEFHNKFLIVSAMLAVISWLSLGVVSFPMLAGTLGISTKAAATVVNLISAYSTVTAVISIVGAITGVGSIGSGIAATVLYILKKKGAAKAALW

>Streptococcus-pneumoniae_CVM98135.1

MKSKRMEFHNKFLIVSAMLAVISWLSLGVVSFPMLAGTLGISTKAAATVVNLISAYSTVTAVISIVGAITGVGSIGSGIAATVLYILKKKGAAKAALW

>Streptococcus-pneumoniae_CVT25520.1

MKSKRMEFHNKFLIVSAMLAVISWLSLGVVSFPMLAGTLGISTKAAATVVNLISAYSTVTAVISIVGAITGVGSIGSGIAATVLYILKKKGAAKAALW

>Streptococcus-pneumoniae_CVL18234.1

MKSKRMEFHNKFLIVSAMLAVISWLSLGVVSFPMLAGTLGISTKAAATVVNLISAYSTVTAVISIVGAITGVGSIGSGIAATVLYILKKKGAAKAALW

>Streptococcus-pneumoniae_CVL31349.1

MKSKRMEFHNKFLIVSAMLAVISWLSLGVVSFPMLAGTLGISTKAAATVVNLISAYSTVTAVISIVGAITGVGSIGSGIAATVLYILKKKGAAKAALW

>Streptococcus-pneumoniae_CVP95568.1

MKSKRMEFHNKFLIVSAMLAVISWLSLGVVSFPMLAGTLGISTKAAATVVNLISAYSTVTAVISIVGAITGVGSIGSGIAATVLYILKKKGAAKAALW

>Streptococcus-pneumoniae_CVO97398.1

MKSKRMEFHNKFLIVSAMLAVISWLSLGVVSFPMLAGTLGISTKAAATVVNLISAYSTVTAVISIVGAITGVGSIGSGIAATVLYILKKKGAAKAALW

>Streptococcus-pneumoniae_CVM28626.1

MKSKRMEFHNKFLIVSAMLAVISWLSLGVVSFPMLAGTLGISTKAAATVVNLISAYSTVTAVISIVGAITGVGSIGSGIAATVLYILKKKGAAKAALW

>Streptococcus-pneumoniae_CVS94516.1

MKSKRMEFHNKFLIVSAMLAVISWLSLGVVSFPMLAGTLGISTKAAATVVNLISAYSTVTAVISIVGAITGVGSIGSGIAATVLYILKKKGAAKAALW

>Streptococcus-pneumoniae_CVV95075.1

MKSKRMEFHNKFLIVSAMLAVISWLSLGVVSFPMLAGTLGISTKAAATVVNLISAYSTVTAVISIVGAITGVGSIGSGIAATVLYILKKKGAAKAALW

>Streptococcus-pneumoniae_CVM75646.1

MKSKRMEFHNKFLIVSAMLAVISWLSLGVVSFPMLAGTLGISTKAAATVVNLISAYSTVTAVISIVGAITGVGSIGSGIAATVLYILKKKGAAKAALW

>Streptococcus-pneumoniae_CVQ33740.1

MKSKRMEFHNKFLIVSAMLAVISWLSLGVVSFPMLAGTLGISTKAAATVVNLISAYSTVTAVISIVGAITGVGSIGSGIAATVLYILKKKGAAKAALW

>Streptococcus-pneumoniae_CVT63543.1

MKSKRMEFHNKFLIVSAMLAVISWLSLGVVSFPMLAGTLGISTKAAATVVNLISAYSTVTAVISIVGAITGVGSIGSGIAATVLYILKKKGAAKAALW

>Streptococcus-pneumoniae_CVP66286.1

MKSKRMEFHNKFLIVSAMLAVISWLSLGVVSFPMLAGTLGISTKAAATVVNLISAYSTVTAVISIVGAITGVGSIGSGIAATVLYILKKKGAAKAALW

>Streptococcus-pneumoniae_CVY07999.1

MKSKRMEFHNKFLIVSAMLAVISWLSLGVVSFPMLAGTLGISTKAAATVVNLISAYSTVTAVISIVGAITGVGSIGSGIAATVLYILKKKGAAKAALW

>Streptococcus-pneumoniae_CVX11288.1

MKSKRMEFHNKFLIVSAMLAVISWLSLGVVSFPMLAGTLGISTKAAATVVNLISAYSTVTAVISIVGAITGVGSIGSGIAATVLYILKKKGAAKAALW

>Streptococcus-pneumoniae_CVL25282.1

MKSKRMEFHNKFLIVSAMLAVISWLSLGVVSFPMLAGTLGISTKAAATVVNLISAYSTVTAVISIVGAITGVGSIGSGIAATVLYILKKKGAAKAALW

>Streptococcus-pneumoniae_CVV79976.1

MKSKRMEFHNKFLIVSAMLAVISWLSLGVVSFPMLAGTLGISTKAAATVVNLISAYSTVTAVISIVGAITGVGSIGSGIAATVLYILKKKGAAKAALW

>Streptococcus-pneumoniae_CVR59254.1

MKSKRMEFHNKFLIVSAMLAVISWLSLGVVSFPMLAGTLGISTKAAATVVNLISAYSTVTAVISIVGAITGVGSIGSGIAATVLYILKKKGAAKAALW

>Streptococcus-pneumoniae_CVQ11034.1

MKSKRMEFHNKFLIVSAMLAVISWLSLGVVSFPMLAGTLGISTKAAATVVNLISAYSTVTAVISIVGAITGVGSIGSGIAATVLYILKKKGAAKAALW

>Streptococcus-pneumoniae_CVL80312.1

MKSKRMEFHNKFLIVSAMLAVISWLSLGVVSFPMLAGTLGISTKAAATVVNLISAYSTVTAVISIVGAITGVGSIGSGIAATVLYILKKKGAAKAALW

>Streptococcus-pneumoniae_CVK66143.1

MKSKRMEFHNKFLIVSAMLAVISWLSLGVVSFPMLAGTLGISTKAAATVVNLISAYSTVTAVISIVGAITGVGSIGSGIAATVLYILKKKGAAKAALW

>Streptococcus-pneumoniae_CVW82411.1

MKSKRMEFHNKFLIVSAMLAVISWLSLGVVSFPMLAGTLGISTKAAATVVNLISAYSTVTAVISIVGAITGVGSIGSGIAATVLYILKKKGAAKAALW

>Streptococcus-pneumoniae_CVQ60099.1

MKSKRMEFHNKFLIVSAMLAVISWLSLGVVSFPMLAGTLGISTKAAATVVNLISAYSTVTAVISIVGAITGVGSIGSGIAATVLYILKKKGAAKAALW

>Streptococcus-pneumoniae_CVN29824.1

MKSKRMEFHNKFLIVSAMLAVISWLSLGVVSFPMLAGTLGISTKAAATVVNLISAYSTVTAVISIVGAITGVGSIGSGIAATVLYILKKKGAAKAALW

>Streptococcus-pneumoniae_CVQ20721.1

MKSKRMEFHNKFLIVSAMLAVISWLSLGVVSFPMLAGTLGISTKAAATVVNLISAYSTVTAVISIVGAITGVGSIGSGIAATVLYILKKKGAAKAALW

>Streptococcus-pneumoniae_CVS95802.1

MKSKRMEFHNKFLIVSAMLAVISWLSLGVVSFPMLAGTLGISTKAAATVVNLISAYSTVTAVISIVGAITGVGSIGSGIAATVLYILKKKGAAKAALW

>Streptococcus-pneumoniae_CVT22824.1

MKSKRMEFHNKFLIVSAMLAVISWLSLGVVSFPMLAGTLGISTKAAATVVNLISAYSTVTAVISIVGAITGVGSIGSGIAATVLYILKKKGAAKAALW

>Streptococcus-pneumoniae_CVK65769.1

MKSKRMEFHNKFLIVSAMLAVISWLSLGVVSFPMLAGTLGISTKAAATVVNLISAYSTVTAVISIVGAITGVGSIGSGIAATVLYILKKKGAAKAALW

>Streptococcus-pneumoniae_CVX10166.1

MKSKRMEFHNKFLIVSAMLAVISWLSLGVVSFPMLAGTLGISTKAAATVVNLISAYSTVTAVISIVGAITGVGSIGSGIAATVLYILKKKGAAKAALW

>Streptococcus-pneumoniae_CVL71610.1

MKSKRMEFHNKFLIVSAMLAVISWLSLGVVSFPMLAGTLGISTKAAATVVNLISAYSTVTAVISIVGAITGVGSIGSGIAATVLYILKKKGAAKAALW

>Streptococcus-pneumoniae_CVP66135.1

MKSKRMEFHNKFLIVSAMLAVISWLSLGVVSFPMLAGTLGISTKAAATVVNLISAYSTVTAVISIVGAITGVGSIGSGIAATVLYILKKKGAAKAALW

>Streptococcus-pneumoniae_CVS39735.1

MKSKRMEFHNKFLIVSAMLAVISWLSLGVVSFPMLAGTLGISTKAAATVVNLISAYSTVTAVISIVGAITGVGSIGSGIAATVLYILKKKGAAKAALW

>Streptococcus-pneumoniae_CVY28242.1

MKSKRMEFHNKFLIVSAMLAVISWLSLGVVSFPMLAGTLGISTKAAATVVNLISAYSTVTAVISIVGAITGVGSIGSGIAATVLYILKKKGAAKAALW

>Streptococcus-pneumoniae_CVS57055.1

MKSKRMEFHNKFLIVSAMLAVISWLSLGVVSFPMLAGTLGISTKAAATVVNLISAYSTVTAVISIVGAITGVGSIGSGIAATVLYILKKKGAAKAALW

>Streptococcus-pneumoniae_CVT06096.1

MKSKRMEFHNKFLIVSAMLAVISWLSLGVVSFPMLAGTLGISTKAAATVVNLISAYSTVTAVISIVGAITGVGSIGSGIAATVLYILKKKGAAKAALW

>Streptococcus-pneumoniae_CVW39822.1

MKSKRMEFHNKFLIVSAMLAVISWLSLGVVSFPMLAGTLGISTKAAATVVNLISAYSTVTAVISIVGAITGVGSIGSGIAATVLYILKKKGAAKAALW

>Streptococcus-pneumoniae_CVO96107.1

MKSKRMEFHNKFLIVSAMLAVISWLSLGVVSFPMLAGTLGISTKAAATVVNLISAYSTVTAVISIVGAITGVGSIGSGIAATVLYILKKKGAAKAALW

>Streptococcus-pneumoniae_CVT25227.1

MKSKRMEFHNKFLIVSAMLAVISWLSLGVVSFPMLAGTLGISTKAAATVVNLISAYSTVTAVISIVGAITGVGSIGSGIAATVLYILKKKGAAKAALW

>Streptococcus-pneumoniae_CVN19853.1

MKSKRMEFHNKFLIVSAMLAVISWLSLGVVSFPMLAGTLGISTKAAATVVNLISAYSTVTAVISIVGAITGVGSIGSGIAATVLYILKKKGAAKAALW

>Streptococcus-pneumoniae_CVN95990.1

MKSKRMEFHNKFLIVSAMLAVISWLSLGVVSFPMLAGTLGISTKAAATVVNLISAYSTVTAVISIVGAITGVGSIGSGIAATVLYILKKKGAAKAALW

>Streptococcus-pneumoniae_CXF44380.1

MKSKRMEFHNKFLIVSAMLAVISWLSLGVVSFPMLAGTLGISTKAAATVVNLISAYSTVTAVISIVGAITGVGSIGSGIAATVLYILKKKGAAKAALW

>Streptococcus-pneumoniae_CWC86809.1

MKSKRMEFHNKFLIVSAMLAVISWLSLGVVSFPMLAGTLGISTKAAATVVNLISAYSTVTAVISIVGAITGVGSIGSGIAATVLYILKKKGAAKAALW

>Streptococcus-pneumoniae_CWB11328.1

MKSKRMEFHNKFLIVSAMLAVISWLSLGVVSFPMLAGTLGISTKAAATVVNLISAYSTVTAVISIVGAITGVGSIGSGIAATVLYILKKKGAAKAALW

>Streptococcus-pneumoniae_CWI25415.1

MKSKRMEFHNKFLIVSAMLAVISWLSLGVVSFPMLAGTLGISTKAAATVVNLISAYSTVTAVISIVGAITGVGSIGSGIAATVLYILKKKGAAKAALW

>Streptococcus-pneumoniae_CXF25471.1

MKSKRMEFHNKFLIVSAMLAVISWLSLGVVSFPMLAGTLGISTKAAATVVNLISAYSTVTAVISIVGAITGVGSIGSGIAATVLYILKKKGAAKAALW

>Streptococcus-pneumoniae_CWG01134.1

MKSKRMEFHNKFLIVSAMLAVISWLSLGVVSFPMLAGTLGISTKAAATVVNLISAYSTVTAVISIVGAITGVGSIGSGIAATVLYILKKKGAAKAALW

>Streptococcus-pneumoniae_CWB02410.1

MKSKRMEFHNKFLIVSAMLAVISWLSLGVVSFPMLAGTLGISTKAAATVVNLISAYSTVTAVISIVGAITGVGSIGSGIAATVLYILKKKGAAKAALW

>Streptococcus-pneumoniae_CWD27670.1

MKSKRMEFHNKFLIVSAMLAVISWLSLGVVSFPMLAGTLGISTKAAATVVNLISAYSTVTAVISIVGAITGVGSIGSGIAATVLYILKKKGAAKAALW

>Streptococcus-pneumoniae_CWI46928.1

MKSKRMEFHNKFLIVSAMLAVISWLSLGVVSFPMLAGTLGISTKAAATVVNLISAYSTVTAVISIVGAITGVGSIGSGIAATVLYILKKKGAAKAALW

>Streptococcus-pneumoniae_CWB47483.1

MKSKRMEFHNKFLIVSAMLAVISWLSLGVVSFPMLAGTLGISTKAAATVVNLISAYSTVTAVISIVGAITGVGSIGSGIAATVLYILKKKGAAKAALW

>Streptococcus-pneumoniae_CWM38274.1

MKSKRMEFHNKFLIVSAMLAVISWLSLGVVSFPMLAGTLGISTKAAATVVNLISAYSTVTAVISIVGAITGVGSIGSGIAATVLYILKKKGAAKAALW

>Streptococcus-pneumoniae_CWH01347.1

MKSKRMEFHNKFLIVSAMLAVISWLSLGVVSFPMLAGTLGISTKAAATVVNLISAYSTVTAVISIVGAITGVGSIGSGIAATVLYILKKKGAAKAALW

>Streptococcus-pneumoniae_CWA32973.1

MKSKRMEFHNKFLIVSAMLAVISWLSLGVVSFPMLAGTLGISTKAAATVVNLISAYSTVTAVISIVGAITGVGSIGSGIAATVLYILKKKGAAKAALW

>Streptococcus-pneumoniae_CWE47840.1

MKSKRMEFHNKFLIVSAMLAVISWLSLGVVSFPMLAGTLGISTKAAATVVNLISAYSTVTAVISIVGAITGVGSIGSGIAATVLYILKKKGAAKAALW

>Streptococcus-pneumoniae_CWB36353.1

MKSKRMEFHNKFLIVSAMLAVISWLSLGVVSFPMLAGTLGISTKAAATVVNLISAYSTVTAVISIVGAITGVGSIGSGIAATVLYILKKKGAAKAALW

>Streptococcus-pneumoniae_CWF33923.1

MKSKRMEFHNKFLIVSAMLAVISWLSLGVVSFPMLAGTLGISTKAAATVVNLISAYSTVTAVISIVGAITGVGSIGSGIAATVLYILKKKGAAKAALW

>Streptococcus-pneumoniae_CWJ12765.1

MKSKRMEFHNKFLIVSAMLAVISWLSLGVVSFPMLAGTLGISTKAAATVVNLISAYSTVTAVISIVGAITGVGSIGSGIAATVLYILKKKGAAKAALW

>Streptococcus-pneumoniae_CWF39813.1

MKSKRMEFHNKFLIVSAMLAVISWLSLGVVSFPMLAGTLGISTKAAATVVNLISAYSTVTAVISIVGAITGVGSIGSGIAATVLYILKKKGAAKAALW

>Streptococcus-pneumoniae_CWA48394.1

MKSKRMEFHNKFLIVSAMLAVISWLSLGVVSFPMLAGTLGISTKAAATVVNLISAYSTVTAVISIVGAITGVGSIGSGIAATVLYILKKKGAAKAALW

>Streptococcus-pneumoniae_CWG74954.1

MKSKRMEFHNKFLIVSAMLAVISWLSLGVVSFPMLAGTLGISTKAAATVVNLISAYSTVTAVISIVGAITGVGSIGSGIAATVLYILKKKGAAKAALW

>Streptococcus-pneumoniae_CWH77137.1

MKSKRMEFHNKFLIVSAMLAVISWLSLGVVSFPMLAGTLGISTKAAATVVNLISAYSTVTAVISIVGAITGVGSIGSGIAATVLYILKKKGAAKAALW

>Streptococcus-pneumoniae_CWH95377.1

MKSKRMEFHNKFLIVSAMLAVISWLSLGVVSFPMLAGTLGISTKAAATVVNLISAYSTVTAVISIVGAITGVGSIGSGIAATVLYILKKKGAAKAALW

>Streptococcus-pneumoniae_CWB58154.1

MKSKRMEFHNKFLIVSAMLAVISWLSLGVVSFPMLAGTLGISTKAAATVVNLISAYSTVTAVISIVGAITGVGSIGSGIAATVLYILKKKGAAKAALW

>Streptococcus-pneumoniae_CWG36756.1

MKSKRMEFHNKFLIVSAMLAVISWLSLGVVSFPMLAGTLGISTKAAATVVNLISAYSTVTAVISIVGAITGVGSIGSGIAATVLYILKKKGAAKAALW

>Streptococcus-pneumoniae_CWH77363.1

MKSKRMEFHNKFLIVSAMLAVISWLSLGVVSFPMLAGTLGISTKAAATVVNLISAYSTVTAVISIVGAITGVGSIGSGIAATVLYILKKKGAAKAALW

>Streptococcus-pneumoniae_CWH89623.1

MKSKRMEFHNKFLIVSAMLAVISWLSLGVVSFPMLAGTLGISTKAAATVVNLISAYSTVTAVISIVGAITGVGSIGSGIAATVLYILKKKGAAKAALW

>Streptococcus-pneumoniae_CWF19949.1

MKSKRMEFHNKFLIVSAMLAVISWLSLGVVSFPMLAGTLGISTKAAATVVNLISAYSTVTAVISIVGAITGVGSIGSGIAATVLYILKKKGAAKAALW

>Streptococcus-pneumoniae_CVY69115.1

MKSKRMEFHNKFLIVSAMLAVISWLSLGVVSFPMLAGTLGISTKAAATVVNLISAYSTVTAVISIVGAITGVGSIGSGIAATVLYILKKKGAAKAALW

>Streptococcus-pneumoniae_CWD91421.1

MKSKRMEFHNKFLIVSAMLAVISWLSLGVVSFPMLAGTLGISTKAAATVVNLISAYSTVTAVISIVGAITGVGSIGSGIAATVLYILKKKGAAKAALW

>Streptococcus-pneumoniae_CWC28006.1

MKSKRMEFHNKFLIVSAMLAVISWLSLGVVSFPMLAGTLGISTKAAATVVNLISAYSTVTAVISIVGAITGVGSIGSGIAATVLYILKKKGAAKAALW

>Streptococcus-pneumoniae_CWL25447.1

MKSKRMEFHNKFLIVSAMLAVISWLSLGVVSFPMLAGTLGISTKAAATVVNLISAYSTVTAVISIVGAITGVGSIGSGIAATVLYILKKKGAAKAALW

>Streptococcus-pneumoniae_CWG57057.1

MKSKRMEFHNKFLIVSAMLAVISWLSLGVVSFPMLAGTLGISTKAAATVVNLISAYSTVTAVISIVGAITGVGSIGSGIAATVLYILKKKGAAKAALW

>Streptococcus-pneumoniae_CWF66973.1

MKSKRMEFHNKFLIVSAMLAVISWLSLGVVSFPMLAGTLGISTKAAATVVNLISAYSTVTAVISIVGAITGVGSIGSGIAATVLYILKKKGAAKAALW

>Streptococcus-pneumoniae_CWG84442.1

MKSKRMEFHNKFLIVSAMLAVISWLSLGVVSFPMLAGTLGISTKAAATVVNLISAYSTVTAVISIVGAITGVGSIGSGIAATVLYILKKKGAAKAALW

>Streptococcus-pneumoniae_CVZ98225.1

MKSKRMEFHNKFLIVSAMLAVISWLSLGVVSFPMLAGTLGISTKAAATVVNLISAYSTVTAVISIVGAITGVGSIGSGIAATVLYILKKKGAAKAALW

>Streptococcus-pneumoniae_CWD18715.1

MKSKRMEFHNKFLIVSAMLAVISWLSLGVVSFPMLAGTLGISTKAAATVVNLISAYSTVTAVISIVGAITGVGSIGSGIAATVLYILKKKGAAKAALW

>Streptococcus-pneumoniae_CWD76874.1

MKSKRMEFHNKFLIVSAMLAVISWLSLGVVSFPMLAGTLGISTKAAATVVNLISAYSTVTAVISIVGAITGVGSIGSGIAATVLYILKKKGAAKAALW

>Streptococcus-pneumoniae_CWG91811.1

MKSKRMEFHNKFLIVSAMLAVISWLSLGVVSFPMLAGTLGISTKAAATVVNLISAYSTVTAVISIVGAITGVGSIGSGIAATVLYILKKKGAAKAALW

>Streptococcus-pneumoniae_CWD23708.1

MKSKRMEFHNKFLIVSAMLAVISWLSLGVVSFPMLAGTLGISTKAAATVVNLISAYSTVTAVISIVGAITGVGSIGSGIAATVLYILKKKGAAKAALW

>Streptococcus-pneumoniae_CWH04675.1

MKSKRMEFHNKFLIVSAMLAVISWLSLGVVSFPMLAGTLGISTKAAATVVNLISAYSTVTAVISIVGAITGVGSIGSGIAATVLYILKKKGAAKAALW

>Streptococcus-pneumoniae_CWF77404.1

MKSKRMEFHNKFLIVSAMLAVISWLSLGVVSFPMLAGTLGISTKAAATVVNLISAYSTVTAVISIVGAITGVGSIGSGIAATVLYILKKKGAAKAALW

>Streptococcus-pneumoniae_CWG44125.1

MKSKRMEFHNKFLIVSAMLAVISWLSLGVVSFPMLAGTLGISTKAAATVVNLISAYSTVTAVISIVGAITGVGSIGSGIAATVLYILKKKGAAKAALW

>Streptococcus-pneumoniae_CWJ17705.1

MKSKRMEFHNKFLIVSAMLAVISWLSLGVVSFPMLAGTLGISTKAAATVVNLISAYSTVTAVISIVGAITGVGSIGSGIAATVLYILKKKGAAKAALW

>Streptococcus-pneumoniae_CWJ79233.1

MKSKRMEFHNKFLIVSAMLAVISWLSLGVVSFPMLAGTLGISTKAAATVVNLISAYSTVTAVISIVGAITGVGSIGSGIAATVLYILKKKGAAKAALW

>Streptococcus-pneumoniae_CVY45633.1

MKSKRMEFHNKFLIVSAMLAVISWLSLGVVSFPMLAGTLGISTKAAATVVNLISAYSTVTAVISIVGAITGVGSIGSGIAATVLYILKKKGAAKAALW

>Streptococcus-pneumoniae_CWH79283.1

MKSKRMEFHNKFLIVSAMLAVISWLSLGVVSFPMLAGTLGISTKAAATVVNLISAYSTVTAVISIVGAITGVGSIGSGIAATVLYILKKKGAAKAALW

>Streptococcus-pneumoniae_CWI43207.1

MKSKRMEFHNKFLIVSAMLAVISWLSLGVVSFPMLAGTLGISTKAAATVVNLISAYSTVTAVISIVGAITGVGSIGSGIAATVLYILKKKGAAKAALW

>Streptococcus-pneumoniae_CWG99362.1

MKSKRMEFHNKFLIVSAMLAVISWLSLGVVSFPMLAGTLGISTKAAATVVNLISAYSTVTAVISIVGAITGVGSIGSGIAATVLYILKKKGAAKAALW

>Streptococcus-pneumoniae_CWJ12479.1

MKSKRMEFHNKFLIVSAMLAVISWLSLGVVSFPMLAGTLGISTKAAATVVNLISAYSTVTAVISIVGAITGVGSIGSGIAATVLYILKKKGAAKAALW

>Streptococcus-pneumoniae_CWJ55294.1

MKSKRMEFHNKFLIVSAMLAVISWLSLGVVSFPMLAGTLGISTKAAATVVNLISAYSTVTAVISIVGAITGVGSIGSGIAATVLYILKKKGAAKAALW

>Streptococcus-pneumoniae_CWE18019.1

MKSKRMEFHNKFLIVSAMLAVISWLSLGVVSFPMLAGTLGISTKAAATVVNLISAYSTVTAVISIVGAITGVGSIGSGIAATVLYILKKKGAAKAALW

>Streptococcus-pneumoniae_CWI91199.1

MKSKRMEFHNKFLIVSAMLAVISWLSLGVVSFPMLAGTLGISTKAAATVVNLISAYSTVTAVISIVGAITGVGSIGSGIAATVLYILKKKGAAKAALW

>Streptococcus-pneumoniae_CWG57362.1

MKSKRMEFHNKFLIVSAMLAVISWLSLGVVSFPMLAGTLGISTKAAATVVNLISAYSTVTAVISIVGAITGVGSIGSGIAATVLYILKKKGAAKAALW

>Streptococcus-pneumoniae_CWG48209.1

MKSKRMEFHNKFLIVSAMLAVISWLSLGVVSFPMLAGTLGISTKAAATVVNLISAYSTVTAVISIVGAITGVGSIGSGIAATVLYILKKKGAAKAALW

>Streptococcus-pneumoniae_CWK77098.1

MKSKRMEFHNKFLIVSAMLAVISWLSLGVVSFPMLAGTLGISTKAAATVVNLISAYSTVTAVISIVGAITGVGSIGSGIAATVLYILKKKGAAKAALW

>Streptococcus-pneumoniae_CVY10970.1

MKSKRMEFHNKFLIVSAMLAVISWLSLGVVSFPMLAGTLGISTKAAATVVNLISAYSTVTAVISIVGAITGVGSIGSGIAATVLYILKKKGAAKAALW

>Streptococcus-pneumoniae_CWF92954.1

MKSKRMEFHNKFLIVSAMLAVISWLSLGVVSFPMLAGTLGISTKAAATVVNLISAYSTVTAVISIVGAITGVGSIGSGIAATVLYILKKKGAAKAALW

>Streptococcus-pneumoniae_CWL15970.1

MKSKRMEFHNKFLIVSAMLAVISWLSLGVVSFPMLAGTLGISTKAAATVVNLISAYSTVTAVISIVGAITGVGSIGSGIAATVLYILKKKGAAKAALW

>Streptococcus-pneumoniae_CVZ91322.1

MKSKRMEFHNKFLIVSAMLAVISWLSLGVVSFPMLAGTLGISTKAAATVVNLISAYSTVTAVISIVGAITGVGSIGSGIAATVLYILKKKGAAKAALW

>Streptococcus-pneumoniae_CWA86791.1

MKSKRMEFHNKFLIVSAMLAVISWLSLGVVSFPMLAGTLGISTKAAATVVNLISAYSTVTAVISIVGAITGVGSIGSGIAATVLYILKKKGAAKAALW

>Streptococcus-pneumoniae_CWI36424.1

MKSKRMEFHNKFLIVSAMLAVISWLSLGVVSFPMLAGTLGISTKAAATVVNLISAYSTVTAVISIVGAITGVGSIGSGIAATVLYILKKKGAAKAALW

>Streptococcus-pneumoniae_CWK28727.1

MKSKRMEFHNKFLIVSAMLAVISWLSLGVVSFPMLAGTLGISTKAAATVVNLISAYSTVTAVISIVGAITGVGSIGSGIAATVLYILKKKGAAKAALW

>Streptococcus-pneumoniae_CVY87750.1

MKSKRMEFHNKFLIVSAMLAVISWLSLGVVSFPMLAGTLGISTKAAATVVNLISAYSTVTAVISIVGAITGVGSIGSGIAATVLYILKKKGAAKAALW

>Streptococcus-pneumoniae_CWL58568.1

MKSKRMEFHNKFLIVSAMLAVISWLSLGVVSFPMLAGTLGISTKAAATVVNLISAYSTVTAVISIVGAITGVGSIGSGIAATVLYILKKKGAAKAALW

>Streptococcus-pneumoniae_CWJ49626.1

MKSKRMEFHNKFLIVSAMLAVISWLSLGVVSFPMLAGTLGISTKAAATVVNLISAYSTVTAVISIVGAITGVGSIGSGIAATVLYILKKKGAAKAALW

>Streptococcus-pneumoniae_CVY67860.1

MKSKRMEFHNKFLIVSAMLAVISWLSLGVVSFPMLAGTLGISTKAAATVVNLISAYSTVTAVISIVGAITGVGSIGSGIAATVLYILKKKGAAKAALW

>Streptococcus-pneumoniae_CWM40588.1

MKSKRMEFHNKFLIVSAMLAVISWLSLGVVSFPMLAGTLGISTKAAATVVNLISAYSTVTAVISIVGAITGVGSIGSGIAATVLYILKKKGAAKAALW

>Streptococcus-pneumoniae_CWK21705.1

MKSKRMEFHNKFLIVSAMLAVISWLSLGVVSFPMLAGTLGISTKAAATVVNLISAYSTVTAVISIVGAITGVGSIGSGIAATVLYILKKKGAAKAALW

>Streptococcus-pneumoniae_CWI00392.1

MKSKRMEFHNKFLIVSAMLAVISWLSLGVVSFPMLAGTLGISTKAAATVVNLISAYSTVTAVISIVGAITGVGSIGSGIAATVLYILKKKGAAKAALW

>Streptococcus-pneumoniae_CWF12574.1

MKSKRMEFHNKFLIVSAMLAVISWLSLGVVSFPMLAGTLGISTKAAATVVNLISAYSTVTAVISIVGAITGVGSIGSGIAATVLYILKKKGAAKAALW

>Streptococcus-pneumoniae_CWL38076.1

MKSKRMEFHNKFLIVSAMLAVISWLSLGVVSFPMLAGTLGISTKAAATVVNLISAYSTVTAVISIVGAITGVGSIGSGIAATVLYILKKKGAAKAALW

>Streptococcus-pneumoniae_CWI34924.1

MKSKRMEFHNKFLIVSAMLAVISWLSLGVVSFPMLAGTLGISTKAAATVVNLISAYSTVTAVISIVGAITGVGSIGSGIAATVLYILKKKGAAKAALW

>Streptococcus-pneumoniae_CWM00634.1

MKSKRMEFHNKFLIVSAMLAVISWLSLGVVSFPMLAGTLGISTKAAATVVNLISAYSTVTAVISIVGAITGVGSIGSGIAATVLYILKKKGAAKAALW

>Streptococcus-pneumoniae_CWE28820.1

MKSKRMEFHNKFLIVSAMLAVISWLSLGVVSFPMLAGTLGISTKAAATVVNLISAYSTVTAVISIVGAITGVGSIGSGIAATVLYILKKKGAAKAALW

>Streptococcus-pneumoniae_CWH06672.1

MKSKRMEFHNKFLIVSAMLAVISWLSLGVVSFPMLAGTLGISTKAAATVVNLISAYSTVTAVISIVGAITGVGSIGSGIAATVLYILKKKGAAKAALW

>Streptococcus-pneumoniae_CWL13486.1

MKSKRMEFHNKFLIVSAMLAVISWLSLGVVSFPMLAGTLGISTKAAATVVNLISAYSTVTAVISIVGAITGVGSIGSGIAATVLYILKKKGAAKAALW

>Streptococcus-pneumoniae_CVZ06985.1

MKSKRMEFHNKFLIVSAMLAVISWLSLGVVSFPMLAGTLGISTKAAATVVNLISAYSTVTAVISIVGAITGVGSIGSGIAATVLYILKKKGAAKAALW

>Streptococcus-pneumoniae_CWB20359.1

MKSKRMEFHNKFLIVSAMLAVISWLSLGVVSFPMLAGTLGISTKAAATVVNLISAYSTVTAVISIVGAITGVGSIGSGIAATVLYILKKKGAAKAALW

>Streptococcus-pneumoniae_CWJ43800.1

MKSKRMEFHNKFLIVSAMLAVISWLSLGVVSFPMLAGTLGISTKAAATVVNLISAYSTVTAVISIVGAITGVGSIGSGIAATVLYILKKKGAAKAALW

>Streptococcus-pneumoniae_CWK55819.1

MKSKRMEFHNKFLIVSAMLAVISWLSLGVVSFPMLAGTLGISTKAAATVVNLISAYSTVTAVISIVGAITGVGSIGSGIAATVLYILKKKGAAKAALW

>Streptococcus-pneumoniae_CWD15819.1

MKSKRMEFHNKFLIVSAMLAVISWLSLGVVSFPMLAGTLGISTKAAATVVNLISAYSTVTAVISIVGAITGVGSIGSGIAATVLYILKKKGAAKAALW

>Streptococcus-pneumoniae_CWE20999.1

MKSKRMEFHNKFLIVSAMLAVISWLSLGVVSFPMLAGTLGISTKAAATVVNLISAYSTVTAVISIVGAITGVGSIGSGIAATVLYILKKKGAAKAALW

>Streptococcus-pneumoniae_CWI03502.1

MKSKRMEFHNKFLIVSAMLAVISWLSLGVVSFPMLAGTLGISTKAAATVVNLISAYSTVTAVISIVGAITGVGSIGSGIAATVLYILKKKGAAKAALW

>Streptococcus-pneumoniae_CWH93432.1

MKSKRMEFHNKFLIVSAMLAVISWLSLGVVSFPMLAGTLGISTKAAATVVNLISAYSTVTAVISIVGAITGVGSIGSGIAATVLYILKKKGAAKAALW

>Streptococcus-pneumoniae_CWE60537.1

MKSKRMEFHNKFLIVSAMLAVISWLSLGVVSFPMLAGTLGISTKAAATVVNLISAYSTVTAVISIVGAITGVGSIGSGIAATVLYILKKKGAAKAALW

>Streptococcus-pneumoniae_CWC24989.1

MKSKRMEFHNKFLIVSAMLAVISWLSLGVVSFPMLAGTLGISTKAAATVVNLISAYSTVTAVISIVGAITGVGSIGSGIAATVLYILKKKGAAKAALW

>Streptococcus-pneumoniae_CWJ49753.1

MKSKRMEFHNKFLIVSAMLAVISWLSLGVVSFPMLAGTLGISTKAAATVVNLISAYSTVTAVISIVGAITGVGSIGSGIAATVLYILKKKGAAKAALW

>Streptococcus-pneumoniae_CWH30708.1

MKSKRMEFHNKFLIVSAMLAVISWLSLGVVSFPMLAGTLGISTKAAATVVNLISAYSTVTAVISIVGAITGVGSIGSGIAATVLYILKKKGAAKAALW

>Streptococcus-pneumoniae_CWK50275.1

MKSKRMEFHNKFLIVSAMLAVISWLSLGVVSFPMLAGTLGISTKAAATVVNLISAYSTVTAVISIVGAITGVGSIGSGIAATVLYILKKKGAAKAALW

>Streptococcus-pneumoniae_CWA62026.1

MKSKRMEFHNKFLIVSAMLAVISWLSLGVVSFPMLAGTLGISTKAAATVVNLISAYSTVTAVISIVGAITGVGSIGSGIAATVLYILKKKGAAKAALW

>Streptococcus-pneumoniae_CVZ81553.1

MKSKRMEFHNKFLIVSAMLAVISWLSLGVVSFPMLAGTLGISTKAAATVVNLISAYSTVTAVISIVGAITGVGSIGSGIAATVLYILKKKGAAKAALW

>Streptococcus-pneumoniae_CWJ09344.1

MKSKRMEFHNKFLIVSAMLAVISWLSLGVVSFPMLAGTLGISTKAAATVVNLISAYSTVTAVISIVGAITGVGSIGSGIAATVLYILKKKGAAKAALW

>Streptococcus-pneumoniae_CWB56134.1

MKSKRMEFHNKFLIVSAMLAVISWLSLGVVSFPMLAGTLGISTKAAATVVNLISAYSTVTAVISIVGAITGVGSIGSGIAATVLYILKKKGAAKAALW

>Streptococcus-pneumoniae_CVZ95236.1

MKSKRMEFHNKFLIVSAMLAVISWLSLGVVSFPMLAGTLGISTKAAATVVNLISAYSTVTAVISIVGAITGVGSIGSGIAATVLYILKKKGAAKAALW

>Streptococcus-pneumoniae_CWA45421.1

MKSKRMEFHNKFLIVSAMLAVISWLSLGVVSFPMLAGTLGISTKAAATVVNLISAYSTVTAVISIVGAITGVGSIGSGIAATVLYILKKKGAAKAALW

>Streptococcus-pneumoniae_CWI62670.1

MKSKRMEFHNKFLIVSAMLAVISWLSLGVVSFPMLAGTLGISTKAAATVVNLISAYSTVTAVISIVGAITGVGSIGSGIAATVLYILKKKGAAKAALW

>Streptococcus-pneumoniae_CWF94526.1

MKSKRMEFHNKFLIVSAMLAVISWLSLGVVSFPMLAGTLGISTKAAATVVNLISAYSTVTAVISIVGAITGVGSIGSGIAATVLYILKKKGAAKAALW

>Streptococcus-pneumoniae_CWA96172.1

MKSKRMEFHNKFLIVSAMLAVISWLSLGVVSFPMLAGTLGISTKAAATVVNLISAYSTVTAVISIVGAITGVGSIGSGIAATVLYILKKKGAAKAALW

>Streptococcus-pneumoniae_CWB76400.1

MKSKRMEFHNKFLIVSAMLAVISWLSLGVVSFPMLAGTLGISTKAAATVVNLISAYSTVTAVISIVGAITGVGSIGSGIAATVLYILKKKGAAKAALW

>Streptococcus-pneumoniae_CWI63958.1

MKSKRMEFHNKFLIVSAMLAVISWLSLGVVSFPMLAGTLGISTKAAATVVNLISAYSTVTAVISIVGAITGVGSIGSGIAATVLYILKKKGAAKAALW

>Streptococcus-pneumoniae_CWB80596.1

MKSKRMEFHNKFLIVSAMLAVISWLSLGVVSFPMLAGTLGISTKAAATVVNLISAYSTVTAVISIVGAITGVGSIGSGIAATVLYILKKKGAAKAALW

>Streptococcus-pneumoniae_CWB88394.1

MKSKRMEFHNKFLIVSAMLAVISWLSLGVVSFPMLAGTLGISTKAAATVVNLISAYSTVTAVISIVGAITGVGSIGSGIAATVLYILKKKGAAKAALW

>Streptococcus-pneumoniae_CWK05799.1

MKSKRMEFHNKFLIVSAMLAVISWLSLGVVSFPMLAGTLGISTKAAATVVNLISAYSTVTAVISIVGAITGVGSIGSGIAATVLYILKKKGAAKAALW

>Streptococcus-pneumoniae_CWB28904.1

MKSKRMEFHNKFLIVSAMLAVISWLSLGVVSFPMLAGTLGISTKAAATVVNLISAYSTVTAVISIVGAITGVGSIGSGIAATVLYILKKKGAAKAALW

>Streptococcus-pneumoniae_CWA75686.1

MKSKRMEFHNKFLIVSAMLAVISWLSLGVVSFPMLAGTLGISTKAAATVVNLISAYSTVTAVISIVGAITGVGSIGSGIAATVLYILKKKGAAKAALW

>Streptococcus-pneumoniae_CWM27834.1

MKSKRMEFHNKFLIVSAMLAVISWLSLGVVSFPMLAGTLGISTKAAATVVNLISAYSTVTAVISIVGAITGVGSIGSGIAATVLYILKKKGAAKAALW

>Streptococcus-pneumoniae_CWA80755.1

MKSKRMEFHNKFLIVSAMLAVISWLSLGVVSFPMLAGTLGISTKAAATVVNLISAYSTVTAVISIVGAITGVGSIGSGIAATVLYILKKKGAAKAALW

>Streptococcus-pneumoniae_CVZ89171.1

MKSKRMEFHNKFLIVSAMLAVISWLSLGVVSFPMLAGTLGISTKAAATVVNLISAYSTVTAVISIVGAITGVGSIGSGIAATVLYILKKKGAAKAALW

>Streptococcus-pneumoniae_CWH06463.1

MKSKRMEFHNKFLIVSAMLAVISWLSLGVVSFPMLAGTLGISTKAAATVVNLISAYSTVTAVISIVGAITGVGSIGSGIAATVLYILKKKGAAKAALW

>Streptococcus-pneumoniae_CWF85015.1

MKSKRMEFHNKFLIVSAMLAVISWLSLGVVSFPMLAGTLGISTKAAATVVNLISAYSTVTAVISIVGAITGVGSIGSGIAATVLYILKKKGAAKAALW

>Streptococcus-pneumoniae_CWB24843.1

MKSKRMEFHNKFLIVSAMLAVISWLSLGVVSFPMLAGTLGISTKAAATVVNLISAYSTVTAVISIVGAITGVGSIGSGIAATVLYILKKKGAAKAALW

>Streptococcus-pneumoniae_CWI76685.1

MKSKRMEFHNKFLIVSAMLAVISWLSLGVVSFPMLAGTLGISTKAAATVVNLISAYSTVTAVISIVGAITGVGSIGSGIAATVLYILKKKGAAKAALW

>Streptococcus-pneumoniae_CVZ73542.1

MKSKRMEFHNKFLIVSAMLAVISWLSLGVVSFPMLAGTLGISTKAAATVVNLISAYSTVTAVISIVGAITGVGSIGSGIAATVLYILKKKGAAKAALW

>Streptococcus-pneumoniae_CWK77031.1

MKSKRMEFHNKFLIVSAMLAVISWLSLGVVSFPMLAGTLGISTKAAATVVNLISAYSTVTAVISIVGAITGVGSIGSGIAATVLYILKKKGAAKAALW

>Streptococcus-pneumoniae_CWC45623.1

MKSKRMEFHNKFLIVSAMLAVISWLSLGVVSFPMLAGTLGISTKAAATVVNLISAYSTVTAVISIVGAITGVGSIGSGIAATVLYILKKKGAAKAALW

>Streptococcus-pneumoniae_CVY90421.1

MKSKRMEFHNKFLIVSAMLAVISWLSLGVVSFPMLAGTLGISTKAAATVVNLISAYSTVTAVISIVGAITGVGSIGSGIAATVLYILKKKGAAKAALW

>Streptococcus-pneumoniae_CWM18953.1

MKSKRMEFHNKFLIVSAMLAVISWLSLGVVSFPMLAGTLGISTKAAATVVNLISAYSTVTAVISIVGAITGVGSIGSGIAATVLYILKKKGAAKAALW

>Streptococcus-pneumoniae_CWJ89830.1

MKSKRMEFHNKFLIVSAMLAVISWLSLGVVSFPMLAGTLGISTKAAATVVNLISAYSTVTAVISIVGAITGVGSIGSGIAATVLYILKKKGAAKAALW

>Streptococcus-pneumoniae_CWD34420.1

MKSKRMEFHNKFLIVSAMLAVISWLSLGVVSFPMLAGTLGISTKAAATVVNLISAYSTVTAVISIVGAITGVGSIGSGIAATVLYILKKKGAAKAALW

>Streptococcus-pneumoniae_CWC79453.1

MKSKRMEFHNKFLIVSAMLAVISWLSLGVVSFPMLAGTLGISTKAAATVVNLISAYSTVTAVISIVGAITGVGSIGSGIAATVLYILKKKGAAKAALW

>Streptococcus-pneumoniae_CVZ89959.1

MKSKRMEFHNKFLIVSAMLAVISWLSLGVVSFPMLAGTLGISTKAAATVVNLISAYSTVTAVISIVGAITGVGSIGSGIAATVLYILKKKGAAKAALW

>Streptococcus-pneumoniae_CWL85912.1

MKSKRMEFHNKFLIVSAMLAVISWLSLGVVSFPMLAGTLGISTKAAATVVNLISAYSTVTAVISIVGAITGVGSIGSGIAATVLYILKKKGAAKAALW

>Streptococcus-pneumoniae_CWK33478.1

MKSKRMEFHNKFLIVSAMLAVISWLSLGVVSFPMLAGTLGISTKAAATVVNLISAYSTVTAVISIVGAITGVGSIGSGIAATVLYILKKKGAAKAALW

>Streptococcus-pneumoniae_CWK20575.1

MKSKRMEFHNKFLIVSAMLAVISWLSLGVVSFPMLAGTLGISTKAAATVVNLISAYSTVTAVISIVGAITGVGSIGSGIAATVLYILKKKGAAKAALW

>Streptococcus-pneumoniae_CWB19675.1

MKSKRMEFHNKFLIVSAMLAVISWLSLGVVSFPMLAGTLGISTKAAATVVNLISAYSTVTAVISIVGAITGVGSIGSGIAATVLYILKKKGAAKAALW

>Streptococcus-pneumoniae_CVZ32693.1

MKSKRMEFHNKFLIVSAMLAVISWLSLGVVSFPMLAGTLGISTKAAATVVNLISAYSTVTAVISIVGAITGVGSIGSGIAATVLYILKKKGAAKAALW

>Streptococcus-pneumoniae_CWJ18773.1

MKSKRMEFHNKFLIVSAMLAVISWLSLGVVSFPMLAGTLGISTKAAATVVNLISAYSTVTAVISIVGAITGVGSIGSGIAATVLYILKKKGAAKAALW

>Streptococcus-pneumoniae_CWI08642.1

MKSKRMEFHNKFLIVSAMLAVISWLSLGVVSFPMLAGTLGISTKAAATVVNLISAYSTVTAVISIVGAITGVGSIGSGIAATVLYILKKKGAAKAALW

>Streptococcus-pneumoniae_CWB93451.1

MKSKRMEFHNKFLIVSAMLAVISWLSLGVVSFPMLAGTLGISTKAAATVVNLISAYSTVTAVISIVGAITGVGSIGSGIAATVLYILKKKGAAKAALW

>Streptococcus-pneumoniae_CWC36149.1

MKSKRMEFHNKFLIVSAMLAVISWLSLGVVSFPMLAGTLGISTKAAATVVNLISAYSTVTAVISIVGAITGVGSIGSGIAATVLYILKKKGAAKAALW

>Streptococcus-pneumoniae_CWD27569.1

MKSKRMEFHNKFLIVSAMLAVISWLSLGVVSFPMLAGTLGISTKAAATVVNLISAYSTVTAVISIVGAITGVGSIGSGIAATVLYILKKKGAAKAALW

>Streptococcus-pneumoniae_CWA86342.1

MKSKRMEFHNKFLIVSAMLAVISWLSLGVVSFPMLAGTLGISTKAAATVVNLISAYSTVTAVISIVGAITGVGSIGSGIAATVLYILKKKGAAKAALW

>Streptococcus-pneumoniae_CWB28993.1

MKSKRMEFHNKFLIVSAMLAVISWLSLGVVSFPMLAGTLGISTKAAATVVNLISAYSTVTAVISIVGAITGVGSIGSGIAATVLYILKKKGAAKAALW

>Streptococcus-pneumoniae_CWE51965.1

MKSKRMEFHNKFLIVSAMLAVISWLSLGVVSFPMLAGTLGISTKAAATVVNLISAYSTVTAVISIVGAITGVGSIGSGIAATVLYILKKKGAAKAALW

>Streptococcus-pneumoniae_CWA60705.1

MKSKRMEFHNKFLIVSAMLAVISWLSLGVVSFPMLAGTLGISTKAAATVVNLISAYSTVTAVISIVGAITGVGSIGSGIAATVLYILKKKGAAKAALW

>Streptococcus-pneumoniae_CWA09329.1

MKSKRMEFHNKFLIVSAMLAVISWLSLGVVSFPMLAGTLGISTKAAATVVNLISAYSTVTAVISIVGAITGVGSIGSGIAATVLYILKKKGAAKAALW

>Streptococcus-pneumoniae_CWH06350.1

MKSKRMEFHNKFLIVSAMLAVISWLSLGVVSFPMLAGTLGISTKAAATVVNLISAYSTVTAVISIVGAITGVGSIGSGIAATVLYILKKKGAAKAALW

>Streptococcus-pneumoniae_CWA32146.1

MKSKRMEFHNKFLIVSAMLAVISWLSLGVVSFPMLAGTLGISTKAAATVVNLISAYSTVTAVISIVGAITGVGSIGSGIAATVLYILKKKGAAKAALW

>Streptococcus-pneumoniae_CWG56885.1

MKSKRMEFHNKFLIVSAMLAVISWLSLGVVSFPMLAGTLGISTKAAATVVNLISAYSTVTAVISIVGAITGVGSIGSGIAATVLYILKKKGAAKAALW

>Streptococcus-pneumoniae_CWH58416.1

MKSKRMEFHNKFLIVSAMLAVISWLSLGVVSFPMLAGTLGISTKAAATVVNLISAYSTVTAVISIVGAITGVGSIGSGIAATVLYILKKKGAAKAALW

>Streptococcus-pneumoniae_CWH26166.1

MKSKRMEFHNKFLIVSAMLAVISWLSLGVVSFPMLAGTLGISTKAAATVVNLISAYSTVTAVISIVGAITGVGSIGSGIAATVLYILKKKGAAKAALW

>Streptococcus-pneumoniae_CWD84059.1

MKSKRMEFHNKFLIVSAMLAVISWLSLGVVSFPMLAGTLGISTKAAATVVNLISAYSTVTAVISIVGAITGVGSIGSGIAATVLYILKKKGAAKAALW

>Streptococcus-pneumoniae_CVZ43338.1

MKSKRMEFHNKFLIVSAMLAVISWLSLGVVSFPMLAGTLGISTKAAATVVNLISAYSTVTAVISIVGAITGVGSIGSGIAATVLYILKKKGAAKAALW

>Streptococcus-pneumoniae_CWE75829.1

MKSKRMEFHNKFLIVSAMLAVISWLSLGVVSFPMLAGTLGISTKAAATVVNLISAYSTVTAVISIVGAITGVGSIGSGIAATVLYILKKKGAAKAALW

>Streptococcus-pneumoniae_CWH89988.1

MKSKRMEFHNKFLIVSAMLAVISWLSLGVVSFPMLAGTLGISTKAAATVVNLISAYSTVTAVISIVGAITGVGSIGSGIAATVLYILKKKGAAKAALW

>Streptococcus-pneumoniae_CWM01652.1

MKSKRMEFHNKFLIVSAMLAVISWLSLGVVSFPMLAGTLGISTKAAATVVNLISAYSTVTAVISIVGAITGVGSIGSGIAATVLYILKKKGAAKAALW

>Streptococcus-pneumoniae_CWA56916.1

MKSKRMEFHNKFLIVSAMLAVISWLSLGVVSFPMLAGTLGISTKAAATVVNLISAYSTVTAVISIVGAITGVGSIGSGIAATVLYILKKKGAAKAALW

>Streptococcus-pneumoniae_CWJ03654.1

MKSKRMEFHNKFLIVSAMLAVISWLSLGVVSFPMLAGTLGISTKAAATVVNLISAYSTVTAVISIVGAITGVGSIGSGIAATVLYILKKKGAAKAALW

>Streptococcus-pneumoniae_CVY92893.1

MKSKRMEFHNKFLIVSAMLAVISWLSLGVVSFPMLAGTLGISTKAAATVVNLISAYSTVTAVISIVGAITGVGSIGSGIAATVLYILKKKGAAKAALW

>Streptococcus-pneumoniae_CVZ99829.1

MKSKRMEFHNKFLIVSAMLAVISWLSLGVVSFPMLAGTLGISTKAAATVVNLISAYSTVTAVISIVGAITGVGSIGSGIAATVLYILKKKGAAKAALW

>Streptococcus-pneumoniae_CWG03015.1

MKSKRMEFHNKFLIVSAMLAVISWLSLGVVSFPMLAGTLGISTKAAATVVNLISAYSTVTAVISIVGAITGVGSIGSGIAATVLYILKKKGAAKAALW

>Streptococcus-pneumoniae_CWJ15745.1

MKSKRMEFHNKFLIVSAMLAVISWLSLGVVSFPMLAGTLGISTKAAATVVNLISAYSTVTAVISIVGAITGVGSIGSGIAATVLYILKKKGAAKAALW

>Streptococcus-pneumoniae_CWB54947.1

MKSKRMEFHNKFLIVSAMLAVISWLSLGVVSFPMLAGTLGISTKAAATVVNLISAYSTVTAVISIVGAITGVGSIGSGIAATVLYILKKKGAAKAALW

>Streptococcus-pneumoniae_CWK43780.1

MKSKRMEFHNKFLIVSAMLAVISWLSLGVVSFPMLAGTLGISTKAAATVVNLISAYSTVTAVISIVGAITGVGSIGSGIAATVLYILKKKGAAKAALW

>Streptococcus-pneumoniae_CVZ92003.1

MKSKRMEFHNKFLIVSAMLAVISWLSLGVVSFPMLAGTLGISTKAAATVVNLISAYSTVTAVISIVGAITGVGSIGSGIAATVLYILKKKGAAKAALW

>Streptococcus-pneumoniae_CWL54850.1

MKSKRMEFHNKFLIVSAMLAVISWLSLGVVSFPMLAGTLGISTKAAATVVNLISAYSTVTAVISIVGAITGVGSIGSGIAATVLYILKKKGAAKAALW

>Streptococcus-pneumoniae_CWG21562.1

MKSKRMEFHNKFLIVSAMLAVISWLSLGVVSFPMLAGTLGISTKAAATVVNLISAYSTVTAVISIVGAITGVGSIGSGIAATVLYILKKKGAAKAALW

>Streptococcus-pneumoniae_CVY32375.1

MKSKRMEFHNKFLIVSAMLAVISWLSLGVVSFPMLAGTLGISTKAAATVVNLISAYSTVTAVISIVGAITGVGSIGSGIAATVLYILKKKGAAKAALW

>Streptococcus-pneumoniae_CVY44670.1

MKSKRMEFHNKFLIVSAMLAVISWLSLGVVSFPMLAGTLGISTKAAATVVNLISAYSTVTAVISIVGAITGVGSIGSGIAATVLYILKKKGAAKAALW

>Streptococcus-pneumoniae_CWM22127.1

MKSKRMEFHNKFLIVSAMLAVISWLSLGVVSFPMLAGTLGISTKAAATVVNLISAYSTVTAVISIVGAITGVGSIGSGIAATVLYILKKKGAAKAALW

>Streptococcus-pneumoniae_CWH17126.1

MKSKRMEFHNKFLIVSAMLAVISWLSLGVVSFPMLAGTLGISTKAAATVVNLISAYSTVTAVISIVGAITGVGSIGSGIAATVLYILKKKGAAKAALW

>Streptococcus-pneumoniae_CWC03999.1

MKSKRMEFHNKFLIVSAMLAVISWLSLGVVSFPMLAGTLGISTKAAATVVNLISAYSTVTAVISIVGAITGVGSIGSGIAATVLYILKKKGAAKAALW

>Streptococcus-pneumoniae_CWC73194.1

MKSKRMEFHNKFLIVSAMLAVISWLSLGVVSFPMLAGTLGISTKAAATVVNLISAYSTVTAVISIVGAITGVGSIGSGIAATVLYILKKKGAAKAALW

>Streptococcus-pneumoniae_CWD21195.1

MKSKRMEFHNKFLIVSAMLAVISWLSLGVVSFPMLAGTLGISTKAAATVVNLISAYSTVTAVISIVGAITGVGSIGSGIAATVLYILKKKGAAKAALW

>Streptococcus-pneumoniae_CWI30025.1

MKSKRMEFHNKFLIVSAMLAVISWLSLGVVSFPMLAGTLGISTKAAATVVNLISAYSTVTAVISIVGAITGVGSIGSGIAATVLYILKKKGAAKAALW

>Streptococcus-pneumoniae_CWK74388.1

MKSKRMEFHNKFLIVSAMLAVISWLSLGVVSFPMLAGTLGISTKAAATVVNLISAYSTVTAVISIVGAITGVGSIGSGIAATVLYILKKKGAAKAALW

>Streptococcus-pneumoniae_CWI80075.1

MKSKRMEFHNKFLIVSAMLAVISWLSLGVVSFPMLAGTLGISTKAAATVVNLISAYSTVTAVISIVGAITGVGSIGSGIAATVLYILKKKGAAKAALW

>Streptococcus-pneumoniae_CWG35366.1

MKSKRMEFHNKFLIVSAMLAVISWLSLGVVSFPMLAGTLGISTKAAATVVNLISAYSTVTAVISIVGAITGVGSIGSGIAATVLYILKKKGAAKAALW

>Streptococcus-pneumoniae_CWK07760.1

MKSKRMEFHNKFLIVSAMLAVISWLSLGVVSFPMLAGTLGISTKAAATVVNLISAYSTVTAVISIVGAITGVGSIGSGIAATVLYILKKKGAAKAALW

>Streptococcus-pneumoniae_CWK05971.1

MKSKRMEFHNKFLIVSAMLAVISWLSLGVVSFPMLAGTLGISTKAAATVVNLISAYSTVTAVISIVGAITGVGSIGSGIAATVLYILKKKGAAKAALW

>Streptococcus-pneumoniae_CWB39789.1

MKSKRMEFHNKFLIVSAMLAVISWLSLGVVSFPMLAGTLGISTKAAATVVNLISAYSTVTAVISIVGAITGVGSIGSGIAATVLYILKKKGAAKAALW

>Streptococcus-pneumoniae_CWA82845.1

MKSKRMEFHNKFLIVSAMLAVISWLSLGVVSFPMLAGTLGISTKAAATVVNLISAYSTVTAVISIVGAITGVGSIGSGIAATVLYILKKKGAAKAALW

>Streptococcus-pneumoniae_CWC48485.1

MKSKRMEFHNKFLIVSAMLAVISWLSLGVVSFPMLAGTLGISTKAAATVVNLISAYSTVTAVISIVGAITGVGSIGSGIAATVLYILKKKGAAKAALW

>Streptococcus-pneumoniae_CWK33606.1

MKSKRMEFHNKFLIVSAMLAVISWLSLGVVSFPMLAGTLGISTKAAATVVNLISAYSTVTAVISIVGAITGVGSIGSGIAATVLYILKKKGAAKAALW

>Streptococcus-pneumoniae_CWJ11327.1

MKSKRMEFHNKFLIVSAMLAVISWLSLGVVSFPMLAGTLGISTKAAATVVNLISAYSTVTAVISIVGAITGVGSIGSGIAATVLYILKKKGAAKAALW

>Streptococcus-pneumoniae_CVZ04832.1

MKSKRMEFHNKFLIVSAMLAVISWLSLGVVSFPMLAGTLGISTKAAATVVNLISAYSTVTAVISIVGAITGVGSIGSGIAATVLYILKKKGAAKAALW

>Streptococcus-pneumoniae_CWL84958.1

MKSKRMEFHNKFLIVSAMLAVISWLSLGVVSFPMLAGTLGISTKAAATVVNLISAYSTVTAVISIVGAITGVGSIGSGIAATVLYILKKKGAAKAALW

>Streptococcus-pneumoniae_CWB33187.1

MKSKRMEFHNKFLIVSAMLAVISWLSLGVVSFPMLAGTLGISTKAAATVVNLISAYSTVTAVISIVGAITGVGSIGSGIAATVLYILKKKGAAKAALW

>Streptococcus-pneumoniae_CWD45675.1

MKSKRMEFHNKFLIVSAMLAVISWLSLGVVSFPMLAGTLGISTKAAATVVNLISAYSTVTAVISIVGAITGVGSIGSGIAATVLYILKKKGAAKAALW

>Streptococcus-pneumoniae_CWB79087.1

MKSKRMEFHNKFLIVSAMLAVISWLSLGVVSFPMLAGTLGISTKAAATVVNLISAYSTVTAVISIVGAITGVGSIGSGIAATVLYILKKKGAAKAALW

>Streptococcus-pneumoniae_CVZ24809.1

MKSKRMEFHNKFLIVSAMLAVISWLSLGVVSFPMLAGTLGISTKAAATVVNLISAYSTVTAVISIVGAITGVGSIGSGIAATVLYILKKKGAAKAALW

>Streptococcus-pneumoniae_CWE48903.1

MKSKRMEFHNKFLIVSAMLAVISWLSLGVVSFPMLAGTLGISTKAAATVVNLISAYSTVTAVISIVGAITGVGSIGSGIAATVLYILKKKGAAKAALW

>Streptococcus-pneumoniae_CWF51844.1

MKSKRMEFHNKFLIVSAMLAVISWLSLGVVSFPMLAGTLGISTKAAATVVNLISAYSTVTAVISIVGAITGVGSIGSGIAATVLYILKKKGAAKAALW

>Streptococcus-pneumoniae_CVZ29385.1

MKSKRMEFHNKFLIVSAMLAVISWLSLGVVSFPMLAGTLGISTKAAATVVNLISAYSTVTAVISIVGAITGVGSIGSGIAATVLYILKKKGAAKAALW

>Streptococcus-pneumoniae_CWA80021.1

MKSKRMEFHNKFLIVSAMLAVISWLSLGVVSFPMLAGTLGISTKAAATVVNLISAYSTVTAVISIVGAITGVGSIGSGIAATVLYILKKKGAAKAALW

>Streptococcus-pneumoniae_CVZ23206.1

MKSKRMEFHNKFLIVSAMLAVISWLSLGVVSFPMLAGTLGISTKAAATVVNLISAYSTVTAVISIVGAITGVGSIGSGIAATVLYILKKKGAAKAALW

>Streptococcus-pneumoniae_CWE20990.1

MKSKRMEFHNKFLIVSAMLAVISWLSLGVVSFPMLAGTLGISTKAAATVVNLISAYSTVTAVISIVGAITGVGSIGSGIAATVLYILKKKGAAKAALW

>Streptococcus-pneumoniae_CWL96101.1

MKSKRMEFHNKFLIVSAMLAVISWLSLGVVSFPMLAGTLGISTKAAATVVNLISAYSTVTAVISIVGAITGVGSIGSGIAATVLYILKKKGAAKAALW

>Streptococcus-pneumoniae_CWL35807.1

MKSKRMEFHNKFLIVSAMLAVISWLSLGVVSFPMLAGTLGISTKAAATVVNLISAYSTVTAVISIVGAITGVGSIGSGIAATVLYILKKKGAAKAALW

>Streptococcus-pneumoniae_CWB29324.1

MKSKRMEFHNKFLIVSAMLAVISWLSLGVVSFPMLAGTLGISTKAAATVVNLISAYSTVTAVISIVGAITGVGSIGSGIAATVLYILKKKGAAKAALW

>Streptococcus-pneumoniae_CWL37561.1

MKSKRMEFHNKFLIVSAMLAVISWLSLGVVSFPMLAGTLGISTKAAATVVNLISAYSTVTAVISIVGAITGVGSIGSGIAATVLYILKKKGAAKAALW

>Streptococcus-pneumoniae_CWH11656.1

MKSKRMEFHNKFLIVSAMLAVISWLSLGVVSFPMLAGTLGISTKAAATVVNLISAYSTVTAVISIVGAITGVGSIGSGIAATVLYILKKKGAAKAALW

>Streptococcus-pneumoniae_CWC17468.1

MKSKRMEFHNKFLIVSAMLAVISWLSLGVVSFPMLAGTLGISTKAAATVVNLISAYSTVTAVISIVGAITGVGSIGSGIAATVLYILKKKGAAKAALW

>Streptococcus-pneumoniae_CWD31021.1

MKSKRMEFHNKFLIVSAMLAVISWLSLGVVSFPMLAGTLGISTKAAATVVNLISAYSTVTAVISIVGAITGVGSIGSGIAATVLYILKKKGAAKAALW

>Streptococcus-pneumoniae_CWI49661.1

MKSKRMEFHNKFLIVSAMLAVISWLSLGVVSFPMLAGTLGISTKAAATVVNLISAYSTVTAVISIVGAITGVGSIGSGIAATVLYILKKKGAAKAALW

>Streptococcus-pneumoniae_CWD72329.1

MKSKRMEFHNKFLIVSAMLAVISWLSLGVVSFPMLAGTLGISTKAAATVVNLISAYSTVTAVISIVGAITGVGSIGSGIAATVLYILKKKGAAKAALW

>Streptococcus-pneumoniae_CWK38207.1

MKSKRMEFHNKFLIVSAMLAVISWLSLGVVSFPMLAGTLGISTKAAATVVNLISAYSTVTAVISIVGAITGVGSIGSGIAATVLYILKKKGAAKAALW

>Streptococcus-pneumoniae_CWL20726.1

MKSKRMEFHNKFLIVSAMLAVISWLSLGVVSFPMLAGTLGISTKAAATVVNLISAYSTVTAVISIVGAITGVGSIGSGIAATVLYILKKKGAAKAALW

>Streptococcus-pneumoniae_CWG22818.1

MKSKRMEFHNKFLIVSAMLAVISWLSLGVVSFPMLAGTLGISTKAAATVVNLISAYSTVTAVISIVGAITGVGSIGSGIAATVLYILKKKGAAKAALW

>Streptococcus-pneumoniae_CVZ19409.1

MKSKRMEFHNKFLIVSAMLAVISWLSLGVVSFPMLAGTLGISTKAAATVVNLISAYSTVTAVISIVGAITGVGSIGSGIAATVLYILKKKGAAKAALW

>Streptococcus-pneumoniae_CWE30566.1

MKSKRMEFHNKFLIVSAMLAVISWLSLGVVSFPMLAGTLGISTKAAATVVNLISAYSTVTAVISIVGAITGVGSIGSGIAATVLYILKKKGAAKAALW

>Streptococcus-pneumoniae_CWJ66480.1

MKSKRMEFHNKFLIVSAMLAVISWLSLGVVSFPMLAGTLGISTKAAATVVNLISAYSTVTAVISIVGAITGVGSIGSGIAATVLYILKKKGAAKAALW

>Streptococcus-pneumoniae_CVZ73939.1

MKSKRMEFHNKFLIVSAMLAVISWLSLGVVSFPMLAGTLGISTKAAATVVNLISAYSTVTAVISIVGAITGVGSIGSGIAATVLYILKKKGAAKAALW

>Streptococcus-pneumoniae_CWA16662.1

MKSKRMEFHNKFLIVSAMLAVISWLSLGVVSFPMLAGTLGISTKAAATVVNLISAYSTVTAVISIVGAITGVGSIGSGIAATVLYILKKKGAAKAALW

>Streptococcus-pneumoniae_CWD22071.1

MKSKRMEFHNKFLIVSAMLAVISWLSLGVVSFPMLAGTLGISTKAAATVVNLISAYSTVTAVISIVGAITGVGSIGSGIAATVLYILKKKGAAKAALW

>Streptococcus-pneumoniae_CWH90714.1

MKSKRMEFHNKFLIVSAMLAVISWLSLGVVSFPMLAGTLGISTKAAATVVNLISAYSTVTAVISIVGAITGVGSIGSGIAATVLYILKKKGAAKAALW

>Streptococcus-pneumoniae_CWD07828.1

MKSKRMEFHNKFLIVSAMLAVISWLSLGVVSFPMLAGTLGISTKAAATVVNLISAYSTVTAVISIVGAITGVGSIGSGIAATVLYILKKKGAAKAALW

>Streptococcus-pneumoniae_CWD96923.1

MKSKRMEFHNKFLIVSAMLAVISWLSLGVVSFPMLAGTLGISTKAAATVVNLISAYSTVTAVISIVGAITGVGSIGSGIAATVLYILKKKGAAKAALW

>Streptococcus-pneumoniae_CVZ17637.1

MKSKRMEFHNKFLIVSAMLAVISWLSLGVVSFPMLAGTLGISTKAAATVVNLISAYSTVTAVISIVGAITGVGSIGSGIAATVLYILKKKGAAKAALW

>Streptococcus-pneumoniae_CWL15408.1

MKSKRMEFHNKFLIVSAMLAVISWLSLGVVSFPMLAGTLGISTKAAATVVNLISAYSTVTAVISIVGAITGVGSIGSGIAATVLYILKKKGAAKAALW

>Streptococcus-pneumoniae_CVZ01400.1

MKSKRMEFHNKFLIVSAMLAVISWLSLGVVSFPMLAGTLGISTKAAATVVNLISAYSTVTAVISIVGAITGVGSIGSGIAATVLYILKKKGAAKAALW

>Streptococcus-pneumoniae_CWB26497.1

MKSKRMEFHNKFLIVSAMLAVISWLSLGVVSFPMLAGTLGISTKAAATVVNLISAYSTVTAVISIVGAITGVGSIGSGIAATVLYILKKKGAAKAALW

>Streptococcus-pneumoniae_CWB41298.1

MKSKRMEFHNKFLIVSAMLAVISWLSLGVVSFPMLAGTLGISTKAAATVVNLISAYSTVTAVISIVGAITGVGSIGSGIAATVLYILKKKGAAKAALW

>Streptococcus-pneumoniae_CWB45764.1

MKSKRMEFHNKFLIVSAMLAVISWLSLGVVSFPMLAGTLGISTKAAATVVNLISAYSTVTAVISIVGAITGVGSIGSGIAATVLYILKKKGAAKAALW

>Streptococcus-pneumoniae_CWA52436.1

MKSKRMEFHNKFLIVSAMLAVISWLSLGVVSFPMLAGTLGISTKAAATVVNLISAYSTVTAVISIVGAITGVGSIGSGIAATVLYILKKKGAAKAALW

>Streptococcus-pneumoniae_CWD66409.1

MKSKRMEFHNKFLIVSAMLAVISWLSLGVVSFPMLAGTLGISTKAAATVVNLISAYSTVTAVISIVGAITGVGSIGSGIAATVLYILKKKGAAKAALW

>Streptococcus-pneumoniae_CWD30908.1

MKSKRMEFHNKFLIVSAMLAVISWLSLGVVSFPMLAGTLGISTKAAATVVNLISAYSTVTAVISIVGAITGVGSIGSGIAATVLYILKKKGAAKAALW

>Streptococcus-pneumoniae_CWA33188.1

MKSKRMEFHNKFLIVSAMLAVISWLSLGVVSFPMLAGTLGISTKAAATVVNLISAYSTVTAVISIVGAITGVGSIGSGIAATVLYILKKKGAAKAALW

>Streptococcus-pneumoniae_CWE08071.1

MKSKRMEFHNKFLIVSAMLAVISWLSLGVVSFPMLAGTLGISTKAAATVVNLISAYSTVTAVISIVGAITGVGSIGSGIAATVLYILKKKGAAKAALW

>Streptococcus-pneumoniae_CWC79499.1

MKSKRMEFHNKFLIVSAMLAVISWLSLGVVSFPMLAGTLGISTKAAATVVNLISAYSTVTAVISIVGAITGVGSIGSGIAATVLYILKKKGAAKAALW

>Streptococcus-pneumoniae_CWL30693.1

MKSKRMEFHNKFLIVSAMLAVISWLSLGVVSFPMLAGTLGISTKAAATVVNLISAYSTVTAVISIVGAITGVGSIGSGIAATVLYILKKKGAAKAALW

>Streptococcus-pneumoniae_CWF44823.1

MKSKRMEFHNKFLIVSAMLAVISWLSLGVVSFPMLAGTLGISTKAAATVVNLISAYSTVTAVISIVGAITGVGSIGSGIAATVLYILKKKGAAKAALW

>Streptococcus-pneumoniae_CWL71186.1

MKSKRMEFHNKFLIVSAMLAVISWLSLGVVSFPMLAGTLGISTKAAATVVNLISAYSTVTAVISIVGAITGVGSIGSGIAATVLYILKKKGAAKAALW

>Streptococcus-pneumoniae_CXF27522.1

MKSKRMEFHNKFLIVSAMLAVISWLSLGVVSFPMLAGTLGISTKAAATVVNLISAYSTVTAVISIVGAITGVGSIGSGIAATVLYILKKKGAAKAALW

>Streptococcus-pneumoniae_CWG53341.1

MKSKRMEFHNKFLIVSAMLAVISWLSLGVVSFPMLAGTLGISTKAAATVVNLISAYSTVTAVISIVGAITGVGSIGSGIAATVLYILKKKGAAKAALW

>Streptococcus-pneumoniae_CWE81207.1

MKSKRMEFHNKFLIVSAMLAVISWLSLGVVSFPMLAGTLGISTKAAATVVNLISAYSTVTAVISIVGAITGVGSIGSGIAATVLYILKKKGAAKAALW

>Streptococcus-pneumoniae_CWK42342.1

MKSKRMEFHNKFLIVSAMLAVISWLSLGVVSFPMLAGTLGISTKAAATVVNLISAYSTVTAVISIVGAITGVGSIGSGIAATVLYILKKKGAAKAALW

>Streptococcus-pneumoniae_CWG03965.1

MKSKRMEFHNKFLIVSAMLAVISWLSLGVVSFPMLAGTLGISTKAAATVVNLISAYSTVTAVISIVGAITGVGSIGSGIAATVLYILKKKGAAKAALW

>Streptococcus-pneumoniae_CWE18576.1

MKSKRMEFHNKFLIVSAMLAVISWLSLGVVSFPMLAGTLGISTKAAATVVNLISAYSTVTAVISIVGAITGVGSIGSGIAATVLYILKKKGAAKAALW

>Streptococcus-pneumoniae_CWJ33722.1

MKSKRMEFHNKFLIVSAMLAVISWLSLGVVSFPMLAGTLGISTKAAATVVNLISAYSTVTAVISIVGAITGVGSIGSGIAATVLYILKKKGAAKAALW

>Streptococcus-pneumoniae_CWE20866.1

MKSKRMEFHNKFLIVSAMLAVISWLSLGVVSFPMLAGTLGISTKAAATVVNLISAYSTVTAVISIVGAITGVGSIGSGIAATVLYILKKKGAAKAALW

>Streptococcus-pneumoniae_CVY87828.1

MKSKRMEFHNKFLIVSAMLAVISWLSLGVVSFPMLAGTLGISTKAAATVVNLISAYSTVTAVISIVGAITGVGSIGSGIAATVLYILKKKGAAKAALW

>Streptococcus-pneumoniae_CVZ80515.1

MKSKRMEFHNKFLIVSAMLAVISWLSLGVVSFPMLAGTLGISTKAAATVVNLISAYSTVTAVISIVGAITGVGSIGSGIAATVLYILKKKGAAKAALW

>Streptococcus-pneumoniae_CWL98835.1

MKSKRMEFHNKFLIVSAMLAVISWLSLGVVSFPMLAGTLGISTKAAATVVNLISAYSTVTAVISIVGAITGVGSIGSGIAATVLYILKKKGAAKAALW

>Streptococcus-pneumoniae_CWK91064.1

MKSKRMEFHNKFLIVSAMLAVISWLSLGVVSFPMLAGTLGISTKAAATVVNLISAYSTVTAVISIVGAITGVGSIGSGIAATVLYILKKKGAAKAALW

>Streptococcus-pneumoniae_CWD32404.1

MKSKRMEFHNKFLIVSAMLAVISWLSLGVVSFPMLAGTLGISTKAAATVVNLISAYSTVTAVISIVGAITGVGSIGSGIAATVLYILKKKGAAKAALW

>Streptococcus-pneumoniae_CVZ33188.1

MKSKRMEFHNKFLIVSAMLAVISWLSLGVVSFPMLAGTLGISTKAAATVVNLISAYSTVTAVISIVGAITGVGSIGSGIAATVLYILKKKGAAKAALW

>Streptococcus-pneumoniae_CWI16279.1

MKSKRMEFHNKFLIVSAMLAVISWLSLGVVSFPMLAGTLGISTKAAATVVNLISAYSTVTAVISIVGAITGVGSIGSGIAATVLYILKKKGAAKAALW

>Streptococcus-pneumoniae_CWL83474.1

MKSKRMEFHNKFLIVSAMLAVISWLSLGVVSFPMLAGTLGISTKAAATVVNLISAYSTVTAVISIVGAITGVGSIGSGIAATVLYILKKKGAAKAALW

>Streptococcus-pneumoniae_CWJ17418.1

MKSKRMEFHNKFLIVSAMLAVISWLSLGVVSFPMLAGTLGISTKAAATVVNLISAYSTVTAVISIVGAITGVGSIGSGIAATVLYILKKKGAAKAALW

>Streptococcus-pneumoniae_CVZ98919.1

MKSKRMEFHNKFLIVSAMLAVISWLSLGVVSFPMLAGTLGISTKAAATVVNLISAYSTVTAVISIVGAITGVGSIGSGIAATVLYILKKKGAAKAALW

>Streptococcus-pneumoniae_CWA53512.1

MKSKRMEFHNKFLIVSAMLAVISWLSLGVVSFPMLAGTLGISTKAAATVVNLISAYSTVTAVISIVGAITGVGSIGSGIAATVLYILKKKGAAKAALW

>Streptococcus-pneumoniae_CWD30857.1

MKSKRMEFHNKFLIVSAMLAVISWLSLGVVSFPMLAGTLGISTKAAATVVNLISAYSTVTAVISIVGAITGVGSIGSGIAATVLYILKKKGAAKAALW

>Streptococcus-pneumoniae_CVZ70387.1

MKSKRMEFHNKFLIVSAMLAVISWLSLGVVSFPMLAGTLGISTKAAATVVNLISAYSTVTAVISIVGAITGVGSIGSGIAATVLYILKKKGAAKAALW

>Streptococcus-pneumoniae_CWH75089.1

MKSKRMEFHNKFLIVSAMLAVISWLSLGVVSFPMLAGTLGISTKAAATVVNLISAYSTVTAVISIVGAITGVGSIGSGIAATVLYILKKKGAAKAALW

>Streptococcus-pneumoniae_CWF79336.1

MKSKRMEFHNKFLIVSAMLAVISWLSLGVVSFPMLAGTLGISTKAAATVVNLISAYSTVTAVISIVGAITGVGSIGSGIAATVLYILKKKGAAKAALW

>Streptococcus-pneumoniae_CWA11783.1

MKSKRMEFHNKFLIVSAMLAVISWLSLGVVSFPMLAGTLGISTKAAATVVNLISAYSTVTAVISIVGAITGVGSIGSGIAATVLYILKKKGAAKAALW

>Streptococcus-pneumoniae_CWF44754.1

MKSKRMEFHNKFLIVSAMLAVISWLSLGVVSFPMLAGTLGISTKAAATVVNLISAYSTVTAVISIVGAITGVGSIGSGIAATVLYILKKKGAAKAALW

>Streptococcus-pneumoniae_CVY33595.1

MKSKRMEFHNKFLIVSAMLAVISWLSLGVVSFPMLAGTLGISTKAAATVVNLISAYSTVTAVISIVGAITGVGSIGSGIAATVLYILKKKGAAKAALW

>Streptococcus-pneumoniae_CWD41313.1

MKSKRMEFHNKFLIVSAMLAVISWLSLGVVSFPMLAGTLGISTKAAATVVNLISAYSTVTAVISIVGAITGVGSIGSGIAATVLYILKKKGAAKAALW

>Streptococcus-pneumoniae_CWL01273.1

MKSKRMEFHNKFLIVSAMLAVISWLSLGVVSFPMLAGTLGISTKAAATVVNLISAYSTVTAVISIVGAITGVGSIGSGIAATVLYILKKKGAAKAALW

>Streptococcus-pneumoniae_CWA92494.1

MKSKRMEFHNKFLIVSAMLAVISWLSLGVVSFPMLAGTLGISTKAAATVVNLISAYSTVTAVISIVGAITGVGSIGSGIAATVLYILKKKGAAKAALW

>Streptococcus-pneumoniae_CXE88081.1

MKSKRMEFHNKFLIVSAMLAVISWLSLGVVSFPMLAGTLGISTKAAATVVNLISAYSTVTAVISIVGAITGVGSIGSGIAATVLYILKKKGAAKAALW

>Streptococcus-pneumoniae_CWL53159.1

MKSKRMEFHNKFLIVSAMLAVISWLSLGVVSFPMLAGTLGISTKAAATVVNLISAYSTVTAVISIVGAITGVGSIGSGIAATVLYILKKKGAAKAALW

>Streptococcus-pneumoniae_CZE05772.1

MKSKRMEFHNKFLIVSAMLAVISWLSLGVVSFPMLAGTLGISTKAAATVVNLISAYSTVTAVISIVGAITGVGSIGSGIAATVLYILKKKGAAKAALW

>Streptococcus-pneumoniae_CZD65269.1

MKSKRMEFHNKFLIVSAMLAVISWLSLGVVSFPMLAGTLGISTKAAATVVNLISAYSTVTAVISIVGAITGVGSIGSGIAATVLYILKKKGAAKAALW

>Streptococcus-pneumoniae_CZD29124.1

MKSKRMEFHNKFLIVSAMLAVISWLSLGVVSFPMLAGTLGISTKAAATVVNLISAYSTVTAVISIVGAITGVGSIGSGIAATVLYILKKKGAAKAALW

>Streptococcus-pneumoniae_CZD32731.1

MKSKRMEFHNKFLIVSAMLAVISWLSLGVVSFPMLAGTLGISTKAAATVVNLISAYSTVTAVISIVGAITGVGSIGSGIAATVLYILKKKGAAKAALW

>Streptococcus-pneumoniae_CYL58765.1

MKSKRMEFHNKFLIVSAMLAVISWLSLGVVSFPMLAGTLGISTKAAATVVNLISAYSTVTAVISIVGAITGVGSIGSGIAATVLYILKKKGAAKAALW

>Streptococcus-pneumoniae_CYN62543.1

MKSKRMEFHNKFLIVSAMLAVISWLSLGVVSFPMLAGTLGISTKAAATVVNLISAYSTVTAVISIVGAITGVGSIGSGIAATVLYILKKKGAAKAALW

>Streptococcus-pneumoniae_CYL86702.1

MKSKRMEFHNKFLIVSAMLAVISWLSLGVVSFPMLAGTLGISTKAAATVVNLISAYSTVTAVISIVGAITGVGSIGSGIAATVLYILKKKGAAKAALW

>Streptococcus-pneumoniae_CZD33172.1

MKSKRMEFHNKFLIVSAMLAVISWLSLGVVSFPMLAGTLGISTKAAATVVNLISAYSTVTAVISIVGAITGVGSIGSGIAATVLYILKKKGAAKAALW

>Streptococcus-pneumoniae_CYN44925.1

MKSKRMEFHNKFLIVSAMLAVISWLSLGVVSFPMLAGTLGISTKAAATVVNLISAYSTVTAVISIVGAITGVGSIGSGIAATVLYILKKKGAAKAALW

>Streptococcus-pneumoniae_CYM27579.1

MKSKRMEFHNKFLIVSAMLAVISWLSLGVVSFPMLAGTLGISTKAAATVVNLISAYSTVTAVISIVGAITGVGSIGSGIAATVLYILKKKGAAKAALW

>Streptococcus-pneumoniae_CYG57142.1

MKSKRMEFHNKFLIVSAMLAVISWLSLGVVSFPMLAGTLGISTKAAATVVNLISAYSTVTAVISIVGAITGVGSIGSGIAATVLYILKKKGAAKAALW

>Streptococcus-pneumoniae_CYM44994.1

MKSKRMEFHNKFLIVSAMLAVISWLSLGVVSFPMLAGTLGISTKAAATVVNLISAYSTVTAVISIVGAITGVGSIGSGIAATVLYILKKKGAAKAALW

>Streptococcus-pneumoniae_CYN43398.1

MKSKRMEFHNKFLIVSAMLAVISWLSLGVVSFPMLAGTLGISTKAAATVVNLISAYSTVTAVISIVGAITGVGSIGSGIAATVLYILKKKGAAKAALW

>Streptococcus-pneumoniae_CYK14477.1

MKSKRMEFHNKFLIVSAMLAVISWLSLGVVSFPMLAGTLGISTKAAATVVNLISAYSTVTAVISIVGAITGVGSIGSGIAATVLYILKKKGAAKAALW

>Streptococcus-pneumoniae_CYO79941.1

MKSKRMEFHNKFLIVSAMLAVISWLSLGVVSFPMLAGTLGISTKAAATVVNLISAYSTVTAVISIVGAITGVGSIGSGIAATVLYILKKKGAAKAALW

>Streptococcus-pneumoniae_CYM08821.1

MKSKRMEFHNKFLIVSAMLAVISWLSLGVVSFPMLAGTLGISTKAAATVVNLISAYSTVTAVISIVGAITGVGSIGSGIAATVLYILKKKGAAKAALW

>Streptococcus-pneumoniae_CYM44012.1

MKSKRMEFHNKFLIVSAMLAVISWLSLGVVSFPMLAGTLGISTKAAATVVNLISAYSTVTAVISIVGAITGVGSIGSGIAATVLYILKKKGAAKAALW

>Streptococcus-pneumoniae_CYM54307.1

MKSKRMEFHNKFLIVSAMLAVISWLSLGVVSFPMLAGTLGISTKAAATVVNLISAYSTVTAVISIVGAITGVGSIGSGIAATVLYILKKKGAAKAALW

>Streptococcus-pneumoniae_CYG26456.1

MKSKRMEFHNKFLIVSAMLAVISWLSLGVVSFPMLAGTLGISTKAAATVVNLISAYSTVTAVISIVGAITGVGSIGSGIAATVLYILKKKGAAKAALW

>Streptococcus-pneumoniae_CYH80029.1

MKSKRMEFHNKFLIVSAMLAVISWLSLGVVSFPMLAGTLGISTKAAATVVNLISAYSTVTAVISIVGAITGVGSIGSGIAATVLYILKKKGAAKAALW

>Streptococcus-pneumoniae_CZD67242.1

MKSKRMEFHNKFLIVSAMLAVISWLSLGVVSFPMLAGTLGISTKAAATVVNLISAYSTVTAVISIVGAITGVGSIGSGIAATVLYILKKKGAAKAALW

>Streptococcus-pneumoniae_CYH10692.1

MKSKRMEFHNKFLIVSAMLAVISWLSLGVVSFPMLAGTLGISTKAAATVVNLISAYSTVTAVISIVGAITGVGSIGSGIAATVLYILKKKGAAKAALW

>Streptococcus-pneumoniae_CXH02137.1

MKSKRMEFHNKFLIVSAMLAVISWLSLGVVSFPMLAGTLGISTKAAATVVNLISAYSTVTAVISIVGAITGVGSIGSGIAATVLYILKKKGAAKAALW

>Streptococcus-pneumoniae_CYM69282.1

MKSKRMEFHNKFLIVSAMLAVISWLSLGVVSFPMLAGTLGISTKAAATVVNLISAYSTVTAVISIVGAITGVGSIGSGIAATVLYILKKKGAAKAALW

>Streptococcus-pneumoniae_CYK22441.1

MKSKRMEFHNKFLIVSAMLAVISWLSLGVVSFPMLAGTLGISTKAAATVVNLISAYSTVTAVISIVGAITGVGSIGSGIAATVLYILKKKGAAKAALW

>Streptococcus-pneumoniae_CYH02287.1

MKSKRMEFHNKFLIVSAMLAVISWLSLGVVSFPMLAGTLGISTKAAATVVNLISAYSTVTAVISIVGAITGVGSIGSGIAATVLYILKKKGAAKAALW

>Streptococcus-pneumoniae_CYL11492.1

MKSKRMEFHNKFLIVSAMLAVISWLSLGVVSFPMLAGTLGISTKAAATVVNLISAYSTVTAVISIVGAITGVGSIGSGIAATVLYILKKKGAAKAALW

>Streptococcus-pneumoniae_CYH37666.1

MKSKRMEFHNKFLIVSAMLAVISWLSLGVVSFPMLAGTLGISTKAAATVVNLISAYSTVTAVISIVGAITGVGSIGSGIAATVLYILKKKGAAKAALW

>Streptococcus-pneumoniae_CYM98168.1

MKSKRMEFHNKFLIVSAMLAVISWLSLGVVSFPMLAGTLGISTKAAATVVNLISAYSTVTAVISIVGAITGVGSIGSGIAATVLYILKKKGAAKAALW

>Streptococcus-pneumoniae_CYO05170.1

MKSKRMEFHNKFLIVSAMLAVISWLSLGVVSFPMLAGTLGISTKAAATVVNLISAYSTVTAVISIVGAITGVGSIGSGIAATVLYILKKKGAAKAALW

>Streptococcus-pneumoniae_CYI32201.1

MKSKRMEFHNKFLIVSAMLAVISWLSLGVVSFPMLAGTLGISTKAAATVVNLISAYSTVTAVISIVGAITGVGSIGSGIAATVLYILKKKGAAKAALW

>Streptococcus-pneumoniae_CYH67798.1

MKSKRMEFHNKFLIVSAMLAVISWLSLGVVSFPMLAGTLGISTKAAATVVNLISAYSTVTAVISIVGAITGVGSIGSGIAATVLYILKKKGAAKAALW

>Streptococcus-pneumoniae_CYH92259.1

MKSKRMEFHNKFLIVSAMLAVISWLSLGVVSFPMLAGTLGISTKAAATVVNLISAYSTVTAVISIVGAITGVGSIGSGIAATVLYILKKKGAAKAALW

>Streptococcus-pneumoniae_CYK41528.1

MKSKRMEFHNKFLIVSAMLAVISWLSLGVVSFPMLAGTLGISTKAAATVVNLISAYSTVTAVISIVGAITGVGSIGSGIAATVLYILKKKGAAKAALW

>Streptococcus-pneumoniae_CYL85335.1

MKSKRMEFHNKFLIVSAMLAVISWLSLGVVSFPMLAGTLGISTKAAATVVNLISAYSTVTAVISIVGAITGVGSIGSGIAATVLYILKKKGAAKAALW

>Streptococcus-pneumoniae_CYI59390.1

MKSKRMEFHNKFLIVSAMLAVISWLSLGVVSFPMLAGTLGISTKAAATVVNLISAYSTVTAVISIVGAITGVGSIGSGIAATVLYILKKKGAAKAALW

>Streptococcus-pneumoniae_CXG41278.1

MKSKRMEFHNKFLIVSAMLAVISWLSLGVVSFPMLAGTLGISTKAAATVVNLISAYSTVTAVISIVGAITGVGSIGSGIAATVLYILKKKGAAKAALW

>Streptococcus-pneumoniae_CYH86266.1

MKSKRMEFHNKFLIVSAMLAVISWLSLGVVSFPMLAGTLGISTKAAATVVNLISAYSTVTAVISIVGAITGVGSIGSGIAATVLYILKKKGAAKAALW

>Streptococcus-pneumoniae_CYI10197.1

MKSKRMEFHNKFLIVSAMLAVISWLSLGVVSFPMLAGTLGISTKAAATVVNLISAYSTVTAVISIVGAITGVGSIGSGIAATVLYILKKKGAAKAALW

>Streptococcus-pneumoniae_CXG06369.1

MKSKRMEFHNKFLIVSAMLAVISWLSLGVVSFPMLAGTLGISTKAAATVVNLISAYSTVTAVISIVGAITGVGSIGSGIAATVLYILKKKGAAKAALW

>Streptococcus-pneumoniae_CXF95370.1

MKSKRMEFHNKFLIVSAMLAVISWLSLGVVSFPMLAGTLGISTKAAATVVNLISAYSTVTAVISIVGAITGVGSIGSGIAATVLYILKKKGAAKAALW

>Streptococcus-pneumoniae_CYL83283.1

MKSKRMEFHNKFLIVSAMLAVISWLSLGVVSFPMLAGTLGISTKAAATVVNLISAYSTVTAVISIVGAITGVGSIGSGIAATVLYILKKKGAAKAALW

>Streptococcus-pneumoniae_CYM81941.1

MKSKRMEFHNKFLIVSAMLAVISWLSLGVVSFPMLAGTLGISTKAAATVVNLISAYSTVTAVISIVGAITGVGSIGSGIAATVLYILKKKGAAKAALW

>Streptococcus-pneumoniae_CYI84584.1

MKSKRMEFHNKFLIVSAMLAVISWLSLGVVSFPMLAGTLGISTKAAATVVNLISAYSTVTAVISIVGAITGVGSIGSGIAATVLYILKKKGAAKAALW

>Streptococcus-pneumoniae_CXG33035.1

MKSKRMEFHNKFLIVSAMLAVISWLSLGVVSFPMLAGTLGISTKAAATVVNLISAYSTVTAVISIVGAITGVGSIGSGIAATVLYILKKKGAAKAALW

>Streptococcus-pneumoniae_CYM00410.1

MKSKRMEFHNKFLIVSAMLAVISWLSLGVVSFPMLAGTLGISTKAAATVVNLISAYSTVTAVISIVGAITGVGSIGSGIAATVLYILKKKGAAKAALW

>Streptococcus-pneumoniae_CYI40681.1

MKSKRMEFHNKFLIVSAMLAVISWLSLGVVSFPMLAGTLGISTKAAATVVNLISAYSTVTAVISIVGAITGVGSIGSGIAATVLYILKKKGAAKAALW

>Streptococcus-pneumoniae_CXG04062.1

MKSKRMEFHNKFLIVSAMLAVISWLSLGVVSFPMLAGTLGISTKAAATVVNLISAYSTVTAVISIVGAITGVGSIGSGIAATVLYILKKKGAAKAALW

>Streptococcus-pneumoniae_CYM93089.1

MKSKRMEFHNKFLIVSAMLAVISWLSLGVVSFPMLAGTLGISTKAAATVVNLISAYSTVTAVISIVGAITGVGSIGSGIAATVLYILKKKGAAKAALW

>Streptococcus-pneumoniae_CYO35725.1

MKSKRMEFHNKFLIVSAMLAVISWLSLGVVSFPMLAGTLGISTKAAATVVNLISAYSTVTAVISIVGAITGVGSIGSGIAATVLYILKKKGAAKAALW

>Streptococcus-pneumoniae_CYK41891.1

MKSKRMEFHNKFLIVSAMLAVISWLSLGVVSFPMLAGTLGISTKAAATVVNLISAYSTVTAVISIVGAITGVGSIGSGIAATVLYILKKKGAAKAALW

>Streptococcus-pneumoniae_CYI96778.1

MKSKRMEFHNKFLIVSAMLAVISWLSLGVVSFPMLAGTLGISTKAAATVVNLISAYSTVTAVISIVGAITGVGSIGSGIAATVLYILKKKGAAKAALW

>Streptococcus-pneumoniae_CXG29989.1

MKSKRMEFHNKFLIVSAMLAVISWLSLGVVSFPMLAGTLGISTKAAATVVNLISAYSTVTAVISIVGAITGVGSIGSGIAATVLYILKKKGAAKAALW

>Streptococcus-pneumoniae_CXG65451.1

MKSKRMEFHNKFLIVSAMLAVISWLSLGVVSFPMLAGTLGISTKAAATVVNLISAYSTVTAVISIVGAITGVGSIGSGIAATVLYILKKKGAAKAALW

>Streptococcus-pneumoniae_CYM61082.1

MKSKRMEFHNKFLIVSAMLAVISWLSLGVVSFPMLAGTLGISTKAAATVVNLISAYSTVTAVISIVGAITGVGSIGSGIAATVLYILKKKGAAKAALW

>Streptococcus-pneumoniae_CYJ03791.1

MKSKRMEFHNKFLIVSAMLAVISWLSLGVVSFPMLAGTLGISTKAAATVVNLISAYSTVTAVISIVGAITGVGSIGSGIAATVLYILKKKGAAKAALW

>Streptococcus-pneumoniae_CYL96933.1

MKSKRMEFHNKFLIVSAMLAVISWLSLGVVSFPMLAGTLGISTKAAATVVNLISAYSTVTAVISIVGAITGVGSIGSGIAATVLYILKKKGAAKAALW

>Streptococcus-pneumoniae_CYN96267.1

MKSKRMEFHNKFLIVSAMLAVISWLSLGVVSFPMLAGTLGISTKAAATVVNLISAYSTVTAVISIVGAITGVGSIGSGIAATVLYILKKKGAAKAALW

>Streptococcus-pneumoniae_CYM04403.1

MKSKRMEFHNKFLIVSAMLAVISWLSLGVVSFPMLAGTLGISTKAAATVVNLISAYSTVTAVISIVGAITGVGSIGSGIAATVLYILKKKGAAKAALW

>Streptococcus-pneumoniae_CYG86919.1

MKSKRMEFHNKFLIVSAMLAVISWLSLGVVSFPMLAGTLGISTKAAATVVNLISAYSTVTAVISIVGAITGVGSIGSGIAATVLYILKKKGAAKAALW

>Streptococcus-pneumoniae_CYI97257.1

MKSKRMEFHNKFLIVSAMLAVISWLSLGVVSFPMLAGTLGISTKAAATVVNLISAYSTVTAVISIVGAITGVGSIGSGIAATVLYILKKKGAAKAALW

>Streptococcus-pneumoniae_CYI73720.1

MKSKRMEFHNKFLIVSAMLAVISWLSLGVVSFPMLAGTLGISTKAAATVVNLISAYSTVTAVISIVGAITGVGSIGSGIAATVLYILKKKGAAKAALW

>Streptococcus-pneumoniae_CXF71475.1

MKSKRMEFHNKFLIVSAMLAVISWLSLGVVSFPMLAGTLGISTKAAATVVNLISAYSTVTAVISIVGAITGVGSIGSGIAATVLYILKKKGAAKAALW

>Streptococcus-pneumoniae_CYI73487.1

MKSKRMEFHNKFLIVSAMLAVISWLSLGVVSFPMLAGTLGISTKAAATVVNLISAYSTVTAVISIVGAITGVGSIGSGIAATVLYILKKKGAAKAALW

>Streptococcus-pneumoniae_CYK96752.1

MKSKRMEFHNKFLIVSAMLAVISWLSLGVVSFPMLAGTLGISTKAAATVVNLISAYSTVTAVISIVGAITGVGSIGSGIAATVLYILKKKGAAKAALW

>Streptococcus-pneumoniae_CYI83911.1

MKSKRMEFHNKFLIVSAMLAVISWLSLGVVSFPMLAGTLGISTKAAATVVNLISAYSTVTAVISIVGAITGVGSIGSGIAATVLYILKKKGAAKAALW

>Streptococcus-pneumoniae_CZD18683.1

MKSKRMEFHNKFLIVSAMLAVISWLSLGVVSFPMLAGTLGISTKAAATVVNLISAYSTVTAVISIVGAITGVGSIGSGIAATVLYILKKKGAAKAALW

>Streptococcus-pneumoniae_CYN79139.1

MKSKRMEFHNKFLIVSAMLAVISWLSLGVVSFPMLAGTLGISTKAAATVVNLISAYSTVTAVISIVGAITGVGSIGSGIAATVLYILKKKGAAKAALW

>Streptococcus-pneumoniae_CYL60847.1

MKSKRMEFHNKFLIVSAMLAVISWLSLGVVSFPMLAGTLGISTKAAATVVNLISAYSTVTAVISIVGAITGVGSIGSGIAATVLYILKKKGAAKAALW

>Streptococcus-pneumoniae_CYI87272.1

MKSKRMEFHNKFLIVSAMLAVISWLSLGVVSFPMLAGTLGISTKAAATVVNLISAYSTVTAVISIVGAITGVGSIGSGIAATVLYILKKKGAAKAALW

>Streptococcus-pneumoniae_CYJ11595.1

MKSKRMEFHNKFLIVSAMLAVISWLSLGVVSFPMLAGTLGISTKAAATVVNLISAYSTVTAVISIVGAITGVGSIGSGIAATVLYILKKKGAAKAALW

>Streptococcus-pneumoniae_CYG72097.1

MKSKRMEFHNKFLIVSAMLAVISWLSLGVVSFPMLAGTLGISTKAAATVVNLISAYSTVTAVISIVGAITGVGSIGSGIAATVLYILKKKGAAKAALW

>Streptococcus-pneumoniae_CXG96558.1

MKSKRMEFHNKFLIVSAMLAVISWLSLGVVSFPMLAGTLGISTKAAATVVNLISAYSTVTAVISIVGAITGVGSIGSGIAATVLYILKKKGAAKAALW

>Streptococcus-pneumoniae_CYO92779.1

MKSKRMEFHNKFLIVSAMLAVISWLSLGVVSFPMLAGTLGISTKAAATVVNLISAYSTVTAVISIVGAITGVGSIGSGIAATVLYILKKKGAAKAALW

>Streptococcus-pneumoniae_CYL24952.1

MKSKRMEFHNKFLIVSAMLAVISWLSLGVVSFPMLAGTLGISTKAAATVVNLISAYSTVTAVISIVGAITGVGSIGSGIAATVLYILKKKGAAKAALW

>Streptococcus-pneumoniae_CZD88350.1

MKSKRMEFHNKFLIVSAMLAVISWLSLGVVSFPMLAGTLGISTKAAATVVNLISAYSTVTAVISIVGAITGVGSIGSGIAATVLYILKKKGAAKAALW

>Streptococcus-pneumoniae_CYH52775.1

MKSKRMEFHNKFLIVSAMLAVISWLSLGVVSFPMLAGTLGISTKAAATVVNLISAYSTVTAVISIVGAITGVGSIGSGIAATVLYILKKKGAAKAALW

>Streptococcus-pneumoniae_CYM81915.1

MKSKRMEFHNKFLIVSAMLAVISWLSLGVVSFPMLAGTLGISTKAAATVVNLISAYSTVTAVISIVGAITGVGSIGSGIAATVLYILKKKGAAKAALW

>Streptococcus-pneumoniae_CYI19759.1

MKSKRMEFHNKFLIVSAMLAVISWLSLGVVSFPMLAGTLGISTKAAATVVNLISAYSTVTAVISIVGAITGVGSIGSGIAATVLYILKKKGAAKAALW

>Streptococcus-pneumoniae_CYN72476.1

MKSKRMEFHNKFLIVSAMLAVISWLSLGVVSFPMLAGTLGISTKAAATVVNLISAYSTVTAVISIVGAITGVGSIGSGIAATVLYILKKKGAAKAALW

>Streptococcus-pneumoniae_CXG21221.1

MKSKRMEFHNKFLIVSAMLAVISWLSLGVVSFPMLAGTLGISTKAAATVVNLISAYSTVTAVISIVGAITGVGSIGSGIAATVLYILKKKGAAKAALW

>Streptococcus-pneumoniae_CYJ49494.1

MKSKRMEFHNKFLIVSAMLAVISWLSLGVVSFPMLAGTLGISTKAAATVVNLISAYSTVTAVISIVGAITGVGSIGSGIAATVLYILKKKGAAKAALW

>Streptococcus-pneumoniae_CYM61940.1

MKSKRMEFHNKFLIVSAMLAVISWLSLGVVSFPMLAGTLGISTKAAATVVNLISAYSTVTAVISIVGAITGVGSIGSGIAATVLYILKKKGAAKAALW

>Streptococcus-pneumoniae_CXF73907.1

MKSKRMEFHNKFLIVSAMLAVISWLSLGVVSFPMLAGTLGISTKAAATVVNLISAYSTVTAVISIVGAITGVGSIGSGIAATVLYILKKKGAAKAALW

>Streptococcus-pneumoniae_CYP06285.1

MKSKRMEFHNKFLIVSAMLAVISWLSLGVVSFPMLAGTLGISTKAAATVVNLISAYSTVTAVISIVGAITGVGSIGSGIAATVLYILKKKGAAKAALW

>Streptococcus-pneumoniae_CYH34921.1

MKSKRMEFHNKFLIVSAMLAVISWLSLGVVSFPMLAGTLGISTKAAATVVNLISAYSTVTAVISIVGAITGVGSIGSGIAATVLYILKKKGAAKAALW

>Streptococcus-pneumoniae_CYL21114.1

MKSKRMEFHNKFLIVSAMLAVISWLSLGVVSFPMLAGTLGISTKAAATVVNLISAYSTVTAVISIVGAITGVGSIGSGIAATVLYILKKKGAAKAALW

>Streptococcus-pneumoniae_CYI36927.1

MKSKRMEFHNKFLIVSAMLAVISWLSLGVVSFPMLAGTLGISTKAAATVVNLISAYSTVTAVISIVGAITGVGSIGSGIAATVLYILKKKGAAKAALW

>Streptococcus-pneumoniae_CZC99703.1

MKSKRMEFHNKFLIVSAMLAVISWLSLGVVSFPMLAGTLGISTKAAATVVNLISAYSTVTAVISIVGAITGVGSIGSGIAATVLYILKKKGAAKAALW

>Streptococcus-pneumoniae_CYH63367.1

MKSKRMEFHNKFLIVSAMLAVISWLSLGVVSFPMLAGTLGISTKAAATVVNLISAYSTVTAVISIVGAITGVGSIGSGIAATVLYILKKKGAAKAALW

>Streptococcus-pneumoniae_CYI35200.1

MKSKRMEFHNKFLIVSAMLAVISWLSLGVVSFPMLAGTLGISTKAAATVVNLISAYSTVTAVISIVGAITGVGSIGSGIAATVLYILKKKGAAKAALW

>Streptococcus-pneumoniae_CYK12822.1

MKSKRMEFHNKFLIVSAMLAVISWLSLGVVSFPMLAGTLGISTKAAATVVNLISAYSTVTAVISIVGAITGVGSIGSGIAATVLYILKKKGAAKAALW

>Streptococcus-pneumoniae_CYH04610.1

MKSKRMEFHNKFLIVSAMLAVISWLSLGVVSFPMLAGTLGISTKAAATVVNLISAYSTVTAVISIVGAITGVGSIGSGIAATVLYILKKKGAAKAALW

>Streptococcus-pneumoniae_CYK35313.1

MKSKRMEFHNKFLIVSAMLAVISWLSLGVVSFPMLAGTLGISTKAAATVVNLISAYSTVTAVISIVGAITGVGSIGSGIAATVLYILKKKGAAKAALW

>Streptococcus-pneumoniae_CYL26793.1

MKSKRMEFHNKFLIVSAMLAVISWLSLGVVSFPMLAGTLGISTKAAATVVNLISAYSTVTAVISIVGAITGVGSIGSGIAATVLYILKKKGAAKAALW

>Streptococcus-pneumoniae_CZD25027.1

MKSKRMEFHNKFLIVSAMLAVISWLSLGVVSFPMLAGTLGISTKAAATVVNLISAYSTVTAVISIVGAITGVGSIGSGIAATVLYILKKKGAAKAALW

>Streptococcus-pneumoniae_CXG94798.1

MKSKRMEFHNKFLIVSAMLAVISWLSLGVVSFPMLAGTLGISTKAAATVVNLISAYSTVTAVISIVGAITGVGSIGSGIAATVLYILKKKGAAKAALW

>Streptococcus-pneumoniae_CYJ12051.1

MKSKRMEFHNKFLIVSAMLAVISWLSLGVVSFPMLAGTLGISTKAAATVVNLISAYSTVTAVISIVGAITGVGSIGSGIAATVLYILKKKGAAKAALW

>Streptococcus-pneumoniae_CYN89462.1

MKSKRMEFHNKFLIVSAMLAVISWLSLGVVSFPMLAGTLGISTKAAATVVNLISAYSTVTAVISIVGAITGVGSIGSGIAATVLYILKKKGAAKAALW

>Streptococcus-pneumoniae_CZD19190.1

MKSKRMEFHNKFLIVSAMLAVISWLSLGVVSFPMLAGTLGISTKAAATVVNLISAYSTVTAVISIVGAITGVGSIGSGIAATVLYILKKKGAAKAALW

>Streptococcus-pneumoniae_CYH34736.1

MKSKRMEFHNKFLIVSAMLAVISWLSLGVVSFPMLAGTLGISTKAAATVVNLISAYSTVTAVISIVGAITGVGSIGSGIAATVLYILKKKGAAKAALW

>Streptococcus-pneumoniae_CYO65098.1

MKSKRMEFHNKFLIVSAMLAVISWLSLGVVSFPMLAGTLGISTKAAATVVNLISAYSTVTAVISIVGAITGVGSIGSGIAATVLYILKKKGAAKAALW

>Streptococcus-pneumoniae_CYO25837.1

MKSKRMEFHNKFLIVSAMLAVISWLSLGVVSFPMLAGTLGISTKAAATVVNLISAYSTVTAVISIVGAITGVGSIGSGIAATVLYILKKKGAAKAALW

>Streptococcus-pneumoniae_CYH57072.1

MKSKRMEFHNKFLIVSAMLAVISWLSLGVVSFPMLAGTLGISTKAAATVVNLISAYSTVTAVISIVGAITGVGSIGSGIAATVLYILKKKGAAKAALW

>Streptococcus-pneumoniae_CYJ19880.1

MKSKRMEFHNKFLIVSAMLAVISWLSLGVVSFPMLAGTLGISTKAAATVVNLISAYSTVTAVISIVGAITGVGSIGSGIAATVLYILKKKGAAKAALW

>Streptococcus-pneumoniae_CYH17967.1

MKSKRMEFHNKFLIVSAMLAVISWLSLGVVSFPMLAGTLGISTKAAATVVNLISAYSTVTAVISIVGAITGVGSIGSGIAATVLYILKKKGAAKAALW

>Streptococcus-pneumoniae_CYO68227.1

MKSKRMEFHNKFLIVSAMLAVISWLSLGVVSFPMLAGTLGISTKAAATVVNLISAYSTVTAVISIVGAITGVGSIGSGIAATVLYILKKKGAAKAALW

>Streptococcus-pneumoniae_CZE27109.1

MKSKRMEFHNKFLIVSAMLAVISWLSLGVVSFPMLAGTLGISTKAAATVVNLISAYSTVTAVISIVGAITGVGSIGSGIAATVLYILKKKGAAKAALW

>Streptococcus-pneumoniae_CYJ24581.1

MKSKRMEFHNKFLIVSAMLAVISWLSLGVVSFPMLAGTLGISTKAAATVVNLISAYSTVTAVISIVGAITGVGSIGSGIAATVLYILKKKGAAKAALW

>Streptococcus-pneumoniae_CZE29523.1

MKSKRMEFHNKFLIVSAMLAVISWLSLGVVSFPMLAGTLGISTKAAATVVNLISAYSTVTAVISIVGAITGVGSIGSGIAATVLYILKKKGAAKAALW

>Streptococcus-pneumoniae_CYM84846.1

MKSKRMEFHNKFLIVSAMLAVISWLSLGVVSFPMLAGTLGISTKAAATVVNLISAYSTVTAVISIVGAITGVGSIGSGIAATVLYILKKKGAAKAALW

>Streptococcus-pneumoniae_CYN47022.1

MKSKRMEFHNKFLIVSAMLAVISWLSLGVVSFPMLAGTLGISTKAAATVVNLISAYSTVTAVISIVGAITGVGSIGSGIAATVLYILKKKGAAKAALW

>Streptococcus-pneumoniae_CYM03140.1

MKSKRMEFHNKFLIVSAMLAVISWLSLGVVSFPMLAGTLGISTKAAATVVNLISAYSTVTAVISIVGAITGVGSIGSGIAATVLYILKKKGAAKAALW

>Streptococcus-pneumoniae_CZE28840.1

MKSKRMEFHNKFLIVSAMLAVISWLSLGVVSFPMLAGTLGISTKAAATVVNLISAYSTVTAVISIVGAITGVGSIGSGIAATVLYILKKKGAAKAALW

>Streptococcus-pneumoniae_CYO87546.1

MKSKRMEFHNKFLIVSAMLAVISWLSLGVVSFPMLAGTLGISTKAAATVVNLISAYSTVTAVISIVGAITGVGSIGSGIAATVLYILKKKGAAKAALW

>Streptococcus-pneumoniae_CYH75830.1

MKSKRMEFHNKFLIVSAMLAVISWLSLGVVSFPMLAGTLGISTKAAATVVNLISAYSTVTAVISIVGAITGVGSIGSGIAATVLYILKKKGAAKAALW

>Streptococcus-pneumoniae_CYH36976.1

MKSKRMEFHNKFLIVSAMLAVISWLSLGVVSFPMLAGTLGISTKAAATVVNLISAYSTVTAVISIVGAITGVGSIGSGIAATVLYILKKKGAAKAALW

>Streptococcus-pneumoniae_CYK52393.1

MKSKRMEFHNKFLIVSAMLAVISWLSLGVVSFPMLAGTLGISTKAAATVVNLISAYSTVTAVISIVGAITGVGSIGSGIAATVLYILKKKGAAKAALW

>Streptococcus-pneumoniae_CYM12161.1

MKSKRMEFHNKFLIVSAMLAVISWLSLGVVSFPMLAGTLGISTKAAATVVNLISAYSTVTAVISIVGAITGVGSIGSGIAATVLYILKKKGAAKAALW

>Streptococcus-pneumoniae_CYM74907.1

MKSKRMEFHNKFLIVSAMLAVISWLSLGVVSFPMLAGTLGISTKAAATVVNLISAYSTVTAVISIVGAITGVGSIGSGIAATVLYILKKKGAAKAALW

>Streptococcus-pneumoniae_CXF75657.1

MKSKRMEFHNKFLIVSAMLAVISWLSLGVVSFPMLAGTLGISTKAAATVVNLISAYSTVTAVISIVGAITGVGSIGSGIAATVLYILKKKGAAKAALW

>Streptococcus-pneumoniae_CYL90373.1

MKSKRMEFHNKFLIVSAMLAVISWLSLGVVSFPMLAGTLGISTKAAATVVNLISAYSTVTAVISIVGAITGVGSIGSGIAATVLYILKKKGAAKAALW

>Streptococcus-pneumoniae_CZE22628.1

MKSKRMEFHNKFLIVSAMLAVISWLSLGVVSFPMLAGTLGISTKAAATVVNLISAYSTVTAVISIVGAITGVGSIGSGIAATVLYILKKKGAAKAALW

>Streptococcus-pneumoniae_CZD16635.1

MKSKRMEFHNKFLIVSAMLAVISWLSLGVVSFPMLAGTLGISTKAAATVVNLISAYSTVTAVISIVGAITGVGSIGSGIAATVLYILKKKGAAKAALW

>Streptococcus-pneumoniae_CYN65242.1

MKSKRMEFHNKFLIVSAMLAVISWLSLGVVSFPMLAGTLGISTKAAATVVNLISAYSTVTAVISIVGAITGVGSIGSGIAATVLYILKKKGAAKAALW

>Streptococcus-pneumoniae_CZE10396.1

MKSKRMEFHNKFLIVSAMLAVISWLSLGVVSFPMLAGTLGISTKAAATVVNLISAYSTVTAVISIVGAITGVGSIGSGIAATVLYILKKKGAAKAALW

>Streptococcus-pneumoniae_CXG33717.1

MKSKRMEFHNKFLIVSAMLAVISWLSLGVVSFPMLAGTLGISTKAAATVVNLISAYSTVTAVISIVGAITGVGSIGSGIAATVLYILKKKGAAKAALW

>Streptococcus-pneumoniae_CYH25260.1

MKSKRMEFHNKFLIVSAMLAVISWLSLGVVSFPMLAGTLGISTKAAATVVNLISAYSTVTAVISIVGAITGVGSIGSGIAATVLYILKKKGAAKAALW

>Streptococcus-pneumoniae_CZD32817.1

MKSKRMEFHNKFLIVSAMLAVISWLSLGVVSFPMLAGTLGISTKAAATVVNLISAYSTVTAVISIVGAITGVGSIGSGIAATVLYILKKKGAAKAALW

>Streptococcus-pneumoniae_CYJ00333.1

MKSKRMEFHNKFLIVSAMLAVISWLSLGVVSFPMLAGTLGISTKAAATVVNLISAYSTVTAVISIVGAITGVGSIGSGIAATVLYILKKKGAAKAALW

>Streptococcus-pneumoniae_CYJ62615.1

MKSKRMEFHNKFLIVSAMLAVISWLSLGVVSFPMLAGTLGISTKAAATVVNLISAYSTVTAVISIVGAITGVGSIGSGIAATVLYILKKKGAAKAALW

>Streptococcus-pneumoniae_CZD94813.1

MKSKRMEFHNKFLIVSAMLAVISWLSLGVVSFPMLAGTLGISTKAAATVVNLISAYSTVTAVISIVGAITGVGSIGSGIAATVLYILKKKGAAKAALW

>Streptococcus-pneumoniae_CYH86587.1

MKSKRMEFHNKFLIVSAMLAVISWLSLGVVSFPMLAGTLGISTKAAATVVNLISAYSTVTAVISIVGAITGVGSIGSGIAATVLYILKKKGAAKAALW

>Streptococcus-pneumoniae_CXF78598.1

MKSKRMEFHNKFLIVSAMLAVISWLSLGVVSFPMLAGTLGISTKAAATVVNLISAYSTVTAVISIVGAITGVGSIGSGIAATVLYILKKKGAAKAALW

>Streptococcus-pneumoniae_CXG32497.1

MKSKRMEFHNKFLIVSAMLAVISWLSLGVVSFPMLAGTLGISTKAAATVVNLISAYSTVTAVISIVGAITGVGSIGSGIAATVLYILKKKGAAKAALW

>Streptococcus-pneumoniae_CZC61883.1

MKSKRMEFHNKFLIVSAMLAVISWLSLGVVSFPMLAGTLGISTKAAATVVNLISAYSTVTAVISIVGAITGVGSIGSGIAATVLYILKKKGAAKAALW

>Streptococcus-pneumoniae_CYI87315.1

MKSKRMEFHNKFLIVSAMLAVISWLSLGVVSFPMLAGTLGISTKAAATVVNLISAYSTVTAVISIVGAITGVGSIGSGIAATVLYILKKKGAAKAALW

>Streptococcus-pneumoniae_CZD50390.1

MKSKRMEFHNKFLIVSAMLAVISWLSLGVVSFPMLAGTLGISTKAAATVVNLISAYSTVTAVISIVGAITGVGSIGSGIAATVLYILKKKGAAKAALW

>Streptococcus-pneumoniae_CXG37991.1

MKSKRMEFHNKFLIVSAMLAVISWLSLGVVSFPMLAGTLGISTKAAATVVNLISAYSTVTAVISIVGAITGVGSIGSGIAATVLYILKKKGAAKAALW

>Streptococcus-pneumoniae_CZD94998.1

MKSKRMEFHNKFLIVSAMLAVISWLSLGVVSFPMLAGTLGISTKAAATVVNLISAYSTVTAVISIVGAITGVGSIGSGIAATVLYILKKKGAAKAALW

>Streptococcus-pneumoniae_CZD11888.1

MKSKRMEFHNKFLIVSAMLAVISWLSLGVVSFPMLAGTLGISTKAAATVVNLISAYSTVTAVISIVGAITGVGSIGSGIAATVLYILKKKGAAKAALW

>Streptococcus-pneumoniae_CZD17178.1

MKSKRMEFHNKFLIVSAMLAVISWLSLGVVSFPMLAGTLGISTKAAATVVNLISAYSTVTAVISIVGAITGVGSIGSGIAATVLYILKKKGAAKAALW

>Streptococcus-pneumoniae_CZE31422.1

MKSKRMEFHNKFLIVSAMLAVISWLSLGVVSFPMLAGTLGISTKAAATVVNLISAYSTVTAVISIVGAITGVGSIGSGIAATVLYILKKKGAAKAALW

>Streptococcus-pneumoniae_CZD09034.1

MKSKRMEFHNKFLIVSAMLAVISWLSLGVVSFPMLAGTLGISTKAAATVVNLISAYSTVTAVISIVGAITGVGSIGSGIAATVLYILKKKGAAKAALW

>Streptococcus-pneumoniae_CXG44771.1

MKSKRMEFHNKFLIVSAMLAVISWLSLGVVSFPMLAGTLGISTKAAATVVNLISAYSTVTAVISIVGAITGVGSIGSGIAATVLYILKKKGAAKAALW

>Streptococcus-pneumoniae_CYG90626.1

MKSKRMEFHNKFLIVSAMLAVISWLSLGVVSFPMLAGTLGISTKAAATVVNLISAYSTVTAVISIVGAITGVGSIGSGIAATVLYILKKKGAAKAALW

>Streptococcus-pneumoniae_CZD88216.1

MKSKRMEFHNKFLIVSAMLAVISWLSLGVVSFPMLAGTLGISTKAAATVVNLISAYSTVTAVISIVGAITGVGSIGSGIAATVLYILKKKGAAKAALW

>Streptococcus-pneumoniae_CZC96883.1

MKSKRMEFHNKFLIVSAMLAVISWLSLGVVSFPMLAGTLGISTKAAATVVNLISAYSTVTAVISIVGAITGVGSIGSGIAATVLYILKKKGAAKAALW

>Streptococcus-pneumoniae_CZC98750.1

MKSKRMEFHNKFLIVSAMLAVISWLSLGVVSFPMLAGTLGISTKAAATVVNLISAYSTVTAVISIVGAITGVGSIGSGIAATVLYILKKKGAAKAALW

>Streptococcus-pneumoniae_CZD02285.1

MKSKRMEFHNKFLIVSAMLAVISWLSLGVVSFPMLAGTLGISTKAAATVVNLISAYSTVTAVISIVGAITGVGSIGSGIAATVLYILKKKGAAKAALW

>Streptococcus-pneumoniae_CZD03402.1

MKSKRMEFHNKFLIVSAMLAVISWLSLGVVSFPMLAGTLGISTKAAATVVNLISAYSTVTAVISIVGAITGVGSIGSGIAATVLYILKKKGAAKAALW

>Streptococcus-pneumoniae_CZD09448.1

MKSKRMEFHNKFLIVSAMLAVISWLSLGVVSFPMLAGTLGISTKAAATVVNLISAYSTVTAVISIVGAITGVGSIGSGIAATVLYILKKKGAAKAALW

>Streptococcus-pneumoniae_CYH54396.1

MKSKRMEFHNKFLIVSAMLAVISWLSLGVVSFPMLAGTLGISTKAAATVVNLISAYSTVTAVISIVGAITGVGSIGSGIAATVLYILKKKGAAKAALW

>Streptococcus-pneumoniae_CYN08527.1

MKSKRMEFHNKFLIVSAMLAVISWLSLGVVSFPMLAGTLGISTKAAATVVNLISAYSTVTAVISIVGAITGVGSIGSGIAATVLYILKKKGAAKAALW

>Streptococcus-pneumoniae_CZE06578.1

MKSKRMEFHNKFLIVSAMLAVISWLSLGVVSFPMLAGTLGISTKAAATVVNLISAYSTVTAVISIVGAITGVGSIGSGIAATVLYILKKKGAAKAALW

>Streptococcus-pneumoniae_CZE37711.1

MKSKRMEFHNKFLIVSAMLAVISWLSLGVVSFPMLAGTLGISTKAAATVVNLISAYSTVTAVISIVGAITGVGSIGSGIAATVLYILKKKGAAKAALW

>Streptococcus-pneumoniae_CZD99100.1

MKSKRMEFHNKFLIVSAMLAVISWLSLGVVSFPMLAGTLGISTKAAATVVNLISAYSTVTAVISIVGAITGVGSIGSGIAATVLYILKKKGAAKAALW

>Streptococcus-pneumoniae_CYN81672.1

MKSKRMEFHNKFLIVSAMLAVISWLSLGVVSFPMLAGTLGISTKAAATVVNLISAYSTVTAVISIVGAITGVGSIGSGIAATVLYILKKKGAAKAALW

>Streptococcus-pneumoniae_CZE02971.1

MKSKRMEFHNKFLIVSAMLAVISWLSLGVVSFPMLAGTLGISTKAAATVVNLISAYSTVTAVISIVGAITGVGSIGSGIAATVLYILKKKGAAKAALW

>Streptococcus-pneumoniae_CXG08487.1

MKSKRMEFHNKFLIVSAMLAVISWLSLGVVSFPMLAGTLGISTKAAATVVNLISAYSTVTAVISIVGAITGVGSIGSGIAATVLYILKKKGAAKAALW

>Streptococcus-pneumoniae_CZE38208.1

MKSKRMEFHNKFLIVSAMLAVISWLSLGVVSFPMLAGTLGISTKAAATVVNLISAYSTVTAVISIVGAITGVGSIGSGIAATVLYILKKKGAAKAALW

>Streptococcus-pneumoniae_CZE05728.1

MKSKRMEFHNKFLIVSAMLAVISWLSLGVVSFPMLAGTLGISTKAAATVVNLISAYSTVTAVISIVGAITGVGSIGSGIAATVLYILKKKGAAKAALW

>Streptococcus-pneumoniae_CZE38809.1

MKSKRMEFHNKFLIVSAMLAVISWLSLGVVSFPMLAGTLGISTKAAATVVNLISAYSTVTAVISIVGAITGVGSIGSGIAATVLYILKKKGAAKAALW

>Streptococcus-pneumoniae_CXG47393.1

MKSKRMEFHNKFLIVSAMLAVISWLSLGVVSFPMLAGTLGISTKAAATVVNLISAYSTVTAVISIVGAITGVGSIGSGIAATVLYILKKKGAAKAALW

>Streptococcus-pneumoniae_CYJ94780.1

MKSKRMEFHNKFLIVSAMLAVISWLSLGVVSFPMLAGTLGISTKAAATVVNLISAYSTVTAVISIVGAITGVGSIGSGIAATVLYILKKKGAAKAALW

>Streptococcus-pneumoniae_CZD96542.1

MKSKRMEFHNKFLIVSAMLAVISWLSLGVVSFPMLAGTLGISTKAAATVVNLISAYSTVTAVISIVGAITGVGSIGSGIAATVLYILKKKGAAKAALW

>Streptococcus-pneumoniae_CZD06640.1

MKSKRMEFHNKFLIVSAMLAVISWLSLGVVSFPMLAGTLGISTKAAATVVNLISAYSTVTAVISIVGAITGVGSIGSGIAATVLYILKKKGAAKAALW

>Streptococcus-pneumoniae_CZE19945.1

MKSKRMEFHNKFLIVSAMLAVISWLSLGVVSFPMLAGTLGISTKAAATVVNLISAYSTVTAVISIVGAITGVGSIGSGIAATVLYILKKKGAAKAALW

>Streptococcus-pneumoniae_CZE03627.1

MKSKRMEFHNKFLIVSAMLAVISWLSLGVVSFPMLAGTLGISTKAAATVVNLISAYSTVTAVISIVGAITGVGSIGSGIAATVLYILKKKGAAKAALW

>Streptococcus-pneumoniae_CYL09923.1

MKSKRMEFHNKFLIVSAMLAVISWLSLGVVSFPMLAGTLGISTKAAATVVNLISAYSTVTAVISIVGAITGVGSIGSGIAATVLYILKKKGAAKAALW

>Streptococcus-pneumoniae_CYO97082.1

MKSKRMEFHNKFLIVSAMLAVISWLSLGVVSFPMLAGTLGISTKAAATVVNLISAYSTVTAVISIVGAITGVGSIGSGIAATVLYILKKKGAAKAALW

>Streptococcus-pneumoniae_CYI62383.1

MKSKRMEFHNKFLIVSAMLAVISWLSLGVVSFPMLAGTLGISTKAAATVVNLISAYSTVTAVISIVGAITGVGSIGSGIAATVLYILKKKGAAKAALW

>Streptococcus-pneumoniae_CXG90730.1

MKSKRMEFHNKFLIVSAMLAVISWLSLGVVSFPMLAGTLGISTKAAATVVNLISAYSTVTAVISIVGAITGVGSIGSGIAATVLYILKKKGAAKAALW

>Streptococcus-pneumoniae_CYK17170.1

MKSKRMEFHNKFLIVSAMLAVISWLSLGVVSFPMLAGTLGISTKAAATVVNLISAYSTVTAVISIVGAITGVGSIGSGIAATVLYILKKKGAAKAALW

>Streptococcus-pneumoniae_CYJ94752.1

MKSKRMEFHNKFLIVSAMLAVISWLSLGVVSFPMLAGTLGISTKAAATVVNLISAYSTVTAVISIVGAITGVGSIGSGIAATVLYILKKKGAAKAALW

>Streptococcus-pneumoniae_CZD76657.1

MKSKRMEFHNKFLIVSAMLAVISWLSLGVVSFPMLAGTLGISTKAAATVVNLISAYSTVTAVISIVGAITGVGSIGSGIAATVLYILKKKGAAKAALW

>Streptococcus-pneumoniae_KXT23487.1

MKSKRMEFHNKFLIVSAMLAVISWLSLGVVSFPMLAGTLGISTKAAATVVNLISAYSTVTAVISIVGAITGVGSIGSGIAATVLYILKKKGAAKAALW

>Streptococcus-pneumoniae_KXV84416.1

MKSKRMEFHNKFLIVSAMLAVISWLSLGVVSFPMLAGTLGISTKAAATVVNLISAYSTVTAVISIVGAITGVGSIGSGIAATVLYILKKKGAAKAALW

>Streptococcus-pneumoniae_KXV86432.1

MKSKRMEFHNKFLIVSAMLAVISWLSLGVVSFPMLAGTLGISTKAAATVVNLISAYSTVTAVISIVGAITGVGSIGSGIAATVLYILKKKGAAKAALW

>Streptococcus-pneumoniae_KXW07070.1

MKSKRMEFHNKFLIVSAMLAVISWLSLGVVSFPMLAGTLGISTKAAATVVNLISAYSTVTAVISIVGAITGVGSIGSGIAATVLYILKKKGAAKAALW

>Streptococcus-pneumoniae_KXW10806.1

MKSKRMEFHNKFLIVSAMLAVISWLSLGVVSFPMLAGTLGISTKAAATVVNLISAYSTVTAVISIVGAITGVGSIGSGIAATVLYILKKKGAAKAALW

>Streptococcus-pneumoniae_KXW23024.1

MKSKRMEFHNKFLIVSAMLAVISWLSLGVVSFPMLAGTLGISTKAAATVVNLISAYSTVTAVISIVGAITGVGSIGSGIAATVLYILKKKGAAKAALW

>Streptococcus-pneumoniae_KXW26988.1

MKSKRMEFHNKFLIVSAMLAVISWLSLGVVSFPMLAGTLGISTKAAATVVNLISAYSTVTAVISIVGAITGVGSIGSGIAATVLYILKKKGAAKAALW

>Streptococcus-pneumoniae_KXW27256.1

MKSKRMEFHNKFLIVSAMLAVISWLSLGVVSFPMLAGTLGISTKAAATVVNLISAYSTVTAVISIVGAITGVGSIGSGIAATVLYILKKKGAAKAALW

>Streptococcus-pneumoniae_KXW37100.1

MKSKRMEFHNKFLIVSAMLAVISWLSLGVVSFPMLAGTLGISTKAAATVVNLISAYSTVTAVISIVGAITGVGSIGSGIAATVLYILKKKGAAKAALW

>Streptococcus-pneumoniae_KXW38067.1

MKSKRMEFHNKFLIVSAMLAVISWLSLGVVSFPMLAGTLGISTKAAATVVNLISAYSTVTAVISIVGAITGVGSIGSGIAATVLYILKKKGAAKAALW

>Streptococcus-pneumoniae_KXW45435.1

MKSKRMEFHNKFLIVSAMLAVISWLSLGVVSFPMLAGTLGISTKAAATVVNLISAYSTVTAVISIVGAITGVGSIGSGIAATVLYILKKKGAAKAALW

>Streptococcus-pneumoniae_KXW47377.1

MKSKRMEFHNKFLIVSAMLAVISWLSLGVVSFPMLAGTLGISTKAAATVVNLISAYSTVTAVISIVGAITGVGSIGSGIAATVLYILKKKGAAKAALW

>Streptococcus-pneumoniae_KXW48916.1

MKSKRMEFHNKFLIVSAMLAVISWLSLGVVSFPMLAGTLGISTKAAATVVNLISAYSTVTAVISIVGAITGVGSIGSGIAATVLYILKKKGAAKAALW

>Streptococcus-pneumoniae_KXW50569.1

MKSKRMEFHNKFLIVSAMLAVISWLSLGVVSFPMLAGTLGISTKAAATVVNLISAYSTVTAVISIVGAITGVGSIGSGIAATVLYILKKKGAAKAALW

>Streptococcus-pneumoniae_KYA85493.1

MKSKRMEFHNKFLIVSAMLAVISWLSLGVVSFPMLAGTLGISTKAAATVVNLISAYSTVTAVISIVGAITGVGSIGSGIAATVLYILKKKGAAKAALW

>Streptococcus-pneumoniae_KYA88467.1

MKSKRMEFHNKFLIVSAMLAVISWLSLGVVSFPMLAGTLGISTKAAATVVNLISAYSTVTAVISIVGAITGVGSIGSGIAATVLYILKKKGAAKAALW

>Streptococcus-pneumoniae_OAB80884.1

MKSKRMEFHNKFLIVSAMLAVISWLSLGVVSFPMLAGTLGISTKAAATVVNLISAYSTVTAVISIVGAITGVGSIGSGIAATVLYILKKKGAAKAALW

>Streptococcus-pneumoniae_ANO35958.1

MKSKRMEFHNKFLIVSAMLAVISWLSLGVVSFPMLAGTLGISTKAAATVVNLISAYSTVTAVISIVGAITGVGSIGSGIAATVLYILKKKGAAKAALW

>Streptococcus-pneumoniae_OCQ83439.1

MKSKRMEFHNKFLIVSAMLAVISWLSLGVVSFPMLAGTLGISTKAAATVVNLISAYSTVTAVISIVGAITGVGSIGSGIAATVLYILKKKGAAKAALW

>Streptococcus-pneumoniae_OCQ87242.1

MKSKRMEFHNKFLIVSAMLAVISWLSLGVVSFPMLAGTLGISTKAAATVVNLISAYSTVTAVISIVGAITGVGSIGSGIAATVLYILKKKGAAKAALW

>Streptococcus-pneumoniae_AOG55001.1

MKSKRMEFHNKFLIVSAMLAVISWLSLGVVSFPMLAGTLGISTKAAATVVNLISAYSTVTAVISIVGAITGVGSIGSGIAATVLYILKKKGAAKAALW

>Streptococcus-pneumoniae_AOG57071.1

MKSKRMEFHNKFLIVSAMLAVISWLSLGVVSFPMLAGTLGISTKAAATVVNLISAYSTVTAVISIVGAITGVGSIGSGIAATVLYILKKKGAAKAALW

>Streptococcus-pneumoniae_ODJ78622.1

MKSKRMEFHNKFLIVSAMLAVISWLSLGVVSFPMLAGTLGISTKAAATVVNLISAYSTVTAVISIVGAITGVGSIGSGIAATVLYILKKKGAAKAALW

>Streptococcus-pneumoniae_ODO23141.1

MKSKRMEFHNKFLIVSAMLAVISWLSLGVVSFPMLAGTLGISTKAAATVVNLISAYSTVTAVISIVGAITGVGSIGSGIAATVLYILKKKGAAKAALW

>Streptococcus-pneumoniae_ODO26006.1

MKSKRMEFHNKFLIVSAMLAVISWLSLGVVSFPMLAGTLGISTKAAATVVNLISAYSTVTAVISIVGAITGVGSIGSGIAATVLYILKKKGAAKAALW

>Streptococcus-pneumoniae_ODO48018.1

MKSKRMEFHNKFLIVSAMLAVISWLSLGVVSFPMLAGTLGISTKAAATVVNLISAYSTVTAVISIVGAITGVGSIGSGIAATVLYILKKKGAAKAALW

>Streptococcus-pneumoniae_CXF61211.1

MKSKRMEFHNKFLIVSAMLAVISWLSLGVVSFPMLAGTLGISTKAAATVVNLISAYSTVTAVISIVGAITGVGSIGSGIAATVLYILKKKGAAKAALW

>Streptococcus-pneumoniae_CXF62119.1

MKSKRMEFHNKFLIVSAMLAVISWLSLGVVSFPMLAGTLGISTKAAATVVNLISAYSTVTAVISIVGAITGVGSIGSGIAATVLYILKKKGAAKAALW

>Streptococcus-pneumoniae_CXF68121.1

MKSKRMEFHNKFLIVSAMLAVISWLSLGVVSFPMLAGTLGISTKAAATVVNLISAYSTVTAVISIVGAITGVGSIGSGIAATVLYILKKKGAAKAALW

>Streptococcus-pneumoniae_CXF49252.1

MKSKRMEFHNKFLIVSAMLAVISWLSLGVVSFPMLAGTLGISTKAAATVVNLISAYSTVTAVISIVGAITGVGSIGSGIAATVLYILKKKGAAKAALW

>Streptococcus-pneumoniae_CXF64755.1

MKSKRMEFHNKFLIVSAMLAVISWLSLGVVSFPMLAGTLGISTKAAATVVNLISAYSTVTAVISIVGAITGVGSIGSGIAATVLYILKKKGAAKAALW

>Streptococcus-pneumoniae_OHX46440.1

MKSKRMEFHNKFLIVSAMLAVISWLSLGVVSFPMLAGTLGISTKAAATVVNLISAYSTVTAVISIVGAITGVGSIGSGIAATVLYILKKKGAAKAALW

>Streptococcus-pneumoniae-WU2_OKQ23643.1

MKSKRMEFHNKFLIVSAMLAVISWLSLGVVSFPMLAGTLGISTKAAATVVNLISAYSTVTAVISIVGAITGVGSIGSGIAATVLYILKKKGAAKAALW

>Streptococcus-pneumoniae_OKQ27361.1

MKSKRMEFHNKFLIVSAMLAVISWLSLGVVSFPMLAGTLGISTKAAATVVNLISAYSTVTAVISIVGAITGVGSIGSGIAATVLYILKKKGAAKAALW

>Streptococcus-pneumoniae_OKQ50630.1

MKSKRMEFHNKFLIVSAMLAVISWLSLGVVSFPMLAGTLGISTKAAATVVNLISAYSTVTAVISIVGAITGVGSIGSGIAATVLYILKKKGAAKAALW

>Streptococcus-pneumoniae_SBW16528.1

MKSKRMEFHNKFLIVSAMLAVISWLSLGVVSFPMLAGTLGISTKAAATVVNLISAYSTVTAVISIVGAITGVGSIGSGIAATVLYILKKKGAAKAALW

>Streptococcus-pneumoniae_SBO78152.1

MKSKRMEFHNKFLIVSAMLAVISWLSLGVVSFPMLAGTLGISTKAAATVVNLISAYSTVTAVISIVGAITGVGSIGSGIAATVLYILKKKGAAKAALW

>Streptococcus-pneumoniae_SBQ24617.1

MKSKRMEFHNKFLIVSAMLAVISWLSLGVVSFPMLAGTLGISTKAAATVVNLISAYSTVTAVISIVGAITGVGSIGSGIAATVLYILKKKGAAKAALW

>Streptococcus-pneumoniae_SBO83938.1

MKSKRMEFHNKFLIVSAMLAVISWLSLGVVSFPMLAGTLGISTKAAATVVNLISAYSTVTAVISIVGAITGVGSIGSGIAATVLYILKKKGAAKAALW

>Streptococcus-pneumoniae_SBU07734.1

MKSKRMEFHNKFLIVSAMLAVISWLSLGVVSFPMLAGTLGISTKAAATVVNLISAYSTVTAVISIVGAITGVGSIGSGIAATVLYILKKKGAAKAALW

>Streptococcus-pneumoniae_SBP92216.1

MKSKRMEFHNKFLIVSAMLAVISWLSLGVVSFPMLAGTLGISTKAAATVVNLISAYSTVTAVISIVGAITGVGSIGSGIAATVLYILKKKGAAKAALW

>Streptococcus-pneumoniae_SBU01777.1

MKSKRMEFHNKFLIVSAMLAVISWLSLGVVSFPMLAGTLGISTKAAATVVNLISAYSTVTAVISIVGAITGVGSIGSGIAATVLYILKKKGAAKAALW

>Streptococcus-pneumoniae_SBP91093.1

MKSKRMEFHNKFLIVSAMLAVISWLSLGVVSFPMLAGTLGISTKAAATVVNLISAYSTVTAVISIVGAITGVGSIGSGIAATVLYILKKKGAAKAALW

>Streptococcus-pneumoniae_SBP91328.1

MKSKRMEFHNKFLIVSAMLAVISWLSLGVVSFPMLAGTLGISTKAAATVVNLISAYSTVTAVISIVGAITGVGSIGSGIAATVLYILKKKGAAKAALW

>Streptococcus-pneumoniae_SBP90696.1

MKSKRMEFHNKFLIVSAMLAVISWLSLGVVSFPMLAGTLGISTKAAATVVNLISAYSTVTAVISIVGAITGVGSIGSGIAATVLYILKKKGAAKAALW

>Streptococcus-pneumoniae_SBV72000.1

MKSKRMEFHNKFLIVSAMLAVISWLSLGVVSFPMLAGTLGISTKAAATVVNLISAYSTVTAVISIVGAITGVGSIGSGIAATVLYILKKKGAAKAALW

>Streptococcus-pneumoniae_SBO77945.1

MKSKRMEFHNKFLIVSAMLAVISWLSLGVVSFPMLAGTLGISTKAAATVVNLISAYSTVTAVISIVGAITGVGSIGSGIAATVLYILKKKGAAKAALW

>Streptococcus-pneumoniae_SBP91045.1

MKSKRMEFHNKFLIVSAMLAVISWLSLGVVSFPMLAGTLGISTKAAATVVNLISAYSTVTAVISIVGAITGVGSIGSGIAATVLYILKKKGAAKAALW

>Streptococcus-pneumoniae_SBV78477.1

MKSKRMEFHNKFLIVSAMLAVISWLSLGVVSFPMLAGTLGISTKAAATVVNLISAYSTVTAVISIVGAITGVGSIGSGIAATVLYILKKKGAAKAALW

>Streptococcus-pneumoniae_SBO83989.1

MKSKRMEFHNKFLIVSAMLAVISWLSLGVVSFPMLAGTLGISTKAAATVVNLISAYSTVTAVISIVGAITGVGSIGSGIAATVLYILKKKGAAKAALW

>Streptococcus-pneumoniae_SBP90796.1

MKSKRMEFHNKFLIVSAMLAVISWLSLGVVSFPMLAGTLGISTKAAATVVNLISAYSTVTAVISIVGAITGVGSIGSGIAATVLYILKKKGAAKAALW

>Streptococcus-pneumoniae_SBV76170.1

MKSKRMEFHNKFLIVSAMLAVISWLSLGVVSFPMLAGTLGISTKAAATVVNLISAYSTVTAVISIVGAITGVGSIGSGIAATVLYILKKKGAAKAALW

>Streptococcus-pneumoniae_SBO78004.1

MKSKRMEFHNKFLIVSAMLAVISWLSLGVVSFPMLAGTLGISTKAAATVVNLISAYSTVTAVISIVGAITGVGSIGSGIAATVLYILKKKGAAKAALW

>Streptococcus-pneumoniae_SBP90721.1

MKSKRMEFHNKFLIVSAMLAVISWLSLGVVSFPMLAGTLGISTKAAATVVNLISAYSTVTAVISIVGAITGVGSIGSGIAATVLYILKKKGAAKAALW

>Streptococcus-pneumoniae_SBO84102.1

MKSKRMEFHNKFLIVSAMLAVISWLSLGVVSFPMLAGTLGISTKAAATVVNLISAYSTVTAVISIVGAITGVGSIGSGIAATVLYILKKKGAAKAALW

>Streptococcus-pneumoniae_SBV69965.1

MKSKRMEFHNKFLIVSAMLAVISWLSLGVVSFPMLAGTLGISTKAAATVVNLISAYSTVTAVISIVGAITGVGSIGSGIAATVLYILKKKGAAKAALW

>Streptococcus-pneumoniae_SBU02039.1

MKSKRMEFHNKFLIVSAMLAVISWLSLGVVSFPMLAGTLGISTKAAATVVNLISAYSTVTAVISIVGAITGVGSIGSGIAATVLYILKKKGAAKAALW

>Streptococcus-pneumoniae_SBV76097.1

MKSKRMEFHNKFLIVSAMLAVISWLSLGVVSFPMLAGTLGISTKAAATVVNLISAYSTVTAVISIVGAITGVGSIGSGIAATVLYILKKKGAAKAALW

>Streptococcus-pneumoniae_SBP91644.1

MKSKRMEFHNKFLIVSAMLAVISWLSLGVVSFPMLAGTLGISTKAAATVVNLISAYSTVTAVISIVGAITGVGSIGSGIAATVLYILKKKGAAKAALW

>Streptococcus-pneumoniae_SBO58178.1

MKSKRMEFHNKFLIVSAMLAVISWLSLGVVSFPMLAGTLGISTKAAATVVNLISAYSTVTAVISIVGAITGVGSIGSGIAATVLYILKKKGAAKAALW

>Streptococcus-pneumoniae_SBP92031.1

MKSKRMEFHNKFLIVSAMLAVISWLSLGVVSFPMLAGTLGISTKAAATVVNLISAYSTVTAVISIVGAITGVGSIGSGIAATVLYILKKKGAAKAALW

>Streptococcus-pneumoniae_SBP91204.1

MKSKRMEFHNKFLIVSAMLAVISWLSLGVVSFPMLAGTLGISTKAAATVVNLISAYSTVTAVISIVGAITGVGSIGSGIAATVLYILKKKGAAKAALW

>Streptococcus-pneumoniae_SBV72050.1

MKSKRMEFHNKFLIVSAMLAVISWLSLGVVSFPMLAGTLGISTKAAATVVNLISAYSTVTAVISIVGAITGVGSIGSGIAATVLYILKKKGAAKAALW

>Streptococcus-pneumoniae_SBQ18950.1

MKSKRMEFHNKFLIVSAMLAVISWLSLGVVSFPMLAGTLGISTKAAATVVNLISAYSTVTAVISIVGAITGVGSIGSGIAATVLYILKKKGAAKAALW

>Streptococcus-pneumoniae_SBP90747.1

MKSKRMEFHNKFLIVSAMLAVISWLSLGVVSFPMLAGTLGISTKAAATVVNLISAYSTVTAVISIVGAITGVGSIGSGIAATVLYILKKKGAAKAALW

>Streptococcus-pneumoniae_SBV83370.1

MKSKRMEFHNKFLIVSAMLAVISWLSLGVVSFPMLAGTLGISTKAAATVVNLISAYSTVTAVISIVGAITGVGSIGSGIAATVLYILKKKGAAKAALW

>Streptococcus-pneumoniae_SBP91287.1

MKSKRMEFHNKFLIVSAMLAVISWLSLGVVSFPMLAGTLGISTKAAATVVNLISAYSTVTAVISIVGAITGVGSIGSGIAATVLYILKKKGAAKAALW

>Streptococcus-pneumoniae_SBV78690.1

MKSKRMEFHNKFLIVSAMLAVISWLSLGVVSFPMLAGTLGISTKAAATVVNLISAYSTVTAVISIVGAITGVGSIGSGIAATVLYILKKKGAAKAALW

>Streptococcus-pneumoniae_SBU01615.1

MKSKRMEFHNKFLIVSAMLAVISWLSLGVVSFPMLAGTLGISTKAAATVVNLISAYSTVTAVISIVGAITGVGSIGSGIAATVLYILKKKGAAKAALW

>Streptococcus-pneumoniae_SBU07928.1

MKSKRMEFHNKFLIVSAMLAVISWLSLGVVSFPMLAGTLGISTKAAATVVNLISAYSTVTAVISIVGAITGVGSIGSGIAATVLYILKKKGAAKAALW

>Streptococcus-pneumoniae_SBW19519.1

MKSKRMEFHNKFLIVSAMLAVISWLSLGVVSFPMLAGTLGISTKAAATVVNLISAYSTVTAVISIVGAITGVGSIGSGIAATVLYILKKKGAAKAALW

>Streptococcus-pneumoniae_SCA93162.1

MKSKRMEFHNKFLIVSAMLAVISWLSLGVVSFPMLAGTLGISTKAAATVVNLISAYSTVTAVISIVGAITGVGSIGSGIAATVLYILKKKGAAKAALW

>Streptococcus-pneumoniae_SBO58266.1

MKSKRMEFHNKFLIVSAMLAVISWLSLGVVSFPMLAGTLGISTKAAATVVNLISAYSTVTAVISIVGAITGVGSIGSGIAATVLYILKKKGAAKAALW

>Streptococcus-pneumoniae_ONG43760.1

MKSKRMEFHNKFLIVSAMLAVISWLSLGVVSFPMLAGTLGISTKAAATVVNLISAYSTVTAVISIVGAITGVGSIGSGIAATVLYILKKKGAAKAALW

>Streptococcus-pneumoniae_ONG46875.1

MKSKRMEFHNKFLIVSAMLAVISWLSLGVVSFPMLAGTLGISTKAAATVVNLISAYSTVTAVISIVGAITGVGSIGSGIAATVLYILKKKGAAKAALW

>Streptococcus-pseudopneumoniae_OOR82075.1

MKSKRMEFHNKFLIVSAMLAVISWLSLGVVSFPMLAGTLGISTKAAATVVNLISAYSTVTAVISIVGAITGVGSIGSGIAATVLYILKKKGAAKAALW

>Streptococcus-pneumoniae_OOS13145.1

MKSKRMEFHNKFLIVSAMLAVISWLSLGVVSFPMLAGTLGISTKAAATVVNLISAYSTVTAVISIVGAITGVGSIGSGIAATVLYILKKKGAAKAALW

>Streptococcus-pneumoniae_OOS14268.1

MKSKRMEFHNKFLIVSAMLAVISWLSLGVVSFPMLAGTLGISTKAAATVVNLISAYSTVTAVISIVGAITGVGSIGSGIAATVLYILKKKGAAKAALW

>Streptococcus-pneumoniae_OPA69886.1

MKSKRMEFHNKFLIVSAMLAVISWLSLGVVSFPMLAGTLGISTKAAATVVNLISAYSTVTAVISIVGAITGVGSIGSGIAATVLYILKKKGAAKAALW

>Streptococcus-pneumoniae_ARD33714.1

MKSKRMEFHNKFLIVSAMLAVISWLSLGVVSFPMLAGTLGISTKAAATVVNLISAYSTVTAVISIVGAITGVGSIGSGIAATVLYILKKKGAAKAALW

>Streptococcus-pneumoniae_ARD37410.1

MKSKRMEFHNKFLIVSAMLAVISWLSLGVVSFPMLAGTLGISTKAAATVVNLISAYSTVTAVISIVGAITGVGSIGSGIAATVLYILKKKGAAKAALW

>Streptococcus-pseudopneumoniae-ATCC-BAA-960-=-CCUG-49455_ORC38917.1

MKSKRMEFHNKFLIVSAMLAVISWLSLGVVSFPMLAGTLGISTKAAATVVNLISAYSTVTAVISIVGAITGVGSIGSGIAATVLYILKKKGAAKAALW

>Streptococcus-pneumoniae_SNM92894.1

MKSKRMEFHNKFLIVSAMLAVISWLSLGVVSFPMLAGTLGISTKAAATVVNLISAYSTVTAVISIVGAITGVGSIGSGIAATVLYILKKKGAAKAALW

>Streptococcus-pneumoniae_SNO15995.1

MKSKRMEFHNKFLIVSAMLAVISWLSLGVVSFPMLAGTLGISTKAAATVVNLISAYSTVTAVISIVGAITGVGSIGSGIAATVLYILKKKGAAKAALW

>Streptococcus-pneumoniae_SNN60821.1

MKSKRMEFHNKFLIVSAMLAVISWLSLGVVSFPMLAGTLGISTKAAATVVNLISAYSTVTAVISIVGAITGVGSIGSGIAATVLYILKKKGAAKAALW

>Streptococcus-pneumoniae_SNP74021.1

MKSKRMEFHNKFLIVSAMLAVISWLSLGVVSFPMLAGTLGISTKAAATVVNLISAYSTVTAVISIVGAITGVGSIGSGIAATVLYILKKKGAAKAALW

>Streptococcus-pneumoniae_SNI95796.1

MKSKRMEFHNKFLIVSAMLAVISWLSLGVVSFPMLAGTLGISTKAAATVVNLISAYSTVTAVISIVGAITGVGSIGSGIAATVLYILKKKGAAKAALW

>Streptococcus-pneumoniae_SNO03108.1

MKSKRMEFHNKFLIVSAMLAVISWLSLGVVSFPMLAGTLGISTKAAATVVNLISAYSTVTAVISIVGAITGVGSIGSGIAATVLYILKKKGAAKAALW

>Streptococcus-pneumoniae_SNP67730.1

MKSKRMEFHNKFLIVSAMLAVISWLSLGVVSFPMLAGTLGISTKAAATVVNLISAYSTVTAVISIVGAITGVGSIGSGIAATVLYILKKKGAAKAALW

>Streptococcus-pneumoniae_SNO19719.1

MKSKRMEFHNKFLIVSAMLAVISWLSLGVVSFPMLAGTLGISTKAAATVVNLISAYSTVTAVISIVGAITGVGSIGSGIAATVLYILKKKGAAKAALW

>Streptococcus-pneumoniae_SNP03205.1

MKSKRMEFHNKFLIVSAMLAVISWLSLGVVSFPMLAGTLGISTKAAATVVNLISAYSTVTAVISIVGAITGVGSIGSGIAATVLYILKKKGAAKAALW

>Streptococcus-pneumoniae_SNN71020.1

MKSKRMEFHNKFLIVSAMLAVISWLSLGVVSFPMLAGTLGISTKAAATVVNLISAYSTVTAVISIVGAITGVGSIGSGIAATVLYILKKKGAAKAALW

>Streptococcus-pneumoniae_SNH07709.1

MKSKRMEFHNKFLIVSAMLAVISWLSLGVVSFPMLAGTLGISTKAAATVVNLISAYSTVTAVISIVGAITGVGSIGSGIAATVLYILKKKGAAKAALW

>Streptococcus-pneumoniae_SNM99646.1

MKSKRMEFHNKFLIVSAMLAVISWLSLGVVSFPMLAGTLGISTKAAATVVNLISAYSTVTAVISIVGAITGVGSIGSGIAATVLYILKKKGAAKAALW

>Streptococcus-pneumoniae_SNP39322.1

MKSKRMEFHNKFLIVSAMLAVISWLSLGVVSFPMLAGTLGISTKAAATVVNLISAYSTVTAVISIVGAITGVGSIGSGIAATVLYILKKKGAAKAALW

>Streptococcus-pneumoniae_SNO22928.1

MKSKRMEFHNKFLIVSAMLAVISWLSLGVVSFPMLAGTLGISTKAAATVVNLISAYSTVTAVISIVGAITGVGSIGSGIAATVLYILKKKGAAKAALW

>Streptococcus-pneumoniae_SNN72494.1

MKSKRMEFHNKFLIVSAMLAVISWLSLGVVSFPMLAGTLGISTKAAATVVNLISAYSTVTAVISIVGAITGVGSIGSGIAATVLYILKKKGAAKAALW

>Streptococcus-pneumoniae_SNP17204.1

MKSKRMEFHNKFLIVSAMLAVISWLSLGVVSFPMLAGTLGISTKAAATVVNLISAYSTVTAVISIVGAITGVGSIGSGIAATVLYILKKKGAAKAALW

>Streptococcus-pneumoniae_SNP20767.1

MKSKRMEFHNKFLIVSAMLAVISWLSLGVVSFPMLAGTLGISTKAAATVVNLISAYSTVTAVISIVGAITGVGSIGSGIAATVLYILKKKGAAKAALW

>Streptococcus-pneumoniae_SNN39643.1

MKSKRMEFHNKFLIVSAMLAVISWLSLGVVSFPMLAGTLGISTKAAATVVNLISAYSTVTAVISIVGAITGVGSIGSGIAATVLYILKKKGAAKAALW

>Streptococcus-pneumoniae_SNO32662.1

MKSKRMEFHNKFLIVSAMLAVISWLSLGVVSFPMLAGTLGISTKAAATVVNLISAYSTVTAVISIVGAITGVGSIGSGIAATVLYILKKKGAAKAALW

>Streptococcus-pneumoniae_SNP57302.1

MKSKRMEFHNKFLIVSAMLAVISWLSLGVVSFPMLAGTLGISTKAAATVVNLISAYSTVTAVISIVGAITGVGSIGSGIAATVLYILKKKGAAKAALW

>Streptococcus-pneumoniae_SNQ01293.1

MKSKRMEFHNKFLIVSAMLAVISWLSLGVVSFPMLAGTLGISTKAAATVVNLISAYSTVTAVISIVGAITGVGSIGSGIAATVLYILKKKGAAKAALW

>Streptococcus-pneumoniae_SNP74945.1

MKSKRMEFHNKFLIVSAMLAVISWLSLGVVSFPMLAGTLGISTKAAATVVNLISAYSTVTAVISIVGAITGVGSIGSGIAATVLYILKKKGAAKAALW

>Streptococcus-pneumoniae_SNN63655.1

MKSKRMEFHNKFLIVSAMLAVISWLSLGVVSFPMLAGTLGISTKAAATVVNLISAYSTVTAVISIVGAITGVGSIGSGIAATVLYILKKKGAAKAALW

>Streptococcus-pneumoniae_SNO37449.1

MKSKRMEFHNKFLIVSAMLAVISWLSLGVVSFPMLAGTLGISTKAAATVVNLISAYSTVTAVISIVGAITGVGSIGSGIAATVLYILKKKGAAKAALW

>Streptococcus-pneumoniae_SNQ16414.1

MKSKRMEFHNKFLIVSAMLAVISWLSLGVVSFPMLAGTLGISTKAAATVVNLISAYSTVTAVISIVGAITGVGSIGSGIAATVLYILKKKGAAKAALW

>Streptococcus-pneumoniae_SNE91291.1

MKSKRMEFHNKFLIVSAMLAVISWLSLGVVSFPMLAGTLGISTKAAATVVNLISAYSTVTAVISIVGAITGVGSIGSGIAATVLYILKKKGAAKAALW

>Streptococcus-pneumoniae_SNO07411.1

MKSKRMEFHNKFLIVSAMLAVISWLSLGVVSFPMLAGTLGISTKAAATVVNLISAYSTVTAVISIVGAITGVGSIGSGIAATVLYILKKKGAAKAALW

>Streptococcus-pneumoniae_SNQ14446.1

MKSKRMEFHNKFLIVSAMLAVISWLSLGVVSFPMLAGTLGISTKAAATVVNLISAYSTVTAVISIVGAITGVGSIGSGIAATVLYILKKKGAAKAALW

>Streptococcus-pneumoniae_SNM06156.1

MKSKRMEFHNKFLIVSAMLAVISWLSLGVVSFPMLAGTLGISTKAAATVVNLISAYSTVTAVISIVGAITGVGSIGSGIAATVLYILKKKGAAKAALW

>Streptococcus-pneumoniae_SNM89066.1

MKSKRMEFHNKFLIVSAMLAVISWLSLGVVSFPMLAGTLGISTKAAATVVNLISAYSTVTAVISIVGAITGVGSIGSGIAATVLYILKKKGAAKAALW

>Streptococcus-pneumoniae_SNK82829.1

MKSKRMEFHNKFLIVSAMLAVISWLSLGVVSFPMLAGTLGISTKAAATVVNLISAYSTVTAVISIVGAITGVGSIGSGIAATVLYILKKKGAAKAALW

>Streptococcus-pneumoniae_SNG03766.1

MKSKRMEFHNKFLIVSAMLAVISWLSLGVVSFPMLAGTLGISTKAAATVVNLISAYSTVTAVISIVGAITGVGSIGSGIAATVLYILKKKGAAKAALW

>Streptococcus-pneumoniae_SNP64128.1

MKSKRMEFHNKFLIVSAMLAVISWLSLGVVSFPMLAGTLGISTKAAATVVNLISAYSTVTAVISIVGAITGVGSIGSGIAATVLYILKKKGAAKAALW

>Streptococcus-pneumoniae_SNI47241.1

MKSKRMEFHNKFLIVSAMLAVISWLSLGVVSFPMLAGTLGISTKAAATVVNLISAYSTVTAVISIVGAITGVGSIGSGIAATVLYILKKKGAAKAALW

>Streptococcus-pneumoniae_SNN59153.1

MKSKRMEFHNKFLIVSAMLAVISWLSLGVVSFPMLAGTLGISTKAAATVVNLISAYSTVTAVISIVGAITGVGSIGSGIAATVLYILKKKGAAKAALW

>Streptococcus-pneumoniae_SNQ23368.1

MKSKRMEFHNKFLIVSAMLAVISWLSLGVVSFPMLAGTLGISTKAAATVVNLISAYSTVTAVISIVGAITGVGSIGSGIAATVLYILKKKGAAKAALW

>Streptococcus-pneumoniae_SNN08531.1

MKSKRMEFHNKFLIVSAMLAVISWLSLGVVSFPMLAGTLGISTKAAATVVNLISAYSTVTAVISIVGAITGVGSIGSGIAATVLYILKKKGAAKAALW

>Streptococcus-pneumoniae_SNM65569.1

MKSKRMEFHNKFLIVSAMLAVISWLSLGVVSFPMLAGTLGISTKAAATVVNLISAYSTVTAVISIVGAITGVGSIGSGIAATVLYILKKKGAAKAALW

>Streptococcus-pneumoniae_SNL14722.1

MKSKRMEFHNKFLIVSAMLAVISWLSLGVVSFPMLAGTLGISTKAAATVVNLISAYSTVTAVISIVGAITGVGSIGSGIAATVLYILKKKGAAKAALW

>Streptococcus-pneumoniae_SNI61802.1

MKSKRMEFHNKFLIVSAMLAVISWLSLGVVSFPMLAGTLGISTKAAATVVNLISAYSTVTAVISIVGAITGVGSIGSGIAATVLYILKKKGAAKAALW

>Streptococcus-pneumoniae_SNO02715.1

MKSKRMEFHNKFLIVSAMLAVISWLSLGVVSFPMLAGTLGISTKAAATVVNLISAYSTVTAVISIVGAITGVGSIGSGIAATVLYILKKKGAAKAALW

>Streptococcus-pneumoniae_SNL73357.1

MKSKRMEFHNKFLIVSAMLAVISWLSLGVVSFPMLAGTLGISTKAAATVVNLISAYSTVTAVISIVGAITGVGSIGSGIAATVLYILKKKGAAKAALW

>Streptococcus-pneumoniae_SNM58821.1

MKSKRMEFHNKFLIVSAMLAVISWLSLGVVSFPMLAGTLGISTKAAATVVNLISAYSTVTAVISIVGAITGVGSIGSGIAATVLYILKKKGAAKAALW

>Streptococcus-pneumoniae_SNP60590.1

MKSKRMEFHNKFLIVSAMLAVISWLSLGVVSFPMLAGTLGISTKAAATVVNLISAYSTVTAVISIVGAITGVGSIGSGIAATVLYILKKKGAAKAALW

>Streptococcus-pneumoniae_SNP85986.1

MKSKRMEFHNKFLIVSAMLAVISWLSLGVVSFPMLAGTLGISTKAAATVVNLISAYSTVTAVISIVGAITGVGSIGSGIAATVLYILKKKGAAKAALW

>Streptococcus-pneumoniae_SNM62994.1

MKSKRMEFHNKFLIVSAMLAVISWLSLGVVSFPMLAGTLGISTKAAATVVNLISAYSTVTAVISIVGAITGVGSIGSGIAATVLYILKKKGAAKAALW

>Streptococcus-pneumoniae_SNO14532.1

MKSKRMEFHNKFLIVSAMLAVISWLSLGVVSFPMLAGTLGISTKAAATVVNLISAYSTVTAVISIVGAITGVGSIGSGIAATVLYILKKKGAAKAALW

>Streptococcus-pneumoniae_SNG62988.1

MKSKRMEFHNKFLIVSAMLAVISWLSLGVVSFPMLAGTLGISTKAAATVVNLISAYSTVTAVISIVGAITGVGSIGSGIAATVLYILKKKGAAKAALW

>Streptococcus-pneumoniae_SNH84269.1

MKSKRMEFHNKFLIVSAMLAVISWLSLGVVSFPMLAGTLGISTKAAATVVNLISAYSTVTAVISIVGAITGVGSIGSGIAATVLYILKKKGAAKAALW

>Streptococcus-pneumoniae_SNO61714.1

MKSKRMEFHNKFLIVSAMLAVISWLSLGVVSFPMLAGTLGISTKAAATVVNLISAYSTVTAVISIVGAITGVGSIGSGIAATVLYILKKKGAAKAALW

>Streptococcus-pneumoniae_SNQ00879.1

MKSKRMEFHNKFLIVSAMLAVISWLSLGVVSFPMLAGTLGISTKAAATVVNLISAYSTVTAVISIVGAITGVGSIGSGIAATVLYILKKKGAAKAALW

>Streptococcus-pneumoniae_SNN02355.1

MKSKRMEFHNKFLIVSAMLAVISWLSLGVVSFPMLAGTLGISTKAAATVVNLISAYSTVTAVISIVGAITGVGSIGSGIAATVLYILKKKGAAKAALW

>Streptococcus-pneumoniae_SNL49483.1

MKSKRMEFHNKFLIVSAMLAVISWLSLGVVSFPMLAGTLGISTKAAATVVNLISAYSTVTAVISIVGAITGVGSIGSGIAATVLYILKKKGAAKAALW

>Streptococcus-pneumoniae_SNG97550.1

MKSKRMEFHNKFLIVSAMLAVISWLSLGVVSFPMLAGTLGISTKAAATVVNLISAYSTVTAVISIVGAITGVGSIGSGIAATVLYILKKKGAAKAALW

>Streptococcus-pneumoniae_SNJ42244.1

MKSKRMEFHNKFLIVSAMLAVISWLSLGVVSFPMLAGTLGISTKAAATVVNLISAYSTVTAVISIVGAITGVGSIGSGIAATVLYILKKKGAAKAALW

>Streptococcus-pneumoniae_SNN52112.1

MKSKRMEFHNKFLIVSAMLAVISWLSLGVVSFPMLAGTLGISTKAAATVVNLISAYSTVTAVISIVGAITGVGSIGSGIAATVLYILKKKGAAKAALW

>Streptococcus-pneumoniae_SNK05042.1

MKSKRMEFHNKFLIVSAMLAVISWLSLGVVSFPMLAGTLGISTKAAATVVNLISAYSTVTAVISIVGAITGVGSIGSGIAATVLYILKKKGAAKAALW

>Streptococcus-pneumoniae_SNK98368.1

MKSKRMEFHNKFLIVSAMLAVISWLSLGVVSFPMLAGTLGISTKAAATVVNLISAYSTVTAVISIVGAITGVGSIGSGIAATVLYILKKKGAAKAALW

>Streptococcus-pneumoniae_SNO38154.1

MKSKRMEFHNKFLIVSAMLAVISWLSLGVVSFPMLAGTLGISTKAAATVVNLISAYSTVTAVISIVGAITGVGSIGSGIAATVLYILKKKGAAKAALW

>Streptococcus-pneumoniae_SNI57253.1

MKSKRMEFHNKFLIVSAMLAVISWLSLGVVSFPMLAGTLGISTKAAATVVNLISAYSTVTAVISIVGAITGVGSIGSGIAATVLYILKKKGAAKAALW

>Streptococcus-pneumoniae_SNF57749.1

MKSKRMEFHNKFLIVSAMLAVISWLSLGVVSFPMLAGTLGISTKAAATVVNLISAYSTVTAVISIVGAITGVGSIGSGIAATVLYILKKKGAAKAALW

>Streptococcus-pneumoniae_SNI46523.1

MKSKRMEFHNKFLIVSAMLAVISWLSLGVVSFPMLAGTLGISTKAAATVVNLISAYSTVTAVISIVGAITGVGSIGSGIAATVLYILKKKGAAKAALW

>Streptococcus-pneumoniae_SNN17145.1

MKSKRMEFHNKFLIVSAMLAVISWLSLGVVSFPMLAGTLGISTKAAATVVNLISAYSTVTAVISIVGAITGVGSIGSGIAATVLYILKKKGAAKAALW

>Streptococcus-pneumoniae_SNL32708.1

MKSKRMEFHNKFLIVSAMLAVISWLSLGVVSFPMLAGTLGISTKAAATVVNLISAYSTVTAVISIVGAITGVGSIGSGIAATVLYILKKKGAAKAALW

>Streptococcus-pneumoniae_SNO59604.1

MKSKRMEFHNKFLIVSAMLAVISWLSLGVVSFPMLAGTLGISTKAAATVVNLISAYSTVTAVISIVGAITGVGSIGSGIAATVLYILKKKGAAKAALW

>Streptococcus-pneumoniae_SNO81374.1

MKSKRMEFHNKFLIVSAMLAVISWLSLGVVSFPMLAGTLGISTKAAATVVNLISAYSTVTAVISIVGAITGVGSIGSGIAATVLYILKKKGAAKAALW

>Streptococcus-pneumoniae_SNF45707.1

MKSKRMEFHNKFLIVSAMLAVISWLSLGVVSFPMLAGTLGISTKAAATVVNLISAYSTVTAVISIVGAITGVGSIGSGIAATVLYILKKKGAAKAALW

>Streptococcus-pneumoniae_SNL62892.1

MKSKRMEFHNKFLIVSAMLAVISWLSLGVVSFPMLAGTLGISTKAAATVVNLISAYSTVTAVISIVGAITGVGSIGSGIAATVLYILKKKGAAKAALW

>Streptococcus-pneumoniae_SNG63356.1

MKSKRMEFHNKFLIVSAMLAVISWLSLGVVSFPMLAGTLGISTKAAATVVNLISAYSTVTAVISIVGAITGVGSIGSGIAATVLYILKKKGAAKAALW

>Streptococcus-pneumoniae_SNK30189.1

MKSKRMEFHNKFLIVSAMLAVISWLSLGVVSFPMLAGTLGISTKAAATVVNLISAYSTVTAVISIVGAITGVGSIGSGIAATVLYILKKKGAAKAALW

>Streptococcus-pneumoniae_SNH96793.1

MKSKRMEFHNKFLIVSAMLAVISWLSLGVVSFPMLAGTLGISTKAAATVVNLISAYSTVTAVISIVGAITGVGSIGSGIAATVLYILKKKGAAKAALW

>Streptococcus-pneumoniae_SNP09259.1

MKSKRMEFHNKFLIVSAMLAVISWLSLGVVSFPMLAGTLGISTKAAATVVNLISAYSTVTAVISIVGAITGVGSIGSGIAATVLYILKKKGAAKAALW

>Streptococcus-pneumoniae_SNN47188.1

MKSKRMEFHNKFLIVSAMLAVISWLSLGVVSFPMLAGTLGISTKAAATVVNLISAYSTVTAVISIVGAITGVGSIGSGIAATVLYILKKKGAAKAALW

>Streptococcus-pneumoniae_SNI80073.1

MKSKRMEFHNKFLIVSAMLAVISWLSLGVVSFPMLAGTLGISTKAAATVVNLISAYSTVTAVISIVGAITGVGSIGSGIAATVLYILKKKGAAKAALW

>Streptococcus-pneumoniae_SNQ02443.1

MKSKRMEFHNKFLIVSAMLAVISWLSLGVVSFPMLAGTLGISTKAAATVVNLISAYSTVTAVISIVGAITGVGSIGSGIAATVLYILKKKGAAKAALW

>Streptococcus-pneumoniae_SNN01981.1

MKSKRMEFHNKFLIVSAMLAVISWLSLGVVSFPMLAGTLGISTKAAATVVNLISAYSTVTAVISIVGAITGVGSIGSGIAATVLYILKKKGAAKAALW

>Streptococcus-pneumoniae_SNP82601.1

MKSKRMEFHNKFLIVSAMLAVISWLSLGVVSFPMLAGTLGISTKAAATVVNLISAYSTVTAVISIVGAITGVGSIGSGIAATVLYILKKKGAAKAALW

>Streptococcus-pneumoniae_SNH08013.1

MKSKRMEFHNKFLIVSAMLAVISWLSLGVVSFPMLAGTLGISTKAAATVVNLISAYSTVTAVISIVGAITGVGSIGSGIAATVLYILKKKGAAKAALW

>Streptococcus-pneumoniae_SNL04623.1

MKSKRMEFHNKFLIVSAMLAVISWLSLGVVSFPMLAGTLGISTKAAATVVNLISAYSTVTAVISIVGAITGVGSIGSGIAATVLYILKKKGAAKAALW

>Streptococcus-pneumoniae_SNF52530.1

MKSKRMEFHNKFLIVSAMLAVISWLSLGVVSFPMLAGTLGISTKAAATVVNLISAYSTVTAVISIVGAITGVGSIGSGIAATVLYILKKKGAAKAALW

>Streptococcus-pneumoniae_SNK42950.1

MKSKRMEFHNKFLIVSAMLAVISWLSLGVVSFPMLAGTLGISTKAAATVVNLISAYSTVTAVISIVGAITGVGSIGSGIAATVLYILKKKGAAKAALW

>Streptococcus-pneumoniae_SNK17536.1

MKSKRMEFHNKFLIVSAMLAVISWLSLGVVSFPMLAGTLGISTKAAATVVNLISAYSTVTAVISIVGAITGVGSIGSGIAATVLYILKKKGAAKAALW

>Streptococcus-pneumoniae_SNE86820.1

MKSKRMEFHNKFLIVSAMLAVISWLSLGVVSFPMLAGTLGISTKAAATVVNLISAYSTVTAVISIVGAITGVGSIGSGIAATVLYILKKKGAAKAALW

>Streptococcus-pneumoniae_SNK65717.1

MKSKRMEFHNKFLIVSAMLAVISWLSLGVVSFPMLAGTLGISTKAAATVVNLISAYSTVTAVISIVGAITGVGSIGSGIAATVLYILKKKGAAKAALW

>Streptococcus-pneumoniae_SNH52050.1

MKSKRMEFHNKFLIVSAMLAVISWLSLGVVSFPMLAGTLGISTKAAATVVNLISAYSTVTAVISIVGAITGVGSIGSGIAATVLYILKKKGAAKAALW

>Streptococcus-pneumoniae_SNH07262.1

MKSKRMEFHNKFLIVSAMLAVISWLSLGVVSFPMLAGTLGISTKAAATVVNLISAYSTVTAVISIVGAITGVGSIGSGIAATVLYILKKKGAAKAALW

>Streptococcus-pneumoniae_SNP11666.1

MKSKRMEFHNKFLIVSAMLAVISWLSLGVVSFPMLAGTLGISTKAAATVVNLISAYSTVTAVISIVGAITGVGSIGSGIAATVLYILKKKGAAKAALW

>Streptococcus-pneumoniae_SNK05864.1

MKSKRMEFHNKFLIVSAMLAVISWLSLGVVSFPMLAGTLGISTKAAATVVNLISAYSTVTAVISIVGAITGVGSIGSGIAATVLYILKKKGAAKAALW

>Streptococcus-pneumoniae_SNF42264.1

MKSKRMEFHNKFLIVSAMLAVISWLSLGVVSFPMLAGTLGISTKAAATVVNLISAYSTVTAVISIVGAITGVGSIGSGIAATVLYILKKKGAAKAALW

>Streptococcus-pneumoniae_SNJ00077.1

MKSKRMEFHNKFLIVSAMLAVISWLSLGVVSFPMLAGTLGISTKAAATVVNLISAYSTVTAVISIVGAITGVGSIGSGIAATVLYILKKKGAAKAALW

>Streptococcus-pneumoniae_SNG33049.1

MKSKRMEFHNKFLIVSAMLAVISWLSLGVVSFPMLAGTLGISTKAAATVVNLISAYSTVTAVISIVGAITGVGSIGSGIAATVLYILKKKGAAKAALW

>Streptococcus-pneumoniae_SNP31251.1

MKSKRMEFHNKFLIVSAMLAVISWLSLGVVSFPMLAGTLGISTKAAATVVNLISAYSTVTAVISIVGAITGVGSIGSGIAATVLYILKKKGAAKAALW

>Streptococcus-pneumoniae_SNF60885.1

MKSKRMEFHNKFLIVSAMLAVISWLSLGVVSFPMLAGTLGISTKAAATVVNLISAYSTVTAVISIVGAITGVGSIGSGIAATVLYILKKKGAAKAALW

>Streptococcus-pneumoniae_SNG85630.1

MKSKRMEFHNKFLIVSAMLAVISWLSLGVVSFPMLAGTLGISTKAAATVVNLISAYSTVTAVISIVGAITGVGSIGSGIAATVLYILKKKGAAKAALW

>Streptococcus-pneumoniae_SNF63065.1

MKSKRMEFHNKFLIVSAMLAVISWLSLGVVSFPMLAGTLGISTKAAATVVNLISAYSTVTAVISIVGAITGVGSIGSGIAATVLYILKKKGAAKAALW

>Streptococcus-pneumoniae_SNM90765.1

MKSKRMEFHNKFLIVSAMLAVISWLSLGVVSFPMLAGTLGISTKAAATVVNLISAYSTVTAVISIVGAITGVGSIGSGIAATVLYILKKKGAAKAALW

>Streptococcus-pneumoniae_SNE30172.1

MKSKRMEFHNKFLIVSAMLAVISWLSLGVVSFPMLAGTLGISTKAAATVVNLISAYSTVTAVISIVGAITGVGSIGSGIAATVLYILKKKGAAKAALW

>Streptococcus-pneumoniae_SNI32624.1

MKSKRMEFHNKFLIVSAMLAVISWLSLGVVSFPMLAGTLGISTKAAATVVNLISAYSTVTAVISIVGAITGVGSIGSGIAATVLYILKKKGAAKAALW

>Streptococcus-pneumoniae_SNH78724.1

MKSKRMEFHNKFLIVSAMLAVISWLSLGVVSFPMLAGTLGISTKAAATVVNLISAYSTVTAVISIVGAITGVGSIGSGIAATVLYILKKKGAAKAALW

>Streptococcus-pneumoniae_SNP74318.1

MKSKRMEFHNKFLIVSAMLAVISWLSLGVVSFPMLAGTLGISTKAAATVVNLISAYSTVTAVISIVGAITGVGSIGSGIAATVLYILKKKGAAKAALW

>Streptococcus-pneumoniae_SNP87798.1

MKSKRMEFHNKFLIVSAMLAVISWLSLGVVSFPMLAGTLGISTKAAATVVNLISAYSTVTAVISIVGAITGVGSIGSGIAATVLYILKKKGAAKAALW

>Streptococcus-pneumoniae_SNI17978.1

MKSKRMEFHNKFLIVSAMLAVISWLSLGVVSFPMLAGTLGISTKAAATVVNLISAYSTVTAVISIVGAITGVGSIGSGIAATVLYILKKKGAAKAALW

>Streptococcus-pneumoniae_SNK41036.1

MKSKRMEFHNKFLIVSAMLAVISWLSLGVVSFPMLAGTLGISTKAAATVVNLISAYSTVTAVISIVGAITGVGSIGSGIAATVLYILKKKGAAKAALW

>Streptococcus-pneumoniae_SNH03011.1

MKSKRMEFHNKFLIVSAMLAVISWLSLGVVSFPMLAGTLGISTKAAATVVNLISAYSTVTAVISIVGAITGVGSIGSGIAATVLYILKKKGAAKAALW

>Streptococcus-pneumoniae_SNI76970.1

MKSKRMEFHNKFLIVSAMLAVISWLSLGVVSFPMLAGTLGISTKAAATVVNLISAYSTVTAVISIVGAITGVGSIGSGIAATVLYILKKKGAAKAALW

>Streptococcus-pneumoniae_SNO67450.1

MKSKRMEFHNKFLIVSAMLAVISWLSLGVVSFPMLAGTLGISTKAAATVVNLISAYSTVTAVISIVGAITGVGSIGSGIAATVLYILKKKGAAKAALW

>Streptococcus-pneumoniae_SNJ03549.1

MKSKRMEFHNKFLIVSAMLAVISWLSLGVVSFPMLAGTLGISTKAAATVVNLISAYSTVTAVISIVGAITGVGSIGSGIAATVLYILKKKGAAKAALW

>Streptococcus-pneumoniae_SNG13807.1

MKSKRMEFHNKFLIVSAMLAVISWLSLGVVSFPMLAGTLGISTKAAATVVNLISAYSTVTAVISIVGAITGVGSIGSGIAATVLYILKKKGAAKAALW

>Streptococcus-pneumoniae_SNM55183.1

MKSKRMEFHNKFLIVSAMLAVISWLSLGVVSFPMLAGTLGISTKAAATVVNLISAYSTVTAVISIVGAITGVGSIGSGIAATVLYILKKKGAAKAALW

>Streptococcus-pneumoniae_SNE07396.1

MKSKRMEFHNKFLIVSAMLAVISWLSLGVVSFPMLAGTLGISTKAAATVVNLISAYSTVTAVISIVGAITGVGSIGSGIAATVLYILKKKGAAKAALW

>Streptococcus-pneumoniae_SNG64216.1

MKSKRMEFHNKFLIVSAMLAVISWLSLGVVSFPMLAGTLGISTKAAATVVNLISAYSTVTAVISIVGAITGVGSIGSGIAATVLYILKKKGAAKAALW

>Streptococcus-pneumoniae_SNM34453.1

MKSKRMEFHNKFLIVSAMLAVISWLSLGVVSFPMLAGTLGISTKAAATVVNLISAYSTVTAVISIVGAITGVGSIGSGIAATVLYILKKKGAAKAALW

>Streptococcus-pneumoniae_SNO09932.1

MKSKRMEFHNKFLIVSAMLAVISWLSLGVVSFPMLAGTLGISTKAAATVVNLISAYSTVTAVISIVGAITGVGSIGSGIAATVLYILKKKGAAKAALW

>Streptococcus-pneumoniae_SNL55164.1

MKSKRMEFHNKFLIVSAMLAVISWLSLGVVSFPMLAGTLGISTKAAATVVNLISAYSTVTAVISIVGAITGVGSIGSGIAATVLYILKKKGAAKAALW

>Streptococcus-pneumoniae_SNE97249.1

MKSKRMEFHNKFLIVSAMLAVISWLSLGVVSFPMLAGTLGISTKAAATVVNLISAYSTVTAVISIVGAITGVGSIGSGIAATVLYILKKKGAAKAALW

>Streptococcus-pneumoniae_SNN74271.1

MKSKRMEFHNKFLIVSAMLAVISWLSLGVVSFPMLAGTLGISTKAAATVVNLISAYSTVTAVISIVGAITGVGSIGSGIAATVLYILKKKGAAKAALW

>Streptococcus-pneumoniae_SNO09843.1

MKSKRMEFHNKFLIVSAMLAVISWLSLGVVSFPMLAGTLGISTKAAATVVNLISAYSTVTAVISIVGAITGVGSIGSGIAATVLYILKKKGAAKAALW

>Streptococcus-pneumoniae_SNP13332.1

MKSKRMEFHNKFLIVSAMLAVISWLSLGVVSFPMLAGTLGISTKAAATVVNLISAYSTVTAVISIVGAITGVGSIGSGIAATVLYILKKKGAAKAALW

>Streptococcus-pneumoniae_SNM33225.1

MKSKRMEFHNKFLIVSAMLAVISWLSLGVVSFPMLAGTLGISTKAAATVVNLISAYSTVTAVISIVGAITGVGSIGSGIAATVLYILKKKGAAKAALW

>Streptococcus-pneumoniae_SNP39206.1

MKSKRMEFHNKFLIVSAMLAVISWLSLGVVSFPMLAGTLGISTKAAATVVNLISAYSTVTAVISIVGAITGVGSIGSGIAATVLYILKKKGAAKAALW

>Streptococcus-pneumoniae_SNL24677.1

MKSKRMEFHNKFLIVSAMLAVISWLSLGVVSFPMLAGTLGISTKAAATVVNLISAYSTVTAVISIVGAITGVGSIGSGIAATVLYILKKKGAAKAALW

>Streptococcus-pneumoniae_SNP55838.1

MKSKRMEFHNKFLIVSAMLAVISWLSLGVVSFPMLAGTLGISTKAAATVVNLISAYSTVTAVISIVGAITGVGSIGSGIAATVLYILKKKGAAKAALW

>Streptococcus-pneumoniae_SNI91192.1

MKSKRMEFHNKFLIVSAMLAVISWLSLGVVSFPMLAGTLGISTKAAATVVNLISAYSTVTAVISIVGAITGVGSIGSGIAATVLYILKKKGAAKAALW

>Streptococcus-pneumoniae_SNI50645.1

MKSKRMEFHNKFLIVSAMLAVISWLSLGVVSFPMLAGTLGISTKAAATVVNLISAYSTVTAVISIVGAITGVGSIGSGIAATVLYILKKKGAAKAALW

>Streptococcus-pneumoniae_SNN84814.1

MKSKRMEFHNKFLIVSAMLAVISWLSLGVVSFPMLAGTLGISTKAAATVVNLISAYSTVTAVISIVGAITGVGSIGSGIAATVLYILKKKGAAKAALW

>Streptococcus-pneumoniae_SNQ14007.1

MKSKRMEFHNKFLIVSAMLAVISWLSLGVVSFPMLAGTLGISTKAAATVVNLISAYSTVTAVISIVGAITGVGSIGSGIAATVLYILKKKGAAKAALW

>Streptococcus-pneumoniae_SNL27366.1

MKSKRMEFHNKFLIVSAMLAVISWLSLGVVSFPMLAGTLGISTKAAATVVNLISAYSTVTAVISIVGAITGVGSIGSGIAATVLYILKKKGAAKAALW

>Streptococcus-pneumoniae_SNP84266.1

MKSKRMEFHNKFLIVSAMLAVISWLSLGVVSFPMLAGTLGISTKAAATVVNLISAYSTVTAVISIVGAITGVGSIGSGIAATVLYILKKKGAAKAALW

>Streptococcus-pneumoniae_SNH99306.1

MKSKRMEFHNKFLIVSAMLAVISWLSLGVVSFPMLAGTLGISTKAAATVVNLISAYSTVTAVISIVGAITGVGSIGSGIAATVLYILKKKGAAKAALW

>Streptococcus-pneumoniae_SNK86788.1

MKSKRMEFHNKFLIVSAMLAVISWLSLGVVSFPMLAGTLGISTKAAATVVNLISAYSTVTAVISIVGAITGVGSIGSGIAATVLYILKKKGAAKAALW

>Streptococcus-pneumoniae_SND94806.1

MKSKRMEFHNKFLIVSAMLAVISWLSLGVVSFPMLAGTLGISTKAAATVVNLISAYSTVTAVISIVGAITGVGSIGSGIAATVLYILKKKGAAKAALW

>Streptococcus-pneumoniae_SNC99566.1

MKSKRMEFHNKFLIVSAMLAVISWLSLGVVSFPMLAGTLGISTKAAATVVNLISAYSTVTAVISIVGAITGVGSIGSGIAATVLYILKKKGAAKAALW

>Streptococcus-pneumoniae_SNK52751.1

MKSKRMEFHNKFLIVSAMLAVISWLSLGVVSFPMLAGTLGISTKAAATVVNLISAYSTVTAVISIVGAITGVGSIGSGIAATVLYILKKKGAAKAALW

>Streptococcus-pneumoniae_SNI42087.1

MKSKRMEFHNKFLIVSAMLAVISWLSLGVVSFPMLAGTLGISTKAAATVVNLISAYSTVTAVISIVGAITGVGSIGSGIAATVLYILKKKGAAKAALW

>Streptococcus-pneumoniae_SNM06163.1

MKSKRMEFHNKFLIVSAMLAVISWLSLGVVSFPMLAGTLGISTKAAATVVNLISAYSTVTAVISIVGAITGVGSIGSGIAATVLYILKKKGAAKAALW

>Streptococcus-pneumoniae_SNO86811.1

MKSKRMEFHNKFLIVSAMLAVISWLSLGVVSFPMLAGTLGISTKAAATVVNLISAYSTVTAVISIVGAITGVGSIGSGIAATVLYILKKKGAAKAALW

>Streptococcus-pneumoniae_SNL36401.1

MKSKRMEFHNKFLIVSAMLAVISWLSLGVVSFPMLAGTLGISTKAAATVVNLISAYSTVTAVISIVGAITGVGSIGSGIAATVLYILKKKGAAKAALW

>Streptococcus-pneumoniae_SNM37106.1

MKSKRMEFHNKFLIVSAMLAVISWLSLGVVSFPMLAGTLGISTKAAATVVNLISAYSTVTAVISIVGAITGVGSIGSGIAATVLYILKKKGAAKAALW

>Streptococcus-pneumoniae_SNO94197.1

MKSKRMEFHNKFLIVSAMLAVISWLSLGVVSFPMLAGTLGISTKAAATVVNLISAYSTVTAVISIVGAITGVGSIGSGIAATVLYILKKKGAAKAALW

>Streptococcus-pneumoniae_SNG96067.1

MKSKRMEFHNKFLIVSAMLAVISWLSLGVVSFPMLAGTLGISTKAAATVVNLISAYSTVTAVISIVGAITGVGSIGSGIAATVLYILKKKGAAKAALW

>Streptococcus-pneumoniae_SNM83434.1

MKSKRMEFHNKFLIVSAMLAVISWLSLGVVSFPMLAGTLGISTKAAATVVNLISAYSTVTAVISIVGAITGVGSIGSGIAATVLYILKKKGAAKAALW

>Streptococcus-pneumoniae_SNG31205.1

MKSKRMEFHNKFLIVSAMLAVISWLSLGVVSFPMLAGTLGISTKAAATVVNLISAYSTVTAVISIVGAITGVGSIGSGIAATVLYILKKKGAAKAALW

>Streptococcus-pneumoniae_SNG66374.1

MKSKRMEFHNKFLIVSAMLAVISWLSLGVVSFPMLAGTLGISTKAAATVVNLISAYSTVTAVISIVGAITGVGSIGSGIAATVLYILKKKGAAKAALW

>Streptococcus-pneumoniae_SNE46722.1

MKSKRMEFHNKFLIVSAMLAVISWLSLGVVSFPMLAGTLGISTKAAATVVNLISAYSTVTAVISIVGAITGVGSIGSGIAATVLYILKKKGAAKAALW

>Streptococcus-pneumoniae_SNK51802.1

MKSKRMEFHNKFLIVSAMLAVISWLSLGVVSFPMLAGTLGISTKAAATVVNLISAYSTVTAVISIVGAITGVGSIGSGIAATVLYILKKKGAAKAALW

>Streptococcus-pneumoniae_SNF55321.1

MKSKRMEFHNKFLIVSAMLAVISWLSLGVVSFPMLAGTLGISTKAAATVVNLISAYSTVTAVISIVGAITGVGSIGSGIAATVLYILKKKGAAKAALW

>Streptococcus-pneumoniae_SNI89217.1

MKSKRMEFHNKFLIVSAMLAVISWLSLGVVSFPMLAGTLGISTKAAATVVNLISAYSTVTAVISIVGAITGVGSIGSGIAATVLYILKKKGAAKAALW

>Streptococcus-pneumoniae_SNI09018.1

MKSKRMEFHNKFLIVSAMLAVISWLSLGVVSFPMLAGTLGISTKAAATVVNLISAYSTVTAVISIVGAITGVGSIGSGIAATVLYILKKKGAAKAALW

>Streptococcus-pneumoniae_SNP00150.1

MKSKRMEFHNKFLIVSAMLAVISWLSLGVVSFPMLAGTLGISTKAAATVVNLISAYSTVTAVISIVGAITGVGSIGSGIAATVLYILKKKGAAKAALW

>Streptococcus-pneumoniae_SNO33679.1

MKSKRMEFHNKFLIVSAMLAVISWLSLGVVSFPMLAGTLGISTKAAATVVNLISAYSTVTAVISIVGAITGVGSIGSGIAATVLYILKKKGAAKAALW

>Streptococcus-pneumoniae_SNM91973.1

MKSKRMEFHNKFLIVSAMLAVISWLSLGVVSFPMLAGTLGISTKAAATVVNLISAYSTVTAVISIVGAITGVGSIGSGIAATVLYILKKKGAAKAALW

>Streptococcus-pneumoniae_SNJ38927.1

MKSKRMEFHNKFLIVSAMLAVISWLSLGVVSFPMLAGTLGISTKAAATVVNLISAYSTVTAVISIVGAITGVGSIGSGIAATVLYILKKKGAAKAALW

>Streptococcus-pneumoniae_SNH83636.1

MKSKRMEFHNKFLIVSAMLAVISWLSLGVVSFPMLAGTLGISTKAAATVVNLISAYSTVTAVISIVGAITGVGSIGSGIAATVLYILKKKGAAKAALW

>Streptococcus-pneumoniae_SNL40852.1

MKSKRMEFHNKFLIVSAMLAVISWLSLGVVSFPMLAGTLGISTKAAATVVNLISAYSTVTAVISIVGAITGVGSIGSGIAATVLYILKKKGAAKAALW

>Streptococcus-pneumoniae_SNL83056.1

MKSKRMEFHNKFLIVSAMLAVISWLSLGVVSFPMLAGTLGISTKAAATVVNLISAYSTVTAVISIVGAITGVGSIGSGIAATVLYILKKKGAAKAALW

>Streptococcus-pneumoniae_SNN32976.1

MKSKRMEFHNKFLIVSAMLAVISWLSLGVVSFPMLAGTLGISTKAAATVVNLISAYSTVTAVISIVGAITGVGSIGSGIAATVLYILKKKGAAKAALW

>Streptococcus-pneumoniae_SNE81550.1

MKSKRMEFHNKFLIVSAMLAVISWLSLGVVSFPMLAGTLGISTKAAATVVNLISAYSTVTAVISIVGAITGVGSIGSGIAATVLYILKKKGAAKAALW

>Streptococcus-pneumoniae_SNM79669.1

MKSKRMEFHNKFLIVSAMLAVISWLSLGVVSFPMLAGTLGISTKAAATVVNLISAYSTVTAVISIVGAITGVGSIGSGIAATVLYILKKKGAAKAALW

>Streptococcus-pneumoniae_SNH07506.1

MKSKRMEFHNKFLIVSAMLAVISWLSLGVVSFPMLAGTLGISTKAAATVVNLISAYSTVTAVISIVGAITGVGSIGSGIAATVLYILKKKGAAKAALW

>Streptococcus-pneumoniae_SNC84109.1

MKSKRMEFHNKFLIVSAMLAVISWLSLGVVSFPMLAGTLGISTKAAATVVNLISAYSTVTAVISIVGAITGVGSIGSGIAATVLYILKKKGAAKAALW

>Streptococcus-pneumoniae_SNM79971.1

MKSKRMEFHNKFLIVSAMLAVISWLSLGVVSFPMLAGTLGISTKAAATVVNLISAYSTVTAVISIVGAITGVGSIGSGIAATVLYILKKKGAAKAALW

>Streptococcus-pneumoniae_SND85626.1

MKSKRMEFHNKFLIVSAMLAVISWLSLGVVSFPMLAGTLGISTKAAATVVNLISAYSTVTAVISIVGAITGVGSIGSGIAATVLYILKKKGAAKAALW

>Streptococcus-pneumoniae_SNG74917.1

MKSKRMEFHNKFLIVSAMLAVISWLSLGVVSFPMLAGTLGISTKAAATVVNLISAYSTVTAVISIVGAITGVGSIGSGIAATVLYILKKKGAAKAALW

>Streptococcus-pneumoniae_SND15039.1

MKSKRMEFHNKFLIVSAMLAVISWLSLGVVSFPMLAGTLGISTKAAATVVNLISAYSTVTAVISIVGAITGVGSIGSGIAATVLYILKKKGAAKAALW

>Streptococcus-pneumoniae_SNK55960.1

MKSKRMEFHNKFLIVSAMLAVISWLSLGVVSFPMLAGTLGISTKAAATVVNLISAYSTVTAVISIVGAITGVGSIGSGIAATVLYILKKKGAAKAALW

>Streptococcus-pneumoniae_SNM19510.1

MKSKRMEFHNKFLIVSAMLAVISWLSLGVVSFPMLAGTLGISTKAAATVVNLISAYSTVTAVISIVGAITGVGSIGSGIAATVLYILKKKGAAKAALW

>Streptococcus-pneumoniae_SNH83158.1

MKSKRMEFHNKFLIVSAMLAVISWLSLGVVSFPMLAGTLGISTKAAATVVNLISAYSTVTAVISIVGAITGVGSIGSGIAATVLYILKKKGAAKAALW

>Streptococcus-pneumoniae_SND76363.1

MKSKRMEFHNKFLIVSAMLAVISWLSLGVVSFPMLAGTLGISTKAAATVVNLISAYSTVTAVISIVGAITGVGSIGSGIAATVLYILKKKGAAKAALW

>Streptococcus-pneumoniae_SNO33231.1

MKSKRMEFHNKFLIVSAMLAVISWLSLGVVSFPMLAGTLGISTKAAATVVNLISAYSTVTAVISIVGAITGVGSIGSGIAATVLYILKKKGAAKAALW

>Streptococcus-pneumoniae_SNN84031.1

MKSKRMEFHNKFLIVSAMLAVISWLSLGVVSFPMLAGTLGISTKAAATVVNLISAYSTVTAVISIVGAITGVGSIGSGIAATVLYILKKKGAAKAALW

>Streptococcus-pneumoniae_SNJ72586.1

MKSKRMEFHNKFLIVSAMLAVISWLSLGVVSFPMLAGTLGISTKAAATVVNLISAYSTVTAVISIVGAITGVGSIGSGIAATVLYILKKKGAAKAALW

>Streptococcus-pneumoniae_SNN51347.1

MKSKRMEFHNKFLIVSAMLAVISWLSLGVVSFPMLAGTLGISTKAAATVVNLISAYSTVTAVISIVGAITGVGSIGSGIAATVLYILKKKGAAKAALW

>Streptococcus-pneumoniae_SNI84227.1

MKSKRMEFHNKFLIVSAMLAVISWLSLGVVSFPMLAGTLGISTKAAATVVNLISAYSTVTAVISIVGAITGVGSIGSGIAATVLYILKKKGAAKAALW

>Streptococcus-pneumoniae_SNJ66620.1

MKSKRMEFHNKFLIVSAMLAVISWLSLGVVSFPMLAGTLGISTKAAATVVNLISAYSTVTAVISIVGAITGVGSIGSGIAATVLYILKKKGAAKAALW

>Streptococcus-pneumoniae_SNF48818.1

MKSKRMEFHNKFLIVSAMLAVISWLSLGVVSFPMLAGTLGISTKAAATVVNLISAYSTVTAVISIVGAITGVGSIGSGIAATVLYILKKKGAAKAALW

>Streptococcus-pneumoniae_SNH21756.1

MKSKRMEFHNKFLIVSAMLAVISWLSLGVVSFPMLAGTLGISTKAAATVVNLISAYSTVTAVISIVGAITGVGSIGSGIAATVLYILKKKGAAKAALW

>Streptococcus-pneumoniae_SNL26336.1

MKSKRMEFHNKFLIVSAMLAVISWLSLGVVSFPMLAGTLGISTKAAATVVNLISAYSTVTAVISIVGAITGVGSIGSGIAATVLYILKKKGAAKAALW

>Streptococcus-pneumoniae_SNO75870.1

MKSKRMEFHNKFLIVSAMLAVISWLSLGVVSFPMLAGTLGISTKAAATVVNLISAYSTVTAVISIVGAITGVGSIGSGIAATVLYILKKKGAAKAALW

>Streptococcus-pneumoniae_SNH24267.1

MKSKRMEFHNKFLIVSAMLAVISWLSLGVVSFPMLAGTLGISTKAAATVVNLISAYSTVTAVISIVGAITGVGSIGSGIAATVLYILKKKGAAKAALW

>Streptococcus-pneumoniae_SNL03360.1

MKSKRMEFHNKFLIVSAMLAVISWLSLGVVSFPMLAGTLGISTKAAATVVNLISAYSTVTAVISIVGAITGVGSIGSGIAATVLYILKKKGAAKAALW

>Streptococcus-pneumoniae_SNP37513.1

MKSKRMEFHNKFLIVSAMLAVISWLSLGVVSFPMLAGTLGISTKAAATVVNLISAYSTVTAVISIVGAITGVGSIGSGIAATVLYILKKKGAAKAALW

>Streptococcus-pneumoniae_SNN73676.1

MKSKRMEFHNKFLIVSAMLAVISWLSLGVVSFPMLAGTLGISTKAAATVVNLISAYSTVTAVISIVGAITGVGSIGSGIAATVLYILKKKGAAKAALW

>Streptococcus-pneumoniae_SNP35354.1

MKSKRMEFHNKFLIVSAMLAVISWLSLGVVSFPMLAGTLGISTKAAATVVNLISAYSTVTAVISIVGAITGVGSIGSGIAATVLYILKKKGAAKAALW

>Streptococcus-pneumoniae_SNM96242.1

MKSKRMEFHNKFLIVSAMLAVISWLSLGVVSFPMLAGTLGISTKAAATVVNLISAYSTVTAVISIVGAITGVGSIGSGIAATVLYILKKKGAAKAALW

>Streptococcus-pneumoniae_SNN47253.1

MKSKRMEFHNKFLIVSAMLAVISWLSLGVVSFPMLAGTLGISTKAAATVVNLISAYSTVTAVISIVGAITGVGSIGSGIAATVLYILKKKGAAKAALW

>Streptococcus-pneumoniae_SNO74530.1

MKSKRMEFHNKFLIVSAMLAVISWLSLGVVSFPMLAGTLGISTKAAATVVNLISAYSTVTAVISIVGAITGVGSIGSGIAATVLYILKKKGAAKAALW

>Streptococcus-pneumoniae_SNK40005.1

MKSKRMEFHNKFLIVSAMLAVISWLSLGVVSFPMLAGTLGISTKAAATVVNLISAYSTVTAVISIVGAITGVGSIGSGIAATVLYILKKKGAAKAALW

>Streptococcus-pneumoniae_SNP66828.1

MKSKRMEFHNKFLIVSAMLAVISWLSLGVVSFPMLAGTLGISTKAAATVVNLISAYSTVTAVISIVGAITGVGSIGSGIAATVLYILKKKGAAKAALW

>Streptococcus-pneumoniae_SNP46624.1

MKSKRMEFHNKFLIVSAMLAVISWLSLGVVSFPMLAGTLGISTKAAATVVNLISAYSTVTAVISIVGAITGVGSIGSGIAATVLYILKKKGAAKAALW

>Streptococcus-pneumoniae_SNK55975.1

MKSKRMEFHNKFLIVSAMLAVISWLSLGVVSFPMLAGTLGISTKAAATVVNLISAYSTVTAVISIVGAITGVGSIGSGIAATVLYILKKKGAAKAALW

>Streptococcus-pneumoniae_SNF68749.1

MKSKRMEFHNKFLIVSAMLAVISWLSLGVVSFPMLAGTLGISTKAAATVVNLISAYSTVTAVISIVGAITGVGSIGSGIAATVLYILKKKGAAKAALW

>Streptococcus-pneumoniae_SNK33510.1

MKSKRMEFHNKFLIVSAMLAVISWLSLGVVSFPMLAGTLGISTKAAATVVNLISAYSTVTAVISIVGAITGVGSIGSGIAATVLYILKKKGAAKAALW

>Streptococcus-pneumoniae_SNN92158.1

MKSKRMEFHNKFLIVSAMLAVISWLSLGVVSFPMLAGTLGISTKAAATVVNLISAYSTVTAVISIVGAITGVGSIGSGIAATVLYILKKKGAAKAALW

>Streptococcus-pneumoniae_SNF91485.1

MKSKRMEFHNKFLIVSAMLAVISWLSLGVVSFPMLAGTLGISTKAAATVVNLISAYSTVTAVISIVGAITGVGSIGSGIAATVLYILKKKGAAKAALW

>Streptococcus-pneumoniae_SNL46090.1

MKSKRMEFHNKFLIVSAMLAVISWLSLGVVSFPMLAGTLGISTKAAATVVNLISAYSTVTAVISIVGAITGVGSIGSGIAATVLYILKKKGAAKAALW

>Streptococcus-pneumoniae_SNJ72879.1

MKSKRMEFHNKFLIVSAMLAVISWLSLGVVSFPMLAGTLGISTKAAATVVNLISAYSTVTAVISIVGAITGVGSIGSGIAATVLYILKKKGAAKAALW

>Streptococcus-pneumoniae_SNF25503.1

MKSKRMEFHNKFLIVSAMLAVISWLSLGVVSFPMLAGTLGISTKAAATVVNLISAYSTVTAVISIVGAITGVGSIGSGIAATVLYILKKKGAAKAALW

>Streptococcus-pneumoniae_SNE37468.1

MKSKRMEFHNKFLIVSAMLAVISWLSLGVVSFPMLAGTLGISTKAAATVVNLISAYSTVTAVISIVGAITGVGSIGSGIAATVLYILKKKGAAKAALW

>Streptococcus-pneumoniae_SNL79523.1

MKSKRMEFHNKFLIVSAMLAVISWLSLGVVSFPMLAGTLGISTKAAATVVNLISAYSTVTAVISIVGAITGVGSIGSGIAATVLYILKKKGAAKAALW

>Streptococcus-pneumoniae_SNN51510.1

MKSKRMEFHNKFLIVSAMLAVISWLSLGVVSFPMLAGTLGISTKAAATVVNLISAYSTVTAVISIVGAITGVGSIGSGIAATVLYILKKKGAAKAALW

>Streptococcus-pneumoniae_SNM02693.1

MKSKRMEFHNKFLIVSAMLAVISWLSLGVVSFPMLAGTLGISTKAAATVVNLISAYSTVTAVISIVGAITGVGSIGSGIAATVLYILKKKGAAKAALW

>Streptococcus-pneumoniae_SNH86130.1

MKSKRMEFHNKFLIVSAMLAVISWLSLGVVSFPMLAGTLGISTKAAATVVNLISAYSTVTAVISIVGAITGVGSIGSGIAATVLYILKKKGAAKAALW

>Streptococcus-pneumoniae_SNJ87122.1

MKSKRMEFHNKFLIVSAMLAVISWLSLGVVSFPMLAGTLGISTKAAATVVNLISAYSTVTAVISIVGAITGVGSIGSGIAATVLYILKKKGAAKAALW

>Streptococcus-pneumoniae_SNH95746.1

MKSKRMEFHNKFLIVSAMLAVISWLSLGVVSFPMLAGTLGISTKAAATVVNLISAYSTVTAVISIVGAITGVGSIGSGIAATVLYILKKKGAAKAALW

>Streptococcus-pneumoniae_SNK76593.1

MKSKRMEFHNKFLIVSAMLAVISWLSLGVVSFPMLAGTLGISTKAAATVVNLISAYSTVTAVISIVGAITGVGSIGSGIAATVLYILKKKGAAKAALW

>Streptococcus-pneumoniae_SNM87939.1

MKSKRMEFHNKFLIVSAMLAVISWLSLGVVSFPMLAGTLGISTKAAATVVNLISAYSTVTAVISIVGAITGVGSIGSGIAATVLYILKKKGAAKAALW

>Streptococcus-pneumoniae_SNN83129.1

MKSKRMEFHNKFLIVSAMLAVISWLSLGVVSFPMLAGTLGISTKAAATVVNLISAYSTVTAVISIVGAITGVGSIGSGIAATVLYILKKKGAAKAALW

>Streptococcus-pneumoniae_SND15130.1

MKSKRMEFHNKFLIVSAMLAVISWLSLGVVSFPMLAGTLGISTKAAATVVNLISAYSTVTAVISIVGAITGVGSIGSGIAATVLYILKKKGAAKAALW

>Streptococcus-pneumoniae_SNP42596.1

MKSKRMEFHNKFLIVSAMLAVISWLSLGVVSFPMLAGTLGISTKAAATVVNLISAYSTVTAVISIVGAITGVGSIGSGIAATVLYILKKKGAAKAALW

>Streptococcus-pneumoniae_SNK16050.1

MKSKRMEFHNKFLIVSAMLAVISWLSLGVVSFPMLAGTLGISTKAAATVVNLISAYSTVTAVISIVGAITGVGSIGSGIAATVLYILKKKGAAKAALW

>Streptococcus-pneumoniae_SNN17533.1

MKSKRMEFHNKFLIVSAMLAVISWLSLGVVSFPMLAGTLGISTKAAATVVNLISAYSTVTAVISIVGAITGVGSIGSGIAATVLYILKKKGAAKAALW

>Streptococcus-pneumoniae_SND22437.1

MKSKRMEFHNKFLIVSAMLAVISWLSLGVVSFPMLAGTLGISTKAAATVVNLISAYSTVTAVISIVGAITGVGSIGSGIAATVLYILKKKGAAKAALW

>Streptococcus-pneumoniae_SND20822.1

MKSKRMEFHNKFLIVSAMLAVISWLSLGVVSFPMLAGTLGISTKAAATVVNLISAYSTVTAVISIVGAITGVGSIGSGIAATVLYILKKKGAAKAALW

>Streptococcus-pneumoniae_SNP36624.1

MKSKRMEFHNKFLIVSAMLAVISWLSLGVVSFPMLAGTLGISTKAAATVVNLISAYSTVTAVISIVGAITGVGSIGSGIAATVLYILKKKGAAKAALW

>Streptococcus-pneumoniae_SNP17265.1

MKSKRMEFHNKFLIVSAMLAVISWLSLGVVSFPMLAGTLGISTKAAATVVNLISAYSTVTAVISIVGAITGVGSIGSGIAATVLYILKKKGAAKAALW

>Streptococcus-pneumoniae_SNJ40416.1

MKSKRMEFHNKFLIVSAMLAVISWLSLGVVSFPMLAGTLGISTKAAATVVNLISAYSTVTAVISIVGAITGVGSIGSGIAATVLYILKKKGAAKAALW

>Streptococcus-pneumoniae_SNK91818.1

MKSKRMEFHNKFLIVSAMLAVISWLSLGVVSFPMLAGTLGISTKAAATVVNLISAYSTVTAVISIVGAITGVGSIGSGIAATVLYILKKKGAAKAALW

>Streptococcus-pneumoniae_SNI84730.1

MKSKRMEFHNKFLIVSAMLAVISWLSLGVVSFPMLAGTLGISTKAAATVVNLISAYSTVTAVISIVGAITGVGSIGSGIAATVLYILKKKGAAKAALW

>Streptococcus-pneumoniae_SNL53987.1

MKSKRMEFHNKFLIVSAMLAVISWLSLGVVSFPMLAGTLGISTKAAATVVNLISAYSTVTAVISIVGAITGVGSIGSGIAATVLYILKKKGAAKAALW

>Streptococcus-pneumoniae_SNN18647.1

MKSKRMEFHNKFLIVSAMLAVISWLSLGVVSFPMLAGTLGISTKAAATVVNLISAYSTVTAVISIVGAITGVGSIGSGIAATVLYILKKKGAAKAALW

>Streptococcus-pneumoniae_SNI73411.1

MKSKRMEFHNKFLIVSAMLAVISWLSLGVVSFPMLAGTLGISTKAAATVVNLISAYSTVTAVISIVGAITGVGSIGSGIAATVLYILKKKGAAKAALW

>Streptococcus-pneumoniae_SND33261.1

MKSKRMEFHNKFLIVSAMLAVISWLSLGVVSFPMLAGTLGISTKAAATVVNLISAYSTVTAVISIVGAITGVGSIGSGIAATVLYILKKKGAAKAALW

>Streptococcus-pneumoniae_SNL52571.1

MKSKRMEFHNKFLIVSAMLAVISWLSLGVVSFPMLAGTLGISTKAAATVVNLISAYSTVTAVISIVGAITGVGSIGSGIAATVLYILKKKGAAKAALW

>Streptococcus-pneumoniae_SNG03522.1

MKSKRMEFHNKFLIVSAMLAVISWLSLGVVSFPMLAGTLGISTKAAATVVNLISAYSTVTAVISIVGAITGVGSIGSGIAATVLYILKKKGAAKAALW

>Streptococcus-pneumoniae_SND30548.1

MKSKRMEFHNKFLIVSAMLAVISWLSLGVVSFPMLAGTLGISTKAAATVVNLISAYSTVTAVISIVGAITGVGSIGSGIAATVLYILKKKGAAKAALW

>Streptococcus-pneumoniae_SNM26289.1

MKSKRMEFHNKFLIVSAMLAVISWLSLGVVSFPMLAGTLGISTKAAATVVNLISAYSTVTAVISIVGAITGVGSIGSGIAATVLYILKKKGAAKAALW

>Streptococcus-pneumoniae_SNP64433.1

MKSKRMEFHNKFLIVSAMLAVISWLSLGVVSFPMLAGTLGISTKAAATVVNLISAYSTVTAVISIVGAITGVGSIGSGIAATVLYILKKKGAAKAALW

>Streptococcus-pneumoniae_SND42943.1

MKSKRMEFHNKFLIVSAMLAVISWLSLGVVSFPMLAGTLGISTKAAATVVNLISAYSTVTAVISIVGAITGVGSIGSGIAATVLYILKKKGAAKAALW

>Streptococcus-pneumoniae_SNI12615.1

MKSKRMEFHNKFLIVSAMLAVISWLSLGVVSFPMLAGTLGISTKAAATVVNLISAYSTVTAVISIVGAITGVGSIGSGIAATVLYILKKKGAAKAALW

>Streptococcus-pneumoniae_SNN37811.1

MKSKRMEFHNKFLIVSAMLAVISWLSLGVVSFPMLAGTLGISTKAAATVVNLISAYSTVTAVISIVGAITGVGSIGSGIAATVLYILKKKGAAKAALW

>Streptococcus-pneumoniae_SNF61169.1

MKSKRMEFHNKFLIVSAMLAVISWLSLGVVSFPMLAGTLGISTKAAATVVNLISAYSTVTAVISIVGAITGVGSIGSGIAATVLYILKKKGAAKAALW

>Streptococcus-pneumoniae_SNL81512.1

MKSKRMEFHNKFLIVSAMLAVISWLSLGVVSFPMLAGTLGISTKAAATVVNLISAYSTVTAVISIVGAITGVGSIGSGIAATVLYILKKKGAAKAALW

>Streptococcus-pneumoniae_SNM54081.1

MKSKRMEFHNKFLIVSAMLAVISWLSLGVVSFPMLAGTLGISTKAAATVVNLISAYSTVTAVISIVGAITGVGSIGSGIAATVLYILKKKGAAKAALW

>Streptococcus-pneumoniae_SNP89412.1

MKSKRMEFHNKFLIVSAMLAVISWLSLGVVSFPMLAGTLGISTKAAATVVNLISAYSTVTAVISIVGAITGVGSIGSGIAATVLYILKKKGAAKAALW

>Streptococcus-pneumoniae_SNK15625.1

MKSKRMEFHNKFLIVSAMLAVISWLSLGVVSFPMLAGTLGISTKAAATVVNLISAYSTVTAVISIVGAITGVGSIGSGIAATVLYILKKKGAAKAALW

>Streptococcus-pneumoniae_SNO08064.1

MKSKRMEFHNKFLIVSAMLAVISWLSLGVVSFPMLAGTLGISTKAAATVVNLISAYSTVTAVISIVGAITGVGSIGSGIAATVLYILKKKGAAKAALW

>Streptococcus-pneumoniae_SNL22024.1

MKSKRMEFHNKFLIVSAMLAVISWLSLGVVSFPMLAGTLGISTKAAATVVNLISAYSTVTAVISIVGAITGVGSIGSGIAATVLYILKKKGAAKAALW

>Streptococcus-pneumoniae_SNO41964.1

MKSKRMEFHNKFLIVSAMLAVISWLSLGVVSFPMLAGTLGISTKAAATVVNLISAYSTVTAVISIVGAITGVGSIGSGIAATVLYILKKKGAAKAALW

>Streptococcus-pneumoniae_SNM22181.1

MKSKRMEFHNKFLIVSAMLAVISWLSLGVVSFPMLAGTLGISTKAAATVVNLISAYSTVTAVISIVGAITGVGSIGSGIAATVLYILKKKGAAKAALW

>Streptococcus-pneumoniae_SNJ58603.1

MKSKRMEFHNKFLIVSAMLAVISWLSLGVVSFPMLAGTLGISTKAAATVVNLISAYSTVTAVISIVGAITGVGSIGSGIAATVLYILKKKGAAKAALW

>Streptococcus-pneumoniae_SNN54353.1

MKSKRMEFHNKFLIVSAMLAVISWLSLGVVSFPMLAGTLGISTKAAATVVNLISAYSTVTAVISIVGAITGVGSIGSGIAATVLYILKKKGAAKAALW

>Streptococcus-pneumoniae_SNF35075.1

MKSKRMEFHNKFLIVSAMLAVISWLSLGVVSFPMLAGTLGISTKAAATVVNLISAYSTVTAVISIVGAITGVGSIGSGIAATVLYILKKKGAAKAALW

>Streptococcus-pneumoniae_SNL03926.1

MKSKRMEFHNKFLIVSAMLAVISWLSLGVVSFPMLAGTLGISTKAAATVVNLISAYSTVTAVISIVGAITGVGSIGSGIAATVLYILKKKGAAKAALW

>Streptococcus-pneumoniae_SNP61038.1

MKSKRMEFHNKFLIVSAMLAVISWLSLGVVSFPMLAGTLGISTKAAATVVNLISAYSTVTAVISIVGAITGVGSIGSGIAATVLYILKKKGAAKAALW

>Streptococcus-pneumoniae_SNK05929.1

MKSKRMEFHNKFLIVSAMLAVISWLSLGVVSFPMLAGTLGISTKAAATVVNLISAYSTVTAVISIVGAITGVGSIGSGIAATVLYILKKKGAAKAALW

>Streptococcus-pneumoniae_SNJ04252.1

MKSKRMEFHNKFLIVSAMLAVISWLSLGVVSFPMLAGTLGISTKAAATVVNLISAYSTVTAVISIVGAITGVGSIGSGIAATVLYILKKKGAAKAALW

>Streptococcus-pneumoniae_SNK82815.1

MKSKRMEFHNKFLIVSAMLAVISWLSLGVVSFPMLAGTLGISTKAAATVVNLISAYSTVTAVISIVGAITGVGSIGSGIAATVLYILKKKGAAKAALW

>Streptococcus-pneumoniae_SNM53957.1

MKSKRMEFHNKFLIVSAMLAVISWLSLGVVSFPMLAGTLGISTKAAATVVNLISAYSTVTAVISIVGAITGVGSIGSGIAATVLYILKKKGAAKAALW

>Streptococcus-pneumoniae_SND32198.1

MKSKRMEFHNKFLIVSAMLAVISWLSLGVVSFPMLAGTLGISTKAAATVVNLISAYSTVTAVISIVGAITGVGSIGSGIAATVLYILKKKGAAKAALW

>Streptococcus-pneumoniae_SNJ10650.1

MKSKRMEFHNKFLIVSAMLAVISWLSLGVVSFPMLAGTLGISTKAAATVVNLISAYSTVTAVISIVGAITGVGSIGSGIAATVLYILKKKGAAKAALW

>Streptococcus-pneumoniae_SNK84063.1

MKSKRMEFHNKFLIVSAMLAVISWLSLGVVSFPMLAGTLGISTKAAATVVNLISAYSTVTAVISIVGAITGVGSIGSGIAATVLYILKKKGAAKAALW

>Streptococcus-pneumoniae_SNF24302.1

MKSKRMEFHNKFLIVSAMLAVISWLSLGVVSFPMLAGTLGISTKAAATVVNLISAYSTVTAVISIVGAITGVGSIGSGIAATVLYILKKKGAAKAALW

>Streptococcus-pneumoniae_SNE41353.1

MKSKRMEFHNKFLIVSAMLAVISWLSLGVVSFPMLAGTLGISTKAAATVVNLISAYSTVTAVISIVGAITGVGSIGSGIAATVLYILKKKGAAKAALW

>Streptococcus-pneumoniae_SND86027.1

MKSKRMEFHNKFLIVSAMLAVISWLSLGVVSFPMLAGTLGISTKAAATVVNLISAYSTVTAVISIVGAITGVGSIGSGIAATVLYILKKKGAAKAALW

>Streptococcus-pneumoniae_SNL77481.1

MKSKRMEFHNKFLIVSAMLAVISWLSLGVVSFPMLAGTLGISTKAAATVVNLISAYSTVTAVISIVGAITGVGSIGSGIAATVLYILKKKGAAKAALW

>Streptococcus-pneumoniae_SNG67319.1

MKSKRMEFHNKFLIVSAMLAVISWLSLGVVSFPMLAGTLGISTKAAATVVNLISAYSTVTAVISIVGAITGVGSIGSGIAATVLYILKKKGAAKAALW

>Streptococcus-pneumoniae_SNH94171.1

MKSKRMEFHNKFLIVSAMLAVISWLSLGVVSFPMLAGTLGISTKAAATVVNLISAYSTVTAVISIVGAITGVGSIGSGIAATVLYILKKKGAAKAALW

>Streptococcus-pneumoniae_SNL75942.1

MKSKRMEFHNKFLIVSAMLAVISWLSLGVVSFPMLAGTLGISTKAAATVVNLISAYSTVTAVISIVGAITGVGSIGSGIAATVLYILKKKGAAKAALW

>Streptococcus-pneumoniae_SNK27672.1

MKSKRMEFHNKFLIVSAMLAVISWLSLGVVSFPMLAGTLGISTKAAATVVNLISAYSTVTAVISIVGAITGVGSIGSGIAATVLYILKKKGAAKAALW

>Streptococcus-pneumoniae_SNI85967.1

MKSKRMEFHNKFLIVSAMLAVISWLSLGVVSFPMLAGTLGISTKAAATVVNLISAYSTVTAVISIVGAITGVGSIGSGIAATVLYILKKKGAAKAALW

>Streptococcus-pneumoniae_SNM18165.1

MKSKRMEFHNKFLIVSAMLAVISWLSLGVVSFPMLAGTLGISTKAAATVVNLISAYSTVTAVISIVGAITGVGSIGSGIAATVLYILKKKGAAKAALW

>Streptococcus-pneumoniae_SNN90303.1

MKSKRMEFHNKFLIVSAMLAVISWLSLGVVSFPMLAGTLGISTKAAATVVNLISAYSTVTAVISIVGAITGVGSIGSGIAATVLYILKKKGAAKAALW

>Streptococcus-pneumoniae_SNG46792.1

MKSKRMEFHNKFLIVSAMLAVISWLSLGVVSFPMLAGTLGISTKAAATVVNLISAYSTVTAVISIVGAITGVGSIGSGIAATVLYILKKKGAAKAALW

>Streptococcus-pneumoniae_SNO24850.1

MKSKRMEFHNKFLIVSAMLAVISWLSLGVVSFPMLAGTLGISTKAAATVVNLISAYSTVTAVISIVGAITGVGSIGSGIAATVLYILKKKGAAKAALW

>Streptococcus-pneumoniae_SNG63510.1

MKSKRMEFHNKFLIVSAMLAVISWLSLGVVSFPMLAGTLGISTKAAATVVNLISAYSTVTAVISIVGAITGVGSIGSGIAATVLYILKKKGAAKAALW

>Streptococcus-pneumoniae_SNK68641.1

MKSKRMEFHNKFLIVSAMLAVISWLSLGVVSFPMLAGTLGISTKAAATVVNLISAYSTVTAVISIVGAITGVGSIGSGIAATVLYILKKKGAAKAALW

>Streptococcus-pneumoniae_SNF72811.1

MKSKRMEFHNKFLIVSAMLAVISWLSLGVVSFPMLAGTLGISTKAAATVVNLISAYSTVTAVISIVGAITGVGSIGSGIAATVLYILKKKGAAKAALW

>Streptococcus-pneumoniae_SND30094.1

MKSKRMEFHNKFLIVSAMLAVISWLSLGVVSFPMLAGTLGISTKAAATVVNLISAYSTVTAVISIVGAITGVGSIGSGIAATVLYILKKKGAAKAALW

>Streptococcus-pneumoniae_SNF60639.1

MKSKRMEFHNKFLIVSAMLAVISWLSLGVVSFPMLAGTLGISTKAAATVVNLISAYSTVTAVISIVGAITGVGSIGSGIAATVLYILKKKGAAKAALW

>Streptococcus-pneumoniae_SNK33631.1

MKSKRMEFHNKFLIVSAMLAVISWLSLGVVSFPMLAGTLGISTKAAATVVNLISAYSTVTAVISIVGAITGVGSIGSGIAATVLYILKKKGAAKAALW

>Streptococcus-pneumoniae_SNP82774.1

MKSKRMEFHNKFLIVSAMLAVISWLSLGVVSFPMLAGTLGISTKAAATVVNLISAYSTVTAVISIVGAITGVGSIGSGIAATVLYILKKKGAAKAALW

>Streptococcus-pneumoniae_SNJ71807.1

MKSKRMEFHNKFLIVSAMLAVISWLSLGVVSFPMLAGTLGISTKAAATVVNLISAYSTVTAVISIVGAITGVGSIGSGIAATVLYILKKKGAAKAALW

>Streptococcus-pneumoniae_SNH58455.1

MKSKRMEFHNKFLIVSAMLAVISWLSLGVVSFPMLAGTLGISTKAAATVVNLISAYSTVTAVISIVGAITGVGSIGSGIAATVLYILKKKGAAKAALW

>Streptococcus-pneumoniae_SNG61487.1

MKSKRMEFHNKFLIVSAMLAVISWLSLGVVSFPMLAGTLGISTKAAATVVNLISAYSTVTAVISIVGAITGVGSIGSGIAATVLYILKKKGAAKAALW

>Streptococcus-pneumoniae_SNN06474.1

MKSKRMEFHNKFLIVSAMLAVISWLSLGVVSFPMLAGTLGISTKAAATVVNLISAYSTVTAVISIVGAITGVGSIGSGIAATVLYILKKKGAAKAALW

>Streptococcus-pneumoniae_SNI01012.1

MKSKRMEFHNKFLIVSAMLAVISWLSLGVVSFPMLAGTLGISTKAAATVVNLISAYSTVTAVISIVGAITGVGSIGSGIAATVLYILKKKGAAKAALW

>Streptococcus-pneumoniae_SNE52541.1

MKSKRMEFHNKFLIVSAMLAVISWLSLGVVSFPMLAGTLGISTKAAATVVNLISAYSTVTAVISIVGAITGVGSIGSGIAATVLYILKKKGAAKAALW

>Streptococcus-pneumoniae_SNO14620.1

MKSKRMEFHNKFLIVSAMLAVISWLSLGVVSFPMLAGTLGISTKAAATVVNLISAYSTVTAVISIVGAITGVGSIGSGIAATVLYILKKKGAAKAALW

>Streptococcus-pneumoniae_SNJ83273.1

MKSKRMEFHNKFLIVSAMLAVISWLSLGVVSFPMLAGTLGISTKAAATVVNLISAYSTVTAVISIVGAITGVGSIGSGIAATVLYILKKKGAAKAALW

>Streptococcus-pneumoniae_SNE83704.1

MKSKRMEFHNKFLIVSAMLAVISWLSLGVVSFPMLAGTLGISTKAAATVVNLISAYSTVTAVISIVGAITGVGSIGSGIAATVLYILKKKGAAKAALW

>Streptococcus-pneumoniae_SNN35665.1

MKSKRMEFHNKFLIVSAMLAVISWLSLGVVSFPMLAGTLGISTKAAATVVNLISAYSTVTAVISIVGAITGVGSIGSGIAATVLYILKKKGAAKAALW

>Streptococcus-pneumoniae_SNQ06598.1

MKSKRMEFHNKFLIVSAMLAVISWLSLGVVSFPMLAGTLGISTKAAATVVNLISAYSTVTAVISIVGAITGVGSIGSGIAATVLYILKKKGAAKAALW

>Streptococcus-pneumoniae_SNE43459.1

MKSKRMEFHNKFLIVSAMLAVISWLSLGVVSFPMLAGTLGISTKAAATVVNLISAYSTVTAVISIVGAITGVGSIGSGIAATVLYILKKKGAAKAALW

>Streptococcus-pneumoniae_SNG56857.1

MKSKRMEFHNKFLIVSAMLAVISWLSLGVVSFPMLAGTLGISTKAAATVVNLISAYSTVTAVISIVGAITGVGSIGSGIAATVLYILKKKGAAKAALW

>Streptococcus-pneumoniae_SNM69602.1

MKSKRMEFHNKFLIVSAMLAVISWLSLGVVSFPMLAGTLGISTKAAATVVNLISAYSTVTAVISIVGAITGVGSIGSGIAATVLYILKKKGAAKAALW

>Streptococcus-pneumoniae_SNJ76553.1

MKSKRMEFHNKFLIVSAMLAVISWLSLGVVSFPMLAGTLGISTKAAATVVNLISAYSTVTAVISIVGAITGVGSIGSGIAATVLYILKKKGAAKAALW

>Streptococcus-pneumoniae_SND31920.1

MKSKRMEFHNKFLIVSAMLAVISWLSLGVVSFPMLAGTLGISTKAAATVVNLISAYSTVTAVISIVGAITGVGSIGSGIAATVLYILKKKGAAKAALW

>Streptococcus-pneumoniae_SND28388.1

MKSKRMEFHNKFLIVSAMLAVISWLSLGVVSFPMLAGTLGISTKAAATVVNLISAYSTVTAVISIVGAITGVGSIGSGIAATVLYILKKKGAAKAALW

>Streptococcus-pneumoniae_SNO86448.1

MKSKRMEFHNKFLIVSAMLAVISWLSLGVVSFPMLAGTLGISTKAAATVVNLISAYSTVTAVISIVGAITGVGSIGSGIAATVLYILKKKGAAKAALW

>Streptococcus-pneumoniae_SNF43583.1

MKSKRMEFHNKFLIVSAMLAVISWLSLGVVSFPMLAGTLGISTKAAATVVNLISAYSTVTAVISIVGAITGVGSIGSGIAATVLYILKKKGAAKAALW

>Streptococcus-pneumoniae_SNF71889.1

MKSKRMEFHNKFLIVSAMLAVISWLSLGVVSFPMLAGTLGISTKAAATVVNLISAYSTVTAVISIVGAITGVGSIGSGIAATVLYILKKKGAAKAALW

>Streptococcus-pneumoniae_SNJ31527.1

MKSKRMEFHNKFLIVSAMLAVISWLSLGVVSFPMLAGTLGISTKAAATVVNLISAYSTVTAVISIVGAITGVGSIGSGIAATVLYILKKKGAAKAALW

>Streptococcus-pneumoniae_SNG68903.1

MKSKRMEFHNKFLIVSAMLAVISWLSLGVVSFPMLAGTLGISTKAAATVVNLISAYSTVTAVISIVGAITGVGSIGSGIAATVLYILKKKGAAKAALW

>Streptococcus-pneumoniae_SNK09557.1

MKSKRMEFHNKFLIVSAMLAVISWLSLGVVSFPMLAGTLGISTKAAATVVNLISAYSTVTAVISIVGAITGVGSIGSGIAATVLYILKKKGAAKAALW

>Streptococcus-pneumoniae_SNE48098.1

MKSKRMEFHNKFLIVSAMLAVISWLSLGVVSFPMLAGTLGISTKAAATVVNLISAYSTVTAVISIVGAITGVGSIGSGIAATVLYILKKKGAAKAALW

>Streptococcus-pneumoniae_SNE86444.1

MKSKRMEFHNKFLIVSAMLAVISWLSLGVVSFPMLAGTLGISTKAAATVVNLISAYSTVTAVISIVGAITGVGSIGSGIAATVLYILKKKGAAKAALW

>Streptococcus-pneumoniae_SNJ71943.1

MKSKRMEFHNKFLIVSAMLAVISWLSLGVVSFPMLAGTLGISTKAAATVVNLISAYSTVTAVISIVGAITGVGSIGSGIAATVLYILKKKGAAKAALW

>Streptococcus-pneumoniae_SNK47221.1

MKSKRMEFHNKFLIVSAMLAVISWLSLGVVSFPMLAGTLGISTKAAATVVNLISAYSTVTAVISIVGAITGVGSIGSGIAATVLYILKKKGAAKAALW

>Streptococcus-pneumoniae_SNK12097.1

MKSKRMEFHNKFLIVSAMLAVISWLSLGVVSFPMLAGTLGISTKAAATVVNLISAYSTVTAVISIVGAITGVGSIGSGIAATVLYILKKKGAAKAALW

>Streptococcus-pneumoniae_SNI96892.1

MKSKRMEFHNKFLIVSAMLAVISWLSLGVVSFPMLAGTLGISTKAAATVVNLISAYSTVTAVISIVGAITGVGSIGSGIAATVLYILKKKGAAKAALW

>Streptococcus-pneumoniae_SNK23708.1

MKSKRMEFHNKFLIVSAMLAVISWLSLGVVSFPMLAGTLGISTKAAATVVNLISAYSTVTAVISIVGAITGVGSIGSGIAATVLYILKKKGAAKAALW

>Streptococcus-pneumoniae_SNN07187.1

MKSKRMEFHNKFLIVSAMLAVISWLSLGVVSFPMLAGTLGISTKAAATVVNLISAYSTVTAVISIVGAITGVGSIGSGIAATVLYILKKKGAAKAALW

>Streptococcus-pneumoniae_SNE96449.1

MKSKRMEFHNKFLIVSAMLAVISWLSLGVVSFPMLAGTLGISTKAAATVVNLISAYSTVTAVISIVGAITGVGSIGSGIAATVLYILKKKGAAKAALW

>Streptococcus-pneumoniae_SNE49747.1

MKSKRMEFHNKFLIVSAMLAVISWLSLGVVSFPMLAGTLGISTKAAATVVNLISAYSTVTAVISIVGAITGVGSIGSGIAATVLYILKKKGAAKAALW

>Streptococcus-pneumoniae_SNI27683.1

MKSKRMEFHNKFLIVSAMLAVISWLSLGVVSFPMLAGTLGISTKAAATVVNLISAYSTVTAVISIVGAITGVGSIGSGIAATVLYILKKKGAAKAALW

>Streptococcus-pneumoniae_SNH15861.1

MKSKRMEFHNKFLIVSAMLAVISWLSLGVVSFPMLAGTLGISTKAAATVVNLISAYSTVTAVISIVGAITGVGSIGSGIAATVLYILKKKGAAKAALW

>Streptococcus-pneumoniae_SNG67119.1

MKSKRMEFHNKFLIVSAMLAVISWLSLGVVSFPMLAGTLGISTKAAATVVNLISAYSTVTAVISIVGAITGVGSIGSGIAATVLYILKKKGAAKAALW

>Streptococcus-pneumoniae_SNG06128.1

MKSKRMEFHNKFLIVSAMLAVISWLSLGVVSFPMLAGTLGISTKAAATVVNLISAYSTVTAVISIVGAITGVGSIGSGIAATVLYILKKKGAAKAALW

>Streptococcus-pneumoniae_SNE43521.1

MKSKRMEFHNKFLIVSAMLAVISWLSLGVVSFPMLAGTLGISTKAAATVVNLISAYSTVTAVISIVGAITGVGSIGSGIAATVLYILKKKGAAKAALW

>Streptococcus-pneumoniae_SNO42103.1

MKSKRMEFHNKFLIVSAMLAVISWLSLGVVSFPMLAGTLGISTKAAATVVNLISAYSTVTAVISIVGAITGVGSIGSGIAATVLYILKKKGAAKAALW

>Streptococcus-pneumoniae_SNG99009.1

MKSKRMEFHNKFLIVSAMLAVISWLSLGVVSFPMLAGTLGISTKAAATVVNLISAYSTVTAVISIVGAITGVGSIGSGIAATVLYILKKKGAAKAALW

>Streptococcus-pneumoniae_SNF19639.1

MKSKRMEFHNKFLIVSAMLAVISWLSLGVVSFPMLAGTLGISTKAAATVVNLISAYSTVTAVISIVGAITGVGSIGSGIAATVLYILKKKGAAKAALW

>Streptococcus-pneumoniae_SNK07680.1

MKSKRMEFHNKFLIVSAMLAVISWLSLGVVSFPMLAGTLGISTKAAATVVNLISAYSTVTAVISIVGAITGVGSIGSGIAATVLYILKKKGAAKAALW

>Streptococcus-pneumoniae_SNF28993.1

MKSKRMEFHNKFLIVSAMLAVISWLSLGVVSFPMLAGTLGISTKAAATVVNLISAYSTVTAVISIVGAITGVGSIGSGIAATVLYILKKKGAAKAALW

>Streptococcus-pneumoniae_SNJ01435.1

MKSKRMEFHNKFLIVSAMLAVISWLSLGVVSFPMLAGTLGISTKAAATVVNLISAYSTVTAVISIVGAITGVGSIGSGIAATVLYILKKKGAAKAALW

>Streptococcus-pneumoniae_SNJ49884.1

MKSKRMEFHNKFLIVSAMLAVISWLSLGVVSFPMLAGTLGISTKAAATVVNLISAYSTVTAVISIVGAITGVGSIGSGIAATVLYILKKKGAAKAALW

>Streptococcus-pneumoniae_SNI73968.1

MKSKRMEFHNKFLIVSAMLAVISWLSLGVVSFPMLAGTLGISTKAAATVVNLISAYSTVTAVISIVGAITGVGSIGSGIAATVLYILKKKGAAKAALW

>Streptococcus-pneumoniae_SNK67464.1

MKSKRMEFHNKFLIVSAMLAVISWLSLGVVSFPMLAGTLGISTKAAATVVNLISAYSTVTAVISIVGAITGVGSIGSGIAATVLYILKKKGAAKAALW

>Streptococcus-pneumoniae_SNE45805.1

MKSKRMEFHNKFLIVSAMLAVISWLSLGVVSFPMLAGTLGISTKAAATVVNLISAYSTVTAVISIVGAITGVGSIGSGIAATVLYILKKKGAAKAALW

>Streptococcus-pneumoniae_SNE36744.1

MKSKRMEFHNKFLIVSAMLAVISWLSLGVVSFPMLAGTLGISTKAAATVVNLISAYSTVTAVISIVGAITGVGSIGSGIAATVLYILKKKGAAKAALW

>Streptococcus-pneumoniae_SND59913.1

MKSKRMEFHNKFLIVSAMLAVISWLSLGVVSFPMLAGTLGISTKAAATVVNLISAYSTVTAVISIVGAITGVGSIGSGIAATVLYILKKKGAAKAALW

>Streptococcus-pneumoniae_SNF06321.1

MKSKRMEFHNKFLIVSAMLAVISWLSLGVVSFPMLAGTLGISTKAAATVVNLISAYSTVTAVISIVGAITGVGSIGSGIAATVLYILKKKGAAKAALW

>Streptococcus-pneumoniae_SNJ95167.1

MKSKRMEFHNKFLIVSAMLAVISWLSLGVVSFPMLAGTLGISTKAAATVVNLISAYSTVTAVISIVGAITGVGSIGSGIAATVLYILKKKGAAKAALW

>Streptococcus-pneumoniae_SNK78127.1

MKSKRMEFHNKFLIVSAMLAVISWLSLGVVSFPMLAGTLGISTKAAATVVNLISAYSTVTAVISIVGAITGVGSIGSGIAATVLYILKKKGAAKAALW

>Streptococcus-pneumoniae_SNF98161.1

MKSKRMEFHNKFLIVSAMLAVISWLSLGVVSFPMLAGTLGISTKAAATVVNLISAYSTVTAVISIVGAITGVGSIGSGIAATVLYILKKKGAAKAALW

>Streptococcus-pneumoniae_SNO35405.1

MKSKRMEFHNKFLIVSAMLAVISWLSLGVVSFPMLAGTLGISTKAAATVVNLISAYSTVTAVISIVGAITGVGSIGSGIAATVLYILKKKGAAKAALW

>Streptococcus-pneumoniae_SNI60959.1

MKSKRMEFHNKFLIVSAMLAVISWLSLGVVSFPMLAGTLGISTKAAATVVNLISAYSTVTAVISIVGAITGVGSIGSGIAATVLYILKKKGAAKAALW

>Streptococcus-pneumoniae_SNG71536.1

MKSKRMEFHNKFLIVSAMLAVISWLSLGVVSFPMLAGTLGISTKAAATVVNLISAYSTVTAVISIVGAITGVGSIGSGIAATVLYILKKKGAAKAALW

>Streptococcus-pneumoniae_SNF52161.1

MKSKRMEFHNKFLIVSAMLAVISWLSLGVVSFPMLAGTLGISTKAAATVVNLISAYSTVTAVISIVGAITGVGSIGSGIAATVLYILKKKGAAKAALW

>Streptococcus-pneumoniae_SNF74231.1

MKSKRMEFHNKFLIVSAMLAVISWLSLGVVSFPMLAGTLGISTKAAATVVNLISAYSTVTAVISIVGAITGVGSIGSGIAATVLYILKKKGAAKAALW

>Streptococcus-pneumoniae_SNJ44317.1

MKSKRMEFHNKFLIVSAMLAVISWLSLGVVSFPMLAGTLGISTKAAATVVNLISAYSTVTAVISIVGAITGVGSIGSGIAATVLYILKKKGAAKAALW

>Streptococcus-pneumoniae_SND49064.1

MKSKRMEFHNKFLIVSAMLAVISWLSLGVVSFPMLAGTLGISTKAAATVVNLISAYSTVTAVISIVGAITGVGSIGSGIAATVLYILKKKGAAKAALW

>Streptococcus-pneumoniae_SNH60243.1

MKSKRMEFHNKFLIVSAMLAVISWLSLGVVSFPMLAGTLGISTKAAATVVNLISAYSTVTAVISIVGAITGVGSIGSGIAATVLYILKKKGAAKAALW

>Streptococcus-pneumoniae_SNO99005.1

MKSKRMEFHNKFLIVSAMLAVISWLSLGVVSFPMLAGTLGISTKAAATVVNLISAYSTVTAVISIVGAITGVGSIGSGIAATVLYILKKKGAAKAALW

>Streptococcus-pneumoniae_SND87795.1

MKSKRMEFHNKFLIVSAMLAVISWLSLGVVSFPMLAGTLGISTKAAATVVNLISAYSTVTAVISIVGAITGVGSIGSGIAATVLYILKKKGAAKAALW

>Streptococcus-pneumoniae_SNM34570.1

MKSKRMEFHNKFLIVSAMLAVISWLSLGVVSFPMLAGTLGISTKAAATVVNLISAYSTVTAVISIVGAITGVGSIGSGIAATVLYILKKKGAAKAALW

>Streptococcus-pneumoniae_SNK18706.1

MKSKRMEFHNKFLIVSAMLAVISWLSLGVVSFPMLAGTLGISTKAAATVVNLISAYSTVTAVISIVGAITGVGSIGSGIAATVLYILKKKGAAKAALW

>Streptococcus-pneumoniae_SNH88738.1

MKSKRMEFHNKFLIVSAMLAVISWLSLGVVSFPMLAGTLGISTKAAATVVNLISAYSTVTAVISIVGAITGVGSIGSGIAATVLYILKKKGAAKAALW

>Streptococcus-pneumoniae_SNL55107.1

MKSKRMEFHNKFLIVSAMLAVISWLSLGVVSFPMLAGTLGISTKAAATVVNLISAYSTVTAVISIVGAITGVGSIGSGIAATVLYILKKKGAAKAALW

>Streptococcus-pneumoniae_SNG60280.1

MKSKRMEFHNKFLIVSAMLAVISWLSLGVVSFPMLAGTLGISTKAAATVVNLISAYSTVTAVISIVGAITGVGSIGSGIAATVLYILKKKGAAKAALW

>Streptococcus-pneumoniae_SNL32637.1

MKSKRMEFHNKFLIVSAMLAVISWLSLGVVSFPMLAGTLGISTKAAATVVNLISAYSTVTAVISIVGAITGVGSIGSGIAATVLYILKKKGAAKAALW

>Streptococcus-pneumoniae_SNG12413.1

MKSKRMEFHNKFLIVSAMLAVISWLSLGVVSFPMLAGTLGISTKAAATVVNLISAYSTVTAVISIVGAITGVGSIGSGIAATVLYILKKKGAAKAALW

>Streptococcus-pneumoniae_SNG63945.1

MKSKRMEFHNKFLIVSAMLAVISWLSLGVVSFPMLAGTLGISTKAAATVVNLISAYSTVTAVISIVGAITGVGSIGSGIAATVLYILKKKGAAKAALW

>Streptococcus-pneumoniae_SNG70465.1

MKSKRMEFHNKFLIVSAMLAVISWLSLGVVSFPMLAGTLGISTKAAATVVNLISAYSTVTAVISIVGAITGVGSIGSGIAATVLYILKKKGAAKAALW

>Streptococcus-pneumoniae_SNE20602.1

MKSKRMEFHNKFLIVSAMLAVISWLSLGVVSFPMLAGTLGISTKAAATVVNLISAYSTVTAVISIVGAITGVGSIGSGIAATVLYILKKKGAAKAALW

>Streptococcus-pneumoniae_SNG79347.1

MKSKRMEFHNKFLIVSAMLAVISWLSLGVVSFPMLAGTLGISTKAAATVVNLISAYSTVTAVISIVGAITGVGSIGSGIAATVLYILKKKGAAKAALW

>Streptococcus-pneumoniae_SNN69333.1

MKSKRMEFHNKFLIVSAMLAVISWLSLGVVSFPMLAGTLGISTKAAATVVNLISAYSTVTAVISIVGAITGVGSIGSGIAATVLYILKKKGAAKAALW

>Streptococcus-pneumoniae_SNE09663.1

MKSKRMEFHNKFLIVSAMLAVISWLSLGVVSFPMLAGTLGISTKAAATVVNLISAYSTVTAVISIVGAITGVGSIGSGIAATVLYILKKKGAAKAALW

>Streptococcus-pneumoniae_SNF62166.1

MKSKRMEFHNKFLIVSAMLAVISWLSLGVVSFPMLAGTLGISTKAAATVVNLISAYSTVTAVISIVGAITGVGSIGSGIAATVLYILKKKGAAKAALW

>Streptococcus-pneumoniae_SNM18831.1

MKSKRMEFHNKFLIVSAMLAVISWLSLGVVSFPMLAGTLGISTKAAATVVNLISAYSTVTAVISIVGAITGVGSIGSGIAATVLYILKKKGAAKAALW

>Streptococcus-pneumoniae_SND31826.1

MKSKRMEFHNKFLIVSAMLAVISWLSLGVVSFPMLAGTLGISTKAAATVVNLISAYSTVTAVISIVGAITGVGSIGSGIAATVLYILKKKGAAKAALW

>Streptococcus-pneumoniae_SNJ06649.1

MKSKRMEFHNKFLIVSAMLAVISWLSLGVVSFPMLAGTLGISTKAAATVVNLISAYSTVTAVISIVGAITGVGSIGSGIAATVLYILKKKGAAKAALW

>Streptococcus-pneumoniae_SNE49446.1

MKSKRMEFHNKFLIVSAMLAVISWLSLGVVSFPMLAGTLGISTKAAATVVNLISAYSTVTAVISIVGAITGVGSIGSGIAATVLYILKKKGAAKAALW

>Streptococcus-pneumoniae_SND92355.1

MKSKRMEFHNKFLIVSAMLAVISWLSLGVVSFPMLAGTLGISTKAAATVVNLISAYSTVTAVISIVGAITGVGSIGSGIAATVLYILKKKGAAKAALW

>Streptococcus-pneumoniae_SNH36755.1

MKSKRMEFHNKFLIVSAMLAVISWLSLGVVSFPMLAGTLGISTKAAATVVNLISAYSTVTAVISIVGAITGVGSIGSGIAATVLYILKKKGAAKAALW

>Streptococcus-pneumoniae_SNJ60734.1

MKSKRMEFHNKFLIVSAMLAVISWLSLGVVSFPMLAGTLGISTKAAATVVNLISAYSTVTAVISIVGAITGVGSIGSGIAATVLYILKKKGAAKAALW

>Streptococcus-pneumoniae_SNH64476.1

MKSKRMEFHNKFLIVSAMLAVISWLSLGVVSFPMLAGTLGISTKAAATVVNLISAYSTVTAVISIVGAITGVGSIGSGIAATVLYILKKKGAAKAALW

>Streptococcus-pneumoniae_SNI68518.1

MKSKRMEFHNKFLIVSAMLAVISWLSLGVVSFPMLAGTLGISTKAAATVVNLISAYSTVTAVISIVGAITGVGSIGSGIAATVLYILKKKGAAKAALW

>Streptococcus-pneumoniae_SND68925.1

MKSKRMEFHNKFLIVSAMLAVISWLSLGVVSFPMLAGTLGISTKAAATVVNLISAYSTVTAVISIVGAITGVGSIGSGIAATVLYILKKKGAAKAALW

>Streptococcus-pneumoniae_SNH69246.1

MKSKRMEFHNKFLIVSAMLAVISWLSLGVVSFPMLAGTLGISTKAAATVVNLISAYSTVTAVISIVGAITGVGSIGSGIAATVLYILKKKGAAKAALW

>Streptococcus-pneumoniae_SNE38591.1

MKSKRMEFHNKFLIVSAMLAVISWLSLGVVSFPMLAGTLGISTKAAATVVNLISAYSTVTAVISIVGAITGVGSIGSGIAATVLYILKKKGAAKAALW

>Streptococcus-pneumoniae_SNE11532.1

MKSKRMEFHNKFLIVSAMLAVISWLSLGVVSFPMLAGTLGISTKAAATVVNLISAYSTVTAVISIVGAITGVGSIGSGIAATVLYILKKKGAAKAALW

>Streptococcus-pneumoniae_SNI96085.1

MKSKRMEFHNKFLIVSAMLAVISWLSLGVVSFPMLAGTLGISTKAAATVVNLISAYSTVTAVISIVGAITGVGSIGSGIAATVLYILKKKGAAKAALW

>Streptococcus-pneumoniae_SNG32146.1

MKSKRMEFHNKFLIVSAMLAVISWLSLGVVSFPMLAGTLGISTKAAATVVNLISAYSTVTAVISIVGAITGVGSIGSGIAATVLYILKKKGAAKAALW

>Streptococcus-pneumoniae_SNL25834.1

MKSKRMEFHNKFLIVSAMLAVISWLSLGVVSFPMLAGTLGISTKAAATVVNLISAYSTVTAVISIVGAITGVGSIGSGIAATVLYILKKKGAAKAALW

>Streptococcus-pneumoniae_SND33666.1

MKSKRMEFHNKFLIVSAMLAVISWLSLGVVSFPMLAGTLGISTKAAATVVNLISAYSTVTAVISIVGAITGVGSIGSGIAATVLYILKKKGAAKAALW

>Streptococcus-pneumoniae_SNL76567.1

MKSKRMEFHNKFLIVSAMLAVISWLSLGVVSFPMLAGTLGISTKAAATVVNLISAYSTVTAVISIVGAITGVGSIGSGIAATVLYILKKKGAAKAALW

>Streptococcus-pneumoniae_SNO46914.1

MKSKRMEFHNKFLIVSAMLAVISWLSLGVVSFPMLAGTLGISTKAAATVVNLISAYSTVTAVISIVGAITGVGSIGSGIAATVLYILKKKGAAKAALW

>Streptococcus-pneumoniae_SNM77298.1
[truncated: 476,474 more chars]
