# Supplementary material for: In Silico Analysis Highlights the Diversity and Novelty of Circular Bacteriocins in Sequenced Microbial Genomes
Source: mSystems. 2020 Jun 2;5(3):e00047-20. doi: 10.1128/mSystems.00047-20 (PMC8534725; doi:10.1128/mSystems.00047-20)
Supplement: TABLE S1 [file msystems.00047-20-st001.doc]

| **Indicator strain** | **Medium^a^** | **Incubation temperature (°C)** | **Antimicrobial activity of supernatant (12 h) of *B. cereus* DDD103^b^** | **Cerecyclin MIC (μM)^c^** |
| --- | --- | --- | --- | --- |
| **Gram-negative bacteria** |  |  |  |  |
| *Escherichia coli* BL21 | LB | 37 | - | - |
| *Escherichia coli* DH5a | LB | 37 | - | - |
| *Salmonella enterica* serotype Paratyphi CMCC50094 | NB | 37 | - | - |
| *Pseudomonas aeruginosa* ATCC27853 | NB | 37 | - | - |
| *Pseudomonas putida Pri3* | NB | 37 | - | - |
|  |  |  |  |  |
| **Gram-positive bacteria** |  |  |  |  |
| *Bacillus cereus* ATCC14579 | LB | 28 | ++ | 0.39 |
| *Bacillus cereus* ATCC49064 | LB | 28 | +++ | 0.29 |
| *Bacillus firmus* DS-1 | LB | 28 | + | 3.13 |
| *Bacillus subtilis* 168 | LB | 37 | ++ | 1.56 |
| *Bacillus thuringiensis* BMB171 | LB | 28 | ++ | 0.78 |
| *Bacillus thuringiensis* YBT-1518 | LB | 28 | ++ | 0.39 |
| *Bacillus amyloliquefaciens* X1 | LB | 37 | + | 6.25 |
| *Bacillus pumilus* SCG I | LB | 37 | - | 12.5 |
| *Staphylococcus aureus* ATCC29213 | NB | 37 | - | - |
| *Staphylococcus aureus* CMCC26003 | NB | 37 | - | - |
| *Listeria monocytogenes* LM201 | TSB | 37 | +++ | 0.39 |
| *Listeria monocytogenes* CMCC54002 | TSB | 37 | +++ | 0.39 |
| *Listeria monocytogenes* ATCC19115 | TSB | 37 | ++ | 0.78 |
| *Enterococcus faecalis* ATCC29212 | NB | 37 | + | 6.25 |
| *Enterococcus faecalis* ATCC51299 | NB | 37 | - | 12.5 |
